# A small-molecule TNIK inhibitor targets fibrosis in preclinical and clinical models

**DOI:** 10.1038/s41587-024-02143-0

**Published:** 2024-03-08

**Authors:** Feng Ren, Alex Aliper, Jian Chen, Heng Zhao, Sujata Rao, Christoph Kuppe, Ivan V. Ozerov, Man Zhang, Klaus Witte, Chris Kruse, Vladimir Aladinskiy, Yan Ivanenkov, Daniil Polykovskiy, Yanyun Fu, Eugene Babin, Junwen Qiao, Xing Liang, Zhenzhen Mou, Hui Wang, Frank W. Pun, Pedro Torres-Ayuso, Alexander Veviorskiy, Dandan Song, Sang Liu, Bei Zhang, Vladimir Naumov, Xiaoqiang Ding, Andrey Kukharenko, Evgeny Izumchenko, Alex Zhavoronkov

**Affiliations:** 1 Insilico Medicine Shanghai Ltd., Shanghai, China; 2 Insilico Medicine AI Limited, Abu Dhabi, UAE; 3 Insilico Medicine Hong Kong Ltd., Hong Kong Science and Technology Park, Hong Kong SAR, China; 4 Department of Clinical Pharmacology, Affiliated Xiaoshan Hospital, Hangzhou Normal University, Hangzhou, China; 5 Insilico Medicine US Inc., New York, NY USA; 6 Institute of Experimental Medicine and Systems Biology, RWTH Aachen University, Aachen, Germany; 7 Department of Nephrology, University Clinic RWTH Aachen, Aachen, Germany; 8 Insilico Medicine Canada Inc, Montreal, Quebec Canada; 9 Department of Cancer and Cellular Biology, Lewis Katz School of Medicine, Temple University, PA USA; 10 Division of Nephrology, Zhongshan Hospital Shanghai Medical College, Fudan University, Shanghai, China; 11 Section of Hematology and Oncology, Department of Medicine, University of Chicago, Chicago, IL USA

**Keywords:** Machine learning, Respiratory tract diseases, Drug development

## Abstract

Idiopathic pulmonary fibrosis (IPF) is an aggressive interstitial lung disease with a high mortality rate. Putative drug targets in IPF have failed to translate into effective therapies at the clinical level. We identify TRAF2- and NCK-interacting kinase (TNIK) as an anti-fibrotic target using a predictive artificial intelligence (AI) approach. Using AI-driven methodology, we generated INS018_055, a small-molecule TNIK inhibitor, which exhibits desirable drug-like properties and anti-fibrotic activity across different organs in vivo through oral, inhaled or topical administration. INS018_055 possesses anti-inflammatory effects in addition to its anti-fibrotic profile, validated in multiple in vivo studies. Its safety and tolerability as well as pharmacokinetics were validated in a randomized, double-blinded, placebo-controlled phase I clinical trial (NCT05154240) involving 78 healthy participants. A separate phase I trial in China, CTR20221542, also demonstrated comparable safety and pharmacokinetic profiles. This work was completed in roughly 18 months from target discovery to preclinical candidate nomination and demonstrates the capabilities of our generative AI-driven drug-discovery pipeline.

## Abstract (web summary)

An AI-generated small-molecule inhibitor treats fibrosis in vivo and in phase I clinical trials.

## Main

Therapeutic target identification is a crucial step of the drug-discovery pipeline for all disease processes, including fibrosis. Erroneous targets selected at the early stage of drug development may result in a costly drug-discovery program, often ending in failure during phase II trials years later^1,2^. As a result, large pharmaceutical companies may behave as though they are risk averse, reducing their willingness to invest in potentially valuable targets and therapeutic strategies^3^. Although development of precise data-driven approaches for drug target discovery has a critical impact on the success of clinical trials, this task still has recognized limitations such as data complexity and batch effects^4,5^. More recently, AI-driven approaches have demonstrated efficacy in discovering target candidates^6^ in the contexts of embryonic–fetal transition^7^ and muscle aging^8^. Advanced pathway analysis and AI algorithms applied to multiomics data can identify targets and biomarkers even when prior evidence is sparse^9,10^. The success of multiomic target-discovery systems has been demonstrated in cancer and age-associated diseases^11–15^. Fibrosis occurs in the final stage of chronic organ deficiencies, such as pulmonary, kidney or liver diseases and is characterized by excessive proliferation of matrix-producing cells that arise from dysregulated chronic inflammation triggered by infective, chemical, autoimmune or radioactive tissue damage^16,17^. IPF is a gradually advancing and generally lethal disease, characterized histologically by fibroblast proliferation and substantial extracellular matrix deposition^18,19^. Myofibroblasts play a crucial role in the fibrotic process, with transforming growth factor (TGF)-β being a major contributor to myofibroblast differentiation^20^. IPF is most prevalent in patients over 60 years of age^21^, and its estimated incidence in the USA was reported to be 6.8 per 100,000 person-years^21^. Given the increasing incidence, the cost of treating patients with IPF poses a substantial burden on healthcare systems and represents a growing public health problem worldwide^21–25^.

In untreated patients, IPF has a highly inconsistent clinical course, with a median survival of 2 to 3 years^26^. Less than 30% of patients with IPF benefit from administration of corticosteroids, with adverse effects reducing patients’ quality of life^27^. Nevertheless, most of these patients succumb to respiratory failure or progressive decline in lung function^28^. Current targeted treatment options for IPF are limited to nintedanib and pirfenidone^29^. Following their FDA approval in 2014, nintedanib and pirfenidone are recommended by the American Thoracic Society, the European Respiratory Society, the Japanese Respiratory Society and the Latin American Thoracic Association for treating IPF^30^. Of these drugs, pirfenidone suppresses TGF-β expression^31^, whereas nintedanib exerts its efficacy in IPF by inhibiting fibroblast growth factor receptor (FGFR), platelet-derived growth factor receptor (PDGFR) and vascular endothelial growth factor (VEGFR)^32^, that is, growth factor receptors in the fibrotic pathway of tyrosine kinase structure, with FGFR being most relevant^33^.

As with IPF, renal fibrosis also correlates with organ failure^34^. Renal fibrosis is histomorphologically identified by tubulointerstitial fibrosis and is the final manifestation of chronic kidney disease (CKD)^35^. The incidence of CKD is increasing worldwide and continues to grow within aged populations^36^. Thus, developing an effective anti-fibrotic medicine that attenuates renal fibrosis remains a large, unmet clinical need. Like IPF, renal fibrosis develops following prolonged exposure to ischemic conditions or inflammatory insults^37^. Increased TGF-β signaling within stromal cell types in the tubule–interstitial space appears to be central to the initiation of fibrosis^38,39^. No specific fibrosis inhibitor has passed clinical trials for the treatment of CKD.

Given the scarcity of anti-fibrotic therapies for IPF or CKD, we used our published generative AI pipeline^14,15,40–42^ to address this clinical need. The process of drug development, from target identification to preclinical testing and ultimately to clinical validation, can take over a decade. In this study, our generative AI platform has identified TNIK inhibition as a potent anti-fibrotic strategy and assisted in developing a highly specific TNIK inhibitor, INS018_055. We have synthesized this compound and demonstrated its selective, anti-fibrotic activity in multiple murine and rat models of fibrosis. We report phase I clinical trial data highlighting the safety and tolerability of our small-molecule inhibitor. This comprehensive approach was completed in roughly 18 months from target discovery to preclinical candidate nomination and demonstrates the capabilities of our generative AI-driven drug-discovery pipeline.

## Results

### AI-discovered anti-fibrotic targets

**Fig. 1:**
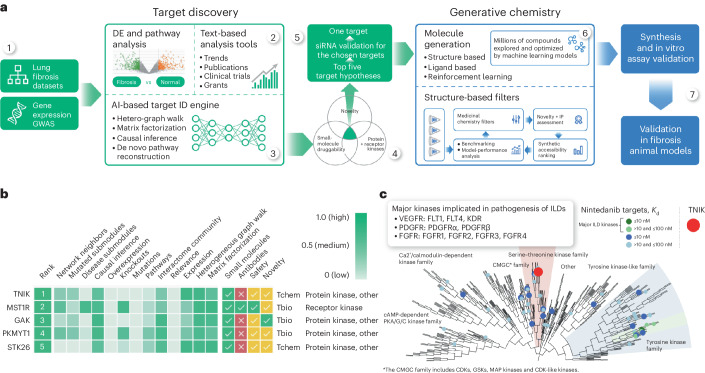
AI-augmented pipeline for target discovery. **a**, The PandaOmics target-discovery platform was applied to lung and kidney fibrosis datasets to generate target hypotheses, followed by the Chemistry42 platform application to generate small-molecule leads targeting TNIK. DE, differential gene; GWAS, genome-wide association study; hetero, heterogeneous; siRNA, small interfering RNA; IP, intellectual property. **b**, TNIK was scored the number 1 candidate using protein and receptor kinase PandaOmics settings based on relatively high values of network neighbors, mutated submodules, causal inference, pathways, interactome community, expression, heterogeneous graph walk and matrix factorization scores. GAK, cyclin G-associated kinase; MST1R, macrophage-stimulating 1 receptor; PKMYT1, protein kinase, membrane-associated tyrosine–threonine 1; STK26, serine–threonine kinase 26; Tchem, genes whose products can be targeted with small molecules better than the following bioactivity cutoff values: 30 nM for kinases, 100 nM for GPCRs and nuclear receptors, 10 μM for ion channels, and 1 μM for other target classes; Tbio, genes annotated with a Gene Ontology Molecular Function or Biological Process with an Experimental Evidence code, or targets with confirmed OMIM phenotype(s), or do not satisfy the Tdark criteria. **c**, TNIK is a member of the serine–threonine kinase STE20 family. This family does not contain any major targets of anti-fibrotic medications including nintedanib, the most prominent kinase inhibitor used for IPF treatment. This illustrates the relative novelty of the target. cAMP, cyclic AMP; FLT, FMS-related receptor tyrosine kinase 1; GSK, glycogen synthase kinase; KDR, kinase insert domain receptor; MAP, mitogen-activated protein; PKA, protein kinase A; ILD, interstial lung disease; *K*_d_, dissociation constant.

Here we used PandaOmics, a commercially available target-discovery platform using multiple AI engines including generative pretrained transformers^14^, for target identification in fibrosis (Fig. 1a). We included a list of multiomics datasets derived from tissue samples of patients with IPF as a basis for the computational pipeline (Fig. 1a, step 1). This pipeline combines several distinct but complementary computational approaches, all of which are similar in terms of output (a ranked list of genes, ordered by most-promising target) but different in terms of the input data. In addition to multiomics datasets, we included methods relying on biological network analysis and text data from scientific literature (Fig. 1a, step 2) with node degree bias control. These data types facilitate target hypotheses inferred from omics into the context of prior evidence, which include clinical trials, publications and grant applications. The text data are temporal in nature, allowing PandaOmics to not only capture trends but even project them into the future (Fig. 1a, step 3).

Unlike other target-discovery approaches such as Open Targets^43^ or NewDrugTargets^44^, our method was designed and validated to search targets for multiple diseases and aging^15^. Thus, we developed a time machine approach, in which we trained the computational models using data published before a certain time point and validated the model outputs by their ability to predict those targets that came into the focus of the pharmaceutical industry after this time point. Several distinct AI-driven target-discovery philosophies, including random walk on heterogeneous graphs and negative matrix factorization, were applied (Supplementary Video 1).

**Extended Data Fig. 1:**
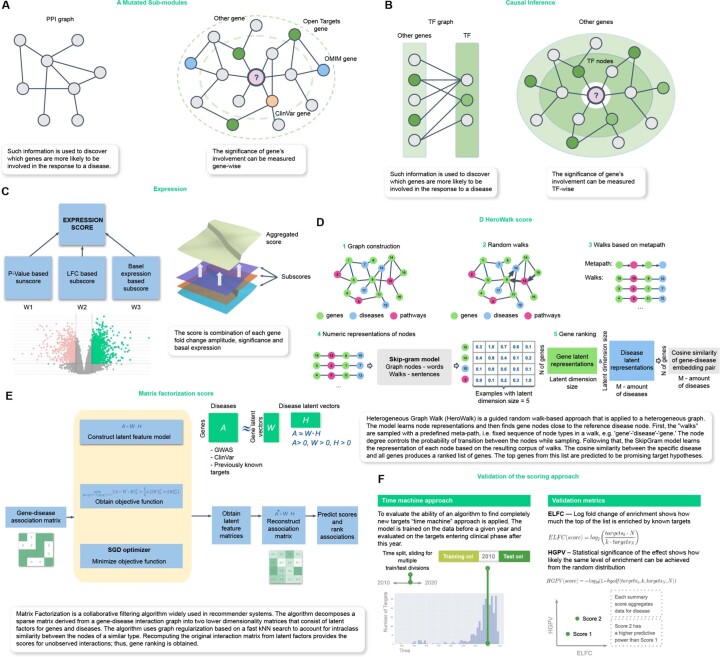
Target ID Approach. **(a)** Mutated sub-modules score scheme. The significance of gene’s involvement can be measured gene-wise. Colored nodes are genes associated with the disease of interest genes according to OMIM (green), ClinVar (orange), Open Targets (blue). **(b)** Causal inference score scheme. The significance of gene’s involvement can be measured TF-wise. **(c)** Expression score scheme. The Expression score is combination of each gene fold change amplitude, significance, and basal expression. **(d)** HeroWalk score scheme. HeroWalk is a guided random walk-based approach that is applied to a heterogeneous graph. The model learns node representations and then finds gene nodes close to the reference disease node. First, the ‘walks’ are sampled with a predefined meta-path, that is fixed sequence of node types in a walk, for example ‘gene’-‘disease’-‘gene.’ The node degree controls the probability of transition between the nodes while sampling. Following that, the SkipGram model learns the representation of each node based on the resulting corpus of walks. The cosine similarity between the specific disease and all genes produces a ranked list of genes. The top genes from this list are predicted to be promising target hypotheses. **(e)** Matrix factorization scheme. Matrix Factorization is a collaborative filtering algorithm widely used in recommender systems. The algorithm decomposes a sparse matrix derived from a gene-disease interaction graph into two lower-dimensionality matrices that consist of latent factors for genes and diseases. The algorithm uses graph regularization based on a fast kNN search to account for intraclass similarity between similar nodes. Recomputing the original interaction matrix from latent factors provides the scores for unobserved interactions; thus, gene ranking is obtained. **(f)** Performance of the models are evaluated using two metrics ELFC and HGPV. Time Machine approach was applied to demonstrate the ability of the models to predict truly novel target hypotheses.

Our platform generated a ranked list of targets after applying a combination of score composition and filters, including disease-agnostic properties like protein family, accessibility by small molecules or therapeutic antibodies, novelty and crystal structure availability, among others (Extended Data Fig. 1). To generate the target hypotheses for fibrosis, we applied the ‘protein and receptor kinase’ scenario to fibrosis-related datasets. It includes scores reflecting the network component of the disease mechanisms combined with the transcriptional factor enrichment-based causal inference (Supplementary Information 1). Protein class (protein and receptor kinases only), novelty and small-molecule druggability filters were enabled in the downstream processing steps (Fig. 1a, step 4).

TNIK was identified as the number 1 target among the five top candidates using the ‘protein and receptor kinase’ approach with relatively high values of network neighbors, causal inference, pathways, interactome community, expression, heterogeneous graph walk and matrix factorization scores (Fig. 1a, step 5, Fig. 1b). TNIK has been associated with fibrosis-driving pathways including WNT^45,46^, TGF-β^46,47^, Hippo (yes-associated protein (YAP)–transcriptional coactivator with PDZ-binding motif (TAZ))^46,48^, JNK^46,49,50^ and nuclear factor (NF)-κB^46,50^ signaling. However, TNIK has not been studied as a therapeutic target in IPF and was thus selected by the AI algorithm. Whereas tyrosine kinase inhibitors like nintedanib, imatinib and nilotinib have been tested in IPF, the role of serine–threonine kinases (such as TNIK) in IPF remains largely uninterrogated (Fig. 1c). As fibrosis can affect any organ and is responsible for up to 45% of all deaths in the industrialized world^51^, we hypothesized that inhibition of TNIK may provide therapeutic benefit in multiple pathologies in which fibrosis may be implicated. In agreement with this hypothesis, TNIK was independently predicted as a disease target associated with multiple hallmarks of aging^15^ and as a regulator of lipid metabolism in aged and/or high-fat diet-fed mice^52^. These findings support the hypotheses of our AI-driven pipeline (Supplementary Information 2).

**Extended Data Fig. 2:**
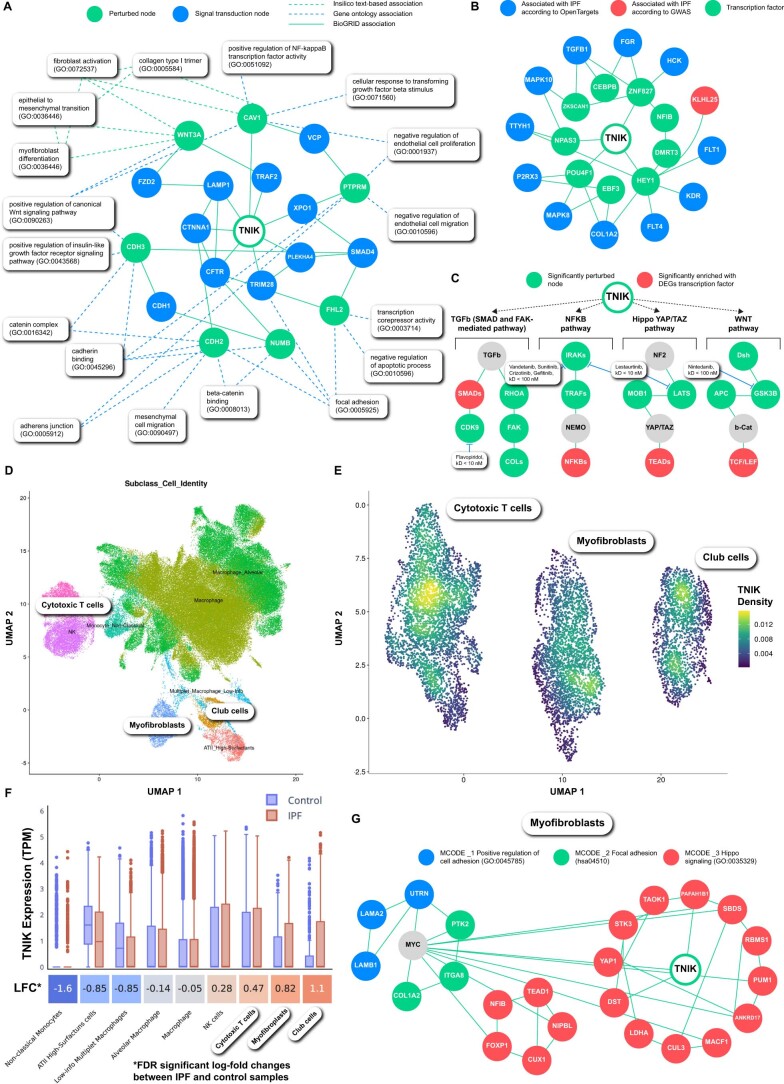
PandaOmics scores transparency and in-silico validation of TNIK. **(a)** High values of the graph-based scores can be explained by the significantly perturbed genes observed in the TNIK interactome community neighborhood formed by proteins implicated in IPF and other fibrotic diseases. **(b)** Causal inference score relies on transcriptional factor inference. Majority of the genes regulated by the transcriptional factors are associated with IPF according to OpenTargets and GWAS studies. **(c)** TNIK regulates major signaling pathways known to be causal for the development of fibrotic conditions. **(d)** Clustering of cells derived from single cell RNA-seq data for IPF and healthy lung tissue. **(e)** Visualization of TNIK gene-weighted density in myofibroblasts, cytotoxic T cells and club cells using Nebulosa package. **(f)** Single cell gene expression profiles of IPF lung tissue are presented in boxplots for various unique cell types: Non-classical Monocytes (n = 1058, 1931), ATII High-Surfactuns cells (n = 2655, 496), Low-info Multiplet Macrophages (n = 1000, 2765), Alveolar Macrophage (n = 23905, 27403), Macrophage (n = 40747, 60483), NK cells (n = 2744, 4007), Cytotoxic T cells (n = 4061, 6296), Myofibroblasts (n = 204, 2886), and Club cells (n = 226, 1855) for the control and IPF groups, respectively. These profiles show high expression of TNIK in the key cell types responsible for disease progression: myofibroblasts, cytotoxic T cells, and club cells. The differential expression between IPF and controls for each unique cell type was calculated using the tl.rank_genes_groups function of scanpy package (method = 'Wilcoxon', two-sided), and the resulting log-fold changes (with p-values adjusted using the Benjamini-Hochberg method and threshold = 0.05, FDR) between the IPF and control groups for each cell type of TNIK were plotted on a heatmap. The center lines in the boxplots indicate the medians, while the box limits represent the 25th and 75th percentiles. (**g**) Simulated knockout profile of TNIK in myofibroblasts derived from IPF patients by scTenifoldKnk virtual knockout tool confirms the importance of Hippo and YAP/TAZ signaling in the TNIK-mediated regulation of the major IPF-related pathways.

We next performed a transparency analysis of the PandaOmics scores. The interactome community transparency revealed that TNIK inhibition is connected to multiple biological processes known to be important for fibrosis progression such as focal adhesion signaling, myofibroblast differentiation and mesenchymal cell migration (Extended Data Fig. 2a). Causal inference transparency revealed that TNIK is tightly connected with genes associated with IPF, including *TGFB1*, *FGR*, *FLT1*, *KDR* and others (Extended Data Fig. 2b). Finally, using an AI-powered de novo pathway-reconstruction tool, we showed that TNIK activates previously described pathways that are associated with IPF. These gene sets are regulated by downstream transcriptional factors including TCF–LEF, SMAD, NF-κB and TEAD families (Extended Data Fig. 2c).

Validation of TNIK association with IPF using the single-cell gene expression dataset from unaffected lung and patients with IPF^53^ demonstrates that *TNIK* expression in cytotoxic T cells, myofibroblasts and club cells is higher in fibrotic tissue than in unaffected controls (Extended Data Fig. 2d–f). This finding confirms TNIK’s potential role in regulating the function of key cells involved in IPF development. We used this single-cell dataset to simulate *TNIK* knockout in IPF myofibroblasts using the scTenifoldKnk approach^54^ followed by the Molecular Complex Detection (MCODE) algorithm^55^ and performed pathway and biological process enrichment analysis. This analysis revealed that inhibiting TNIK primarily activates Hippo signaling and, consequently, downregulates YAP–TAZ (Extended Data Fig. 2g). Thus, TNIK is an attractive target for lung fibrosis, supported by both the unique omic-driven analysis and other AI-based approaches.

### Generative AI-designed TNIK inhibitors

To identify TNIK inhibitors, we exploited available crystal structures of the TNIK kinase domain^56^ and the Chemistry42 structure-based drug-design AI workflow (Fig. 1a, step 6)^40^. As using ATP competitors is a well-established strategy for targeting kinases^57^, we selected the ATP-binding site as a pocket for compound generation. The AI-driven platform was configured to produce small-molecule structures capable of forming hydrogen bonds with the Cys108-NH of the TNIK hinge region. Targeting less-conserved adjacent allosteric pockets (such as a hydrophobic back cavity close to the gatekeeper residue) in addition to the active site can achieve better selectivity of the lead compounds^58^. As such, an additional hydrophobic pharmacophore point was applied to prioritize structures bearing hydrophobic functions to deeply occupy the back cavity formed by Met105, Leu73, Leu103, Ala52 and Val104.

The compounds, selected based on synthetic accessibility, novelty and medicinal chemistry properties, were synthesized and tested using a radiometric enzymatic assay (Fig. 1a, step 7). The first rounds of screening revealed a series of TNIK inhibitors with nanomolar-level binding affinity. However, in vitro absorption, distribution, metabolism and excretion profiling of the primary lead compounds revealed their high clearance in human and mice liver microsomes, cytochrome p450(CYP) inhibition at a half maximal inhibitory concentration (IC_50_) less than 10 µM and kinetic solubility less than 2 µM (Supplementary Information 3). The lead optimization stage, which prioritized an improvement in the absorption, distribution, metabolism and excretion profile of this class of compounds, resulted in INS018_055 (WO2022179528A1) (Supplementary Information 3 and 4).

**Fig. 2:**
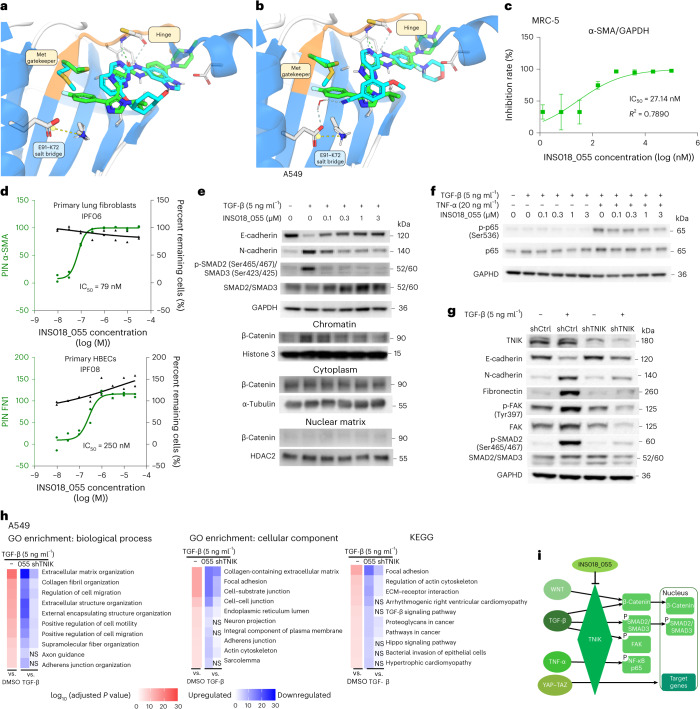
Architectural superposition of TNIK inhibitor structures with the predicted INS018_055-binding mode and effects on TGF-β-induced EMT and FMT cellular programs. **a**, Crystal structure of the NCB-0846 (cyan)-bound TNIK kinase domain (PDB 5D7A) aligned with the predicted binding mode of INS018_055 (green). **b**, Crystal structure of the compound 9 (cyan)-bound TNIK kinase domain (PDB 5AX9) aligned with the predicted binding mode of INS018_055 (green). Differences in Met gatekeeper orientations between inhibitors bound to the TNIK kinase domain are depicted, with Met side chain color corresponding to ligand color (cyan, green). The hinge region is shaded orange. Key ligand interactions are marked with dashed lines in the ligand color (cyan, green). The conservative E91–K72 salt bridge is shown as a yellow dashed line. Ligands and key pocket residues are represented as sticks. **c**, Inhibitory effect of INS018_055 on TGF-β-induced α-SMA protein expression in MRC-5 cells. *n* = 3, mean ± s.d. GAPDH, glyceraldehyde 3-phosphate dehydrogenase. **d**, Top, representative INS018_055-inhibitory effect (green) on FMT in primary human lung fibroblasts by measuring α-SMA. Bottom, INS018_055-inhibitory effect (green) on EMT in primary human bronchial epithelial cells (HBECs) by measuring fibronectin. Percent remaining cells (black) reflects nuclear count, which is a measurement of cell percentage without nuclear loss (*n* = 2 experimental replicates). PIN, percentage inhibition. **e**, Representative western blots showing changes in E-cadherin, N-cadherin, SMAD2/SMAD3, phospho (p)-SMAD2/SMAD3 and β-catenin in different A549 cell fractions following TGF-β stimulation and treatment with INS018_055. HDAC2, histone deacetylase 2. **f**, Representative western blot showing the inhibitory effect of INS018_055 on phosphorylated and total NF-κB p65 in TGF-β- and TNF-α-stimulated A549 cells (*n* = 3 biological replicates). **g**, Representative western blots showing changes in E-cadherin, N-cadherin, fibronectin, phospho-FAK and phospho-SMAD2 induced by treatment of A549 cells with TNIK shRNA (shTNIK) (shTNIK-4). shCtrl, control shRNA. **h**, Significantly upregulated pathways induced by TGF-β treatment and restoration by INS018_055 (055) or shTNIK-1. Gene ontology (GO) enrichment (left, middle) and KEGG analysis (right). *n* = 3 in each condition. Enrichment analysis was performed using the gseapy.enrichr Python package. *P* values were computed using Fisher’s exact test (one-tailed hypergeometric test). Adjusted *P* values (*q* values) were calculated using the Benjamini–Hochberg method for correction for multiple-hypothesis testing. ECM, extracellular matrix; NS, not significant. **i**, Scheme showing TNIK function in regulating TGF-β, WNT, YAP–TAZ and TNF-α pathways as identified by in vitro perturbation experiments. Source data

The carboxyl oxygen of INS018_055 forms a hydrogen bridge with Cys108-NH within the hinge region (Fig. 2a). Hydrogen bonding between the amide NH and the nitrogen in the proximal imidazole stabilizes a planar conformation. Moreover, the NH of this imidazole can form a hydrogen bond with the side chain of the gatekeeper Met105, as the distance of ~3.2 Å between the NH and the sulfur is suitable for bond formation. The second imidazole projects the *p*-fluorophenyl into the back pocket and the isopropyl toward Asn158 and Gln157. The *p*-fluorophenyl is not only properly accommodated within the back cavity but is also coplanar to the C-S-C of Met105 (the distance from the S to the closest C of the ring is 3.8 Å).

**Extended Data Fig. 3:**
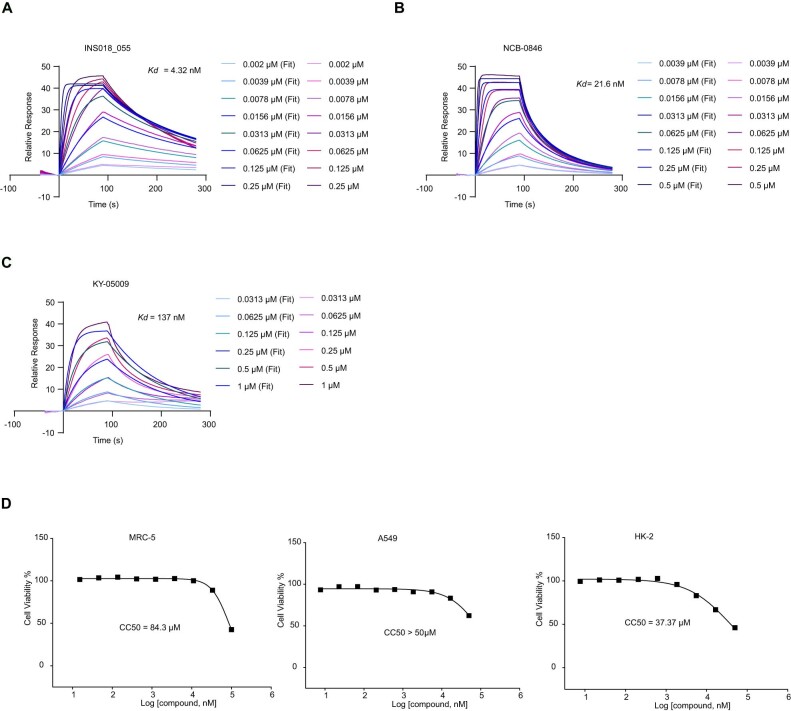
INS018_055 target affinity and cell viability analysis. Surface plasmon resonance (SPR) assay to measure the binding kinetics of (**a**)INS018_055, (**b**) NCB-0846 and (**c**) KY-05009 to His tagged TNIK (9-315). (**d**) Cell viability analysis performed on MRC-5 cells **(left)**, A549 cells **(middle)** and HK-2 cells **(right)**. Cells were seeded at 8,000, 5,000 and 8,000 cells/well in 96-well plate, respectively and incubated with INS018_055 for 72 hours. Cell viability was measured by Cell Titer Glo. All experiments were performed in duplicate wells. CC_50_ was calculated with the data of cell viability (%). To determine the CC_50_ value, the data was fitted using an equation for a sigmoidal dose response (variable slope), as provided by GraphPad™ Prism software. The equation was shown as: Y = Bottom + (Top-Bottom)/(1+10^((LogIC_50_-X)*HillSlope)). Source data

The X-ray structures of two early TNIK inhibitors, NCB-0846 and 4-methoxy-3-(2-(3-methoxy-4-morpholinophenylamino)pyridin-4-yl)benzonitrile (compound 9), in complex with the target were reported previously^56^. These compounds bind to the ATP-binding site and interact with the backbone of Cys108 at the hinge region via two hydrogen bonds (Fig. 2a,b). Crystal structures reveal that the side chain of the gatekeeper Met105 covers back pocket entry, such that these compounds do not contain functions capable of shifting this residue. Unlike NCB-0846, the cyano group of compound 9 interacts with the backbone of Phe172 through a water molecule close to the cavity and contacts Met105. By contrast, INS018_055 is predicted to occupy the back pocket deeply, forming a unique interaction among known TNIK inhibitors. To evaluate the binding affinity of INS018_055, we performed a surface plasmon resonance assay. INS018_055 showed potent affinity (*K*_d_ value of 4.32 nM) relative to two other known TNIK inhibitors (Extended Data Fig. 3a–c).

### Selectivity of INS018_055

We next used a KinaseProfiler panel to assess the kinase selectivity of 10 μM INS018_055. The activity of protein kinases (excluding ATM and DNA-PK) was evaluated in a radiometric format, whereas lipid kinases as well as ATM, DNA-PK and ATR-ATRIP (ataxia telangiectasia-mutated and Rad3-related; ATR-interacting protein) were evaluated by homogeneous time-resolved fluorescence. Subsequently, 42 top-hit kinases were further evaluated to determine the INS018_055 IC_50_. Our dose-dependent study revealed that TNIK was the most inhibited target with an IC_50_ of 31 nM (Supplementary Information 5). Notably, most of the kinases that achieved an IC_50_ at nanomolar (<1 μM) concentrations of INS018_055 have been reported to regulate fibrosis-driving processes, such as ALK4, TGFBR1 and DDR1 (Supplementary Information 5). These results indicate that our lead compound is selective at inhibiting TNIK at nanomolar concentrations and demonstrates a broader affinity toward targeting fibrosis-related kinases.

A published selectivity assay of NCB-0846 using a 50-kinase panel revealed that eight kinases achieved an IC_50_ < 100 nM^56^. INS018_055 inhibited six of these kinases with IC_50_ values at substantially higher concentrations (Supplementary Information 5). Although direct comparison of the data generated by different studies is difficult (even though both assays use *K*_m_ values for ATP concentration), these observations are suggestive of superior INS018_055 selectivity (at least among the reported kinases) associated with its different binding mode (particularly the occupation of the TNIK back pocket).

### INS018_055 ameliorates TGF-β-induced EMT and FMT signaling

**Extended Data Fig. 4:**
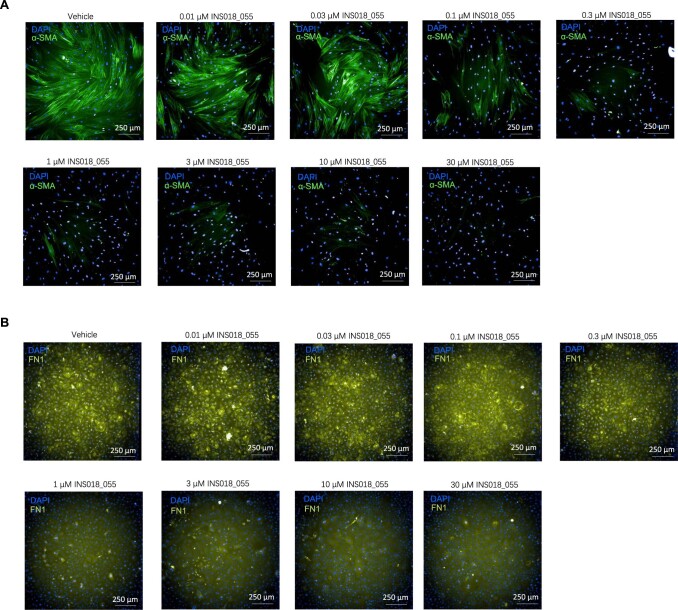
IPF donor fibroblast and epithelial cell imaging for FMT and EMT assays. (**a**) Representative images of fibroblasts from a donor IPF patient stained with DAPI and alpha-smooth muscle actin (α-SMA) antibody. Cells were pre-treated with TGFβ to induce mesenchymal transition after which cells were treated with the indicated concentrations of INS018_055. Automated quantitation of α-SMA fluorescence intensity and cell density was made following INS018_055 treatment. (**b**) Representative images of fibroblasts from a donor IPF patient stained with DAPI and fibronectin (FN1) antibody. Cells were pre-treated with TGFβ to induce mesenchymal transition after which cells were treated with the indicated concentrations of INS018_055. Automated quantitation of FN1 fluorescence intensity and cell density was made following INS018_055 treatment. Fibroblasts and epithelial cells were isolated from 3 IPF donor tissue samples and 3 healthy donor samples. The experiment was carried out with all 6 independent samples in a single experiment by Charles River’s high throughput imaging pipeline. Source data is provided.

Epithelial-to-mesenchymal transition (EMT) and fibroblast-to-myofibroblast transition (FMT) cellular programs drive fibrosis^59^ in a TGF-β-directed process^60–62^. To validate the predicted anti-fibrotic role of TNIK, we treated a lung fibroblast cell line (MRC-5) with INS018_055, which showed a dose-dependent reduction in TGF-β-induced expression of α-smooth muscle actin (α-SMA) protein (IC_50_ = 27.14 nM) (Fig. 2c). A much higher concentration of INS018_055 (50% cytotoxic concentration (CC_50_ ) =84.3 μM) was required to suppress MRC-5 cell viability (Extended Data Fig. 3d). We also tested the effect of INS018_055 on TGF-β-stimulated FMT by measuring α-SMA levels in primary lung fibroblasts from three patients with IPF and three healthy donors (Fig. 2d). Sequentially diluted INS018_055 led to complete and concentration-dependent inhibition of TGF-β-mediated α-SMA expression in the fibroblasts of three donors with IPF, with IC_50_ values of 50 nM, 79 nM and 63 nM (Fig. 2d and Supplementary Information 6). In three healthy donors’ fibroblasts, TGF-β-mediated α-SMA expression was reduced by INS018_055, albeit at higher concentrations (IC_50_ of 79 nM, 200 nM and 320 nM) than in donors with IPF (Supplementary Information 6), suggesting a greater potency of INS018_055 in IPF. Importantly, treatment of donor cell lines with INS018_055 did not induce substantial cytotoxicity as measured by nuclear frequency (Fig. 2d). Finally, representative images of the EMT and FMT assays carried out with donor IPF and healthy fibroblasts align perfectly with the automated quantitation of fibrosis-associated proteins (α-SMA and fibronectin 1 (FN1)) and cell density (Extended Data Fig. 4).

The effect of INS018_055 on EMT was assessed by changes in FN1 in human primary bronchial epithelial cells from patients with IPF and healthy donors. The IC_50_ values of INS018_055 in donors with IPF were 250 nM, 320 nM and 400 nM, whereas IC_50_ values for nintedanib were substantially higher (1,600 nM, 6,300 nM and 7,900 nM, respectively) (Supplementary Information 6). The IC_50_ values of INS018_055 were 63 nM, 63 nM and 500 nM in cells from healthy individuals (Supplementary Information 6), comparable to the IC_50_ value of the reference compound SB5252334. No loss of nuclei was observed in either donors with IPF or healthy donors, indicating low cytotoxicity.

**Extended Data Fig. 5:**
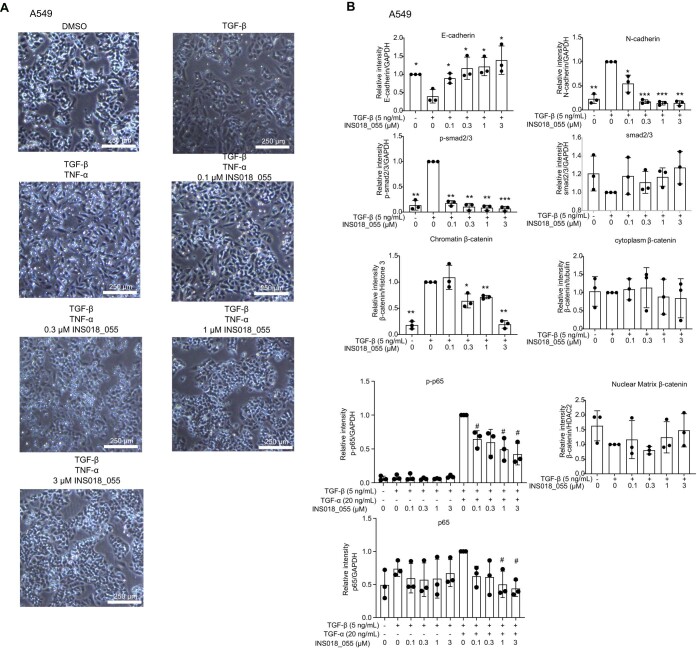
Effect of IN18_055 on TGF-β-induced, or combination of TGF-β and TNF-α induced EMT/FMT cellular programs. **(a)** Representative pictures of morphology change of A549 cells. **(b)** Bar graphs (mean ± SD) of inhibition effect of INS018_055 on TGF-β induced changes of E-cadherin, N-cadherin, phospho-smad2, smad2/3 and beta-catenin levels in A549 cells; Bar graphs (mean ± SD) of inhibition effect of INS018_055 on TGF-β and TGF-α-induced phospho-NF-kb p65 and its total protein changes in A549 cells. Images of blots refers to Fig. 2e, f. Individual values are shown in dots. P values are analyzed by Welch’s t-test (two-sided) among 3 independent experiments. p values < 0.05 were considered statistically significant (*: significant compared to group treated with TGF-β but without INS018_055; #: significant compared to group treated with TGF-β and TGF-α but without INS018_055). Compared to TGF-β control group, group (no TGF-β), groups with INS018_055 (0.1, 0.3, 1 and 3 μM) showed p values (E-cadherin) of 0.0302, 0.024, 0.0288, 0.0127 and 0.031; p values (N-cadherin) of 0.0054, 0.0469, 0.0008, 0.0007 and 0.0012; p values (p-smad2/3) of 0.004, 0.0012, 0.0018, 0.001 and 0.0008; p values (chromatin β-catenin) of 0.5714, 0.0436, 0.0043 and 0.0028. Compared to TGF-β and TGF-α control group, groups with INS018_055 (0.1, 0.3, 1 and 3 μM) showed p values (p-p65) of 0.0383, 0.0675, 0.0341 and 0.0242; p values (p65) of 0.0503, 0.1243, 0.0492 and 0.0151. Source data

**Extended Data Fig. 6:**
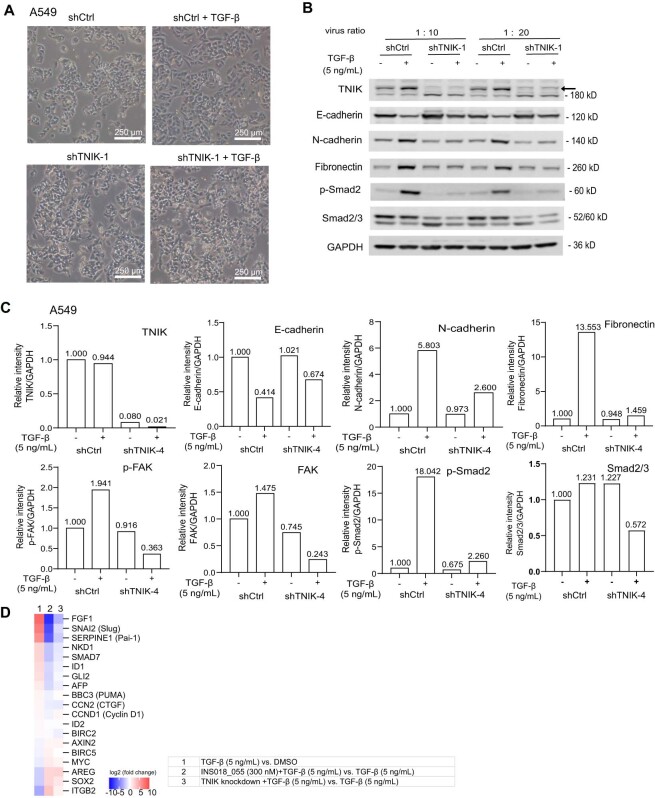
Effect of TNIK deletion on TGF-β signaling in A549 cells. **(a)** Representative pictures of morphology change of A549 cells. Single independent experiment- no statistical comparisons were made. **(b)** Western Blot images of inhibition of TGF-β induced changes of E-cadherin, N-cadherin, fibronectin, and phospho-smad2 by shTNIK (shTNIK-1). Single independent experiment- no statistical comparisons were made. **(c)** Bar graphs of Fig. 2g, the inhibition of TGF-β induced changes of E-cadherin, N-cadherin, fibronectin, phospho-FAK and phospho-smad2 by shTNIK (shTNIK-4). **(d)** Heatmap of log2(fold changes) of gene expression change of Hippo signaling related proteins. Source data

We next sought to compare these observations to a known TNIK inhibitor. The molecule KY-05009 was reported to attenuate TGF-β-induced EMT in the human lung adenocarcinoma cell line A549 (ref. ^46^). Treatment of these epithelial cells with TGF-β induced a fibroblast-like spindle-shape phenotype, which was further enhanced by cotreatment with tumor necrosis factor (TNF)-α (Extended Data Fig. 5a). INS018_055 reversed the TGF-β-induced morphology change that was paralleled by downregulation of N-cadherin and phospho-SMAD2/SMAD3 transcription factor complex levels as well as upregulation of E-cadherin expression in a dose-dependent manner (Fig. 2e and Extended Data Fig. 5b). Furthermore, INS018_055 suppressed the level of phosphorylated p65 protein induced by TGF-β and TNF-α cotreatment (Fig. 2f and Extended Data Fig. 5b). The WNT–β-catenin signaling pathway is involved in wound healing, regulates fibrogenesis and is known to contribute to EMT elicited by TGF-β stimulation^63^. TGF-β-induced relocalization of β-catenin to chromatin was reduced by INS018_055 treatment in a dose-dependent manner, with no significant effect on cytoplasm or nuclear matrix protein levels (Fig. 2e and Extended Data Fig. 5b). These observations suggest that INS018_055 may suppress β-catenin activation by inhibiting its DNA-binding activity. Short hairpin RNA (shRNA)-mediated depletion of TNIK attenuated TGF-β-induced EMT, as evidenced by lower expression of fibronectin, N-cadherin, phospho-SMAD2, phospho-focal adhesion kinase (FAK) and total FAK relative to cells treated with control shRNA. Furthermore, E-cadherin expression was increased following TNIK depletion as compared to the control group (Fig. 2g and Extended Data Fig. 6a–c). As expected, bulk RNA-seq of A549 cells treated with TGF-β revealed an upregulation of transcriptional processes associated with extracellular matrix organization, cell–cell junctions, focal adhesions and collagen fibril organization, while treatment with INS018_055 significantly reverted these transcriptional changes (Fig. 2h). Notably, TNIK knockdown also resulted in transcriptional changes akin to those induced by INS018_055 treatment. Furthermore, Kyoto Encyclopedia of Genes and Genomes (KEGG) analysis revealed a significant enhancement of Hippo signaling in cells treated with TGF-β (Fig. 2h and Extended Data Fig. 6d). Transcripts of Hippo pathway target genes, including those encoding known regulators of fibrosis such as PAI-1, SMAD7, CTGF^64^, GLI2 (ref. ^65^) and PUMA^66^, were downregulated in cells pretreated with INS018_055. These data indicate that INS018_055 effectively inhibits key fibrotic pathways such as WNT–β-catenin, TGF-β–SMAD2, TNF-α–NF-κB and YAP–TAZ signaling (Fig. 2i).

### INS018_055 attenuates murine bleomycin-induced lung fibrosis

**Fig. 3:**
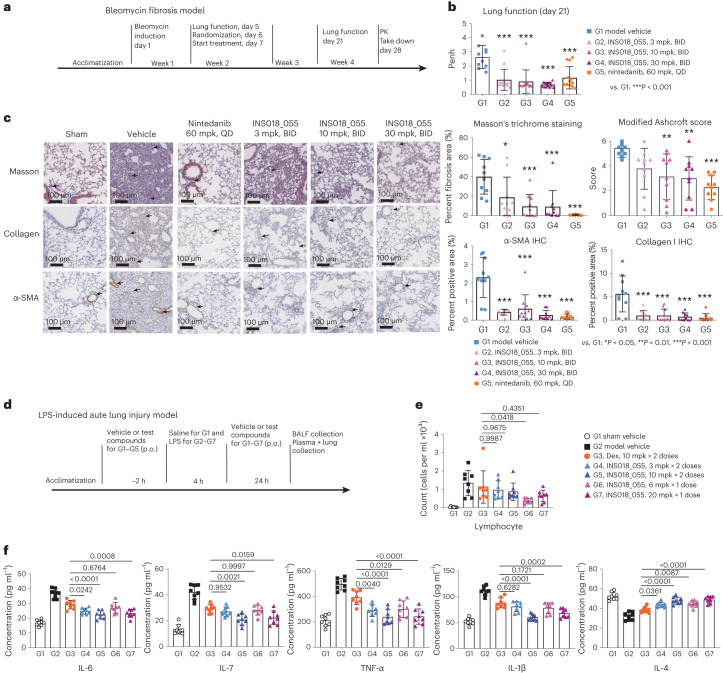
In vivo effects of INS018_055 treatment in mouse models of lung diseases. **a**, Study design of the bleomycin-induced lung fibrosis model in C57BL/6 male mice (*n* = 10 per group). **b**, Lung function on day 21 measured by Penh (mean ± s.d.; group (G)1, *n* = 10; groups 2–5, *n* = 13) (group 1 (vehicle) compared to groups 2–5, *P* < 0.0001; group 5 (nintedanib) compared to groups 2, 3 and 4, with *P* = 0.9986, 0.9426 and 0.464, respectively). **c**, Representative measurements of mice treated with INS018_055 (3, 10 or 30 mg per kg, BID) and nintedanib (60 mg per kg, QD) from **a** showing Masson’s trichrome staining, modified Ashcroft scores and immunohistochemistry (IHC) of collagen 1 and ɑ-SMA. *n* = 10 per group (mean ± s.d.) (Masson’s trichrome staining: group 1 (vehicle) compared to groups 2, 3, 4 and 5, with *P* = 0.0233, 0.0004, 0.0004 and <0.0001, respectively; group 5 compared to groups 2, 3 and 4, with *P* = 0.0799, 0.8126 and 0.8289, respectively. Modified Ashcroft: group 1 compared to groups 2, 3, 4 and 5 with *P* = 0.1021, 0.0071, 0.004 and 0.0001, respectively; group 5 compared to groups 2, 3 and 4 with *P* = 0.1718, 0.791 and 0.8933, respectively. ɑ-SMA: group 1 compared to groups 2–5 with *P* ≤ 0.0001; group 5 compared to groups 2, 3 and 4 with *P* = 0.9689, 0.6125 and >0.9999, respectively. Collagen I: group 1 compared to groups 2–5 with *P* ≤ 0.0001; group 5 compared to groups 2, 3 and 4, with *P* = 0.9985, 0.9986 and >0.9999, respectively). Ordinary one-way ANOVA and post hoc Šídák’s multiple-comparison test were used to assess statistical significance. **d**, Study of INS018_055 in the LPS-induced acute lung injury model in C57BL/6 male mice. *n* = 8 per group. **e**, Lymphocyte cell counts from **d**. *n* = 8 per group (mean ± s.d.) (group 2 (vehicle) compared to groups 3, 4, 5, 6 and 7, *P* = 0.9898, 0.7465, 0.4720, 0.0035 and 0.0698, respectively). Dex, dexamethasone. **f**, Measurements of IL-6, IL-7, TNF-α, IL-1β and IL-4 in BALF by enzyme-linked immunosorbent assay (ELISA). *n* = 8 per group (mean ± s.d.) (group 2 (vehicle) compared for IL-6, IL-7 and IL-1β to groups 3–7 with *P* < 0.0001; for TNF-α, group 3, *P* = 0.0023 and groups 4–7, *P* < 0.0001; for IL-4, group 3, *P* = 0.004 and groups 4–7, *P* < 0.0001). Ordinary one-way ANOVA and post hoc Šídák’s multiple-comparison test was used to assess statistical significance (exact *P* values are provided except for ***P < 0.001). Source data

**Extended Data Fig. 7:**
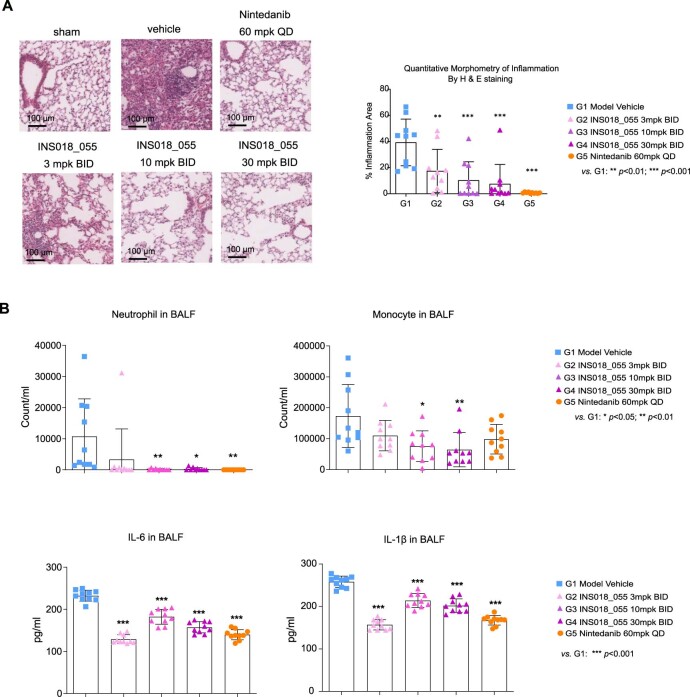
INS018_055 inhibits inflammation in a bleomycin-induced lung (BLM) fibrosis mouse model. **(a)** Images and quantitation of inflammatory area under the treatment of INS018_055 (3, 10, or 30 mg/kg, BID) or nintedanib (60 mg/kg, BID) in bleomycin-induced lung fibrosis model by H&E staining. n = 10 per group. (mean ± SD) Individual values are shown in dots. Comparing G1 (model vehicle group) to G2, 3, 4, 5, yielded p = 0.0092, 0.0003, <0.0001, <0.0001, respectively; Comparing G5 (nintendanib) to G2, 3, 4: p = 0.0789, 0.6504, 0.9063, respectively) **(b)** cell counts of neutrophils, monocytes, and levels of IL-6, IL-1β in BALF of the animals in different treatment groups. n = 10 per group. (mean ± SD) Neutrophils: Comparing G1 (model vehicle) with G2, 3, 4, 5, yielded p = 0.1391, 0.0094, 0.0105, 0.0082, respectively; Comparing G5 (nintedanib) with G2, 3, 4 yielded p = 0.9073, >0.999, >0.999, respectively. Monocytes: Comparing G1 (model vehicle) with G2, 3, 4, 5, resulted in p = 0.2069, 0.01, 0.0031, and 0.0825, respectively; Comparing G5 (nintedanib) with G2, 3, 4, resulted in p = 0.9997, 0.9819, 0.8605, respectively; IL-6: Comparing G1 (model vehicle) with G2, 3, 4, 5, yielded p = <0.0001, <0.0001, <0.0001, <0.0001, respectively; Comparing G5 (nintedanib) with G2, 3, 4 resulted in p = 0.4435, <0.0001, 0.0403, respectively; IL-1β: Comparing G1 (model vehicle) with G2, 3, 4, 5, resulted in p = <0.0001, <0.0001, <0.0001, <0.0001, respectively; compared with G5: G2, 3, 4: p = 0.5115, <0.0001, <0.0001, respectively. All statistical analyses reported here are Ordinary One-way-anova with post-hoc Šídák’s multiple comparisons testing. (asterisks were used instead of an exact value only for *** when p < 0.0001). Source data

To evaluate the anti-fibrotic activity of INS018_055 in vivo, we used the murine bleomycin-induced lung fibrosis model^67,68^. Intratracheal bleomycin administration impaired pulmonary function, as measured by a significant increase in the respiratory parameter ‘enhanced pause‘ (Penh) on day 21 (Fig. 3a). Two weeks of treatment with INS018_055 led to a significant, dose-dependent reduction in Penh, in line with the level achieved by nintedanib treatment (Fig. 3b). INS018_055-treated mice exhibited over 50% reduction in fibrotic area at 3 mg per kg twice daily (BID) and more than 75% reduction at 10 and 30 mg per kg BID dosages relative to vehicle-treated animals. Treatment with INS018_055 and nintedanib significantly decreased modified Ashcroft scores (a semi-quantitative grading for the severity of lung fibrosis) and lung fibrotic area relative to vehicle-treated mice. Additionally, INS018_055- and nintedanib-treated animals showed a marked reduction in α-SMA- and collagen I-positive regions (Fig. 3c). Furthermore, bleomycin administration increased lung inflammation as measured by perivascular and parabronchial infiltration of inflammatory cells, which was attenuated in a dose-dependent manner following INS018_055 or nintedanib administration (Extended Data Fig. 7a). Bronchoalveolar lavage fluid (BALF) showed reduced myeloid cell counts and pro-inflammatory cytokines in INS018_055-treated animals (Extended Data Fig. 7b).

**Extended Data Fig. 8:**
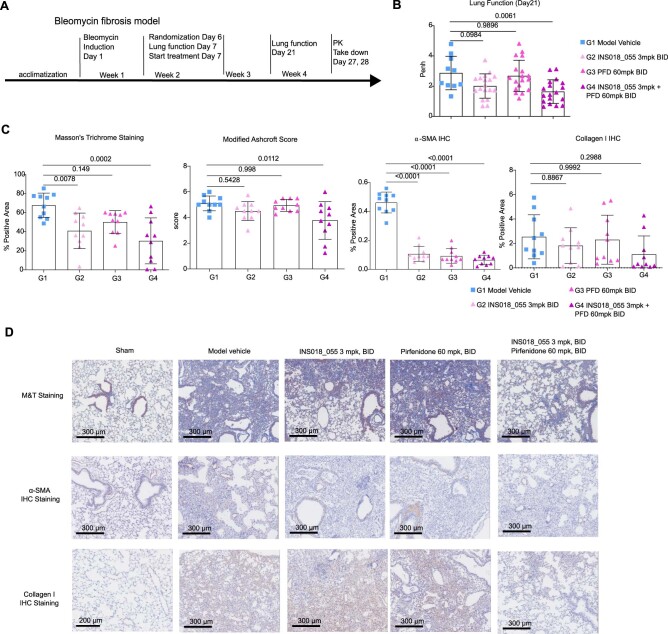
*In vivo* study on the effect of combination therapy of INS018_055 and sub-optimal dose Pirfenidone on mouse model of bleomycin induced lung fibrosis. **(a)** Study design of bleomycin-induced lung fibrosis model in C57BL/6 male mice (n = 10 per group). **(b)** lung function on day 21 measured by Penh (mean ± SD) (n = 10 for G1, and n = 18 for G2-4, with data of animals for PK sampling included). Comparing G1 (model vehicle) with G2, G3, G4, yielded p value = 0.0984, 0.9896, and 0.0061, respectively; Comparing G4 (combo group) with G2, G3 yielded p values = 0.7383 and 0.0062, respectively. **(c)** Quantification of INS018_055 in bleomycin-induced lung fibrosis model Masson’s trichrome staining for fibrosis area, Modified Ashcroft Score, and IHC staining of Collagen 1 and ɑ-SMA (mean ± SD, n = 10). Individual values are shown in dots. MT staining: Comparing G1 (model vehicle) with G2, G3, G4, resulted in p value = 0.0078, 0.149, and 0.0002, respectively; Comparing G4 (combo group) with G2, G3 yielded p values = 0.6478 and 0.0774, respectively. Modified Ashcroft Score: Comparing G1 (model vehicle) with G2, G3, G yielded p value = 0.5428, 0.998, and 0.0112, respectively; Comparing G4 (combo group) with G2, G3 resulted in p values = 0.3414 and 0.0298, respectively. α-SMA: Comparing G1 (model vehicle) with G2, G3, G4 yielded p value = <0.0001, <0.0001, and <0.0001, respectively; Comparing G4 (combo group) with G2, G3 resulted in p values = 0.3935 and 0.7877, respectively. Collagen I: Comparing G1 (model vehicle) with G2, G3, G4 yielded p value = 0.8867, 0.9992, and 0.2988, respectively; Comparing G4 (combo group) with G2, G3 resulted in p values = 0.8884 and 0.4865, respectively. (**d**) Representative histopathology images including M&T Staining, α-SMA IHC Staining and Collagen I IHC Staining. All statistical analyses reported here are Ordinary One-way-anova with post-hoc Šídák’s multiple comparisons testing. (asterisks were used instead of an exact value only for *** when p < 0.0001). Source data

**Extended Data Fig. 9:**
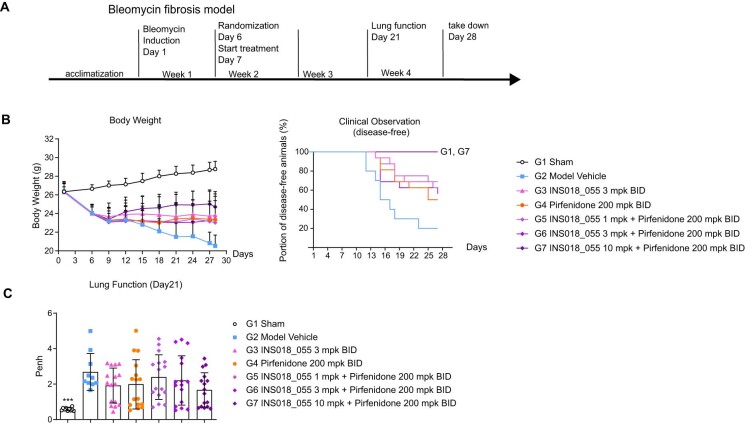
*In vivo* effects of INS018_055 treatment in combination with maximum therapeutic dose of Pirfenidone in mouse models of lung fibrosis. **(a)** study design of bleomycin-induced lung fibrosis model in C57BL/6 male mice. **(b)** Left, body weight change during study process; right: Disease-free curve based on clinical observations (Group,1,2, n = 10, Group 3-7, n = 16) (mean ± SD). **(c)** Lung function measured by Penh (Group,1,2, n = 10, Group 3-7, n = 16) (mean ± SD). Comparing G2 (model vehicle) with G1, p = 0.0005; G3-G7, p = 0.4629, 0.5745, 0.9888, 0.8805 and 0.1635, respectively. All statistical analyses reported here are Ordinary One-way-anova with post-hoc Šídák’s multiple comparisons testing. Source data

To test whether INS018_055 synergizes with the therapeutic benefit of pirfenidone treatment in mice, we measured the in vivo effect of INS018_055 and pirfenidone combination therapy, both at suboptimal doses, in our bleomycin-induced lung fibrosis model (Extended Data Fig. 8a). Two weeks of combined INS018_055–pirfenidone treatment led to a significant improvement in lung function, as measured by a reduction in Penh (Extended Data Fig. 8b). A small but statistically insignificant improvement (*P* = 0.0984) was also observed for the subtherapeutic dose of INS018_055 alone. Treatment with the INS018_055–pirfenidone combination (3 mg per kilogram body weight (mpk) by mouth (p.o.), BID + 60 mpk, p.o., BID) significantly decreased the modified Ashcroft score, fibrotic lung area (Masson’s trichrome staining) and α-SMA abundance as compared with vehicle treatment of mice, while a decreasing trend of collagen I was also observed (Extended Data Fig. 8c,d). Importantly, mice treated with the combined therapies exhibited the strongest anti-fibrotic phenotype across all parameters described. To test the effect of the INS018_055 combination with a therapeutic-level dose of pirfenidone, mice were treated with INS018_055 (3 mpk, p.o., BID), pirfenidone at the maximum therapeutic dose (200 mpk, p.o., BID) or a combination treatment of INS018_055 and pirfenidone (1 mpk + 200 mpk, 3 mpk + 200 mpk, 10 mpk + 200 mpk; p.o., BID) (Extended Data Fig. 9). A complete prevention of clinical symptoms was observed in mice treated with INS018_055 and pirfenidone (10 mpk + 200 mpk, BID).

### INS018_055 reduces murine lipopolysaccharide-induced lung inflammation

As we observed that INS018_055 reduced inflammation in the bleomycin model (Extended Data Fig. 7), we hypothesized that it might elicit a broad anti-inflammatory response. To test this hypothesis, we measured the anti-inflammatory activity of INS018_055 in a lipopolysaccharide (LPS)-induced acute lung injury model (Fig. 3d). We tested two administration schemes: (1) two INS018_055 groups were dosed twice, where one dose was given before and the other after LPS challenge, (2) mice in the other two INS018_055 groups were treated with a single dose after LPS challenge. Intratracheal LPS increased BALF lymphocyte numbers (Fig. 3e), which were reduced by a single dose of INS018_055 administered 4 h after LPS challenge (Fig. 3d). Dexamethasone, a glucocorticoid used to treat acute respiratory distress syndrome^69^, was used as a positive control and was administered once before and once after LPS challenge. INS018_055 at both dosing levels markedly reduced LPS-induced release of interleukin (IL)-1β, IL-6, IL-7 and TNF-α and almost completely restored IL-4 concentrations in BALF (Fig. 3f). Compared to the dexamethasone group, INS018_055-treated groups, particularly groups with 10 mg per kg (two doses) or a single dose of 20 mg per kg (after LPS stimulation), were considerably better at attenuating lung inflammation as measured by IL-1β, IL-6, IL-7 and TNF-α levels. Finally, INS018_055-treated animals had higher levels of the anti-inflammatory cytokine IL-4 than dexamethasone-treated animals. In total, these data argue in favor of INS018_055’s more potent anti-inflammatory effect in this setting.

### INS018_055 inhalation exclusively targets lung fibrosis

**Fig. 4:**
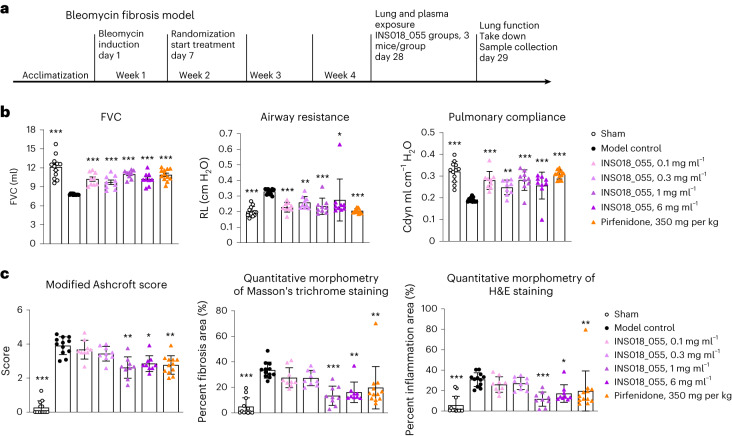
In vivo effects of INS018_055 treatment by inhalation in rat models of lung fibrosis. **a**, Study design of the bleomycin-induced lung fibrosis model in male Sprague Dawley rats (*n* = 12 for INS018_055 groups with three animals killed on day 28 for exposure analysis for plasma and lung, *n* = 12 for other groups). **b**, Lung function on day 29 measured by FVC, airway resistance (RL) and pulmonary compliance (Cdyn) (mean ± s.d.). Statistical analysis was performed using uncorrected two-sided Fisher’s least-significant difference test as post hoc analysis after ANOVA analysis. Compared with the model control group, *P* values of the sham group, INS018_055 groups (0.1 mg ml^−1^, 0.3 mg ml^−1^, 1 mg ml^−1^, 6 mg ml^−1^) and the pirfenidone group (350 mg per kg) are all <0.001 for FVC; <0.001, <0.001, 0.0062, <0.001, 0.0336 and <0.001, respectively, for airway resistance; <0.001, <0.001, 0.002, <0.001, <0.001 and <0.001, respectively, for pulmonary compliance; *n* = 9 for INS018_055 groups, *n* = 12 for other groups (exact *P* values are provided except for ***P < 0.001). **c**, Quantitation for INS018_055 (0.1, 0.3, 1 or 6 mg ml^−1^, QD, inhalation) and pirfenidone (350 mg per kg, QD, oral) groups in the bleomycin-induced lung fibrosis model shown as modified Ashcroft score, Masson’s trichrome staining and hematoxylin and eosin (H&E) staining (mean ± s.d.). Statistical analysis was performed using Dunn’s multiple-comparison tests after the Kruskal–Wallis test (compared with the model control group, *P* values of the sham group, the INS018_055 (1 mg ml^−1^) group, the INS018_055 (6 mg ml^−1^) group and the pirfenidone (350 mg per kg) group are <0.0001, 0.0050, 0.0223 and 0.0066 for modified Ashcroft score, respectively; <0.0001, 0.0007, 0.0040 and 0.0028 for Masson’s trichrome staining, respectively; and <0.0001, 0.0008, 0.0250 and 0.0065 for H&E staining, respectively; *n* = 9 for INS018_055 groups, *n* = 12 for other groups (exact *P* values are provided except for ***P < 0.001). Source data

To achieve a therapeutic concentration in the lung while minimizing systemic exposure, we formulated an aerosolized INS018_055 intervention that was tested in a rat bleomycin-induced lung fibrosis model (Fig. 4a). One week after bleomycin induction, rats were exposed to INS018_055 aerosols generated by nebulization solution (0.1, 0.3, 1 or 6 mg ml^−1^) for 30 min per day for 21 d. These values are equivalent to 0.04, 0.136, 0.485 and 2.575 mg per kg of actually delivered doses, respectively. Control animals were given NaCl solution by inhalation, while rats orally treated with pirfenidone (350 mg per kg) served as a positive control.

Plasma and lung concentrations of INS018_055 were measured in three animals immediately following administration, demonstrating approximately 50-fold higher levels in the lung than in the blood (Supplementary Information 7). Bleomycin-induced loss of lung function was significantly improved in rats treated with aerosolized INS018_055 solution across all doses and groups. Lung function was assessed by restoration of forced vital capacity (FVC, ml), pulmonary compliance (ml cm^−1^ H_2_O) and recovery from increased pulmonary resistance (cm H_2_O) (Fig. 4b). INS018_055 treatment (1 and 6 mg ml^−1^) substantially inhibited lung fibrosis and inflammation (Fig. 4c).

### TNIK inhibition mitigates kidney fibrosis

**Fig. 5:**
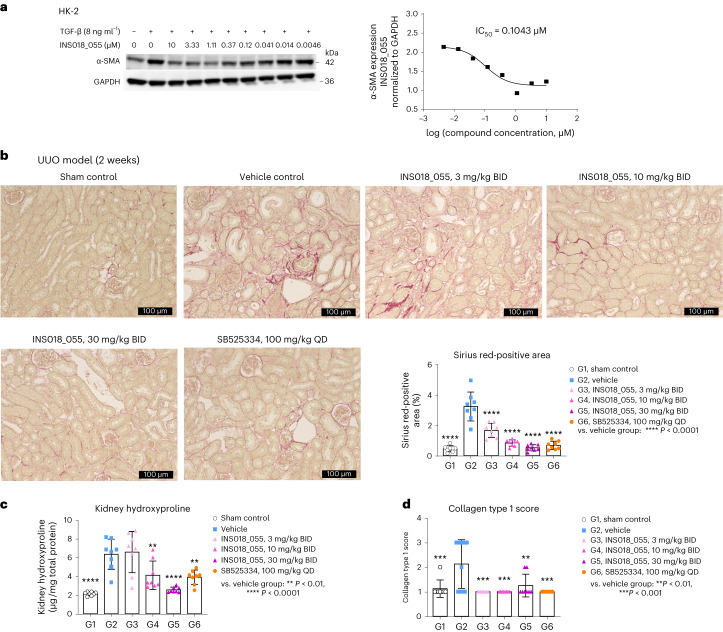
In vitro and in vivo studies on the effect of INS018_055 on kidney cells and the mouse model of kidney fibrosis. **a**, Left, representative western blot showing protein level changes of α-SMA with TGF-β and INS018_055 treatment. Right, graphical representation and IC_50_ determination of the effect of α-SMA expression in HK-2 cells. This represents a single independent experiment: no statistical conclusions were made. **b**, Representative images of Sirius red staining and quantitation of positive area (bottom right). **c**, Quantification of hydroxyproline content in the kidney. **d**, Scoring of IHC staining of collagen type 1 (for **b**–**d**, *n* = 8, mean ± s.d.; statistical analysis was performed using two-sided Bonferroni multiple-comparison test (using Prism 6 software) to compare between the vehicle group and other treatment groups for **b**–**d**). *P* values < 0.05 were considered statistically significant. Compared with the vehicle group, *P* values of the sham control group, INS018_055 groups (3, 10 and 30 mg per kg, BID) and the SB525334 group (100 mg per kg, QD) are all <0.0001 for Sirius red-positive area; <0.0001, >0.9999, 0.0074, <0.0001 and 0.003, respectively, for kidney hydroxyproline content; and <0.0006, 0.0001, 0.0001, 0.0028 and 0.0001, respectively, for collagen type 1 score (exact *P* values are provided except for ***P < 0.001). Source data

Given the lack of anti-fibrotic drugs in other diseases in which fibrosis drives organ pathology, we tested whether INS018_055 could provide therapeutic benefit in a murine kidney fibrosis model. The CC_50_ of INS018_055 in a proximal tubular kidney cell line, HK-2, was 37.37 μM (Extended Data Fig. 3d), which is 300-fold higher than its IC_50_ for α-SMA inhibition (0.104 µM), indicative of anti-fibrotic function with limited cytotoxicity (Fig. 5a). To further evaluate the effect of INS018_055 in vivo, we employed the unilateral ureteral obstruction (UUO) model^70^ which mimics renal interstitial fibrosis found in humans. The vehicle-treated group showed a significant increase in kidney hydroxyproline content and Sirius red staining, indicating higher tissue fibrosis than in naive mice (Fig. 5b,c). By contrast, animals treated with INS018_055 (3, 10 and 30 mg per kg, BID) and the activin receptor-like kinase 5 (ALK5) inhibitor SB525334 (ref. ^71^) exhibited reduced fibrosis compared to vehicle-treated mice. Furthermore, both compounds significantly decreased hydroxyproline content (Fig. 5c) and suppressed collagen 1 staining (Fig. 5d).

### Effects of INS018_055 treatment on dermal fibroblasts

**Extended Data Fig. 10:**
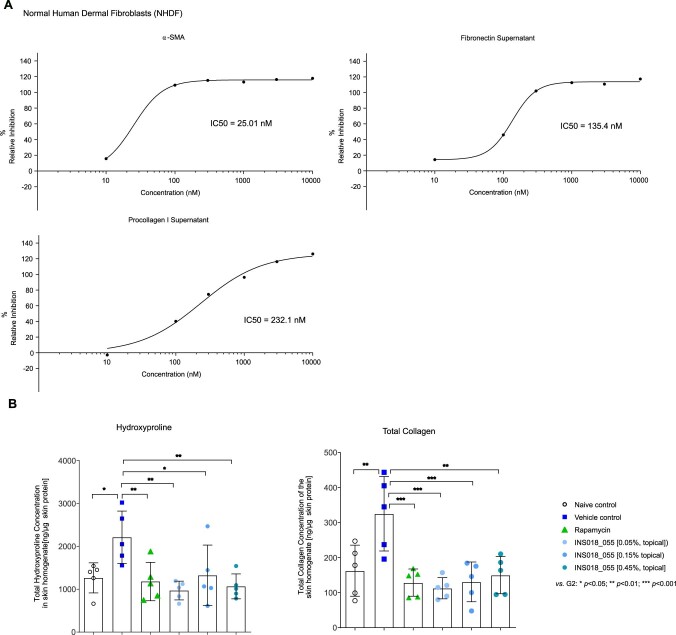
*In vitro* and *in vivo* study of INS018_055 on skin fibrosis. **(a)** IC_50_ values of inhibition effect of INS018_055 on levels of α-SMA expression, and levels of fibronectin and procollagen I in supernatant in cultured NHDF cells. Left: Cells were treated with INS018_055 and TGF-β (0.1 ng/ml) for 72 hours. The fluorescence intensity of α-SMA normalized to the total number of nuclei identified by staining with Hoechst 33258. Right and below: Cells were treated with INS018_055 and TGF-β (10 ng/ml) for 72 hours. Fibronectin and procollagen I contents were measured in the culture supernatants. Relative inhibition (%) = (Mean Stimulated control - Value)/ (Mean Stimulated control –Mean Non-stimulated control) X 100. All experimental conditions were performed in n = 5. (mean ± SEM). **(b)** INS018_055 inhibited collagen level in bleomycin induced skin-thickening rat model. Sprague Dawley rats were injected with 0.1 mL 1 mg/mL bleomycin subcutaneously into two sites on the shaved regions, once daily for 4 weeks. Treatments were administered with vehicle, Rapamycin (1.5 mg/kg, intraperitoneal) or INS018_055 (0.05%, 0.15% or 0.45%, topical), 30 minutes before administration of bleomycin, everyday for four weeks. Hydroxyproline and collagen content was measured (mean ± SD), (n = 5). The concentrations of hydroxyproline and collagen were normalized to total protein content using the Bradford method. Statistical analysis was performed using one-way ANOVA, then Dunnett’s multiple comparison test was performed. For hydroxyproline, compared with vehicle control group (Group 2), p values of Group naive control, Group of rapamycin, Groups of INS018_055 [0.05%, 0.15% and 0.45%], are 0.0165, 0.0085, 0.0015, 0.0261, and 0.0033, respectively. For total collagen, p values of Group naive control, Group of rapamycin, Groups of INS018_055 [0.05%, 0.15% and 0.45%], are 0.0025, 0.0003, 0.0001, 0.0004, and 0.0012, respectively. p values < 0.05 were considered statistically significant. Source data

To assess the anti-fibrotic effect of INS018_055 in other tissues, we used a normal human dermal fibroblast (NHDF) cell line that was previously used to measure the production of extracellular matrix proteins such as fibronectin and collagen in the context of skin fibrosis^72^. INS018_055 treatment of TGF-β-stimulated NHDF cells resulted in a concentration-dependent inhibitory effect on procollagen I, α-SMA and fibronectin expression and release with IC_50_ values of ~232 nM, 25 nM and ~135 nM, respectively (Extended Data Fig. 10a). We next sought to test the effect of topical administration of INS018_055 in a model of bleomycin-induced skin fibrosis. While bleomycin administration significantly increased collagen and hydroxyproline levels, animals treated topically with INS018_055 at doses of 0.05%, 0.15% and 0.45% showed a significant reduction in collagen and hydroxyproline levels compared to control rats (Extended Data Fig. 10b).

### Phase 0 and I clinical trial testing of INS018_055

We carried out a phase 0 single-micro-dose study in healthy participants (ACTRN12621001541897) to evaluate INS018_055 pharmacokinetics (PK), in which we observed that an intravenous administration of 100 μg was well tolerated (Supplementary Information 8). A randomized, double-blind, placebo-controlled phase I clinical trial (NCT05154240) was then initiated to evaluate safety and tolerability (primary objective) as well as PK (secondary objective) of INS018_055 in 78 healthy volunteers. Part A of the trial was a single-ascending-dose-administration (SAD) study, with 40 participants assigned randomly at a ratio of 3:1 to receive INS018_055 or placebo at sequential doses on day 1 (10, 30, 60, 90, 120 mg). To evaluate safety and tolerability in the context of dietary effects, safety and PK evaluations were repeated with a standard high-fat meal in the cohort that received 90 mg. Part B was a multiple-ascending-dose-administration (MAD) study, with 24 healthy participants assigned randomly at a ratio of 3:1 to receive INS015_055 at sequential doses (30, 60, 120 mg) or placebo daily for 7 d.

### PK profile for SAD and MAD trials

**Fig. 6:**
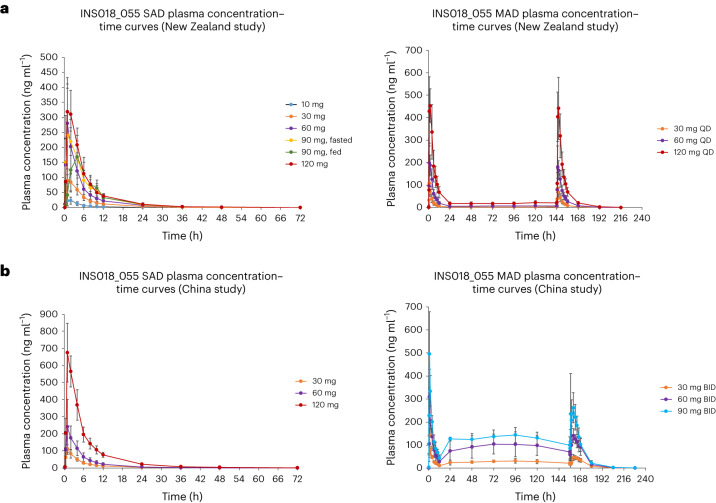
Pharmacokinetic analysis in the clinical phase I trial. **a**,**b**, Plasma concentrations of INS018_055 versus time in SAD and MAD of the phase I study performed in New Zealand (**a**) (mean ± s.d., *n* = 6 per group) and in SAD and MAD of the phase I study performed in China (**b**) (mean ± s.d., *n* = 6 per group). Source data

For SAD, in fasted individuals, profiles of plasma concentration versus time for INS018_055 showed a rapid absorption phase, reaching peak concentrations with median time taken to reach the maximum concentration (*T*_max_) values ranging from 1.00 to 1.53 h after the dose, followed by declines in a multiphasic manner with geometric mean elimination half-life (*t*_1/2_) values ranging from 7.42 to 9.74 h (Fig. 6a and Supplementary Information 9). INS018_055 levels were still detectable up to the last sampling point at 72 h across all doses except the cohort receiving 30 mg, whereas INS018_055 was detectable up to 48 h after the dose. Overall, the INS018_055 geometric mean peak (*C*_max_) and total exposures (area under the curve (AUC)_0−*t*_) and total exposure across time (AUC from zero to infinity (AUC_0−inf_)) increased proportionally (Supplementary Information 9) with dose except for *C*_max_ at the 90-mg level, which was similar to that at the 60-mg dose. The fed scenario in the cohort receiving 90 mg showed lower INS018_055 plasma absorption rates (median *T*_max_ of 3.00 h) than the fasted scenario (median *T*_max_ of 1.01 h). Total geometric *C*_max_ and mean AUC values were lower under fed conditions (169 ng ml^−1^ for *C*_max_, 1,390 and 1,400 h × ng ml^−1^ for AUC_0−*t*_ and AUC_0−inf_) than under fasted conditions (270 ng ml^−1^ for *C*_max_, 1,620 and 1,630 h × ng ml^−1^ for AUC_0−*t*_ and AUC_0−inf_).

For MAD, plasma exposure was measured in intensive time points after the first dose and the final dose as well as once (before the dose) in days between (Fig. 6a and Supplementary Information 9). INS018_055 *C*_max_, exposure (AUC_0−tau_) and average concentration (*C*_av_) values increased with the escalation in dose level. Profiles of plasma concentration versus time for INS018_055 after multiple oral administration once a day (QD) for 7 d at dose levels of 30, 60 and 120 mg under fasted conditions were characterized by a rapid absorption phase, with peak concentrations reached at a median *T*_max_ ranging from 1.00 to 2.00 h after the dose, followed by overall decline in a multiphasic manner, with *t*_1/2_ values consistent across dose levels with values ranging from 9.36 to 11.9 h after the last dose. Mean INS018_055 plasma concentrations increased with escalating doses of INS018_055. Plasma INS018_055 was detectable up to the last sampling time point at 72 h after administration at all dose levels except for the cohort receiving 30 mg, in which plasma concentrations were detectable up to 48 h. Accumulation of INS018_055 was not observed. The mean ratio of *C*_max_ and AUC_0−tau_ on day 7 to day 1 ranged from 0.739 to 0.905 and 0.996 to 1.05, respectively, showing a stable exposure throughout the study.

For the phase I trial conducted in China (CTR20221542), profiles of plasma concentration versus time for INS018_055 in the SAD dose-escalation phase (with dose levels of 30, 60 and 120 mg) and in MAD cohorts administered for 7 d with 30, 60 and 90 mg BID are presented in Fig. 6b. INS018_055 showed overall comparable PK profiles in the Chinese study as compared to the New Zealand study without noteworthy differences. The only notable difference was that the plasma exposure increase from 60 to 120 mg for the single dose was slightly greater than the expected dose proportionality in the Chinese trial. After administering 30-, 60- and 90-mg doses BID for 7 d, no significant plasma accumulation of INS018_055 was observed.

Across both SAD and MAD components of phase I trials of INS018_055, the severity of all treatment-emergent adverse events including fecal occult blood positivity, headache and phlebitis (Supplementary Information 9 and 10) was mild and resolved at the end of the studies. The following exceptions were reported: one individual from the active drug-treated group in the New Zealand MAD trial exhibited influenza-like illness and seven (five from the active drug-treated group and two from the placebo group) individuals in the MAD trial in China also experienced influenza-like illnesses that were not related to the study and coincided with high prevalence of coronavirus disease 2019 (COVID-19) in China during the time of the study. The results of the two phase I studies indicate that INS018_055 is safe and well tolerated in healthy volunteers with good oral bioavailability and dose-proportional PK.

## Discussion

The discovery of more effective anti-fibrotic therapies represents a major clinical challenge due to an incomplete understanding of fibrotic reprogramming in diseased tissue. Here, we navigate this limitation by harnessing the power of a generative AI platform to unbiasedly identify TNIK as an anti-fibrotic target. TNIK was also implicated in multiple hallmarks of aging, suggesting broader therapeutic potential. Using a comprehensive generative AI-driven drug-design technique, we designed a small-molecule inhibitor of TNIK, INS018_055. We demonstrate that TNIK inhibition effectively ameliorates fibrotic processes in vitro and in vivo in lung, kidney and skin fibrosis disease models. Furthermore, INS018_055 improved overall lung function in a murine model of lung fibrosis as compared to that of vehicle-treated mice. Target discovery to preclinical candidate nomination took only 18 months to accomplish, with INS018_055 evaluated in two clinical phase I trials (NCT05154240 and CTR20221542). To date, we have found that INS018_055 demonstrated safety and tolerability in healthy volunteers, providing a strong basis for further clinical studies in patients with IPF. Collectively, our preclinical and phase I clinical trial findings speak to the potential of AI-driven drug discovery in streamlining drug design in the setting of fibrosis and other contexts. Nevertheless, it is important to note that these results require further assessment in phase II and III clinical trials to validate these promising findings. At the time of this publication, two phase II trials using INS018_055 (NCT05975983 and NCT05938920) for the treatment of IPF are underway.

TNIK was identified by tasking the PandaOmics target-discovery platform^14^ to search for new anti-fibrotic targets. PandaOmics was used to prioritize potential candidate targets based on the combination of omic- and text-based models (scores), which were specifically validated to identify targets using the time machine approach (as described in the Methods and Supplementary Information). TNIK scored as number 1 using the ‘protein and receptor kinase’ PandaOmics setting, which was based on the combination of bioinformatic and AI-driven philosophies applied to multi-model omics datasets followed by application of novelty, small-molecule druggability and protein class filters. This general methodology and pipeline can be used for drug discovery in the context of other diseases outside of fibrosis.

Our approach has provided mechanistic insight into the role of TNIK signaling during fibrosis. TNIK belongs to the germinal center kinases, which activate the profibrotic c-JUN pathway^73^. TNIK is also a RAP2 effector, which regulates cell spreading^74^. Furthermore, TNIK has been described as an essential mediator of the WNT signaling axis by direct interaction with the T-cell factor and lymphoid enhancer factor (TCF-LEF) LCF–L complex and TCF in gut epithelial cells^45^. After identifying TNIK as a target in IPF, we used Chemistry42 (refs. ^40,41,75–77^) to discover the lead inhibitory compounds targeting this kinase. Chemistry42 first applied 30 generative models in parallel to generate compound structures. Next, the model included feedback in the virtual screening approach to optimize the compounds further. During the generation process, the structures and associated scores produced by each generative model were dynamically shared with the other models to jointly explore the chemical space, optimize and finally generate favorable leads. INS018_055 showed the most desirable drug properties with strong selectivity for inhibiting TNIK.

While TNIK inhibition has been previously described in the context of cancer progression^46^, our drug-discovery pipeline has circumvented many of the known limitations of this conventional approach. NCB-0846, an inhibitor with high potency^56^, was developed and found to exhibit anti-cancer activity in multiple preclinical cancer models^56,78,79^. More recently, NCB-0846 was also reported to ameliorate liver fibrosis^80^, which is in line with orthogonal findings in our study. However, selectivity profiling has demonstrated that, at a concentration of 0.1 μM, NCB-0846 displayed inhibitory effects on additional kinase targets, including cyclin-dependent kinase (CDK)2 (>80% inhibition)^56^. One study found that NCB-0846 may impair cell viability at therapeutic doses despite its strong inhibitory effect on collagen trafficking and deposition in a murine model of liver fibrosis^80^. By contrast, INS018_055 inhibited cell viability at much higher concentrations, eliciting clear anti-fibrotic effects and ruling out non-specific cytotoxicity (Extended Data Fig. 3). Moreover, INS018_055 exhibits a favorable safety profile in nonclinical toxicology, with results from two phase I clinical trials further confirming that INS018_055 is generally well tolerated and has desirable PK profiles.

As IPF progression is driven by both fibrosis and inflammation^81,82^, INS018_055 targets both main disease-driving processes in IPF evolution. The processes that initiate fibrosis are not yet completely understood, and, as a result, there are no established approaches to prevent its induction. Nevertheless, the prevalence of pulmonary fibrosis has dramatically increased due to the COVID-19 pandemic, reiterating the need for effective anti-fibrotic therapeutic interventions^83^. Given its potent anti-inflammatory and anti-fibrotic activity, INS018_055 may be used as a preventive agent for patients with a high risk of developing post-COVID-19 acute respiratory distress syndrome, including but not limited to older and immunocompromised individuals^84,85^. Moreover, those at high risk of developing organ failure due to progressive CKD may^86^ also benefit from preventative therapy with INS018_055.

In summary, this generative AI-enabled study aimed at developing better compounds for fibrosis-related diseases provides evidence that generative AI platforms offer time-efficient solutions for generating target-specific drugs with potent anti-fibrotic activity. INS018_055 demonstrated good tolerability and favorable PK profiles in both phase I trials conducted in New Zealand and China, with a phase IIa trial in patients with IPF currently underway. We believe that this study underscores the strength of AI-enabled drug-discovery approaches, which will likely revolutionize drug discovery.

## Methods

### Ethics

#### Human tissue and clinical trials

All tissues used for isolation were obtained with informed consent and conformance to HIPAA regulations to protect the privacy of the donor’s personally identifiable information. Humans who participated in any clinical trial in this study provided written consent for study participation. The entirety of this study adheres to the Declaration of Helsinki.

#### Phase 0 clinical trial in Australia

This trial was conducted at the CMAX Clinical Research Center, 18A North Terrace, Adelaide, SA 5000, Australia. This phase 0 clinical trial, also the first-in-human clinical trial of INS018_055, was conducted in Australia (ACTRN12621001541897). This trial was conducted in accordance with the ethical principles of good clinical practice, according to the International Council for Harmonisation (ICH) Harmonised Guideline E6(R2) Integrated Addendum to ICH E6(R1): Guideline for Good Clinical Practice ICH E6(R2), annotated with comments by the Australian Therapeutic Goods Administration (2018). This study was approved by the Bellberry Human Research Ethics Committee.

#### Phase I clinical trial in China

This trial was conducted at Zhejiang Xiaoshan Hospital, 728 Yucai North Road, Xiaoshan District, Hangzhou, Zhejiang 311202, China. Detailed information including facilities and inclusion and exclusion criteria can be found at the following link: http://www.chinadrugtrials.org.cn/clinicaltrials.prosearch.dhtml (registration number CTR20221542). This study was approved by the Zhejiang Xiaoshan Hospital clinical trial ethics committee. The number of the ethics committee approval letter is EC-2022102505.

#### Phase I clinical trial in New Zealand

This trial was conducted at the New Zealand Clinical Research Center, level 4, 264 Antigua Street, Central City, Christchurch, 8011, New Zealand (registration number NCT05154240). This study was approved by the Northern B Health and Disability Ethics Committee. The ethics reference number for this protocol is 2021 FULL 11770.

#### Animal studies

All animal studies were ethically and humanely conducted following institutional animal care and use committee (IACUC), IRB or relevant animal-handling ethics organizational guidelines by our partnering CROs. Murine LPS and bleomycin studies were carried out by HD Biosciences, which were approved under the following AUF protocol numbers: AUF 146 and AUF 117. These AUF protocols were approved by the IACUC at HD Biosciences, and the studies were conducted at an AAALAC-accredited facility. The bleomycin fibrosis study in rats that were administered INS018_055 as an inhalable agent was carried out by JOINN Laboratories. This study’s IACUC-approved procedural number is S-ACU22-1144. JOINN Laboratories is fully accredited by the AAALAC, and the study adheres to regulations and rules laid out by the IACUC. The UUO kidney study was performed by SMC Laboratories. The animal care and use committee-approved protocol number is U32. All animals used in this study were housed and cared for in accordance with the Japanese Pharmacological Society Guidelines for Animal Use. The skin fibrosis model was carried out by TheraIndx LifeSciences. This study was performed using protocols approved by the Institutional Animals Ethics Committee of the test facility, which was designed under CPCSEA guidelines for animal care. The registration number for this protocol is 1852/PO/Rc/S/16/CPCSEA. For all aforementioned studies, animals were housed in groups (<5 per cage) in temperature-controlled facilities (20–26 °C) with 12-h light–12-h dark–light cycles. All animals had ad libitum access to drinking food and water.

### ChatPaperGPT tool

To encourage a thorough, unbiased interrogation of our study, we developed an interactive generative AI tool, ChatPaperGPT, to navigate all contents of our study. We encourage all readers to critically examine our study, its accompanying state-of-the-art drug-discovery methodology and all associated data using our ChatPaperGPT tool (https://papers.insilicogpt.com/).

### Antibodies

Antibodies used were anti-human-fibronectin (Invitrogen, MA5-11981), anti-human E-cadherin (BD Biosciences, 610182), anti-human N-cadherin (D4R1H) (Cell Signaling, 13116S), anti-human phospho-SMAD2 (Ser465/467)/SMAD3 (Ser423/425) (D27F4) (Cell Signaling, 8828S), anti-human phospho-SMAD2 (Ser465/467) (Cell Signaling, 3108S), anti-human SMAD2/SMAD3 (D7G7) (Cell Signaling, 12470S), anti-human phospho-FAK (Tyr397) (D20B1) (Cell Signaling, 8556), anti-human FAK (Cell Signaling, 3285), anti-human TNIK (Cell Signaling, 32712), anti-human NF-κB p65 (D14E12) (Cell Signaling, 8242S), anti-human phospho-NF-κB p65 (Ser536) (93H1) (Cell Signaling, 3033S), anti-human β-catenin (Cell Signaling, 9562), anti-human α-tubulin (Abcam, ab18251), anti-human HDAC2 (Abcam, ab12169), anti-human histone 3 (Abcam, ab176842), anti-α-SMA antibodies (Cell Signaling, 19245; Abcam, ab5694; Abcam, ab32575), anti-GAPDH (Absin, 016D), anti-collagen I antibody (Abcam, ab34710; LSL), goat anti-mouse IgG (Abcam, ab205719), goat anti-rabbit IgG (Abcam, ab205718) and goat polyclonal antibodies conjugated to HRP (DAKO, K4003; Vector Lab, PI-1000).

### Enzyme-linked immunosorbent assay kits

ELISA kits used were as follows: IL-1β (Sinobest Bio, YX-E01291M), IL-4 (Sinobest Bio, YX-E00064M), IL-6 (Sinobest Bio, YX-E00066M), TNF-α (Sinobest Bio, YX-E00104M), fibronectin (Takara, MK115), procollagen type I C peptide (Takara, MK101), rat hydroxyproline (KinesisDx, K11-0512), collagen (Abcam, ab222942).

### Other reagents

Other reagents used were as follows: 20× TBS Tween-20 (Thermo, 228360), 4–20% Criterion TGX Precast Gels, 26 well, (Bio-Rad, 5675671095), 4% Paraformaldehyde Fix Solution (Beyotime, P0099), bovine pituitary extract (ScienCell, 0713-1100mg), BSA (Sigma, B2064), bleomycin sulfate (MCE, HY-17565), CellTiter-Glo Buffer (Promega, G756B), CellTiter-Glo Substrate (Promega, G755B), cOmplete, EDTA-free Protease Inhibitor Cocktail (Roche, 4693134693132001), Difco skim milk (BD, 23232100), dimethylsulfoxide (DMSO) (Sigma, D2650-1100mL), DMEM high-glucose culture medium (Gibco, 8128120212), DMEM (Gibco, 119611960051), Dulbecco’s phosphate-buffered saline (DPBS) (Corning, 2103121031-CVC), eosin Y (Baso, BA4022), FBS (ExCell Bio, 12040), hematoxylin solution (Baso, BA4041), human recombinant epidermal growth factor (EGF) (Gibco, PHG0314), human TNF-α (Sigma, T0157), iBlot 2 NC Regular Stacks (Invitrogen, IB223001), keratinocyte serum-free medium (K-SFM) (Gibco, 10724011), Lipofectamine 3000 Transfection Kit (Thermo Fisher, L3000008), LPS from *Escherichia coli* O55:B5 (Sigma, L2880), MEM non-essential amino acids (100×) (Gibco, 11140050), methylcellulose (Sigma, M7140), MOPS SDS Running Buffer (1×) (Invitrogen, NP0001), nintedanib esylate (DC, DC8608), Novex ECL Chemiluminescent Substrate Reagent Kit (Invitrogen, WP220005), NuPAGE 4–12% Bis-Tris Gel, 15 well (Invitrogen, NP0330336BOX), NuPAGE LDS sample buffer (4×) (Invitrogen, NP0007), Opti-MEM (Gibco, 319831985062), PageRuler Plus Prestained Protein Ladder (Thermo Fisher, 226619), penicillin–streptomycin solution (10,000 U ml^−1^ penicillin, 10,000 μg ml^−1^ streptomycin) (HyClone, SV330010), Phosphatase Inhibitor Cocktail 2 (Sigma, P57265ML), Phosphatase Inhibitor Cocktail 3 (Sigma, P00445ML), PhosSTOP phosphatase inhibitor cocktail (Roche, 4906844906845001), Pierce western blot transfer buffer (10×) (Thermo Fisher, 335040), Pierce BCA Protein Assay Kit (Pierce, 223227), PMSF protease inhibitor (Dalian Meilun, MA0001-May06E), PMSF protease inhibitor (Beyotime, 105212110521210126), Recombinant Human TGF-β 1 Protein (MCE, AV6120051, R&D, 240-B-010 and 240-b002), Restore Western Blot Stripping Buffer (Pierce, 221059), radioimmunoprecipitation assay (RIPA) buffer (Sigma, R0278), RIPA lysis buffer (Beyotime, 926192092619200827), RPMI 1640 medium (Gibco, 22400089), saline (Hualu, NMPN 37022750), Subcellular Protein Fractionation Kit for Cultured Cells (Thermo Fisher, 778840), SuperSignal West Femto Maximum Sensitivity Substrate (Thermo Fisher, 334096), Thermo Scientific PageRuler Prestained Protein Ladder (Thermo, 226619), trypsin-EDTA (0.25%), phenol red (Gibco, 25200072).

### Compounds

INS018_055 was provided by WuXi AppTec. SB525334, was purchased from TCI. Nintedanib esylate was purchased from DC (DC8608). Dexamethasone was purchased from Sigma (4902). Pirfenidone was purchased from DC (DC8792).

### Synthesis of INS018_055

Synthesis, stability and characterization of INS018_055 are laid out in detail in Supplementary Information 4.

### In vitro experiments

#### Cell lines

The human fetal lung fibroblast cell line MRC-5 was purchased from ATCC (CCL-171) (the cell viability assay was performed at WuXi AppTec) and Shanghai Cell Bank, Chinese Academy of Sciences (the α-SMA assay was performed at Boji). Human primary bronchial epithelial cells were derived from three donors with IPF (IPF05, IPF06 and IPF08) and three healthy donors (Br285, Br311 and 410955). Human primary lung fibroblasts were derived from three donors with IPF (IPF05, IPF06 and IPF08) and three healthy donors (FB218, 03HF67101 and FB2382). The human lung adenocarcinoma cell line A549 was purchased from ATCC (CCL-185). Human embryonic kidney 293T/17 cells were purchased from ATCC (CRL-11268). The human kidney cell line HK-2 was purchased from ATCC (CRL-2190). NHDFs were purchased from Bioalternatives (PF2).

#### Mycoplasma testing

All cell lines used were checked and cleared for mycoplasma at the following contract research organizations: WuXi AppTec (China), Charles Rivers (Netherlands), Guangzhou Boji Medical Biotechnological (China), Bioalternatives (France).

#### Cell culture conditions

All cell lines were cultured at 37 °C in a humidified atmosphere of 95% air and 5% CO_2_. MRC-5 cells were cultured in MEM medium supplemented with 1% GlutaMAX, 10% FBS, 1% non-essential amino acids and 1% penicillin–streptomycin (100 U ml^−1^). Human primary lung fibroblasts and human primary bronchial epithelial cells were cultured according to Charles River’s internal protocol. A549 cells were cultured in RPMI 1640 medium supplemented with 10% FBS and antibiotics (penicillin and streptomycin, 100 U ml^−1^ each). The 293T/17 cells were cultured in DMEM medium supplemented with 10% FBS and 1% penicillin–streptomycin solution (100 U ml^−1^). HK-2 cells were cultured in K-SFM containing 50% growth factor. Human primary lung fibroblasts, NHDF cells, were cultured in DMEM supplemented with 2 mM glutamine, 50 U ml^−1^ penicillin, 50 μg ml^−1^ streptomycin and 10% fetal calf serum.

#### Surface plasmon resonance assay for binding to His-tagged TNIK (9–315)

Binding kinetics for INS018_055, NCB-0846 and KY-05009 were measured with the Biacore 8K system. His-tagged TNIK (9–315) (WuXi AppTec) was immobilized on an NTA sensor chip for 43 s at 30 µg ml^−1^ and 5 µg ml^−1^ (His capture and amine coupling). The three compounds INS018_055, NCB-0846 and KY-05009 were prepared by twofold dilutions from 1,000 nM to 2 nM. The association time and the dissociation time were 90 s and 180 s, respectively (flow rate, 30 μl per min; sample compartment temperature, 15 °C; analysis temperature, 15 °C). The running buffer contained 20 mM HEPES, pH 7.5, 150 mM NaCl, 1 mM MgCl_2_, 0.05% Tween-20, 2% glycerol, 1 mM TCEP and 2% DMSO.

#### Enzyme selectivity profiling

INS018_055 was tested against selected kinases using Eurofins standard KinaseProfiler assays. A total of 430 kinases were tested. Protein kinases (with the exception of ATM(h) and DNA-PK(h)) were assayed in a radiometric format, whereas lipid kinases, ATM(h), ATR/ATRIP(h) and DNA-PK(h) were assayed using a homogeneous time-resolved fluorescence format. INS018_055 was prepared in a working stock 50× the final assay concentration in 100% DMSO. The required volume of the 50× stock of INS018_055 was added to the assay well, and then a reaction mix containing enzyme and substrate was added to the well. ATP at the selected concentration initiated the reaction. Data were processed by custom-built in-house analysis software at Eurofins. Results are presented as percentages of kinase activities remaining in comparison to the DMSO control. The following formula was used to calculate these values:

(Mean of sample counts − mean of blank counts)/(mean of control counts).

IC_50_ values were calculated using XLfit version 5.3 (ID Business Solutions). Sigmoidal dose–response (variable slope) curves were fit based on the mean result for each test concentration. Non-linear regression analysis was applied. When the top or bottom of the curve falls >10% outside of 100 or 0, either or both of these limits may be constrained at 100 and 0 when the QC criterion on *R*^2^ is met.

#### Measurement of FMT and EMT on human primary cells by high-content analysis

For the measurement of FMT by α-SMA, on the first day, human lung-derived primary lung fibroblast cells were seeded. After 2 d, the medium of the cells was refreshed. On day 5, cells from donors with IPF or healthy donors were prepared in two sets and exposed to INS018_055. After 1 h, all cells were treated with 1.25 ng ml^−1^ TGF-β1. On the eighth day (72 h after triggering), cells were fixed with 4% formaldehyde, stained for α-SMA and with DAPI and then imaged and quantified via high-content analysis (HCA) (IN Cell Analyzer 2200, GE Healthcare).

For the measurement of EMT by FN1, on the first day, human primary bronchial epithelial cells were seeded. After 2 d, the medium of the cells was refreshed. On day 6, cells from donors with IPF or healthy donors were prepared in two sets and exposed to INS018_055. After 1 h, all cells were treated with 5 ng ml^−1^ TGF-β1. On the ninth day (72 h after triggering), cells were fixed with 4% formaldehyde, stained for FN1 and with DAPI and then imaged and quantified via HCA (IN Cell Analyzer 2200, GE Healthcare).

##### Analysis of α-SMA and FN1

Segmentation and quantification of α-SMA and FN1 immunoreactivity was carried out with an HCA algorithm, with density × area (*D* × *A*) output. Data normalization of raw α-SMA or FN1 (*D* × *A*) to percent inhibition values was performed on a plate-to-plate basis.

Percent inhibition = (100 − (*μ*_p_ − *X*_i_)/(*μ*_p_ − *X*_n_)) × 100

*μ*_p_ is the average α-SMA or FN1 value of the positive control (TGF-β1 + 1 μM SB525334). *μ*_n_ is the average α-SMA or FN1 value of the vehicle control (TGF-β1 + 0.1% DMSO). *X*_i_ is the compound α-SMA or FN1 value. IC_50_ values for all compounds (if calculable, based on the point of inflection) were calculated with GraphPad Prism using non-linear fit of log (inhibitor) versus response (four parameter).

##### Analysis of percent remaining cells

DAPI fluorescence was applied for HCA-based quantification of the number of imaged cells on a plate-to-plate basis.

Percent remaining cells = (*X*_i_/*μ*_n_) × 100%

*μ*_n_ is the average number of nuclei of the vehicle control (TGF-β1 + 0.1% DMSO). *X*_i_ is the compound number of nuclei.

#### INS018_055 treatment of A549 cells in the TGF-β- and TNF-α-induced EMT assay

A549 cells were cultured in RPMI 1640 medium supplemented with 10% FBS and antibiotics (penicillin and streptomycin, 100 U ml^−1^ each) at 37 °C in a humidified atmosphere of 95% air and 5% CO_2_. A549 cells were seeded into six-well plates at 4 × 10^5^ cells per well. After overnight culture, the medium was replaced with serum-free medium for 24 h of starvation before cellular induction with TGF-β alone at a final concentration of 5 ng ml^−1^ or with TGF-β and TNF-α at a final concentration of 20 ng ml^−1^ in combination with INS018_055 or DMSO for an additional 48 h, respectively. At the end of induction and compound treatment, whole-cell lysates were prepared using RIPA buffer (Sigma, R0278) with protease inhibitor or nuclear and cytoplasm fractions were extracted using the Subcellular Protein Fractionation Kit (Thermo Fisher Scientific).

#### Knockdown of *TNIK* by short hairpin RNA in A549 cells

##### TNIK short hairpin RNA sequences and vector information

TNIK shRNA^79^ was cloned into the vector PLKO.1-puro by a CRO (GENEWIZ). Packaging plasmids (psPAX2, PCMV-VSV-G) were purchased from Addgene. Sequences were as follows: shRNA control (F, 5′-CCGGCAACAAGATGAAGAGCACCAACTCGAGTTGGTGCTCTTCATCTTGTTGTTTTT-3′; R, 5′-AATTAAAAACAACAAGATGAAGAGCACCAACTCGAGTTGGTGCTCTTCATCTTGTTG-3′), shTNIK-1 (F, 5′-CCGGGCTCCTAAACCGTATCATAAACTCGAGTTTATGATACGGTTTAGGAGCTTTTT-3′; R, 5′-AATTAAAAAGCTCCTAAACCGTATCATAAACTCGAGTTTATGATACGGTTTAGGAGC-3′), shTNIK-4 (F, 5′-CCGGGGGCAAGGCAAAGTCTATAATCTCGAGATTATAGACTTTGCCTTGCCCTTTTTG-3′; R, 5′-AATTCAAAAAGGGCAAGGCAAAGTCTATAATCTCGAGATTATAGACTTTGCCTTGCCC-3′).

##### Lentivirus packaging

Lentivirus were produced in 293T/17 cells transfected with PLKO.1-puro-shRNA (shRNA control, shTNIK-1 and shTNIK-4) using the Lipofectamine 3000 transfection kit. Next, 293T/17 cells were seeded in DMEM complete culture medium in a 10-cm tissue culture dish. Cells were incubated at 37 °C with 5% CO_2_ overnight. Mixture I was composed of 5 µg PLKO.1-puro-shRNA, 7.5 µg psPAX2, 2.5 µg PCMV-VSV-G, 30 µl P3000 reagent and 1,500 µl Opti-MEM. Mixture II was composed of 45 µl Lipofectamine 3000 and 1,500 μl Opti-MEM.

After a 5-min incubation at room temperature, mixture I and mixture II were mixed and incubated for another 20 min at room temperature to allow DNA–Lipofectamine 3000 complexes to form. DNA–Lipofectamine 3000 complexes were added to the 293T/17 culture plate and incubated overnight. The next day, medium containing the DNA–Lipofectamine 3000 complexes was removed and replaced with fresh culture medium. Cells were incubated at 37 °C for another 72 h in a CO_2_ incubator.

At 72 h after transfection, virus-containing supernatants were collected and filtered with a 0.45-µm filter. Viral stocks were aliquoted and stored at −80 °C.

##### Infection

RPMI 1640 medium supplement with 10% FBS and 1% penicillin–streptomycin was used for A549 culture medium. A549 cells were seeded at 2 × 10^6^ cells per plate in culture medium in a 10-cm dish and incubated with 5% CO_2_ at 37 °C overnight. The next day, the complete culture medium was replaced with fresh medium containing different virus ratios of 1/10 and 1/20. Virus at each ratio was used to treat cells in each dish. A total of 10 ml medium containing virus was added to each dish. Polybrene was added to each dish at a concentration of 6 μg ml^−1^, and dishes were incubated overnight. The next day, the medium was replaced with fresh culture medium, and the cells were incubated for another 48 h.

##### TGF-β treatment

After 2 d of incubation, the infected A549 cells were split into six-well plates at a density of 4.0 × 10^5^ cells per well in culture medium and cultured overnight. The next day, the medium was substituted with a serum-free medium for 24 h of starvation. A549 cells were treated in serum-free medium with or without 5 ng ml^−1^ TGF-β in duplicate for 48 h at 37 °C after starvation. At the end of induction, cells were lysed in RIPA buffer supplemented with protease inhibitor and Phosphatase Inhibitor Cocktails 2 and 3 (Sigma). The supernatant was stored at −80 °C after centrifugation. The total amount of protein in the lysate was determined using the BCA Protein Assay Kit. In experiment I, cell morphology was captured with a ×4 objective lens after induction and finally was magnified at ×40.

#### INS018_055 treatment of MRC-5 cells

MRC-5 cells were cultured in a monolayer with DMEM high-glucose medium supplemented with 10% FBS at 37 °C with 5% CO_2_. Cells were seeded in six-well plates in three replicate wells per condition at a density of 2.5 × 10^5^ cells per well and cultured for 24 h. The medium was replaced with serum-free medium, and cells were starved for 4 h. Afterward, the compound at eight concentrations (~100 μM–1.28 nM, fivefold gradient dilution) was added into each well. Vehicle (2% FBS–DMEM, containing 0.1% DMSO) was added into the blank control group and the model group, and pre-incubation was continued for 2 h. Next, TGF-β1 was added into each well of the administration group and the model group (final concentration in each well was 2 ng ml^−1^), and the same volume of vehicle was added into each well of the blank control group for 72 h of incubation (the culture medium was changed once after 48 h of incubation). After incubation, the cells were collected and digested with 0.5% trypsin, protein was extracted and total protein content was determined by the BCA method.

#### INS018_055 treatment of HK-2 cells

INS018-055 stock was diluted to eight concentrations in a threefold dilution with DMSO from the final highest concentration at 10 μM. A total of 3.0 × 10^5^ HK-2 cells per well were seeded into a six-well plate containing 2.0 ml culture medium at a density of 3.0 × 10^5^ cells per well. The cells were cultured overnight at 37 °C. The next day, the complete medium was replaced with K-SFM medium supplemented with 0.025 mg ml^−1^ bovine pituitary extract, 2.5 ng ml^−1^ EGF and 1% penicillin–streptomycin for starvation. After 24 h of incubation, HK-2 cells were treated with compounds (eight concentrations, threefold dilution) for 30 min before being stimulated with 8 ng ml^−1^ TGF-β for an additional 48 h. TGF-β induction was used as the maximum induction positive control. The final DMSO concentration in the assay was 0.1%. Cells were cultured for 48 h at 37 °C. The culture medium was discarded at the end of induction. Cells were washed once using ice-cold DPBS. RIPA buffer was added to lyse the cells for 20 min. Cells were collected, and the supernatant was stored at −80 °C. The total amount of protein in the lysate was determined using the BCA Protein Assay Kit.

#### Protein extraction and western blot

For whole-cell lysates, cells were collected and lysed on ice for 20 min with RIPA buffer with protease and phosphatase inhibitors (cOmplete and PhosSTOP, Roche). The protein concentration of the cell lysates was determined using the BCA Protein Assay Kit. After denaturation, whole-cell lysates and cytoplasm and nuclear extraction fractions were loaded onto 4–12% gradient Bis-Tris gels (15 wells) and 4–20% Tris-glycine gels (26 wells), respectively. For western blot detection of target proteins, 15 μg of whole-cell lysates, 5–10 μg of the cytoplasm fraction, 1–2 μg of nuclear matrix and 2–4 μg of the chromatin fraction were loaded into each lane. For western blot detection of histone 3, 0.4–1 μg of the chromatin fraction was loaded. The gels were run for 0.5 h at 80 V before being run at 120 V for another 1 h. When electrophoresis was completed, the gels containing target proteins were transferred onto NC membrane using the iBlot 2 Gel Transfer Device for 13 min at 20 V. All membranes were blocked with 5% BSA in TBST buffer at room temperature for 1 h and then incubated with primary antibodies specific to the indicated targets in TBST buffer containing 5% BSA at 4 °C overnight. After the primary antibody, membranes were washed three times with TBST buffer before incubation with HRP-conjugated secondary antibodies at room temperature for 1 h. Membranes were stripped with Restore Western Blot Stripping Buffer (Thermo Fisher) for GAPDH reference protein detection after exposure for the target proteins. Images of blots were acquired using Image Quant LAS 4000. Integrated intensity of bands from 16-bit blot images was used for quantitation with the software Image Studio Lite. Protein-quantification data of two independent experiments were calculated and analyzed using GraphPad.

#### α-SMA evaluation in normal human dermal fibroblasts

NHDF cells were seeded in a 96-well plate and cultured for 24 h. The medium was then replaced with an assay medium containing or lacking (control conditions) the test compound, and TGF-β (0.1 ng ml^−1^) was added or not (unstimulated control). The cells were then incubated for 72 h. All experimental conditions were performed in five replicates. For in situ immunolabeling and image analysis, at the end of the incubation, fibroblasts were rinsed, fixed and permeabilized. The cells were then labeled using a primary antibody (anti-α-SMA), followed by the corresponding fluorescent secondary antibody (GAM Alexa 488). In parallel, cell nuclei were stained using Hoechst solution 33258 (bisbenzimide). Image acquisition was performed using a high-resolution imaging system, the IN Cell Analyzer 2200 (GE Healthcare) automated microscope (objective lens, ×20). Five pictures per well were taken. Labeling was quantified by measuring the fluorescence intensity of α-SMA signals normalized to the total number of nuclei identified (integration of numerical data was performed with the Developer Toolbox 1.5, GE Healthcare software).

#### Fibronectin and procollagen I evaluation in NHDFs

NHDF cells were seeded in 96-well plates and cultured for 24 h. The medium was then replaced with assay medium containing or not containing (control conditions) the test compounds, and TGF-β (10 ng ml^−1^) was added or not (unstimulated control). The cells were then incubated for 72 h. Fibronectin and procollagen I contents were measured in the culture supernatants using specific ELISA kits (Takara, MK115 for fibronectin and MK101 for procollagen type I) according to the supplier’s instructions.

#### Cell viability

MRC-5 cells were seeded at 8,000 cells per well in 10% FBS medium and cultured with concentrations diluted from 100 μM for 72 h at 37 °C with 5% CO_2_. A549 cells were seeded at 5,000 cells per well in 10% FBS medium and cultured with concentrations diluted from 50 μM for 72 h at 37 °C with 5% CO_2_. HK-2 cells were seeded at 8,000 cells per well in 10% FBS medium and cultured with concentrations diluted from 50 μM for 72 h at 37 °C with 5% CO_2_.

CellTiter-Glo (Promega) was added to all conditions followed by 15 min of incubation at room temperature. Luminescent signal was detected with PerkinElmer EnVision. Data analysis was performed by calculating cell viability (%) with luminescent signal: cell viability (%) = (luminescent signal of sample − mean luminescent signal of medium)/(mean luminescent signal of cell control − mean luminescent signal of medium) × 100. CC_50_ values were determined according to the fitting curve of the emission ratio versus log (compound concentration) with GraphPad Prism.

### Animal studies

#### Mice

For the bleomycin-induced lung fibrosis model and the LPS-induced acute lung injury model, C57BL/6 mice (male, 8 weeks old, ~23–26 g) were purchased from Jiangsu Gempharmatech. Animals were acclimatized for 1 week before the experiment. All in vivo experimental procedures were approved by the IACUC. All euthanasia was performed using CO_2_ inhalation, and all efforts were made to minimize animal suffering.

For the UUO study, 7-week-old female C57BL/6 mice were obtained from Japan SLC. Animals were housed and fed a normal diet (CE-2, CLEA Japan) under controlled conditions. All animals used in the study were housed and cared for following the Japanese Pharmacological Society Guidelines for Animal Use.

### Animal models

#### Preparation for dosing formulations for animal model studies

For bleomycin formulation for lung fibrosis, bleomycin was dissolved in saline and vortexed to obtain a clear solution. For bleomycin formulation for the scarring model, bleomycin was dissolved in PBS. For LPS formulation, LPS was dissolved in saline and vortexed to obtain a clear solution. INS018_055 (p.o.) treatment solutions were formulated in 0.5% methylcellulose, and formulations were prepared daily. INS018_055 (topical) was formulated in 15% DMSO, 41% Transcutol, 10% IPM, 8% glycerol, 8% oleic acid, 8% diisopropyl adipate, 3% Brij L4, 4% cetostearyl alcohol and 2% hydroxypropyl cellulose (wt/wt%). Nintedanib treatment solutions were formulated in 0.5% methylcellulose. Homogeneous SB525334 suspensions were prepared in 5% DMSO + 95% (0.5% HPMC and 0.1% Tween-80 in reverse-osmosis (RO) water), and formulations were freshly prepared before administration. Dexamethasone was formulated in 7.70 ml saline and kept on ice before use. Rapamycin was prepared in 0.2% sodium carboxymethyl cellulose and 0.25% polysorbate 80.

#### LPS-induced acute lung injury model

One day before the first experimental day, animals were grouped. All mice were anesthetized with Zoletil–xylazine (50 mpk and 10 mpk, i.p.). At 0 h, mice received LPS at a dose of 50 μl as 50 μg per mouse (groups 2–5, *n* = 8 per group) or 50 μl saline (group 1, *n* = 8 per group) by intratracheal administration. In the INS018_055 groups (groups 3 and 4), animals were treated with INS018_055 for 4 h after the LPS challenge. In the dexamethasone group (group 5), animals were treated with dexamethasone both 2 h before and 4 h after the LPS challenge. Twenty-four hours after the LPS challenge, animals were euthanized, and BALF samples were collected.

#### Bleomycin lung mouse model

Mice received bleomycin on day 1 at a dose of 0.66 mg per kg (equivalent to 1 U per kg), in a volume of 50 μl by intratracheal administration, or received saline (sham group). On day 7, dosing with the corresponding compounds was started. The vehicle group (group 1) was treated with 0.5% methylcellulose BID. Groups 2–4 were treated with INS018_055 at dosages of 3 mg per kg, 10 mg per kg and 30 mg per kg BID, respectively. Group 5 was treated with 60 mg per kg nintedanib QD. On days 5 and 21, lung function measurement was performed. On day 28, all animals were killed, and blood and tissue samples were collected along with BALF. The method of model development and study design of combination studies of INS018_055 on bleomycin-induced lung fibrosis mouse models were similar.

##### Lung function measures in the bleomycin-induced lung fibrosis mouse model

Lung function was measured according to the HDB standard protocol. Expiratory time, relaxation time, peak expiratory flow and peak inspiratory flow were collected and used to calculate Penh data.

#### Inhalation administration bleomycin-induced lung fibrosis rat model

On day 1 of the study, bleomycin hydrochloride was administered to 8–9-week-old Sprague Dawley rats at a final concentration of 1.5 mg ml^−1^ in sodium chloride solution (dosed volume at 1.0 ml per kg) by intratracheal atomization (once in the morning and once in the afternoon) to build the pulmonary fibrosis model. INS018_055 inhalation solutions were prepared at 0.1 mg ml^−1^, 0.3 mg ml^−1^, 1.0 mg ml^−1^ and 6.0 mg ml^−1^ in sodium citrate buffer.

##### Aerosol generation and environmental conditions

A German PARI compression atomizer (PARI TurboBOY) was set at an aerosol flow rate of 9–10 l min^−1^. Aerosols from INS018_055 solutions were generated in single-chamber mode. The dilution flow of aerosol was set at 0 l min^−1^, and the pumping-out flow from the chamber was set at 6 l min^−1^. The fluid rate was set at 0.5 ml min^−1^ for test article replenishment. Before nebulization, aerosol-generation conditions were validated three times.

In this experiment, temperature was set at 20–26 °C, humidity between 30% and 80%, O_2_ level ≥ 19% and CO_2_ level ≤ 1%. And these parameters were recorded in real time during the experiment.

Aerosols of INS018_055 solutions were sampled and analyzed for concentrations and sizes of aerosol particles. For concentration assessment, a glass fiber filter membrane (*φ* = 37 mm) was used to measure aerosol concentrations during drug administration from approximately day 8 to day 28. Sampling was taken at 5 min (±1 min) after drug administration from a random exposure port. Sampling flow for the solution of 0.1 mg ml^−1^ INS018_055 was 1 l min^−1^ for 10 min. Sampling flow for solutions of 0.3 mg ml^−1^, 1.0 mg ml^−1^ and 6.0 mg ml^−1^ INS018_055 was 1 l min^−1^ for 5 min. For particle size assessment, during administration on day 8, day 15, day 22 and day 28, an exposure port was selected randomly and connected with a next-generation impactor for sampling (MOC disc filter paper) for aerosol particle size measurement at a sampling flow rate of 15 l min^−1^ for 5 min. Aerosol particle size parameters were calculated with the system software: median mass aerodynamic diameter (MMAD), geometric standard deviation, fine particle fraction (percentage of fine particles with MMAD < 5 μm), etc. The actual delivered dose and parameters of aerosol particle size distribution are:

**Table:** 

Index		0.1 mg ml^−1^	0.3 mg ml^−1^	1.0 mg ml^−1^	6.0 mg ml^−1^
Actual delivered dose (mg/kg)^a^		0.040 ± 0.008	0.136 ± 0.017	0.485 ± 0.081	2.575 ± 0.242
NGI	MMAD (μm)	2.747 ± 0.317	2.580 ± 0.077	2.435 ± 0.053	2.5175 ± 0.167
	GSD	1.314 ± 0.111	1.760 ± 0.036	1.856 ± 0.121	1.957 ± 0.066
	FPF (%)	99.935 ± 0.007	85.454 ± 4.675	80.866 ± 5.827	80.294 ± 4.187

^a^Actual delivered dose was calculated as follows:

$${\mathrm{Dose}}\ ({\mathrm{mg}}\ {\mathrm{per}}\ {\mathrm{kg}}) = \frac{C (\upmu{\mathrm{g}}\ {\mathrm{l}}^{-1}) \times {\mathrm{RMV}} ({\mathrm{l}}\ {\mathrm{min}}^{-1}) \times D ({\mathrm{min}}) \times {\mathrm{DF}}}{{\mathrm{BW}}\ ({\mathrm{kg}}) \times 1,000}.$$

*C*, concentration (µg l^−1^) in air inhaled; RMV, respiratory minute volume (l min^−1^); RMV (l min^−1^) = 0.608 × BW (kg)^0.852^; *D*, duration of exposure (min); DF, deposition fraction, assumed as 100% for calculation of the actual delivered dose; BW, body weight (kg); FPF, fine particle fraction; GSD, geometric standard deviation; NGI, next-generation impactor.

##### Measurement of lung function in the lung fibrosis rat model

On day 29 and before euthanasia, animals were anesthetized with chloral hydrate (90 mg ml^−1^, 5 ml per kg, i.p.). Necks were cut longitudinally at the middle to isolate trachea, and a catheter was intubated into the trachea toward the tail direction and fixed with a suture thread. The anesthetized animals were placed into the plethysmography chamber, to which the airway was connected. The AniRes2005 animal lung function-analysis system was used to assess parameters including FVC, airway resistance and pulmonary compliance. The ventilator respiration rate was set to 65 beats per minute; the respiration ratio was set to 20:10; the negative pressure controller was set to 30 cm H_2_O. FVC detection was set to pressure-control mode, while pressure was set to 30 cm H_2_O. The startup mode was selected to automatically detect at the end of breath, and the ‘Start’ button was clicked to passively inhale and then turned to exhale after reaching the set pressure value. Next, the ‘Stop’ button was clicked to complete an FVC detection. Each animal was evaluated for FVC at least five times. The final result was the mean value after the maximum and minimum data were excluded for analysis.

#### Unilateral urethral obstruction model in mice

The surgery took place on two separate days. Mice were divided into two slots based on their body weight before the day of the surgery. On day 0 (day of surgery), UUO surgery was performed after administration of three types of mixed anesthetic agents (medetomidine, midazolam, butorphanol). Mice were shaved at the incision site, the abdomen was cut open, and the left ureter was exteriorized and ligated with 4-0 silk sutures at two points. Finally, the peritoneum and the skin were closed with sutures.

Group 1 was sham-operated mice, and group 2 was treated with vehicle BID. Groups 3–5 were treated with INS018_055 at a dose of 3 mg per kg, 10 mg per kg and 30 mg per kg BID, respectively. Group 6 was treated with the ALK5 inhibitor SB525334 at a dose of 100 mg per kg QD. Treatments were applied from day 0 to day 14. And animals were killed on day 14, and blood and tissue samples were collected.

#### Bleomycin-induced scarring model in rats

Animals were anesthetized using isoflurane. Using a 1-ml syringe containing a 27-gauge needle, 100 µl bleomycin (1 mg ml^−1^ in PBS) solution was injected subcutaneously into two sites on the shaved dorsal regions, QD for 4 weeks. Rats in the naive control group were injected daily with an equivalent volume of sterile PBS. Treatments, vehicle (topical), rapamycin (1.5 mg per kg, i.p.) or INS018_055 (0.05%, 0.15% or 0.45%, topical) were administered 30 min before bleomycin administration, QD for 4 weeks. After 4 weeks of treatment, animals were killed with CO_2_, and skin was collected.

#### Bronchoalveolar lavage fluid collection

Mice were first anesthetized with Zoletil–xylazine (50 mpk and 10 mpk, i.p.). Next, a 1.5–2-cm longitudinal incision was made on the ventral side of the neck to expose the trachea by blunt dissection, and curved forceps were placed underneath the trachea. A 10–12-cm piece of suture was then threaded underneath the trachea using forceps. The ends of the suture were then pulled cranially to ‘stretch’ the trachea toward the surgeon. A 20-G needle was then used to poke a hole in the trachea as close to the larynx as possible. A 22-G blunt stainless steel needle connected to PE tubing was placed in the hole and inserted approximately 5–8 mm into the trachea. The cannula was secured in the trachea by tying the suture around it with an overhand knot. A 1-ml syringe containing 0.4 ml saline was gently pushed forward and backward three times to recover BALF fluid. BALF was transferred into empty tubes and stored on ice. This process was repeated twice with 0.3 ml saline for a total volume of 1 ml.

#### BALF cytological analysis

Total cell numbers were counted with a standard hemocytometer by mixing 20 μl BALF with 20 μl trypan blue. Cytospins were prepared by cytocentrifugation using Cytospins at 106*g* for 5 min, and then smears of BALF cells were stained with Diff-Quick stain. Cell differentiation was performed by counting at least 400 cells using standard hemocytologic criteria to classify them as alveolar macrophages or monocytes, neutrophils, eosinophils or lymphocytes. The rest of the BALF fluid was centrifuged, and supernatants were collected and stored at −80 °C for cytokine, chemokine and total protein analysis.

#### BALF cytokine and chemokine analysis

From the collected BALF, concentrations of cytokines were measured from mice in groups 2–7 killed on day 28. Mouse BALF cytokines and chemokines were measured using the ELISA Analysis Kit (Sinobest Bio). Mouse BALF soluble collagen was measured using the Soluble Collagen kit (Biocolor). Mouse lung hydroxyproline content was measured using the Hydroxyproline Assay Kit (Njjcbio).

#### Kidney hydroxyproline content

Frozen kidney samples were processed by an alkaline acid hydrolysis method as follows. Kidney samples were dissolved in 2 N NaOH at 65 °C and autoclaved at 121 °C for 20 min. The lysed samples (400 μl) were acid hydrolyzed with 400 μl of 6 N HCl at 121 °C for 20 min and neutralized with 400 μl of 4 N NaOH containing 10 mg ml^−1^ activated carbon. Next, AC buffer (2.2 M acetic acid and 0.48 M citric acid, 400 μl) was added to the samples, followed by centrifugation to collect the supernatant. The prepared samples and standards (serial dilutions of *trans*-4-hydroxy-l-proline (Sigma-Aldrich) starting at 16 μg ml^−1^, each 400 μl) were mixed with 400 μl chloramine T solution (Nacalai Tesque) and incubated for 25 min at room temperature. The samples were then mixed with Ehrlich’s solution (400 μl) and heated at 65 °C for 20 min to develop color. After samples were cooled on ice and centrifuged to remove precipitates, the optical density of each supernatant was measured at 560 nm. Hydroxyproline concentrations were calculated from the hydroxyproline standard curve. Kidney hydroxyproline contents were expressed as μg per mg protein, and the amount of protein was determined using the BCA Protein Assay Kit (Thermo Fisher Scientific).

#### Hematoxylin and eosin staining and quantification (lung)

Left lung lobes were removed from each mouse and fixed in 10% NBF solution, embedded in paraffin and processed to obtain 4-μm sections for staining with H&E. H&E-stained slides were scanned using an Aperio ScanScope Model CS2 (Leica) at ×200 magnification. Images were then opened in the HALO Plus 5 workstation (version 2.3, Indica Labs) program. Using the pen annotation tool, the whole-lung section was selected as an annotation layer. The area of vessels was defined as background using the exclusion drawing tool. The total inflammation area was quantified by selecting the dark blue inflammation cells using the pen annotation tool manually. The percentage of H&E staining (inflammation area/total area of the lung) in the selected annotation was then calculated using the program.

#### Masson trichrome staining and quantification (lung)

Masson’s trichrome staining was conducted using a ready-to-use kit (Trichrome Stain (Masson) Kit, HT15, Sigma-Aldrich) as described by the manufacturer.

Lung sections were cut at a thickness of 4 μm, dried in an oven for 1 h and stained with M&T using our standard protocol. Briefly, sections were stained with Weigert’s iron hematoxylin working solution for 10 min, stained in Biebrich scarlet–acid fuchsin solution for 10 min, differentiated in phosphomolybdic–phosphotungstic acid solution for 5 min or until the collagen was not red, transferred to aniline blue solution, stained for 1 min and then dedifferentiated in 1% acetic acid solution. Next, sections were dehydrated and coverslipped for subsequent image analysis. For image analysis of collagen deposition, Masson’s trichrome-stained slides were scanned by using Aperio ScanScope Model CS2 (Leica) at ×200 magnification. Images were then opened with HALO. Using the pen tool, the whole left lung section was selected as an annotation layer. The area occupied by blue collagen fibers was measured using the pen tool. The percentage of fibrosis (positive areas) in the selected annotation was then calculated with the program. Fibrosis was expressed as a percentage per lung section.

#### Sirius red staining and quantification (kidney)

At the end of the UUO study, the left kidney was fixed in Bouin’s solution and embedded in paraffin. Kidney sections were stained using picro-Sirius red solution (Waldeck). To quantify the interstitial fibrosis area, brightfield images in the corticomedullary region were captured using a digital camera (DFC295) at 200-fold magnification, and the positive areas in five fields per section were measured using ImageJ software (National Institute of Health).

#### Immunohistochemistry staining and quantification of lung tissue

Sections (4 μm thick) were placed on slides, and, after overnight drying, paraffin was removed with xylene. Next, sections were placed in a graded ethanol series and immersed in distilled water. After heat-induced citrate antigen (pH 6.0) unmasking, sections were immersed in 3% hydrogen peroxide solution for 5 min. The sections were then incubated in blocking serum (Dako, X0909) for 15 min at room temperature, followed by using primary rabbit antibodies (anti-α-SMA and anti-collagen I) for 1 h. Next, secondary antibodies conjugated to HRP were added. For image analysis of fibrosis, stained sections were used and scanned with the Aperio CS2 Scanner machine. Images were then opened with HALO. Using the pen tool, the whole left lung section was selected as an annotation layer. The bronchus was excluded in the annotation layer. The area occupied by collagen fibers was measured using the ‘Area Quantification v2.1.3’ module. The percentage of positive areas in the selected annotation was then calculated using the program.

#### Immunohistochemistry staining and quantification of kidney tissue

Paraffin sections were deparaffinized and hydrophilized with xylene, 100–70% alcohol series and RO water, and then circles were drawn around the kidney sections. Endogenous peroxidase activity was blocked using 0.3% H_2_O_2_ for 5 min, and antigen retrieval was performed using antigen retrieval solution H (citrate buffer) at 121 °C for 10 min. After washing with PBS, kidney sections were treated with PBS with Tween-20 (PBST), followed by incubation with Block Ace (DS Pharma Biomedical) at room temperature for 10 min. The sections were incubated with the primary antibody (anti-collagen I) at 4 °C overnight. After washing with PBS, kidney sections were treated with PBST, followed by incubation with secondary antibody at room temperature for 30 min. After washing with PBS, kidney sections were fixed with 1% glutaraldehyde solution at room temperature for 1 min. After washing with RO water, kidney sections were treated with PBST and then colored using a chromogenic substrate (Simple Stain DAB, Nichirei Bioscience). After washing with RO water, kidney sections were immersed in an eightfold-diluted hematoxylin solution for 1 min and washed with RO water immediately. The stained sections were placed in running water for 15 min and sealed with Aquatex (Merck). For scoring of IHC analyses, brightfield images were captured using a digital camera (DFC295) at 200-fold magnification, and the score in one field per section was determined.

#### Evaluation of hydroxyproline and collagen content in skin

Hydroxyproline and collagen contents were measured in skin lysates using rat hydroxyproline (KinesisDx, K11-0512) and collagen (Abcam, ab222942) ELISA kits. Hydroxyproline and collagen concentrations were normalized to total protein content using the Bradford method.

### Statistical analysis

For western blot figures, *P* values between groups were analyzed by Welch’s *t*-test among three independent experiments. *P* values < 0.05 were considered statistically significant. For LPS and bleomycin mouse models, statistical analysis was performed using ordinary one-way-ANOVA, and post hoc Šídák’s multiple-comparison test was performed to calculate statistical analysis between groups. The difference was considered significant when *P* < 0.05. For the inhalation study in the bleomycin rat model, in analyzing parameters of lung functions (FVC, airway resistance and pulmonary compliance), statistical analysis was performed using uncorrected Fisher’s least-significant difference test as post hoc analysis after ANOVA analysis; in analyzing pathology results, statistical analysis was performed using Kruskal–Wallis test and Dunn’s multiple-comparison test as post hoc analysis after ANOVA analysis. Differences were considered significant when *P* < 0.05. For the UUO model, statistical analyses were performed using the Bonferroni multiple-comparison test. *P* values < 0.05 were considered statistically significant. For the bleomycin-induced skin scarring experiment, statistical analysis was performed using one-way ANOVA, and then Dunnett’s multiple-comparison test was performed. *P* values < 0.05 were considered statistically significant. For Fig. 2h, enrichment analysis was performed using the gseapy.enrichr Python package. *P* values were computed using Fisher’s exact test. This is a binomial proportion test that assumes a binomial distribution and independence for probability of any gene belonging to any set. Adjusted *P* values (*q* values) were calculated using the Benjamini–Hochberg method for correction for multiple-hypothesis testing.

### Single-cell RNA sequencing analysis

Single-cell RNA sequencing (scRNA-seq) data from 32 IPF and 28 control lungs were collected from the GEO database (GEO accession GSE136831)^53^. Data were preprocessed with a standard pipeline using the Scanpy package. Single-cell data was filtered by applying two conditions: including cells with a minimum of 200 genes and with genes expressed in more than three cells. Cells with mitochondrial fraction greater than 20% were excluded. Data were initially normalized with a scale factor of 10,000 and log transformed. PCA was run with the sc.pp.pca function and ‘n_comps’ = 50. Batch effects were corrected with ‘sce.pp.harmony_integrate’ function. A neighborhood graph was computed on the first 50 principal components derived after batch correction, and results were visualized using UMAP. Cell type annotation was obtained from the original article. Cluster gene signatures were identified by testing for differential expression of a subgroup against all other cells using a Wilcoxon rank-sum with the ‘tl.rank_genes_groups’ function. Differential expression between IPF and controls for each cell type was calculated using the ‘tl.rank_genes_groups function’ (method = ‘Wilcoxon’), and *TNIK* expression was plotted (non zero values with *P* values, Benjamini–Hochberg adjustment and a threshold of 0.05) using the plotly package.

### Simulated knockout profile of *TNIK*

scTenifoldKnk^54^ is a method developed to perform virtual knockout experiments to predict gene functions. First, scTenifoldKnk, based on scRNA-seq data, constructs a denoised single-cell gene regulatory network (scGRN). The scGRN is copied, and then outward edges of the target gene in the adjacency matrix of the copied scGRN are zeroed out, thus creating a pseudo-knockout scGRN. Having two scGRNs (one initial net and a second pseudo-knockout net), target gene regulatory significance can be estimated with a manifold alignment procedure. Two scGRNs are mapped to the same low-dimensional space, and distance between gene projections shows the impact of gene knockout in the scGRN: larger perturbation of genes in low-dimensional space indicates the importance of the target gene in the scGRN. We used IPF myofibroblast scRNA-seq data derived from GSE136831 (ref. ^53^) in scTenifoldKnk and made a pseudo-knockout of *TNIK*. The list of perturbed genes was sorted by fold changes of distances between gene projections of two scGRNs; probabilities were assigned using *χ*^2^ distribution with one degree of freedom. The most perturbed genes are supposed to have a tight connection with the target gene. Next, we applied the MCODE algorithm^47^ for *TNIK* and the top 100 most perturbed genes and performed pathway and process enrichment analysis.

### Chemistry42

The Molecular Sets platform (MOSES)^87^ was used to train and benchmark the generative chemistry models in Chemistry42. The structure-based drug-design workflow implemented in the Chemistry42 platform was used to generate a library of virtual structures^40^. Pocket and Pharmacophore Reward modules were used for scoring produced designs and navigating the generative process. The TNIK ATP-binding site was selected as a target-binding pocket. The generated structures had to match a two-point pharmacophore hypothesis that consisted of the hydrogen bond acceptor forming an H-bond with the NH of Cys108 of the hinge region and hydrophobic center occupying the space near the gatekeeper Met105. In addition to the assessment by Pocket and Pharmacophore modules, the engine penalized structures that violated the predefined ranges of the physicochemical properties (logP, molecular weight, number of hydrogen bond donors, hydrogen bond acceptor, topological polar surface area, number of atoms, number of rotatable bonds, number of aromatic rings), medicinal chemistry filters and the synthetic accessibility score threshold.

### Phase 0 study (Australia)

A phase 0 micro dosing clinical trial was conducted in Australia (ACTRN12621001541897) and can be found at https://www.anzctr.org.au/TrialSearch.aspx. Complete information on clinical trial registration, study protocol, data collection and outcomes is provided in the Reporting summary as well as in Supplementary Information 8. This includes statistical considerations, study design, patient-selection criteria, procedures, outcomes and PK analysis.

### Phase I study (New Zealand)

The general design of this clinical trial (NCT05154240) can be found at https://www.clinicaltrials.gov. The randomized, double-blind, placebo-controlled study of INS018_055 was conducted from 21 February 2022 (first participant administered first dose) until 30 September 2022 (last participant contacted). Complete information on clinical trial registration, study protocol, data collection and outcomes is provided in the Reporting summary as well as in Supplementary Information 9. This includes statistical considerations, study design, patient-selection criteria, procedures, outcomes and PK analysis.

### Phase I study (China)

Detailed information including facilities and inclusion and exclusion criteria can be found at the following link: http://www.chinadrugtrials.org.cn/clinicaltrials.prosearch.dhtml (registration number CTR20221542).

Complete information on clinical trial registration, study protocol, data collection and outcomes are provided in the Reporting summary as well as in Supplementary Information 10. This includes statistical considerations, study design, patient-selection criteria, procedures, outcomes and PK analysis.

Full clinical study protocols for all three trials are provided in the Supplementary Information.

### Reporting summary

Further information on research design is available in the Nature Portfolio Reporting Summary linked to this article.

## Online content

Any methods, additional references, Nature Portfolio reporting summaries, source data, extended data, supplementary information, acknowledgements, peer review information; details of author contributions and competing interests; and statements of data and code availability are available at 10.1038/s41587-024-02143-0.

## Supplementary information

Supplementary Information Supplementary Information 1–10 and Clinical Study Protocols for Australia Phase 0, New Zealand Phase I and China Phase I

Reporting Summary

Supplementary Video 1 A demonstration of TNIK identification in IPF using PandaOmics.

## Source data

Source Data Figs. 2 and 5 and Extended Data Figs. 5 and 6 Unprocessed western blots for Figs. 2 and 5 and Extended Data Figs. 5 and 6.

Source Data Fig. 3 Statistical source data.

Source Data Fig. 4 Statistical source data.

Source Data Fig. 5 Statistical source data.

Source Data Fig. 6 Statistical source data.

Source Data Extended Data Fig. 3 Statistical source data.

Source Data Extended Data Fig. 5 Statistical source data.

Source Data Extended Data Fig. 6 Statistical source data.

Source Data Extended Data Fig. 7 Statistical source data.

Source Data Extended Data Fig. 8 Statistical source data.

Source Data Extended Data Fig. 9 Statistical source data.

Source Data Extended Data Fig. 10 Statistical source data.

## Data Availability

Raw data from all experimental studies are available for public access through our repository, which can be found at https://insilico.com/repository/nbt-ins018-055-tnik. RNA sequencing data are available for download through the repository link along with all relevant source and raw data that were presented in this study. The molecular sets used for training are available through our open-access benchmarking platform MOSES^87^. For the current study, target ID scoring was calculated on 15 IPF datasets collected from GEO database. A list of used datasets is as follows: GSE93606, GSE38958, GSE28042 and GSE33566 derived from blood tissue and GSE101286, GSE72073, GSE150910, GSE92592, GSE52463, GSE83717, GSE21369, GSE15197, GSE99621, GSE138283 and GSE24206 derived from lung tissue. GSE136831 was used for scRNA-seq analysis (Single-cell RNA sequencing analysis). Other data that support the findings of this study, including clinical data, are available from the corresponding author upon reasonable request. Source data are provided with this paper.

## Code availability

Code for benchmarking of the generative chemistry models is available in the provided reference^87^. PandaOmics and Chemistry42 are industry-grade commercial software platforms used by the industry since 2020. Both platforms are available at https://pandaomics.com and http://chemistry42.com, respectively. A detailed algorithmic workflow for running the platforms is described in the Methods and Supplementary Information. Demonstrations and trial access to the platforms are available from the corresponding author upon reasonable request.
