# Supplementary material for: A small-molecule TNIK inhibitor targets fibrosis in preclinical and clinical models
Source: Nat Biotechnol. 2024 Mar 8;43(1):63–75. doi: 10.1038/s41587-024-02143-0 (PMC11738990; doi:10.1038/s41587-024-02143-0)
Supplement: Supplementary file 1 — Supplementary Information 1–10 and Clinical Study Protocols for Australia Phase 0, New Zealand Phase I and China Phase I [file 41587_2024_2143_MOESM1_ESM.pdf]

nature biotechnology

Article https://doi.org/10.1038/s41587-024-02143-0

# A small-molecule TNIK inhibitor targets fibrosis in preclinical and clinical models

In the format provided by the authors and unedited

**Supplementary information 1: Notes for PandaOmics and Node degree Control for Target ID**

In silico-based PandaOmics target discovery/scoring (Target ID) approach was applied to identify novel molecular targets for idiopathic pulmonary fibrosis (IPF). This approach is based on the combination of multiple scores derived from text and omics data. Text-based scores are derived from various sources including scientific publications, grants, patents, clinical trials and the key opinion leaders, and thus represent how strongly a particular target is associated with a disease. Specifically, text-based scores contain Attention, Trend, Attention Spike, Evidence, Grant funding, Funding per Publication, Grant Size, Average Hirsch, Impact Factor and Credibility attention index scores. In contrast, omics scores are based on the differential expression, GWAS studies, somatic and germline mutations, interactome topology, signaling pathway perturbation analysis algorithms, knockout/overexpression experiments and more omics-data sources, and thus represent the target-diseases association according to molecular connections between proposed target and disease of interest. Omics scores include thirteen models (Heterogeneous Graph Walk, Matrix Factorization, Interactome Community, Causal inference, Overexpression/Knockout, Mutated/Disease Sub-modules, Mutations, Pathway, Network Neighbors, Relevance and Expression) that can be subdivided into classic bioinformatics approaches and complex AI-based models. All the models regardless of the particular methodology output the ranked lists of target hypotheses. Combination of described scores results in a ranked list of targets proposed for a given disease and can be filtered out in regard to their novelty, small molecules synthesis availability, availability of PDB structure and other useful filters. Description of all mentioned scores and filters is available in the User manual section of PandaOmics (https://insilico.com/pandaomics/help).

Target ID: PandaOmics (https://pandaomics.com) platform was used to conduct a target hypothesis generation for IPF, meta-analysis combined the data from 15 experiments resulting in 491 disease samples and 292 healthy controls. All used datasets are publicly available in GEO database and listed below: GSE93606, GSE38958, GSE28042, GSE33566 are the datasets derived from blood tissue and GSE101286, GSE72073, GSE150910, GSE92592, GSE52463, GSE83717, GSE21369, GSE15197, GSE99621, GSE138283, GSE24206 are the datasets derived from lung tissue. The gene expression datasets were processed according to the platform-specific protocols. Differential expression analysis for all datasets was performed using the Limma package. Obtained gene-wise p-values were corrected by the Benjamini-Hochberg procedure (FDR). The resulting gene expression comparisons were combined into one meta-analysis that was further utilized for the identification of novel targets. Novel kinase (causal) scenario was used to obtain a ranked list of potential first-in-class kinase targets. In IPF targets identification, we restricted the small molecule filter to the highest level and the Safety and

Novelty filter to the medium level. Only targets that belong to protein kinase or receptor kinase and with available structures were included in the calculation. Targets with activity in DrugCentral with known MoA (TClin) were excluded. We used five omics scores models to obtain the ranked list of targets, which are Mutated sub-modules, Causal inference, Expression, Heterogeneous graph walk, and Matrix factorization. All the models regardless of the particular methodology output the ranked lists of target hypotheses. The outputs of multiple models was further combined into a single aggregated ranking. Text-based, Financial, and KOL models were turned off because we wanted to find novel targets that we should not rely on literature. Target rankings were calculated under these settings. Well-known hub genes were eliminated using a described combination of filters. As the last step targets with the narrow expression in the tissues irrelevant to the fibrotic indications were excluded including BMX excluded due to its high tissue expression specificity in the epididymis (See Supplementary material: PandaOmics screencast). TNIK was identified as the number 1 target in the target list under this setting.

We chose the 5 omics models because they are more likely to find potential causal targets. The descriptions of these models are listed below:

Mutated sub-modules **(Extended Data Fig. 1A)**: This score estimates target relevance for the process/disease phenotype based on the diffusion signal propagation method through the PPI graph. STRINGdb is used for PPI network, filtered by the combined score of 800. Additionally, ubiquitins and isolated nodes are removed from the resulting PPI graph. The score relies on a manually curated database and the data coming from external sources (OMIM, ClinVar, Open Targets). Mutated sub-modules score utilizes the NHGRI-EBI GWAS Catalog database (https://www.ebi.ac.uk/gwas/), with a cutoff for Genome-wide significance applied. The loci are mapped to the gene symbols according to the Ensembl mapping pipeline (https://www.ebi.ac.uk/gwas/docs/faq#faq-E4).

Causal inference **(Extended Data Fig. 1B)**: This score is based on the causal inference of transcription factors. It estimates the number of genes associated with the disease progression/treatment, controlled by a similar set of transcription factors to a given gene. It uses a manually curated regulatory network and known drug targets to predict potential disease-modifying transcription factors. The Causal Inference score gives a higher rank to the genes regulated by the transcription factors perturbed in the analyzed datasets. We use binomial test p-value to estimate the statistical significance of the enrichment given the degree of the particular transcriptional factor and regulated gene.

Expression **(Extended Data Fig. 1C)**: Expression relies on the differential gene expression analysis defined by a collection of datasets of interest. Machine learning-based

models are used to normalize gene expression data available across multiple samples from unrelated datasets.

Heterogeneous graph walk **(Extended Data Fig. 1D)**: Heterogeneous Graph Walk is a guided random walk-based approach that is applied to a heterogeneous graph. The model learns node representations and then finds gene nodes close to the reference disease node. First, the "walks" are sampled with a predefined meta-path, i.e. fixed sequence of node types in a walk, e.g. 'gene'-'disease'-'gene.' The node degree controls the probability of transition between the nodes while sampling. The transition probability is adjusted for the node degree as 1/(node degree). Following that, the SkipGram model learns the representation of each node based on the resulting corpus of walks. The cosine similarity between the specific disease and all genes produces a ranked list of genes. The top genes from this list are predicted to be promising target hypotheses.

Matrix factorization **(Extended Data Fig. 1E)**: Matrix Factorization is a collaborative filtering algorithm widely used in recommender systems. The algorithm decomposes a sparse matrix derived from a gene-disease interaction graph into two lower dimensionality matrices that consist of latent factors for genes and diseases. The algorithm uses graph regularization based on a fast kNN search to account for intraclass similarity between the nodes of a similar type. Recomputing the original interaction matrix from latent factors provides the scores for unobserved interactions; thus, gene ranking is obtained.

The details about the entire set of the Target ID scores used can be found in PandaOmics manual (https://insilico.com/pandaomics/help). All the Target ID scores are calculated in the context of a certain disease.

Additionally to prove the validity of the approach, the relationship between node degree and the scores utilized for gene ranking in the novel kinase (causal) PandaOmics setting was studied. As depicted in (Supplementary 1, Figure, A, B), a correlation between the score and the node degree in the first and second vicinity (D1 and D2, respectively) was noticeable for certain scores used (Expression, Causal inference), but not for the others (Mutated submodules, Heterogeneous graph walk, Matrix Factorization). This was evident in both scenarios - for all genes (Supplementary 1, Figure, A) and for genes that meet novelty, small molecule druggability and protein class filters as per the novel kinase (causal) setting (Supplementary 1, Figure, B). Next, we executed a correlation analysis between the node degree and the scores, excluding the top 100 scored genes for each score independently as described in https://www.nature.com/articles/ng.3168. The findings indicate a positive correlation between node degree and scores even without the top 100 scored genes (Supplementary 1, Figure, C). Interestingly, this correlation diminishes

significantly when calculated solely for the top-ranked 100 genes for each score, as depicted in (Supplementary 1, Figure, D). This implies that the importance of node degree is substantial for genes with lower scores, while it becomes notably less influential for genes with top scores. This suggests that when gene evidence is scarce, additional connections wield a pronounced effect on the score. In contrast, as the number of connections saturates, scores exhibit reduced sensitivity to node degree. While we observe that not all scores exhibit a strong correlation with the individual scores, we acknowledge that the combined score significantly correlates with the node degree. This phenomenon aligns with a straightforward observation – genes with higher degrees in the network tend to have more independent pieces of evidence across various data types, leading to higher ranks. It can be seen that the D1 and D2 degrees of TNIK are quite low compared to the majority of the genes, but it is ranked quite high (Supplementary 1, Figure, E, F). Hence, the high score of TNIK could not be directly attributed to its extensive connections within the graph.

One of the unique features of PandaOmics target discovery platform is the validation platform used to estimate the performance of the models **(Extended Data Fig. 1F)**. We use two simple metrics to evaluate performance of the models. The first is a fold change enrichment of the top of the ranked list with the previously known targets on the logarithmic scale (ELFC). The second is the statistical significance of this enrichment based on the hypergeometric test p-value (HGPV). Using these two metrics as coordinates it is possible to map the performance of the models used to calculate ranking of the target hypotheses with respect to their relative performance. In order to demonstrate the ability of the models to predict truly novel target hypotheses a Time Machine approach was applied. This means that the models are trained using data published before a certain year and validated by their ability to predict those targets that came into focus of the pharmaceutical industry after this year.

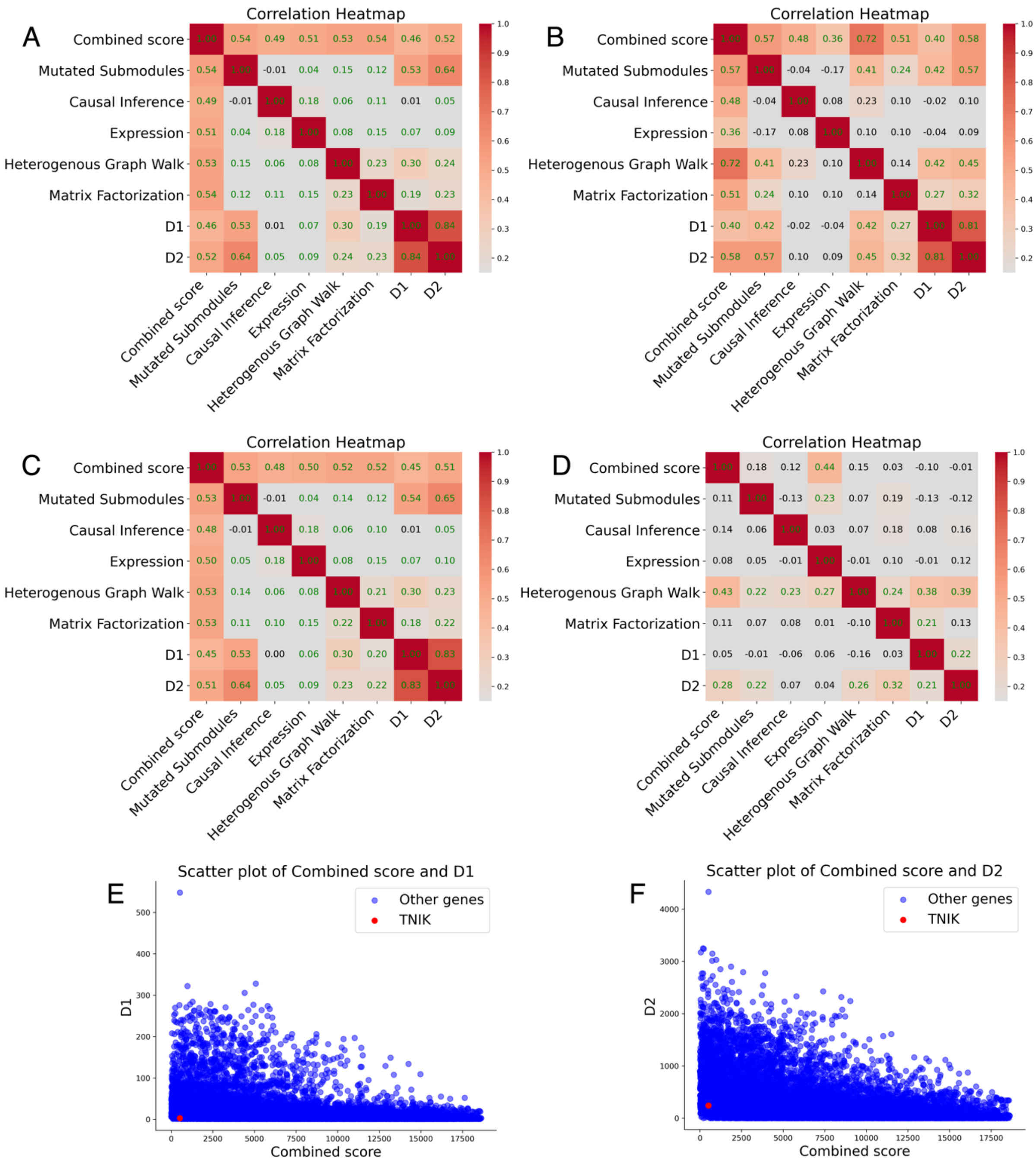


**Node degree control of the Target ID approach.** (**A,B**) Illustrating the correlation between node degree in the PPI graph and scores for all genes (**A**) and genes adhering to novelty, small molecule druggability, and protein class filters within the protein and receptor kinase setting (**B**). Correlation values surpassing the significance threshold of 0.05 are denoted in green. (**C,D**) Correlation between the scores and node degree for all genes without top 100 genes from each score (**C**) and for top 100 genes only (**D**). Each row represents a score for the top 100 genes either removed (**C**) or retained exclusively (**D**). Correlation values surpassing the significance threshold of 0.05 are denoted in green. (**E,F**) TNIK degree and rank for all genes based on combined score.

**Supplementary information 2: TNIK Indication prioritization analysis**

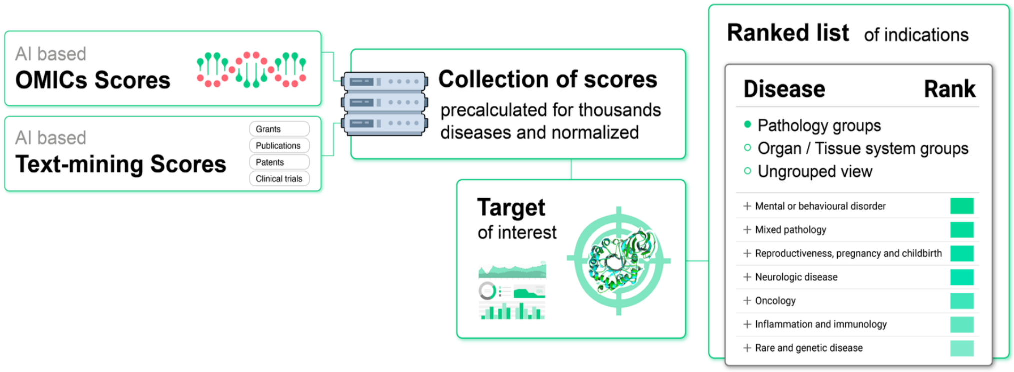


**Indication prioritization analysis pipeline using PandaOmics**. Indication Prioritization offers precalculated scores for over 7,000 indications, derived from text, financial, and omics data. All scores are considered when calculating the disease rank. Disease groups and indications are prioritized in ascending order based on their overall

| Disease | Overall rank | Rank in class |
| --- | --- | --- |
| Type 2 diabetes mellitus | 2 | 1 |
| Alzheimer's disease | 3 | 1 |
| Parkinson's disease | 18 | 2 |
| Rheumatoid arthritis | 33 | 2 |
| Amyotrophic lateral sclerosis | 40 | 3 |
| Chronic obstructive pulmonary disease | 45 | 4 |
| Idiopathic pulmonary fibrosis | 85 | 3 |
| Obesity | 98 | 5 |
| Chronic kidney disease | 100 | 3 |
| Cirrhosis of liver | 118 | 5 |
| Osteoporosis | 127 | 8 |
| Osteoarthritis | 131 | 20 |
| Pulmonary arterial hypertension | 145 | 8 |
| Primary myelofibrosis | 270 | 20 |

**Table**: TNIK Indication Prioritization for Aging-related diseases. Overall rank corresponds to ungrouped predictions. Rank in class is the rank within a corresponding disease area, *e.g.,* Neurologic or Fibrotic diseases.

**Supplementary information 3: Properties of INS018_025 and INS018_055. Similarity of INS018_055 to known TNIK inhibitors**

**Figure S3 Inhibitory effect or INS018_025 and INS018_055 on TNIK**

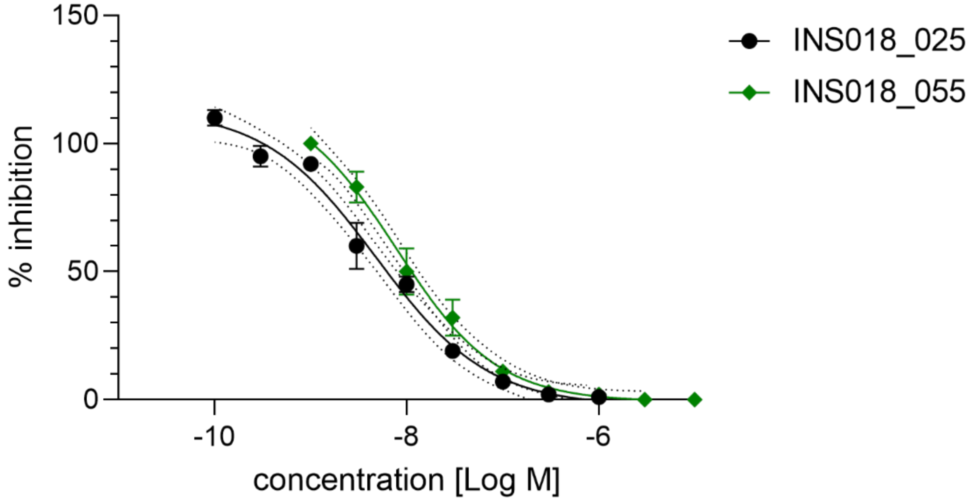


**Table S3-1 Property comparison between INS018_025 and INS018_055**

| **Property** | | **INS018_025** | **INS018_055** |
|---|---|---|---|
| **TNIK**, $IC_{50}$ (nM) | | 4.8 | 7.8 |
| **KS**, pH 7.4 (μM) | | <1.56 | 174 |
| **logP** | | 4.32 | 2.9 |
| **MMS** **$CL_{int}$** **(mic)** (μL/min/mg) | HLM | >120 | 25.2 |
| | MLM | >120 | 61.2 |
| **CYP**, $IC_{50}$ (μM) | | 1A2/2D6 ~ 2<br>2C9/2C19/3A4-M <0.5 | 1A2/2C9/2D6/<br>2C19/3A4-M > 30 |

**Table S3-2. Tanimoto similarity scores between INS018_055 and known TNIK inhibitors.**

| Molecule Name | SMILES | Tanimoto Similarity Score |
|---|---|---|
| Compound 21k[1] | O=C1c2ccc(-c3cnc4[nH]ccc4c3)cc2OCCN1Cc1ccc(F)cc1 | 0.15 |
| NCB-0846[2] | OC1CCC(Oc2cccc3cnc(Nc4ccc5nc[nH]c5c4)nc23)CC1 | 0.05 |
| Compound 16 [3] | CN1CCN(Cc2ccc(-c3cnc4[nH]cc(-c5cc(C#N)cc(NS(C)(=O)=O)c5)c4c3)cc2)CC1 | 0.08 |
| Compound 8 [4] | COc1ccc(C#N)cc1-c1ccnc(Nc2ccc(N3CCOCC3)cc2)c1 | 0.15 |
| PF-794 [5] | CC(C)NC(=O)c1ccc(-c2cnc(N)c(-c3ccc(C#N)cc3)c2)cc1 | 0.07 |
| ON108600 [6] | O=C1Nc2cc(S(=O)(=O)Cc3c(Cl)cccc3Cl)ccc2SC1=Cc1ccc(O)c([N+](=O)[O-])c1 | 0.06 |
| Compound 3 [7] | CN1CCN(c2ccc(NC(=O)c3ccc(-c4cc(Cl)ccc4Cl)o3)cc2)CC1 | 0.36 |

# Supplementary information 4: Synthesis and Characterization of INS018_055 and stability of INS018_055 acetate salt

## 1. Synthesis and Characterization of INS018_055

## I. Synthetic route of INS018_055

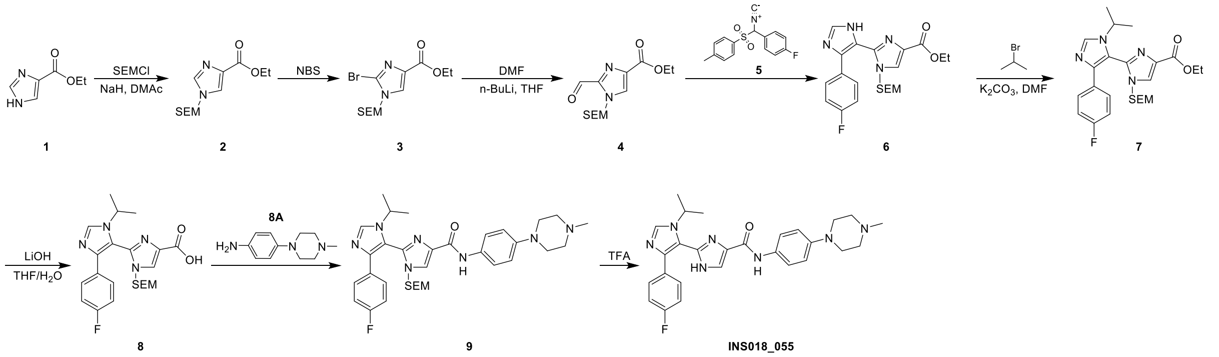


**Scheme 1** Synthetic route of INS018_055

## II. Synthesis of INS018_055

### Procedure for preparation of compound 2

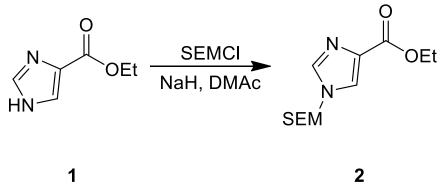


To a suspension of NaH (85.62 g, 2.14 mol, 60% purity, 1.5 eq) in THF (2 L) was added Compound **1** (200 g, 1.43 mol, 1 eq) in portions at 0 °C, it was stirred at 20 °C for 0.5 h, SEM-Cl (356.90 g, 2.14 mol, 378.88 mL, 1.5 eq) was added dropwise at 0 °C, it was stirred at 25 °C for 16 hrs. TLC (PE:EA 1:1) showed Compound 1 (Rf=0.1) was consumed completely and a new spot(Rf=0.3) was detected. LCMS showed Compound 1 was consumed and desired mass was detected. It was poured into the saturated $NH_4Cl$ (2L) aqueous, EA (2L) was added, the organic layer was washed with brine (1L × 3), dried over $Na_2SO_4$, filtered and concentrated in vacuum. The crude was purified by column chromatography ($SiO_2$, PE:EA 10:1~1:1). Compound **2** (210 g, 776.63 mmol, 54.42% yield) was obtained as a yellow oil.

### Procedure for preparation of compound 3

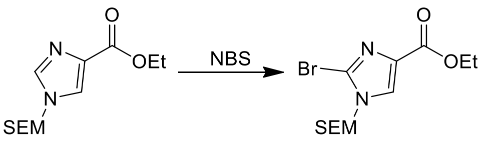

**2** **3**

To a solution of ethyl Compound **2** (150 g, 554.74 mmol, 1 eq) in $CHCl_3$ (1.5 L) was added NBS (108.60 g, 610.21 mmol, 1.1 eq) and AIBN (10.93 g, 66.57 mmol, 0.12 eq), it was stirred at 65 °C for 4 hrs. TLC (PE:EA 3:1) showed Compound 2 (Rf=0.1) was consumed and a new peak (Rf=0.4) was detected. It was washed with the saturated $NaHSO_3$ aqueous (1L) and brine (1L), the organic layer was dried over $Na_2SO_4$ and concentrated in vacuum. The crude was purified by column chromatography ($SiO_2$, PE:EA 100:1~3:1, Compound **3** (138 g, 395.08 mmol, 71.22% yield) was obtained as a yellow solid.

## Procedure for preparation of compound 4

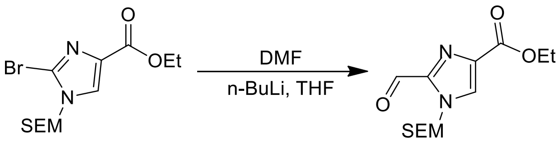

**3** **4**

To a solution of Compound 3 (28 g, 80.16 mmol, 1 *eq*) in THF (300 mL) was added *i*-PrMgCl (2 M, 120.24 mL, 3 *eq*) at -40°C. The mixture was stirred at -40°C for 10 min. To the mixture was added DMF (35.16 g, 480.97 mmol, 37.01 mL, 6 *eq*) at -70 °C. The mixture was stirred at 20 °C for 1 hour. LCMS was showed desired mass was detected. The mixture was poured to 1N HCl (500mL). The mixture was extracted with EA (300 mL × 2). The combined organic layers were dried over $Na_2SO_4$, filtered and concentrated under reduced pressure to give a residue. The residue was purified by column chromatography on silica gel (PE:EA=10:1-3:1). Compound **4** (15 g, 50.27 mmol, 62.71% yield) was obtained as yellow oil, which was checked with HNMR.

**LCMS**: (M+H) = 299.2

**HNMR**: (400 MHz, CHLOROFORM-d) δ = 8.03 - 7.95 (m, 1H), 5.80 (s, 2H), 4.47 - 4.41 (m, 2H), 3.63 - 3.57 (m, 2H), 1.46 - 1.40 (m, 3H), 0.99 - 0.93 (m, 2H), 0.00 (s, 8H)

## Procedure for preparation of compound 6

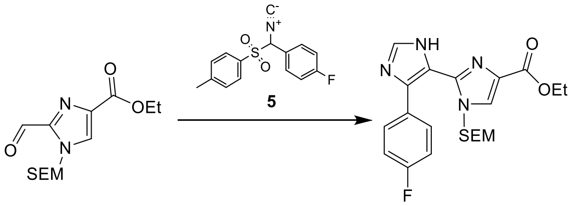

4 6

To a mixture of Compound **4** (12.5 g, 41.89 mmol, 1 *eq*) in THF (200 mL) was added $NH_3$•$H_2O$ (22.73 g, 162.11 mmol, 24.97 mL, 25% purity, 3.87 *eq*). The mixture was stirred at 20 °C for 4 hours. To the mixture was added Compound **5** (14.54 g, 50.27 mmol, 1.2 *eq*) and DIEA (16.24 g, 125.67 mmol, 21.89 mL, 3 *eq*) at 20 °C. The mixture was stirred at 20 °C for 2 hrs. TLC (PE:EA=0:1) showed Compound **4** was consumed and a new spot (Rf=0.3) was detected. The residue was poured into water (150 mL) and extracted with ethyl acetate (150 mL × 3). The combined organic phase was washed with brine (200 mL × 1), dried with anhydrous $Na_2SO_4$, filtered and concentrated in vacuum. The crude product was purified by column chromatography on silica gel (PE:EA=1:1-0:1). Compound **6** (10 g, 22.51 mmol, 53.74% yield, 96.914% purity) was obtained as yellow solid, which was checked with LCMS.

**LCMS**: (M+H) = 431.2

**Procedure for preparation of compound 7**

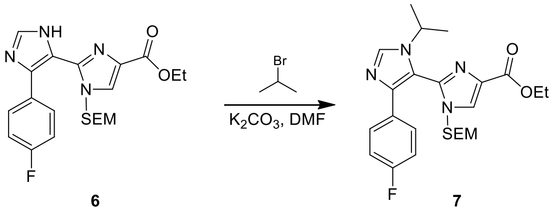

6 7

To a mixture of Compound **6** (10 g, 23.23 mmol, 1 *eq*) in DMF (60 mL) was added $K_2CO_3$ (9.63 g, 69.68 mmol, 3 *eq*) 2-bromopropane (8.57 g, 69.68 mmol, 6.54 mL, 3 *eq*). The mixture was stirred at 60 °C for 6 hours. LCMS showed Compound **6** was consumed and the desired mass was detected. The residue was poured into ice-water (500 mL) and extracted with ethyl acetate (100 mL × 3). The combined organic phase was washed with brine (200 mL × 1), dried with anhydrous $Na_2SO_4$, filtered and concentrated in vacuum. The residue was purified by silica gel chromatography (PE:EA=1:1-1:5). Compound **7** (7.6 g, crude) was obtained as yellow solid, which was checked with HNMR.

**LCMS**: (M+H) = 473.3

**HNMR**: (400 MHz, DMSO-d6) δ = 8.30 (s, 1H), 8.16 - 8.09 (m, 1H), 7.27 (br dd, J = 5.6, 8.8 Hz, 2H), 7.17 - 7.06 (m, 2H), 5.03 - 4.83 (m, 2H), 4.29 (br d, J = 6.4 Hz, 2H), 4.06 (br s, 1H), 3.28 - 3.18 (m, 2H), 1.39 (br s, 6H), 1.32 (t, J = 7.2 Hz, 3H), 0.61 - 0.50 (m, 2H), -0.07 - -0.14 (m, 9H)

**Procedure for preparation of compound 8**

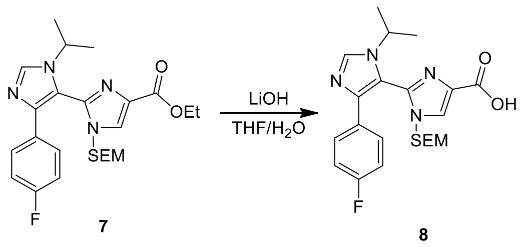

To a mixture of Compound **7** (7.6 g, 16.08 mmol, 1 *eq*) in THF (80 mL) and $H_2O$ (16 mL) was added LiOH (2.70 g, 112.56 mmol, 7 *eq*). The mixture was stirred at 20 °C for 3 hours. LCMS showed reactant was consumed, and desired mass was detected. The residue was acidified with the aqueous HCl (1 M) and the resulting mixture was extracted with ethyl acetate (100 mL × 3). The combined organic phase was washed with brine (150 mL), dried with anhydrous $Na_2SO_4$, filtered and concentrated in vacuum. The crude compound was used into the next step without further purification. Compound **8** (6.1 g, crude) was obtained as yellow solid, which was checked with HNMR.

**LCMS**: (M+H) = 445.3

**HNMR**: (400 MHz, DMSO-d6) δ = 8.98 - 8.76 (m, 1H), 8.27 (s, 1H), 7.42 - 7.16 (m, 4H), 5.00 (br s, 2H), 4.16 (td, J = 6.8, 13.2 Hz, 1H), 3.21 (br s, 2H), 1.43 (br d, J = 6.8 Hz, 6H), 0.53 (br d, J = 3.2 Hz, 2H), -0.04 - -0.15 (m, 9H)

**Procedure for preparation of compound 9**

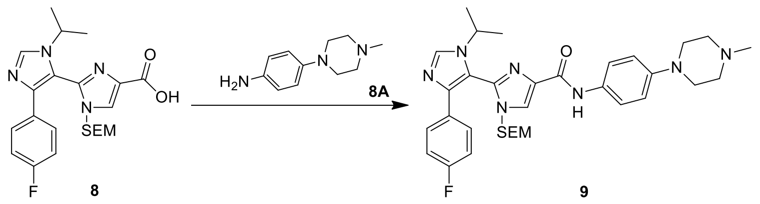

To a mixture of Compound **8** (6.5 g, 14.62 mmol, 1 *eq*) and Compound **8A** (4.19 g, 21.93 mmol, 1.5 *eq*) in DMF (50 mL) was added HATU (8.34 g, 21.93 mmol, 1.5 *eq*) and DIEA (5.67 g, 43.86 mmol, 7.64 mL, 3 *eq*). The mixture was stirred at 20 °C for 3 hours. LCMS showed Compound **8** was consumed and desired mass was detected. The residue was poured into water (500 mL) and extracted with ethyl acetate (150 mL × 3). The combined organic phase was washed with brine (200 mL), dried with anhydrous $Na_2SO_4$, filtered and concentrated in vacuum. The crude compound was used into the next step without further purification. Compound **9** (9 g, crude) was obtained as brown oil.

**LCMS**: (M+H) = 618.4

**Procedure for preparation of INS018_055**

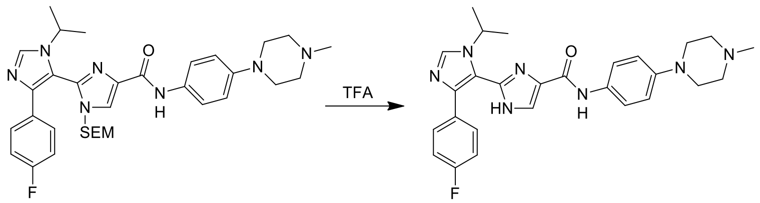

**9** **INS018_055**

To a mixture of Compound **9** (9 g, 14.57 mmol, 1 *eq*) in $CH_2Cl_2$ (2 mL) was added TFA (30.80 g, 270.12 mmol, 20.00 mL, 18.54 *eq*). The mixture was stirred at 20 °C for 5 hours. LCMS showed Compound **9** was consumed and the desired mass was detected. The mixture was basified with aqueous $NaHCO_3$) till pH = 8 and the resulting mixture was extracted with ethyl acetate (200 mL × 4). The combined organic phase was washed with brine (100 mL), dried with anhydrous $Na_2SO_4$, filtered and concentrated in vacuum. INS018_055 (4.16 g, 58.5% yield, 94.71% purity) was obtained as crude product. The crude product was dissolved in EtOH (5V) and then water (5V) was added drop-wise at 15-25°C. Resulting suspension was stirred at 15-25°C for 3hrs, and then filtered. The cake was washed with water (3V) and then dried at 45°C to give final INS018_055 (2.91g, 40.97% yield, 97.87% purity).

See **HRMS** and **NMR** data in the next part.

## III. Characterization of INS018_055

### HRMS

High resolution mass spectrometric analysis was performed on Agilent G6224A TOF LC/MS. Electrospray ionization was performed in positive ion mode.

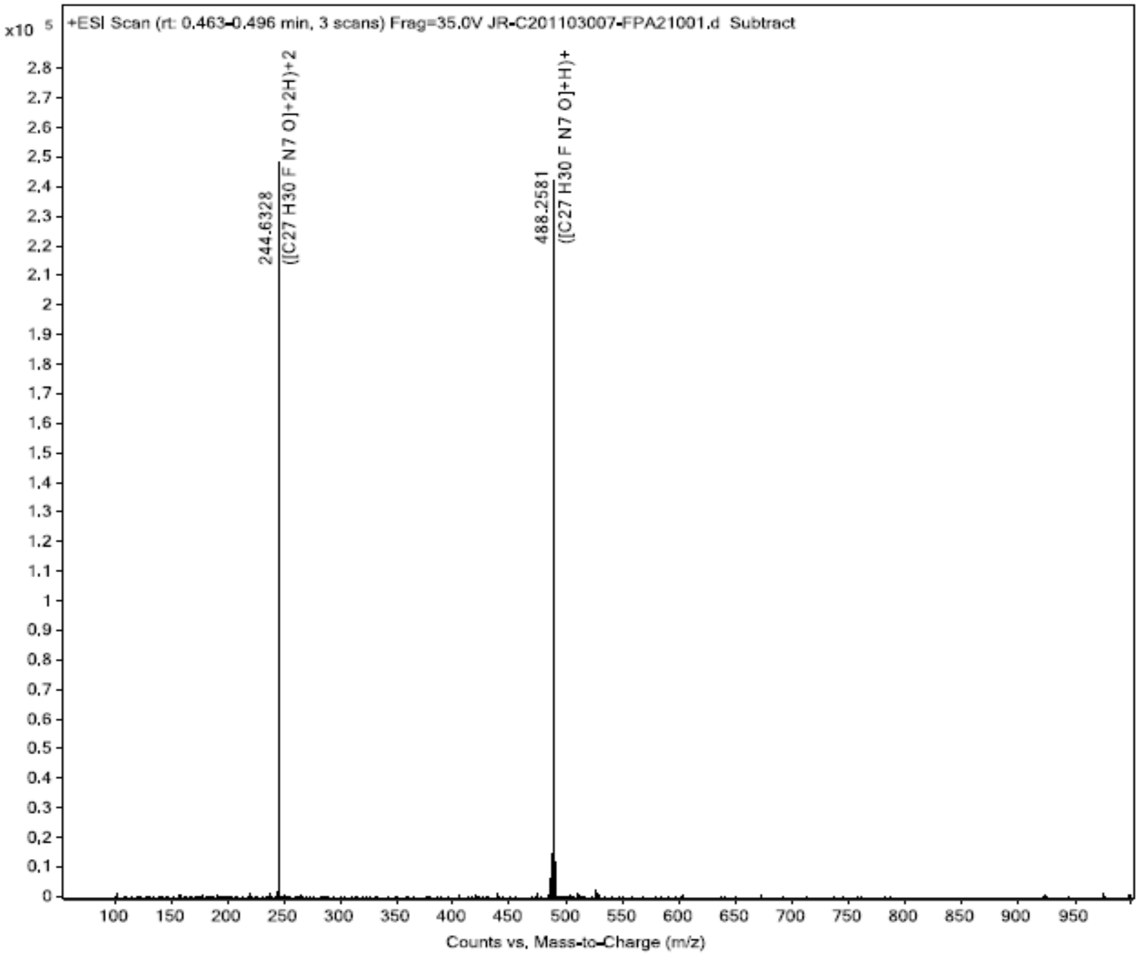


High Resolution Mass Spectrum for INS018_055

The accurate mass of the protonated molecule (m/z 488.2581) is consistent with a formula of $C_{27}H_{31}FN_7O^+$ (2.43 ppm difference compared to theoretical value m/z 488.2569). Pseudo molecular ion $[M+2H]^{2+}$ was observed at m/z 244.6328 corresponding to the $C_{27}H_{32}FN_7O^{2+}$ (2.91 ppm difference compared to the theoretical value m/z 244.6321). The data is consistent with the molecular formula proposed for the drug substance.

**NMR**

A Bruker AVANCE III 400 MHz NMR spectrometer equipped with a Bruker 5 mm PABBO BB-400 MHz Zgradient high resolution probe and supported by TOPSPIN 3.2 software, was used to collect 1-dimensional (1D) proton, fluorine and carbon data at 300K.

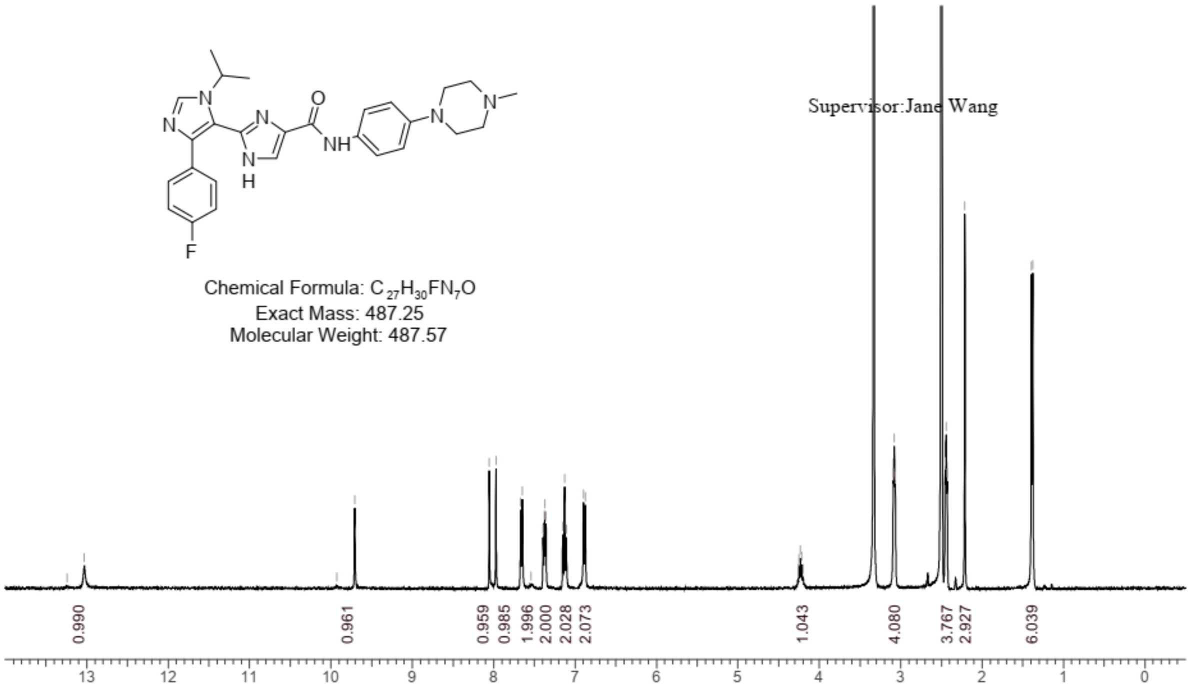


$^1$H NMR Spectrum for INS018_055

**$^1$H NMR** (400 MHz, DMSO-*d*6) 13.32 - 12.89 (m, 1H), 9.96 - 9.66 (m, 1H), 8.09 - 8.01 (m, 1H), 7.97 (s, 1H), 7.70 - 7.49 (m, 2H), 7.41 - 7.32 (m, 2H), 7.18 - 7.07 (m, 2H), 6.96 - 6.80 (m, 2H), 4.29 - 4.14 (m, 1H), 3.15 - 3.02 (m, 4H), 2.48 - 2.39 (m, 4H), 2.21 (s, 3H), 1.39 (d, *J* = 6.8 Hz, 6H)

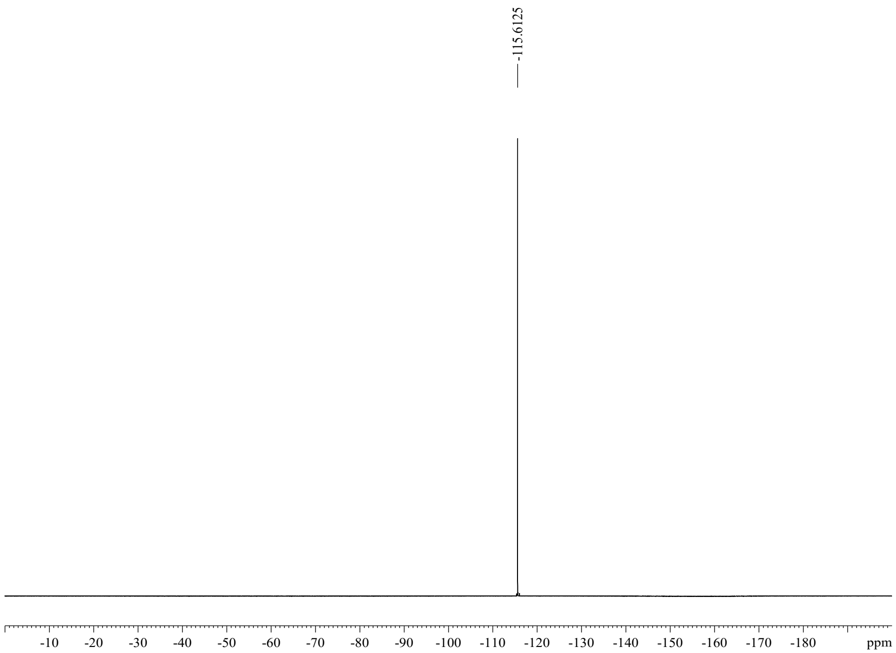


**Figure 4** $^{19}$F NMR Spectrum for INS018_055

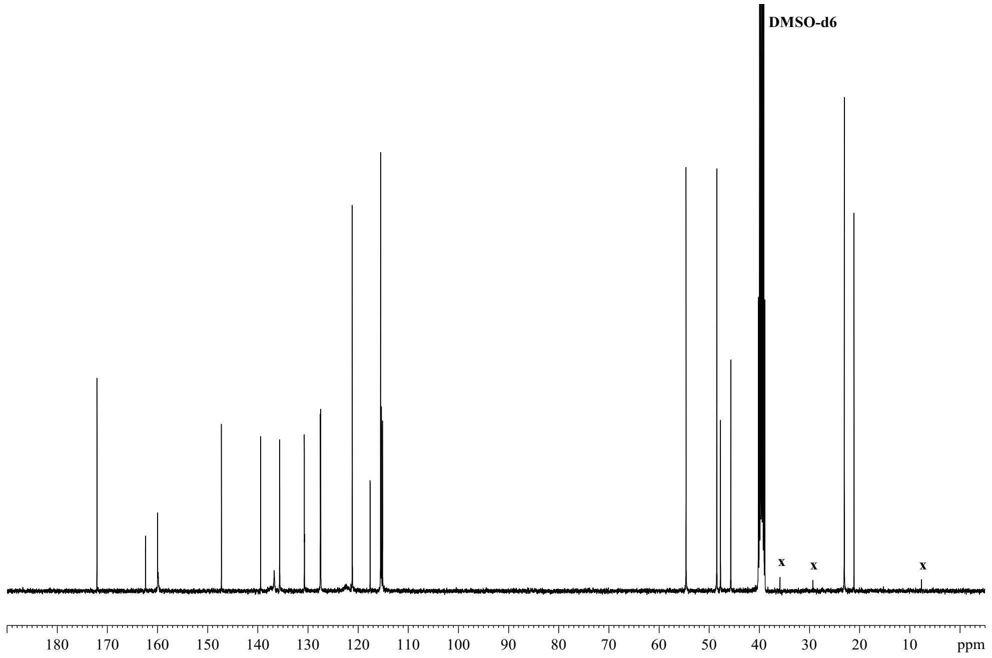


$^{13}$C NMR Spectrum for INS018_055

**$^{13}$C NMR** (100 MHz, DMSO-*d6*)δ172.05, 161.13 (d, *J* = 243.6 Hz)$^{*}$, 159.81, 147.25, 139.43, 137.26, 136.71, 135.63, 130.72, 130.70 (d, *J* = 3.8 Hz)$^{*}$, 127.49 (d, *J* = 8.0 Hz)$^{*}$, 122.34, 121.15, 117.61, 115.51, 115.23 (d, *J* = 21.4 Hz)$^{*}$, 54.60, 48.47, 47.73, 45.68, 23.04, 21.10

$^{*}$: The carbon is coupled with fluorine and appeared as doublet in the $^{13}$C NMR spectrum.

## 2. stability of INS018_055 acetate salt

| **Stability Test Condition** | **Purity, HPLC (% area)** | | | | | **Note** |
|---|---|---|---|---|---|---|
| | Initial | 01 Month | 03 Month | 06 Month | 09 Month | |

| | | | | | | |
|---|---|---|---|---|---|---|
| 25 ºC± 2 ºC/60%RH±5%RH, Normal | 99.3% | 99.3% | 99.2% | 99.3% | 99.3% | A typical API sample. No obvious impurity increase during stability test |
| 40 ºC± 2 ºC/75%RH±5%RH, Normal | 99.3% | 99.3% | 99.2% | 99.3% | 99.3% | |

**Supplementary information 5: Selectivity of INS018_055**

Table S-5 : selectivity Profile of INS018_055

| Kinase (h)* | ATP (µM)** | Activity (% control) at INS018_055=10 µM | $IC_{50}$ (nM) | Relation to fibrosis |
|---|---|---|---|---|
| (Wild type) | | | | |
| **TNIK (h)** | **70** | **4** | **31** | |
| Yes (h) | 45 | 0 | 48 | |
| ALK4 (h) | 10 | -1 | 59 | Fibrosis driver [8] |
| MINK (h) | 200 | -1 | 115 | |
| TGFBR1 (h) | 10 | -5 | 168 | Fibrosis driver [8] |
| Fms (h) | 200 | 1 | 170 | Fibrosis driver [9] |
| DDR1 (h) | 15 | -5 | 184 | Fibrosis driver [10] |
| MAP4K4 (h) | 200 | | 277 | |
| PKD3 (h) | 45 | 5 | 300 | Increased and activated in IPF [11] |
| PKD2 (h) | 155 | 9 | 368 | Increased and activated in IPF [11] |
| ACK1 (h) | 70 | 6 | 549 | |
| Lck (h) | 90 | 13 | 771 | Fibrosis driver [12] |
| PKCµ (h) | 45 | 6 | 771 | |
| KDR (h) | 90 | 14 | 1100 | |
| MAP4K5 (h) | 45 | 11 | 1228 | |
| ALK1 (h) | 10 | 4 | 1355 | |
| Fyn (h) | 70 | 7 | 1576 | Fibrosis driver [13] |
| Lyn (h) | 70 | 5 | 1756 | Fibrosis driver [14] |
| EGFR (h) | 10 | 13 | 2391 | Highly expressed in lung fibrosis [15] |
| Fgr (h) | 45 | 20 | 2520 | Fibrosis driver [16] |
| Flt1 (h) | 200 | 18 | 2526 | |
| Flt4 (h) | 200 | 23 | 2589 | |

| | | | | |
|---|---|---|---|---|
| JNK3 (h) | 10 | 18 | 2633 | |
| ErbB2 (h) | 10 | 17 | 4202 | Fibrosis driver [17] |
| Abl (h) | 45 | 36 | 4981 | |
| Aurora-C (h) | 15 | 49 | 5092 | |
| Aurora-B (h) | 10 | 40 | >10,000 | |
| ErbB4 (h) | 15 | 47 | >10,000 | |
| (Mutant) | | | | |
| Abl(H396P) (h) | 15 | 12 | 1668 | |
| Abl(M351T) (h) | 15 | 10 | 629 | |
| Abl(Q252H) (h) | 15 | 1 | 251 | |
| Abl(Y253F) (h) | 15 | 14 | 1579 | |
| cKit(D816H) (h) | 15 | 5 | 68 | |
| cKit(V654A) (h) | 200 | 2 | 209 | |
| cKit(V560G) (h) | 200 | 8 | 1130 | |
| EGFR(L858R) (h) | 200 | 7 | 96 | |
| EGFR(L861Q) (h) | 15 | 3 | 113 | |
| EGFR(T790M) (h) | 10 | 17 | 1406 | |
| EGFR(T790M, L858R) (h) | 45 | 7 | 208 | |
| PDGFRα(D842V) (h) | 15 | -1 | 62 | |
| PDGFRα(V561D) (h) | 200 | 4 | 472 | |
| Src(1-530) (h) | 45 | 16 | 2226 | |
| Reported targets of NCB-0846 | | | | |
| JAK3 | 10 | 109 | | |
| PDGFRa | 120 | 65 | | |
| TRKA, | 120 | 99 | | |
| CDK2/CycA, | 45 | 101 | | |
| FLT3 | 200 | 111 | | |
| PYK2 | 90 | 97 | | |

*(h): human; ** Km values were used as ATP concentrations.

**Supplementary information 6:Effect of INS018_055 treatment on primary human lung fibroblasts and primary human bronchial epithelial cells**

The inhibitory effect of INS018_055 on fibroblast to mesenchymal transition (FMT) by measurement of α-SMA (blue) and evaluation of cytotoxicity (yellow) was performed in **(A)** cells from IPF donors (IPF05, IPF06 and IPF08) and (**B)** cells from healthy donors (FB2382, 03HF067101 and FB218). The inhibitory effect of INS018_055 on EMT by measurement of fibronectin (FN1) (blue) and evaluation of cytotoxicity (yellow) was performed in **(C)** cells from IPF donors (IPF05, IPF06 and IPF08) and (**D)** cells from healthy donors (Br285, Br311 and 410955). Nintedanib was used as a reference compound in assays on cells from IPF donors. SB525334 was used as a reference compound in assays on cells from healthy donors. % remaining cells is a nuclear count, measuring the percentage of cells without nuclear loss. Biological duplicates were performed on two different cell plates on one occasion.

A

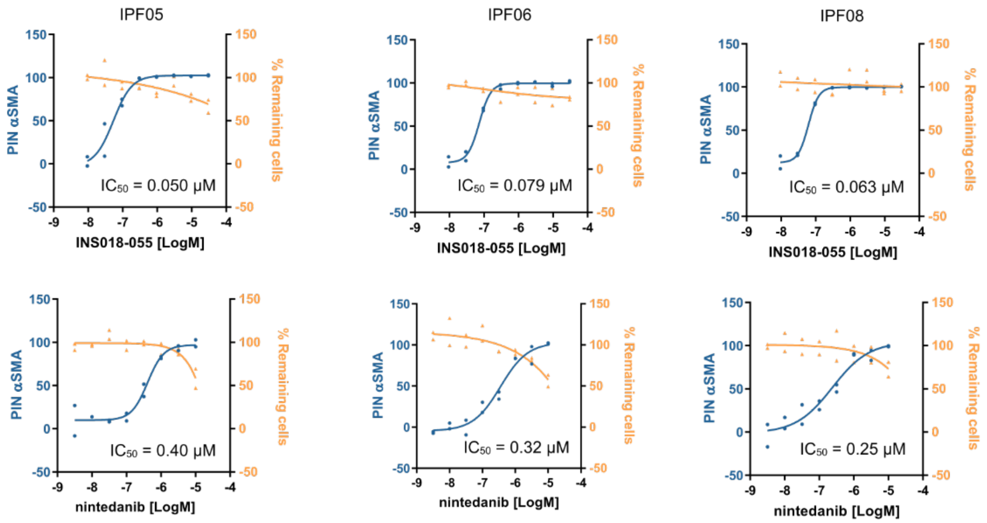
IPF05
IPF06
IPF08
PIN αSMA
% Remaining cells
IC50 = 0.050 μM
IC50 = 0.079 μM
IC50 = 0.063 μM
INS018-055 [LogM]
IC50 = 0.40 μM
IC50 = 0.32 μM
IC50 = 0.25 μM
nintedanib [LogM]

B

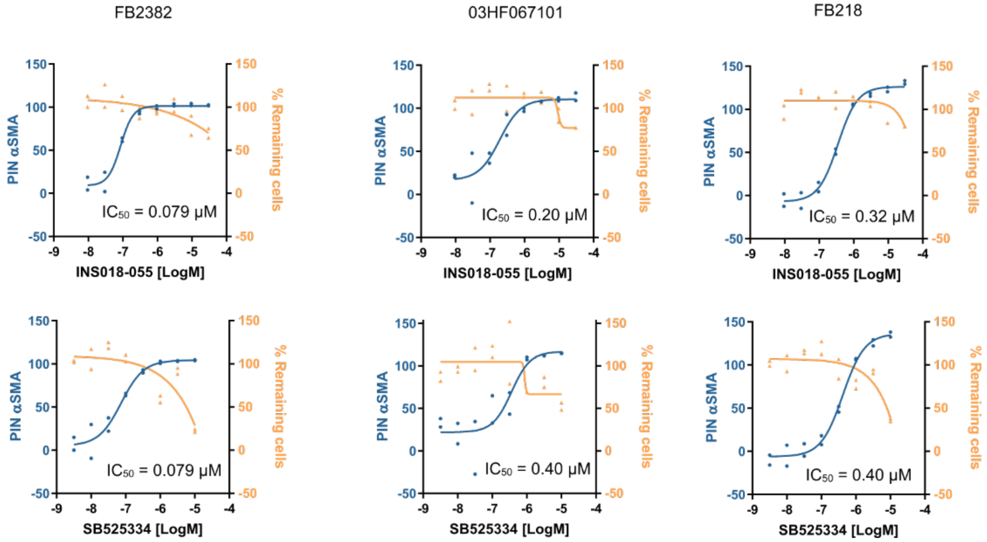
FB2382
03HF067101
FB218
PIN αSMA
% Remaining cells
IC50 = 0.079 μM
IC50 = 0.20 μM
IC50 = 0.32 μM
INS018-055 [LogM]
IC50 = 0.079 μM
IC50 = 0.40 μM
IC50 = 0.40 μM
SB525334 [LogM]

C

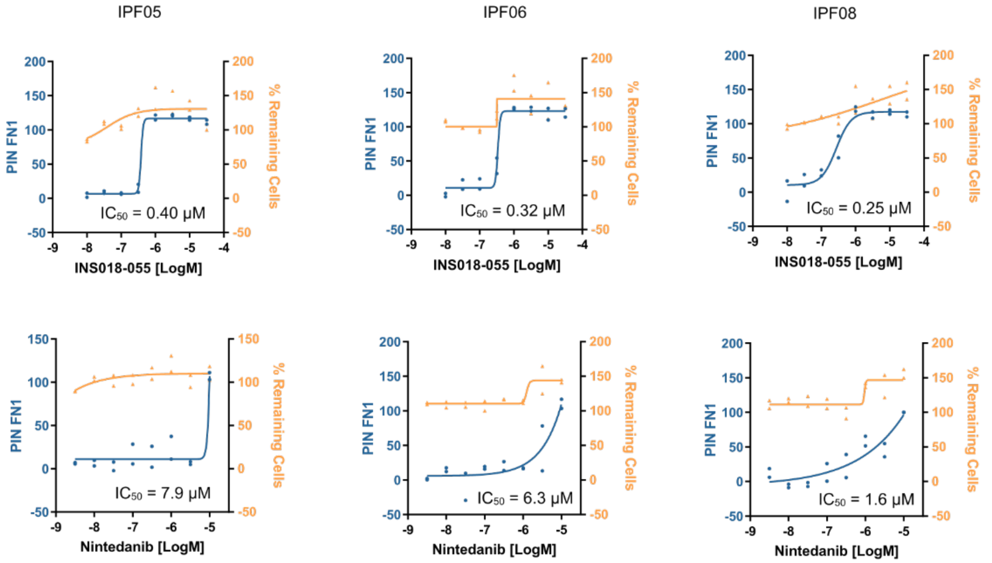

IPF05
IPF06
IPF08
PIN FN1
% Remaining Cells
IC50 = 0.40 μM
IC50 = 0.32 μM
IC50 = 0.25 μM
INS018-055 [LogM]
IC50 = 7.9 μM
IC50 = 6.3 μM
IC50 = 1.6 μM
Nintedanib [LogM]


D

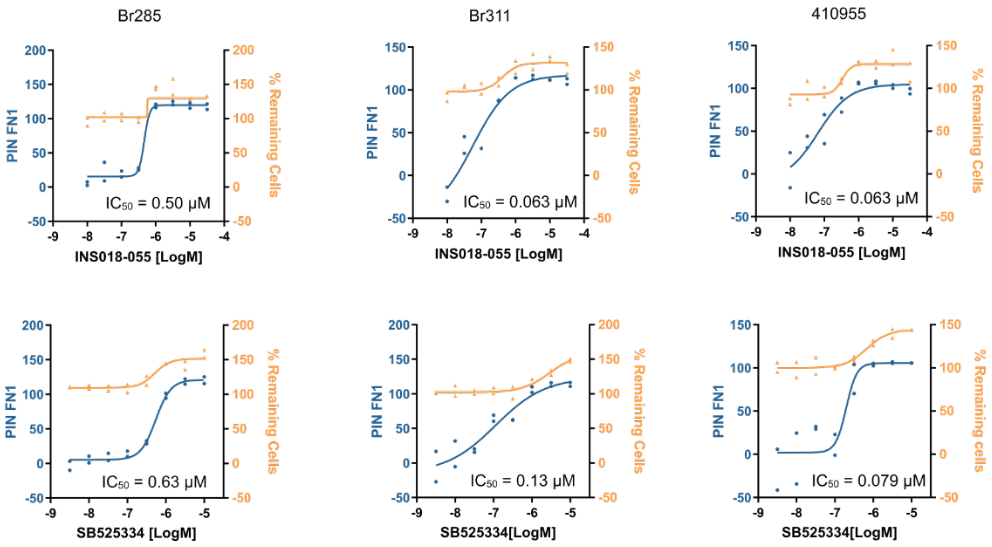

Br285
Br311
410955
PIN FN1
% Remaining Cells
IC50 = 0.50 μM
IC50 = 0.063 μM
IC50 = 0.063 μM
INS018-055 [LogM]
IC50 = 0.63 μM
IC50 = 0.13 μM
IC50 = 0.079 μM
% Remaining cells
SB525334 [LogM]
SB525334[LogM]
SB525334[LogM]

**Supplementary information 7: Inhalation Study Data**

Lung and plasma concentration of INS018_055 in inhalation study.

| Test article groups (mg/mL) | Actual delivered dose (mg/kg) | Concentration of INS018-055 | | |
|---|---|---|---|---|
| | | Plasma(ng/mL) | Left lung tissue(ng/g) | Right lung tissue(ng/g) |
| 0.1 | 0.040 | 0.33±0.12 | 16.18±3.73 | 17.37±3.81 |
| 0.3 | 0.136 | 1.13±0.37 | 77.55±23.52 | 78.35±10.05 |
| 1.0 | 0.485 | 4.41±0.80 | 301.47±66.25 | 340.47±53.82 |
| 6.0 | 2.575 | 30.40±3.63 | 1468.07±278.61 | 1490.45±328.68 |

Blood samples and lung tissue homogenates were collected from the animals immediately after Day 28 administration to detect the concentration in the sample. The concentration of INS018_055 in animal plasma and lung tissue was measured on Day 28.

**Supplementary information 8: Complete Phase 0 Clinical Trial Information**

A phase 0 clinical trial, also being the first-in-human clinical trial of INS018_055, was conducted in Australia (ACTRN12621001541897). After single IV micro-doses (100 µg/individual) administration, PK of plasma INS018_055 was evaluated in healthy adult subjects.
A total of 8 healthy adult subjects were enrolled. On day -1, baseline data was taken and safety were evaluated. On day 1, A single dose of 100 µg INS018_055 was given to each subject through IV injection. Blood was collected pre-dose and 5, 15, 30 minutes, then 1, 2, 4, 8 and 24 hours post-dose for PK evaluation. After dosing, individuals were monitored for safety until discharged in Day 2.

**Number of participants:** eight
**Determination of Sample size:**
The number of participants was selected to allow for evaluation of safety/tolerability and PK in this study and is consistent with standards of practice for Phase 0 studies.

This trial was conducted in accordance with the ethical principles of Good Clinical Practice, according to the International Council for Harmonisation (ICH) Harmonised Guideline E6(R2) Integrated Addendum to ICH E6(R1): Guideline for Good Clinical Practice ICH E6(R2), annotated with comments by the Australian Therapeutic Goods Administration (TGA; 2018).

**Objectives:**
Primary:
The primary objective of the study was to determine the PK of plasma INS018_055 after a single IV microdose administered to healthy participants.

**Endpoints:**
Primary:
The primary endpoints of this study were:
•Volume of distribution of INS018_055 in healthy participants.
•Elimination half-life (t1/2) of INS018_055.
•Clearance of INS018_055 in healthy participants.

**Inclusion Criteria**
Participants who met all of the following criteria at Screening were eligible to participate in the study:
1. Healthy female or male aged ≥ 18 and ≤ 55 years at Screening. Healthy was defined as no clinically relevant abnormalities identified by a

detailed medical history, full physical examination, including blood pressure and pulse rate measurement, 12-lead electrocardiogram (ECG), and clinical laboratory tests.

2. BMI of 17.50 to 30.50 kg/m2; and a total body weight > 50 kg at Screening and Day -1.

3. Sufficient venous access for the purposes of the study.

4. Non-smoker. The participant must not have used any tobacco products within 2 months prior to Screening.

5. Females were non-pregnant and non-lactating, and agreed to use an acceptable, highly effective double contraception from Screening until study completion, including the Follow-up period. Double contraception was defined as a condom AND one other form of the following:

a. Established hormonal contraception (with approved long-acting implantable hormones, injectable hormones). Oral contraceptive pills [OCPs] could not be used as a second form of contraception by female participants, due to the unknown potential for OCP reduced effectiveness when administered in combination with INS018_055.

b. A vaginal ring or an intrauterine device (IUD) (including a hormonal IUD).

c. Documented evidence of surgical sterilisation at least 6 months prior to Screening (eg, tubal occlusion, hysterectomy, bilateral salpingectomy, or bilateral oophorectomy for women or vasectomy for men [with appropriate post-vasectomy documentation of the absence of sperm in semen] provided the male partner was a sole partner).

Women not of childbearing potential were post-menopausal for ≥ 12 months. Post-menopausal status was confirmed through testing of follicle-stimulating hormone (FSH) levels ≥ 40 IU/L at Screening for amenorrhoeic female participants. Females who were abstinent from heterosexual intercourse were also eligible.

Periodic abstinence (eg, calendar, ovulation, symptothermal, post-ovulation methods) and withdrawal were not considered highly effective methods of birth control. Participant complete abstinence for the duration of the study and for 90 days after the last study treatment was acceptable.

Female participants who were in same-sex relationships were not required to use contraception.

Women of childbearing potential (WOCBP) had a negative pregnancy test at Screening and Day 1 and were willing to have additional pregnancy tests as required throughout the study.

Males were surgically sterile (> 30 days since vasectomy with no viable sperm), abstinent, or if engaged in sexual relations with a WOCBP, the participant and his partner were surgically sterile (eg, tubal occlusion, hysterectomy, bilateral salpingectomy, bilateral oophorectomy) or using an acceptable, highly effective contraceptive method from Screening until study completion, including the Follow-up period. Acceptable methods of contraception included the use of condoms and the use of an effective contraceptive for the female partner that included: OCPs, long-acting implantable hormones, injectable hormones, a vaginal ring, or an IUD. Participants with same-sex partners (abstinence from penile-vaginal intercourse) were eligible when this was their preferred and usual lifestyle.

Males could not donate sperm for at least 90 days after the last study treatment.

6. Willing and able to attend the trial visits and complete study assessments.

7. Willing to consume standard meals provided.

8. Able to read and understand study documents and follow Investigator and study personnel instructions during visits.

9. Able to sign the HREC approved ICF.

**Exclusion Criteria**

Participants who met any of the following criteria at Screening were not eligible to participate in the study:

1. Positive toxicology screening panel (urine test including qualitative identification of barbiturates, tetrahydrocannabinol [THC], amphetamines, benzodiazepines, opiates, and cocaine), or with a history of substance abuse or dependency or history of recreational IV drug use over the last 5 years (by self-declaration).

2. Positive alcohol breath test at Screening or a history of regular alcohol consumption exceeding 14 drinks/week for women or 21 drinks/week for men (1 drink = 150 mL of wine or 360 mL of beer or 45 mL of hard liquor) within the 6 months prior to Screening.

3. Major surgery or significant trauma within 28 days (4 weeks) prior to Screening.

4. Blood pressure (BP) > 150 mmHg (systolic) or > 95 mmHg (diastolic) at Screening and Day -1, following at least 5 minutes of supine rest. If BP was > 140 mmHg (systolic) or > 90 mmHg (diastolic), the BP measurements was repeated 2 more times, at least 2 minutes apart, and the average of the 3 BP values was used to determine the participant's eligibility.

5. Heart rate < 45 beats per minute (bpm) or > 100 bpm at Screening and Day -1, following at least 5 minutes of supine rest. If heart rate was below 45 bpm or exceeds 100 bpm, the heart rate was repeated 2 more times, at least 2 minutes apart, and the average of the 3 heart rate values was used to determine the participant's eligibility.

6. 12-lead ECG demonstrating QTc > 450 msec for males or > 470 msec for females, or a QRS interval ≥ 120 msec at Screening and Day -1. If QTc exceeded 450 msec (males) or 470 msec (females), or QRS exceeded 120 msec, the ECG was repeated 2 more times, at least 2 minutes apart, and the average of the 3 QTc (or QRS) values was used to determine the participant's eligibility.

7. ANY of the following abnormalities in clinical laboratory tests at Screening, as assessed by the study-specific laboratory and confirmed by a single repeat, if deemed necessary: − Serum creatinine level above the upper limit of normal (ULN) or an estimated glomerular filtration rate value < 80 mL/min, based on the Cockcroft-Gault calculation, at Screening.

− Aspartate aminotransferase (AST) / serum glutamic oxaloacetic transaminase (SGOT) or alanine aminotransferase (ALT) / serum glutamic pyruvic transaminase (SGPT) > 1.5 × ULN.

− Fasting glucose > 5.4 mmol/L.

− Total bilirubin > 1.5 × ULN.

8. A white blood cell count < 4.0 × $10^9$/L. Participants with borderline clinical laboratory values outside the reference range could be included in the study if the Investigator deemed that the values were not clinically significant.

9. Absolute neutrophil count of < 2 × $10^9$/L.

10. Haematocrit below 0.4 for males and 0.35 for females.

11. Use of any IP or investigational medical device within 30 days prior to Screening, or 5 half-lives of the product (whichever was the longest) or participation in more than 4 investigational drug studies within 1 year prior to Screening.

12. Use of prescription or non-prescription drugs and dietary supplements within 7 days or 5 half-lives (whichever was longer) prior to INS018_055

administration, with the exception of paracetamol, which could be used at doses of ≤ 2 g/day, and contraceptives.

13. Blood donation (excluding plasma donations) of ≥ 500 mL or significant blood loss within 56 days prior to dosing.

14. History of sensitivity to heparin or heparin-induced thrombocytopenia.

15. Other severe acute or chronic medical or psychiatric condition including recent (within the past year) or active suicidal ideation or behaviour or laboratory abnormality, or any other abnormality that in the opinion of the Investigator could increase the risk associated with study participation or IP administration or could interfere with the interpretation of study results and make the participant inappropriate for entry into this study.

16. CRU staff members directly involved in the conduct of the study and their family members, CRU members otherwise supervised by the Investigator, or participants who were Sponsor employees including their family members directly involved in the conduct of the study.

17. Vaccination with live virus, attenuated live virus, or any live viral components within the 6 weeks prior to the first dose of study drug or was to receive these vaccines at any time during treatment or within 8 weeks following the end of study visit. Scheduled/intended to have a COVID-19 vaccine during the study (ie, from Screening through to Day 8).

18. History of any lymphoproliferative disorder (such as EBV related lymphoproliferative disorder, as reported in some participants on other immunosuppressive drugs), history of lymphoma, leukaemia, myeloproliferative disorders, multiple myeloma, or signs and symptoms suggestive of current lymphatic disease.

19. Clinically significant infection currently or within 6 months of first dose of study drug (those requiring hospitalisation or parenteral antimicrobial therapy or opportunistic infections), or a history of chronic or recurrent infectious disease.

20. Known infection with or test positive at Screening for HIV, hepatitis B or C viruses.

21. History of malignancy, except for non-melanoma skin cancer, excised more than 2 years ago, and cervical intraepithelial neoplasia that had been successfully cured more than 5 years prior to Screening.

22. Consumption of grapefruit or grapefruit juice or citrus fruits (ie, Seville oranges, pomelos, tangelos) within 7 days prior to the first dose of study medication until collection of the final pharmacokinetic blood sample.

23. History of severe allergic reactions (eg, anaphylaxis) or known sensitivity to any of the constituents of the test product.

24. Pregnant or lactating at Screening or planning to become pregnant (self or partner) at any time during the study, including the Follow-up period.

25. History of benign ethnic neutropenia.

1) Safety

No severe treatment emergent adverse events (TEAE) of CTCAE Grade 3 or higher was reported in any subject during the study, and no TEAE resulted in discontinuation of INS018_055 reported.

Mild TEAEs were reported, including 3 of 8 subjects and 4 events occurred in total.

One event (myoclonus) **(Table S8-1)** in 1 of 8 subjects was considered of mild severity and maybe a treatment-related TEAE. No AE was reported in haematology, clinical chemistry, or urinalysis, which showed no abnormalities of clinically significance. In evaluation of vital signs,

electrocardiogram monitoring, physical examinations and telemetry, no concerned consistent changes were reported. In summary, no risk was identified in this micro-doses (100 µg/individual, IV) administration.

2) PK

PK data is presented in **Table S8-2**.

The $T_{max}$ is at 0 hour hour dose as expected as after IV administration. Between 0.25 and 4 hours post dose, INS018_055 plasma concentration rapidly decreased and more gradually after 4 hours. The half-life of INS018_055 is 4.644 ± 1.936 hours. Mean $AUC_{(0-24)}$ was 2.576 h*ng/mL.

### Table S8-1: Summary of TEAE

| | **INS018_055 (N=8) n (%) M** |
|---|---|
| **Participants with at least one TEAE** | **3 (37.5%) 4** |
| | |
| **General disorders and administration site conditions** | **2 (25.0%) 3** |
| Administration site erythema | 1 (12.5%) 1 |
| Administration site irritation | 1 (12.5%) 1 |
| Administration site pruritus | 1 (12.5%) 1 |
| **Nervous system disorders** | **1 (12.5%) 1** |
| Myoclonus | 1 (12.5%) 1 |

Abbreviations: m = number of adverse events; n = number of participants with events.

Notes: Treatment-emergent adverse events (TEAEs) were defined as AEs that occurred or worsened from the first administration of INS018_055 up to and including the follow-up period. If a participant has multiple occurrences of a TEAE, the participant is presented only once in the Participant count (n) column for a given System Organ Class and Preferred Term. Occurrences are counted each time in the Occurrence (M) column. SOCs and PTs within each SOC is presented in decreasing order of treatment group.

**Table S8-2: Summary of Pharmacokinetic Parameters**

| Treatment Group | Parameter (Unit) | n | n NC | Mean | SD | Median | Minimum | Maximum | Geometric Mean |
|---|---|---|---|---|---|---|---|---|---|
| INS018_055 (N=8) | $AUC_{(0-24)}$ (h*ng/mL) | 8 | 0 | 2.576 | 0.630 | 2.710 | 1.59 | 3.57 | 2.504 |
| | $AUC_{(0-inf)}$ (h*ng/mL) | 8 | 0 | 2.650 | 0.679 | 2.755 | 1.60 | 3.77 | 2.570 |
| | $AUC_{(0-t)}$ (h*ng/mL) | 8 | 0 | 2.522 | 0.668 | 2.657 | 1.48 | 3.57 | 2.439 |
| | $AUC_{\%Extrap}$ | 8 | 0 | 5.075 | 1.911 | 4.541 | 3.33 | 8.10 | 4.787 |
| | C0 (ng/mL) | 8 | 0 | 12.460 | 11.937 | 9.342 | 0.52 | 33.33 | 6.470 |
| | CL (L/h) | 8 | 0 | 40.215 | 11.472 | 36.339 | 26.50 | 62.62 | 38.912 |
| | Kel (1/h) | 8 | 0 | 0.182 | 0.093 | 0.128 | 0.10 | 0.32 | 0.164 |
| | $t_{1/2}$ (h) | 8 | 0 | 4.644 | 1.936 | 5.397 | 2.13 | 6.95 | 4.228 |
| | $T_{last}$ (h) | 8 | 0 | 17.637 | 7.956 | 23.175 | 8.03 | 23.78 | 15.670 |
| | Vz (L) | 8 | 0 | 253.596 | 95.524 | 256.441 | 115.61 | 432.92 | 237.377 |

Abbreviations: $AUC_{(0-24)}$ = area under the drug concentration-time curve, from time zero to 24 hours; $AUC_{(0-inf)}$ = area under the drug concentration-time curve, from time zero to infinity; $AUC_{(0-t)}$ = area under the drug concentration-time curve, from time zero to the last time point with measurable concentration; $AUC_{\%Extrap}$ = area under the drug concentration-time curve, percent extrapolated; $C_0$ = hypothetical concentration at time zero; CL = apparent clearance; h = hour; Kel = apparent terminal elimination rate constant; L = litre; mL = milliliter; n = number; NC = not calculable; SD = standard deviation; $t_{1/2}$ = apparent plasma terminal half life; $T_{last}$ = time of final quantifiable concentration; Vz = apparent volume of distribution at terminal phase.
Source: Table 14.2.2

**Supplementary information 9: Phase I New Zealand Clinical Trial Full Information**

**Phase I study (New Zealand):**

The general design of clinical trial (NCT05154240) can be found in www.clinicaltrials.gov The randomized, double blind, placebo controlled study of INS018_055 was conducted from Feb 21, 2022 ( first subject administered first dose) until Sept 30th, 2022 ( last subject contacted).

**Statistical considerations:**

For Parts A and B, the determination of sample sizes were based on previous clinical and practical considerations instead of formal statistical power calculation. For Part C, sample size of 16 subjects was considered sufficient to detect any possible interaction between INS018_055 and caffeine. To conclude, the sample sizes of 40 and 24 subjects enrolled in Parts A and B, respectively, were considered sufficient for the objectives of the study.

**Study design and participants: (facilities, in/ex)**

This is a phase I, randomized, double-blind, placebo-controlled, oral single and multiple ascending doses, parallel group and exploratory drug-drug interaction study to evaluate the safety, tolerability, pharmacokinetics, and interaction potential of INS018_055 in healthy subjects, conducted in one clinical site in New Zealand. Protocol and its amendments were approved by the IRB before implementation unless proceeding with the changes was in the subject's best interest. Planned enrollment was a total of 80 healthy male and female subjects (40, 24, 16 subjects in Part A, B and C, respectively), aged from 18 to 55 years, with body mass index (BMI) of from 18 to 32 $kg/m^2$, and a total body weight ≥50 kg, considered by the investigator to be in good general health based at screening.

**Inclusion criteria:**

Each subject must meet all of the following criteria to be enrolled in this study:

1. The subject is a male or female 18 to 55 years of age, inclusive.
2. The subject has a body mass index 18 to 32 kg/m2, inclusive, and a total body weight ≥50 kg, inclusive, at screening.
3. The subject is considered by the investigator to be in good general health as determined by medical history, clinical laboratory test results, vital sign measurements, 12-lead ECG results, and physical examination findings at screening.
4. Female subjects of childbearing potential must be non-pregnant and non-lactating and must use one of the methods of contraception listed below for the duration of the treatment until at least 28 days after the last dose of the study drug, or be surgically sterile (ie, hysterectomy, bilateral tubal ligation, or bilateral oophorectomy) or postmenopausal (defined as amenorrhea 12 consecutive months and documented plasma follicle-stimulating hormone level >40 IU/mL). Female subjects must have a negative pregnancy test at screening and before the first dose of study drug.

Highly effective methods of contraception are those that result in a failure rate of less than 1% per year when used consistently. Examples are provided below:
a. Implant contraceptive (eg, Jadelle®)
b. Intrauterine device (IUD) containing either copper or levonorgestrel (eg, Mirena®)
c. Male sterilization with absence of sperm in the post-vasectomy ejaculate

OR an effective method that results in a failure rate of less than 5% to 10% per year. Examples are provided below:
d. Injectable contraceptive (eg, Depo Provera)
e. Oral contraceptive pill (combined hormonal contraceptive pill or progestogen-only 'mini-pill')

f. Vaginal contraceptive ring (eg, NuvaRing®)
Female subjects must also agree not to donate eggs, from dosing until at least 28 days after the last dose of study drug.
A male subject and his female partner who is of childbearing potential must agree to use one of the methods of contraception listed above for the duration of the treatment until at least 28 days after the last dose of the study drug. A male subject must also agree not to donate sperm, for the duration of the treatment until at least 28 days after the last dose of the study drug.

5. The subject agrees to comply with all protocol requirements.
6. The subject is able to provide written informed consent.

**Exclusion criteria:**

1. The subject has current evidence or history of clinically significant hematological, renal, endocrine, pulmonary, GI, cardiovascular, hepatic, psychiatric, neurologic, or allergic disease (including drug allergies, but excluding untreated, asymptomatic, seasonal allergies at time of dosing).
2. The subject has any condition possibly affecting drug absorption (eg, gastrectomy).
3. The subject has a history of cancer with the exception of adequately treated basal cell or squamous cell carcinoma of the skin.
4. The subject has supine blood pressure (BP) >140 mm Hg (systolic) or >90 mm Hg (diastolic), following at least 5 minutes of supine rest. If BP is >140 mm Hg (systolic) or >90 mm Hg (diastolic), the BP should be repeated 2 more times and the average of the 3 BP values should be used to determine the subject's eligibility at screening.
5. The subject has 12-lead ECG demonstrating corrected QT interval by Fridericia (QTcF) >450 msec, or a QRS interval >120 msec at screening. If QTcF exceeds 450 msec, or QRS interval exceeds 120 msec, the ECG should be repeated 2 more times and the average of the 3 QTcF (or QRS interval) values should be used to determine the subject's eligibility.
6. The subject has ANY of the following abnormalities in clinical laboratory tests at screening, as assessed by the study-specific laboratory and confirmed by a single repeat, if deemed necessary:
   a. Serum creatinine (SCr) level above the upper limit of normal (ULN) or an estimated glomerular filtration rate (GFR) value <80 mL/min/1.73 m2 calculated with the Chronic Kidney Disease Epidemiology Collaboration (CKD-EPI) formula and the absence of protein in urine, at screening.
   b. Aspartate aminotransferase (AST) or alanine aminotransferase (ALT) values more than >1.5 × ULN.
   c. Fasting glucose >110 mg/dL (6.1 mmol/L).
   d. Total bilirubin >1.5 × ULN.

e. Hematological values outside the normal reference range for local laboratory results.
f. Positive fecal occult blood test at screening or at check-in (Day -1).

7. The subject has any medical history of disease that has the potential to cause a rise in total bilirubin over the ULN. Subjects with borderline clinical laboratory values outside the reference range may be included in the study if the investigator deems that the values are not clinically significant. Note: Subjects with a history of Gilbert's syndrome may have a direct bilirubin measured and would be eligible for this study provided the direct bilirubin is <ULN.
8. The subject has a history of any lymphoproliferative disorder (such as Epstein Barr Virus-related lymphoproliferative disorder, as reported in some subjects on immunosuppressive drugs), history of lymphoma, leukemia, myeloproliferative disorders, multiple myeloma, or signs and symptoms suggestive of current lymphatic disease.
9. The subject has a history of relevant drug and/or food allergies (ie, allergy to any study drug or excipients, or any significant food allergy that could preclude a standard diet in the clinical unit).
10. The subject has a clinically significant infection currently or within 6 months of first dose of study drug (eg, those requiring hospitalization or parenteral antimicrobial therapy or opportunistic infections), or a history of chronic or recurrent infectious disease.
11. The subject has other severe acute or chronic medical or psychiatric condition including recent (within the past year) or active suicidal ideation or behavior or laboratory abnormality that may increase the risk associated with study participation or investigational product administration or may interfere with the interpretation of study results and, in the judgment of the investigator, would make the subject inappropriate for entry into this study.
12. The subject has or has had symptomatic herpes zoster or herpes simplex within 12 weeks, more than one episode of local herpes zoster, or a history (single episode) of disseminated zoster.
13. The subject has a positive test result for hepatitis B surface antigen, hepatitis C virus antibody, or human immunodeficiency virus (HIV) types 1 or 2 antibodies at screening.
14. The subject is a female who is pregnant or lactating.
15. The subject is a fertile male who is unwilling or unable to use a highly effective method of contraception as outlined in this protocol for the duration of the study and for at least 28 days after the last dose of investigational product.
16. The subject is unwilling or unable to comply with the lifestyle restrictions described in this protocol (Section 4.3.1).
17. The subject is a smoker or has used nicotine or nicotine-containing products (eg, snuff, nicotine patch, nicotine chewing gum, mock cigarettes, or inhalers) within 6 months before the first dose of study drug.
18. The subject has a positive test result for drugs of abuse or cotinine (indicating active current smoking) at screening or before the first dose of study drug.
19. The subject has used any prescription or over-the-counter medications (except paracetamol [up to 2 g per day]), including herbal supplements, within 14 days before the first dose of study drug. Nutritional supplements are allowed if unlikely to interfere with the study results and agreed by medical monitor and investigator.
20. The subject has consumed grapefruit or grapefruit juice, Seville orange or Seville orange-containing products (eg, marmalade), or alcohol-, caffeine-, or xanthine-containing products within 48 hours before the first dose of study drug.
21. The subject has used a known strong or moderate inhibitor or inducer of CYP1A2 within 4 weeks prior to Day 1 and through the last PK sampling point on Day 21 (only for Part C, DDI).
22. The subject will have vaccination with live virus, attenuated live virus, or any live viral components within the 2 weeks prior to the first dose of study

drug or is to receive these vaccines at any time during treatment or within 8 weeks following completion of study treatment.

23. The subject has a positive test result for severe acute respiratory syndrome-related coronavirus 2 (SARS-CoV-2). The subject has received the Coronavirus disease 2019 (COVID-19) vaccine within 2 weeks prior to the first dose of study drug or plans to receive a COVID-19 vaccine within 12 weeks after study drug dosing or has positive test for SARS-CoV-2 during screening or presence of COVID-19 symptoms within 4 weeks prior to Day -1.
24. The subject has undergone significant trauma or major surgery within 4 weeks of screening.
25. The subject has a bleeding risk: genetic predisposition to bleeding, a hemorrhagic event in the 12 months before the start of screening, or abnormal laboratory coagulation parameters.
26. The subject has a first-degree relative with a hereditary immunodeficiency.
27. The subject has investigator site staff member directly involved in the conduct of the study and their family members, site staff member otherwise supervised by the investigator, or subjects who are sponsor employees including their family members are directly involved in the conduct of the study.
28. The subject has a history of alcohol abuse or drug addiction within the last year or excessive alcohol consumption (regular alcohol intake >21 units per week for male subjects and >14 units of alcohol per week for female subjects) (1 unit is equal to approximately ½ pint [200 mL] of beer, 1 small glass [100 mL] of wine, or 1 measure [25 mL] of spirits) or use of alcohol 48 hours before the first dose of study drug.
29. The subject is involved in strenuous activity or contact sports within 24 hours before dosing and during the study.
30. The subject has donated blood or blood products >450 mL within 30 days before the first dose of study drug.
31. The subject has received study drug in another investigational study within 30 days of dosing
32. The subject received cytochrome P450 (CYP)/ multidrug and toxin extrusion (MATE) classes of medications within 4 weeks of first dose of INS018_055 or was likely to receive CYP/MATE classes of medications during the study.
33. In the opinion of the investigator, the subject is not suitable for entry into the study.

**Study procedures: (cohorts)**

Part A (SAD): After pre-dosing procedures, subjects were dosed on Day 1 with a corresponding dose of INS018_055 or a matching dose of placebo orally. Fasting required at least 10 hours before and 4 hours after dosing, and water was not allowed 1 hour and after dosing. When starting each cohort, 2 subjects (1 active and 1 placebo in a blinded fashion) as a sentinel cohort were dosed and then monitored for at least one day. After investigators confirmed the safety of treatment in the 2 subjects, dosings were initiated in the remaining 6 subjects. Dose escalation: Only after at least 6 subjects in preceding dose cohort were assessed for PK and tolerability, and considered safe and well tolerated by the safety review committee (SRC), dose escalation could occur. Dose escalation to cohorts 4 and 5 only occurred after PK data of at least 2 cohorts were assessed and the data was sufficient to model exposure for cohorts 4 and 5. Food Effect Assessment (only in Cohort 4, 90 mg): Following the standard SAD dosing on Day 1 and at least 3 days of washout (or 5 times the estimated half-life of INS018_055 based on the observed data, whichever was longer) (Period 1), subjects entered Period 2 and were repeated the same treatment as Period 1 (except for procedures in Day-1)

after completing a standard high-fat meal 30 minutes prior to dosing. Subjects fasted overnight for at least 10 hours and received a standard high-fat breakfast 30 minutes prior to dosing. Subjects remained at the study site on Day 4 of Period 2 until the scheduled procedures and review of the Day 4 safety data by the investigator (or designee) were completed.

Part B (MAD): The dosing schedule was decided by the SRC after review of the PK, safety, and tolerability data from the first 3 cohorts of Part A. The initial MAD cohorts began dosing with the remaining SAD cohorts. Dose escalation to the next cohort will only occur when the safety and tolerability data up to Day 11 (inclusive) of at least 6 subjects in the preceding MAD dose cohort were assessed and considered safe by the SRC. The top dose explored in Part B (120 mg, QD) did not exceed the maximum dose explored in Part A (120 mg, single dose). For the morning dosing on Days 1 and 7 for Cohorts 6 to 8, subjects fasted for at least 8 hours prior to and 4 hours after dosing and water was not allowed 1 hour prior to and 1 hour after dosing. Pharmacokinetic sampling and safety and tolerability assessments were performed at predefined time points throughout the study.

**Outcomes: (primary and secondary endpoints)**
The primary objective of this phase I clinical trial was to assess the safety and tolerability of single and multiple oral escalating doses of INS018_055 administered to healthy subjects. Assessments of safety and tolerability includes monitoring and recording of AEs (to be added), clinical laboratory test results (hematology, coagulation, serum chemistry, urinalysis, and fecal occult blood test) occurred at screening, check-in, day of discharge, and other time points specified in the SOE, vital sign measurements (systolic and diastolic BP, pulse rate, respiratory rate, and body temperature), 12-lead ECG results, and physical examination findings. The secondary objectives of this study includes the determination of pharmacokinetics (PK) of INS018_055 following single and multiple oral escalating doses in healthy subjects and the assessment of the effect of food on the PK of INS018_055 following an oral dose.

**PK analysis:**
For Part A: Blood samples for PK analysis were collected on Day 1 before dosing (within 45 min prior to dosing) and at 0.25 (± 5 min), 0.5 (± 5 min), 1 (± 5 min), 2 (± 5 min), 4 (± 5 min), 6 (± 10 min), 8 (± 10 min), 10 (± 10 min), and 12 hours (± 10 min); 24 (± 30 min) and 36 hours (± 30 min) on Day 2; 48 (± 60 min) on Day 3; and 72 hours (± 60 min) on Day 4 after administration of INS018_055.
For Part B: Blood samples for PK analysis were collected on Day 1: before dosing (0 hour) (within 45 min prior to dosing) and at 0.25 (± 5 min), 0.5 (± 5 min), 1 (± 5 min), 2 (± 5 min), 4 (± 5 min), 6 (± 10 min), 8 (± 10 min), 10 (± 10 min), and 12 hours (± 10 min) after dosing; Days 2, 3, 4, 5, and 6: before dosing, following at least an 8-hour fasting period before breakfast; Day 7: before dosing (0 hour) (within 45 min prior to dosing) and at 0.25 (± 5 min), 0.5 (± 5 min), 1 (± 5 min), 2 (± 5 min), 4 (± 5 min), 6 (± 10 min), 8 (± 10 min), 10 (± 10 min), 12 (± 10 min), 24 (± 30 min), 48 (± 60 min), and 72 hours (± 60 min) after dosing.

Pharmacokinetic samples were analyzed by 360biolabs (Victoria, Australia) using a validated assay using liquid chromatography coupled with tandem mass spectrometry for INS018_055.

Data analysis of PK results were analyzed as below: a non-linear power model was used to assess the dose proportionality within study parts (A and B). To examine the differences in $T_{max}$ between treatment, nonparametric methods (Wilcoxon signed-rank test) were used. Food effect: Following natural logarithmic transformation, PK parameters $AUC_{0\text{-}t}$, $AUC_{0\text{-}inf}$, and $C_{max}$ of INS018_055 were analyzed using a mixed effects model with treatment as fixed effects and subject as the random effect. The geometric least squares (LS) mean difference was calculated and 90% confidence intervals (CIs) of the difference in the geometric LS mean, consistent with the 2 one-sided tests, was provided. The comparison of interest was Fed (test) versus Fasted (reference). In Part B, steady state was evaluated by regression analysis of trough level concentrations of individual subjects from predose samples collected on Days 1 through Day 7.

**Table S9-1 Geometric Mean (Geometric CV%) Plasma Pharmacokinetic Parameters of INS018_055 - SAD – Pharmacokinetic Population**

| INS018_055 Parameter (unit) | Summary Statistics | INS018_055 Treatments | | | | | |
|---|---|---|---|---|---|---|---|
| | | 10 mg Fasted (N=6) | 30 mg Fasted (N=6) | 60 mg Fasted (N=6) | 90 mg Fasted (N=6) | 90 mg Fed (N=6) | 120 mg Fasted (N=6) |
| $C_{max}$ (ng/mL) | n | 6 | 6 | 6 | 6 | 6 | 6 |
| | Geometric Mean | 25.4 | 105 | 273 | 270 | 169 | 339 |
| | Geometric CV% | 66.8 | 44.4 | 47.5 | 44.1 | 42.8 | 32.1 |
| $T_{max}$ (h)[a] | n | 6 | 6 | 6 | 6 | 6 | 6 |
| | Median | 1.00 | 1.53 | 1.00 | 1.01 | 3.00 | 1.50 |
| | Minimum | 1.00 | 1.00 | 0.50 | 0.50 | 2.00 | 1.00 |
| | Maximum | 2.00 | 4.00 | 2.00 | 2.00 | 4.00 | 2.00 |
| $AUC_{0-t}$ (h*ng/mL) | n | 6 | 6 | 6 | 6 | 6 | 6 |
| | Geometric Mean | 124 | 552 | 1210 | 1620 | 1390 | 1980 |
| | Geometric CV% | 69.3 | 46.0 | 61.0 | 40.5 | 44.9 | 35.3 |
| $AUC_{0\text{--}inf}$ (h*ng/mL) | n | 6 | 6 | 6 | 6 | 6 | 6 |
| | Geometric Mean | 135 | 559 | 1220 | 1630 | 1400 | 2000 |
| | Geometric CV% | 63.3 | 45.7 | 61.0 | 40.4 | 44.6 | 35.1 |
| $t_{1/2}$ (h) | n | 6 | 6 | 6 | 6 | 6 | 6 |
| | Geometric Mean | 9.72 | 7.42 | 7.96 | 8.61 | 9.71 | 9.74 |
| | Geometric CV% | 23.8 | 9.30 | 23.6 | 48.9 | 35.0 | 39.2 |
| CL/F (L/h) | n | 6 | 6 | 6 | 6 | 6 | 6 |
| | Geometric Mean | 74.2 | 53.7 | 49.1 | 55.2 | 64.1 | 60.0 |
| | Geometric CV% | 63.3 | 45.7 | 61.0 | 40.4 | 44.6 | 35.1 |
| $V_z$/F (L) | n | 6 | 6 | 6 | 6 | 6 | 6 |
| | Geometric Mean | 1040 | 575 | 563 | 685 | 898 | 843 |
| | Geometric CV% | 68.7 | 39.2 | 39.9 | 29.2 | 22.9 | 61.0 |

Abbreviations: $AUC_{0\text{--}inf}$, AUC from time 0 extrapolated to infinity; $AUC_{0-t}$, AUC from time 0 to the last measurable observed concentration; CL/F, apparent total body clearance; $C_{max}$, maximum observed concentration; CV, coefficient of variation; n, number of observations; N, number of subjects in the treatment; SAD, single ascending dose; $t_{1/2}$, apparent terminal elimination half-life; $T_{max}$, time of maximum observed concentration; $V_z$/F, apparent volume of distribution during the terminal phase.

[a] For $T_{max}$, the median (minimum, maximum) values are presented.

**Table S9-2 Geometric Mean (Geometric CV%) Plasma Pharmacokinetic Parameters of INS018_055 - MAD – Pharmacokinetic Population**

| INS018_055 Parameter (unit) | Summary Statistics | INS018_055 Treatments | | | | | |
|---|---|---|---|---|---|---|---|
| | | Day 1 | Day 7 | Day 1 | Day 7 | Day 1 | Day 7 |
| | | 30 mg Fasted (N=6) | 30 mg Fasted (N=5) | 60 mg Fasted (N=6) | 60 mg Fasted (N=6) | 120 mg Fasted (N=6) | 120 mg Fasted (N=6) |
| $C_{max}$ (ng/mL) | N | 6 | 5 | 6 | 6 | 6 | 6 |
| | Geometric Mean | 106 | 79.4 | 226 | 191 | 511 | 463 |
| | Geometric CV% | 28.1 | 50.1 | 24.1 | 36.5 | 13.5 | 26.5 |
| $T_{max}$ (h)[a] | n | 6 | 5 | 6 | 6 | 6 | 6 |
| | Median | 1.00 | 1.00 | 1.00 | 1.50 | 1.50 | 2.00 |
| | Minimum | 1.00 | 1.00 | 1.00 | 0.52 | 1.00 | 1.00 |
| | Maximum | 1.00 | 1.98 | 2.00 | 2.00 | 4.00 | 2.00 |
| $AUC_{0-tau}$ (h*ng/mL) | n | 6 | 5 | 6 | 6 | 6 | 6 |
| | Geometric Mean | 404 | 439 | 1150 | 1210 | 3050 | 3040 |
| | Geometric CV% | 34.8 | 39.8 | 25.3 | 29.9 | 24.9 | 25.3 |
| $AUC_{0--t}$ (h*ng/mL) | n | 6 | 5 | 6 | 6 | 6 | 6 |
| | Geometric Mean | 404 | 473 | 1150 | 1340 | 3050 | 3380 |
| | Geometric CV% | 34.8 | 42.8 | 25.3 | 29.2 | 24.9 | 24.8 |
| $t_{1/2}$ (h) | n | NA | 5 | NA | 6 | NA | 6 |
| | Geometric Mean | NA | 9.36 | NA | 11.9 | NA | 10.2 |
| | Geometric CV% | NA | 24.4 | NA | 13.9 | NA | 11.4 |
| CL/F (L/h) | n | NA | 5 | NA | 6 | NA | 6 |
| | Geometric Mean | NA | 68.3 | NA | 49.7 | NA | 39.5 |
| | Geometric CV% | NA | 39.8 | NA | 29.9 | NA | 25.3 |
| $V_z$/F (L) | n | NA | 5 | NA | 6 | NA | 6 |
| | Geometric Mean | NA | 922 | NA | 850 | NA | 581 |
| | Geometric CV% | NA | 35.4 | NA | 25.3 | NA | 33.4 |
| $C_{av}$ (ng/mL) | n | NA | 5 | NA | 6 | NA | 6 |
| | Geometric Mean | NA | 18.3 | NA | 50.3 | NA | 127 |
| | Geometric CV% | NA | 39.8 | NA | 29.9 | NA | 25.3 |
| PTR | n | NA | 5 | NA | 6 | NA | 6 |
| | Geometric Mean | NA | 30.8 | NA | 27.1 | NA | 23.1 |
| | Geometric CV% | NA | 16.1 | NA | 30.7 | NA | 24.8 |
| $ARC_{max}$ | n | NA | 5 | NA | 6 | NA | 6 |
| | Geometric Mean | NA | 0.739 | NA | 0.843 | NA | 0.905 |
| | Geometric CV% | NA | 25.9 | NA | 33.0 | NA | 21.7 |
| ARAUC | n | NA | 5 | NA | 6 | NA | 6 |
| | Geometric Mean | NA | 1.02 | NA | 1.05 | NA | 0.996 |
| | Geometric CV% | NA | 20.0 | NA | 5.81 | NA | 3.66 |

Abbreviations: ARAUC, accumulation ratio based on AUC; $ARC_{max}$, accumulation ratio based on $C_{max}$; $AUC_{0-t}$, AUC from time 0 to the last measurable observed concentration; $AUC_{0-tau}$, AUC within a dosing interval; $C_{av}$, average concentration within a dosing interval; CL/F, apparent total body clearance; $C_{max}$, maximum observed concentration; CV, coefficient of variation; MAD, multiple ascending dose; n, number of observations; N, number of subjects in the treatment; NA, not applicable; PTR, peak to trough ratio; $t_{1/2}$, apparent terminal elimination half-life; $T_{max}$, time of maximum observed concentration; $V_z/F$, apparent volume of distribution during the terminal phase.

Dosing interval (tau) is 24 hours.

[a] For $T_{max}$, the median (minimum, maximum) values are presented.

**Table S9-3** Statistical Assessment of Dose Proportionality of Plasma Pharmacokinetic Parameters of INS018_055 in SAD and MAD Pharmacokinetic Population

SAD:

| INS018_055 Dose Range | Parameter | n | Intercept | Estimated Slope | Standard Error of the Slope | 90% CI of the Slope |
|---|---|---|---|---|---|---|
| 10 mg – 120 mg | $C_{max}$ (ng/mL) | 30 | 0.932 | 1.06 | 0.0973 | (0.896 ,1.23) |
| | $AUC_{0\text{-}t}$ (h*ng/mL) | 30 | 2.33 | 1.13 | 0.0975 | (0.963 ,1.30) |
| | $AUC_{0\text{-}inf}$ (h*ng/mL) | 30 | 2.48 | 1.10 | 0.0944 | (0.937 ,1.26) |

Abbreviations: $AUC_{0\text{--}inf}$, AUC from time 0 extrapolated to infinity; $AUC_{0\text{-}t}$, AUC from time 0 to the last measurable observed concentration; CI, confidence interval, $C_{max}$, maximum observed concentration; n, number of observations; SAD, single ascending dose.

Note: The power model, ln(parameter) = intercept + slope*ln(dose), was used to estimate the slope and corresponding 90% CI.

MAD

| Day | INS018_055 Dose Range | Parameter | n | Intercept | Estimated Slope | Standard Error of the Slope | 90% CI of the Slope |
|---|---|---|---|---|---|---|---|
| 1 | 30 mg – 120 mg | $C_{max}$ (ng/mL) | 18 | 0.780 | 1.14 | 0.0906 | (0.980 ,1.30) |
| | | $AUC_{0\text{-}tau}$ (h*ng/mL) | 18 | 1.05 | 1.46 | 0.113 | (1.26 ,1.66) |
| 7 | 30 mg – 120 mg | $C_{max}$ (ng/mL) | 17 | 0.0473 | 1.27 | 0.154 | (1.00 ,1.54) |
| | | $AUC_{0\text{-}tau}$ (h*ng/mL) | 17 | 1.36 | 1.39 | 0.130 | (1.17 ,1.62) |

Abbreviations: $AUC_{0\text{-}tau}$, AUC within a dosing interval; CI, confidence interval; $C_{max}$, maximum observed concentration; n, number of observations; MAD, multiple ascending dose.

Note: The power model, ln(parameter) = intercept + slope*ln(dose), was used to estimate the slope and corresponding 90% CI.

**Table S9-4 Overall Summary of Treatment-Emergent Adverse Events - Part A (Safety Population)**

| | INS018_055 10 mg Fasted (N=6) n (%) [E] | INS018_055 30 mg Fasted (N=6) n (%) [E] | INS018_055 60 mg Fasted (N=6) n (%) [E] | INS018_055 90 mg Fasted (N=6) n (%) [E] | INS018_055 90 mg Fed (N=6) n (%) [E] | INS018_055 120 mg Fasted (N=6) n (%) [E] | Pooled Placebo (N=10) n (%) [E] | Any INS018_055 (N=30) n (%) [E] | Total (N=40) n (%) [E] |
|---|---|---|---|---|---|---|---|---|---|
| Any TEAE | 2 (33.3) [3] | 1 (16.7) [1] | 3 (50.0) [4] | 3 (50.0) [3] | 1 (16.7) [1] | 1 (16.7) [2] | 4 (40.0) [7] | 11 (36.7) [14] | 15 (37.5) [21] |
| Any Treatment-Related TEAE | 1 (16.7) [1] | 0 [0] | 2 (33.3) [2] | 0 [0] | 0 [0] | 1 (16.7) [1] | 4 (40.0) [6] | 4 (13.3) [4] | 8 (20.0) [10] |
| Any Mild TEAE | 2 (33.3) [3] | 1 (16.7) [1] | 3 (50.0) [4] | 3 (50.0) [3] | 1 (16.7) [1] | 1 (16.7) [2] | 4 (40.0) [7] | 11 (36.7) [14] | 15 (37.5) [21] |
| Any Treatment-Related Mild TEAE | 1 (16.7) [1] | 0 [0] | 2 (33.3) [2] | 0 [0] | 0 [0] | 1 (16.7) [1] | 4 (40.0) [6] | 4 (13.3) [4] | 8 (20.0) [10] |
| Any Moderate TEAE | 0 [0] | 0 [0] | 0 [0] | 0 [0] | 0 [0] | 0 [0] | 0 [0] | 0 [0] | 0 [0] |
| Any Treatment-Related Moderate TEAE | 0 [0] | 0 [0] | 0 [0] | 0 [0] | 0 [0] | 0 [0] | 0 [0] | 0 [0] | 0 [0] |
| Any Severe TEAE | 0 [0] | 0 [0] | 0 [0] | 0 [0] | 0 [0] | 0 [0] | 0 [0] | 0 [0] | 0 [0] |
| Any Treatment-Related Severe TEAE | 0 [0] | 0 [0] | 0 [0] | 0 [0] | 0 [0] | 0 [0] | 0 [0] | 0 [0] | 0 [0] |
| Any SAE | 0 [0] | 0 [0] | 0 [0] | 0 [0] | 0 [0] | 0 [0] | 0 [0] | 0 [0] | 0 [0] |
| Any Treatment-Related SAE | 0 [0] | 0 [0] | 0 [0] | 0 [0] | 0 [0] | 0 [0] | 0 [0] | 0 [0] | 0 [0] |
| Any TEAE Leading to Early Discontinuation | 0 [0] | 0 [0] | 0 [0] | 0 [0] | 0 [0] | 0 [0] | 0 [0] | 0 [0] | 0 [0] |
| Any Death | 0 [0] | 0 [0] | 0 [0] | 0 [0] | 0 [0] | 0 [0] | 0 [0] | 0 [0] | 0 [0] |

Abbreviations: AE, adverse event; SAE, serious adverse event; TEAE, treatment-emergent adverse event.

Note: A TEAE is defined as any event not present before exposure to study drug or any event already present that worsens in severity or frequency after exposure. A treatment-related TEAE is defined as any TEAE that is related to the treatment. At each level of subject summarization, a subject is counted once if the subject reported one or more events. In Cohort 4 (INS018_055 90 mg Fasted, INS018_055 90 mg Fed, and matching Placebos), a subject with the most related/severe event will be counted in the "Any INS018_055", "Pooled Placebo", and "Total" column summaries. n represents the number of subjects at each level of summarization. Percentages are based on the number of subjects in the Safety Population within each column. [E] represents the number of events at each level of summarization.

Source Data: Table 14.3.1.1.

**Table S9-5 Overall Summary of Treatment-Emergent Adverse Events - Part B (Safety Population)**

| | INS018_055 30 mg Fasted (N=6) n (%) [E] | INS018_055 60 mg Fasted (N=6) n (%) [E] | INS018_055 120 mg Fasted (N=6) n (%) [E] | Pooled Placebo (N=6) n (%) [E] | Any INS018_055 (N=18) n (%) [E] | Total (N=24) n (%) [E] |
|---|---|---|---|---|---|---|
| Any TEAE | 5 (83.3) [16] | 5 (83.3) [17] | 6 (100.0) [13] | 5 (83.3) [9] | 16 (88.9) [46] | 21 (87.5) [55] |
| Any Treatment-Related TEAE | 2 (33.3) [2] | 5 (83.3) [10] | 6 (100.0) [12] | 1 (16.7) [1] | 13 (72.2) [24] | 14 (58.3) [25] |
| Any Mild TEAE | 5 (83.3) [15] | 5 (83.3) [17] | 6 (100.0) [13] | 5 (83.3) [9] | 16 (88.9) [45] | 21 (87.5) [54] |
| Any Treatment-Related Mild TEAE | 2 (33.3) [2] | 5 (83.3) [10] | 6 (100.0) [12] | 1 (16.7) [1] | 13 (72.2) [24] | 14 (58.3) [25] |
| Any Moderate TEAE | 1 (16.7) [1] | 0 [0] | 0 [0] | 0 [0] | 1 (5.6) [1] | 1 (4.2) [1] |
| Any Treatment-Related Moderate TEAE | 0 [0] | 0 [0] | 0 [0] | 0 [0] | 0 [0] | 0 [0] |
| Any Severe TEAE | 0 [0] | 0 [0] | 0 [0] | 0 [0] | 0 [0] | 0 [0] |
| Any Treatment-Related Severe TEAE | 0 [0] | 0 [0] | 0 [0] | 0 [0] | 0 [0] | 0 [0] |
| Any SAE | 0 [0] | 0 [0] | 0 [0] | 0 [0] | 0 [0] | 0 [0] |
| Any Treatment-Related SAE | 0 [0] | 0 [0] | 0 [0] | 0 [0] | 0 [0] | 0 [0] |
| Any TEAE Leading to Early Discontinuation | 1 (16.7) [1] | 0 [0] | 0 [0] | 0 [0] | 1 (5.6) [1] | 1 (4.2) [1] |
| Any Death | 0 [0] | 0 [0] | 0 [0] | 0 [0] | 0 [0] | 0 [0] |

Abbreviations: AE, adverse event; SAE, serious adverse event; TEAE, treatment-emergent adverse event.

Note: A TEAE is defined as any event not present before exposure to study drug or any event already present that worsens in severity or frequency after exposure. A treatment-related TEAE is defined as any TEAE that is related to the treatment. At each level of subject summarization, a subject is counted once if the subject reported one or more events. In Cohort 4 (INS018_055 90 mg Fasted, INS018_055 90 mg Fed, and matching Placebos), a subject with the most related/severe event will be counted in the "Any INS018_055", "Pooled Placebo", and "Total" column summaries. n represents the number of subjects at each level of summarization. Percentages are based on the number of subjects in the Safety Population within each column. [E] represents the number of events at each level of summarization.

**Table S9-6 Summary of Treatment-Emergent Adverse Events by System Organ Class and Preferred Term - Part A (Safety Population)**

| | INS018_055 10 mg Fasted (N=6) n (%) [E] | INS018_055 30 mg Fasted (N=6) n (%) [E] | INS018_055 60 mg Fasted (N=6) n (%) [E] | INS018_055 90 mg Fasted (N=6) n (%) [E] | INS018_055 90 mg Fed (N=6) n (%) [E] | INS018_055 120 mg Fasted (N=6) n (%) [E] | Pooled Placebo (N=10) n (%) [E] | Any INS018_055 (N=30) n (%) [E] | Total (N=40) n (%) [E] |
|---|---|---|---|---|---|---|---|---|---|
| Any TEAE | 2 (33.3) [3] | 1 (16.7) [1] | 3 (50.0) [4] | 3 (50.0) [3] | 1 (16.7) [1] | 1 (16.7) [2] | 4 (40.0) [7] | 11 (36.7) [14] | 15 (37.5) [21] |
| Investigations | 1 (16.7) [1] | 0 [0] | 1 (16.7) [1] | 0 [0] | 0 [0] | 0 [0] | 2 (20.0) [2] | 2 (6.7) [2] | 4 (10.0) [4] |
| Fecal occult blood positive | 1 (16.7) [1] | 0 [0] | 1 (16.7) [1] | 0 [0] | 0 [0] | 0 [0] | 2 (20.0) [2] | 2 (6.7) [2] | 4 (10.0) [4] |
| Nervous system disorders | 1 (16.7) [1] | 0 [0] | 1 (16.7) [1] | 0 [0] | 0 [0] | 0 [0] | 2 (20.0) [3] | 2 (6.7) [2] | 4 (10.0) [5] |
| Headache | 0 [0] | 0 [0] | 1 (16.7) [1] | 0 [0] | 0 [0] | 0 [0] | 1 (10.0) [1] | 1 (3.3) [1] | 2 (5.0) [2] |
| Dizziness | 0 [0] | 0 [0] | 0 [0] | 0 [0] | 0 [0] | 0 [0] | 1 (10.0) [1] | 0 [0] | 1 (2.5) [1] |
| Paraesthesia | 0 [0] | 0 [0] | 0 [0] | 0 [0] | 0 [0] | 0 [0] | 1 (10.0) [1] | 0 [0] | 1 (2.5) [1] |
| Syncope | 1 (16.7) [1] | 0 [0] | 0 [0] | 0 [0] | 0 [0] | 0 [0] | 0 [0] | 1 (3.3) [1] | 1 (2.5) [1] |
| General disorders and administration site conditions | 1 (16.7) [1] | 0 [0] | 0 [0] | 2 (33.3) [2] | 0 [0] | 0 [0] | 0 [0] | 3 (10.0) [3] | 3 (7.5) [3] |
| Medical device site irritation | 0 [0] | 0 [0] | 0 [0] | 2 (33.3) [2] | 0 [0] | 0 [0] | 0 [0] | 2 (6.7) [2] | 2 (5.0) [2] |
| Fatigue | 1 (16.7) [1] | 0 [0] | 0 [0] | 0 [0] | 0 [0] | 0 [0] | 0 [0] | 1 (3.3) [1] | 1 (2.5) [1] |

| | INS018_055 10 mg Fasted (N=6) n (%) [E] | INS018_055 30 mg Fasted (N=6) n (%) [E] | INS018_055 60 mg Fasted (N=6) n (%) [E] | INS018_055 90 mg Fasted (N=6) n (%) [E] | INS018_055 90 mg Fed (N=6) n (%) [E] | INS018_055 120 mg Fasted (N=6) n (%) [E] | Pooled Placebo (N=10) n (%) [E] | Any INS018_055 (N=30) n (%) [E] | Total (N=40) n (%) [E] |
|---|---|---|---|---|---|---|---|---|---|
| Vascular disorders | 0 [0] | 1 (16.7) [1] | 1 (16.7) [1] | 0 [0] | 0 [0] | 1 (16.7) [1] | 0 [0] | 3 (10.0) [3] | 3 (7.5) [3] |
| Phlebitis | 0 [0] | 1 (16.7) [1] | 1 (16.7) [1] | 0 [0] | 0 [0] | 1 (16.7) [1] | 0 [0] | 3 (10.0) [3] | 3 (7.5) [3] |
| Infections and infestations | 0 [0] | 0 [0] | 1 (16.7) [1] | 0 [0] | 0 [0] | 0 [0] | 1 (10.0) [1] | 1 (3.3) [1] | 2 (5.0) [2] |
| COVID-19 | 0 [0] | 0 [0] | 1 (16.7) [1] | 0 [0] | 0 [0] | 0 [0] | 1 (10.0) [1] | 1 (3.3) [1] | 2 (5.0) [2] |
| Gastrointestinal disorders | 0 [0] | 0 [0] | 0 [0] | 0 [0] | 0 [0] | 1 (16.7) [1] | 0 [0] | 1 (3.3) [1] | 1 (2.5) [1] |
| Constipation | 0 [0] | 0 [0] | 0 [0] | 0 [0] | 0 [0] | 1 (16.7) [1] | 0 [0] | 1 (3.3) [1] | 1 (2.5) [1] |
| Musculoskeletal and connective tissue disorders | 0 [0] | 0 [0] | 0 [0] | 0 [0] | 1 (16.7) [1] | 0 [0] | 0 [0] | 1 (3.3) [1] | 1 (2.5) [1] |
| Musculoskeletal stiffness | 0 [0] | 0 [0] | 0 [0] | 0 [0] | 1 (16.7) [1] | 0 [0] | 0 [0] | 1 (3.3) [1] | 1 (2.5) [1] |
| Respiratory, thoracic and mediastinal disorders | 0 [0] | 0 [0] | 0 [0] | 1 (16.7) [1] | 0 [0] | 0 [0] | 0 [0] | 1 (3.3) [1] | 1 (2.5) [1] |
| Cough | 0 [0] | 0 [0] | 0 [0] | 1 (16.7) [1] | 0 [0] | 0 [0] | 0 [0] | 1 (3.3) [1] | 1 (2.5) [1] |
| Skin and subcutaneous tissue disorders | 0 [0] | 0 [0] | 0 [0] | 0 [0] | 0 [0] | 0 [0] | 1 (10.0) [1] | 0 [0] | 1 (2.5) [1] |
| Alopecia | 0 [0] | 0 [0] | 0 [0] | 0 [0] | 0 [0] | 0 [0] | 1 (10.0) [1] | 0 [0] | 1 (2.5) [1] |

| | INS018_055 10 mg Fasted (N=6) n (%) [E] | INS018_055 30 mg Fasted (N=6) n (%) [E] | INS018_055 60 mg Fasted (N=6) n (%) [E] | INS018_055 90 mg Fasted (N=6) n (%) [E] | INS018_055 90 mg Fed (N=6) n (%) [E] | INS018_055 120 mg Fasted (N=6) n (%) [E] | Pooled Placebo (N=10) n (%) [E] | Any INS018_055 (N=30) n (%) [E] | Total (N=40) n (%) [E] |
|---|---|---|---|---|---|---|---|---|---|

Abbreviations: COVID-19, coronavirus disease 2019; TEAE, treatment emergent adverse event.

Note: A TEAE is defined as any event not present before exposure to study drug or any event already present that worsens in severity or frequency after exposure. At each level of subject summarization, a subject is counted once if the subject reported one or more events. In Cohort 4 (INS018_055 90 mg Fasted, INS018_055 90 mg Fed, and matching Placebos), a subject with the most related/severe event will be counted in the "Any INS018_055", "Pooled Placebo", and "Total" column summaries. n represents the number of subjects at each level of summarization.
Percentages are based on the number of subjects in the Safety Population within each column.
[E] represents the number of events at each level of summarization. Adverse events were coded using MedDRA version 25.1.

**Table S9-7 Summary of Treatment-Emergent Adverse Events by System Organ Class and Preferred Term - Part B (Safety Population)**

| | INS018_055 30 mg Fasted (N=6) n (%) [E] | INS018_055 60 mg Fasted (N=6) n (%) [E] | INS018_055 120 mg Fasted (N=6) n (%) [E] | Pooled Placebo (N=6) n (%) [E] | Any INS018_055 (N=18) n (%) [E] | Total (N=24) n (%) [E] |
|---|---|---|---|---|---|---|
| Any TEAE | 5 (83.3) [16] | 5 (83.3) [17] | 6 (100.0) [13] | 5 (83.3) [9] | 16 (88.9) [46] | 21 (87.5) [55] |
| Nervous system disorders | 3 (50.0) [5] | 3 (50.0) [6] | 4 (66.7) [4] | 0 [0] | 10 (55.6) [15] | 10 (41.7) [15] |
| Headache | 3 (50.0) [4] | 3 (50.0) [6] | 4 (66.7) [4] | 0 [0] | 10 (55.6) [14] | 10 (41.7) [14] |
| Dizziness | 1 (16.7) [1] | 0 [0] | 0 [0] | 0 [0] | 1 (5.6) [1] | 1 (4.2) [1] |

| | INS018_055 30 mg Fasted (N=6) n (%) [E] | INS018_055 60 mg Fasted (N=6) n (%) [E] | INS018_055 120 mg Fasted (N=6) n (%) [E] | Pooled Placebo (N=6) n (%) [E] | Any INS018_055 (N=18) n (%) [E] | Total (N=24) n (%) [E] |
|---|---|---|---|---|---|---|
| Investigations | 1 (16.7) [1] | 5 (83.3) [6] | 3 (50.0) [5] | 0 [0] | 9 (50.0) [12] | 9 (37.5) [12] |
| Fecal occult blood positive | 1 (16.7) [1] | 4 (66.7) [5] | 2 (33.3) [2] | 0 [0] | 7 (38.9) [8] | 7 (29.2) [8] |
| Alanine aminotransferase increased | 0 [0] | 0 [0] | 1 (16.7) [1] | 0 [0] | 1 (5.6) [1] | 1 (4.2) [1] |
| Aspartate aminotransferase increased | 0 0] | 0 [0] | 1 (16.7) [1] | 0 [0] | 1 (5.6) [1] | 1 (4.2) [1] |
| Blood creatine phosphokinase increased | 0 [0] | 0 [0] | 1 (16.7) [1] | 0 [0] | 1 (5.6) [1] | 1 (4.2) [1] |
| Lymph node palpable | 0 [0] | 1 (16.7) [1] | 0 [0] | 0 [0] | 1 (5.6) [1] | 1 (4.2) [1] |
| General disorders and administration site conditions | 4 (66.7) [4] | 0 [0] | 1 (16.7) [1] | 2 (33.3) [2] | 5 (27.8) [5] | 7 (29.2) [7] |
| Catheter site inflammation | 0 [0] | 0 [0] | 1 (16.7) [1] | 1 (16.7) [1] | 1 (5.6) [1] | 2 (8.3) [2] |
| Catheter site bruise | 1 (16.7) [1] | 0 [0] | 0 [0] | 0 [0] | 1 (5.6) [1] | 1 (4.2) [1] |
| Catheter site pain | 0 [0] | 0 [0] | 0 [0] | 1 (16.7) [1] | 0 [0] | 1 (4.2) [1] |
| Catheter site swelling | 1 (16.7) [1] | 0 [0] | 0 [0] | 0 [0] | 1 (5.6) [1] | 1 (4.2) [1] |
| Fatigue | 1 (16.7) [1] | 0 [0] | 0 [0] | 0 [0] | 1 (5.6) [1] | 1 (4.2) [1] |
| Influenza like illness | 1 (16.7) [1] | 0 [0] | 0 [0] | 0 [0] | 1 (5.6) [1] | 1 (4.2) [1] |
| Gastrointestinal disorders | 1 (16.7) [1] | 2 (33.3) [2] | 1 (16.7) [1] | 2 (33.3) [2] | 4 (22.2) [4] | 6 (25.0) [6] |
| Nausea | 0 [0] | 0 [0] | 1 (16.7) [1] | 1 (16.7) [1] | 1 (5.6) [1] | 2 (8.3) [2] |
| Abdominal pain | 1 (16.7) [1] | 0 [0] | 0 [0] | 0 [0] | 1 (5.6) [1] | 1 (4.2) [1] |
| Aphthous ulcer | 0 [0] | 1 (16.7) [1] | 0 [0] | 0 [0] | 1 (5.6) [1] | 1 (4.2) [1] |
| Diarrhoea | 0 [0] | 1 (16.7) [1] | 0 [0] | 0 [0] | 1 (5.6) [1] | 1 (4.2) [1] |
| Gastrointestinal disorder | 0 [0] | 0 [0] | 0 [0] | 1 (16.7) [1] | 0 [0] | 1 (4.2) [1] |
| Infections and infestations | 4 (66.7) [4] | 0 [0] | 0 [0] | 0 [0] | 4 (22.2) [4] | 4 (16.7) [4] |

| | INS018_055 30 mg Fasted (N=6) n (%) [E] | INS018_055 60 mg Fasted (N=6) n (%) [E] | INS018_055 120 mg Fasted (N=6) n (%) [E] | Pooled Placebo (N=6) n (%) [E] | Any INS018_055 (N=18) n (%) [E] | Total (N=24) n (%) [E] |
|---|---|---|---|---|---|---|
| Upper respiratory tract infection | 3 (50.0) [3] | 0 [0] | 0 [0] | 0 [0] | 3 (16.7) [3] | 3 (12.5) [3] |
| COVID-19 | 1 (16.7) [1] | 0 [0] | 0 [0] | 0 [0] | 1 (5.6) [1] | 1 (4.2) [1] |
| Respiratory, thoracic and mediastinal disorders | 0 [0] | 1 (16.7) [1] | 0 [0] | 2 (33.3) [3] | 1 (5.6) [1] | 3 (12.5) [4] |
| Rhinorrhoea | 0 [0] | 0 [0] | 0 [0] | 2 (33.3) [3] | 0 [0] | 2 (8.3) [3] |
| Nasal congestion | 0 [0] | 1 (16.7) [1] | 0 [0] | 0 [0] | 1 (5.6) [1] | 1 (4.2) [1] |
| Skin and subcutaneous tissue disorders | 1 (16.7) [1] | 0 [0] | 1 (16.7) [1] | 1 (16.7) [1] | 2 (11.1) [2] | 3 (12.5) [3] |
| Pruritus | 1 (16.7) [1] | 0 [0] | 0 [0] | 0 [0] | 1 (5.6) [1] | 1 (4.2) [1] |
| Skin mass | 0 [0] | 0 [0] | 1 (16.7) [1] | 0 [0] | 1 (5.6) [1] | 1 (4.2) [1] |
| Urticaria | 0 [0] | 0 [0] | 0 [0] | 1 (16.7) [1] | 0 [0] | 1 (4.2) [1] |
| Musculoskeletal and connective tissue disorders | 0 [0] | 1 (16.7) [1] | 0 [0] | 1 (16.7) [1] | 1 (5.6) [1] | 2 (8.3) [2] |
| Back pain | 0 [0] | 1 (16.7) [1] | 0 [0] | 1 (16.7) [1] | 1 (5.6) [1] | 2 (8.3) [2] |
| Immune system disorders | 0 [0] | 1 (16.7) [1] | 0 [0] | 0 [0] | 1 (5.6) [1] | 1 (4.2) [1] |
| Seasonal allergy | 0 [0] | 1 (16.7) [1] | 0 [0] | 0 [0] | 1 (5.6) [1] | 1 (4.2) [1] |
| Psychiatric disorders | 0 [0] | 0 [0] | 1 (16.7 [1] | 0 [0] | 1 (5.6) [1] | 1 (4.2) [1] |
| Nightmare | 0 [0] | 0 [0] | 1 (16.7) [1] | 0 [0] | 1 (5.6) [1] | 1 (4.2) [1] |

Abbreviations: COVID-19, coronavirus disease 2019; TEAE, treatment emergent adverse event.

Note: A TEAE is defined as any event not present before exposure to study drug or any event already present that worsens in severity or frequency after exposure. At each level of subject summarization, a subject is counted once if the subject reported one or more events. In Cohort 4 (INS018_055 90 mg Fasted, INS018_055 90 mg Fed, and matching Placebos), a subject with the most related/severe event will be counted in the "Any INS018_055", "Pooled Placebo", and "Total" column summaries. n represents the number of subjects at each level of summarization.
Percentages are based on the number of subjects in the Safety Population within each column.
[E] represents the number of events at each level of summarization. Adverse events were coded using MedDRA version 25.1.

## Supplementary information 10: Phase I China Full Clinical Trial Information

**Study design and Outcomes:**

Detailed information including facilities, inclusion/exclusion criteria could be found in link http://www.chinadrugtrials.org.cn/clinicaltrials.prosearch.dhtml (registration number: CTR20221542). SAD consists of cohorts receiving single dose of 30 mg, 60 mg, or 120 mg. MAD part consists of cohorts receiving 30 mg, 60 mg or 90 mg, BID for seven days.

**Sample Size Consideration:**

The number of subjects was determined based on clinical and practical considerations from previous, similar studies rather than on formal statistical certainty calculations. The total sample size of 56 subjects was considered sufficient for the purpose of the study. Following the successful enrollment and outcome of this study when 48 participants were enrolled, patient enrollment was terminated thus resulting in 48 total participants for this study which our clinical collaborators considered sufficient statistical power.

**Inclusion criteria:**

Each subject must meet all of the following criteria to be enrolled in this study:

1. Subjects were male or female between 18 and 45 years of age, inclusive.
2. Subjects had a body mass index of 19 to 26 kg/m2 (inclusive) and weighed ≥ 50 kg (inclusive) for men and ≥ 45 kg (inclusive) for women at the time of screening.
3. Based on medical history, clinical laboratory findings, vital sign measurements, 12-lead ECG results, physical examination, chest radiograph results, and serum virological findings at screening, the investigators concluded that the subjects were in good general health.
4. Female subjects of childbearing potential must be non-pregnant and non- lactating and must be using one of the following methods of contraception throughout the treatment period until at least 28 days after the last dose of study drug, or have been surgically sterilized (i.e., hysterectomy, bilateral tubal ligation, or bilateral oophorectomy) or are postmenopausal (defined as 12 consecutive months of amenorrhea with documented plasma follicle stimulating hormone levels > 40 IU/mL). Female subjects must have a negative pregnancy test result at screening and prior to the first dose of study drug.

A highly effective method of contraception is one that has a contraceptive failure rate of less than 1% per year when used consistently. Examples are as follows:

a. Implanted contraceptives (e.g., Jadelle® )

b. IUDs containing copper or levonorgestrel (e.g., Mirena® )

c. Male sterilization, no sperm in ejaculation after vasectomy

d. Double barrier method: condom and occlusion cap (diaphragm or cervical cap/dome cap), barrier method with spermicide (foam/gel/film/cream/suppository) must be used as an add-on

e. Abstinence, defined as complete and continuous avoidance of all heterosexual sex (including during the entire period of risk associated with study treatment), was allowed without contraception only if this was the subject's preferred and daily lifestyle.

or an effective method with a contraceptive failure rate of less than 5% to 10% per year. Examples are as follows:

f. Injectable contraceptives (e.g., Depo Provera)

g. Oral contraceptives (combination hormonal contraceptives or progestogen-only "mini-pills")

h. Vaginal contraceptive ring (e.g., NuvaRing® )

Female subjects must also agree not to donate eggs from the time of administration until at least 28 days after the last dose of study drug.

Male subjects and their fertile female partners must agree to use one of the above methods of contraception for the entire treatment period until at least 28 days after the last dose of study drug. Male subjects must also agree not to donate sperm for the entire treatment period until at least 28 days after the last dose of study drug.

5. Subjects agree to comply with all protocol requirements.
6. Subjects were able to provide written informed consent.

**Exclusion criteria:**

Subjects who met any of the following criteria were excluded from the study:

1. Subject has current evidence or history of clinically significant hematologic, renal, endocrine, pulmonary, gastrointestinal, cardiovascular, hepatic, psychiatric, neurologic, or allergic disease (including drug allergy, but excluding asymptomatic seasonal allergy that was untreated at the time of administration).
2. Subjects have any condition that may affect drug absorption (e.g., gastrectomy).
3. Subjects had a history of cancer, except adequately treated basal cell or squamous cell carcinoma of the skin.
4. The subject rests for at least 5 minutes with a blood pressure (BP) > 140 mm Hg (systolic) or > 90 mm Hg (diastolic). At screening, if BP is > 140 mm Hg (systolic) or > 90 mm Hg (diastolic), BP should be measured 2 additional times and the average of the 3 BP values should be used to determine the subject's eligibility to participate.
5. At screening, the subject's 12-lead ECG shows a QT interval (QTc) corrected by the Bazett formula (QTc=QT/RR0.5 ) > 450 msec, or a QRS interval > 120 msec. The average of three QTc (or QRS interval) values from three standard 12-lead ECGs (repeated at intervals of no more than 5 minutes) should be used to determine the subject's eligibility for participation.
6. At the time of screening, the subject has any of the following abnormalities in clinical laboratory tests (if necessary, retest once for confirmation):

a. At screening, serum creatinine levels above the upper limit of normal (ULN) or creatinine clearance (Ccr) < 80 mL/min using the Cockcroft - Gault formula (Appendix 3) and no protein in the urine.

b. Aspartate aminotransferase or alanine aminotransferase values > 1.5 × ULN.

c. Fasting glucose > 110 mg/dL (6.1 mmol/L).

d. Total bilirubin > 1.5 × ULN.

e. Routine blood test values that are outside the normal reference range of local laboratory findings and are considered clinically significant by the investigator.

f. Positive fecal occult blood test result at screening or registration (day -1).

7. Subjects have a history of any disease that may have caused total bilirubin to be higher than ULN. Subjects whose clinical laboratory test values are not significantly outside the reference range may be enrolled in this study if the investigator does not consider the values to be clinically significant.

Note: In subjects with a history of Gilbert's syndrome, direct bilirubin may be measured, and if direct bilirubin < ULN, the subject is eligible for this study.

8. Subjects have any history of lymphoproliferative disease (such as Epstein Barr virus-associated lymphoproliferative disease as reported by some subjects receiving immunosuppressive drugs), lymphoma, leukemia, myeloproliferative disease, multiple myeloma, or signs and symptoms suggestive of current lymphatic disease.

9. Subjects have a history of relevant drug and/or food allergies (i.e., allergy to any study drug or excipient, or any severe food allergy that could result in inability to consume the standard diet of the clinical institution).

10. Subject has a current or clinically significant infection (e.g., an infection requiring hospitalization or parenteral antimicrobial therapy or the presence of an opportunistic infection within the past 6 months) or a history of chronic or recurrent infectious disease within the 6 months prior to the first dose of study drug.

11. Subject has other serious acute or chronic medical or psychiatric illness (including recent (within the past year) or active suicidal ideation or behavior or abnormal experimental results (which may increase the risk associated with study participation or experimental drug administration or may interfere with the interpretation of study results and, in the judgment of the investigator, may render the subject unsuitable for entry into this study).

12. Subjects have a history of symptomatic herpes zoster or herpes simplex, more than one episode of localized herpes zoster, or disseminated herpes zoster (single episode) present or within 12 weeks.

13. Subjects tested positive for hepatitis B surface antigen, hepatitis C virus antibody, or human immunodeficiency virus type 1 or 2 antibody at screening.

14. Subjects were pregnant or lactating females.

15. Subjects are men of childbearing potential who are unwilling or unable to use the contraceptive methods described in this protocol throughout the study period and for at least 28 days after the last dose of the experimental drug.

16. Subjects are unwilling or unable to comply with the lifestyle restrictions described in this protocol.

17. Subjects were smokers or had used nicotine or nicotine-containing products (e.g., snus, nicotine patches, nicotine chewing gum, simulated cigarettes, or inhalants) within 6 months prior to the first dose of the study drug.

18. Subjects tested positive for substance abuse or cotinine (indicating current active smoking) prior to the first dose of study drug.

19. Subjects have used any prescription or over-the-counter medication (except paracetamol [up to 2 g/day]), including herbal supplements, within 14 days prior to the first dose of study drug. Nutritional supplements are permitted provided that they are unlikely to interfere with the study results and that investigator consent has been obtained.

20. Subjects ingested grapefruit or grapefruit juice, limes or products containing limes (e.g., orange marmalade) or products containing alcohol, caffeine or xanthines within 48 hours prior to the first dose of the study drug.

21. Subjects will be vaccinated with live virus, live attenuated virus, or any live viral component within 2 weeks prior to the first dose of study drug, or will receive these vaccines at any time during the study or within 8 weeks of study completion.
22. Subject tested positive for Severe Acute Respiratory Syndrome-associated Coronavirus 2 (SARS-CoV-2). Subject received the 2019 coronavirus disease (COVID-19) vaccine within 2 weeks prior to the first dose of study drug or is scheduled to receive the COVID-19 vaccine within 12 weeks of study drug administration, or tested positive for SARS-CoV-2 during screening or had COVID-19 symptoms within 4 weeks prior to Day -1.
23. Subjects who have undergone major trauma or major surgery within 4 weeks prior to screening or who are expected to require major surgery during the trial.
24. Subjects are at risk for bleeding: genetic predisposition to bleeding, a bleeding event within 12 months prior to screening start, or abnormal laboratory coagulation parameters.
25. Subjects have a first-degree relative with a genetic immunodeficiency.
26. Subjects were study site staff and their family members who were directly involved in the implementation of the study, study site staff who were otherwise supervised by the investigator, or sponsor employees (including their family members) who were directly involved in the implementation of the study.
27. Subject has a history of alcohol abuse or drug addiction or excessive alcohol consumption (regular alcohol intake > 21 units/week for male subjects and > 14 units/week for female subjects; 1 unit equals approximately ½ pint [200 mL] of beer, 1 small glass [100 mL] of wine, or 1 cup [25 mL] of spirits) within the past year or consumed alcohol 24 hours prior to the first dose of study drug.
28. Subjects engaged in strenuous activity or contact sports within 24 hours prior to dosing and during the study.
29. The subject donated > 450 mL of blood or blood products within 30 days prior to the first dose of the study drug.
30. Subjects received study drug in another pilot study within 30 days prior to dosing or 5 drug half-lives, whichever is longer.
31. Subjects received cytochrome P450 (CYP3A4 and CYP2C8) and P-gp inhibitors and/or inducers within 4 weeks prior to the first dose of INS018_055 or may have received CYP3A4 and CYP2C8 and P-gp inhibitors and/or inducers during the study.
32. Subjects were deemed by the investigator to be unsuitable for enrollment in the study.

**Primary Objective:**
The primary objective of this study is to evaluate the safety and tolerability of single and multiple dose escalation oral administration of INS018_055 in healthy subjects.

**Secondary Objective:**
The secondary objectives of this study is to determine the PK characteristics of INS018_055 in healthy subjects after receiving single and multiple incremental oral doses of the drug.

**PK analysis:**
SAD: Blood samples for PK analysis were collected on Day 1 before dosing (within 45 min prior to dosing) and at 0.25 (± 5 min), 0.5 (± 5 min), 1 (± 5 min), 2 (± 5 min), 4 (± 5 min), 6 (± 10 min), 8 (± 10 min), 10 (± 10 min), and 12 hours (± 10 min); 24 (± 30 min) and 36 hours (± 30 min) on Day 2; 48 (± 60 min) on Day 3; and 72 hours (± 60 min) on Day 4 after administration of INS018_055.

MAD: Blood samples for PK analysis were collected on Day 1: before morning dosing (0 hour) (within 45 min prior to dosing) and at 0.25 (± 5 min), 0.5 (± 5 min), 1 (± 5 min), 2 (± 5 min), 4 (± 5 min), 6 (± 10 min), 8 (± 10 min), 10 (± 10 min), and 12 hours (± 10 min) after morning dosing; Days 2, 3, 4, 5, and 6: before morning dosing; Day 7: before evening dosing (0 hour) (within 45 min prior to dosing) and at 0.25 (± 5 min), 0.5 (± 5 min), 1 (± 5 min), 2 (± 5 min), 4 (± 5 min), 6 (± 10 min), 8 (± 10 min), 10 (± 10 min), 12 (± 10 min), 24 (± 30 min), 48 (± 60 min), and 72 hours (± 60 min) after evening dosing.

**Table S10-1.** Summary of Treatment-Emergent Adverse Events by System Organ Class and Preferred Terms -SAD

| | 30 mg | | 60 mg | | 120 mg | | Placebo | | Any INS018_055 | | Total | |
|---|---|---|---|---|---|---|---|---|---|---|---|---|
| | N=6 | | N=6 | | N=6 | | N=6 | | N=18 | | N=24 | |
| | n | % | n | % | n | % | n | % | n | % | n | % |
| **Any TEAE** | 5 | 83.3 | 2 | 33.3 | 3 | 50.0 | 4 | 66.7 | 10 | 55.6 | 14 | 58.3 |
| **Investigations** | 4 | 66.7 | 0 | 0.0 | 2 | 33.3 | 4 | 66.7 | 6 | 33.3 | 10 | 41.7 |
| Fecal occult positive | 3 | 50.0 | 0 | 0.0 | 2 | 33.3 | 2 | 33.3 | 5 | 27.8 | 7 | 29.2 |
| Body temperature increased | 1 | 16.7 | 0 | 0.0 | 0 | 0.0 | 1 | 16.7 | 1 | 5.6 | 2 | 8.3 |
| Blood uric acid increased | 1 | 16.7 | 0 | 0.0 | 0 | 0.0 | 0 | 0.0 | 1 | 5.6 | 1 | 4.2 |
| White blood cells urine positive | 0 | 0.0 | 0 | 0.0 | 0 | 0.0 | 1 | 16.7 | 0 | 0.0 | 1 | 4.2 |
| Blood glucose increased | 0 | 0.0 | 0 | 0.0 | 0 | 0.0 | 1 | 16.7 | 0 | 0.0 | 1 | 4.2 |
| **Metabolism and nutrition disorders** | 2 | 33.3 | 1 | 16.7 | 2 | 33.3 | 0 | 0.0 | 5 | 27.8 | 5 | 20.8 |
| Hypertriglyceridaemia | 2 | 33.3 | 1 | 16.7 | 2 | 33.3 | 0 | 0.0 | 5 | 27.8 | 5 | 20.8 |
| **Cardiac disorders** | 1 | 16.7 | 1 | 16.7 | 0 | 0.0 | 1 | 16.7 | 2 | 11.1 | 3 | 12.5 |
| Sinus bradycardia | 1 | 16.7 | 1 | 16.7 | 0 | 0.0 | 1 | 16.7 | 2 | 11.1 | 3 | 12.5 |
| **Vascular disorders** | 1 | 16.7 | 0 | 0.0 | 0 | 0.0 | 0 | 0.0 | 1 | 5.6 | 1 | 4.2 |
| Hypotension | 1 | 16.7 | 0 | 0.0 | 0 | 0.0 | 0 | 0.0 | 1 | 5.6 | 1 | 4.2 |
| **Nervous system disorders** | 0 | 0.0 | 0 | 0.0 | 1 | 16.7 | 0 | 0.0 | 1 | 5.6 | 1 | 4.2 |
| Headache | 0 | 0.0 | 0 | 0.0 | 1 | 16.7 | 0 | 0.0 | 1 | 5.6 | 1 | 4.2 |
| **Gastrointestinal disorders** | 0 | 0.0 | 0 | 0.0 | 1 | 16.7 | 0 | 0.0 | 1 | 5.6 | 1 | 4.2 |

| | | | | | | | | | | | | |
|---|---|---|---|---|---|---|---|---|---|---|---|---|
| Noninfective gingivitis | 0 | 0.0 | 0 | 0.0 | 1 | 16.7 | 0 | 0.0 | 1 | 5.6 | 1 | 4.2 |

Abbreviations :TEAE, treatment emergent adverse event.
Note: A TEAE is defined as any event not present before exposure to study drug or any event already present that worsens in severity or frequency after exposure. At each level of subject summarization, a subject is counted once if the subject reported one or more events. A subject with the most related/severe event will be counted in the "Any INS018_055", "Pooled Placebo", and "Total" column summaries. n represents the number of subjects at each level of summarization. Percentages are based on the number of subjects in the Safety Population within each column. Adverse events were coded using MedDRA version 25.1.

**Table S10-2**. Summary of Treatment-Emergent Adverse Events by System Organ Class and Preferred Terms -MAD

| | **30 mg** | | **60 mg** | | **90 mg** | | **Placebo** | | **Any INS018_055** | | **Total** | |
|---|---|---|---|---|---|---|---|---|---|---|---|---|
| | **N=6** | | **N=6** | | **N=6** | | **N=6** | | **N=18** | | **N=24** | |
| | **n** | **%** | **n** | **%** | **n** | **%** | **n** | **%** | **n** | **%** | **n** | **%** |
| **Any TEAE** | 4 | 66.7 | 6 | 100.0 | 6 | 100.0 | 5 | 83.3 | 16 | 88.9 | 21 | 87.5 |
| **Investigations** | 3 | 50.0 | 6 | 100.0 | 5 | 83.3 | 4 | 66.7 | 14 | 77.8 | 18 | 75.0 |
| Fecal occult positive | 1 | 16.7 | 4 | 66.7 | 5 | 83.3 | 3 | 50.0 | 10 | 55.6 | 13 | 54.2 |
| Blood fibrinogen decreased | 1 | 16.7 | 1 | 16.7 | 0 | 0.0 | 1 | 16.7 | 2 | 11.1 | 3 | 12.5 |
| White blood cells urine positive | 0 | 0.0 | 1 | 16.7 | 1 | 16.7 | 1 | 16.7 | 2 | 11.1 | 3 | 12.5 |
| Blood pressure increased | 0 | 0.0 | 0 | 0.0 | 1 | 16.7 | 1 | 16.7 | 1 | 5.6 | 2 | 8.3 |
| Basophil count increased | 1 | 16.7 | 0 | 0.0 | 0 | 0.0 | 0 | 0.0 | 1 | 5.6 | 1 | 4.2 |
| Blood pressure decreased | 1 | 16.7 | 0 | 0.0 | 0 | 0.0 | 0 | 0.0 | 1 | 5.6 | 1 | 4.2 |
| Blood bilirubin increased | 0 | 0.0 | 1 | 16.7 | 0 | 0.0 | 0 | 0.0 | 1 | 5.6 | 1 | 4.2 |
| Blood creatine phosphokinase increased | 0 | 0.0 | 1 | 16.7 | 0 | 0.0 | 0 | 0.0 | 1 | 5.6 | 1 | 4.2 |
| Total bile acids increased | 0 | 0.0 | 1 | 16.7 | 0 | 0.0 | 0 | 0.0 | 1 | 5.6 | 1 | 4.2 |
| Hepatic enzyme increased | 0 | 0.0 | 0 | 0.0 | 1 | 16.7 | 0 | 0.0 | 1 | 5.6 | 1 | 4.2 |
| **Metabolism and nutrition disorders** | 3 | 50.0 | 4 | 66.7 | 3 | 50.0 | 2 | 33.3 | 10 | 55.6 | 12 | 50.0 |

| | | | | | | | | | | | | |
|---|---|---|---|---|---|---|---|---|---|---|---|---|
| Hypertriglyceridaemia | 3 | 50.0 | 4 | 66.7 | 2 | 33.3 | 2 | 33.3 | 9 | 50.0 | 11 | 45.8 |
| Hyperphosphataemia | 0 | 0.0 | 1 | 16.7 | 0 | 0.0 | 0 | 0.0 | 1 | 5.6 | 1 | 4.2 |
| Hyponatraemia | 0 | 0.0 | 0 | 0.0 | 1 | 16.7 | 0 | 0.0 | 1 | 5.6 | 1 | 4.2 |
| Electrolyte imbalance | 0 | 0.0 | 0 | 0.0 | 1 | 16.7 | 0 | 0.0 | 1 | 5.6 | 1 | 4.2 |
| **General disorders and administration site conditions** | 0 | 0.0 | 1 | 16.7 | 6 | 100.0 | 2 | 33.3 | 7 | 38.9 | 9 | 37.5 |
| Influenza like illness | 0 | 0.0 | 0 | 0.0 | 5 | 83.3 | 2 | 33.3 | 5 | 27.8 | 7 | 29.2 |
| Pyrexia | 0 | 0.0 | 1 | 16.7 | 1 | 16.7 | 0 | 0.0 | 2 | 11.1 | 2 | 8.3 |
| **Musculoskeletal and connective tissue disorders** | 0 | 0.0 | 3 | 50.0 | 3 | 50.0 | 2 | 33.3 | 6 | 33.3 | 8 | 33.3 |
| Pain in extremity | 0 | 0.0 | 3 | 50.0 | 2 | 33.3 | 0 | 0.0 | 5 | 27.8 | 5 | 20.8 |
| Arthralgia | 0 | 0.0 | 0 | 0.0 | 1 | 16.7 | 1 | 16.7 | 1 | 5.6 | 2 | 8.3 |
| Limb discomfort | 0 | 0.0 | 0 | 0.0 | 0 | 0.0 | 1 | 16.7 | 0 | 0.0 | 1 | 4.2 |

Abbreviations: TEAE, treatment emergent adverse event.

Note: A TEAE is defined as any event not present before exposure to study drug or any event already present that worsens in severity or frequency after exposure. At each level of subject summarization, a subject is counted once if the subject reported one or more events. A subject with the most related/severe event will be counted in the "Any INS018_055", "Pooled Placebo", and "Total" column summaries. n represents the number of subjects at each level of summarization. Percentages are based on the number of subjects in the Safety Population within each column. Adverse events were coded using MedDRA version 25.1.

Insilico Medicine Restricted (Level 3)

**Table S10-2. Summary of Treatment-Emergent Adverse Events by System Organ Class and Preferred Terms -MAD *(Continued)***

| | 30 mg | | 60 mg | | 90 mg | | Placebo | | Any INS018_055 | | Total | |
|---|---|---|---|---|---|---|---|---|---|---|---|---|
| | N=6 | | N=6 | | N=6 | | N=6 | | N=18 | | N=24 | |
| | n | % | n | % | n | % | n | % | n | % | n | % |
| **Skin and subcutaneous tissue disorders** | 1 | 16.7 | 0 | 0.0 | 3 | 50.0 | 3 | 50.0 | 4 | 22.2 | 7 | 29.2 |
| Rash | 1 | 16.7 | 0 | 0.0 | 2 | 33.3 | 2 | 33.3 | 3 | 16.7 | 5 | 20.8 |
| Pruritus | 0 | 0.0 | 0 | 0.0 | 1 | 16.7 | 1 | 16.7 | 1 | 5.6 | 2 | 8.3 |
| **Nervous system disorders** | 0 | 0.0 | 2 | 33.3 | 5 | 83.3 | 0 | 0.0 | 7 | 38.9 | 7 | 29.2 |
| Dizziness | 0 | 0.0 | 0 | 0.0 | 4 | 66.7 | 0 | 0.0 | 4 | 22.2 | 4 | 16.7 |
| Headache | 0 | 0.0 | 2 | 33.3 | 1 | 16.7 | 0 | 0.0 | 3 | 16.7 | 3 | 12.5 |
| **Gastrointestinal disorders** | 0 | 0.0 | 2 | 33.3 | 2 | 33.3 | 1 | 16.7 | 4 | 22.2 | 5 | 20.8 |
| Mouth ulceration | 0 | 0.0 | 2 | 33.3 | 0 | 0.0 | 0 | 0.0 | 2 | 11.1 | 2 | 8.3 |
| Nausea | 0 | 0.0 | 0 | 0.0 | 1 | 16.7 | 1 | 16.7 | 1 | 5.6 | 2 | 8.3 |
| Abdominal distension | 0 | 0.0 | 0 | 0.0 | 1 | 16.7 | 0 | 0.0 | 1 | 5.6 | 1 | 4.2 |
| Vomiting | 0 | 0.0 | 0 | 0.0 | 1 | 16.7 | 0 | 0.0 | 1 | 5.6 | 1 | 4.2 |
| **Renal and urinary disorders** | 1 | 16.7 | 1 | 16.7 | 1 | 16.7 | 1 | 16.7 | 3 | 16.7 | 4 | 16.7 |
| Haematuria | 1 | 16.7 | 1 | 16.7 | 1 | 16.7 | 1 | 16.7 | 3 | 16.7 | 4 | 16.7 |
| **Respiratory, thoracic and mediastinal disorders** | 0 | 0.0 | 0 | 0.0 | 3 | 50.0 | 0 | 0.0 | 3 | 16.7 | 3 | 12.5 |
| Epistaxis | 0 | 0.0 | 0 | 0.0 | 2 | 33.3 | 0 | 0.0 | 2 | 11.1 | 2 | 8.3 |
| Nasal obstruction | 0 | 0.0 | 0 | 0.0 | 1 | 16.7 | 0 | 0.0 | 1 | 5.6 | 1 | 4.2 |
| **Cardiac disorders** | 1 | 16.7 | 0 | 0.0 | 0 | 0.0 | 1 | 16.7 | 1 | 5.6 | 2 | 8.3 |
| Sinus bradycardia | 1 | 16.7 | 0 | 0.0 | 0 | 0.0 | 1 | 16.7 | 1 | 5.6 | 2 | 8.3 |
| **Blood and lymphatic system disorders** | 0 | 0.0 | 0 | 0.0 | 2 | 33.3 | 0 | 0.0 | 2 | 11.1 | 2 | 8.3 |
| Anaemia | 0 | 0.0 | 0 | 0.0 | 2 | 33.3 | 0 | 0.0 | 2 | 11.1 | 2 | 8.3 |

| | | | | | | | | | | | | |
|---|---|---|---|---|---|---|---|---|---|---|---|---|
| **Hepatobiliary disorders** | 0 | 0.0 | 0 | 0.0 | 1 | 16.7 | 1 | 16.7 | 1 | 5.6 | 2 | 8.3 |
| Hyperbilirubinaemia | 0 | 0.0 | 0 | 0.0 | 1 | 16.7 | 1 | 16.7 | 1 | 5.6 | 2 | 8.3 |

Abbreviations: TEAE, treatment emergent adverse event.

Note: A TEAE is defined as any event not present before exposure to study drug or any event already present that worsens in severity or frequency after exposure. At each level of subject summarization, a subject is counted once if the subject reported one or more events. A subject with the most related/severe event will be counted in the "Any INS018_055", "Pooled Placebo", and "Total" column summaries. n represents the number of subjects at each level of summarization. Percentages are based on the number of subjects in the Safety Population within each column. Adverse events were coded using MedDRA version 25.1.

**Table S10-2. Summary of Treatment-Emergent Adverse Events by System Organ Class and Preferred Terms -MAD *(Continued)***

| | 30 mg | | 60 mg | | 90 mg | | Placebo | | Any INS018_055 | | Total | |
|---|---|---|---|---|---|---|---|---|---|---|---|---|
| | N=6 | | N=6 | | N=6 | | N=6 | | N=18 | | N=24 | |
| | n | % | n | % | n | % | n | % | n | % | n | % |
| **Infections and infestations** | 0 | 0.0 | 1 | 16.7 | 0 | 0.0 | 0 | 0.0 | 1 | 5.6 | 1 | 4.2 |
| Upper respiratory tract infection | 0 | 0.0 | 1 | 16.7 | 0 | 0.0 | 0 | 0.0 | 1 | 5.6 | 1 | 4.2 |

Abbreviations: TEAE, treatment emergent adverse event.
Note: A TEAE is defined as any event not present before exposure to study drug or any event already present that worsens in severity or frequency after exposure. At each level of subject summarization, a subject is counted once if the subject reported one or more events. A subject with the most related/severe event will be counted in the "Any INS018_055", "Pooled Placebo", and "Total" column summaries. n represents the number of subjects at each level of summarization. Percentages are based on the number of subjects in the Safety Population within each column. Adverse events were coded using MedDRA version 25.1.

References


1. Li, Y. et al. Discovery of 3,4-Dihydrobenzo[f][1,4]oxazepin-5(2H)-one Derivatives as a New Class of Selective TNIK Inhibitors and Evaluation of Their Anti-Colorectal Cancer Effects. *J Med Chem* **65**, 1786-1807 (2022).
2. Masuda, M. et al. TNIK inhibition abrogates colorectal cancer stemness. *Nat Commun* **7**, 12586 (2016).
3. Yang, B. et al. Discovery of a series of 1H-pyrrolo[2,3-b]pyridine compounds as potent TNIK inhibitors. *Bioorg Med Chem Lett* **33**, 127749 (2021).
4. Ho, K.K. et al. Discovery of 4-phenyl-2-phenylaminopyridine based TNIK inhibitors. *Bioorg Med Chem Lett* **23**, 569-573 (2013).
5. Wang, Q. et al. Identification of Phosphorylation Consensus Sequences and Endogenous Neuronal Substrates of the Psychiatric Risk Kinase TNIK. *J Pharmacol Exp Ther* **356**, 410-423 (2016).
6. Padgaonkar, A. et al. The dual CK2/TNIK inhibitor, ON108600 targets cancer stem cells and induces apoptosis of paclitaxel resistant triple-negative breast cancer cells. [abstract]. *Cancer Res* **75**, 75(15 Suppl) (2015).

7. Bujak, A. et al. Discovery of TRAF-2 and NCK-interacting kinase (TNIK) inhibitors by ligand-based virtual screening methods. *Med. Chem. Commun.* **6**, 1564-1572 (2015).
8. DaCosta Byfield, S., Major, C., Laping, N.J. & Roberts, A.B. SB-505124 is a selective inhibitor of transforming growth factor-beta type I receptors ALK4, ALK5, and ALK7. *Mol Pharmacol* **65**, 744-752 (2004).
9. Koyama, K. et al. The Tyrosine Kinase Inhibitor TAS-115 Attenuates Bleomycin-induced Lung Fibrosis in Mice. *Am J Respir Cell Mol Biol* **60**, 478-487 (2019).
10. Borza, C.M. et al. DDR1 contributes to kidney inflammation and fibrosis by promoting the phosphorylation of BCR and STAT3. *JCI Insight* **7** (2022).
11. Gan, H., McKenzie, R., Hao, Q., Idell, S. & Tang, H. Protein kinase D is increased and activated in lung epithelial cells and macrophages in idiopathic pulmonary fibrosis. *PLoS One* **9**, e101983 (2014).
12. Zhao, H. et al. Betulinic acid prevents liver fibrosis by binding Lck and suppressing Lck in HSC activation and proliferation. *J Ethnopharmacol* **296**, 115459 (2022).
13. Du, G. et al. Targeting Src family kinase member Fyn by Saracatinib attenuated liver fibrosis in vitro and in vivo. *Cell Death Dis* **11**, 118 (2020).
14. Pham, H. et al. Essential Role of Lyn in Fibrosis. *Front Physiol* **7**, 387 (2016).
15. Tzouvelekis, A. et al. Increased expression of epidermal growth factor receptor (EGF-R) in patients with different forms of lung fibrosis. *Biomed Res Int* **2013**, 654354 (2013).
16. Mukherjee, A. et al. Ionizing irradiation-induced Fgr in senescent cells mediates fibrosis. *Cell Death Discov* **7**, 349 (2021).
17. Faress, J.A. et al. Bleomycin-induced pulmonary fibrosis is attenuated by a monoclonal antibody targeting HER2. *J Appl Physiol (1985)* **103**, 2077-2083 (2007).

Insilico Medicine

# INS018_055

## Protocol Number: INS018-005-001

# A PHASE 0 SINGLE MICRODOSE STUDY TO EVALUATE THE PHARMACOKINETICS OF INS018_055 IN HEALTHY PARTICIPANTS

**Authors:** Novotech (Australia) Pty Limited

**Development Phase:** Phase 0

**Document Version:** Protocol Version 2.0 dated 27 September 2021

Property of Insilico Medicine Hong Kong Limited– Confidential

May not be used, divulged, published, or otherwise disclosed without the consent of Insilico Medicine Hong Kong Limited

# PROTOCOL AUTHORISATION

Title: A Phase 0 Single Microdose Study to Evaluate the Pharmacokinetics of INS018_055 in Healthy Participants

As Insilico Medicine Hong Kong Limited ("Sponsor") representative, I confirm that the study protocol was subjected to critical review. The information it contains is consistent with current knowledge of the risks and benefits of the investigational product (IP), as well as with the moral, ethical, and scientific principles governing clinical research as set out in the current Declaration of Helsinki (Ethical Principles for Medical Research Involving Human Subjects) and the International Council for Harmonisation (ICH) guidelines on Good Clinical Practice (GCP).

[signature] 27.09.2021

Alex Zhavoronkov Date

CEO

Insilico Medicine Hong Kong Limited

# INVESTIGATOR'S AGREEMENT

**Title:** A Phase 0 Single Microdose Study to Evaluate the Pharmacokinetics of INS018_055 in Healthy Participants

All documentation for this study that is supplied to me and that has not been previously published will be kept in the strictest confidence. This documentation includes this study protocol, Investigator's Brochure(s) (IB), electronic Case Report Forms (eCRFs), and other scientific data.

The study will not be commenced without the prior written approval of a properly constituted Human Research Ethics Committee (HREC). No changes will be made to the study protocol without the prior written approval of the Sponsor and the Ethics Committee, except where necessary to avert an immediate hazard to the participants.

I have read the protocol and agree that the study will be conducted in compliance with the protocol and in accordance with the principles of the current version of the Declaration of Helsinki (Ethical Principles for Medical Research Involving Human Subjects), and with the National Health and Medical Research Council (NHMRC) National Statement on Ethical Conduct in Human Research 2007 (updated 2018). The conduct of the study will be in accordance with the Integrated Addendum to ICH E6 (R1): Guideline for Good Clinical Practice (GCP) ICH E6 (R2), annotated with comments by the Australian Therapeutic Goods Administration (TGA; 2018).

I acknowledge that I am responsible for the overall study conduct. I agree to personally conduct or supervise the described clinical study. I agree to ensure that all associates, colleagues, and employees assisting in the conduct of the study at my site are informed about their obligations. Mechanisms are in place to ensure that site staff receive the appropriate information throughout the study.

CMAX Clinical Research Pty Ltd

Investigational Site

Angela Rowland

Printed Name of Investigator

DocuSigned by:

Signature of Investigator

Signer Name: Angela Rowland
Signing Reason: I approve this document
Signing Time: 27-Sep-2021 | 16:26 ACST
10B0A6875D77487F9B717D260F82FDF7

27-Sep-2021 | 16:26 ACST

Date

# 2. SYNOPSIS

| | |
|---|---|
| **Name of Sponsor/Company:**<br>Insilico Medicine Hong Kong Limited | |
| **Name of Investigational Product:**<br>INS018_055 | |
| **Name of Active Ingredient:**<br>INS018_055 | |
| **Protocol Number:**<br>INS018-005-001 | |
| **Title of Study:**<br>A Phase 0 Single Microdose Study to Evaluate the Pharmacokinetics of INS018_055 in Healthy Participants | |
| **Study Centre(s):**<br>CMAX Clinical Research Pty Ltd, 18a North Terrace, Adelaide, South Australia 5000 | |
| **Principal Investigator(s):**<br>Dr Angela Rowland, MBBS, FRACP | |
| **Studied Period (Years):**<br>Estimated date first participant enrolled: Q4 2021<br>Estimated date last participant completed: Q1 2022 | **Phase of Development:**<br>Phase 0 |
| **Objectives:**<br>**Primary:**<br>• To determine the pharmacokinetics (PK) of plasma INS018_055 after a single IV microdose administered to healthy participants.<br>The safety of a single dose of INS018_055 administered via IV injection in healthy adult volunteers will be monitored throughout the study. There are no specific safety endpoints for this study but safety will be monitored continuously throughout the study based on incidence and severity of adverse events, use of concomitant medications, abnormal clinically significant vital signs, physical examination, 12-lead ECG, telemetry, and laboratory tests (haematology, chemistry, and urinalysis). | |
| **Endpoints:**<br>The primary endpoints of this study are:<br>• Volume of distribution of INS018_055 in healthy volunteers (HV)<br>• Elimination half-life ($t_{1/2}$) of INS018_055 in HV<br>• Clearance of INS018_055 in HV | |
| **Methodology:**<br>This is a single centre, open-label, Phase 0 exploratory first-in-human study to determine the PK of a single dose of 100 µg INS018_055 administered IV to healthy adult volunteers. | |

This study will enrol 8 eligible adults. Safety oversight for this study will be provided by the Investigator, Sponsor's Medical Monitor (MM) and/or delegate, and an Independent MM.

The study will consist of 3 periods:

**Screening (Day -28 to Day -1)**

After the completion of informed consent, potential participants will be assessed for eligibility for the study. Participants who meet all eligibility criteria will be enrolled into the study and asked to return to the clinical research unit (CRU) on Day -1.

**Treatment Period (Day -1 to Day 2)**

Participants will be admitted to the CRU on Day -1 where they will be domiciled until Day 2 (ie, 24 hours after administration of INS018_055). On Day -1, after the confirmation of eligibility, Baseline and safety assessments will be performed.

On Day 1, participants will be administered a single dose of INS018_055 (100 µg) via IV injection and will be monitored for safety. This study will include sentinel dosing, with 1 sentinel participant being dosed at least 24 hours before the dosing of other participants in the cohort. If there are no clinically-significant safety signals up to 24 hours after dosing of the sentinel participant, as assessed by the Principal Investigator (PI), the remaining participants for the cohort will be dosed. Blood for PK analysis will be collected before and after (ie, 5, 15, and 30 minutes, and 1, 2, 4, and 8 hours) the administration of INS018_055.

On Day 2 (ie, 24 hours after INS018_055 administration) a final blood sample for PK analysis will be collected, and participants will undergo safety assessments before being discharged from the CRU.

**Follow-up (Day 8 [± 1 day])**

Participants will return to the CRU on Day 8 (± 1 day) to undergo safety assessments.

**Number of Participants (Planned):** 8

**Diagnosis and Main Criteria for Inclusion:**
**Main Inclusion Criteria**

1. Healthy female or male aged ≥ 18 and ≤ 55 years at Screening.
2. Body Mass Index (BMI) of 17.50 to 30.50 kg/m$^2$; and a total body weight > 50 kg at Screening and Day -1.
3. Sufficient venous access for the purposes of the study
4. Participants must be a non-smoker and must not have used any tobacco products within two months prior to Screening.
5. Females must be non-pregnant and non-lactating, and must use an acceptable, highly effective double contraception from Screening until study completion, including the Follow-up period. Double contraception is defined as a condom AND one other form of the following:
   a. Established hormonal contraception (with approved long-acting implantable hormones, injectable hormones). Oral contraceptive pills [OCPs] should not be used as a second

form of contraception by female participants due to the unknown potential for their reduced effectiveness when administered in combination with INS018_055.

b. A vaginal ring or an intrauterine device (IUD) (including a hormonal IUD).

c. Documented evidence of surgical sterilisation at least 6 months prior to Screening (eg, tubal occlusion, hysterectomy, bilateral salpingectomy, or bilateral oophorectomy for women or vasectomy for men [with appropriate post-vasectomy documentation of the absence of sperm in semen] provided the male partner is a sole partner).

Women not of childbearing potential must be post-menopausal for ≥ 12 months. Post-menopausal status will be confirmed through testing of follicle-stimulating hormone (FSH) levels ≥ 40 IU/L at Screening for amenorrhoeic female subjects. Females who are abstinent from heterosexual intercourse will also be eligible.

Periodic abstinence (eg, calendar, ovulation, symptothermal, post-ovulation methods) and withdrawal are not considered highly effective methods of birth control. Subject complete abstinence for the duration of the study and for 90 days after the last study treatment is acceptable.

Female subjects who are in same-sex relationships are not required to use contraception.

Women of childbearing potential (WOCBP) must have a negative pregnancy test at Screening and Day 1 and be willing to have additional pregnancy tests as required throughout the study.

Males must be surgically sterile (> 30 days since vasectomy with no viable sperm), abstinent, or if engaged in sexual relations with a WOCBP, the subject and his partner must be surgically sterile (eg, tubal occlusion, hysterectomy, bilateral salpingectomy, bilateral oophorectomy) or using an acceptable, highly effective contraceptive method from Screening until study completion, including the Follow-up period. Acceptable methods of contraception include the use of condoms and the use of an effective contraceptive for the female partner that includes: OCPs, long-acting implantable hormones, injectable hormones, a vaginal ring, or an IUD. Subjects with same-sex partners (abstinence from penile-vaginal intercourse) are eligible when this is their preferred and usual lifestyle.

Males must not donate sperm for at least 90 days after the last study treatment.

6. Willing and able to attend the trial visits and complete study assessments.
7. Willing to consume standard meals provided.
8. Able to read and understand study documents and follow Investigator and study personnel instructions during visits.
9. Signed HREC approved Informed Consent Form (ICF).

**Main Exclusion Criteria**

1. Positive toxicology screening panel (urine test including qualitative identification of barbiturates, tetrahydrocannabinol [THC], amphetamines, benzodiazepines, opiates and cocaine), or with a history of substance abuse or dependency or history of recreational IV drug use over the last 5 years (by self-declaration).

2. Positive alcohol breath test at Screening or a history of regular alcohol consumption exceeding 14 drinks/week for women or 21 drinks/week for men (1 drink = 150 mL of wine or 360 mL of beer or 45 mL of hard liquor) within the 6 months prior to Screening.
3. Major surgery or significant trauma within 28 days (4 weeks) prior to Screening.
4. Blood pressure (BP) > 150 mmHg (systolic) or > 95 mmHg (diastolic) at Screening and Day -1, following at least 5 minutes of supine rest. If BP is > 140 mmHg (systolic) or > 90 mmHg (diastolic), the BP measurements should be repeated 2 more times, at least 2 minutes apart, and the average of the 3 BP values should be used to determine the participant's eligibility.
5. Heart rate < 45 beats per minute (bpm) or > 100 bpm at Screening and Day -1, following at least 5 minutes of supine rest. If heart rate is below 45 bpm or exceeds 100 bpm, the heart rate should be repeated 2 more times, at least 2 minutes apart, and the average of the 3 heart rate values should be used to determine the participant's eligibility.
6. 12-lead ECG demonstrating QTc > 450 msec for males or > 470 msec for females, or a QRS interval ≥ 120 msec at Screening and Day -1. If QTc exceeds 450 msec (males) or 470 msec (females), or QRS exceeds 120 msec, the ECG should be repeated 2 more times, at least 2 minutes apart, and the average of the 3 QTc (or QRS) values should be used to determine the participant's eligibility.
7. ANY of the following abnormalities in clinical laboratory tests at Screening, as assessed by the study-specific laboratory and confirmed by a single repeat, if deemed necessary:
   - Serum creatinine level above the upper limit of normal (ULN) or an estimated glomerular filtration rate (GFR) value < 80 mL/min, based on the Cockcroft-Gault calculation, at Screening.
   - Aspartate aminotransferase (AST) / serum glutamic oxaloacetic transaminase (SGOT) or alanine aminotransferase (ALT) / serum glutamic pyruvic transaminase (SGPT) > 1.5 × ULN.
   - Fasting glucose > 5.4 mmol/L.
   - Total bilirubin > 1.5 × ULN.
8. A white blood cell count < 4.0 × $10^9$/L. Participants with borderline clinical laboratory values outside the reference range may be included in the study if the Investigator deems that the values are not clinically significant.
9. Absolute neutrophil count of < 2 × $10^9$/L.
10. Haematocrit below 0.4 for males and 0.35 for females.
11. Use of any investigational product (IP) or investigational medical device within 30 days prior to Screening, or 5 half-lives of the product (whichever is the longest) or participation in more than four investigational drug studies within 1 year prior to Screening
12. Use of prescription or non-prescription drugs and dietary supplements within 7 days or 5 half-lives (whichever is longer) prior to INS018_005 administration, with the exception of paracetamol, which may be used at doses of ≤ 2 g/day, and contraceptives.
13. Blood donation (excluding plasma donations) of ≥ 500 mL or significant blood loss within 56 days prior to dosing.

14. History of sensitivity to heparin or heparin-induced thrombocytopenia.
15. Other severe acute or chronic medical or psychiatric condition including recent (within the past year) or active suicidal ideation or behaviour or laboratory abnormality, or any other abnormality that in the opinion of the Investigator may increase the risk associated with study participation or IP administration or may interfere with the interpretation of study results and make the participant inappropriate for entry into this study.
16. CRU staff members directly involved in the conduct of the study and their family members, CRU members otherwise supervised by the Investigator, or participants who are Sponsor employees including their family members directly involved in the conduct of the study.
17. Will have vaccination with live virus, attenuated live virus, or any live viral components within the 6 weeks prior to the first dose of study drug or is to receive these vaccines at any time during treatment or within 8 weeks following the end of study visit. Are scheduled/intend to have a COVID-19 vaccine during the study (ie, from Screening through to Day 8).
18. Have a history of any lymphoproliferative disorder (such as Epstein Barr Virus [EBV] related lymphoproliferative disorder, as reported in some participants on other immunosuppressive drugs), history of lymphoma, leukaemia, myeloproliferative disorders, multiple myeloma, or signs and symptoms suggestive of current lymphatic disease.
19. Have a clinically significant infection currently or within 6 months of first dose of study drug (those requiring hospitalisation or parenteral antimicrobial therapy or opportunistic infections), or a history of chronic or recurrent infectious disease.
20. Are known to be infected with or test positive at Screening for human immunodeficiency virus (HIV), hepatitis B or C viruses
21. History of malignancy, except for non-melanoma skin cancer, excised more than 2 years ago, and cervical intraepithelial neoplasia that has been successfully cured more than 5 years prior to Screening.
22. Consumption of grapefruit or grapefruit juice or citrus fruits (ie, Seville oranges, pomelos, tangelos) within 7 days prior to the first dose of study medication until collection of the final pharmacokinetic blood sample.
23. History of severe allergic reactions (eg, anaphylaxis) or known sensitivity to any of the constituents of the test product.
24. Pregnant or lactating at Screening or planning to become pregnant (self or partner) at any time during the study, including the Follow-up period.
25. History of benign ethnic neutropenia.

**Investigational Product, Dosage, and Mode of Administration:**
A single dose of 100 μg INS018_055 administered via IV injection.

**Duration of Treatment:**
Participants in this study will be administered a single dose of INS018_055. This study consists of a 28-day Screening Period, a 3-day Treatment Period in which participants will be administered a single dose of INS018_055, and a 7-day Follow-up period.

**Reference Therapy, Dosage, and Mode of Administration:**
Not applicable

**Criteria for Evaluation:**
**Pharmacokinetics:**

Plasma INS018_055 PK parameters to be estimated will include $C_0$, $AUC_{(0-24)}$, $AUC_{(0-t)}$, $AUC_{(0-inf)}$, Kel, $T_{last}$, $AUC_{\%\_Extrap}$, $t_{½}$, CL, and Vz.

**Safety:**

Safety assessments will include AE collection, complete and symptom-directed physical examinations, measurement of vital signs, 12-lead ECG, telemetry, laboratory tests (haematology and chemistry), use of concomitant medications, and pregnancy testing.

**Statistical Methods:**
**Safety:**

All safety assessments, including concomitant medications, AEs, laboratory evaluations, vital signs, 12-lead ECGs, telemetry, and other safety assessments will be analysed using the Safety Population.

Adverse events will be coded using the most current version of the MedDRA® available at Novotech. A by participant AE data listing, including verbatim term, preferred term (PT), system organ class (SOC), severity and relationship to study drug, will be provided. The number of participants experiencing treatment-emergent adverse events (TEAE) and number of individual TEAEs will be summarised by SOC and PT. TEAEs will also be summarised by severity and by relationship to INS018_055. Separate summaries for severe TEAEs leading to discontinuation and deaths will be provided.

Laboratory evaluations (including haematology, chemistry, and urinalysis) will be listed and summarised by protocol-specified collection time point. Change from baseline clinical laboratory data will be summarised for each protocol-specified collection time point.

Abnormal urinalysis result (dipstick and microscopy), if applicable, will be listed only.

Vital signs (BP [systolic and diastolic], heart rate, respiratory rate, and tympanic temperature) will be listed and summarised by protocol-specified collection time point. Change from baseline will be summarised at each protocol-specified collection time point.

ECG values will be listed and summarised by clinical assessment (normal, abnormal but not clinically significant, or abnormal and clinically significant) by protocol-specified collection time point. A summary of change from baseline at each protocol-specified time point will also presented. Change from baseline for ECG clinical assessments will be presented.

The following assessments will be listed by participant: telemetry, pregnancy test/FSH test, urine drug screen / Alcohol breath test, physical examination, serology (HIV, hepatitis B and C screen) and COVID-19 screen (optional).

**Pharmacokinetics:**
The PK Population will be used for the PK analysis and the summaries and graphical presentation of all PK data.

Plasma concentrations and actual blood sampling times will be listed by treatment and protocol-specified time point and summarised using descriptive statistics — number of

measurements, arithmetic mean, SD, and %CV, geometric mean, minimum, median, and maximum — at each scheduled time point. Individual and mean plasma concentration-time profiles will also be presented graphically for each treatment.

Pharmacokinetic analysis of plasma INS018_055 will be performed by a noncompartmental method using validated Phoenix WinNonlin® software (Version 8.3 or higher, Certara, USA). Pharmacokinetic analysis will be performed on the PK Population using the actual sampling times. AUC will be calculated using the Linear Up and Log Down method. Plasma INS018_055 PK parameters to be estimated will include $C_0$, $AUC_{(0-24)}$, $AUC_{(0-t)}$, $AUC_{(0-inf)}$, Kel, $T_{last}$, $AUC_{\%\_Extrap}$, $t_{½}$, CL, and Vz.

Values for $k_{el}$, $t_{½}$, $AUC_{0-inf}$, CL or $V_z$ will not be reported for cases that do not exhibit a terminal log-linear phase (if the adjusted coefficient of determination [$R^2$] is < 0.80 or $\%AUC_{extrap}$ is > 20) in the plasma concentration versus time profile. Additional analyses will be performed as deemed necessary upon review of the data.

# TABLE OF CONTENTS


1. TITLE PAGE ........ 1
PROTOCOL AUTHORISATION ........ 2
INVESTIGATOR'S AGREEMENT ........ 3
2. SYNOPSIS ........ 4
3. TABLE OF CONTENTS, LIST OF TABLES, AND LIST OF FIGURES ........ 11
4. LIST OF ABBREVIATIONS AND DEFINITIONS OF TERMS ........ 18
5. FACILITIES AND PERSONNEL ........ 20
6. BACKGROUND AND INTRODUCTION ........ 21
6.1. Introduction ........ 21
6.2. Summary of Nonclinical and Clinical Studies ........ 21
6.2.1. Nonclinical Studies ........ 21
*6.2.1.1.* Primary Pharmacodynamics: In Vitro ........ 21
6.2.1.2. Primary Pharmacodynamics: In Vivo ........ 22
6.2.2. Clinical Studies ........ 22
6.3. Summary of Potential Risks and Benefits ........ 22
6.4. Dosage Rationale ........ 24
6.5. Participant Population ........ 26
6.6. Ethical Principles ........ 26
7. TRIAL OBJECTIVES AND ENDPOINTS ........ 27
7.1. Objectives ........ 27
7.1.1. Primary Objective ........ 27
7.2. Endpoints ........ 27
7.2.1. Primary Endpoints ........ 27
8. INVESTIGATIONAL PLAN ........ 28
8.1. Overall Study Design ........ 28
8.2. Number of Participants ........ 29
8.3. Treatment Assignment ........ 29
8.4. Safety Oversight ........ 29
8.5. Stopping Criteria ........ 29

| | | |
|---|---|---|
| 8.6. | Criteria for Study Termination | 29 |
| 9. | SELECTION AND WITHDRAWAL OF PARTICIPANTS | 34 |
| 9.1. | Participant Inclusion Criteria | 34 |
| 9.2. | Participant Exclusion Criteria | 35 |
| 9.3. | Prohibitions and Restrictions in the Study | 37 |
| 9.3.1. | Medications | 37 |
| 9.3.2. | Contraception | 37 |
| 9.3.3. | Physical Activity | 37 |
| 9.3.3.1. | Fasting | 37 |
| 9.4. | Screen Failures | 38 |
| 9.5. | Participant Replacement | 38 |
| 9.6. | Participant Withdrawal Criteria | 38 |
| 10. | TREATMENT OF PARTICIPANTS | 40 |
| 10.1. | Description of Study Drug | 40 |
| 10.1.1. | Investigational Product | 40 |
| 10.1.2. | Reference Products | 40 |
| 10.1.3. | Dosage and Treatment Period | 40 |
| 10.2. | Concomitant Medications | 41 |
| 10.3. | Treatment Compliance | 41 |
| 10.4. | Protocol Deviations | 41 |
| 10.5. | Randomisation and Blinding | 41 |
| 10.5.1. | Randomisation | 41 |
| 10.5.2. | Blinding | 41 |
| 11. | STUDY DRUG MATERIALS AND MANAGEMENT | 42 |
| 11.1. | Study Drug | 42 |
| 11.2. | Study Drug Packaging and Labelling | 42 |
| 11.3. | Study Drug Storage | 42 |
| 11.4. | Study Drug Preparation | 42 |
| 11.5. | Administration | 42 |
| 11.6. | Study Drug Accountability | 42 |
| 11.7. | Study Drug Handling and Disposal | 42 |
| 12. | STUDY SCHEDULE | 43 |
| 12.1. | Screening (Day -28 to Day -1) | 43 |

12.2. Admission to Clinic (Day -1) ....43
12.3. Day of INS018_055 Administration (Day 1) ....44
12.3.1. Before INS018_055 Administration ....44
12.3.2. INS018_055 Administration ....44
12.3.3. After INS018_055 Administration ....45
12.4. Day 2 (24 hours [±2 hours] after INS018_055 Administration) ....45
12.5. Follow-up (Day 8 [± 1 day]) ....45
12.6. Early Termination (If Applicable) ....46
13. PHARMACOKINETIC ASSESSMENTS ....47
13.1. Blood Sample Collection ....47
13.2. Sample Analysis ....47
14. ASSESSMENT OF SAFETY ....48
14.1. Safety Parameters ....48
14.1.1. Demographic/Medical and Surgical History ....48
14.1.2. Vital Signs ....48
14.1.3. Weight and Height ....48
14.1.4. Physical Examination ....48
14.1.5. Electrocardiogram ....49
14.1.6. Telemetry ....49
14.1.7. Laboratory Assessments ....49
14.1.7.1. Haematology ....49
14.1.7.2. Chemistry ....50
14.1.7.3. Urinalysis ....50
14.1.7.4. Viral Serology ....51
14.1.7.5. Urine Drug Screen ....51
14.1.7.6. Alcohol Breath Test ....51
14.1.7.7. Pregnancy Testing ....51
14.1.7.8. Follicle-stimulating Hormone Testing ....51
14.2. Adverse and Serious Adverse Events ....52
14.2.1. Definition of Adverse Events ....52
14.2.2. Severity of an Adverse Event ....53
14.2.3. Causal Relationship of an Adverse Event ....53
14.2.4. Action Taken with Investigational Product ....54

14.2.5. Outcome ....54
14.3. Definition of Serious Adverse Event ....54
14.3.1. Notification of a Serious Adverse Event ....55
14.4. Clinical Laboratory Abnormalities and Other Abnormal Assessments as Adverse Events and Serious Adverse Events ....56
14.5. Recording Adverse Events ....56
14.6. Follow-up of Adverse Events and Serious Adverse Events ....56
14.7. Pregnancy ....57
15. EXPLORATORY RESEARCH DATA (OPTIONAL) ....58
15.1. Rationale ....58
15.2. Background ....58
15.3. Assessments ....58
15.3.1. MindAge ....58
15.3.2. Blood Age ....58
15.3.2.1. Blood Chemistry ....59
15.3.2.2. Lipid Panel ....59
15.4. Analysis and Reporting ....59
16. STATISTICS ....60
16.1. Sample Size ....60
16.2. Analysis Populations ....60
16.2.1. Safety Population ....60
16.2.2. Pharmacokinetic (PK) Population ....60
16.3. Statistical Methods ....60
16.3.1. Participant Disposition ....60
16.3.2. Demographics, Medical History, and Baseline Characteristics ....60
16.3.3. Prior and Concomitant Medication ....61
16.3.4. Treatment Compliance and Exposure ....61
16.4. Safety Analyses ....61
16.4.1. Adverse Event ....61
16.4.2. Laboratory Evaluations ....61
16.4.3. Vital Signs ....61
16.4.4. Electrocardiograms ....61
16.4.5. Other Safety Assessments ....61

16.5. Pharmacokinetics....62
17. DIRECT ACCESS TO SOURCE DATA/DOCUMENTS....63
17.1. Study Monitoring....63
17.2. Data Management....63
17.3. Audits and Inspections....64
18. QUALITY CONTROL AND QUALITY ASSURANCE....65
18.1. Compliance with Good Clinical Practice....65
18.2. Archiving and Regulatory Inspection....65
19. ETHICS....66
19.1. Ethics Review....66
19.2. Ethical Conduct of the Study....66
19.3. Written Informed Consent....66
19.4. Data Protection....67
20. REGULATORY REQUIREMENTS....68
21. DATA HANDLING AND RECORDKEEPING....69
21.1. Inspection of Records....69
21.2. Retention of Records....69
21.3. Liability/Indemnity/Insurance....69
22. PUBLICATION POLICY....70
23. LIST OF REFERENCES....71

# LIST OF TABLES

Table 1: Abbreviations and Specialist Terms ..... 18
Table 2: Facilities and Personnel ..... 20
Table 3: INS018_055 Summary of Risk Management ..... 23
Table 4: Schedule of Assessments ..... 31
Table 5: Investigational Product ..... 40
Table 6: Pharmacokinetic Parameters to be Estimated ..... 62

# LIST OF FIGURES

Figure 1: Study Design....................................................................................................28

# 4. LIST OF ABBREVIATIONS AND DEFINITIONS OF TERMS

The following abbreviations and specialist terms are used in this study protocol.

**Table 1: Abbreviations and Specialist Terms**

| Abbreviation or Specialist Term | Explanation |
|---|---|
| AE | Adverse event |
| ATC | Anatomical therapeutic chemical |
| ATII | Alveolar epithelial type II |
| BILI | Bilirubin |
| BMI | Body mass index |
| BP | Blood pressure |
| COVID-19 | 2019 novel coronavirus |
| CRU | Clinical research unit |
| CS | Clinically significant |
| CTN | Clinical Trials Notification |
| EBV | Epstein Barr Virus |
| eCRF | Electronic case report form |
| ECG | Electrocardiogram |
| EOS | End of study |
| FSH | Follicle-stimulating hormone |
| GCP | Good Clinical Practice |
| GLU | Glucose |
| GFR | Globular filtration rate |
| HBsAg | Hepatitis B surface antigen |
| HCV | Hepatitis C virus |
| HDL | High density lipoprotein |
| HIV | Human Immunodeficiency Virus |
| HREC | Human Research Ethics Committee |
| HV | Healthy volunteer |
| IB | Investigator's Brochure |
| $IC_{50}$ | Half-maximal inhibitory concentration |
| ICF | Informed Consent Form |
| ICH | International Conference on Harmonisation |
| IP | Investigational product |

| Abbreviation or Specialist Term | Explanation |
|---|---|
| IPF | Idiopathic pulmonary fibrosis |
| IUD | Intrauterine device |
| LDL | Low density lipoprotein |
| LOAEL | Lowest-observed-adverse-effect level |
| MDMA | Ecstasy |
| MedDRA | Medical Dictionary for Regulatory Activities |
| MM | Medical Monitor |
| NHMRC | National Health and Medical Research Council |
| NOAEL | No-observed-adverse-effect level |
| OCP | Oral contraceptive pill |
| OTC | Over-the-counter |
| PI | Principal Investigator |
| PK | Pharmacokinetics |
| PT | Preferred term |
| PV | Pharmacovigilance |
| SAE | Serious adverse event |
| SAP | Statistical analysis plan |
| SGOT | Serum glutamic oxaloacetic transaminase |
| SOC | System organ class |
| TEAE | Treatment-emergent adverse event |
| TGA | Therapeutic Goods Administration |
| TNIK | Serine/threonine kinase Traf2- and Nck-interacting kinase |
| ULN | Upper limit of normal |
| WHO | World Health Organization |
| WOCBP | Woman of childbearing potential |
| Young.AI | Young Artificial Intelligence |

Pharmacokinetic parameters are defined in Table 6.

# 5. FACILITIES AND PERSONNEL

**Table 2: Facilities and Personnel**

| | |
|---|---|
| Sponsor | Insilico Medicine Hong Kong Limited<br>Unit 307A, Core Building 1<br>No. 1 Science Park East Avenue<br>Pak Shek Kok, New Territories, Hong Kong |
| Local Australian Sponsor | Novotech (Australia) Pty Limited<br>Level 3, 235 Pyrmont Street<br>Sydney, NSW 2009, Australia |
| Principal Investigator | Dr Angela Rowland, MBBS, FRACP<br>Clinical Research Pty Ltd<br>18a North Terrace<br>Adelaide, SA 5000, Australia |
| Local Medical Monitor | Novotech (Australia) Pty Limited<br>Level 3, 235 Pyrmont Street<br>Sydney, NSW 2009, Australia |
| Clinical Laboratories Facilities | Australian Clinical Labs (ACL)<br>1 Butler Boulevard<br>Adelaide Airport, SA 5950, Australia |
| Pharmacokinetic Laboratory | Agilex Biolabs Pty Ltd<br>28 Dalgleish Street<br>Thebarton, SA 5031 Australia |
| Biostatistical Analysis | Novotech (Australia) Pty Limited<br>Level 3, 235 Pyrmont Street<br>Sydney, NSW 2009, Australia |
| Data Management / Project Management / Monitoring | Novotech (Australia) Pty Limited<br>Level 3, 235 Pyrmont Street<br>Sydney, NSW 2009, Australia |

# 6. BACKGROUND AND INTRODUCTION

## 6.1. Introduction

INS018_055 is a highly potent and selective Traf2- and Nck-interacting kinase (TNIK) inhibitor, that is being developed by Insilico Medicine for the treatment of fibrotic diseases, particularly idiopathic pulmonary fibrosis (IPF).

IPF is a fatal lung disease, characterised by distorted lung architecture and loss of respiratory function as a result of alveolar epithelial cell injury and hyperplasia, enhanced extracellular matrix deposition, and myofibroblast activation. Activated myofibroblasts promote excessive extracellular matrix deposition resulting in characteristic fibroblast foci lesions (Myers, 1998). The occurrence and number fibroblast foci can be correlated with survival (King et al., 2001).

Fibroblast foci occur in subepithelial layers, close to areas of alveolar epithelial cell injury and repair, suggesting that impaired epithelial-mesenchymal crosstalk contributes to the pathobiology of IPF. It is generally accepted that repetitive injury and subsequent repair of alveolar epithelial type II (ATII) cells, in the presence or absence of local inflammation, represent a key pathogenic mechanism in IPF. This mechanism leads to aberrant growth factor activation and perpetuation of fibrotic transformation (Selman et al., 2001). Although several soluble mediators, such as transforming growth factor (TGF)-β1 or interleukin (IL)-1b, have a clear pathogenic role in IPF and are possible therapeutic targets (Selman et al., 2001), no pharmaceutical has been successful in clinical use to date.

Although the molecular characterisation of IPF shows a complex interdependence of various mechanisms, the Wnt pathway is consistently identified as a major activated signalling cascade that significantly contributes to pathogenesis of lung fibrosis (Chilosi et al., 2003; Morrisey, 2003; Mahmoudi et al., 2009; Yamada et al., 2017). TNIK is known to interact with TCF7L2/β-catenin transcription complex, directly and indirectly mediating an important signalling mechanism of Wnt/β-catenin pathway in its downstream part. Therefore, TNIK is a feasible target of pharmacologic intervention for manipulation of the Wnt signalling pathway. Inhibition of TNIK is expected to be potentially effective to repress Wnt-mediated signalling events.

## 6.2. Summary of Nonclinical and Clinical Studies

### 6.2.1. Nonclinical Studies

INS018_055 has undergone an extensive nonclinical safety evaluation.

All nonclinical studies conducted for INS018_055 are described in detail in the INS018_055 Investigator's Brochure (Insilico Medicine, 2021).

#### *6.2.1.1.* Primary Pharmacodynamics: In Vitro

INS018_055 showed potent inhibition of TNIK in a biochemical enzymatic assay with a half-maximal inhibitory concentration ($IC_{50}$) value of 23.5 nmol/L. Furthermore, INS018_055 inhibited few other kinases that are known to be involved in pro-fibrotic pathways ie, Alk4, DDR1, Fms, PDGFR, TGFBR1.

INS018_055 potently inhibited TGF-β induced expression of αSMA and collagen I in the lung fibroblast cell line MRC-5. In an in vitro translational pharmacology cell-based model, INS018_055 showed concentration dependent inhibition of TGF-β1-mediated αSMA expression (in fibroblast to myofibroblast transition assay) and fibronectin expression (in epithelial to mesenchymal transition assay) in cells derived from IPF patients, with no or minimal signs of toxicity or anti-proliferative side effects at the concentrations tested.

#### 6.2.1.2. Primary Pharmacodynamics: In Vivo

In an in vivo efficacy model of BLM-induced lung fibrosis, INS018_055 demonstrated potent efficacy and prevented BLM-induced lung fibrosis dose-dependently.

A pivotal IV toxicity study in mice did not reveal serious acute or delayed toxicity at single doses of 1, 3, and 10 mg/kg. Dose-dependent pharmacological effects on white blood cells were seen in male mice, consisting of moderate decreases in monocyte and basophilic lymphocyte counts one and fourteen days after injection, respectively. In light of the effects of INS018_055 on the expression and release of various cytokines, these changes in immunologically active cell populations were regarded as extended pharmacology. Since the resulting absolute and relative values remained within the normal range of white blood cell populations in CD-1 mice and were not associated with abnormal histopathological findings, they were regarded as non-adverse. At the top dose of 10 mg/kg a reduced cellularity of the white pulp of the spleen was observed in male and, less frequently, female mice, that could be related to the changes in lymphocyte populations. No histopathological changes were observed in the examined lymph nodes (inguinal and mesenteric) or any other tissue. Serum chemistry did show few incidental statistical differences between groups, without any apparent dose-dependency of clinical significance. Fourteen days after dosing, glucose levels were higher in treated groups of male mice, while females showed a tendency to lower glucose concentrations. Due to this discrepancy, it was assumed that the statistical differences in serum glucose were chance findings, not related to the study drug. In summary, INS018_055 was well tolerated after single IV injection of up to 10 mg/kg. As a conservative approach, the changes in white blood cell populations, associated with histopathological changes in spleen at 10 mg/kg, were used to define the lowest-observed adverse-effect level (LOAEL), and 3 mg/kg IV were defined as the no-observed-adverse-effect level (NOAEL) dose.

### 6.2.2. Clinical Studies

This is an exploratory first-in-human study of INS018_055. No clinical studies of INS018_055 have been conducted to date.

## 6.3. Summary of Potential Risks and Benefits

A summary of risk management is presented in Table 3.

Overall, the safety information currently known for INS018_055 is considered to have an acceptable profile for a first-in-human study. This study is conducted in healthy volunteers (HVs) and so there will be no benefit to the participants in this study.

**Table 3: INS018_055 Summary of Risk Management**

| INS018_055 Risk | Impact on Study Design |
|---|---|
| There is no human safety data for INS018_055, participants may be exposed to toxicities or experience adverse effects related to the IP. | Nonclinical safety assessments model regulatory guidance provided in ICH guideline M3(R2) on non-clinical safety studies for the conduct of human clinical trials and marketing authorisation for pharmaceuticals (ICH, 2009).<br>The study design and safety assessments model regulatory guidance to mitigate risks for a first-in-human clinical study (EMA, 2017).<br>The INS018_055 microdose administered to participants will result in extremely low plasma concentrations.<br>Participants will be domiciled at the CRU for continuous safety monitoring during the 24-hour period after INS018_055 administration.<br>Safety oversight will be provided by the Investigator, Sponsor's Medical Monitor and/or delegate, and an Independent MM (see Section 8.4). |
| INS018_055 reproductive toxicity data are absent. | Participant Inclusion Criteria mandates women of childbearing potential and fertile males must use highly effective contraception (see Section 9.1). |
| It is not known if INS018_055 is excreted in human breast milk. | Due to the potential serious adverse effect to the nursing child, breastfeeding subjects are not eligible to participant on study (see Section 9.1). |
| Human CYP450 induction evaluation data suggests that INS018_055 is an inducer of CYP1A2 at 1, 3, 10, and 30 µM, CYP2B6 at 30 µM, as well as an inducer of CYP3A4 at 3, 10, and 30 µM. | The oral contraceptive pill (OCP) should not be used as a second form of contraception by female participants, due to the unknown potential for OCP reduced effectiveness when administered in combination with INS018_055 (see Section 9.1). Hormonal OCPs are CYP3A4 substrates.<br>Participant use of prescription and non-prescription medications should be discussed with the Investigator and Sponsor's Medical Monitor and/or delegate before INS018_055 administration (see Section 10.2). Participants are not permitted to consume grapefruit or grapefruit juice or citrus fruits (ie, Seville oranges, pomelos, tangelos) within 7 days prior to INS018_055 administration until collection of the final PK blood sample (see Section 9.2). |

| INS018_055 Risk | Impact on Study Design |
|---|---|
| In the high dose treated groups, CD-1 mice administered with a single IV injection of INS018_055 showed decreased lymphocyte cellularity in the white pulp of the spleen of male and, less pronounced, female mice. | Participants with a white blood cell count $< 4.0 \times 10^9$/L will be excluded from the study (see Section 9.2).<br>Participants with a history of benign ethnic neutropenia will also be excluded from the study (see Section 9.2). |
| In a 14-day repeat non-GLP oral dose range-finding study, male mice treated with INS018_055 at a daily dose of 180 mg/kg showed a reduced erythrocyte count (19.8% reduction) and haemoglobin concentration (16.8% reduction) | Participants with haematocrit below 0.4 for males and 0.35 for females will be excluded from the study (see Section 9.2). |
| It is not known if administering approved vaccinations in combination with INS018_055 will cause adverse events and/or reduce vaccine effectiveness in study participants. | Participants planning to have vaccination with live virus, attenuated live virus, or any live viral components within the 6 weeks prior to the first dose of study drug or are to receive these vaccines at any time during treatment or within 8 weeks following completion of the end of study visit will be excluded from the study (see Section 9.2).<br>Participants who are scheduled/intend to have a COVID-19 vaccine during the study (ie, from Screening through to Day 8) will also be excluded from the study (see Section 9.2). |

## 6.4. Dosage Rationale

The study dose of 100 µg INS018_055 was calculated based on the pharmacological activity and nonclinical safety of INS018_055.

INS018_055 has been investigated in a pivotal single dose intravenous toxicity study in mice, who received doses of 1, 3, and 10 mg/kg. Compared to the planned clinical Phase 0 dose of 100 µg, ie, 2 µg/kg in a 50 kg adult, these doses are 500, 1500, and 5000-fold higher than the proposed clinical dose. In that study, INS018_055 was generally well tolerated at an individual dose of up to 10 mg/kg. At the top dose of 10 mg/kg a reduced cellularity of the white pulp of the spleen was observed in male and, less frequently, female mice 24 hours post dose, but no histopathological changes were observed in the examined lymph nodes (inguinal and mesenteric) or any other tissue. At the end of the 14-day observation period, no histopathological abnormalities were observed any more, but male mice showed a decrease in lymphocyte and white blood cell count, accompanied by a mild increase in neutrophils. Since the haematological changes in males occurred 14 days after the single injection of INS018_055, were not dose-dependent, and females showed mild changes in the opposite direction, a direct relationship with the investigational product remains questionable. As a conservative approach, the changes in white blood cell populations, associated with histopathological changes in spleen at 10 mg/kg, were used to define the LOAEL, and 3 mg/kg IV was defined as the NOAEL dose. Thus, the planned clinical dose is at least 1500-fold lower than the NOAEL in the pivotal intravenous mouse toxicity study on a mg/kg basis.

In an oral 14-day dose range-finding study in CD-1 mice, a reduced cellularity of the white pulp of the spleen was also seen in animals treated with ≥ 120 mg/kg/day, but it was not associated with changes in blood lymphocyte count. In two ongoing 28-day GLP toxicity studies in mice and beagle dogs, that are not fully analysed and reported yet, INS018_055 in oral doses up to 90 mg/kg/day (mice) and 80 mg/kg/day (dogs) was not associated with a reduction in circulating lymphocytes, neither at the end of the 28-day treatment period nor after the additional 28-day recovery period.

Furthermore, it should be taken into account that the planned human microdose study will result in extremely low plasma concentrations. Assuming a human volume of distribution ($V_D$) between 1 and 2 L/kg body weight, which is a conservative assumption in light of the $V_D$ values of approximately 3 and 8 L/kg in mice and dogs, the planned 100 µg dose of INS018_055 will lead to a hypothetical maximum concentration in a 50 kg adult between 2 and 1 ng/mL. These values are 140- and 280-fold lower than the observed maximum plasma concentration after the IV dose of 1 mg/kg in male mice, and 66- and 132-fold lower than in female mice. Thus, even if the changes in white blood cells in male mice of the pivotal IV toxicity study were related to the investigational product (IP), they would have occurred at exposures far higher than the one that could be achieved in HVs in the Phase 0 microdose study.

The pharmacologically active dose of INS018_055 has been determined in a nonclinical efficacy study in the well-established model of BLM-induced pulmonary fibrosis in mice. In that study, INS018_055 was given orally at total daily doses of 6, 20, and 60 mg/kg during a 3-week treatment study, and the lowest dose was found to show first signs of therapeutic efficacy. Since the compound was given twice daily, the lowest individual, pharmacologically active oral dose was 3 mg/kg (daily). Based on pharmacokinetic (PK) studies testing the same individual doses in mice, the absolute oral bioavailability of INS018_055 at 3 mg/kg is approximately 30%, so that the lowest pharmacological dose of 3 mg/kg orally is equivalent to an intravenous dose of 0.9 mg/kg in mice. Thus, the pharmacological dose is approximately 450-fold higher than the planned clinical dose of 2 µg/kg.

In this study, the planned dose of 100 µg, ie, 2 µg/kg in a 50 kg adult, does not exceed 1/100th of the pharmacological and NOAEL dose in mice and is, therefore, regarded as safe for first dosing in humans. The rationale and design of this study is in in accordance with the ICH guideline M3 (R2) on non-clinical safety studies for the conduct of human clinical trials and marketing authorisation for pharmaceuticals (ICH, 2009). An IP intended for oral administration may be administered as a single dose via the IV route in a Phase 0 microdose PK study (ICH, 2009). The PK parameters of clearance and distribution volume are generally determined from an IV bolus PK study, which avoids first-pass hepatic metabolism that can occur following IP administration via the oral route.

Conducting this microdose study early in the INS018_055 clinical development program will permit an initial understanding of the INS018_055 human PK parameters (ie, half life, clearance, distribution volume) that will better support the design, dose, and dosing regimen of a future Phase 1 study investigating INS018_055. In this study, the planned dose of 100 µg, i.e. 2 µg/kg in a 50 kg adult, does not exceed 1/100th of the pharmacological and NOAEL dose in mice and is, therefore, regarded as safe for first dosing in humans. These PK parameters will facilitate PK modelling of different oral INS018_055 dose regimens. PK modelling using human PK

parameters is expected to provide greater accuracy compared to PK modelling using non-clinical PK in vivo data, especially in relation to dose frequency (which primarily depends on half-life).

## 6.5. Participant Population

The study will be conducted in healthy male and female volunteers aged 18 to 55 years (inclusive at the time of Screening).

Women of childbearing potential (WOCBP) will be included and are subject to contraceptive requirements during the study from Screening until study completion, including the Follow-up period, and for at least 90 days after the last dose of study drug. WOCBP must demonstrate negative pregnancy testing at Screening and on Day -1. This is in line with regulatory Guidance on Nonclinical Safety Studies for the Conduct of Human Clinical Trials and Marketing Authorisation for Pharmaceuticals (FDA 2006).

## 6.6. Ethical Principles

This study will be conducted in accordance with the principles of the current Declaration of Helsinki (Ethical Principles for Medical Research Involving Human Subjects) and with the NHMRC National Statement on Ethical Conduct in Human Research 2007 (updated 2018). The conduct of the study will be in accordance with the ICH Integrated Addendum to E6(R1): Guideline for GCP ICH E6(R2), annotated with comments by the Australian TGA (TGA; 2018).

This study will be conducted under a protocol reviewed and approved by an HREC and investigations will be undertaken by scientifically and medically qualified persons where the benefits of the study are in proportion to the risks.

# 7. TRIAL OBJECTIVES AND ENDPOINTS

## 7.1. Objectives

### 7.1.1. Primary Objective

- To determine the PK of plasma INS018_055 after a single IV microdose administered to healthy participants.

The safety of a single dose of INS018_055 administered via IV injection in healthy adult volunteers will be monitored throughout the study. There are no specific safety endpoints for this study but safety will be monitored continuously throughout the study based on incidence and severity of AEs, use of concomitant medications, abnormal clinically significant vital signs, physical examination, 12-lead electrocardiogram (ECG), telemetry, and laboratory tests (haematology, chemistry, and urinalysis).

## 7.2. Endpoints

### 7.2.1. Primary Endpoints

The primary endpoints of this study are:

- Volume of distribution of INS018_055 in HV
- Elimination half-life ($t_{1/2}$) of INS018_055 in HV
- Clearance of INS018_055 in HV

# 8. INVESTIGATIONAL PLAN

## 8.1. Overall Study Design

This is a single centre, open-label, Phase 0 exploratory first-in-human study to determine the PK of a single dose of 100 µg INS018_055 administered IV to healthy adult volunteers.

This study will enrol 8 eligible adults. Safety oversight for this study will be provided by the Investigator, Sponsor's MM and/or delegate, and an Independent MM.

A schematic of the design is provided in Figure 1.

**Figure 1: Study Design**

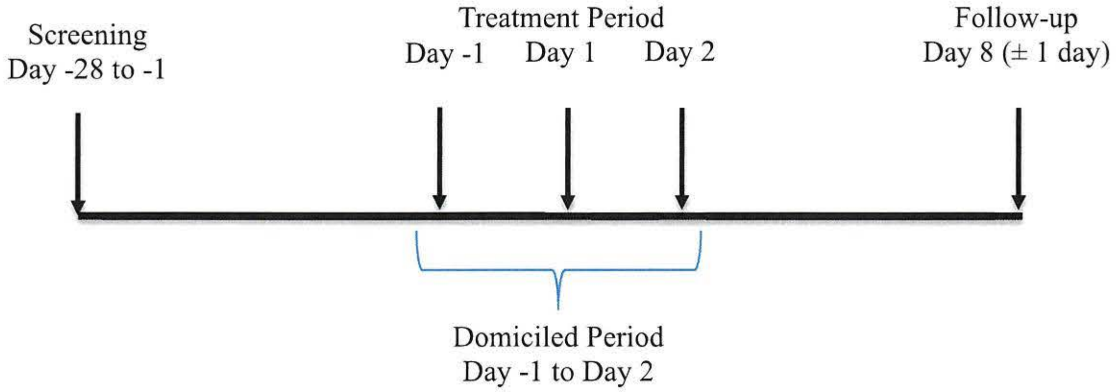


The study will consist of 3 periods:

**Screening (Day -28 to Day -1)**

After the completion of informed consent, potential participants will be assessed for eligibility for the study. Participants who meet all eligibility criteria will be enrolled into the study and asked to return to the CRU on Day -1.

**Treatment Period (Day -1 to Day 2)**

Participants will be admitted to the CRU on Day -1 where they will be domiciled until Day 2 (ie, 24 hours after administration of INS018_055). On Day -1 after the confirmation of eligibility, Baseline and safety assessments will be performed.

On Day 1, participants will be administered a single dose of INS018_055 (100 µg) via IV injection and will be monitored for safety. This study will include sentinel dosing, with 1 sentinel participant being dosed at least 24 hours before the dosing of other participants in the cohort. If there are no clinically-significant safety signals up to 24 hours after dosing of the sentinel participant, as assessed by the Principal Investigator (PI), the remaining participants for the cohort will be dosed. Blood for PK analysis will be collected before and after (ie, 5, 15, and 30 minutes, and 1, 2, 4, and 8 hours) the administration of INS018_055.

On Day 2 (ie, 24 hours after INS018_055 administration), a final blood sample for PK analysis will be collected and participants will undergo safety assessments before being discharged from the CRU.

**Follow-up (Day 8 [± 1 day])**

Participants will return to the CRU on Day 8 (± 1 day) to undergo safety assessments.

Study visits and assessments will occur as delineated in the Schedule of Assessments presented in Table 4.

## 8.2. Number of Participants

Eight (8) healthy adults will be enrolled in this study.

## 8.3. Treatment Assignment

All participants will receive a single dose of 100 µg INS018_055 via IV injection.

## 8.4. Safety Oversight

Safety oversight will be provided by the Investigator, Sponsor's MM and/or delegate, and an Independent MM.

If at any time the study is terminated, a written statement fully documenting the reasons for termination will be provided to the relevant HREC.

## 8.5. Stopping Criteria

The study may be temporarily paused or terminated should the Investigator, the MM, and the Sponsor determine that the safety of the participants is significantly jeopardised. The decision for a temporary or permanent study hold will depend on the nature, frequency and severity of AEs that were observed in all enrolled participants to date. In a temporary study hold, no additional participants will be enrolled into the study or be administered INS018_055 until the study team members (including the PI, MM, and Sponsor) decide it is safe to proceed with the study.

## 8.6. Criteria for Study Termination

The study will be completed as planned unless:

- New information or other evaluation regarding the safety of the study medication indicates a change in the known risk/benefit profile for the compound, such that the risk/benefit is no longer acceptable for participants participating in the study. This may be determined by the Sponsor, the Investigator, the HREC or regulatory authorities.
- The study is terminated by the Sponsor for administrative reasons.

The Sponsor, Investigator, and the HREC reserve the right to terminate or suspend the study at any time; however, this should be discussed between the relevant parties beforehand and the reason for such decision recorded. Should this occur, all data available will also be recorded in the eCRFs. If the Sponsor, the HREC, or regulatory authority elects to terminate or suspend the study or the participation of the investigational site, a study-specific procedure for early

termination or suspension will be provided by the Sponsor. The procedure will be followed by the investigational site during termination or study suspension.

The Investigator should notify the relevant HREC in writing of the study's completion or early discontinuation.

**Table 4: Schedule of Assessments**

| | Screening | | Treatment | | | | | | | | | | Follow-up |
|---|---|---|---|---|---|---|---|---|---|---|---|---|---|
| **Study Day** | **Day -28 to -1** | **Day -1[1]** | **Day 1[2]** | | | | | | | | | **Day 2[2]** | **Day 8 (± 1 day) /EOS[2, 3]** |
| **Hours Post Dose (Window)** | **NA** | **NA** | **Pre-dose** | **0** | **5 min** | **15 min** | **30 min** | **1 h** | **2 h** | **4 h** | **8 h** | **24 h (±2 h)** | **168 h (± 1 day)** |
| Informed consent | X | | | | | | | | | | | | |
| Admission to CRU[4] | | X | | | | | | | | | | | |
| Discharge from the CRU[4] | | | | | | | | | | | | X | |
| Outpatient Visit | X | | | | | | | | | | | | X |
| Inclusion/Exclusion Criteria | X | X | | | | | | | | | | | |
| Demographics[5] | X | | | | | | | | | | | | |
| Drug/Alcohol/Tobacco History | X | X | | | | | | | | | | | |
| Medical/Surgical History | X | X | | | | | | | | | | | |
| Prior Medications[6] | X | X | X | | | | | | | | | | |
| Complete Physical Examination[7] | X | | | | | | | | | | | | X |
| Symptom-directed Physical Examination | | ◄ | — | — | — | — | — | — | — | — | — | ► | |
| Body Weight (kg) | X | X | | | | | | | | | | | |
| Height (cm) | X | | | | | | | | | | | | |
| Pregnancy Test[8] for WOCBP only | X | X | | | | | | | | | | | X |
| FSH Test[9] (if applicable) | X | | | | | | | | | | | | |
| Serology[10] | X | | | | | | | | | | | | |

| | **Screening** | | **Treatment** | | | | | | | | | | **Follow-up** |
|---|---|---|---|---|---|---|---|---|---|---|---|---|---|
| **Study Day** | **Day -28 to -1** | **Day -1**[1] | **Day 1**[2] | | | | | | | | | **Day 2**[2] | **Day 8 (± 1 day) /EOS**[2, 3] |
| **Hours Post Dose (Window)** | **NA** | **NA** | **Pre-dose** | **0** | **5 min** | **15 min** | **30 min** | **1 h** | **2 h** | **4 h** | **8 h** | **24 h (±2 h)** | **168 h (± 1 day)** |
| Haematology and chemistry tests[11] | X | X | | | | | | | | | | X | X |
| Urinalysis[11] | X | X | | | | | | | | | | X | X |
| Urine Drug Test[12] | X | X | | | | | | | | | | | |
| Alcohol Breath Test | X | X | | | | | | | | | | | |
| 12-lead ECG[13] | X | X | X | X | | X | | X | | | | X | X |
| Telemetry[14] | | | ←→ | | | | | | | | | | |
| Vital signs[15] | X | X | X | X | | X | | X | | X | X | X | X |
| INS018_055 administration[16] | | | | X | | | | | | | | | |
| PK Blood Sampling[17] | | | X | X | X | X | X | X | X | X | X | X | |
| Adverse Events[18] | X | X | ←→ | | | | | | | | | X | X |
| Concomitant Medication[6] | | | ←→ | | | | | | | | | X | X |
| Exploratory research data[19] | | X | | | | | | | | | | | X |

Abbreviations: AE = Adverse event; CRU = clinical research unit; ECG = electrocardiograph; EOS=end of study; FSH = Follicle-stimulating hormone; h = hours; HIV = Human immunodeficiency virus; min = minutes; NA = not applicable; PK = pharmacokinetic; WOCBP = women of childbearing potential.

1 If Screening occurs on Day -1, identical assessments for Screening and Day-1 do not need to be repeated after the participant is admitted to the CRU.

2 Additional safety tests (eg, haematology, chemistry, urinalysis, 12-lead ECG, etc.) may be performed whenever clinically indicated, at the Investigator's discretion.

3 The study assessments as described at Day 8 are also the assessments for the early termination visit.

4 Participants will reside at the CRU until 24 hours after INS018_055 administration.

5 Includes date of birth, age (calculated), sex, ethnicity, and race.

[6] The use of prior medication from 30 days prior to Screening up to Day -1 will be documented. The use of all concomitant medication will be documented from Day 1 through to Day 8.

[7] Complete physical examination includes: general appearance, head, eyes, ears, nose, throat, mouth, neck, thyroid, lymph nodes, cardiovascular system, respiratory system, gastrointestinal system, renal system, central and peripheral nervous system, musculoskeletal system, dermatologic system, other.

[8] A serum pregnancy test will be performed at the Screening visit and a urine pregnancy test will be performed on Day -1 and Day 8 (EOS) visits for WOCBP only.

[9] Postmenopausal status will be confirmed through testing of FSH levels ≥ 40 IU/L at Screening.

[10] Participant must not have a history of positive HIV status or positive HIV status at Screening. Participant must have hepatitis B and C documented as negative for study inclusion.

[11] Blood and urine will be collected for safety laboratory testing which will include haematology, chemistry, and urinalysis. Participants must fast for 8 hours prior to haematology and chemistry testing.

[12] Urine drug screen will include: tetrahydrocannabinol, cocaine, amphetamines, barbiturates, benzodiazepines, opiates, methadone, methamphetamines, ecstasy (MDMA), phencyclidine, and cotinine.

[13] ECGs will be collected at pre-dose (within 60 minutes prior to dosing), and 0, and 15 minutes (±5 minutes), and 1 (±30 minutes), 24 (±2 hours ) and 168 hours (± 1 day) after INS018_055 administration. A standard 12-lead ECG will be recorded after 5 minutes in a supine position. If any abnormality is detected on the single trace, 2 additional traces, at least 2 minutes apart, will be performed for Investigator (or designee) review. When the time of ECG monitoring coincides with a blood draw, the ECG will be taken before the scheduled blood draw while ensuring the blood draw is within the window specified in the protocol. Where multiple assessments are scheduled for the same timepoint, the order of assessments will be 12-lead ECG, vital signs, blood draw, ensuring the blood draw is within the window specified.

[14] To establish a baseline, telemetry should be recorded for at least 2 hours before dosing. This may be done -2 hours immediately prior to dosing or at some 2-hour continuous interval in the 24 hours prior to dosing, as long as the recording is performed when the participant is awake. Continuous cardiac monitoring will be conducted 15 minutes pre-dose through the 8-hour post dose period.

[15] Vital signs will be collected at pre-dose (within 60 minutes prior to dosing), 0 and 15 minutes (±5 minutes), and 1 hour (±30 minutes), 4 hours (±45 minutes), 8 hours (±1 hour), 24 hours (±2 hours), and 168 hours (± 1 day) after INS018_055 administration. Vital signs will include body temperature, systolic and diastolic blood pressures, heart rate, and respiration rate. Blood pressure and heart rate will be measured in participants resting for at least 5 minutes in a supine position. When the time of vital signs measurement coincides with a blood draw, the vital signs will be taken before the scheduled blood draw where possible, ensuring the blood draw is within the window specified in the protocol. Where multiple assessments are scheduled for the same timepoint, the order of assessments will be 12-lead ECG, vital signs, blood draw, ensuring the blood draw is within the window specified. Assessment of body temperature by the tympanic route.

[16] A single 100 μg dose of INS018_055 will be administered via IV injection. This study will include sentinel dosing, with 1 sentinel participant being dosed at least 24 hours before the dosing of other participants in the cohort. If there are no clinically significant safety signals up to 24 hours after dosing of the sentinel participant, as assessed by the Principal Investigator, the remaining participants for the cohort will be dosed.

[17] Blood samples for PK will be collected at pre-dose (within 30 minutes prior to dosing), and 0, 5 minutes (±3 minutes), 15 minutes (±5 minutes), and 30 minutes (±10 minutes), and 1 hour (±10 minutes), 2 hours (±10 minutes), 4 hours (±10 minutes), 8 hours (±10 minutes), and 24 hours (±2 hours) after INS018_055 administration.

[18] All AEs will be documented from the time of consent to Day 8.

[19] Exploratory research data (optional) will include participant's completing a survey for MindAge, and blood sampling for the assessment of Blood Age (Blood Chemistry and Lipid Panel). Participants will fast for at least 8 hours prior to blood draw for lipid panel testing which will include cholesterol, triglycerides, high density lipoprotein (HDL), and low density lipoprotein (LDL).

# 9. SELECTION AND WITHDRAWAL OF PARTICIPANTS

## 9.1. Participant Inclusion Criteria

To be eligible for this study, a participant has to meet ***all*** of the following inclusion criteria:

1. Healthy[1] female or male aged ≥ 18 and ≤ 55 years at Screening.
2. BMI of 17.50 to 30.50 kg/m$^2$; and a total body weight > 50 kg at Screening and Day -1.
3. Sufficient venous access for the purposes of the study.
4. Participants must be a non-smoker and must not have used any tobacco products within two months prior to Screening.
5. Females must be non-pregnant and non-lactating, and must use an acceptable, highly effective double contraception from Screening until study completion, including the Follow-up period. Double contraception is defined as a condom AND one other form of the following:

    a. Established hormonal contraception (with approved long-acting implantable hormones, injectable hormones). Oral contraceptive pills [OCPs] should not be used as a second form of contraception by female participants, due to the unknown potential for OCP reduced effectiveness when administered in combination with INS018_055.

    b. A vaginal ring or an intrauterine device (IUD) (including a hormonal IUD).

    c. Documented evidence of surgical sterilisation at least 6 months prior to Screening (eg, tubal occlusion, hysterectomy, bilateral salpingectomy, or bilateral oophorectomy for women or vasectomy for men [with appropriate post-vasectomy documentation of the absence of sperm in semen] provided the male partner is a sole partner).

    Women not of childbearing potential must be post-menopausal for ≥ 12 months. Post-menopausal status will be confirmed through testing of FSH levels ≥ 40 IU/L at Screening for amenorrhoeic female subjects. Females who are abstinent from heterosexual intercourse will also be eligible.

    Periodic abstinence (eg, calendar, ovulation, symptothermal, post-ovulation methods) and withdrawal are not considered highly effective methods of birth control. Subject complete abstinence for the duration of the study and for 90 days after the last study treatment is acceptable.

    Female subjects who are in same-sex relationships are not required to use contraception.

    WOCBP must have a negative pregnancy test at Screening and Day 1 and be willing to have additional pregnancy tests as required throughout the study.

[1] Healthy is defined as no clinically relevant abnormalities identified by a detailed medical history, full physical examination, including blood pressure and pulse rate measurement, 12-lead ECG, and clinical laboratory tests.

Males must be surgically sterile (> 30 days since vasectomy with no viable sperm), abstinent, or if engaged in sexual relations with a WOCBP, the subject and his partner must be surgically sterile (eg, tubal occlusion, hysterectomy, bilateral salpingectomy, bilateral oophorectomy) or using an acceptable, highly effective contraceptive method from Screening until study completion, including the Follow-up period. Acceptable methods of contraception include the use of condoms and the use of an effective contraceptive for the female partner that includes: OCPs, long-acting implantable hormones, injectable hormones, a vaginal ring, or an IUD. Subjects with same-sex partners (abstinence from penile-vaginal intercourse) are eligible when this is their preferred and usual lifestyle.

Males must not donate sperm for at least 90 days after the last study treatment.

6. Willing and able to attend the trial visits and complete study assessments.
7. Willing to consume standard meals provided.
8. Able to read and understand study documents and follow Investigator and study personnel instructions during visits.
9. Signed HREC approved Informed Consent Form (ICF).

## 9.2. Participant Exclusion Criteria

A participant who meets ***any*** of the following exclusion criteria must be excluded from the study:

1. Positive toxicology screening panel (urine test including qualitative identification of barbiturates, tetrahydrocannabinol [THC], amphetamines, benzodiazepines, opiates and cocaine), or with a history of substance abuse or dependency or history of recreational IV drug use over the last 5 years (by self-declaration).
2. Positive alcohol breath test at Screening or a history of regular alcohol consumption exceeding 14 drinks/week for women or 21 drinks/week for men (1 drink = 150 mL of wine or 360 mL of beer or 45 mL of hard liquor) within the 6 months prior to Screening.
3. Major surgery or significant trauma within 28 days (4 weeks) prior to Screening.
4. BP > 150 mmHg (systolic) or > 95 mmHg (diastolic) at Screening and Day -1, following at least 5 minutes of supine rest. If BP is > 140 mmHg (systolic) or > 90 mmHg (diastolic), the BP measurements should be repeated 2 more times, at least 2 minutes apart, and the average of the 3 BP values should be used to determine the participant's eligibility.
5. Heart rate < 45 beats per minute (bpm) or > 100 bpm at Screening and Day -1, following at least 5 minutes of supine rest. If heart rate is below 45 bpm or exceeds 100 bpm, the heart rate should be repeated 2 more times, at least 2 minutes apart, and the average of the 3 heart rate values should be used to determine the participant's eligibility.
6. 12-lead ECG demonstrating QTc > 450 msec for males or > 470 msec for females, or a QRS interval ≥ 120 msec at Screening and Day -1. If QTc exceeds 450 msec (males) or 470 msec (females), or QRS exceeds 120 msec, the ECG should be repeated 2 more

times, at least 2 minutes apart, and the average of the 3 QTc (or QRS) values should be used to determine the participant's eligibility.

7. ANY of the following abnormalities in clinical laboratory tests at Screening, as assessed by the study-specific laboratory and confirmed by a single repeat, if deemed necessary:
   - Serum creatinine level above the upper limit of normal (ULN) or an estimated GFR value < 80 mL/min, based on the Cockcroft-Gault calculation, at Screening.
   - Aspartate aminotransferase (AST) / serum glutamic oxaloacetic transaminase (SGOT) or alanine aminotransferase (ALT) / serum glutamic pyruvic transaminase (SGPT) > 1.5 × ULN.
   - Fasting glucose > 5.4 mmol/L.
   - Total bilirubin > 1.5 × ULN.
8. A white blood cell count < 4.0 × $10^9$/L. Participants with borderline clinical laboratory values outside the reference range may be included in the study if the Investigator deems that the values are not clinically significant.
9. Absolute neutrophil count of < 2 × $10^9$/L.
10. Haematocrit below 0.4 for males and 0.35 for females.
11. Use of any IP or investigational medical device within 30 days prior to Screening, or 5 half-lives of the product (whichever is the longest) or participation in more than four investigational drug studies within 1 year prior to Screening.
12. Use of prescription or non-prescription drugs and dietary supplements within 7 days or 5 half-lives (whichever is longer) prior to INS018_005 administration, with the exception of paracetamol, which may be used at doses of ≤ 2 g/day, and contraceptives.
13. Blood donation (excluding plasma donations) of ≥ 500 mL or significant blood loss within 56 days prior to dosing.
14. History of sensitivity to heparin or heparin-induced thrombocytopenia.
15. Other severe acute or chronic medical or psychiatric condition including recent (within the past year) or active suicidal ideation or behaviour or laboratory abnormality, or any other abnormality that in the opinion of the Investigator may increase the risk associated with study participation or IP administration or may interfere with the interpretation of study results and make the participant inappropriate for entry into this study.
16. CRU staff members directly involved in the conduct of the study and their family members, CRU members otherwise supervised by the Investigator, or participants who are Sponsor employees including their family members directly involved in the conduct of the study.
17. Will have vaccination with live virus, attenuated live virus, or any live viral components within the 6 weeks prior to the first dose of study drug or is to receive these vaccines at any time during treatment or within 8 weeks following the end of study visit. Are scheduled/intend to have a COVID-19 vaccine during the study (ie, from Screening through to Day 8).

18. Have a history of any lymphoproliferative disorder (such as EBV related lymphoproliferative disorder, as reported in some participants on other immunosuppressive drugs), history of lymphoma, leukaemia, myeloproliferative disorders, multiple myeloma, or signs and symptoms suggestive of current lymphatic disease.
19. Have a clinically significant infection currently or within 6 months of first dose of study drug (those requiring hospitalisation or parenteral antimicrobial therapy or opportunistic infections), or a history of chronic or recurrent infectious disease.
20. Are known to be infected with or test positive at Screening for HIV, hepatitis B or C viruses.
21. History of malignancy, except for non-melanoma skin cancer, excised more than 2 years ago, and cervical intraepithelial neoplasia that has been successfully cured more than 5 years prior to Screening.
22. Consumption of grapefruit or grapefruit juice or citrus fruits (ie, Seville oranges, pomelos, tangelos) within 7 days prior to the first dose of study medication until collection of the final pharmacokinetic blood sample.
23. History of severe allergic reactions (eg, anaphylaxis) or known sensitivity to any of the constituents of the test product.
24. Pregnant or lactating at Screening or planning to become pregnant (self or partner) at any time during the study, including the Follow-up period.
25. History of benign ethnic neutropenia.

## 9.3. Prohibitions and Restrictions in the Study

### 9.3.1. Medications

Use of prescription or non-prescription drugs and dietary supplements within 7 days or 5 half-lives (whichever is longer) prior to INS018_005 administration, with the exception of paracetamol, which may be used at doses of ≤ 2 g/day, and contraceptives.

### 9.3.2. Contraception

Contraceptive requirements are described in Section 9.1.

### 9.3.3. Physical Activity

Strenuous exercise is not permitted during the Treatment Period of this study.

#### 9.3.3.1. Fasting

All participants must fast for at least 8 hours prior to blood draws for safety haematology and chemistry tests, and Blood Age (optional), at Screening, Day -1, Day 2, and Day 8. Water is permitted *ad libitum* during this time.

## 9.4. Screen Failures

Screen failures are defined as participants who consent to participate in the clinical study but are not subsequently enrolled in the study. A minimal set of screen failure information is required to ensure transparent reporting of screen failure participants to meet the Consolidated Standards of Reporting Trials publishing requirements and to respond to queries from regulatory authorities. Minimal information includes screen failure details, eligibility criteria, and any serious adverse event (SAE). Individuals who do not meet the criteria for participation in this study (screen failure) may be re-screened. Re-Screening will be allowed within the recruitment period for the study. Re-screened participants should be assigned a new participant number.

## 9.5. Participant Replacement

Participants who are enrolled but who do not receive any dose of study drug will be replaced and the replacement participants will be assigned the same treatment sequence as the participants they are replacing.

Participants who discontinue the study prior to completion of dosing may be replaced (at the discretion of the Sponsor in communication with the PI).

## 9.6. Participant Withdrawal Criteria

Participants may withdraw their consent to participate in the study at any time. If a participant withdraws consent, the date and reason for consent withdrawal should be documented. Participants will be encouraged to remain in the clinic to complete all necessary assessments and until the Investigator deems that it is safe to be discharged. Participant data will be included in the analysis up to the date of the withdrawal of consent.

Apart from withdrawal of consent, reasons for early termination of individual participants can include:

- Protocol deviations or participant non-compliance (must be specified on the appropriate eCRF)
- Serious or severe AEs
- Administrative decision by the Investigator or the Sponsor
- Death
- Other (must be specified)

The primary reason for withdrawal will be identified and recorded on the appropriate eCRF, along with the date of withdrawal.

In accordance with applicable regulations, a participant has the right to withdraw from the study, at any time and for any reason, without prejudice to future medical care.

If a participant is withdrawn because of an AE, the Investigator must arrange for the participant to have appropriate follow-up care until the AE is resolved or has stabilised. Unresolved AEs will be followed until the last scheduled Follow-up/End of Study (EOS) visit or until the PI and MM determine that further follow-up is no longer indicated. In addition to AEs, other reasons for removal of participants from the study might include, but are not limited to, withdrawal of

consent, administrative decision by the Investigator or the Sponsor, protocol deviation, or participant noncompliance.

If a participant asks or decides to withdraw from the study, all efforts will be made to complete and report the observations, especially those related to the listed primary and secondary objectives, as thoroughly as possible up to the date of withdrawal. Wherever possible, the tests and evaluations, including those listed for the EOS/Follow-up visit, should be performed for all participants who discontinue prior to the completion of the study.

# 10. TREATMENT OF PARTICIPANTS

## 10.1. Description of Study Drug

### 10.1.1. Investigational Product

INS018_055 is a Type I kinase inhibitor. INS018_055 acetate salt was selected for further development due to its solubility in water and chemical and physical stability at different temperatures.

**Table 5: Investigational Product**

| | **Investigational Product** |
|---|---|
| **Product Name:** | INS018_055 |
| **IUPAC Chemical Name** | 2-[4-(4-fluorophenyl)-1-(prop-2-yl)imidazol-5-yl]-*N*-[4-(4-methylhexahydropyrazin-1-yl)phenyl]-1*H*-imidazole-4-carboxamide |
| **Unit Dose** | 100 µg |
| **Route of Administration** | IV injection |
| **Physical Description** | Clear, aqueous solution |
| **Dosing Instructions** | INS018_055 is to be administered as an IV bolus injection as a slow and constant push which should not exceed 2 minutes. Please refer to the Pharmacy Manual. |
| **Manufacturer** | Shanghai STA Pharmaceutical Product Co., Ltd. |

INS018_055 Injection is formulated for IV administration as a sterile product in 9.0 mg/mL sodium chloride, 1.0 mg/mL monothioglycerol, 0.48 mg/mL citric acid monohydrate, 0.80 mg/mL tri-sodium citrate dihydrate, and water for injection (pH 4.5). The injection is supplied for clinical use in a 2 mL vial containing an INS018_055 concentration of 50 µg/mL. No preservative is used as the vial is designed for single use.

The product is supplied in single-use 2R clear vials with coated stoppers and aluminium crimp seals with "flip-off" caps.

### 10.1.2. Reference Products

No reference product will be used in this study.

### 10.1.3. Dosage and Treatment Period

Participants will be administered a single 100 µg dose of INS018_055 via IV injection on Day 1. This study will include sentinel dosing, with 1 sentinel participant being dosed at least 24 hours before the dosing of other participants in the cohort. If there are no clinically-significant safety signals up to 24 hours after dosing of the sentinel participant, as assessed by the PI, the remaining participants for the cohort will be dosed.

## 10.2. Concomitant Medications

All medications, including over-the-counter (OTC) medications, vitamins, and herbal supplements, taken during the 30 days prior to the first study drug administration will be recorded and reviewed by the Investigator to determine whether the participant is suitable for inclusion in the study.

All medications taken during the Treatment Period (Day -1 to Day 2) and up to 8 days after the INS018_055 administration will be regarded as concomitant medication.

Medications taken before and stopped prior to the first study treatment will be treated as prior medications.

The use of any IP or investigational medical device within 30 days prior to Screening, or 5 half-lives of the product (whichever is longest) is prohibited. Additional restrictions relating to concomitant medication use are outlined in Section 9.3.1.

Prior therapy or concomitant therapy (after study drug administration) with any medications, including both prescription and non-prescription drugs, should be discussed with the Investigator and Sponsor's MM and/or delegate before study drug administration, except in the case of necessary treatment of AEs or where appropriate medical care necessitates that therapy should begin before the Investigator can consult with the Independent MM.

Paracetamol/acetaminophen ($\leq$ 2 g/day) may be used for minor ailments during the course of the study, at the discretion of the Investigator, without prior consultation with Sponsor's MM and/or delegate.

## 10.3. Treatment Compliance

A single dose of INS018_055 will be administered at the CRU by the Investigator or their designee.

## 10.4. Protocol Deviations

Should any protocol deviation occur, it must be reported to the study monitor as soon as is reasonably practical. The deviation and the reason for its occurrence must be documented, reported to the relevant HREC (if required), and included in the clinical study report. Protocol deviations will be classified as important or not important.

## 10.5. Randomisation and Blinding

### 10.5.1. Randomisation

Randomisation will not be used in this study.

### 10.5.2. Blinding

This is an open-label study and blinding will not be used.

# 11. STUDY DRUG MATERIALS AND MANAGEMENT

## 11.1. Study Drug

The Sponsor will supply the INS018_055 to the CRU. The study drug provided for this study was manufactured under current Good Manufacturing Practices and will be suitable for human use.

## 11.2. Study Drug Packaging and Labelling

The Sponsor is responsible for the preparation and labelling and providing details of batch numbers, safety, and stability data.

The study drug will be labelled in accordance with local regulatory requirements and will be shipped at a temperature of 2°C to 8°C.

## 11.3. Study Drug Storage

Upon receipt, the study drug must be stored at 2°C to 8°C.

The Investigator or designee will be fully responsible for the security, accessibility, and storage of the study drug while it is at the investigational facility.

## 11.4. Study Drug Preparation

Procedures relating to study drug preparation and dispensing are outlined in the Pharmacy Manual.

## 11.5. Administration

The Investigator or designee will administer the study drug to the participant.

## 11.6. Study Drug Accountability

A record will be maintained by the CRU that will account for all dispensing and return of any used and unused study drug. At the end of the study, the study drug will be reconciled, and a copy of the record given to the study monitor.

## 11.7. Study Drug Handling and Disposal

On completion of the study, any surplus study drug supplies will be destroyed upon receipt of written approval from the Sponsor. Evidence of the destruction of any surplus study drug will be supplied to the study monitor. If no supplies remain, this will be documented in the dispensing record.

# 12. STUDY SCHEDULE

A Schedule of Assessments is provided in Table 4.

Where possible, assessments should be conducted in order of least invasive to most invasive.

## 12.1. Screening (Day -28 to Day -1)

Prior to enrolling in the study, and before performance of any procedures, potential participants will attend a Screening session in a fasted state (ie, ≥ 8 hours fast) at which time they will be provided with full information concerning details of the study assessments and procedures. They will also be provided with an ICF. Prior to being asked to sign the consent form, participants will be given time to review study information and ask any questions.

After the consent form is signed, screening assessments will be carried out as follows:

- Review of the Inclusion/Exclusion criteria
- Documentation of demographics
- Documentation of drug, alcohol, tobacco, and medical/surgical history
- Documentation of use of prior medications
- Complete physical examination
- Measurement of body weight and height
- Serum pregnancy test for WOCBP only
- Blood draw for FSH test for post-menopausal women only
- Blood draw for serology testing
- Blood draw for safety haematology and chemistry tests
- Urine collection for urinalysis
- Urine collection for drug screen
- Alcohol Breath Test
- 12-lead ECG
- Measurement of vital signs
- Documentation of AEs

## 12.2. Admission to Clinic (Day -1)

Participants will return to the CRU in a fasted state (≥ 8 hours fast) where they will be domiciled until Day 2 (ie, 24 hours after INS018_055 administration). The following assessments will be performed unless Screening occurs on Day -1 and the assessment is identical to the Screening assessment:

- Confirmation of eligibility
- Review of drug, alcohol, tobacco, and medical/surgical history

- Alcohol breath test
- Review of use of prior medications
- Symptom-directed physical examination
- Body weight
- Urine pregnancy test for WOCBP only
- Blood draw for safety haematology and chemistry tests
- Urine collection for urinalysis
- Urine collection for drug screen
- 12-lead ECG
- Measurement of vital signs
- Review and documentation of AEs
- MindAge Survey (optional)
- Blood draw for Blood Age (optional)

## 12.3. Day of INS018_055 Administration (Day 1)

### 12.3.1. Before INS018_055 Administration

Before INS018_055 administration on Day 1, the following assessments will be performed:

- Review of use of prior medications
- Symptom-directed physical examination
- 12-lead ECG (within 60 minutes prior to dosing)
- Measurement of vital signs (within 60 minutes prior to dosing)
- Telemetry (should be recorded for at least 2 hours prior to INS018_055 administration and continue until 8 hours post INS018_055 administration)
- Blood sample for PK analysis (within 30 minutes prior to dosing)
- Review and documentation of AEs and concomitant medication

Participants will remain in the CRU through to the completion of all scheduled procedures on Day 2.

### 12.3.2. INS018_055 Administration

After the completion of all pre-dosing assessments, participants will be administered a single 100 μg dose of INS018_055 via IV injection, which will be administered by assigned clinical staff.

#### 12.3.3. After INS018_055 Administration

The following procedures will be conducted after the completion of INS018_055 administration:

- Symptom-directed physical examination
- 12-lead ECG at 0, and 15 minutes (±5 minutes), and 1 hour (±30 minutes) after INS018_055 administration
- Continuous telemetry until 8 hours post INS018_055 administration
- Measurement of vital signs 0, and 15 minutes (±5 minutes), 1 hour (±30 minutes), 4 hours (±45 minutes) and 8 hours (±1 hour) post INS018_055 administration
- Blood sample for PK analysis at timepoints 0, 5 minutes (±3 minutes), 15 minutes (±5 minutes), and 30 minutes (±10 minutes), and 1 hour (±10 minutes), 2 hours (±10 minutes), 4 hours (±10 minutes), and 8 hours (±10 minutes) post INS018_055 administration
- Review and documentation of AEs and concomitant medications

### 12.4. Day 2 (24 hours [±2 hours] after INS018_055 Administration)

The following procedures will be conducted 24 hours after the administration of INS018_055. Participants are to have fasted for ≥ 8 hours before the following assessments are commenced:

- Symptom-directed physical examination
- 12-lead ECG
- Measurement of vital signs 24 hours post INS018_055 administration
- Blood draw for safety haematology and chemistry tests
- Urine sample for urinalysis
- Blood sample for PK analysis at 24 hours (± 2 hours) post INS018_055 administration
- Review and documentation of AEs and concomitant medications

At the completion of all study procedures, participants will be discharged from the CRU at the discretion of the Investigator.

## 12.5. Follow-up (Day 8 [± 1 day])

Participants will return to the CRU in a fasted state (ie, ≥ 8 hours fast) for a Follow-up visit on Day 8 (± 1 day).

The following procedures will be conducted at the Follow-up visit:

- Complete physical examination
- Urine pregnancy test for WOCBP only
- Blood draw for safety haematology and chemistry tests
- Urine collection for urinalysis

- 12-lead ECG
- Measurement of vital signs
- Review and documentation of AEs and concomitant medications
- MindAge Survey (optional)
- Blood draw for Blood Age (optional)

This visit marks the end of participation in this study.

## 12.6. Early Termination (If Applicable)

Participants who withdraw early from the study will be encouraged to return to the CRU for an EOS assessment. The EOS assessments will be those described for Day 8 (Section 12.5).

This visit marks the end of participation for participants that withdraw early from the study.

# 13. PHARMACOKINETIC ASSESSMENTS

## 13.1. Blood Sample Collection

Blood samples for PK analysis will be obtained, according to the site's standard operating procedures, within 30 minutes prior to dosing and at the time points delineated in the Schedule of Assessments (Table 4).

The actual collection time of each sample must be recorded in the source data documentation, on the collection tube, and in the eCRF.

## 13.2. Sample Analysis

Plasma PK sample analysis will be performed using validated procedures and methods as outlined in the Laboratory Manual.

The Sponsor will supply complete written instructions for handling, processing, storage, and shipping of samples prior to study initiation.

# 14. ASSESSMENT OF SAFETY

## 14.1. Safety Parameters

Study procedures should be completed as delineated in the Schedule of Assessments (Table 4). However, if a participant is unable to attend a visit within the specified window, the Investigator or designee should discuss appropriate scheduling with the Sponsor's MM or appropriate designee. Any unscheduled procedures required for urgent evaluation of safety concerns must take precedence over all routine scheduled procedures.

### 14.1.1. Demographic/Medical and Surgical History

Medical and surgical history (including alcohol, drug, and smoking status), date of birth, age (calculated), sex, ethnicity, and race will be recorded at Screening.

### 14.1.2. Vital Signs

Vital signs (body temperature, systolic and diastolic blood pressures, heart rate, and respiration rate) will be measured at the time points specified in the Schedule of Assessments with participants resting for at least 5 minutes in a supine position.

When the time of vital signs measurement coincides with a blood draw, the vital signs will be taken before the scheduled blood draw where possible, ensuring the blood draw is within the window specified in the protocol.

Where multiple assessments are scheduled for the same timepoint, the order of assessments will be 12-lead ECG, vital signs, blood draw, ensuring the blood draw is within the window specified.

Assessment of body temperature by the tympanic route.

Additional measurement of vital signs may be performed at other times if deemed necessary.

### 14.1.3. Weight and Height

Body height (centimetres) and body weight (kilograms) will be measured at the time points delineated in the Schedule of Assessments and will be used to calculate BMI. BMI is calculated by dividing the participant's body weight in kilograms by the participant's height in metres squared ($kg/m^2$). Body weight and height will be obtained with the participant's shoes and jacket or coat removed.

### 14.1.4. Physical Examination

Complete and symptom-directed physical examinations will be performed by a licensed physician at the time points specified in the study schedules.

Complete physical examination includes: general appearance, head, eyes, ears, nose, throat, mouth, neck, thyroid, lymph nodes, cardiovascular system, respiratory system, gastrointestinal system, renal system, central and peripheral nervous system, musculoskeletal system, dermatologic system, other.

Physical examinations may be performed at various unscheduled time points if deemed necessary by the Investigator.

### 14.1.5. Electrocardiogram

A single 12-lead ECG will be taken at the time points delineated in the Schedule of Assessments. Additional ECG monitoring may be performed at other times if deemed necessary.

ECGs will be performed with participants in a supine position. Participants must be in this position for at least 5 minutes before the reading is taken. If any abnormality is detected on the single trace, 2 additional traces, at least 2 minutes apart, will be performed for Investigator (or designee) review.

All ECG tracings will be reviewed by the PI or designee.

When the time of ECG monitoring coincides with a blood draw, the ECG will be taken before the scheduled blood draw while ensuring the blood draw is within the window specified in the protocol.

Where multiple assessments are scheduled for the same timepoint, the order of assessments will be 12-lead ECG, vital signs, blood draw, ensuring the blood draw is within the window specified.

### 14.1.6. Telemetry

To establish a baseline, telemetry should be recorded for at least 2 hours prior to dosing. This may be done 2 hours immediately prior to dosing or at some 2-hour continuous interval in the 24 hours prior to dosing. The recording is to be performed when the participant is awake. Continuous cardiac monitoring will be conducted 15 minutes pre-dose and continuously through the 8-hour post dose period.

### 14.1.7. Laboratory Assessments

Fasting safety laboratory tests (haematology, chemistry, and urinalysis) will be performed at the time points specified in the Schedule of Assessments. Additional clinical laboratory tests may be performed at other times if deemed necessary based on the participant's clinical condition.

#### 14.1.7.1. Haematology

Haematology parameters to be tested are:

- Red blood cell count (RBC)
- Haematocrit (HCT)
- Haemoglobin (HGB)
- Mean corpuscular volume (MCV)
- Mean corpuscular haemoglobin (MCH)
- Mean platelet volume (MPV)
- Platelets (PLAT)
- White Cell Count with differential (including Neutrophils [NEUT], Lymphocytes [LYM], Monocytes [MONO], Eosinophils [ESN], Basophils [BASO])

#### 14.1.7.2. Chemistry

Chemistry parameters to be tested are:

- C-reactive protein (CRP)
- Urea (U)
- Creatinine (CREAT)
- Total Bilirubin (BILI) and Direct Bilirubin (BILIDIR)
- Urate (URATE)
- Albumin (ALB)
- Globulin (GLOBUL)
- Alkaline Phosphatase (ALP)
- Creatine Kinase (CK)
- Aspartate Aminotransferase (AST)
- Alanine Aminotransferase (ALT)
- Gamma-GT (GGT)
- Glucose (GLU) (fasting labs only)
- Sodium (NA)
- Potassium (K)
- Calcium (CA)
- Chloride (CL)
- Phosphate (PHOS)
- Bicarbonate (BICARB)

#### 14.1.7.3. Urinalysis

A urinalysis test (dipstick) will be performed for each participant. Urinary analysis will be performed at Screening and other times according to the study schedule. If abnormality is noted for protein, blood, nitrite, or leukocyte esterase (and at the discretion of the Investigator) a microscopic examination of RBC, WBC, bacteria, and casts will be performed.

Microscopic urinalysis parameters to be tested are:

- pH (PH)
- Specific Gravity (SPGRAV)
- Protein (PROT)
- Glucose (GLUC)
- Ketones (KETONES)

- Occult Blood (OCCBLD)
- Nitrite (NITRITE)
- Urobilinogen (UROBIL)
- Leukocyte esterase (LEUKOCYTE ESTERASE)

#### 14.1.7.4. Viral Serology

Hepatitis B surface antigen (HBsAg), anti-hepatitis C virus (HCV), and HIV antibody testing will be performed at Screening. A COVID-19 test may be performed at Screening and/or upon admission to the CRU (Day -1) at the discretion of the Investigator in accordance with local health directives.

#### 14.1.7.5. Urine Drug Screen

A urine drug screen will be performed at Screening and at admission to the CRU (Day -1). This will include screening for:

- Tetrahydrocannabinol
- Cocaine
- Amphetamines
- Barbiturates
- Benzodiazepines
- Opiates
- Methadone
- Methamphetamines
- Ecstasy (MDMA)
- Phencyclidine
- Cotinine

#### 14.1.7.6. Alcohol Breath Test

An alcohol breath test will be performed at Screening and upon admission to the CRU (Day -1).

#### 14.1.7.7. Pregnancy Testing

A serum pregnancy test will be performed at the Screening visit and a urine pregnancy test will be performed on Day -1 and at the Day 8/EOS visit for WOCBP only.

#### 14.1.7.8. Follicle-stimulating Hormone Testing

Women not of childbearing potential must be postmenopausal (defined as cessation of regular menstrual periods for at least 12 months). Postmenopausal status will be confirmed through testing of FSH levels ≥ 40 IU/L at Screening.

## 14.2. Adverse and Serious Adverse Events

In this study, AEs will be reported for all participants from Day -1 until the completion of the Day 8/EOS visit. Serious adverse events will be reported for all participants (enrolled and not enrolled) from Day -1. Treatment-emergent AEs will be evaluated from the first administration of study drug until the Day 8/EOS visit or up to a 30-day Follow-up period for AEs deemed related to treatment. Adverse events that are ongoing at the EOS visit will be marked as Not Recovered/Not resolved on the AE eCRF page.

All spontaneously volunteered and enquired for, as well as observed AEs, will be recorded in the participant's medical records and the eCRF.

### 14.2.1. Definition of Adverse Events

An AE is any event, side-effect, or other untoward medical occurrence that occurs in conjunction with the use of a medicinal product in humans, whether or not considered to have a causal relationship to this treatment. An AE can, therefore, be any unfavourable and unintended sign (that could include a clinically significant abnormal laboratory finding), symptom, or disease temporally associated with the use of a medicinal product, whether or not considered related to the medicinal product.

An AE that starts on or after the first study treatment or that worsened after the first study treatment will be regarded as a treatment-emergent adverse event (TEAE).

Events meeting the definition of an AE include:

- Exacerbation of a chronic or intermittent pre-existing condition including either an increase in frequency and/or intensity of the condition.
- New conditions detected or diagnosed after study drug administration that occur during the reporting periods, even though it may have been present prior to the start of the study.
- Signs, symptoms, or the clinical sequelae of a suspected interaction.
- Signs, symptoms, or the clinical sequelae of a suspected overdose of either study drug or concomitant medications (overdose per se will not be reported as an AE/ SAE).

Events that do not meet the definition of an AE include:

- Medical or surgical procedure (eg, endoscopy, appendectomy); the condition that leads to the procedure should be reported as an AE if it meets the criteria of an AE.
- Situations where an untoward medical occurrence did not occur (eg, social and/or convenience admission to a hospital).
- Anticipated day-to-day fluctuations of pre-existing disease(s) or condition(s) present or detected at the start of the study that do not worsen.

If there is evidence of an AE through report or observation, the Investigator or designee will evaluate further and record the following information:

- Time of onset and resolution
- Severity

- Seriousness
- Causality/relation to study treatment
- Action taken regarding study drug
- Action taken regarding AE
- Outcome

### 14.2.2. Severity of an Adverse Event

Severity of AEs will be graded by the Investigator as one of:

- **Mild (Grade 1):** A type of AE that is usually transient and may require only minimal treatment or therapeutic intervention. The event does not generally interfere with usual activities of daily living.
- **Moderate (Grade 2):** A type of AE that is usually alleviated with additional specific therapeutic intervention. The event interferes with usual activities of daily living, causing discomfort but poses no significant or permanent risk of harm to the research participant.
- **Severe (Grade 3):** A type of AE that interrupts usual activities of daily living, or significantly affects clinical status, or may require intensive therapeutic intervention.
- **Life-threatening (Grade 4):** A type of AE that places the participant at immediate risk of death.
- **Death (Grade 5):** Events that result in death.

### 14.2.3. Causal Relationship of an Adverse Event

The Investigator will assess the relationship between study drug and the occurrence of each AE. The Investigator's assessment of the relationship of each AE to study drug will be recorded in the source documents and the eCRF. Alternative causes, such as medical history, concomitant therapy, other risk factors, and the temporal relationship of the event to the study drug should be considered and investigated, if appropriate. The following definitions are general guidelines to help assign grade of attribution:

- **Not related:** The event is clearly related to other factors such as the participant's environment or clinical state, therapeutic interventions or concomitant drugs administered to the participant. This is especially so when an event occurs prior to the commencement of treatment with the study drug.
- **Unlikely:** The temporal association, participant history, and/or circumstances are such that the study drug is not likely to have had an association with the observed event. Other conditions, including concurrent illness, progression, or expression of the disease state, or reaction to a concomitant drug administered appear to explain the event.
- **Possible:** The event follows a reasonable temporal sequence from the time of study drug administration or follows a known response to the study drug but could have

been produced by other factors such as the participant's clinical state, other therapeutic interventions, or concomitant drugs administered to the participant.

- **Probable:** The event follows a reasonable temporal sequence from the time of study drug administration and follows a known response to the study drug and cannot be reasonably explained by other factors such as the participant's clinical state, other therapeutic interventions, or concomitant drugs administered to the participant.
- **Definite:** The event follows a reasonable temporal sequence from the time of study drug administration or control abates upon discontinuation or cannot be explained by known characteristics of the participant's clinical state.

### 14.2.4. Action Taken with Investigational Product

Should the Investigator need to alter the administration of the study drug from the procedure described in the protocol due to the well-being and safety of the participant then the action taken will be recorded on the AE eCRF page, as one of the following options:

- Dose Reduced
- Drug Interrupted
- Drug Withdrawn
- Not Applicable
- Other

### 14.2.5. Outcome

Outcome of an AE will be recorded on the AE eCRF as follows:

- Recovered / Resolved
- Recovering / Resolving
- Recovered / Resolved with Sequelae
- Not Recovered / Not Resolving
- Fatal
- Unknown

## 14.3. Definition of Serious Adverse Event

An SAE is an AE occurring during any study phase (ie, Baseline, Treatment, Washout, or Follow-up), and at any dose of the study drug (active or placebo), that fulfils one or more of the following:

- Results in death
- It is immediately life-threatening
- It requires in-patient hospitalisation or prolongation of existing hospitalisation
- It results in persistent or significant disability or incapacity

- Results in a congenital abnormality or birth defect
- It is an important medical event that may jeopardise the participant or may require medical intervention to prevent one of the outcomes listed above

Important medical events that may not be one of the above may be considered an SAE by the Investigator when, based upon appropriate medical judgement, they are considered clinically significant and may jeopardise the participant, or may require medical or surgical intervention to prevent one of the outcomes listed above.

An AE is considered “life-threatening” if, in the opinion of either the Investigator or the Sponsor, its occurrence places the participant at immediate risk of death. It does not include an AE that, had it occurred in a more severe form, might have caused death.

#### 14.3.1. Notification of a Serious Adverse Event

All SAEs, regardless of relationship to the study drug, during the period starting from the time the patient signed the ICF through to the EOS will be recorded in the eCRF. Once the Investigator becomes aware of an SAE, they must report the SAE to the Novotech Pharmacovigilance (PV) Team within 24 hours of knowledge of the event.

The SAE information will be sent by the investigative site via secure email connection to:

Novotech Safety Mailbox: Safety@novotech-cro.com.

If requested, supporting de-identified source documents (eg, hospital discharge summary, autopsy report when available, results of relevant diagnostic tests completed to evaluate the event) will also be sent to the Novotech PV Team.

A written SAE report must include a full description of the event including the below parameters:

- Diagnosis or description of event
- Onset date
- Severity assessment
- Causal relationship to the IP
- Assessment of seriousness of the event
- Corrective treatment administered for the SAE
- Action taken related to study drug include the following: dose interruption, dose delay, dose reduction, or study drug discontinuation
- Outcome of event and end date

The Sponsor is responsible for notifying the relevant regulatory authorities of certain events. It is the Investigator’s responsibility to notify the HREC of all SAEs in accordance with the HREC SAE reporting policy. The Investigator will also be notified of all unexpected, serious, drug-related events that occur during the clinical trial. The investigational site is responsible for notifying its HREC of these additional SAEs.

## 14.4. Clinical Laboratory Abnormalities and Other Abnormal Assessments as Adverse Events and Serious Adverse Events

Abnormal laboratory findings (eg, haematology, chemistry, and urinalysis) or other abnormal assessments (eg, ECG and vital signs) per se are not reported as AEs. However, those abnormal findings that are deemed clinically significant by the PI and/or delegate or are associated with signs and/or symptoms must be recorded as AEs if they meet the definition of an AE (and recorded as an SAE if they meet the criteria of being serious) as previously described. Clinically significant abnormal laboratory or other abnormal findings that are detected after consent or that are present at baseline and worsen after consent are included as AEs (and SAEs if serious).

The Investigator should exercise medical and scientific judgement in deciding whether an abnormal laboratory finding or other abnormal assessment is clinically significant. To be considered clinically significant, the abnormality should be associated with a clinically evident sign or symptom or be likely to result in an evident sign or symptom in the near term. A clinically significant laboratory abnormality in the absence of clinical symptoms may jeopardise the participant and may require intervention to prevent immediate consequences. For example, a markedly low serum glucose concentration may not be accompanied by coma or convulsions yet be of a magnitude to require glucose administration to prevent such sequelae.

## 14.5. Recording Adverse Events

Adverse events spontaneously reported by the participant and/or in response to an open question from the study personnel or revealed by observation will be recorded in accordance with the Investigator's normal clinical practice and on the AE page of the eCRF during the study at the investigational site.

However, abnormal values that constitute an SAE or lead to discontinuation of administration of study drug must be reported and recorded as an AE. The AE term should be reported in standard medical terminology when possible. For each AE, the Investigator will evaluate and report the onset (date and time), resolution (date and time), intensity, causality, action taken, serious outcome (if applicable), and whether or not it caused the participant to discontinue the study. AEs that occur during the study must be documented in the participant's medical record, on the AE eCRF, and on the SAE report form. If an SAE report is completed, pertinent laboratory data should be recorded on the SAE form, preferably with baseline values and copies of laboratory reports.

In addition, if the abnormal assessment meets the criteria for being serious, the SAE form must also be completed. A diagnosis, if known, or clinical signs or symptoms if the diagnosis is unknown, rather than the clinically significant laboratory finding or abnormal assessment, should be used to complete the AE/SAE page. If no diagnosis is known and clinical signs or symptoms are not present, then the abnormal finding should be recorded.

## 14.6. Follow-up of Adverse Events and Serious Adverse Events

All AEs and SAEs that are deemed related, possibly related, or probably related to the study drug must be followed until resolution, until the condition stabilises, until the event is otherwise explained, or until the participant dies or is lost to follow-up. The Investigator is responsible for ensuring that follow-up includes any supplemental investigations as may be indicated to

elucidate as completely as practical the nature and/or causality of the AE/SAE. This may include additional laboratory tests or investigations or consultation with other health care professionals.

The Sponsor may request that the Investigator perform or arrange for the conduct of supplemental measurements and/or evaluations. If a participant dies during participation in the study or during a recognised Follow-up period, the Sponsor should be provided with a copy of any post-mortem findings, including histopathology.

## 14.7. Pregnancy

Pregnancy testing should be performed in all WOCBP throughout the study as per the Schedule of Assessments and the pregnancy results should be captured in the eCRF.

All WOCBP will be instructed to contact the Investigator immediately if they suspect they might be pregnant (eg, missed or late menstrual period) at any time during the trial. Male participants will contact the Investigator immediately if they suspect they may have fathered a child during the study Treatment Period. When possible, the partner's pregnancies should be followed (for a period of not less than 12 months following birth) to determine the outcome.

If a participant becomes pregnant during the clinical trial, the Investigator will report the details on a pregnancy form to the Sponsor/assigned designee within 24 hours of knowledge of the pregnancy. Even though participants agree to withdraw or terminate the clinical trial, the Investigator should follow up (for a period of not less than 12 months following birth) and document the process and results of all the pregnancies.

If a male participant's female partner becomes pregnant while enrolled in the trial, a pregnancy form should be completed and sent to the Novotech PV Team expeditiously, irrespective of whether it meets the criteria for expedited reporting. Abortions (spontaneous, accidental, or therapeutic) must also be reported to the Novotech PV Team. Congenital anomalies/birth defects always meet SAE criteria, and should therefore, be expeditiously reported as an SAE, using the previously described process for SAE reporting. A pregnancy form should also have been previously completed and will need to be updated to reflect the outcome of the pregnancy. The Investigator must report any pregnancy (including pregnancy of a male participant's partner), even if no AE has occurred, on a Pregnancy Report Form within 24 hours of the Investigator becoming aware of the pregnancy.

# 15. EXPLORATORY RESEARCH DATA (OPTIONAL)

## 15.1. Rationale

Exploratory research data will be used to quantify the intensity of the aging process in study participants and the changes thereof (if any). This data, which is not related to the goals of the study objectives and endpoints (see Section 7), is intended to:

- validate the proposed aging clocks in the setting of clinical trials with the healthy volunteers,
- provide the Baseline for future, more aging-focused trials.

Exploratory research data will be collected on Day -1 and Day 8. The data will be used in developing the Young Artificial Intelligence (Young.AI) platform, which is being developed by Deep Longevity (a collaborator of Insilico Medicine). All assessments generating exploratory research data will be optional for all participants, who will be consented on a separate PICF. Young.AI is a free platform that grants its users access to state-of-the-art aging clocks. It serves as a demonstration of Deep Longevity products, which contain reports on aging processes to be used in the clinical setting.

## 15.2. Background

Aging is an everyday occurrence, yet it is difficult to robustly define it for scientific research. Its scientific definition (Galkin et al., 2020) inadvertently relies on an aging model. In this particular case, it will be defined with two "aging clocks": MindAge and BloodAge.

Given the aging global population (Ritchie and Roser, 2019), validation of such models is essential for the development of geroprotectors — a potential class of drugs affecting the basic aging processes. Currently, many pharmaceuticals (Longevity Marketcap, 2021) with promising geroprotective properties are being studied in clinical trials. However, these trials do not commonly include aging clocks, despite this technology being inexpensive and readily available.

## 15.3. Assessments

### 15.3.1. MindAge

The purpose of MindAge (Zhavoronkov et al., 2020) is to estimate the most superficial level of aging that can be easily perceived as a drop in overall well-being.

The mental age of each participant will be assessed using the MindAge survey which will be completed by the participant on Day -1 and Day 8 and compared to their chronological age.

### 15.3.2. Blood Age

The purpose of BloodAge (Mamoshina et al., 2018) is to estimate aging by considering processes happening on physiological level and that cannot be easily perceived by individuals.

#### 15.3.2.1. Blood Chemistry

The physiological age of each participant will be assessed using standard blood chemistry parameters. Blood draws will be taken on Day -1 and Day 8. The total blood drawn from each participant for Blood Age analysis will be 30 mL.

#### 15.3.2.2. Lipid Panel

Participants will fast for at least 8 hours prior to blood draw for lipid panel testing which will include cholesterol, triglycerides, high density lipoprotein (HDL), and low density lipoprotein (LDL) on Day -1 and Day 8.

## 15.4. Analysis and Reporting

The exploratory research data collected during this study for research purposes (MindAge and Blood Age) will be analysed and reported separately.

The Sponsor intends to perform statistical tests to determine if the detected pace of aging changes after the administration of INS018_055 eg, comparing the means of the total sample, estimating the effect of INS018_055 on the pace of aging with a mixed-effects linear model.

# 16. STATISTICS

Statistical methods will be further outlined in a statistical analysis plan (SAP) and approved by the Sponsor prior to any analysis. Procedures outlined in the SAP will supersede protocol-specified statistical methods in the event of divergence.

In general, descriptive statistics (eg, arithmetic mean, SD, median, minimum, and maximum) will be calculated for continuous safety data by treatment and protocol-specified time point, while frequency summary (eg, number of observed and percentage of each categories) will be applied for categorical safety data by treatment and protocol-specified time point.

## 16.1. Sample Size

This study will be conducted in 8 healthy adult volunteers.

## 16.2. Analysis Populations

Participant inclusion into each population will be determined prior to the final analysis.

### 16.2.1. Safety Population

All participants who receive any amount of INS018_055 will be included in the Safety Population. The Safety Population will be used for the summaries of all safety assessments.

### 16.2.2. Pharmacokinetic (PK) Population

All participants who receive any amount of INS018_055 and have sufficiently evaluable concentration-time profile to allow determination of at least one PK parameter ($C_0$, $AUC_{0\text{-}t}$, $AUC_{0\text{-}inf}$) will be included in the PK Population. An evaluable PK profile will be determined at the discretion of the pharmacometrician following examination of participants with dosing or protocol deviations that could potentially affect the PK profile. The PK Population will be used for the PK analysis and the summaries and graphical presentation of all PK data.

## 16.3. Statistical Methods

### 16.3.1. Participant Disposition

Participant disposition will be analysed using counts and percentages. The number and percentage of screened participants, enrolled participants, treated participants, participants discontinued from the study, as well as the primary reason for discontinuation will be analysed and listed.

### 16.3.2. Demographics, Medical History, and Baseline Characteristics

Demography and baseline characteristics data will be summarised using descriptive statistics for the Safety Population.

Medical history terms will be coded using the most recent version of the Medical Dictionary for Regulatory Activities (MedDRA®) available at Novotech. Medical history will be analysed using descriptive statistics by MedDRA® system organ class (SOC) and preferred term (PT) for the Safety Population.

### 16.3.3. Prior and Concomitant Medication

Prior and concomitant medications (as defined in Section 10.2) will be coded using the most current version of the WHO drug dictionary available at Novotech. Prior and concomitant medications will be listed by participant and summarised by treatment using anatomic therapeutic chemical (ATC) and preferred name for the Safety Population.

### 16.3.4. Treatment Compliance and Exposure

Treatment compliance and exposure will be summarised and listed for all participants in the Safety Population.

## 16.4. Safety Analyses

All safety assessments, including concomitant medications, AEs, laboratory evaluations, vital signs, 12-lead ECGs, telemetry, and other safety assessments will be analysed using the Safety Population.

### 16.4.1. Adverse Event

Adverse events will be coded using the most current version of the MedDRA® available at Novotech. A by participant AE data listing, including verbatim term, PT, SOC, severity and relationship to study drug, will be provided. The number of participants experiencing TEAEs (defined in Section 14.2.1) and number of individual TEAEs will be summarised by SOC and PT. TEAEs will also be summarised by severity and by relationship to INS018_055. Separate summaries for severe TEAEs leading to discontinuation and deaths will be provided.

### 16.4.2. Laboratory Evaluations

Laboratory evaluations (including haematology, chemistry, and urinalysis) will be listed and summarised by protocol-specified collection time point. Change from baseline clinical laboratory data will be summarised for each protocol-specified collection time point.

The abnormal urinalysis result (dipstick and microscopy), if applicable, will be listed only.

### 16.4.3. Vital Signs

Vital signs (BP [systolic and diastolic], heart rate, respiratory rate, and tympanic temperature) will be listed and summarised by protocol-specified collection time point. Change from baseline will be summarised at each protocol-specified collection time point.

### 16.4.4. Electrocardiograms

ECG values will be listed and summarised by clinical assessment (normal, abnormal but not clinically significant, or abnormal and clinically significant) by protocol-specified collection time point. A summary of change from baseline at each protocol-specified time point will also presented. Change from baseline for ECG clinical assessments will be presented.

### 16.4.5. Other Safety Assessments

The following assessments will be listed by participant:

- Telemetry
- Pregnancy test/FSH test
- Urine drug screen / Alcohol breath test
- Physical examination
- Serology (HIV, hepatitis B and C screen)
- COVID-19 screen (optional)

## 16.5. Pharmacokinetics

Plasma concentrations and actual blood sampling times will be listed by treatment and protocol-specified time point and summarised using descriptive statistics — number of measurements, arithmetic mean, SD, and %CV, geometric mean, minimum, median, and maximum — at each scheduled time point. Individual and mean plasma concentration-time profiles will also be presented graphically for each treatment.

Pharmacokinetic analysis of plasma INS018_055 will be performed by a noncompartmental method using validated Phoenix WinNonlin® software (Version 8.3 or higher, Certara, USA). Pharmacokinetic analysis will be performed on the PK Population using the actual sampling times. AUC will be calculated using the Linear Up and Log Down method.

The following PK parameters will be estimated (Table 6):

**Table 6: Pharmacokinetic Parameters to be Estimated**

| Parameter | Definition |
|---|---|
| $C_0$ | Hypothetical concentration at time zero |
| $AUC_{(0\text{-}24)}$ | Area under the drug concentration-time curve, from time zero to 24 hours |
| $AUC_{(0\text{-}t)}$ | Area under the drug concentration-time curve, from time zero to t hours |
| $AUC_{(0\text{-}inf)}$ | Area under the drug concentration-time curve, from time zero to infinity |
| Kel | Apparent terminal elimination rate constant |
| $T_{last}$ | Time of final quantifiable concentration |
| $AUC_{\%\_Extrap}$ | Area under the drug concentration-time curve, % extrapolated |
| $t_{½}$ | Apparent terminal half-life |
| CL | Apparent clearance calculated as Dose/$AUC_{(0\text{-}inf)}$ |
| Vz | Apparent volume of distribution at terminal phase, calculated as Dose/(Kel × $AUC_{(0\text{-}inf)}$) |

Values for $k_{el}$, $t_{½}$, $AUC_{0\text{–}inf}$, CL, or $V_z$ will not be reported for cases that do not exhibit a terminal log-linear phase (if the adjusted coefficient of determination [$R^2$] is < 0.80 or $\%AUC_{extrap}$ is > 20) in the plasma concentration versus time profile. Additional analyses will be performed as deemed necessary upon review of the data.

# 17. DIRECT ACCESS TO SOURCE DATA/DOCUMENTS

## 17.1. Study Monitoring

Before an investigational site can enter a participant into the study, a representative of the Sponsor will visit the investigational study site to:

- Determine the adequacy of the facilities.
- Discuss with the Investigator(s) and other personnel their responsibilities regarding protocol adherence, and the responsibilities of the Sponsor or its representatives. This will be documented between the Sponsor and the Investigator.

The Sponsor has appointed Novotech to manage and monitor the study to assure them of the adequate conduct of the study and to act as the contact with the investigational site. A study monitor will be identified and will be responsible for liaison with, and support of, the investigational site.

The study monitor and regulatory authority inspectors are responsible for contacting and visiting the investigative site for the purpose of inspecting the facilities and, upon request, inspecting the various records of the study (eg, eCRFs, essential documentation, and other pertinent data) provided that participant confidentiality is respected.

During the study, the monitor will have regular contacts with the investigational site for the following:

- Provide information and support to the Investigator(s).
- Confirm that facilities remain acceptable.
- Confirm that the investigational team is adhering to the protocol, that data are being accurately recorded in the eCRFs, and that study drug accountability checks are being performed.
- Perform source data verification. This includes a comparison of the data in the eCRFs with the participant's medical records at the hospital or practice, and other records relevant to the study. This will require direct access to all original records for each participant (eg, clinic charts).
- Record and report any protocol deviations not previously sent to the Sponsor.
- Confirm AEs and SAEs have been properly documented on eCRFs.
- Confirm any SAEs have been forwarded to the Sponsor and those SAEs that met criteria for reporting have been forwarded to the HREC.

The monitor will be available between visits if the Investigator(s) or other staff needs information or advice.

## 17.2. Data Management

All data will be recorded in individual source documents. An eCRF will be created by the data management group for recording of the required data in the study database. All eCRF

information is to be filled in by site staff. If an item is not available or is not applicable, this fact should be indicated. Blank spaces should not be present unless otherwise directed.

The study monitor will perform source data verification of data entered into the eCRF and raise queries for correction by the site. The data entered into the eCRF will be subject to data validation checks for consistency and completeness by the data management group. Data queries will then be generated and sent to the investigational site for response before the database is locked and released for statistical analysis.

All eCRFs should be maintained on the system with details of any changes logged accordingly.

## 17.3. Audits and Inspections

Authorised representatives of the Sponsor, a regulatory authority, or an HREC may visit the site to perform audits or inspections, including source data verification. The purpose of a Sponsor audit or inspection is to systematically and independently examine all study-related activities and documents to determine whether these activities were conducted, and data were recorded, analysed, and accurately reported according to the protocol, ICH GCP guidelines, and any applicable regulatory requirements. The Investigator should contact the Sponsor immediately if contacted by a regulatory agency about an inspection.

# 18. QUALITY CONTROL AND QUALITY ASSURANCE

## 18.1. Compliance with Good Clinical Practice

To ensure compliance with GCP and all applicable regulatory requirements, the Sponsor or its appointed representative(s) may conduct a quality assurance audit.

The study will be carried out in accordance with the current version of the Declaration of Helsinki (Ethical Principles for Medical Research Involving Human Subjects), and with the NHMRC National Statement on Ethical Conduct in Human Research 2007 (updated 2018). The conduct of the study will be in accordance with the Integrated Addendum to ICH E6 (R1): Guideline for Good Clinical Practice ICH E6 (R2), annotated with comments by the Australian TGA (June 2018).

## 18.2. Archiving and Regulatory Inspection

All study-related documents and records are to be retained for a minimum of 15 years after trial completion. Written agreement from the Sponsor must precede destruction of the same.

In accordance with ICH GCP, this study may be selected for audit. Inspection of site facilities (eg, pharmacy, medication storage areas, laboratories) and review of study-related records may occur by the Sponsor, the Sponsor's representative(s), or regulatory authority to evaluate the study conduct and compliance with the protocol, ICH GCP, and applicable regulatory requirements.

# 19. ETHICS

## 19.1. Ethics Review

The final study protocol, including the final version of the ICF, must be approved, or given a favourable opinion in writing by an HREC as appropriate. The Investigator must submit written approval to the Sponsor before they can enrol any participant into the study.

The PI is responsible for informing the HREC of any amendment to the protocol in accordance with local requirements. In addition, the HREC must approve all advertising used to recruit participants for the study. The protocol must be re-approved by the HREC upon receipt of amendments and annually, as local regulations require.

The PI is also responsible for providing the HREC with reports of any reportable serious adverse drug reactions from any other study conducted with the study drug (active). The Sponsor will provide this information to the PI.

Progress reports and notifications of serious adverse drug reactions will be provided to the HREC according to local regulations and guidelines.

## 19.2. Ethical Conduct of the Study

The study will be performed in accordance with ethical principles that have their origin in the Declaration of Helsinki (Ethical Principles for Medical Research Involving Human Subjects) and are consistent with ICH GCP applicable regulatory requirements.

## 19.3. Written Informed Consent

The PI will ensure that the participant is given full and adequate oral and written information about the nature, purpose, possible risk and benefit of the study. Participants must also be notified that they are free to discontinue from the study at any time without prejudice. The participant should be given the opportunity to ask questions and allowed time to consider the information provided before voluntarily signing the written ICF.

The participant's signed and dated informed consent must be obtained before conducting any study procedures. The participants will be informed of their rights to privacy but will be made aware that the study data will be submitted to the Sponsor and possibly to drug regulatory authorities for review and evaluation. They will be informed also that the study monitor may inspect their medical records to verify the accuracy and completeness of the study records and results.

The acquisition of informed consent should be documented in the participant's medical records, as required, and the ICF will be signed and personally dated by the participant and by the person who conducted the informed consent discussion.

The PI must maintain the original, signed ICF. A copy of the signed ICF must be given to the participant or legal representative. The date that informed consent was signed will be recorded on the eCRF.

## 19.4. Data Protection

Participants will be informed that data will be held on file by the Sponsor and that these data may be viewed by staff including the study monitor and by external auditors on behalf of the Sponsor and appropriate regulatory authorities. Participants will also be informed that a study report will be prepared and may be submitted to regulatory authorities and for publication. However, participants will be identified in such reports only by study identification number, gender, and age. All participant data will be held in strict confidence.

## 20. REGULATORY REQUIREMENTS

Novotech will act as the legal Australian Sponsor for the study and will fulfil the obligations that this role entails. Novotech shall, to the extent required by the applicable laws and regulations, interact with the regulators on behalf of the Sponsor in connection with this study. The planned regulatory pathway for this trial is through the Clinical Trials Notification (CTN) scheme. Aside from approval by the HREC and notification of the regulator, no other regulatory approval will be required.

# 21. DATA HANDLING AND RECORDKEEPING

## 21.1. Inspection of Records

The Sponsor will be allowed to conduct site visits to the investigation facilities for the purpose of monitoring any aspect of the study. The Investigator agrees to allow the monitor to inspect the drug storage area, study drug stocks, drug accountability records, participant charts and study source documents, and other records relative to study conduct.

## 21.2. Retention of Records

All source data, clinical records and laboratory data relating to the study will be archived for 15 years after the completion of the study. All data will be available for retrospective review or audit.

Source documents are original documents, data, and records from which the participant's eCRF data are obtained. These include, but are not limited to: hospital records, clinical and office charts, laboratory and pharmacy records, diaries, microfiches, radiographs, angiograms, study drug accountability logs, and correspondence.

The Investigator and study staff are responsible for maintaining a comprehensive filing system of all study-related (essential) documentation. These include, but are not limited to: HREC correspondence, study drug accountability logs, and curricula vitae of all personnel participating in the study. These files must be suitable for inspection at any time by the Sponsor, monitor, and/or applicable regulatory authorities. All essential documentation should be retained by the institution for 15 years (as required in Australia).

No study document should be destroyed without prior written agreement between the Sponsor and the Investigator. If the Investigator wishes to assign the study records to another party or move them to another location, the PI must notify the Sponsor in writing of the new responsible person and/or the new location.

## 21.3. Liability/Indemnity/Insurance

The Sponsor will ensure sufficient insurance is available to enable it to indemnify and hold the Investigator(s) and relevant staff as well as any hospital, institution, ethics committee or the like, harmless from any claims for damages for unexpected injuries, including death, that may be caused by the study drug but only to the extent that the claim is not caused by the fault or negligence of the participants or Investigator(s).

## 22. PUBLICATION POLICY

The publication, presentation, or other public disclosure of study results (each, a "Publication") will be accurate and honest, undertaken with integrity and transparency and in accordance with the Sponsor's approval.

Publication of results will be subjected to fair peer-review. Authorship will be discussed between researchers prior to study commencement (or as soon as possible thereafter) and reviewed whenever there are changes in participation.

All conflicts arising through disputes about authorship will be reviewed by the Sponsor. Authorship should be consistent with the guidelines described in the Australian Code for Responsible Conduct of Research (section on Authorship).

Acknowledgment will be given to collaborating institutions and hospitals and other individuals and organisations providing finance or facilities. Participant confidentiality will be maintained by referring to individual participants by their identifying code used in the trial. Data will not be released publicly until the manuscript is accepted for publication. In the case of no publication, information will only be released to the public and media in accordance with the Sponsor's approval.

Study data that have not been published, presented, or otherwise disclosed in accordance with the clinical trial agreement shall remain confidential information of the Sponsor. The Investigator may not disclose or permit the disclosure of such unpublished data to any third party, nor may they disclose or permit the disclosure of any study data to any third party in greater detail than the same have been disclosed in any permitted publication, presentation, or other disclosure.

The results summary will be posted to the Australian New Zealand Clinical Trials Registry as required by legal agreement, local law, or regulation.

## 23. LIST OF REFERENCES

Chilosi M, et al. Aberrant Wnt/beta-catenin pathway activation in idiopathic pulmonary fibrosis. Am J Pathol (2003) 162: 1495–1502.

European Medicines Agency. Guideline on strategies to identify and mitigate risks for first-in-human and early clinical trials with investigational medicinal products. July 2017. EMEA/CHMP/SWP/28367/07 Rev. 1. Available from: https://www.ema.europa.eu/en/documents/scientific-guideline/guideline-strategies-identify-mitigate-risks-first-human-early-clinical-trials-investigational_en.pdf

Galkin F, et al. Biohorology and biomarkers of aging: Current state-of-the-art, challenges and opportunities. Ageing Res Rev (2020) 60: 101050.

ICH guideline M3(R2) on non-clinical safety studies for the conduct of human clinical trials and marketing authorisation for pharmaceuticals. December 2009. EMA/CPMP/ICH/286/1995. Available from: https://www.ema.europa.eu/en/documents/scientific-guideline/ich-guideline-m3r2-non-clinical-safety-studies-conduct-human-clinical-trials-marketing-authorisation_en.pdf

Insilico Medicine. INS018_055 Investigational Brochure. Version 2.0. Date: 16 September 2021.

King TE Jr, et al. Idiopathic pulmonary fibrosis: relationship between histopathologic features and mortality. Am J Respir Crit Care Med (2001) 164: 1025–1032.

Longevity Marketcap. Longevity Clinical Trial Tracker [Internet]. Available from: https://longevitymarketcap.com/longevity-trial-tracker/ (dated accessed 16 September 2021).

Mahmoudi T, et al. The kinase TNIK is an essential activator of Wnt target genes. EMBO J (2009) 28: 3329–3340.

Mamoshina P, et al. Population specific biomarkers of human aging: A big data study using South Korean, Canadian, and Eastern European patient populations. J Gerontol A Biol Sci Med Sci. (2018) 73: 1482–1490.

Morrisey EE. Wnt Signaling and Pulmonary Fibrosis. Am J Pathol (2003) 162: 1393–1397.

Myers JL. Idiopathic pulmonary fibrosis: clinical relevance of pathologic classification. Am J Respir Crit Care Med (1998) 157: 1301–1315.

Ritchie H and Roser M. Age Structure [Internet]. Our World In Data (2019). Available from: https://ourworldindata.org/age-structure (date accessed 16 September 2021).

Selman M, et al. Idiopathic pulmonary fibrosis: prevailing and evolving hypotheses about its pathogenesis and implications for therapy. Ann Intern Med (2001) 134: 136–151.

Yamada T, et al. Emergence of TNIK inhibitors in cancer therapeutics. Cancer Sci (2017) 108: 818–823.

Zhavoronkov A, et al. PsychoAge and SubjAge: development of deep markers of psychological and subjective age using artificial intelligence. Aging (2020) 12: 23548-23577.

Insilico Medicine

# INS018_055

## Protocol Number: INS018-005-001

### A PHASE 0 SINGLE MICRODOSE STUDY TO EVALUATE THE PHARMACOKINETICS OF INS018_055 IN HEALTHY PARTICIPANTS

**Subject:** ***PROTOCOL CLARIFICATION LETTER***

**Protocol Criteria: Schedule of Assessments**

Per the Schedule of Assessments, ECG, vital signs and PK are required at time 0 (i.e., end of injection) and 15 minutes post-dose. There is a +/- 5 min window on ECG/vitals and +/- 3 mins for PK. ECGs and Vital signs will also be collected at pre-dose (within 60 minutes prior to dosing).

**Clarification:**

With regard to the above-mentioned criteria, the following clarification applies:

- It will not be possible to conduct these scheduled assessments in the order required per protocol (ECG – vital signs – PK) within the windows specified.
- An increase for the tolerance window of ECG and vital sign assessments to +/- 10 minutes at the time 0 and 15-minute timepoint will be utilised as the required time period tolerance for Clinical Operations.
- There are no safety concerns with widening the window to +/-10 minutes for the ECGs and vital signs as it is adding an additional 5 minute window for these assessments.

Regards

Sign: Date: 12 Nov 2021

***Andrey Kukharenkko***
***Medical Monitor (Insilico Medicine)***

Insilico Medicine 307A,
Core Building 1,
No. 1 Science Park East Avenue,
Pak Shek Kok, New Territories, Hong Kong

# File Note

| | |
|---|---|
| **Client name and project reference:** | Insilico Medicine<br>INS018-005-001<br>Insilico Medicine |
| **Principal Investigator:** | Dr Angela Rowland |
| **Institution name:** | Novotech |
| **Site #:** | AU01 |
| **Date:** | _24_ / _Feb__ / _2022__<br>*dd mmm yyyy* |

## Details:

This Note to File serves to document the Protocol Deviation discrepancies between CTMS and Listings.

The CTMS deviation # 1-1EPH6X was associated with a participant that was a screen failure and not part of the study safety population.

The CTMS deviation # 1-1EOZBB is a site level protocol deviation associated with the following subjects: 101-001, 101-008, 101-010, 101-015. This deviation was not included in the Listings as it was not directly linked to study deviations.
The deviations are not applicable to Important Protocol Deviations.

### CTMS Deviations:

| PD # | PD Level | Region | Site # | PI Last Name | PI First Name | Screening # | Enrolment # | Visit Name | Date of Deviation | Date Deviation Identified | Deviation Type | Deviation Classification | Deviation Description | Action related to Subject | Action at Site | IPD Form Completed | Internal Review Status | Deviation Locked |
|---|---|---|---|---|---|---|---|---|---|---|---|---|---|---|---|---|---|---|
| 1-1EPH6X | Subject | Australia | AU01 | ROWLAND | ANGELA | 101-002 | N/A | Screening visit | 4-Nov-21 | 13-Dec-21 | Protocol Deviation | Operational Deviation | PICF was conducted by Dr Khine on 04Nov2021, while she was delegated by PI to work in the study on 10Nov2021 - GCP deviation | No Action Required | No Action Required | Not Applicable | Open | N |
| 1-1EOZBB | Site | Australia | AU01 | ROWLAND | ANGELA | | | | 3-Nov-21 | 6-Dec-21 | Protocol Deviation | Operational Deviation | Bacteria were not assessed as per protocol in urine microscopy for all subjects who were positive to protein, blood or leucocyte esterase in urinalysis | No Action Required | No Action Required | Not Applicable | Open | N |

## Deviation Listings:

Sponsor: Insilico Medicine Hongkong Limited
Study: INS018-005-001

Final [Clinical cut-off: 2021-12-23

Listing 16.2.2.1
Protocol Deviation
(Safety Population)

| Participant ID | Date of Deviation (YYYY-MM-DD) | Type of Deviation | Deviation Description | Important Deviation |
|---|---|---|---|---|
| 101-001 | 2021-11-23 | Protocol Deviation | Respiration rate data collection was missed in pre-dose vital signs due to staff error | No |
| 101-003 | 2021-11-23 | Protocol Deviation | Lipid studies tests were performed on Day 2 in error which is not required as per protocol | No |
| | 2021-11-30 | Protocol Deviation | All follow-up visit activities were not performed within the window (168 hrs, +/- 1 day) after the stop time of IP infusion as per the protocol | No |
| 101-006 | 2021-11-11 | Protocol Deviation | PICF was conducted by Dr Pradhan on 11Nov2021, while she was delegated by PI to work in the study on 22Nov2021 - GCP deviation | No |
| 101-009 | 2021-11-23 | Protocol Deviation | 0-min 12-lead ECG was performed 1-min away from the protocol window (+/- 10 minutes) after stop time of IP infusion | No |
| 101-010 | 2021-11-23 | Protocol Deviation | 8-hr post-dose vital signs time is not recorded in source due to staff error and the activity time is unknown | No |

**From:** Benjamin Marrone <Benjamin.Marrone@cmax.com.au>
**Sent:** Thursday, 17 February 2022 4:45 PM
**To:** Jackie Murphy <Jackie.Murphy@novotech-cro.com>
**Cc:** Dania Ruminski-Smith <Dania.Ruminski-Smith@novotech-cro.com>
**Subject:** [EXTERNAL]: RE: Insilico Medicine

CAUTION: This email originated from outside of the organisation. Do not click links or open attachments unless you recognize the sender and know the content is safe.

Hi Jackie,

This PD related to the following subjects: 101-001, 101-008, 101-010, 101-015.

Hope this helps.

Kind Regards,
Ben

**Benjamin Marrone**
Study Coordinator

*If you are feeling unwell or have had contact with a confirmed or suspected case of COVID-19, please do not enter the facility and instead consult a health professional such as your GP.*

| | | | | |
|---|---|---|---|---|
| **Name:** | Evgenii Babin | | | |
| **Position:** | Project Manager (Insilico Medicine) | | | |
| **Signature:** | Evgenii Babin | *Electronically signed by: Evgenii Babin Reason: I have reviewed Date: Mar 23, 2022 09:44 GMT+3* | **Date:** | ____ / ______ / _______ *dd mmm yyyy* |
| **Name:** | Briohny Johnston | | | |
| **Position:** | Project Manager (CMAX) | | | |
| **Signature:** | Briohny Johnston | *Electronically signed by: Briohny Johnston Reason: I have reviewed Date: Mar 23, 2022 11:22 GMT+10.5* | **Date:** | ____ / ______ / _______ *dd mmm yyyy* |

# File Note

| | |
|---|---|
| **Client name and project reference:** | Insilico Medicine<br>INS018-005-001 |
| **Principal Investigator:** | Dr Angela Rowland |
| **Institution name:** | Novotech |
| **Site #:** | AU01 |
| **Date:** | _25_ / _Feb__ / _2022__<br>*dd mmm yyyy* |

## Details:

This Note to File serves to document additional information from the site CMAX regarding the Listing 16.2.7.1 and AE associated with participant 101-008 that is listed as “Possibly Related”

The sponsor requested additional information from the CMAX site regarding this AE.

The participants book indicates, there are no other medications recorded from shortly prior to screening through to EOS, other than the study drug itself.

Notes left by the PI are transcribed below.

The PI initial/final reviewed and closed the AE as it occurred approximately 45 mins after dosing with no subsequent episodes.

23 Nov 2021 11:15 – Localised muscle ‘spasm’ (L) Tricep + (L) neck mm (points to sternocleidomastoid muscle ) ~ 45 min post dose. Non-dosing arm. Brief and self-limiting with 3 beats / spasms myoclonus with short pause and then further 2. Not painful. No other associated symptoms. Not typical for participant. Had been lying supine in bed but not related to any study interventions at that time. None further for 45 mins. Examined – no observed fasciculations or myoclonus. Muscle belly of triceps/biceps + sternocleidomastoid muscle soft and non-tender

Site communication emails have been included in this File Note.

[EXTERNAL]: RE: Insilico Medicine

BM Benjamin Marrone <Benjamin.Marrone@cmax.com.au>
To Evgenii Babin; Jackie Murphy
Cc Briohny Johnston; Andrey Kukharenko; Manny Marquez; Sandra Kent; Aaron Tabensky

Reply | Reply All | Forward

Wed 23/02/2022 11:25 AM

This sender Benjamin.Marrone@cmax.com.au is from outside your organization.

External Senders - EXTERNAL: This email originated from outside of the organisation. Do not click any links or open any attachments unless you trust the sender and know the content is safe.

CAUTION: This email originated from outside of the organisation. Do not click links or open attachments unless you recognize the sender and know the content is safe.

Hi All,

I have transcribed the notes left by the PI below. The PI initial/final reviewed and closed the AE as it occurred approximately 45 mins after dosing with no subsequent episodes.

23 Nov 2021 11:15 – Localised muscle 'spasm' (L) Tricep + (L) neck mm (points to sternocleidomastoid muscle ) ~ 45 min post dose. Non-dosing arm. Brief and self-limiting with 3 beats / spasms myoclonus with short pause and then further 2. Not painful. No other associated symptoms. Not typical for participant. Had been lying supine in bed but not related to any study interventions at that time. None further for 45 mins. Examined – no observed fasciculations or myoclonus. Muscle belly of triceps/biceps + sternocleidomastoid muscle soft and non-tender

No medical history was reported by the participant.

Kind Regards,
Ben

**Benjamin Marrone**
**Study Coordinator**

*If you are feeling unwell or have had contact with a confirmed or suspected case of COVID-19, please do not enter the facility and instead consult a health professional such as your GP.*

[EXTERNAL]: RE: Insilico Medicine

BM Benjamin Marrone <Benjamin.Marrone@cmax.com.au>
To Evgenii Babin
Cc Jackie Murphy; Briohny Johnston; Andrey Kukharenko; Manny Marquez; Sandra Kent; Aaron Tabensky

Reply | Reply All | Forward

Thu 24/02/2022 3:15 PM

This sender Benjamin.Marrone@cmax.com.au is from outside your organization.

External Senders - EXTERNAL: This email originated from outside of the organisation. Do not click any links or open any attachments unless you trust the sender and know the content is safe.

CAUTION: This email originated from outside of the organisation. Do not click links or open attachments unless you recognize the sender and know the content is safe.

Hi Evgenii,

After looking through the participants book, there are no other medications recorded from shortly prior to screening through to EOS, other than the study drug itself.

Kind Regards,
Ben

**Benjamin Marrone**
**Study Coordinator**

*If you are feeling unwell or have had contact with a confirmed or suspected case of COVID-19, please do not enter the facility and instead consult a health professional such as your GP.*

| | | | |
|---|---|---|---|
| **Name:** | Evgenii Babin | | |
| **Position:** | Project Manager (Insilico Medicine) | | |
| **Signature:** | Evgenii Babin *Electronically signed by: Evgenii Babin Reason: I have reviewed Date: Mar 23, 2022 09:44 GMT+3* | **Date:** | ____ / ______ / _______ dd mmm yyyy |
| **Name:** | Briohny Johnston | | |
| **Position:** | Project Manager (CMAX) | | |
| **Signature:** | Briohny Johnston *Electronically signed by: Briohny Johnston Reason: I have reviewed Date: Mar 23, 2022 11:22 GMT+10.5* | **Date:** | ____ / ______ / _______ dd mmm yyyy |

DocuSign Envelope ID: 774CDD36-E051-4E09-B4BF-7A852F085DB0

# CLINICAL STUDY PROTOCOL

# A PHASE 1, RANDOMIZED, DOUBLE-BLIND, PLACEBO-CONTROLLED, ORAL SINGLE AND MULTIPLE ASCENDING DOSES, PARALLEL GROUP AND EXPLORATORY DRUG-DRUG INTERACTION STUDY TO EVALUATE THE SAFETY, TOLERABILITY, PHARMACOKINETICS, AND INTERACTION POTENTIAL OF INS018_055 IN HEALTHY SUBJECTS

## PROTOCOL NO. INS018-055-001

| | |
|---|---|
| **Sponsor:** | InSilico Medicine Hong Kong Limited<br>Unit 307A, Core Building 1, No. 1 Science Park East Avenue, Pak Shek Kok, New Territories, Hong Kong |
| **Sponsor Contact:** | Sujata Rao, MD, PhD<br>Senior Vice President, Clinical Development<br>Telephone: +86 21 50831718 |
| **Medical Monitor:** | PPD Medical Monitor<br>3900 Paramount Parkway<br>Morrisville, NC 27560 USA<br>Telephone (24/7): +1-888-483-7729<br>Fax: +1-888-529-3580 |
| **Version of Protocol:** | Amendment 3 (Version 4.0) |
| **Date of Protocol:** | 23 August 2022 |

## CONFIDENTIAL

The concepts and information contained in this document or generated during the study are considered proprietary and may not be disclosed in whole or in part without the expressed, written consent of InSilico Medicine Hong Kong Limited.

The study will be conducted according to the International Council for Harmonisation Guideline E6(R2): Good Clinical Practice.

DocuSign Envelope ID: 774CDD36-E051-4E09-B4BF-7A852F085DB0



# SIGNATURE PAGE

**PROTOCOL TITLE:** A Phase 1, Randomized, Double-Blind, Placebo-Controlled, Oral Single and Multiple Ascending Doses, Parallel Group and Exploratory Drug-Drug Interaction Study to Evaluate the Safety, Tolerability, Pharmacokinetics, and Interaction Potential of INS018_055 in Healthy Subjects

**PROTOCOL NUMBER:** INS018-055-001

DocuSigned by:
Sujata Rao
0D676E39AC874C0...

8/23/2022

Sujata Rao
Senior Vice President, Clinical Development
InSilico Medicine Hong Kong Limited

Date

# INVESTIGATOR PROTOCOL AGREEMENT PAGE

I have carefully read this study protocol and agree that it contains all necessary information required to conduct this study. I agree to conduct the study according to this protocol (including any amendments) and in accordance with clinical sites Standard Operating Procedures, International Council for Harmonisation Good Clinical Practice, all other applicable regulations, and the recommendations laid down in the most recent version of the Declaration of Helsinki.

[signature]
Signature of Principal Investigator

07 Sep 22
Date

Dr Chris Wynne, MBChB, FRACR, DPharm Med

Printed Name of Principal Investigator

DocuSign Envelope ID: 774CDD36-E051-4E09-B4BF-7A852F085DB0



# TABLE OF CONTENTS

**SIGNATURE PAGE** ..... **2**
**INVESTIGATOR PROTOCOL AGREEMENT PAGE** ..... **3**
**TABLE OF CONTENTS** ..... **4**
**PROTOCOL SYNOPSIS** ..... **7**
**1. INTRODUCTION** ..... **20**
1.1 BACKGROUND ..... 20
1.2 RATIONALE FOR STUDY ..... 23
1.3 RATIONALE FOR DOSE SELECTION ..... 24
**2. STUDY OBJECTIVES** ..... **24**
2.1 PRIMARY OBJECTIVE ..... 24
2.2 SECONDARY OBJECTIVES ..... 24
2.3 EXPLORATORY OBJECTIVE ..... 25
**3. STUDY DESIGN** ..... **25**
3.1 SCHEDULE OF EVENTS ..... 31
**4. STUDY POPULATION** ..... **40**
4.1 INCLUSION CRITERIA ..... 40
4.2 EXCLUSION CRITERIA ..... 41
4.3 OTHER SCREENING CONSIDERATIONS ..... 45
4.3.1 Lifestyle Restrictions ..... 45
4.3.1.1 Meals and Dietary Requirements ..... 45
4.3.1.2 Caffeine and Alcohol ..... 46
4.3.1.3 Activity ..... 46
4.3.1.4 Contraception ..... 47
4.4 WITHDRAWAL OF SUBJECTS FROM THE STUDY ..... 48
4.4.1 Reasons for Withdrawal ..... 48
4.4.2 Handling of Withdrawals ..... 49
4.4.3 Replacements ..... 50
**5. STUDY TREATMENTS** ..... **50**
5.1 TREATMENTS ADMINISTERED ..... 51
5.1.1 Dose Escalation ..... 52
5.1.1.1 Part A (SAD) ..... 52
5.1.1.2 Part B (MAD) ..... 53
5.1.2 Dose Escalation Stopping Criteria ..... 54
5.1.3 Part C (DDI) ..... 55
5.2 INVESTIGATIONAL PRODUCTS ..... 56
5.2.1 Study Drug Packaging and Storage ..... 56
5.2.2 Study Drug Accountability ..... 57

DocuSign Envelope ID: 774CDD36-E051-4E09-B4BF-7A852F085DB0



5.3 METHOD OF ASSIGNING SUBJECTS TO TREATMENT GROUPS.........57
5.4 BLINDING.........57
5.4.1 Blinding Procedures.........57
5.4.2 Breaking the Blind.........58
5.5 TREATMENT COMPLIANCE.........58
5.5.1 Prior and Concomitant Medications.........58
5.5.1.1 Prior Medications.........58
5.5.1.2 Concomitant Medications.........58
**6. STUDY PROCEDURES.........59**
6.1 PHARMACOKINETIC ASSESSMENTS AND ENDPOINTS.........59
6.1.1 Pharmacokinetic Sample Collection.........62
6.1.2 Pharmacokinetic Sample Analysis.........62
6.2 SAFETY ASSESSMENTS AND ENDPOINTS.........62
6.2.1 Adverse Events.........63
6.2.1.1 Adverse Event Definitions.........63
6.2.1.2 Eliciting and Documenting Adverse Events.........64
6.2.1.3 Reporting Adverse Events.........65
6.2.1.4 Assessment of Severity.........66
6.2.1.5 Assessment of Causality.........66
6.2.1.6 Follow-Up of Adverse Events.........67
6.2.2 Clinical Laboratory Testing.........67
6.2.3 Vital Sign Measurements.........68
6.2.4 Electrocardiograms.........69
6.2.5 Physical Examinations.........69
6.3 BIOMARKERS.........70
6.3.1 CD4+ and CD8+ Subpopulation of T-Cells.........70
6.3.2 Phospho-NF-kB p65 (Ser536), Phospho-Smad2 (Ser465/467), Total NF-kB p65, and Total Smad2.........71
6.3.3 IL-6 and TGF-β.........71
6.3.4 MMP-2, MMP-9, and MMP-7.........72
6.4 METABOLITE IDENTIFICATION AND PROFILING.........72
**7. STATISTICAL ANALYSIS PLANS.........72**
7.1 SAMPLE SIZE CALCULATIONS.........72
7.2 ANALYSIS SETS.........73
7.3 DESCRIPTION OF SUBGROUPS TO BE ANALYZED.........73
7.4 STATISTICAL ANALYSIS.........73
7.4.1 Pharmacokinetic Analyses.........74
7.4.2 Safety Analyses.........75

DocuSign Envelope ID: 774CDD36-E051-4E09-B4BF-7A852F085DB0



7.4.3 Biomarker Analyses ........ 75
7.5 HANDLING OF MISSING DATA ........ 76
7.6 INTERIM ANALYSES ........ 76
**8. REFERENCE LIST ........ 77**
**9. APPENDICES ........ 80**
9.1 APPENDIX 1: LIST OF ABBREVIATIONS ........ 80
9.2 APPENDIX 2: STUDY GOVERNANCE ........ 82
9.2.1 Data Quality Assurance ........ 82
9.2.2 Investigator Obligations ........ 82
9.2.2.1 Confidentiality ........ 82
9.2.2.2 Institutional Review ........ 83
9.2.2.3 Subject Consent ........ 83
9.2.2.4 Study Reporting Requirements ........ 83
9.2.2.5 Investigator Documentation ........ 84
9.2.2.6 Study Conduct ........ 84
9.2.2.7 Case Report Forms and Source Documents ........ 84
9.2.2.8 Adherence to Protocol ........ 85
9.2.2.9 Reporting Adverse Events ........ 85
9.2.2.10 Investigator’s Final Report ........ 85
9.2.2.11 Records Retention ........ 85
9.2.2.12 Publications ........ 86
9.2.3 Study Management ........ 86
9.2.3.1 Monitoring ........ 86
9.2.3.2 Management of Protocol Amendments and Deviations ........ 87
9.2.3.3 Study Termination ........ 87
9.2.3.4 Final Report ........ 88
9.3 APPENDIX 3: ESTIMATED GLOMERULAR FILTRATION RATE ........ 89

DocuSign Envelope ID: 774CDD36-E051-4E09-B4BF-7A852F085DB0



# PROTOCOL SYNOPSIS

**PROTOCOL NO.:** INS018-055-001

**TITLE:** A Phase 1, Randomized, Double-Blind, Placebo-Controlled, Oral Single and Multiple Ascending Doses, Parallel Group and Exploratory Drug-Drug Interaction Study to Evaluate the Safety, Tolerability, Pharmacokinetics, and Interaction Potential of INS018_055 in Healthy Subjects

**STUDY PHASE:** 1

**STUDY SITE(S):** 1 clinical site in New Zealand

**OBJECTIVES:**

The primary objective of this study is to assess the safety and tolerability of single and multiple oral ascending doses of INS018_055 administered to healthy subjects.

The secondary objectives of this study are:

- To determine the pharmacokinetics (PK) of INS018_055 following single and multiple oral escalating doses in healthy subjects.
- To assess the effect of food on the PK of INS018_055 following an oral dose.

**STUDY DESIGN:**

This is a Phase 1 study of INS018_055, designed in 3-parts (A, B, and C):

- Parts A and B will be randomized, double-blind, placebo controlled single (Part A) and multiple (Part B) ascending dose study parts designed to assess the safety, tolerability, and PK of INS018_055 when administered as oral doses to healthy subjects. Additionally, the impact of food on the PK of INS018_055 will be investigated
- Part C will be an open-label, non-randomized, 2-period drug-drug interaction (DDI) study part to evaluate the effect of multiple oral doses of INS018_055 on the single-dose PK of caffeine in healthy subjects.

**Part A (Single Ascending Dose [SAD]):**

Eight healthy subjects will be assigned to each of up to 5 sequential dose cohorts and will be randomly assigned within each dose cohort to receive INS018_055 or a matched placebo in a ratio of 3:1 (6 active, 2 placebo) on Day 1 for a total of up to approximately 40 subjects. Subjects will be admitted to the study site on Day -1 and dosed on Day 1 after fasting (nothing to eat or drink, except water) for at least 10 hours. Water is not allowed 1 hour prior to and 1 hour after dosing. Water can be consumed ad libitum at other times. Subjects will remain fasted on Day 1 for 4 hours after dosing. Subjects will be discharged from the study site on Day 4, after the scheduled procedures and review of the Day 4 safety data by the investigator (or designee). Subjects will come back for an end-of-study (EOS) visit on Day 8 (± 1 day).

A sentinel cohort of 2 subjects will be used to mitigate the risk of unexpected adverse events (AEs) not predicted by preclinical pharmacology and toxicology studies for each dosing

DocuSign Envelope ID: 774CDD36-E051-4E09-B4BF-7A852F085DB0



cohort, starting with Cohort 1 (the initial dose cohort). The sentinel subjects will be dosed in a blinded fashion (1 active, 1 placebo) and monitored for at least 1 day before the remaining 6 subjects in that cohort are dosed. Initiation of dosing of the remaining 6 subjects will depend on an initial safety review by the investigator indicating that administration of the study treatment was safe and well tolerated in the sentinel subjects.

Dose escalation will occur only after the real time PK, safety, and tolerability data (including reported AEs, physical examinations, vital signs, electrocardiograms [ECGs], and clinical laboratory results up to 48 hours after dosing) of the preceding dose cohort for at least 6 subjects are assessed and the study treatment is deemed safe and well tolerated by the safety review committee (SRC). As new safety and/or PK data become available, the anticipated dose escalation scheme may change following a review of the data by the SRC. Preliminary PK data from subjects in this study will help guide dose escalation to higher doses. Dose escalation in Cohorts 4 and 5 will only occur after the PK data from at least the first 2 cohorts have been assessed and deemed sufficient to model exposures for Cohorts 4 and 5.

**Food Effect Assessment (Cohort 4 in Part A only)**: Subjects in Cohort 4 of Part A will participate in a food effect arm. The actual dose level for food effect cohort is subject to change and will be confirmed by the SRC after review of data from at least the first 2 cohorts of the SAD. Following the standard SAD dosing on Day 1 and at least 3 days of follow up/washout (or 5 times the estimated half-life of INS018_055 based on the observed data, whichever is longer) (Period 1), enrolled subjects will enter Period 2 and be re-administered the same dose of INS018_055 or matching placebo (the same treatment that they received on Day 1 of Period 1) after completing a standard high-fat meal. Subjects will fast overnight (nothing to eat and drink, except water) for at least 10 hours and receive a standard high-fat breakfast 30 minutes prior to dosing. The high-fat breakfast will be consumed within approximately 30 minutes. Subjects will remain at the study site on Day 4 of Period 2 until the scheduled procedures and review of the Day 4 safety data by the investigator (or designee) are completed. The washout period of at least 3 days (or 5 times the estimated half-life of INS018_055 based on the observed data, whichever is longer) may be extended based on available PK data from previous cohorts. The Day -1 procedures will not be repeated for Period 2.

Physical examinations, vital sign measurements, 12-lead ECGs, and clinical laboratory evaluations will be performed at selected time points throughout the study. Subjects will also be closely monitored for AEs throughout the study. Blood samples for PK will be collected up to 72 hours after study treatment administration on Day 1 for all cohorts. For biomarkers (cluster of differentiation [CD]4+ and CD8+ T-cells), blood samples will be collected at check-in (Day -1), Day 2, and Day 4 after administration of INS018_055. Fecal occult blood tests will be done with all stools passed at screening, check-in (Day -1), and during the 72-hour observation period. Subjects will be discharged on Day 4.

**Part B (Multiple Ascending Dose [MAD]):**

Eight healthy subjects will be assigned to each of up to 3 sequential once daily (QD) or twice daily (BID) dose cohorts and will be randomly assigned within each dose cohort to receive INS018_055 or a matched placebo for 7 days in a ratio of 3:1 (6 active, 2 placebo), for a total of up to approximately 24 subjects.

DocuSign Envelope ID: 774CDD36-E051-4E09-B4BF-7A852F085DB0



Subjects in Part B will begin dosing, after safety and tolerability data (including reported AEs, physical examination, clinical laboratory results, 12-lead ECGs, and vital signs) from at least the first 3 cohorts (Cohorts 1 to 3) in Part A are assessed and the study treatment is deemed safe and well tolerated by the SRC. Additionally, sufficient PK data from at least the first 3 cohorts of Part A must be obtained to model exposures in Part B. The dosing schedule, either QD or BID, will be decided by the SRC after review of the PK, safety, and tolerability data from the first 3 cohorts of Part A.

The initial MAD cohorts will begin dosing with the remaining SAD cohorts. The top dose explored in Part B will not exceed the maximum dose explored in Part A. Subsequent dose escalations in Part B will not occur until the safety (including AEs, physical examination, clinical laboratory results, vital signs, and 12-lead ECGs) and tolerability data up to and including Day 11 of the preceding MAD dose cohort for at least 6 subjects are assessed and the study treatment is deemed safe by the SRC.

As new safety or PK data become available, the dose escalation scheme may change. Subjects may not receive a subsequent higher dose and may instead be administered a lower dose or may repeat the same daily dose with a different administration schema, eg, BID instead of QD dosing, to achieve lower maximum observed plasma concentration ($C_{max}$) values, particularly if the safety findings are believed to be linked to the $C_{max}$ values.

The highest dose in the MAD (either QD or BID) will be dosed in a staggered way if accrued PK data from previous cohorts predict potential exposures above the no observed adverse effect level, with 2 subjects starting dosing at least 7 days before the remaining 6 subjects; initiation of dosing of the remaining 6 subjects will depend on an initial safety review by the investigator indicating that administration of the study treatment was safe and well tolerated in the sentinel subjects.

Each subject in Part B will be admitted to the study site on Day -1 and will begin daily dosing on Day 1. Study treatment is to be administered daily at approximately the same time in the morning. For BID dosing, the second dose of study treatment will be administered approximately 12 hours after the morning dose of study treatment.

Prior to the morning dosing on Days 1 and 7 for Cohorts 6 to 8, subjects will fast (nothing to eat and drink, except water) for at least 8 hours overnight and remain fasted for 4 hours after study treatment administration. Water is not allowed 1 hour prior to and 1 hour after dosing. Water can be consumed ad libitum at other times. Water is not restricted for the evening doses when study treatment is being dosed BID. On Days 1 and 7, lunch will be provided approximately 4 hours after the morning dose, dinner will be provided approximately 10 hours after the morning dose, and a snack will be provided approximately 13 hours after the morning dose.

On Days 2 to 6 for Cohorts 6 to 8, breakfast will be provided approximately 60 minutes after study treatment administration. Standardized meals will be provided to the subjects throughout the study. Lunch will be provided approximately 4 hours after the morning dose, dinner will be provided approximately 10 hours after the morning dose, and a snack will be provided approximately 13 hours after the morning dose.

Physical examinations, vital sign measurements, 12-lead ECGs, and clinical laboratory evaluations (including liver function test results) will be performed at selected time points

DocuSign Envelope ID: 774CDD36-E051-4E09-B4BF-7A852F085DB0



throughout the dosing interval. Subjects will be closely monitored for AEs throughout the study. Blood samples for PK analyses will be collected up to 72 hours after study treatment administration on Day 7. Urine samples for PK analysis will be collected before dosing (Day -1 as a single void collected within 0 to 24 hours) and over the following intervals after dosing: 0 to 4, 4 to 8, 8 to 12, and 12 to 24 hours on Day 1 and 0 to 4, 4 to 8, 8 to 12, 12 to 24, and 24 to 48 hours on Day 7 for either QD or BID cohorts. For biomarkers (CD4+ and CD8+ T-cells, phospho-nuclear factor [NF]-kB p65 [Ser536], phospho-SMAD family member [Smad]2 [Ser465/467], interleukin [IL]-6, transforming growth factor [TGF]-β, matrix metalloproteinase [MMP]-2, MMP-9, MMP-7, total NF-kB p65, and total Smad2), blood samples will be collected at selected time points for either QD or BID dosing cohorts. Fecal occult blood tests will be done with all stool samples at screening, check-in (Day -1), and throughout Part B on site observation periods, ie, up to Day 11.

Plasma and urine samples will be collected from subjects in Cohort 8 (only) up to 24 hours after dosing on Day 7 for metabolite identification and profiling of INS018_055.

Subjects will be discharged from the study site on Day 11, after the scheduled procedures and review of the safety data by the investigator (or designee). Subjects will return to the study site on Day 25 (± 3 days) for an EOS visit.

**Part C (Drug-Drug Interaction [DDI]):**

Sixteen healthy subjects will be admitted to the study site on Day -1. Part C will have 2 treatment periods: Period 1 (Days 1 through 4) and Period 2 (Days 5 through 21). In Period 1, all subjects will receive a single oral dose of caffeine 200 mg in the morning on Day 1, followed by a washout from Days 2 to 4. In Period 2, subjects will receive multiple oral doses of INS018_055 (either QD or BID) for 14 days (Days 5 through 18) at the highest well tolerated dose tested (Cohort 8 dose, unless tolerability results require selection of a lower dose level) in Part B (MAD), with a single oral dose of caffeine 200 mg (Day 18). On Day 18, dosing of caffeine will occur 2 hours prior to INS018_055 dosing. Subjects will stay at the study site from Day -1 until study discharge on Day 21.

Subjects in Part C will begin dosing with INS018_055 in Period 2, after safety and tolerability data (including reported AEs, physical examination, clinical laboratory results, 12-lead ECGs, and vital signs) from the 3 cohorts (Cohorts 6 to 8) in Part B (MAD) are assessed and the study treatment is deemed safe and well tolerated by the SRC. Additionally, sufficient PK data from the 3 cohorts of Part B must be obtained to model exposures in Part C.

Prior to caffeine dosing on Days 1 and 18 (serial PK sampling days), subjects will fast (nothing to eat and drink, except water) for at least 8 hours overnight and remain fasted for 4 hours after study treatment administration. Water is not allowed 1 hour prior to and 1 hour after dosing. Water can be consumed ad libitum at other times. Water is not restricted for INS018_055 only dosing days (Days 5 through 17). On Days 1 and 18, lunch will be provided approximately 4 hours after the morning dose, dinner will be provided approximately 10 hours after the morning dose, and a snack will be provided approximately 13 hours after the morning dose. On Days 5 to 17, breakfast will be provided approximately 60 minutes after study treatment administration. Standardized meals will be provided to the subjects throughout the study. Lunch will be provided approximately 4 hours after the

DocuSign Envelope ID: 774CDD36-E051-4E09-B4BF-7A852F085DB0



morning dose, dinner will be provided approximately 10 hours after the morning dose, and a snack will be provided approximately 13 hours after the morning dose.

Physical examinations, vital sign measurements, 12-lead ECGs, and clinical laboratory evaluations will be performed at selected time points throughout the dosing interval. Subjects will be closely monitored for AEs throughout the study. Blood samples for PK analysis of caffeine will be collected up to 72 hours after study treatment administration on Day 1 and Day 18, following dosing with caffeine. Blood samples for PK analysis of INS018_055 in plasma will also be collected before administration of INS018_055 on Days 5 through 18 for the assessment of steady state. Fecal occult blood tests will be done with all stool samples at screening, check-in (Day -1), and during Period 2 from Day 5 through Day 21.

Subjects will be discharged from the study site on Day 21, after the scheduled procedures and review of the safety data by the investigator (or designee). Subjects will return to the study site on Day 24 (± 2 days) for an EOS visit.

**STUDY POPULATION:**
**Inclusion Criteria:**
Each subject must meet all of the following criteria to be enrolled in this study:

1. The subject is a male or female 18 to 55 years of age, inclusive.
2. The subject has a body mass index 18 to 32 kg/m$^2$, inclusive, and a total body weight ≥50 kg, inclusive, at screening.
3. The subject is considered by the investigator to be in good general health as determined by medical history, clinical laboratory test results, vital sign measurements, 12-lead ECG results, and physical examination findings at screening.
4. Female subjects of childbearing potential must be non-pregnant and non-lactating and must use one of the methods of contraception listed below for the duration of the treatment until at least 28 days after the last dose of the study drug, or be surgically sterile (ie, hysterectomy, bilateral tubal ligation, or bilateral oophorectomy), or postmenopausal (defined as amenorrhea 12 consecutive months and documented plasma follicle-stimulating hormone level >40 IU/mL). Female subjects must have a negative pregnancy test at screening and before the first dose of study drug.

   Highly effective methods of contraception are those that result in a failure rate of less than 1% per year when used consistently. Examples are provided below:

   a. Implant contraceptive (eg, Jadelle®)
   b. Intrauterine device containing either copper or levonorgestrel (eg, Mirena®)
   c. Male sterilization with absence of sperm in the post-vasectomy ejaculate

   OR an effective method that result in a failure rate of less than 5% to 10% per year. Examples are provided below:

   d. Injectable contraceptive (eg, Depo Provera)
   e. Oral contraceptive pill (combined hormonal contraceptive pill or progestogen-only ‘mini-pill’)

DocuSign Envelope ID: 774CDD36-E051-4E09-B4BF-7A852F085DB0



f. Vaginal contraceptive ring (eg, NuvaRing®)

Female subjects must also agree not to donate eggs, from dosing until at least 28 days after the last dose of study drug.

A male subject and his female partner who is of childbearing potential must agree to use one of the methods of contraception listed above for the duration of the treatment until at least 28 days after the last dose of the study drug. A male subject must also agree not to donate sperm, for the duration of the treatment until at least 28 days after the last dose of the study drug.

5. The subject agrees to comply with all protocol requirements.
6. The subject is able to provide written informed consent.

**Exclusion Criteria:**
Subjects meeting any of the following criteria will be excluded from the study:

1. The subject has current evidence or history of clinically significant hematological, renal, endocrine, pulmonary, gastrointestinal, cardiovascular, hepatic, psychiatric, neurologic, or allergic disease (including drug allergies, but excluding untreated, asymptomatic, seasonal allergies at time of dosing).
2. The subject has any condition possibly affecting drug absorption (eg, gastrectomy).
3. The subject has a history of cancer with the exception of adequately treated basal cell or squamous cell carcinoma of the skin.
4. The subject has supine blood pressure (BP) >140 mm Hg (systolic) or >90 mm Hg (diastolic), following at least 5 minutes of supine rest. If BP is >140 mm Hg (systolic) or >90 mm Hg (diastolic), the BP should be repeated 2 more times and the average of the 3 BP values should be used to determine the subject's eligibility at screening.
5. The subject has 12-lead ECG demonstrating corrected QT interval by Fridericia (QTcF) >450 msec, or a QRS interval >120 msec at screening. If QTcF exceeds 450 msec, or QRS interval exceeds 120 msec, the ECG should be repeated 2 more times and the average of the 3 QTcF (or QRS interval) values should be used to determine the subject's eligibility.
6. The subject has ANY of the following abnormalities in clinical laboratory tests at screening, as assessed by the study-specific laboratory and confirmed by a single repeat, if deemed necessary:
   a. Serum creatinine level above the upper limit of normal (ULN) or an estimated glomerular filtration rate value <80 mL/min/1.73 $m^2$ calculated with the Chronic Kidney Disease Epidemiology Collaboration formula and the absence of protein in urine, at screening.
   b. Aspartate aminotransferase or alanine aminotransferase values more than >1.5 × ULN.
   c. Fasting glucose >110 mg/dL (6.1 mmol/L).
   d. Total bilirubin >1.5 × ULN.
   e. Hematological values outside the normal reference range for local laboratory results.

DocuSign Envelope ID: 774CDD36-E051-4E09-B4BF-7A852F085DB0



f. Positive fecal occult blood test at screening or at check-in (Day -1).

7. The subject has any medical history of disease that has the potential to cause a rise in total bilirubin over the ULN. Subjects with borderline clinical laboratory values outside the reference range may be included in the study if the investigator deems that the values are not clinically significant.

   Note: Subjects with a history of Gilbert's syndrome may have a direct bilirubin measured and would be eligible for this study provided the direct bilirubin is <ULN.

8. The subject has a history of any lymphoproliferative disorder (such as Epstein Barr Virus-related lymphoproliferative disorder, as reported in some subjects on immunosuppressive drugs), history of lymphoma, leukemia, myeloproliferative disorders, multiple myeloma, or signs and symptoms suggestive of current lymphatic disease.
9. The subject has a history of relevant drug and/or food allergies (ie, allergy to any study drug or excipients, or any significant food allergy that could preclude a standard diet in the clinical unit).
10. The subject has a clinically significant infection currently or within 6 months of first dose of study drug (eg, those requiring hospitalization or parenteral antimicrobial therapy or opportunistic infections within the last 6 months), or a history of chronic or recurrent infectious disease.
11. The subject has other severe acute or chronic medical or psychiatric condition including recent (within the past year) or active suicidal ideation or behavior or laboratory abnormality that may increase the risk associated with study participation or investigational product administration or may interfere with the interpretation of study results and, in the judgment of the investigator, would make the subject inappropriate for entry into this study.
12. The subject has or has had symptomatic herpes zoster or herpes simplex within 12 weeks, more than one episode of local herpes zoster, or a history (single episode) of disseminated zoster.
13. The subject has a positive test result for hepatitis B surface antigen, hepatitis C virus antibody, or human immunodeficiency virus types 1 or 2 antibodies at screening.
14. The subject is a female who is pregnant or lactating.
15. The subject is a fertile male who is unwilling or unable to use a highly effective method of contraception as outlined in this protocol for the duration of the study and for at least 28 days after the last dose of investigational product.
16. The subject is unwilling or unable to comply with the lifestyle restrictions described in this protocol.
17. The subject is a smoker or has used nicotine or nicotine-containing products (eg, snuff, nicotine patch, nicotine chewing gum, mock cigarettes, or inhalers) within 6 months before the first dose of study drug.
18. The subject has a positive test result for drugs of abuse, or cotinine (indicating active current smoking) at screening or before the first dose of study drug.

DocuSign Envelope ID: 774CDD36-E051-4E09-B4BF-7A852F085DB0



19. The subject has used any prescription or over-the-counter medications (except paracetamol [up to 2 g per day]), including herbal supplements, within 14 days before the first dose of study drug. Nutritional supplements are allowed if unlikely to interfere with the study results and agreed by medical monitor and investigator.
20. The subject has consumed grapefruit or grapefruit juice, Seville orange or Seville orange-containing products (eg, marmalade), or alcohol-, caffeine-, or xanthine-containing products within 48 hours before the first dose of study drug.
21. The subject has used a known strong or moderate inhibitor or inducer of CYP1A2 within 4 weeks prior to Day 1 and through the last PK sampling point on Day 21 (only for Part C, DDI).
22. The subject will have vaccination with live virus, attenuated live virus, or any live viral components within 2 weeks prior to the first dose of study drug or is to receive these vaccines at any time during treatment or within 8 weeks following completion of study treatment.
23. The subject has a positive test result for severe acute respiratory syndrome-related coronavirus 2 (SARS-CoV-2). The subject has received the Coronavirus disease 2019 (COVID-19) vaccine within 2 weeks prior to the first dose of study drug or plans to receive a COVID-19 vaccine within 12 weeks after study drug dosing or has positive test for SARS-CoV-2 during screening or presence of COVID-19 symptoms within 4 weeks prior to Day -1.
24. The subject has undergone significant trauma or major surgery within 4 weeks of screening.
25. The subject has bleeding risk: genetic predisposition to bleeding, a hemorrhagic event in the 12 months before the start of screening, or abnormal laboratory coagulation parameters.
26. The subject has a first-degree relative with a hereditary immunodeficiency.
27. The subject has investigator site staff members directly involved in the conduct of the study and their family members, site staff members otherwise supervised by the investigator, or subjects who are sponsor employees including their family members are directly involved in the conduct of the study.
28. The subject has a history of alcohol abuse or drug addiction within the last year or excessive alcohol consumption (regular alcohol intake >21 units per week for male subjects and >14 units of alcohol per week for female subjects) (1 unit is equal to approximately ½ pint [200 mL] of beer, 1 small glass [100 mL] of wine, or 1 measure [25 mL] of spirits) or use of alcohol 48 hours before the first dose of study drug.
29. The subject is involved in strenuous activity or contact sports within 24 hours before dosing and during the study.
30. The subject has donated blood or blood products >450 mL within 30 days before the first dose of study drug.
31. The subject has received study drug in another investigational study within 30 days of dosing.

DocuSign Envelope ID: 774CDD36-E051-4E09-B4BF-7A852F085DB0



32. The subject received cytochrome P450 (CYP)/ multidrug and toxin extrusion (MATE) classes of medications within 4 weeks of first dose of INS018_055 or was likely to receive CYP/MATE classes of medications during the study.
33. In the opinion of the investigator, the subject is not suitable for entry into the study.

**STUDY TREATMENTS:**

**Part A**: Within each dose cohort, subjects will receive 1 of 2 study treatments (INS018_055 or matched placebo in a ratio of 3:1) on Day 1 according to random assignment as follows: 10 mg INS018_055 or placebo (Cohort 1), 30 mg or placebo (Cohort 2), 60 mg or placebo (Cohort 3), 90 mg or placebo (Cohort 4), and 120 mg or placebo (Cohort 5). Following the standard SAD dosing on Day 1 and at least 3 days of follow up/washout (or 5 times the estimated half-life of INS018_055 based on the observed data, whichever is longer), subjects in Cohort 4 will be re-administered the same dose of INS018_055 or matched placebo after completing a standard high-fat meal.

**Part B**: Within each dose cohort, subjects will receive 1 of 2 study treatments (INS018_055 or matched placebo at a ratio of 3:1) either QD or BID on Days 1 through 7 for all 3 cohorts according to random assignment. The dosing schedule, either QD or BID, will be decided by the SRC after review of the PK, safety, and tolerability data from the first 3 cohorts of Part A. The top dose explored in Part B will not exceed the maximum dose explored in Part A. Subsequent dose escalations in Part B will be done based on a review of the safety and tolerability data up to and including Day 11 of the preceding MAD dose cohort for at least 6 subjects and the study treatment is deemed safe by the SRC.

**Part C**: Single oral dose of 200 mg caffeine will be administered on Day 1 and Day 18. Multiple oral doses of INS018_055 at the highest well tolerated dose tested (Cohort 8 dose unless tolerability results require selection of a lower dose level) in Part B (MAD) will be administered either QD or BID on Days 5 through 18.

**STUDY PROCEDURES:**

**Pharmacokinetic Assessments and Endpoints:**

**Part A (SAD)**: Blood samples for PK analysis of INS018_055 and metabolites (INS018_063 and INS018_095) will be collected on Day 1 at the following time points: before dosing (0 hour) and at 0.25, 0.5, 1, 2, 4, 6, 8, 10, and 12 hours, on Day 2 at 24 and 36 hours; on Day 3 at 48 hours; and on Day 4 at 72 hours after administration of INS018_055.

**Part B (MAD)**: For QD dosing, blood samples for PK analysis of INS018_055 and metabolites (INS018_063 and INS018_095) will be collected at the following time points:

- Day 1: before dosing (0 hour) and at 0.25, 0.5, 1, 2, 4, 6, 8, 10, and 12 hours after dosing.
- Days 2, 3, 4, 5, and 6: before dosing.
- Day 7: before dosing (0 hour) and at 0.25, 0.5, 1, 2, 4, 6, 8, 10, 12, 24 (Day 8), 48 (Day 9), and 72 (Day 10) hours after dosing.

For BID dosing, blood samples for PK analysis of INS018_055 and metabolites (INS018_063 and INS018_095) will be collected at the following time points:

- Day 1: before the morning dosing (0 hour) and at 0.25, 0.5, 1, 2, 4, 6, 8, 10, and 12 hours after the morning dosing, which is 0 hours before the evening dosing.

DocuSign Envelope ID: 774CDD36-E051-4E09-B4BF-7A852F085DB0



- Days 2, 3, 4, 5, and 6: before the morning dosing.
- Day 7: before the evening dosing (0 hour) and at 0.25, 0.5, 1, 2, 4, 6, 8, 10, 12, 24 (Day 8), 48 (Day 9), and 72 (Day 10) hours after the evening dosing.

The following plasma PK parameters will be calculated as endpoints for INS018_055 and metabolites (INS018_063 and INS018_095) using actual sampling times rather than scheduled sampling times:

- Area under the plasma concentration versus time curve (AUC) from time 0 to the last quantifiable concentration ($AUC_{0\text{-}t}$)
- AUC from time 0 extrapolated to infinity ($AUC_{0\text{-}inf}$)
- AUC from time 0 to the time of the dosing interval ($\tau$; $AUC_{0\text{-}\tau}$)
- Accumulation ratio (AR), calculated as $AUC_{0\text{-}\tau}$ (Day 7) / $AUC_{0\text{-}\tau}$ (Day 1)
- AR calculated as $C_{max}$ (Day 7) / $C_{max}$ (Day 1)
- Maximum observed plasma concentration ($C_{max}$)
- Time to reach $C_{max}$ ($T_{max}$)
- Pre-dose concentrations on Days 1 through 7 ($C_{trough}$)
- Average concentration on Day 1 and Day 7 ($C_{av}$)
- Terminal elimination rate constant ($K_{el}$)
- Terminal elimination half-life ($t_{1/2}$)
- Apparent total body clearance (CL/F)
- Peak to trough ratio calculated as $C_{max}/C_{trough}$
- Apparent volume of distribution ($V_d/F$)
- Metabolite-to parent ratio based on AUC calculated as $AUC_{metabolite}/AUC_{parent}$
- Metabolite-to parent ratio based on $C_{max}$ calculated as $C_{max,\ metabolite}/C_{max,\ parent}$

For either QD or BID dosing cohorts, urine will be collected on Day -1 for PK analysis of INS018_055 and metabolites (INS018_063 and INS018_095) before dosing as a single void collected within 0 to 24 hours and over the following intervals after dosing: 0 to 4, 4 to 8, 8 to 12, and 12 to 24 hours on Day 1 and 0 to 4, 4 to 8, 8 to 12, 12 to 24, and 24 to 48 hours on Day 7.

The following urine PK parameters will be calculated as endpoints:

- Renal clearance (CLr)
- Fraction of the dose excreted unchanged in urine over all time intervals (0 to t) ($Fe\%_{0\text{-}t}$)
- Total amount of drug excreted unchanged in urine over all time intervals (0 to t) ($XU_{0\text{-}t}$)

DocuSign Envelope ID: 774CDD36-E051-4E09-B4BF-7A852F085DB0



**Part C (DDI):** Blood samples for PK analysis of caffeine will be collected from all subjects at the following time points on Days 1 and 18: before dosing (0 hour) and at 0.25, 0.5, 1, 2, 4, 6, 8, 10, 12, 24, 48, and 72 hours after administration of caffeine.

Blood samples for PK analysis of INS018_055 in plasma will also be collected before administration of INS018_055 on Days 5 through 18 for the assessment of steady state.

The following plasma PK parameters will be calculated as endpoints for caffeine using actual sampling times rather than scheduled sampling times:

- $AUC_{0\text{-}t}$
- $AUC_{0\text{-}inf}$
- $C_{max}$
- $T_{max}$
- $K_{el}$
- $t_{1/2}$
- CL/F
- $V_d$/F

**Biomarker Assessments and Endpoints:**
Blood concentrations of subpopulation of T-cells (CD4+ and CD8+), IL-6, TGF-β, MMP-2, MMP-9, and MMP-7 in plasma and phospho-NF-kB p65 (Ser536), phospho-Smad2 (Ser465/467), total NF-kB p65, and total Smad2 in peripheral blood mononuclear cells will be assessed.

**Metabolite Identification and Profiling Assessments and Endpoints:**
Plasma and urine samples will be collected from subjects in Cohort 8 (only) up to 24 hours after dosing on Day 7 for metabolite identification and profiling of INS018_055.

**Safety Assessments and Endpoints:**
Safety and tolerability will be assessed by the following endpoints: monitoring and recording of AEs, clinical laboratory test results (hematology, coagulation, serum chemistry, urinalysis, and fecal occult blood test), vital sign measurements, 12-lead ECG results, and physical examination findings.

**STATISTICAL ANALYSIS PLANS:**
**Sample Size:**
The total sample size of 80 subjects (40 subjects in Part A, 24 subjects in Part B, and 16 subjects in Part C) is considered sufficient for the objectives of the study. For Parts A and B, the number of subjects is based on clinical and practical considerations and not on a formal statistical power calculation.

For Part C, based on an intra-subject coefficient of variation (CV) of 21.4% for $AUC_{0\text{-}inf}$, the standard deviation of paired differences in AUC on log scale would be expected to be 0.3. A sample size of 16 evaluable subjects in Part C would give a 90% confidence interval (CI) with good precision (half width of 0.131 on log scale leading to 1.3-fold between the lower

DocuSign Envelope ID: 774CDD36-E051-4E09-B4BF-7A852F085DB0



and upper 90% CI limits). Furthermore, should there be no drug-drug interaction, then there would be 76% probability (power) that the 90% CI would fall within the 0.80 to 1.25 limits.

**Analysis Sets:**
The PK population will include subjects who receive at least 1 dose of INS018_055 (Parts A and B) or at least 1 dose of caffeine (Part C) and have sufficient concentration data to support accurate estimation of at least 1 PK parameter. Subjects who experience vomiting within 2 times the median $T_{max}$ after study drug dosing will be excluded from the PK analysis.

The pharmacodynamic (PD) population will include subjects who receive at least 1 dose of INS018_055 or placebo and have a valid baseline value and at least 1 non-missing post-baseline value for biomarker measurement.

The safety population will include all subjects who receive at least 1 dose of study treatment.

Data from subjects receiving placebo in each cohort will be pooled into 1 group for analysis.

**Pharmacokinetic Analyses:**
Plasma concentrations will be listed and summarized descriptively (number of subjects, arithmetic mean, SD, CV, median, geometric mean, geometric CV, minimum, and maximum). Plasma concentration versus actual time profiles for each subject will be presented graphically. The mean plasma concentration versus scheduled time profiles will be presented graphically. Individual urine concentration (and sample volumes) will be presented in data listings.

Plasma PK parameters derived from plasma samples using noncompartmental methods and will be summarized by treatment using descriptive statistics (number of subjects, mean, SD, CV, geometric mean, geometric CV, median, minimum, and maximum). However, $T_{max}$ will be summarized using the following descriptive statistics only: number of subjects, median, minimum, and maximum. Urine PK parameters derived from urine samples will also be summarized by treatment using descriptive statistics (number of subjects, mean, SD, CV, geometric mean, geometric CV, median, minimum, and maximum).

To evaluate the effect of food on the PK of INS018_055 and metabolites (INS018_063 and INS018_095), an analysis of variance (ANOVA) model will be performed on the natural logarithms of AUCs and $C_{max}$ to calculate the ratio of geometric means and its 90% CI between the fed cohort and the fasted cohort. Similarly, an ANOVA model will be performed on the natural logarithms of AUCs and $C_{max}$ to calculate the ratio of geometric means and its 90% CI between caffeine with INS018_055 (test) versus caffeine alone (reference). Wilcoxon Signed Rank test will be used to compare the $T_{max}$ between the fed cohort and the fasted cohort.

Dose proportionality will be tested on Days 1 and 7 using the power regression model for AUCs and $C_{max}$. Plots of AUCs and $C_{max}$ versus dose will be presented. The model is defined as:

$$\ln[\text{PK parameter}] = \beta_0 + \beta_1 \ln[\text{dose}]$$

where the PK parameter is an AUC or $C_{max}$. The null hypothesis being tested is that the AUCs and $C_{max}$ values are dose proportional, or slope ($\beta_1$) = 1.

DocuSign Envelope ID: 774CDD36-E051-4E09-B4BF-7A852F085DB0



Steady state will be tested by regression analysis of trough level concentrations of individual subjects from samples collected on Days 1 through 7. The model used to estimate the slope and corresponding 90% CI is:

$$C_{trough} = \beta_0 + \beta_1(\text{day} + \text{error})$$

Statistical evidence that the subject has not achieved steady state will be found if the slope is positive and significantly different from zero at the 5% level.

**Safety Analyses:**
Adverse events will be coded by preferred term and system organ class using the latest version of the Medical Dictionary for Regulatory Activities. All AE data will be presented in a data listing. Treatment-emergent AEs will be summarized by treatment and overall, as well as by severity and relationship to study drug. Serious AEs, AEs leading to early discontinuation, and death will also be presented in data listings.

Actual values and changes from baseline for clinical laboratory test results, vital sign measurements, and 12-lead ECG results will be summarized by treatment at each time point using descriptive statistics (number of subjects, mean, SD, median, minimum, and maximum). Shift tables will be generated for clinical laboratory test results. Frequency and percentage will be applied to summarize fecal occult blood results by visit. Clinical laboratory test results, vital sign measurements, 12-lead ECG results, and physical examination findings will be presented in data listings.

**Biomarker Analyses:**
Summaries for all biomarker analysis will be based on the PD population. Summary statistics and change and percent change from baseline for all biomarkers will be presented by visit and time point. Last recheck values collected prior to the first dose of study treatment will be considered as baseline and all rechecks will be excluded from post-dose observations in calculating summary statistics.

**DATE OF PROTOCOL:** 23 August 2022

DocuSign Envelope ID: 774CDD36-E051-4E09-B4BF-7A852F085DB0



# 1. INTRODUCTION

## 1.1 BACKGROUND

Idiopathic pulmonary fibrosis (IPF) is a fatal lung disease, characterized by distorted lung architecture and loss of respiratory function as a result of alveolar epithelial cell injury and hyperplasia, enhanced extracellular matrix (ECM) deposition, and myofibroblast activation. It has been reported that epithelial to mesenchymal transition (EMT) is one of the major drivers of fibrosis with approximately 30% of ECM-producing myofibroblasts being derived from epithelial cells through EMT (Kim et al 2006; Chapman 2011; Weigle et al 2019). Fibroblast to myofibroblast transformation (FMT) is another important contributor to IPF pathology. Activated myofibroblast promote excessive ECM deposition resulting in characteristic fibroblast foci lesions (Katzenstein and Myers 1998). The occurrence and number of fibroblast foci can be correlated with survival (Lee et al 2017).

Fibroblast foci occur in subepithelial layers close to areas of alveolar epithelial cell injury and repair suggesting that impaired epithelial-mesenchymal crosstalk contributes to the pathobiology of IPF. It is generally accepted that repetitive injury and subsequent repair of alveolar epithelial cells, in the presence or absence of local inflammation, represent a key pathogenic mechanism in IPF. This mechanism leads to aberrant growth factor activation and perpetuation of fibrotic transformation (Selman et al 2001). Transforming growth factor (TGF)-β1 (TGF-β1) is known to be a key fibrogenic driver of both EMT and FMT. Additionally, tumor necrosis factor-α (TNF-α) as an important proinflammatory cytokine, is expressed by macrophages and epithelial cells in the lungs of patients with IPF and has been shown to promote TGF-β1 induced disease progression in the scenario of IPF (Piguet et al 1993; Balogh A, Harder S, Vollandt R, et al. Intra-individual variability of caffeine elimination in healthy subjects. Int J Clin Pharmacol Ther Toxicol. 1992 Oct;30(10):383-7. Erratum in: Int J Clin Pharmacol Ther Toxicol. 1993;31(4):208.

Camara and Jarai 2010). Although several soluble mediators, such as TGF-β1 or interleukin-1b, have a clear pathogenic role in IPF and are possible therapeutic targets (Selman et al 2001), no pharmaceutical has been successful in clinical use till date. Two treatments (pirfenidone and nintedanib) targeting the biologic processes that drive fibrosis are approved for the treatment of IPF. While each attenuates the rate of lung function decline, neither halts disease progression nor affects long-term survival (King et al 2014; Richeldi et al 2014).

Although molecular characterization of IPF shows complex interdependence of various mechanisms, the Wnt pathway is consistently identified as a major activated signaling

DocuSign Envelope ID: 774CDD36-E051-4E09-B4BF-7A852F085DB0



cascade that significantly contributes to the pathogenesis of lung fibrosis (Chilosi et al 2003; Morrisey 2003; Mahmoudi et al 2009; Yamada and Masuda 2017).

The serine/threonine kinase Traf2- and Nck-interacting kinase (TNIK), is an essential activator of Wnt target genes (Mahmoudi et al 2009; Shitashige et al 2010) and interacts with the T-cell factor /lymphoid enhancer factor and β-catenin transcriptional complex, directly mediating an important signaling mechanism of the Wnt/β-catenin pathway in its downstream part (Yamada and Masuda 2017). The Wnt/β-catenin signaling pathway also partially mediates the fibrogenic effect of TGF-β (Oda et al 2016; Cao et al 2018).

Persistently activated NF-κB pathway is involved in a variety of inflammatory diseases that can especially mediate the progression of pulmonary fibrosis (Hou et al 2018). One of the upstream regulators of NF-κB signal are TRAF proteins, which are known to cooperate with TNIK (Shkoda et al 2012)

Therefore, TNIK is a feasible target of pharmacologic intervention for manipulation of the Wnt signaling pathway and engages other IPF-related signaling pathways including NF-κB and TGF-β signaling pathways. Inhibition of TNIK is expected to be potentially effective to repress the signaling events even in those cases when the upstream regulators are mutated.

INS018_055 is a potent inhibitor of the TNIK and is currently being developed by InSilico Medicine Hong Kong Limited as an orally administered potential treatment for IPF in adult humans. INS018_055 showed potent inhibition of TNIK in a biochemical enzymatic assay with a half-maximal inhibitory concentration ($IC_{50}$) value of 23 nM. Furthermore, INS018_055 inhibited few other kinases that are known to be involved in pro-fibrotic pathways (ie, Alk4, DDR1, Fms, PDGFR, TGFBR1).

INS018_055 also potently inhibited TGF-β induced expression of α-smooth muscle actin (α-SMA) and collagen I in the lung fibroblast cell line MRC-5. In an in vitro translational cell-based model, INS018_055 showed concentration dependent inhibition of TGF-β1-mediated α-SMA expression (in FMT assay) and fibronectin expression (in EMT assay) in cells derived from IPF patients, with no or minimal signs of cytotoxicity or anti-proliferative effects at the concentrations tested.

In an in vivo efficacy model of bleomycin (BLM)-induced lung fibrosis, INS018_055 demonstrated potent efficacy and prevented BLM-induced lung fibrosis dose-dependently.

DocuSign Envelope ID: 774CDD36-E051-4E09-B4BF-7A852F085DB0



An intravenous toxicity study in mice did not reveal serious acute or delayed toxicity at single doses of 1, 3, and 10 mg/kg, and oral, single-dose maximum tolerated dose studies in mice and dogs showed a good tolerability of INS018_055 in doses up to 300 mg/kg.

Pivotal 28-day toxicity studies in mice and dogs showed dose-dependent toxicity from 60 mg/kg/day in mice and 30 mg/kg/day in dogs. The lymphohematopoietic system and the gastrointestinal (GI) tract were the major target organs of toxicity. Dose-dependent increases were also seen in physis width as well as cortical and trabecular bone thickness, that may represent (extended) pharmacological effects of INS018_055 on chondro-/osteoclast activity. In addition, there was no evidence for genotoxicity with compound INS018_055 and its 2 metabolites in a valid battery of GLP-compliant in vitro tests that included bacterial reverse mutation assay in five strains of *Salmonella typhimurium* (TA97a, TA98, TA100, TA102, and TA1535) and the chromosome aberration test in Chinese Hamster Lung cell. There was also no evidence of clastogenic and/or aneugenic potential of compound INS018_055 in an GLP-compliant in vivo micronucleus test in peripheral blood reticulocytes following oral administration. Safety pharmacology studies for central nervous, respiratory, cardiovascular, or GI systems were performed in vitro or in vivo following oral administration of compound INS018_055 at pharmacological and supra-pharmacological doses. Compound INS018_055 had no apparent effects on physiological functions of the central nervous system, cardiovascular, respiratory, or GI systems. Compound INS018_055 and their 2 metabolites also did not produce significant effect on the cardiac repolarization, in vitro or in vivo.

For further information on INS018_055, please refer to the investigator’s brochure (IB) for INS018_055.

The preclinical data generated so far along with the bioinformatic analysis provides a strong rationale to pursue INS018_055 as a clinical candidate for IPF.

The overall safety profile of INS018_055 in nonclinical studies supports clinical evaluation of safety, tolerability, pharmacokinetic (PK) profile, and biomarker responses to single and multiple (up to 7 days) oral administrations of INS018_055 in the dose ranges specified in this protocol in healthy subjects. Importantly, the adverse effects observed in nonclinical studies at high multiple of exposures are monitorable in the setting of the current clinical study. All subjects will be informed of the potential risks prior to enrollment in the study.

In vitro data indicate that INS018_055 may induce the expression of the hepatic CYP1A2 enzyme, which could give rise to drug-drug interactions (DDI). Therefore, a DDI evaluation has been included in Part C of the MAD phase, using the recommended CYP1A2 index substrate caffeine.

DocuSign Envelope ID: 774CDD36-E051-4E09-B4BF-7A852F085DB0



## 1.2 RATIONALE FOR STUDY

**Part A (SAD) and Part B (MAD)**

This study is primarily designed to provide initial single and multiple ascending doses safety, tolerability, PK, and biomarkers data regarding INS018_055 in healthy subjects for future clinical studies. Analysis of urine and plasma concentrations will characterize the single and multiple dose PK of INS018_055 (including metabolites INS018_063 and INS018_095) and help to refine the dosing strategy for future studies. A food effect assessment will also be undertaken to investigate the influence of food on the safety, and PK of INS018_055.

This study has been designed as a sequential, ascending-dose study in healthy subjects. In Part A of the study, sequential ascending-dose healthy subject cohorts will be orally administered a single dose of INS018_055. In Part B, sequential once daily (QD) or twice daily (BID) dose cohorts after 7-day oral administration of INS018_055 will be assessed in healthy subjects.

Subjects in Part B will begin dosing, after safety and tolerability data (including reported adverse events [AEs], physical examination, clinical laboratory results, 12-lead electrocardiograms [ECGs], and vital signs) from at least the first 3 cohorts (Cohorts 1 to 3) in Part A are assessed and the study treatment is deemed safe and well tolerated by the safety review committee (SRC). Additionally, sufficient PK data from at least the first 3 cohorts of Part A must be obtained to model exposures in Part B. The dosing schedule, either QD or BID, will be decided by the SRC after review of the PK, safety, and tolerability data from the first 3 cohorts of Part A.

The initial multiple ascending dose (MAD) cohorts will begin dosing with the remaining single ascending dose (SAD) cohorts. The top dose explored in Part B will not exceed the maximum dose explored in Part A. Subsequent dose escalations in Part B will not occur until the safety (including AEs, physical examination, clinical laboratory results, vital signs, and 12-lead ECGs) and tolerability data up to and including Day 11 of the preceding MAD dose cohort for at least 6 subjects are assessed and the study treatment is deemed safe by the SRC.

**Part C (DDI)**

INS018_055 in vitro studies demonstrated that INS018_055 has the potential to induce CYP1A2 and published data indicated that pirfenidone was primarily metabolized via CYP1A2 with about 70%-80% of the total CYP contribution. Given that the upcoming Phase 2 study will include the IPF patients under treatment with pirfenidone and continue the treatment during the Phase 2, it is necessary to evaluate the effect of INS018_055 on

pirfenidone PK profile and then probably adjust the dose of pirfenidone to make sure the patients can still benefit from the treatment. Additionally, caffeine is the recommended CYP1A2 index substrate by FDA guidance, so this study will use caffeine instead of pirfenidone to determine the extent of CYP1A2 induction by INS018_055.

## 1.3 RATIONALE FOR DOSE SELECTION

Based on the no observed adverse effect level (NOAEL) in the most sensitive animal test species of 30 mg/kg/day in mice, a human equivalent dose (HED) was calculated and further reduced by use of a 10-fold safety factor resulting in a maximum recommended starting dose of 0.24 mg/kg, as the initial clinical dose in this study. Therefore, the clinical starting dose in Part A of the Phase 1 study was defined as 10 mg, equivalent to 0.24 mg/kg in a 60 kg adult.

The following formula was used to calculate the HED using animal NOAEL dose levels and animal weights as described in the US Food and Drug Administration Guidance for Industry: Estimating the Maximum Safe Starting Dose in Initial Clinical Trials for Therapeutics in Adult Healthy Volunteers (DHHS 2005):

$$\text{HED} = \text{animal dose (mg/kg)} \times \left[\frac{\text{animal weight (kg)}}{\text{human weight (kg)}}\right]^{0.33}$$

# 2. STUDY OBJECTIVES

## 2.1 PRIMARY OBJECTIVE

The primary objective of this study is to assess the safety and tolerability of single and multiple oral escalating doses of INS018_055 administered to healthy subjects.

## 2.2 SECONDARY OBJECTIVES

The secondary objectives of this study are:

- To determine the PK of INS018_055 following single and multiple oral escalating doses in healthy subjects.
- To assess the effect of food on the PK of INS018_055 following an oral dose.

DocuSign Envelope ID: 774CDD36-E051-4E09-B4BF-7A852F085DB0



## 2.3 EXPLORATORY OBJECTIVE

- To determine the effect of single and multiple oral escalating doses of INS018_055 on the circulating cluster of differentiation (CD)4+ and CD8+ subpopulation of T-cells (as a measure of pharmacological activity) in healthy subjects.
- To determine the effect of multiple oral escalating doses of INS018_055 on the levels of phospho-nuclear factor (NF)-kB p65 (Ser536), phospho-SMAD family member (Smad)2 (Ser465/467), interleukin (IL)-6, TGF-β, matrix metalloproteinase (MMP)-2, MMP-7, MMP-9, total NF-kB p65, and total Smad2 in healthy subjects.
- To assess the interaction potential of INS018_055 with CYP1A2 substrates.
- To characterize the metabolite profile of INS018_055 in plasma and urine following the highest dose level in the MAD study part (Cohort 8 in Part B).

# 3. STUDY DESIGN

This is a Phase 1 study of INS018_055, designed in 3-parts (A, B, and C):

- Parts A and B will be randomized, double-blind, placebo controlled single (Part A) and multiple (Part B) ascending dose study parts designed to assess the safety, tolerability, and PK of INS018_055 when administered as oral doses to healthy subjects. Additionally, the impact of food on the PK of INS018_055 will be investigated, metabolite identification and profiling of INS018_055 in plasma and urine will be also conducted following the highest dose level in the MAD study part (Cohort 8 in Part B).
- Part C will be an open-label, non-randomized, 2-period DDI study part to evaluate the effect of multiple oral doses of INS018_055 on the single-dose PK of caffeine in healthy subjects.

The study Parts A and B will consist of a screening period, check-in, a treatment period, and an end-of-study (EOS) visit. Part C will consist of a screening period, check-in, 2 treatment periods, and an EOS visit.

**Part A (SAD)**

Eight healthy subjects will be assigned to each of up to 5 sequential dose cohorts and will be randomly assigned within each dose cohort to receive INS018_055 or a matched placebo in a ratio of 3:1 (6 active, 2 placebo) on Day 1 for a total of up to approximately 40 subjects.

Subjects will be admitted to the study site on Day -1 and dosed on Day 1 after fasting (nothing to eat or drink, except water) for at least 10 hours. Water is not allowed 1 hour prior to and 1 hour after dosing. Water can be consumed ad libitum at other times. Subjects will remain fasted on Day 1 for 4 hours after dosing. Subjects will be discharged from the study site on Day 4, after the scheduled procedures and review of the Day 4 safety data by the investigator (or designee). Subjects will come back for an EOS visit on Day 8 (± 1 day).

A sentinel cohort of 2 subjects will be used to mitigate the risk of unexpected AEs not predicted by preclinical pharmacology and toxicology studies for each dosing cohort, starting with Cohort 1 (the initial dose cohort). The sentinel subjects who will be dosed in a blinded fashion (1 active, 1 placebo) and monitored for at least 1 day before the remaining 6 subjects in that cohort are dosed. Initiation of dosing of the remaining 6 subjects will depend on an initial safety review by the investigator indicating that administration of the study treatment was safe and well tolerated in the sentinel subjects.

Dose escalation will occur only after the real time PK, safety, and tolerability data (including reported AEs, physical examinations, vital signs, ECGs, and clinical laboratory results up to 48 hours after dosing) of the preceding dose cohort for at least 6 subjects are assessed and the study treatment is deemed safe and well tolerated by the SRC. As new safety and/or PK data become available, the anticipated dose escalation scheme listed in Table 5.1-1 may change following a review of the data by the SRC. Preliminary PK data from subjects in this study will help guide dose escalation to higher doses. Dose escalation to Cohorts 4 and 5 will only occur after the PK data from at least the first 2 cohorts have been assessed and deemed sufficient to model exposures for Cohorts 4 and 5.

**Food Effect Assessment (Cohort 4 in Part A only)**

Subjects in Cohort 4 of Part A will participate in a food effect arm. The actual dose level for food effect cohort is subject to change and will be confirmed by the SRC after review of data from at least the first 2 cohorts of the SAD. Following the standard SAD dosing on Day 1 and at least 3 days of follow up/washout (or 5 times the estimated half-life of INS018_055 based on the observed data, whichever is longer) (Period 1), enrolled subjects will enter Period 2 and be re-administered the same dose of INS018_055 or matched placebo (the same treatment that they received on Day 1 of Period 1) after completing a standard high-fat meal. Subjects will fast overnight (nothing to eat and drink, except water) for at least 10 hours and receive a standard high-fat breakfast 30 minutes prior to dosing. The high-fat breakfast will be consumed within approximately 30 minutes. Subjects will remain at the study site on Day 4 of Period 2 until the scheduled procedures and review of the Day 4 safety data by the investigator (or designee) are completed. The washout period of at least 3 days (or 5 times

DocuSign Envelope ID: 774CDD36-E051-4E09-B4BF-7A852F085DB0



the estimated half-life of INS018_055 based on the observed data, whichever is longer) may be extended based on available PK data from previous cohorts. The Day -1 procedures will not be repeated for Period 2.

Physical examinations, vital sign measurements, 12-lead ECGs, and clinical laboratory (hematology, coagulation, serum chemistry, urinalysis, and fecal occult blood test) evaluations will be performed at selected time points throughout the study. Subjects will also be closely monitored for AEs throughout the study. Blood samples for PK will be collected before dosing (0 hour) and up to 72 hours after study treatment administration on Day 1 for all cohorts. For biomarkers (CD4+ and CD8+ T-cells), blood samples will be collected at check-in (Day -1), Day 2, and Day 4 after administration of INS018_055. Fecal occult blood tests will be done with all stools passed at screening, check-in (Day -1), and during the 72-hour observation period.

Subjects in each cohort will be confined to study site from Day –1 until discharge on Day 4. The duration of the study, excluding screening, is approximately 10 days. For Cohort 4, where dosing and all safety and PK evaluations will be repeated with food, the duration of the study will be approximately 17 days, excluding screening.

**Part B (MAD)**

Eight healthy subjects will be assigned to each of up to 3 sequential QD or BID dose cohorts and will be randomly assigned within each dose cohort to receive INS018_055 or a matched placebo for 7 days in a ratio of 3:1 (6 active, 2 placebo), for a total of up to approximately 24 subjects.

Subjects in Part B will begin dosing, after safety and tolerability data (including reported AEs, physical examination, clinical laboratory results, 12-lead ECGs, and vital signs) from at least the first 3 cohorts (Cohorts 1 to 3) in Part A are assessed and the study treatment is deemed safe and well tolerated by the SRC. Additionally, sufficient PK data from at least the first 3 cohorts of Part A must be obtained to model exposures in Part B. The dosing schedule, either QD or BID, will be decided by the SRC after review of the PK, safety, and tolerability data from the first 3 cohorts of Part A.

The initial MAD cohorts will begin dosing with the remaining SAD cohorts. The top dose explored in Part B will not exceed the maximum dose explored in Part A. Subsequent dose escalations in Part B will not occur until the safety (including AEs, physical examination, clinical laboratory results, vital signs, and 12-lead ECGs) and tolerability data up to and including Day 11 of the preceding MAD dose cohort for at least 6 subjects are assessed and the study treatment is deemed safe by the SRC.

DocuSign Envelope ID: 774CDD36-E051-4E09-B4BF-7A852F085DB0



As new safety or PK data become available, the dose escalation scheme may change. Subjects may not receive a subsequent higher dose and may instead be administered a lower dose or may repeat the same daily dose with a different administration schema, eg, BID instead of QD dosing, to achieve lower maximum observed plasma concentration ($C_{max}$) values, particularly if the safety findings are believed to be linked to the $C_{max}$ values.

The highest dose in the MAD (either QD or BID) will be dosed in a staggered way if accrued PK data from previous cohorts predict potential exposures above the NOAEL, with 2 subjects starting dosing at least 7 days before the remaining 6 subjects; initiation of dosing of the remaining 6 subjects will depend on an initial safety review by the investigator indicating that administration of the study treatment was safe and well tolerated in the sentinel subjects.

Each subject in Part B will be admitted to the study site on Day -1 and will begin daily dosing on Day 1. Study treatment is to be administered daily at approximately the same time in the morning. For BID dosing, the second dose of study treatment will be administered approximately 12 hours after the morning dose of study treatment.

Prior to the morning dosing on Days 1 and 7 for Cohorts 6 to 8, subjects will fast (nothing to eat and drink, except water) for at least 8 hours overnight and remain fasted for 4 hours after study treatment administration. Water is not allowed 1 hour prior to and 1 hour after dosing. Water can be consumed ad libitum at other times. Water is not restricted for the evening doses when study treatment is being dosed BID. On Days 1 and 7, lunch will be provided approximately 4 hours after the morning dose, dinner will be provided approximately 10 hours after the morning dose, and a snack will be provided approximately 13 hours after the morning dose.

On Days 2 to 6 for Cohorts 6 to 8, breakfast will be provided approximately 60 minutes after study treatment administration. Standardized meals will be provided to the subjects throughout the study. Lunch will be provided approximately 4 hours after the morning dose, dinner will be provided approximately 10 hours after the morning dose, and a snack will be provided approximately 13 hours after the morning dose.

Physical examinations, vital sign measurements, 12-lead ECGs, and clinical laboratory evaluations (including liver function test results) will be performed at selected time points throughout the dosing interval. Subjects will be closely monitored for AEs throughout the study. Blood samples for PK analyses will be collected up to 72 hours after study treatment administration on Day 7. Urine samples for PK analysis will be collected before dosing (Day -1 as a single void collected within 0 to 24 hours) and over the following intervals after

DocuSign Envelope ID: 774CDD36-E051-4E09-B4BF-7A852F085DB0



dosing: 0 to 4, 4 to 8, 8 to 12, and 12 to 24 hours on Day 1 and 0 to 4, 4 to 8, 8 to 12, 12 to 24, and 24 to 48 hours on Day 7 for either QD or BID cohorts. For biomarkers (CD4+ and CD8+ T-cells, phospho-NF-kB p65 [Ser536], phospho-Smad2 [Ser465/467], IL-6, TGF-β, MMP-2, MMP 9, MMP 7, total NF-kB p65, and total Smad2), blood samples will be collected at selected time points for either QD or BID dosing cohorts. Fecal occult blood tests will be done with all stool samples at screening, check-in (Day -1), and throughout Part B on site observation periods, ie, up to Day 11. Plasma and urine samples will be collected from subjects in Cohort 8 (only) up to 24 hours after dosing on Day 7 for metabolite identification and profiling of INS018_055.

Subjects in each cohort will be confined to study site from Day –1 until discharge on Day 11, after the scheduled procedures and review of the safety data by the investigator (or designee). Subjects will return to the study site on Day 25 (± 3 days) for an EOS visit. The duration of the study, excluding screening, is approximately 29 days.

**Part C (DDI)**

Sixteen healthy subjects will be admitted to the study site on Day -1. Part C will have 2 treatment periods: Period 1 (Days 1 through 4) and Period 2 (Days 5 through 21). In Period 1, all subjects will receive a single oral dose of caffeine 200 mg in the morning on Day 1, followed by a washout from Days 2 to 4. In Period 2, subjects will receive multiple oral doses of INS018_055 (either QD or BID) for 14 days (Days 5 through 18) at the highest well tolerated dose tested (Cohort 8 dose, unless tolerability results require the use of a lower dose) in Part B (MAD), with a single oral dose of caffeine 200 mg (Day 18). On Day 18, dosing of caffeine will occur 2 hours prior to INS018_055 dosing. Subjects will stay at the study site from Day -1 until study discharge on Day 21.

Subjects in Part C will begin dosing with INS018_055 in Period 2, after safety and tolerability data (including reported AEs, physical examination, clinical laboratory results, 12-lead ECGs, and vital signs) from the 3 cohorts (Cohorts 6 to 8) in Part B are assessed and the study treatment is deemed safe and well tolerated by the SRC. Additionally, sufficient PK data from the 3 cohorts of Part B must be obtained to model exposures in Part C.

Prior to caffeine dosing on Days 1 and 18 (serial PK sampling days), subjects will fast (nothing to eat and drink, except water) for at least 8 hours overnight and remain fasted for 4 hours after study treatment administration. Water is not allowed 1 hour prior to and 1 hour after dosing. Water can be consumed ad libitum at other times. Water is not restricted for INS018_055 only dosing days (Days 5 through 17). On Days 1 and 18, lunch will be provided approximately 4 hours after the morning dose, dinner will be provided

DocuSign Envelope ID: 774CDD36-E051-4E09-B4BF-7A852F085DB0



approximately 10 hours after the morning dose, and a snack will be provided approximately 13 hours after the morning dose. On Days 5 to 17, breakfast will be provided approximately 60 minutes after study treatment administration. Standardized meals will be provided to the subjects throughout the study. Lunch will be provided approximately 4 hours after the morning dose, dinner will be provided approximately 10 hours after the morning dose, and a snack will be provided approximately 13 hours after the morning dose.

Physical examinations, vital sign measurements, 12-lead ECGs, and clinical laboratory evaluations will be performed at selected time points throughout the dosing interval. Subjects will be closely monitored for AEs throughout the study. Blood samples for PK analysis of caffeine will be collected up to 72 hours after study treatment administration on Days 1 and 18, following dosing with caffeine. Blood samples for PK analysis of INS018_055 in plasma will also be collected before administration of INS018_055 on Days 5 through 18 for the assessment of steady state. Fecal occult blood tests will be done with all stool samples at screening, check-in (Day -1), and during Period 2 from Day 5 through Day 21.

Subjects will be discharged from the study site on Day 21, after the scheduled procedures and review of the safety data by the investigator (or designee). Subjects will return to the study site on Day 24 (± 2 days) for an EOS visit. The duration of the study, excluding screening, is approximately 27 days.

DocuSign Envelope ID: 774CDD36-E051-4E09-B4BF-7A852F085DB0



## 3.1 SCHEDULE OF EVENTS

Study assessment and procedures for screening and on treatment are presented in Table 3.1-1 (Part A), Table 3.1-2 (Part B), and Table 3.1-3 (Part C).

**Table 3.1-1 Schedule of Events for Part A (SAD)**

| Phase | Screening | Check-In | Treatment Period | | | | EOS |
|---|---|---|---|---|---|---|---|
| **Procedure[a]** Day | **–28 to –2** | **–1** | **1** | **2** | **3** | **4** | **8 (± 1)** |
| Admission to clinic | | X | | | | | |
| Discharge from clinic[b] | | | | | | X | |
| Outpatient visit[c] | | | | | | | X |
| Informed consent | X | | | | | | |
| Demographics | X | | | | | | |
| Serology[d] | X | | | | | | |
| Covid-19 screening[e] | X | X | | | | | |
| Serum FSH[f] | X | | | | | | |
| Inclusion/exclusion criteria | X | X | | | | | |
| Medical history | X | X | | | | | |
| Height, weight, and BMI[g] | X | X | | | | | X |
| Physical examination[h] | X | X | | X | X | | X |
| Vital sign measurements[i] | X | X | X | X | X | X | X |
| 12-lead ECG[j] | X | X | X | X | X | X | X |
| Clinical laboratory testing[k] | X | X | | X | | X | |
| Urine drug/alcohol/cotinine screen[l] | X | X | | | | | |
| Pregnancy test[m] | X | X | | | | | X |
| Urinalysis | X | X | | X | | X | |
| Study drug administration[n] | | | X | | | | |
| PK blood sample collection[o] | | | X | X | X | X | |
| Biomarkers (CD4+ and CD8+ T-cells) sample collection[p] | | X | | X | | X | |
| Fecal occult blood test[q] | X | X | X | X | X | X | |
| Fasting period[r] | | X | X | | | X | |
| Non-fasting period[r] | | | X | X | X | X | |
| AEs[s] | | | ← X → | | | | |
| Prior/concomitant medications | ← X → | | | | | | |

Abbreviations: AE, adverse event; BMI, body mass index; CD, cluster of differentiation; Covid-19, Coronavirus disease 2019; ECG, electrocardiogram; EOS, end of study; FSH, follicle-stimulating hormone; PK, pharmacokinetic; SAD, single ascending dose; ULN, upper limit of normal.

Notes:

(a) When procedures overlap or occur at the same time point, all blood draws should follow vital signs or ECGs, and PK sampling should be timed to occur last and as close to the scheduled time window as possible.

(b) Discharge following final PK sample collection.

DocuSign Envelope ID: 774CDD36-E051-4E09-B4BF-7A852F085DB0



(c) EOS visit will occur 4 days following discharge.
(d) Serology testing will include hepatitis B surface antigen, hepatitis C virus antibody, and human immunodeficiency virus types 1 and 2 antibodies. The testing will be conducted at screening. A complete list of serology assessments is provided in Section 6.2.2.
(e) The study site will follow their standard procedures and/or local guidelines with respect to COVID□19 testing at screening, check-in, and any other time points during the study, if deemed necessary by the investigator.
(f) Females only, to confirm postmenopausal status. Further details are provided in Section 6.2.2.
(g) Height and weight will be measured, and BMI calculated at screening only. Only weight will be measured at check-in and EOS.
(h) A full physical examination will be performed at screening. A brief physical examination will be performed at check-in and EOS. Physical examinations will also be performed approximately 24 and 48 hours after dosing. Further details are provided in Section 6.2.5.
(i) Vital signs (respiratory rate, body temperature, pulse rate, and blood pressure) will be recorded at screening, check-in (Day -1). Day 1: pre-dose (within 45 min prior to dosing) and at 1 (± 30 min), 2 (± 30 min), 4 (± 30 min), 8 (± 30 min), and 12 hours (± 30 min) after dose. In addition: on Days 2, 3, 4, and EOS (Day 8). Further details on vital sign measurements are provided in Section 6.2.3.
(j) Standard 12- lead ECGs will be recorded in triplicate at screening and check-in (Day -1). Day 1: before dose and at 1 (± 15 min), 2 (± 15 min), 4 (± 15 min), and 8 hours (± 15 min) after dose. In addition: on Days 2, 3, 4, and 8 (EOS) additional ECG recordings will be taken. Further details on ECG recordings are provided in Section 6.2.4.
(k) Clinical laboratory tests (hematology, serum chemistry coagulation, and urinalysis) will be collected at screening, check-in (Day -1), Day 2, and Day 4 (day of discharge). Laboratory samples will be taken following a minimum 10-hour overnight fast. *If an increase in serum aminotransferases to > 3 × ULN occurs, repeat testing of serum chemistry laboratory tests should be conducted within 48 to 72 hours to confirm the abnormalities and to determine if they are increasing or decreasing. Close monitoring should be initiated if drug-induced liver injury is suspected, which may include repeating liver enzyme and serum bilirubin tests 2 or 3 times weekly at the investigator's discretion. Further details on clinical laboratory assessments, including a complete list of assessments, are provided in Section 6.2.2.
(l) Urine drug/alcohol/cotinine screen will occur at screening and check-in. Further details are provided in Section 6.2.2.
(m) All women will have a serum pregnancy test performed at screening, check-in, and EOS.
(n) The time of INS018_055 dosing will be called "0" hour in each cohort and occurs on days denoted with grey shading. Further dosing details are provided in Section 5.1.
(o) Blood samples for PK analysis will be collected on Day 1 before dosing (within 45 minutes prior to dosing) and at 0.25 (± 5 min), 0.5 (± 5 min), 1 (± 5 min), 2 (± 5 min), 4 (± 5 min), 6 (± 10 min), 8 (± 10 min), 10 (± 10 min), and 12 hours (± 10 min); 24 (± 30 min) and 36 hours (± 30 min) on Day 2; 48 (± 60 min) on Day 3; and 72 hours (± 60 min) on Day 4 after administration of INS018_055. Based on emerging data in each cohort, the PK blood sampling time points may be modified. Further details on the collection of blood samples for PK analysis are provided in Section 6.1.
(p) Blood sample for biomarker (CD4+ and CD8+ T-cells) analyses will be collected at check-in (Day -1), Day 2, and Day 4 after administration of INS018_055. Further details on the collection of blood samples for biomarker analyses are provided in Section 6.3.
(q) One sample from each bowel movement will be tested at screening, check-in (Day -1), and Day 1 through Day 4. Any subject with a positive fecal occult blood on Day 4 may remain at the study site for longer observation at the discretion of the investigator.
(r) Further details on fasting and non-fasting periods are provided in Section 4.3.1.1.
(s) Further details on collection and reporting of AEs are provided in Section 6.2.1.

DocuSign Envelope ID: 774CDD36-E051-4E09-B4BF-7A852F085DB0



**Table 3.1-2 Schedule of Events for Part B (MAD)**

| Phase | Screening | Check-In | Treatment Period | | | | | | | | | | | EOS |
|---|---|---|---|---|---|---|---|---|---|---|---|---|---|---|
| **Procedure[(a)]** **Day** | **–28 to –2** | **–1** | **1** | **2** | **3** | **4** | **5** | **6** | **7** | **8** | **9** | **10** | **11** | **Day 25 (± 3)** |
| Admission to clinic | | X | | | | | | | | | | | | |
| Discharge from clinic[(b)] | | | | | | | | | | | | | X | |
| Outpatient visit[(c)] | | | | | | | | | | | | | | X |
| Informed consent | X | | | | | | | | | | | | | |
| Demographics | X | | | | | | | | | | | | | |
| Serology[(d)] | X | | | | | | | | | | | | | |
| Covid-19 screening[(e)] | X | X | | | | | | | | | | | | |
| Serum FSH[(f)] | X | | | | | | | | | | | | | |
| Inclusion/exclusion criteria | X | X | | | | | | | | | | | | |
| Medical history | X | X | | | | | | | | | | | | |
| Height, weight, and BMI[(g)] | X | X | | | | | | | | | | | | X |
| Physical examination[(h)] | X | X | | X | | | X | | X | | | | X | X |
| Vital sign measurements[(i)] | X | X | X | X | X | X | X | X | X | X | X | X | X | X |
| 12-lead ECG[(j)] | X | X | X | X | | X | | X | X | X | X | | X | |
| Clinical laboratory testing[(k)] | X | X | | X | | X | | | X | X | | | X | |
| Urinalysis | X | X | | X | | X | | | X | X | | | X | |
| Urine drug/alcohol/cotinine screen[(l)] | X | X | | | | | | | | | | | | |
| Pregnancy test[(m)] | X | X | | | | | | | | | | | | X |
| Study drug administration[(n)] | | | X | X | X | X | X | X | X | | | | | |
| PK blood sample collection[(o)] | | | X | X | X | X | X | X | X | X | X | X | | |
| PK urine sample collection[(p)] | | X | X | X | | | | | X | X | X | | | |
| Biomarkers sample collection | | | | | | | | | | | | | | |
| CD4+ and CD8+ T-cells[(q)] | | X | | | | | | | X | | | | | |
| Phospho-NF-kB p65 (Ser536), phospho-Smad2 (Ser465/467), IL-6, TGF-β, MMP-2, MMP-9, MMP-7 biomarkers, total NF-kB p65, and total Smad2 [(r)] | | X | | | | | | | X | | | | | |
| Metabolite identification and profiling of INS018_055 plasma sample collection[(s)] | | | | | | | | | X | | | | | |

DocuSign Envelope ID: 774CDD36-E051-4E09-B4BF-7A852F085DB0



| Phase | Screening | Check-In | Treatment Period | | | | | | | | | | | EOS |
|---|---|---|---|---|---|---|---|---|---|---|---|---|---|---|
| **Procedure[(a)] Day** | **–28 to –2** | **–1** | **1** | **2** | **3** | **4** | **5** | **6** | **7** | **8** | **9** | **10** | **11** | **Day 25 (± 3)** |
| Metabolite identification and profiling of INS018_055 urine sample collection[(t)] | | | | | | | | | X | | | | | |
| Fecal occult blood test[(u)] | X | X | X | X | X | X | X | X | X | X | X | X | X | |
| Fasting period[(v)] | | | X | | | | | | X | | | | | |
| Non-fasting period[(v)] | | | X | X | X | X | X | X | X | X | X | X | X | X |
| AEs[(w)] | | | ◄——— X ———► | | | | | | | | | | | |
| Prior/concomitant medications | ◄——— X ———► | | | | | | | | | | | | | |

Abbreviations: AE, adverse event; BID, twice daily; BMI, body mass index; CD, cluster of differentiation; Covid-19, Coronavirus disease 2019; ECG, electrocardiogram; EOS, end of study; FSH, follicle-stimulating hormone; IL, interleukin; MAD, multiple ascending dose; MMP, matrix metalloproteinase, PK, pharmacokinetic; QD, once daily; TGF, transforming growth factor; ULN, upper limit of normal.

Notes:

(a) When procedures overlap or occur at the same time point, all blood draws should follow vital signs or ECGs, and PK sampling should be timed to occur last and as close to the scheduled time window as possible.

(b) Subjects in either the QD or BID cohorts will be discharged on Day 11 following clinical laboratory and fecal occult blood sample collection.

(c) EOS visit will occur 14-17 days following discharge.

(d) Serology testing will include hepatitis B surface antigen, hepatitis C virus antibody, and human immunodeficiency virus types 1 and 2 antibodies. The testing will be conducted at screening. A complete list of serology assessments is provided in Section 6.2.2.

(e) The clinic will follow their standard procedures and/or local guidelines with respect to COVID□19 testing at screening, check-in, and any other time points during the study, if deemed necessary by the investigator.

(f) Females only, to confirm postmenopausal status. Further details are provided in Section 6.2.2.

(g) Height and weight will be measured, and BMI calculated at screening only. Only weight will be measured at check-in and EOS.

(h) A full physical examination will be performed at screening. A brief physical examination will be performed at check-in and EOS. Brief physical examinations will also be performed on Days 2, 5, 7, and 11. Further details are provided in Section 6.2.5.

(i) Vital signs (respiratory rate, body temperature, pulse rate, and blood pressure) will be recorded at screening, check-in (Day -1), Days 1 to 11, and EOS (Day 25). Days 1 and 7: pre-dose (within 45 minutes prior to dosing) and at 1 (± 30 min), 2 (± 30 min), 4 (± 30 min), 8 (± 30 min), 12 (± 30 min), and 24 hours (± 60 min) after dose. Vital sign measurements will be obtained prior to the morning dose on Days 2 to 6. Vital sign measurements taken on Days 9, 10, 11, and EOS (Day 25) will be performed in the morning. Further details on vital sign measurements are provided in Section 6.2.3.

(j) Standard 12-lead ECGs will be recorded in triplicate at screening and check-in (Day -1); single ECGs will be collected at all other specified visits. Days 1 and 7: pre-dose and at 1 (± 15 min), 2 (± 15 min), 4 (± 15 min), 8 (± 15 min), and 12 hours (± 15 min) after dose. In addition, ECGs will be obtained prior to the morning dose on Days 2, 4, and 6; on the mornings of Days 8 and 9 (12 and 36 hours after the evening dose on Day 7); and before discharge on Day 11. Further details on ECG recordings are provided in Section 6.2.4.

(k) Clinical laboratory tests (hematology, serum chemistry, coagulation, and urinalysis) will be collected at screening, check-in (Day -1), Days 2, 4, 7, 8, and on the day of discharge (Day 11). Laboratory samples will be taken following a minimum 8-hour overnight fast. *If an increase in serum aminotransferases to >3 × ULN occurs, repeat testing of serum chemistry laboratory tests should be conducted within 48 to 72 hours to confirm the abnormalities and to determine

DocuSign Envelope ID: 774CDD36-E051-4E09-B4BF-7A852F085DB0



if they are increasing or decreasing. Close monitoring should be initiated if drug-induced liver injury is suspected, which may include repeating liver enzyme and serum bilirubin tests 2 or 3 times weekly at the investigator's discretion. Further details on clinical laboratory assessments, including a complete list of assessments, are provided in Section 6.2.2.

(l) Urine drug/alcohol/cotinine screen will occur at screening and check-in. Further details are provided in Section 6.2.2.

(m) All women will have a serum pregnancy test performed at screening, check-in, and EOS.

(n) The time of INS018_055 dosing will be called "0" hour in each cohort, except for BID cohorts where the second dose will be administered 12 hours after the first dose and occurs on days denoted with grey shading. Further dosing details are provided in Section 5.1.

(o) For QD dosing, blood samples for PK analysis will be collected on Day 1: before dosing (0 hour) (within 45 minutes prior to dosing) and at 0.25 (± 5 min), 0.5 (± 5 min), 1 (± 5 min), 2 (± 5 min), 4 (± 5 min), 6 (± 10 min), 8 (± 10 min), 10 (± 10 min), and 12 hours (± 10 min) after dosing; Days 2, 3, 4, 5, and 6: before dosing, following at least an 8-hour fasting period before breakfast; Day 7: before dosing (0 hour) (within 45 minutes prior to dosing) and at 0.25 (± 5 min), 0.5 (± 5 min), 1 (± 5 min), 2 (± 5 min), 4 (± 5 min), 6 (± 10 min), 8 (± 10 min), 10 (± 10 min), 12 (± 10 min), 24 (± 30 min), 48 (± 60 min), and 72 hours (± 60 min) after dosing. For BID dosing, blood samples for PK analysis will be collected on Day 1: before the morning dosing (0 hour) (within 45 minutes prior to dosing) and at 0.25 (± 5 min), 0.5 (± 5 min), 1 (± 5 min), 2 (± 5 min), 4 (± 5 min), 6 (± 10 min), 8 (± 10 min), 10 (± 10 min), and 12 hours (± 10 min) after the morning dosing; Days 2, 3, 4, 5, and 6: before the morning dosing, following at least an 8-hour fasting period, before breakfast; Day 7: before the evening dosing (0 hour) (within 45 minutes prior to dosing) and at 0.25 (± 5 min), 0.5 (± 5 min), 1 (± 5 min), 2 (± 5 min), 4 (± 5 min), 6 (± 10 min), 8 (± 10 min), 10 (± 10 min), 12 (± 10 min), 24 (± 30 min), 48 (± 60 min), and 72 hours (± 60 min) after the evening dosing. Further details on the collection of blood samples for PK analysis are provided in Section 6.1.

(p) Urine samples for PK analysis will be collected before dosing (collected on Day -1 as a single void collected within 0 to 24 hours) and over the following intervals after dosing: 0 to 4, 4 to 8, 8 to 12, and 12 to 24 hours on Day 1 and 0 to 4, 4 to 8, 8 to 12, 12 to 24, and 24 to 48 hours on Day 7 for either QD or BID cohorts.

(q) For either QD or BID dosing cohorts, blood sample for biomarker (CD4+ and CD8+ T-cells) analyses will be collected at check-in (Day -1) and 6 hours after the morning dose on Day 7. Further details on the collection of blood samples for biomarkers analyses are provided in Section 6.3.

(r) For either QD or BID dosing cohorts, blood sample for additional biomarkers (phospho-NF-kB p65 [Ser536], phospho-Smad2 [Ser465/467], IL-6, TGF-β, MMP-2, MMP 9, MMP 7, total NF-kB p65, and total Smad2) analyses will be collected at check-in (Day -1) and 6 hours after the morning dose on Day 7. Further details on the collection of blood samples for biomarkers analyses are provided in Section 6.3.

(s) For subjects in Cohort 8 (only), plasma samples for metabolite identification and profiling of INS018_055 will be collected on Day 7 at the following time points: before dosing (0 hours) (within 45 minutes prior to dosing) and at 0.25 (± 5 min), 0.5 (± 5 min), 1 (± 5 min), 2 (± 5 min), 4 (± 5 min), 6 (± 10 min), 8 (± 10 min), 10 (± 10 min), 12 (± 10 min), and 24 hours (± 30 min).

(t) For subjects in Cohort 8 (only), urine samples for metabolite identification and profiling of INS018_055 will be collected over the following intervals after dosing 0 to 4, 4 to 8, 8 to 12, 12 to 24 hours on Day 7.

(u) One sample from each bowel movement will be tested at screening, check-in (Day -1), and throughout Part B on site observation periods, ie, up to Day 11. Any subject with a positive fecal occult blood test on Day 11 may remain in the study site for longer observation at the discretion of the investigator.

(v) Further details on fasting and non-fasting periods are provided in Section 4.3.1.1.

(w) Further details on collection and reporting of AEs are provided in Section 6.2.1.

DocuSign Envelope ID: 774CDD36-E051-4E09-B4BF-7A852F085DB0



**Table 3.1-3 Schedule of Events for Part C (DDI)**

| Phase | Screening | Check-in | Treatment Period 1 | | | | Treatment Period 2 | | | | | | | | | | | | | | | | | EOS |
|---|---|---|---|---|---|---|---|---|---|---|---|---|---|---|---|---|---|---|---|---|---|---|---|---|
| **Procedure[(a)] Day** | **–28 to –2** | **–1** | **1** | **2** | **3** | **4** | **5** | **6** | **7** | **8** | **9** | **10** | **11** | **12** | **13** | **14** | **15** | **16** | **17** | **18** | **19** | **20** | **21** | **24 (± 2)** |
| Admission to clinic | | X | | | | | | | | | | | | | | | | | | | | | | |
| Discharge from clinic[(b)] | | | | | | | | | | | | | | | | | | | | | | | X | |
| Outpatient visit[(c)] | | | | | | | | | | | | | | | | | | | | | | | | X |
| Informed consent | X | | | | | | | | | | | | | | | | | | | | | | | |
| Demographics | X | | | | | | | | | | | | | | | | | | | | | | | |
| Serology[(d)] | X | | | | | | | | | | | | | | | | | | | | | | | |
| Covid-19 screening[(e)] | X | X | | | | | | | | | | | | | | | | | | | | | | |
| Serum FSH[(f)] | X | | | | | | | | | | | | | | | | | | | | | | | |
| Inclusion/exclusion criteria | X | X | | | | | | | | | | | | | | | | | | | | | | |
| Medical history | X | X | | | | | | | | | | | | | | | | | | | | | | |
| Height, weight, and BMI[(g)] | X | X | | | | | | | | | | | | | | | | | | | | | | X |
| Physical examination[(h)] | X | X | | | | X | | | | | | | X | | | | | | | X | | | X | X |
| Vital sign measurements[(i)] | X | X | X | X | X | X | X | X | X | X | X | X | X | X | X | X | X | X | X | X | X | X | X | X |
| 12-lead ECG[(j)] | X | X | X | | | | X | | | | | | X | | | | | | | X | | | X | |
| Clinical laboratory testing[(k)] | X | X | | X | | | X | X | | | | | | X | | | | | | X | X | | X | |
| Urinalysis | X | X | | X | | | X | X | | | | | | X | | | | | | X | X | | X | |
| Urine drug/alcohol/cotinine screen[(l)] | X | X | | | | | | | | | | | | | | | | | | | | | | |
| Pregnancy test[(m)] | X | X | | | | | | | | | | | | | | | | | | | | | | X |
| Caffeine administration[(n)] | | | X | | | | | | | | | | | | | | | | | X | | | | |
| INS018_055 administration[(o)] | | | | | | | X | X | X | X | X | X | X | X | X | X | X | X | X | X | | | | |
| Caffeine PK blood sample collection[(p)] | | | X | X | X | X | | | | | | | | | | | | | | X | X | X | X | |

DocuSign Envelope ID: 774CDD36-E051-4E09-B4BF-7A852F085DB0



| Phase | Screening | Check-in | Treatment Period 1 | | | | Treatment Period 2 | | | | | | | | | | | | | | | | | EOS |
|---|---|---|---|---|---|---|---|---|---|---|---|---|---|---|---|---|---|---|---|---|---|---|---|---|
| **Procedure[(a)] Day** | **–28 to –2** | **–1** | **1** | **2** | **3** | **4** | **5** | **6** | **7** | **8** | **9** | **10** | **11** | **12** | **13** | **14** | **15** | **16** | **17** | **18** | **19** | **20** | **21** | **24 (± 2)** |
| INS018_055 PK blood sample collection[(q)] | | | | | | | X | X | X | X | X | X | X | X | X | X | X | X | X | X | | | | |
| Fecal occult blood test[(r)] | X | X | | | | | X | X | X | X | X | X | X | X | X | X | X | X | X | X | X | X | X | |
| Fasting period[(s)] | | | X | | | | | | | | | | | | | | | | | X | | | | |
| Non-fasting period[(s)] | | | X | X | X | X | X | X | X | X | X | X | X | X | X | X | X | X | X | X | X | X | X | |
| AEs[(t)] | | | ←——————— X ———————→ | | | | | | | | | | | | | | | | | | | | | |
| Prior/concomitant medications | ←——————— X ———————→ | | | | | | | | | | | | | | | | | | | | | | | |

Abbreviations: AE, adverse event; BMI, body mass index; Covid-19, Coronavirus disease 2019; DDI, drug-drug interaction; ECG, electrocardiogram; EOS, end of study; FSH, follicle-stimulating hormone; PK, pharmacokinetic; ULN, upper limit of normal.

Notes:

[(a)] When procedures overlap or occur at the same time point, all blood draws should follow vital signs or ECGs, and PK sampling should be timed to occur last and as close to the scheduled time window as possible.

[(b)] Discharge following final PK sample collection.

[(c)] Follow-up/EOS visit will occur 3-5 days following discharge.

[(d)] A complete list of serology assessments is provided in Section 6.2.2.

[(e)] The clinic will follow their standard procedures and/or local guidelines with respect to COVID□19 testing at screening, check-in, and any other time points during the study, if deemed necessary by the investigator.

[(f)] Females only, to confirm postmenopausal status. Further details are provided in Section 6.2.2.

[(g)] Height and weight will be measured, and BMI calculated at screening only. Only weight will be measured at check-in and EOS.

[(h)] A full physical examination will be performed at screening. A brief physical examination will be performed at check-in and EOS. Brief physical examinations will also be performed on Days 4, 11, 18, and 21. Further details are provided in Section 6.2.5.

[(i)] Vital signs (respiratory rate, body temperature, pulse rate, and blood pressure) will be recorded at screening, check-in (Day -1), Days 1 to 21, and EOS (Day 24). Days 1 and 18: pre-dose (within 45 minutes prior to dosing) and at 1 (± 30 min), 2 (± 30 min), 4 (± 30 min), 8 (± 30 min), 12 (± 30 min), and 24 hours (± 60 min) after caffeine dose. Vital sign measurements will be obtained prior to the morning dose of INS018_055 on Days 5 to 17. Vital sign measurements taken on Days 2 to 4, Days 19 to 21, and EOS (Day 24) will be performed in the morning. Further details on vital sign measurements are provided in Section 6.2.3.

[(j)] Standard 12-lead ECGs will be recorded in triplicate at screening and check-in (Day -1); single ECGs will be collected at all other specified visits. Days 1 and 18: pre-dose and at 1 (± 15 min), 2 (± 15 min), 4 (± 15 min), 8 (± 15 min), and 12 hours (± 15 min) after the caffeine dose. In addition, ECGs will be obtained prior to the morning dose of INS018_055 on Days 5 and 11; and before discharge on Day 21. Further details on ECG recordings are provided in Section 6.2.4.

[(k)] Clinical laboratory tests (hematology, serum chemistry, coagulation, and urinalysis) will be collected at screening, check-in (Day -1), Days 2, 5, 6, 12, 18, 19, and on the day of discharge (Day 21). Laboratory samples will be taken following a minimum 8-hour overnight fast. *If an increase in serum

DocuSign Envelope ID: 774CDD36-E051-4E09-B4BF-7A852F085DB0



aminotransferases to >3 × ULN occurs, repeat testing of serum chemistry laboratory tests should be conducted within 48 to 72 hours to confirm the abnormalities and to determine if they are increasing or decreasing. Close monitoring should be initiated if drug-induced liver injury is suspected, which may include repeating liver enzyme and serum bilirubin tests 2 or 3 times weekly at the investigator's discretion. Further details on clinical laboratory assessments, including a complete list of assessments, are provided in Section 6.2.2.

(l) Urine drug/alcohol/cotinine screen will occur at screening and check-in. Further details are provided in Section 6.2.2.

(m) All women will have a serum pregnancy test performed at screening, check-in, and EOS.

(n) The time of caffeine dosing will be called "0" hour in each period and occurs on days denoted with grey shading. On Day 18, dosing of caffeine will occur 2 hours prior to INS018_055 dosing. Further dosing details are provided in Section 5.1.

(o) Further details on dosing of INS018_055 are provided in Section 5.1.

(p) Blood samples for PK analysis of caffeine will be collected on Days 1 and 18: before dosing (0 hour) (within 45 minutes prior to dosing) and at 0.25 (± 5 min), 0.5 (± 5 min), 1 (± 5 min), 2 (± 5 min), 4 (± 5 min), 6 (± 10 min), 8 (± 10 min), 10 (± 10 min), 12 (± 10 min), 24 (± 30 min), 48 (± 60 min), and 72 hours (± 60 min) after caffeine dosing.

(q) Blood samples for PK analysis of INS018_055 will be collected before administration of INS018_055 during Period 2, from Days 5 through 18 for the analysis of INS018_055 steady state. Further details on the collection of blood samples for PK analysis are provided in Section 6.1.

(r) One sample from each bowel movement will be tested at screening, check-in (Day -1), and Day 5 through Day 21. Any subject with a positive fecal occult blood test on Day 21 may remain in the study site for longer observation at the discretion of the investigator.

(s) Further details on fasting and non-fasting periods are provided in Section 4.3.1.1.

(t) Further details on collection and reporting of AEs are provided in Section 6.2.1.

DocuSign Envelope ID: 774CDD36-E051-4E09-B4BF-7A852F085DB0



# 4. STUDY POPULATION

Approximately 80 male and female subjects (40 subjects in Part A, 24 subjects in Part B, and 16 subjects in Part C of the study) will be enrolled across 1 center in New Zealand. Sample size determination is discussed in Section 7.1.

## 4.1 INCLUSION CRITERIA

Each subject must meet all of the following criteria to be enrolled in this study:

1. The subject is a male or female 18 to 55 years of age, inclusive.
2. The subject has a body mass index 18 to 32 kg/m$^2$, inclusive, and a total body weight ≥50 kg, inclusive, at screening.
3. The subject is considered by the investigator to be in good general health as determined by medical history, clinical laboratory test results, vital sign measurements, 12-lead ECG results, and physical examination findings at screening.
4. Female subjects of childbearing potential must be non-pregnant and non-lactating and must use one of the methods of contraception listed below for the duration of the treatment until at least 28 days after the last dose of the study drug, or be surgically sterile (ie, hysterectomy, bilateral tubal ligation, or bilateral oophorectomy) or postmenopausal (defined as amenorrhea 12 consecutive months and documented plasma follicle-stimulating hormone level >40 IU/mL). Female subjects must have a negative pregnancy test at screening and before the first dose of study drug.

   Highly effective methods of contraception are those that result in a failure rate of less than 1% per year when used consistently. Examples are provided below:

   a. Implant contraceptive (eg, Jadelle®)
   b. Intrauterine device (IUD) containing either copper or levonorgestrel (eg, Mirena®)
   c. Male sterilization with absence of sperm in the post-vasectomy ejaculate

   OR an effective method that results in a failure rate of less than 5% to 10% per year. Examples are provided below:

   d. Injectable contraceptive (eg, Depo Provera)
   e. Oral contraceptive pill (combined hormonal contraceptive pill or progestogen-only ‘mini-pill’)

DocuSign Envelope ID: 774CDD36-E051-4E09-B4BF-7A852F085DB0



f. Vaginal contraceptive ring (eg, NuvaRing®)

Female subjects must also agree not to donate eggs, from dosing until at least 28 days after the last dose of study drug.

A male subject and his female partner who is of childbearing potential must agree to use one of the methods of contraception listed above for the duration of the treatment until at least 28 days after the last dose of the study drug. A male subject must also agree not to donate sperm, for the duration of the treatment until at least 28 days after the last dose of the study drug.

5. The subject agrees to comply with all protocol requirements.

6. The subject is able to provide written informed consent.

## 4.2 EXCLUSION CRITERIA

Subjects meeting any of the following criteria will be excluded from the study:

1. The subject has current evidence or history of clinically significant hematological, renal, endocrine, pulmonary, GI, cardiovascular, hepatic, psychiatric, neurologic, or allergic disease (including drug allergies, but excluding untreated, asymptomatic, seasonal allergies at time of dosing).

2. The subject has any condition possibly affecting drug absorption (eg, gastrectomy).

3. The subject has a history of cancer with the exception of adequately treated basal cell or squamous cell carcinoma of the skin.

4. The subject has supine blood pressure (BP) >140 mm Hg (systolic) or >90 mm Hg (diastolic), following at least 5 minutes of supine rest. If BP is >140 mm Hg (systolic) or >90 mm Hg (diastolic), the BP should be repeated 2 more times and the average of the 3 BP values should be used to determine the subject's eligibility at screening.

5. The subject has 12-lead ECG demonstrating corrected QT interval by Fridericia (QTcF) >450 msec, or a QRS interval >120 msec at screening. If QTcF exceeds 450 msec, or QRS interval exceeds 120 msec, the ECG should be repeated 2 more times and the average of the 3 QTcF (or QRS interval) values should be used to determine the subject's eligibility.

DocuSign Envelope ID: 774CDD36-E051-4E09-B4BF-7A852F085DB0



6. The subject has ANY of the following abnormalities in clinical laboratory tests at screening, as assessed by the study-specific laboratory and confirmed by a single repeat, if deemed necessary:

   a. Serum creatinine (SCr) level above the upper limit of normal (ULN) or an estimated glomerular filtration rate (GFR) value <80 mL/min/1.73 $m^2$ calculated with the Chronic Kidney Disease Epidemiology Collaboration (CKD-EPI) formula and the absence of protein in urine, at screening.

   b. Aspartate aminotransferase (AST) or alanine aminotransferase (ALT) values more than >1.5 × ULN.
   c. Fasting glucose >110 mg/dL (6.1 mmol/L).
   d. Total bilirubin >1.5 × ULN.
   e. Hematological values outside the normal reference range for local laboratory results.
   f. Positive fecal occult blood test at screening or at check-in (Day -1).

7. The subject has any medical history of disease that has the potential to cause a rise in total bilirubin over the ULN. Subjects with borderline clinical laboratory values outside the reference range may be included in the study if the investigator deems that the values are not clinically significant.

   Note: Subjects with a history of Gilbert's syndrome may have a direct bilirubin measured and would be eligible for this study provided the direct bilirubin is <ULN.

8. The subject has a history of any lymphoproliferative disorder (such as Epstein Barr Virus-related lymphoproliferative disorder, as reported in some subjects on immunosuppressive drugs), history of lymphoma, leukemia, myeloproliferative disorders, multiple myeloma, or signs and symptoms suggestive of current lymphatic disease.

9. The subject has a history of relevant drug and/or food allergies (ie, allergy to any study drug or excipients, or any significant food allergy that could preclude a standard diet in the clinical unit).

10. The subject has a clinically significant infection currently or within 6 months of first dose of study drug (eg, those requiring hospitalization or parenteral antimicrobial therapy or opportunistic infections), or a history of chronic or recurrent infectious disease.

11. The subject has other severe acute or chronic medical or psychiatric condition including recent (within the past year) or active suicidal ideation or behavior or laboratory abnormality that may increase the risk associated with study participation or

DocuSign Envelope ID: 774CDD36-E051-4E09-B4BF-7A852F085DB0



investigational product administration or may interfere with the interpretation of study results and, in the judgment of the investigator, would make the subject inappropriate for entry into this study.

12. The subject has or has had symptomatic herpes zoster or herpes simplex within 12 weeks, more than one episode of local herpes zoster, or a history (single episode) of disseminated zoster.

13. The subject has a positive test result for hepatitis B surface antigen, hepatitis C virus antibody, or human immunodeficiency virus (HIV) types 1 or 2 antibodies at screening.

14. The subject is a female who is pregnant or lactating.

15. The subject is a fertile male who is unwilling or unable to use a highly effective method of contraception as outlined in this protocol for the duration of the study and for at least 28 days after the last dose of investigational product.

16. The subject is unwilling or unable to comply with the lifestyle restrictions described in this protocol (Section 4.3.1).

17. The subject is a smoker or has used nicotine or nicotine-containing products (eg, snuff, nicotine patch, nicotine chewing gum, mock cigarettes, or inhalers) within 6 months before the first dose of study drug.

18. The subject has a positive test result for drugs of abuse or cotinine (indicating active current smoking) at screening or before the first dose of study drug.

19. The subject has used any prescription or over-the-counter medications (except paracetamol [up to 2 g per day]), including herbal supplements, within 14 days before the first dose of study drug. Nutritional supplements are allowed if unlikely to interfere with the study results and agreed by medical monitor and investigator.

20. The subject has consumed grapefruit or grapefruit juice, Seville orange or Seville orange-containing products (eg, marmalade), or alcohol-, caffeine-, or xanthine-containing products within 48 hours before the first dose of study drug.

21. The subject has used a known strong or moderate inhibitor or inducer of CYP1A2 within 4 weeks prior to Day 1 and through the last PK sampling point on Day 21 (only for Part C, DDI).

DocuSign Envelope ID: 774CDD36-E051-4E09-B4BF-7A852F085DB0



22. The subject will have vaccination with live virus, attenuated live virus, or any live viral components within the 2 weeks prior to the first dose of study drug or is to receive these vaccines at any time during treatment or within 8 weeks following completion of study treatment.

23. The subject has a positive test result for severe acute respiratory syndrome-related coronavirus 2 (SARS-CoV-2). The subject has received the Coronavirus disease 2019 (COVID-19) vaccine within 2 weeks prior to the first dose of study drug or plans to receive a COVID-19 vaccine within 12 weeks after study drug dosing or has positive test for SARS-CoV-2 during screening or presence of COVID-19 symptoms within 4 weeks prior to Day -1.

24. The subject has undergone significant trauma or major surgery within 4 weeks of screening.

25. The subject has a bleeding risk: genetic predisposition to bleeding, a hemorrhagic event in the 12 months before the start of screening, or abnormal laboratory coagulation parameters.

26. The subject has a first-degree relative with a hereditary immunodeficiency.

27. The subject has investigator site staff member directly involved in the conduct of the study and their family members, site staff member otherwise supervised by the investigator, or subjects who are sponsor employees including their family members are directly involved in the conduct of the study.

28. The subject has a history of alcohol abuse or drug addiction within the last year or excessive alcohol consumption (regular alcohol intake >21 units per week for male subjects and >14 units of alcohol per week for female subjects) (1 unit is equal to approximately ½ pint [200 mL] of beer, 1 small glass [100 mL] of wine, or 1 measure [25 mL] of spirits) or use of alcohol 48 hours before the first dose of study drug.

29. The subject is involved in strenuous activity or contact sports within 24 hours before dosing and during the study.

30. The subject has donated blood or blood products >450 mL within 30 days before the first dose of study drug.

31. The subject has received study drug in another investigational study within 30 days of dosing.

DocuSign Envelope ID: 774CDD36-E051-4E09-B4BF-7A852F085DB0



32. The subject received cytochrome P450 (CYP)/ multidrug and toxin extrusion (MATE) classes of medications within 4 weeks of first dose of INS018_055 or was likely to receive CYP/MATE classes of medications during the study.

33. In the opinion of the investigator, the subject is not suitable for entry into the study.

## 4.3 OTHER SCREENING CONSIDERATIONS

### 4.3.1 Lifestyle Restrictions

#### 4.3.1.1 Meals and Dietary Requirements

1. Subjects are not permitted to consume grapefruit or grapefruit juice, Seville orange or Seville orange containing products (eg, marmalade) within 48 hours before the first dose of study drug treatment until study discharge.

2. During Part A, subjects are required to fast (nothing to eat or drink, except water) for at least 10 hours prior to each dose throughout the study and remain fasted until 4 hours after study treatment administration on Day 1. Participants may not drink water 1 hour prior to and 1 hour after study treatment administration, except with dosing. Water may be consumed ad libitum at other times. A standard lunch will be served 4 hours after the morning dose, a standard dinner will be served 10 hours after the morning dose, and a light snack will be served approximately 13 hours after the morning dose. All meals on Day 1 must be identical for Part A.

3. For the fed portion of Cohort 4 (Part A), study treatment administration will occur after subjects complete a standard high-fat meal. Subjects will fast (nothing to eat and drink, except water) for at least 10 hours overnight and will receive a standard high-fat breakfast 30 minutes prior to dosing. The high-fat breakfast will be consumed within approximately 30 minutes. Subjects will fast until 4 hours after study treatment administration.

4. During Part B, for morning dosing (either QD or BID), subjects will fast (nothing to eat and drink, except water) for at least 8 hours overnight on serial PK sampling days (Days 1 and 7) and remain fasted for 4 hours after study treatment administration. Water is not allowed 1 hour prior to and 1 hour after dosing. Water can be consumed ad libitum at other times. Water is not restricted for the evening doses when study treatment is administered as BID. On Days 1 and 7, lunch will be provided approximately 4 hours after the morning dose, dinner will be provided approximately 10 hours after the morning

DocuSign Envelope ID: 774CDD36-E051-4E09-B4BF-7A852F085DB0



dose, and a snack will be provided approximately 13 hours after the morning dose. Meals provided on Days 1 and 7 must be identical in content.

5. On Days 2 to 6 for all Part B cohorts, breakfast will be provided approximately 60 minutes after study treatment administration. Lunch will be provided approximately 4 hours after the morning dose, dinner will be provided approximately 10 hours after the morning dose, and a snack will be provided approximately 13 hours after the morning dose. Standardized meals will be provided to the subjects on Days 2 to 6.

6. During Part C, subjects will be required to fast (nothing to eat or drink except water) for at least 8 hours prior to dosing on serial PK sampling days (Days 1 and 18), and subjects must remain fasted until 4 hours after study treatment administration. Breakfast will be provided approximately 60 minutes after study treatment administration on INS018_055 only dosing days. Subjects may not drink water 1 hour before and 1 hour after study treatment administration, except with dosing. Water may be consumed ad libitum at other times.

7. During Part C, the food content of meals on serial PK sampling days (Days 1 and 18) should be the same, and must not contain curcumin, which is present in Asian spices such as turmeric and cumin (curcumin is known to inhibit CYP1A2 and increase the exposure of caffeine). The content of meals may vary on other days. Lunch will be provided approximately 4 hours after dosing, dinner will be provided approximately 10 hours after dosing, and a light snack will be provided approximately 13 hours after dosing.

#### 4.3.1.2 Caffeine and Alcohol

1. Subjects will abstain from ingesting caffeine- or xanthine containing products (eg, coffee, tea, cola drinks, and chocolate) for 48 hours before dosing and for the duration of the confinement period of the study.

2. Subjects will abstain from alcohol for 48 hours prior to study drug administration, during confinement at the study site until discharge, and for 48 hours prior to ambulatory visits.

#### 4.3.1.3 Activity

1. Subjects should refrain from strenuous exercise within 24 hours prior to admission to the study site and for the duration of the study. Subjects may participate in light recreational activities (eg, watching television, reading) during study.

DocuSign Envelope ID: 774CDD36-E051-4E09-B4BF-7A852F085DB0



2. Subjects are required to remain in the clinical facility from Day -1 until the end of confinement.

3. Subjects will be advised not to donate blood or plasma for at least 3 months after the last study treatment administration.

4. Subjects should maintain a semi-recumbent position for at least 4 hours after the dose on Day 1 (Part A), Days 1 and 7 (Part B), and Days 1 and 18 (Part C) except as required for study procedures. On Days 2 through 6 (Part B) and Days 2 through 17 (Part C), subjects should maintain an upright (seated or standing) position for at least 1 hour after the dose. Subjects must be under constant supervision during the upright position time.

#### 4.3.1.4 Contraception

All fertile female subjects who are, in the opinion of the investigator, sexually active and at risk of pregnancy and fertile male subjects with partners of childbearing potential, must agree to use a highly effective method of contraception consistently and correctly for the duration of the active treatment period and for at least 28 days after the last dose of study drug. The investigator or his/her designee, in consultation with the subject, will confirm the subject has selected the most appropriate method of contraception for the individual subject and his female partner from the permitted list of contraception methods (see below) and will confirm that the subject has been instructed in its consistent and correct use. At the time of informed consent, the investigator or designee will inform the subject of the need to use highly effective contraception consistently and correctly and document the conversation and the subject's affirmation in the subject's chart. In addition, the investigator or designee will instruct the subject to call immediately if the selected contraception method is discontinued by the subject or partner or if pregnancy is known or suspected in the subject's partner.

Highly effective methods of contraception are those that result in a failure rate of less than 1% per year when used consistently as follows:

1. Implant contraceptive (eg, Jadelle)

2. IUD containing either copper or levonorgestrel (eg, Mirena)

3. Male sterilization with absence of sperm in the post-vasectomy ejaculate

4. Female sterilization (eg, bilateral tubal ligation ['clipping or tying tubes'] or hysterectomy)

DocuSign Envelope ID: 774CDD36-E051-4E09-B4BF-7A852F085DB0



OR an effective method that results in a failure rate of less than 5% to 10% per year as follows:

5. Injectable contraceptive (eg, Depo Provera)
6. Oral contraceptive pill (combined hormonal contraceptive pill or progestogen-only ‘mini-pill’)
7. Vaginal contraceptive ring (eg, NuvaRing)

NOTE: Sexual abstinence, defined as completely and persistently refraining from all heterosexual intercourse (including during the entire period of risk associated with the study treatments) may obviate the need for contraception ONLY if this is the preferred and usual lifestyle of the subject.

Female subjects must also agree not to donate eggs and male subjects must agree not to donate sperm, for the duration of the active treatment period and for at least 28 days after the last dose of study drug.

If a pregnancy occurs in a female subject or female partner of male subject, it must be reported to the investigator as soon as possible. The female subject or female partner will be asked to give consent for her information and her infant’s information to be collected for monitoring purposes.

## 4.4 WITHDRAWAL OF SUBJECTS FROM THE STUDY

### 4.4.1 Reasons for Withdrawal

Subjects can withdraw consent and discontinue from the study at any time, for any reason, without prejudice to further treatment.

The investigator may withdraw a subject from the study if the subject:

1. Is non-compliant with the protocol
2. Experiences a serious AE (SAE) or intolerable AE(s) that in the investigator’s opinion requires withdrawal from the study
3. Has laboratory safety assessments that reveal clinically significant hematological or biochemical changes from baseline values

DocuSign Envelope ID: 774CDD36-E051-4E09-B4BF-7A852F085DB0



4. Develops, during the course of the study, symptoms or conditions listed in the exclusion criteria

5. Requires a medication prohibited by the protocol (Section 4.2), or

6. Requests an early discontinuation for any reason.

The investigator can also withdraw a subject upon the request of the sponsor, or if the sponsor terminates the study. Upon occurrence of an SAE or intolerable AE, the investigator will confer with the sponsor. If a subject is discontinued because of an AE, the event will be followed until it is resolved, stable, or judged by the investigator to be not clinically significant.

### 4.4.2 Handling of Withdrawals

Subjects are free to withdraw from the study at any time upon request. Subject participation in the study may be stopped at any time at the discretion of the investigator or at the request of the sponsor.

When a subject withdraws from the study, the reason(s) for withdrawal shall be recorded by the investigator on the relevant page of the electronic case report form (eCRF). Whenever possible, any subject who withdraws from the study prematurely will undergo all EOS assessments. Any subject who fails to return for final assessments will be contacted by the site in an attempt to have them comply with the protocol. The status of subjects who fail to complete final assessments will be documented in the eCRF.

If the subject withdraws from the study and also withdraws consent for disclosure of future information, no further evaluations should be performed, and no additional data should be collected. The sponsor may retain and continue to use any data collected before such withdrawal of consent.

**Lost to follow up**

If a subject does not return for a scheduled visit, every effort should be made to contact the subject. The investigator or site staff should attempt to contact the subject twice. After 2 attempts, CRU staff may send a registered letter. If no response is received from the subject, the subject will be considered lost to follow up. All attempts to contact the subject and information received during contact attempts must be documented in the subject's medical record. In any circumstance, every effort should be made to document subject outcome, if possible. The investigator should inquire about the reason for withdrawal, request

DocuSign Envelope ID: 774CDD36-E051-4E09-B4BF-7A852F085DB0



that the subject return for a final visit, if applicable, and follow up with the subject regarding any unresolved AEs.

All reasonable efforts must be made to locate subjects to determine and report their ongoing status. This includes follow up with persons authorized by the subject as noted above. All attempts should be documented in the subject's medical records. If it is determined that the subject has died, the site will use locally permissible methods to obtain the date and cause of death. If the investigator's use of a third-party representative to assist in the follow up portion of the study has been included in the subject's informed consent, then the investigator may use a sponsor-retained third-party representative to assist site staff with obtaining the subject's contact information or other public vital status data necessary to complete the follow up portion of the study. The site staff and representative will consult publicly available sources, such as public health registries and databases, in order to obtain updated contact information. If, after all attempts, the subject remains lost to follow up, then the last-known-alive date as determined by the investigator should be reported and documented in the subject's medical records.

### 4.4.3 Replacements

At the discretion of the investigator, and after consultation with the medical monitor, any subject who withdraws before completing the study may be replaced to retain the target of 80 evaluable subjects. Any replacement subject will be assigned to receive the same treatment as the subject he or she is replacing.

# 5. STUDY TREATMENTS

Study treatment is defined as any investigational treatment(s), marketed product(s), or placebo intended to be administered to a study subject according to the study randomization or treatment allocation.

- INS018_055 capsules at doses of 10, 30, 60, 90, and 120 mg in Part A
- Matched placebo capsules
- The top dose explored in Part B will not exceed the maximum dose explored in Part A. Subsequent dose escalations in the MAD phase will be done based on a review of the safety and tolerability data of the preceding MAD dose level.
- INS018_055 capsules at the highest dose explored in MAD (Cohort 8) in Part C

DocuSign Envelope ID: 774CDD36-E051-4E09-B4BF-7A852F085DB0



- Caffeine tablets 200 mg in Part C

Study treatment includes both investigational [medicinal] product (IP/IMP) and non-IP / non-IMP) and can consist of the following:

- All products, active or placebo, being tested or used as a comparator in a clinical study
- Study-required premedication

An IP, also known as IMP in some regions, is defined a pharmaceutical form of an active substance or placebo being tested or used as a reference in a clinical study, including products already with a marketing authorization but used or assembled (formulated or packaged) differently than the authorized form, or used for an unauthorized indication, or when used to gain further information about the authorized form.

INS018_055 will be provided to subjects as capsules or matching placebo and caffeine will be provided to subjects as tablets for oral administration. Instructions for storage and administration will be provided to the investigative site separate of the protocol.

Other medications used as support or escape medication for preventative, diagnostic, or therapeutic reasons, as components of the standard of care for a given diagnosis, may be considered as non-IPs.

## 5.1 TREATMENTS ADMINISTERED

The selection and timing of dosing is dependent on the randomization and includes the following IPs:

**Table 5.1-1 Selection and Timing of Dose**

| Study Treatment | Unit Dose Strengths/Dosage levels | Dosage Formulation Frequency of Administration | Route of Administration |
|---|---|---|---|
| **Part A[0]** | | | |
| INS018_055 | 10, 30, 60, 90, and 120 mg | Single dose on Day 1 | Oral |
| INS018_055 Placebo Match | N/A | Single dose on Day 1 | Oral |
| **Part B** | | | |
| INS018_055 | Will be determined based on safety, tolerability, and PK data from SAD and MAD cohorts | QD/BID from Day 1 through Day 7 | Oral |
| INS018_055 Placebo Match | N/A | QD/BID from Day 1 through Day 7 | Oral |

DocuSign Envelope ID: 774CDD36-E051-4E09-B4BF-7A852F085DB0



| Study Treatment | Unit Dose Strengths/Dosage levels | Dosage Formulation Frequency of Administration | Route of Administration |
|---|---|---|---|
| **Part C** | | | |
| INS018_055 | Highest dose tested in Part B | QD/BID from Day 5 through Day 18 | Oral |
| Caffeine | 200 mg | Single dose on Day 1 and Day 18 | Oral |

Abbreviations: BID, twice daily; MAD, multiple ascending dose; N/A, not applicable; PK, pharmacokinetic; QD, once daily; SAD, single ascending dose.

[a] During the food effect assessment (Period 2) of Cohort 4, subjects will be re-administered the same dose of INS018_055 or matching placebo (same study treatment that they received on Day 1 of Period 1).

During Part A, each subject will receive the assigned dose of study treatment in the morning on Day 1, as per the randomization schedule. During Part B, each subject will receive the assigned dose of study treatment in the morning from Day 1 through Day 7, as per the randomization schedule. For BID dosing, the evening dose will be administered approximately 12 hours after the morning dose. During Part C, all subjects will receive study treatment in 2 periods. In Period 1, subjects will receive single oral dose of caffeine 200 mg on Day 1. In Period 2, subjects will receive multiple oral doses of INS018_055 at the highest dose tested in Part B (MAD) for 14 days (from Days 5 through 18) and a single oral dose of caffeine 200 mg on Day 18. Dosing of caffeine will occur 2 hours prior to INS018_055 dosing on Day 18. There will be a washout of at least 3 days between dosing in each period.

The treatments to be administered in each part are outlined in Section 3. At the time of dosing, 240 mL of room temperature water will be administered to the subject along with the study drug. The time of dose administration will be called “0” hour.

Restrictions related to food and fluid intake are described in Section 4.3.1.

### 5.1.1 Dose Escalation

#### 5.1.1.1 Part A (SAD)

The sponsor will form an internal SRC for a blinded review of safety data for each subject and the entire cohort and will make a recommendation on whether to proceed to the next dose level. A sentinel cohort of 2 subjects will be used to mitigate the risk of unexpected AEs not predicted by preclinical pharmacology and toxicology studies for all cohorts. The sentinel subjects will be dosed in a blinded fashion (1 active, 1 placebo) and monitored for at least 1 day before the remaining 6 subjects in that cohort are dosed. Initiation of dosing of the remaining 6 subjects will depend on an initial safety review by the investigator indicating that administration of the study treatment was safe and well tolerated in the sentinel subjects.

DocuSign Envelope ID: 774CDD36-E051-4E09-B4BF-7A852F085DB0



Dose escalation will occur only after the real time PK, safety, and tolerability data (including reported AEs, physical examinations, vital signs, 12-lead ECGs, and clinical laboratory results up to 48 hours after dosing) of the preceding dose cohort for at least 6 subjects are assessed and the study treatment is deemed safe and well tolerated by the SRC.

As new safety and/or PK data become available, the anticipated dose escalation scheme listed in Table 5.1-1 may change following a review of the data by the SRC. Preliminary PK data from subjects in this study will help guide dose escalation to higher doses. Dose escalation to Cohorts 4 and 5 will only occur after the PK data from at least the first 2 cohorts have been assessed and deemed sufficient to model exposures for Cohorts 4 and 5.

The SRC member details will be provided in the safety committee charter. The recommendations of this committee will also be discussed with the investigator. A recommendation to stop dosing will be binding.

#### 5.1.1.2 Part B (MAD)

Subjects in Part B will begin dosing, either QD or BID, after PK, safety, and tolerability data (including reported AEs, physical examination, clinical laboratory results, 12-lead ECGs, and vital signs) from at least the first 3 cohorts (Cohorts 1 to 3) in Part A are assessed and the study treatment is deemed safe and well tolerated by the SRC. Additionally, sufficient PK data from at least the first 3 cohorts of Part A must be obtained to model exposures in Part B. The remaining SAD cohorts will continue to be dosed per protocol in parallel with the dosing of the initial MAD cohort(s). The top dose explored in Part B will not exceed the maximum dose explored in Part A. Subsequent dose escalations in Part B will not occur until the safety and tolerability data up to and including Day 11 of the preceding MAD dose cohort for at least 6 subjects are assessed and the study treatment is deemed safe by the SRC.

As new safety or PK data become available, the dose escalation scheme may change. Subjects may not receive a subsequent higher dose and may instead be administered a lower dose or may repeat the same daily dose with a different administration schema, eg, BID instead of QD dosing to achieve lower $C_{max}$ values, particularly if the safety findings are believed to be linked to the $C_{max}$ values. Before implementing any change, the institutional review board (IRB) will be notified and provided with the rationale.

The highest dose in the MAD (either QD or BID) will be dosed in a staggered way if accrued PK data from previous cohorts predict potential exposures above the NOAEL, with 2 subjects starting dosing before the remaining 6 subjects; initiation of dosing of the

DocuSign Envelope ID: 774CDD36-E051-4E09-B4BF-7A852F085DB0



remaining 6 subjects will depend on an initial safety review by the investigator indicating that administration of the study treatment was safe and well tolerated in the sentinel subjects.

### 5.1.2 Dose Escalation Stopping Criteria

Dosing within a dose cohort will be stopped and dose escalation will be suspended until safety information can be reviewed if any one of the following criteria is met and confirmed by repeat test as appropriate (a repeat draw must be performed within 24 hours):

- SAEs related to study treatment that in the opinion of the SRC indicate that the limits of safety and tolerability have been met and that preclude further safe dosing of subjects
- Two or more subjects within the same dose cohort have the same (potentially) study treatment-related AE and at severe intensity, while exposed to study treatment
- Two or more subjects within the same dose cohort have, while exposed to study treatment, a clinically relevant non-major bleeding event defined as acute clinically overt bleeding that does not satisfy additional criteria required for the bleeding event to be defined as a major bleeding event and meets at least one of the following criteria: hospital admission for bleeding or physician-guided medical or surgical treatment for bleeding
- One subject, while exposed to study treatment, has major bleeding defined as intracranial hemorrhage, a hemoglobin drop of ≥5 g/dL, a reduction of hematocrit by ≥30%, or a requirement for blood transfusion
- One subject has serum creatinine >2 × ULN, confirmed by repeat
- Two or more subjects within a dose cohort have QTcF >500 msec, confirmed by repeat ECG
- Two subjects have AST and/or ALT >5 × ULN, confirmed by repeat
- One subject has AST and/or ALT >3 × ULN AND total bilirubin >2 × ULN, confirmed by repeat
- Two or more subjects within the same dose cohort have a total bilirubin >3 × ULN, confirmed by repeat

DocuSign Envelope ID: 774CDD36-E051-4E09-B4BF-7A852F085DB0



- Two subjects within a dose cohort have ALT or AST >3 × ULN (confirmed by repeat) associated with signs or symptoms the investigator considers to be consistent with hepatic injury

InSilico Medicine Hong Kong Limited reserves the right to terminate access to the supplied study treatment if the development of INS018_055 is terminated for other reasons including, but not limited to, not meeting other key study objectives.

The sponsor and investigator may decide to halt dosing within a cohort or dose escalation for reasons not defined above, including but not limited to, observing a single SAE in individual subjects and/or observing trends in a given dose cohort and/or across dose cohorts.

If any of the above criteria are met within a dose level, the progression to a higher dose level will be put on hold and all relevant safety data available across the study will be evaluated to estimate the risk of proceeding to the higher dose level. The review may include unblinding of the subject(s) who experienced AEs listed above. The unblinded data set may include subjects from a dose cohort, or if appropriate, all treated subjects to date. Unblinding recommendation must be approved by the sponsor's medical monitor. Upon conclusion of this in-depth safety review, one of the following recommendations will be made:

- To continue with the study as planned
- To continue with the study by repeating the current dose in more subjects
- To continue with the study at a dose between the current dose and the next planned dose or at a dose between the current dose and the previous lower dose
- To continue with a modified dose regimen deemed necessary by safety, tolerability, or PK analysis
- To terminate the study

The maximum acceptable dose will be the dose level below the dose level at which the stopping criteria are met unless it is determined that a lower dose should be designated as the maximum acceptable dose.

### 5.1.3 Part C (DDI)

Subjects in Part C will begin dosing with INS018_055 in Period 2, after safety and tolerability data (including reported AEs, physical examination, clinical laboratory results,

DocuSign Envelope ID: 774CDD36-E051-4E09-B4BF-7A852F085DB0



12-lead ECGs, and vital signs) from the 3 cohorts (Cohorts 6 to 8) in Part B are assessed and the study treatment is deemed safe and well tolerated by the SRC. Additionally, sufficient PK data from the 3 cohorts of Part B must be obtained to model exposures in Part C.

## 5.2 INVESTIGATIONAL PRODUCTS

The study drugs that will be used are as follows:

| Product | Supplied Formulation |
|---|---|
| INS018_055 | 5, 10, 30 mg capsule |
| Matching placebo | 0 mg capsule |
| Caffeine | 100 mg |

INS018_055 acetate salt is the active ingredient added directly into empty hypromellose capsule and contains no inactive excipients. The placebo for INS018_055 will be identical in appearance but will not include the active compound.

Caffeine tablet contains the following inactive excipients: glucose monohydrate, magnesium stearate, and sodium starch glycollate.

Further information on the study drug can be found in the IB.

### 5.2.1 Study Drug Packaging and Storage

InSilico Medicine Hong Kong Limited will provide the investigator and clinical unit with adequate quantities of INS018_055 (5, 10, and 30 mg capsules) and matching placebo. The clinical unit pharmacy will prepare the study treatments for each subject according to the schedule of events (SOE; Section 3.1). The capsules will be provided in unit dose containers and labeled in accordance with InSilico Medicine Hong Kong Limited regulations and the clinical unit’s labeling requirements.

The 5 and 10 mg capsules are of Size 4 and the 30 mg capsule is Size 3. The 5 mg capsule has a standard yellow cap and Swedish orange opaque body, while the 10 and 30 mg capsules have Swedish orange opaque caps and bodies.

INS018_055 capsules, 5 mg, 10 mg, and 30 mg, are packaged in 45 mL high-density polyethylene (HDPE) bottles sealed with polypropylene (PP) continuous-thread child-resistant caps (CRC) with an induction sealed and aluminum-faced liner. Each bottle is filled with 30 capsules. The study drug should be stored at controlled room temperature (15°C to 25°C).

DocuSign Envelope ID: 774CDD36-E051-4E09-B4BF-7A852F085DB0



Caffeine 100 mg tablets will be supplied by the study site.

All study drugs must be stored according to the labeled instructions in a secure cabinet or room with access restricted to necessary clinic personnel. The site will be required to keep a temperature log to establish a record of compliance with storage conditions.

### 5.2.2 Study Drug Accountability

The investigator will maintain accurate records of receipt of all study drugs, including dates of receipt. Accurate records will be kept regarding when and how much study drug is dispensed and used by each subject in the study. Reasons for departure from the expected dispensing regimen must also be recorded. At the completion of the study, and to satisfy regulatory requirements regarding drug accountability, all study drugs will be reconciled and retained or destroyed according to applicable regulations.

## 5.3 METHOD OF ASSIGNING SUBJECTS TO TREATMENT GROUPS

**Part A (SAD) and Part B (MAD)**

PPD will generate the randomization schedule for Parts A and B. Eligible subjects will be enrolled into the current dose cohort at a ratio of 3:1. Each cohort will be independently randomly assigned by a qualified person who is not directly involved in study conduct, data management, or data analysis.

**Part C (DDI)**

This is a non-randomized study part. Subjects who meet all inclusion and none of the exclusion criteria will receive all study treatments according to the SOE (Table 3.1-3).

## 5.4 BLINDING

### 5.4.1 Blinding Procedures

**Part A (SAD) and Part B (MAD)**

These study parts will employ a double-blind study design. The INS018_055 and matching placebo capsules will be identical in appearance. The unblinded pharmacist will be responsible for dispensing the study drug in a manner consistent with maintaining the blind.

DocuSign Envelope ID: 774CDD36-E051-4E09-B4BF-7A852F085DB0



**Part C (DDI)**

Part C is open-label; blinding procedures are not applicable.

### 5.4.2 Breaking the Blind

The medical monitor will be responsible for maintaining the blind throughout the study. If a subject becomes seriously ill or pregnant during the study, the blind will be broken only if knowledge of the administered study drug will affect that subject's available treatment options. In the event of a medical emergency requiring identification of the study drug administered to an individual subject, the investigator will make every attempt to contact the medical monitor to explain the need for opening the code within 24 hours of opening the code. The investigator will be responsible for documenting the time, date, reason for the code break, and the names of the personnel involved.

## 5.5 TREATMENT COMPLIANCE

All doses of the study treatment will be administered in the clinical unit under direct observation of clinic personnel and recorded in the eCRF. Clinic personnel will confirm that the subject has received the entire dose of study treatment.

The date and time of study drug dosing will be recorded on the appropriate page of the eCRF. If a subject is not administered study treatment, the reason for the missed dose will be recorded.

### 5.5.1 Prior and Concomitant Medications

Restrictions for prior and concomitant medications and therapies are provided in Sections 4.1 and 4.2. Prior and concomitant medications and therapies will be coded using the latest version of the World Health Organization Drug Dictionary.

#### 5.5.1.1 Prior Medications

Information regarding prior medications taken by the subject within the 30 days before signing the informed consent form (ICF) will be recorded in the subject's electronic case report form.

#### 5.5.1.2 Concomitant Medications

Any concomitant medication deemed necessary for the welfare of the subject for the treatment of an AE during the study may be given at the discretion of the investigator. If a concomitant medication is taken, except for those specified in the protocol, a joint decision

will be made by the investigator and the sponsor to continue or discontinue the subject based on the time the medication was administered, its pharmacology and PK, and whether the use of the medication will compromise the safety of the subject or the interpretation of the data. The investigator is responsible for ensuring that details regarding the medication are adequately recorded in the eCRF.

# 6. STUDY PROCEDURES

Before performing any study procedures, all potential subjects will sign an ICF as outlined in Section 9.2.2.3. Subjects will undergo study procedures at the time points specified in the SOE (Section 3.1).

The total amount of blood collected from each subject over the duration of the study, including any extra assessments that may be required, will not exceed 500 mL.

## 6.1 PHARMACOKINETIC ASSESSMENTS AND ENDPOINTS

**Part A (SAD)**

Blood samples for PK analysis of INS018_055 and metabolites (INS018_063 and INS018_095) will be collected on Day 1 at the following time points: before dosing (0 hours) (within 45 minutes prior to dosing) and at 0.25 (± 5 min), 0.5 (± 5 min), 1 (± 5 min), 2 (± 5 min), 4 (± 5 min), 6 (± 10 min), 8 (± 10 min), 10 (± 10 min), and 12 hours (± 10 min); on Day 2 at 24 (± 30 min) and 36 hours (± 30 min); on Day 3 at 48 hours (± 60 min); and on Day 4 at 72 hours (± 60 min) after administration of INS018_055.

**Part B (MAD)**

For QD dosing, blood samples for PK analysis of INS018_055 and metabolites (INS018_063 and INS018_095) will be collected at the following time points:

- Day 1: before dosing (0 hour) (within 45 minutes prior to dosing) and at 0.25 (± 5 min), 0.5 (± 5 min), 1 (± 5 min), 2 (± 5 min), 4 (± 5 min), 6 (± 10 min), 8 (± 10 min), 10 (± 10 min), and 12 hours (± 10 min) after dosing
- Days 2, 3, 4, 5, and 6: before dosing, following at least an 8-hour fasting period, before breakfast
- Day 7: before dosing (0 hour) (within 45 minutes prior to dosing) and at 0.25 (± 5 min), 0.5 (± 5 min), 1 (± 5 min), 2 (± 5 min), 4 (± 5 min), 6 (± 10 min), 8 (± 10 min),

DocuSign Envelope ID: 774CDD36-E051-4E09-B4BF-7A852F085DB0



10 (± 10 min), 12 (± 10 min), 24 (± 30 min), 48 (± 60 min), and 72 hours (± 60 min) after dosing

For BID dosing, blood samples for PK analysis of INS018_055 and metabolites (INS018_063 and INS018_095) will be collected at the following time points:

- Day 1: before the morning dosing (0 hour) (within 45 minutes prior to dosing) and at 0.25 (± 5 min), 0.5 (± 5 min), 1 (± 5 min), 2 (± 5 min), 4 (± 5 min), 6 (± 10 min), 8 (± 10 min), 10 (± 10 min), and 12 hours (± 10 min) after the morning dosing, which is 0 hours before the evening dosing
- Days 2, 3, 4, 5, and 6: before the morning dosing, following at least an 8-hour fasting period, before breakfast
- Day 7: before the evening dosing (0 hour) (within 45 minutes prior to dosing) and at 0.25 (± 5 min), 0.5 (± 5 min), 1 (± 5 min), 2 (± 5 min), 4 (± 5 min), 6 (± 10 min), 8 (± 10 min), 10 (± 10 min), 12 (± 10 min), 24 (± 30 min), 48 (± 60 min), and 72 hours (± 60 min) after the evening dosing.

The following plasma PK parameters will be calculated as endpoints for INS018_055 and metabolites (INS018_063 and INS018_095) using actual sampling times rather than scheduled sampling times:

- Area under the plasma concentration versus time curve (AUC) from time 0 to the last quantifiable concentration ($AUC_{0-t}$)
- AUC from time 0 extrapolated to infinity ($AUC_{0-inf}$)
- AUC from time 0 to the time of the dosing interval (τ; $AUC_{0-\tau}$)
- Accumulation ratio (AR), calculated as $AUC_{0-\tau}$ (Day 7)/$AUC_{0-\tau}$ (Day 1)
- AR calculated as $C_{max}$ (Day 7)/$C_{max}$ (Day 1)
- $C_{max}$
- Time to reach $C_{max}$ ($T_{max}$)
- Pre-dose concentrations on Days 1 through 7 ($C_{trough}$)
- Average concentration on Day 1 and Day 7 ($C_{av}$)

DocuSign Envelope ID: 774CDD36-E051-4E09-B4BF-7A852F085DB0



- Terminal elimination rate constant ($K_{el}$)
- Terminal elimination half-life ($t_{1/2}$)
- Apparent total body clearance (CL/F)
- Peak to trough ratio calculated as $C_{max}/C_{trough}$
- Apparent volume of distribution ($V_d/F$)
- Metabolite-to parent ratio based on AUC calculated as $AUC_{metabolite}/AUC_{parent}$
- Metabolite-to parent ratio based on $C_{max}$ calculated as $C_{max, metabolite}/C_{max, parent}$

Urine samples will be collected on Day -1 for either QD or BID dosing schedules for PK analysis before dosing as a single void collected within 0 to 24 hours and over the following intervals after dosing: 0 to 4, 4 to 8, 8 to 12, and 12 to 24 hours on Day 1 and 0 to 4, 4 to 8, 8 to 12, 12 to 24, and 24 to 48 hours on Day 7.

The following urine PK parameters will be calculated as endpoints:

- Renal clearance (CLr)
- Fraction of the dose excreted unchanged in urine over all time intervals (0 to t) ($Fe\%_{0-t}$)
- Total amount of drug excreted unchanged in urine over all time intervals (0 to t) ($XU_{0-t}$)

**Part C (DDI)**

Blood samples for PK analysis of caffeine in plasma will be collected on Days 1 and 18: before dosing (0 hour) (within 45 minutes prior to dosing) and at 0.25 (± 5 min), 0.5 (± 5 min), 1 (± 5 min), 2 (± 5 min), 4 (± 5 min), 6 (± 10 min), 8 (± 10 min), 10 (± 10 min), 12 (± 10 min), 24 (± 30 min), 48 (± 60 min), and 72 hours (± 60 min) following caffeine dosing.

Blood samples for PK analysis of INS018_055 in plasma will be collected before administration of INS018_055 on Days 5 through 18 for the assessment of INS018_055 steady state.

The following plasma PK parameters will be calculated as endpoints for caffeine using actual sampling times rather than scheduled sampling times:

DocuSign Envelope ID: 774CDD36-E051-4E09-B4BF-7A852F085DB0



- $AUC_{0-t}$
- $AUC_{0-inf}$
- $C_{max}$
- $T_{max}$
- $K_{el}$
- $t_{1/2}$
- CL/F
- $V_d$/F

### 6.1.1 Pharmacokinetic Sample Collection

Details for the collection, processing, storage, and shipping of PK samples will be provided to the clinical unit in a separate PK manual.

### 6.1.2 Pharmacokinetic Sample Analysis

Pharmacokinetic samples will be analyzed using a validated liquid chromatography coupled with tandem mass spectrometry assay for INS018_055, INS018_063, and INS018_095 analyte(s) and caffeine (Part C only) in human plasma and urine. Assay results and validation details will be provided in a separate bioanalytical report.

## 6.2 SAFETY ASSESSMENTS AND ENDPOINTS

Safety and tolerability will be assessed by the following endpoints: monitoring and recording of AEs, clinical laboratory test results (hematology, coagulation, serum chemistry, urinalysis, and fecal occult blood test), vital sign measurements, 12-lead ECG results, and physical examination findings.

For all safety assessments, the investigator will determine whether results are clinically significant, which is defined as any variation in a result that has medical relevance and may result in an alteration in medical care (eg, active observation, diagnostic measures, or therapeutic measures). If clinical significance is noted, the result and reason for significance will be documented and an AE reported on the AE page of the subject's eCRF. The investigator will monitor the subject until the result has reached the reference range or the

DocuSign Envelope ID: 774CDD36-E051-4E09-B4BF-7A852F085DB0



result at screening, or until the investigator determines that follow up is no longer medically necessary.

### 6.2.1 Adverse Events

Adverse events will be assessed from the time of INS018_055 dosing (Parts A and B) and caffeine dosing (Part C, DDI) until EOS and should be followed until they are resolved, stable, or judged by the investigator to be not clinically significant.

The investigator is responsible for ensuring that all AEs and SAEs are recorded in the eCRF and reported to the sponsor, regardless of their relationship to study drug or clinical significance. If there is any doubt as to whether a clinical observation is an AE, the event should be reported.

#### 6.2.1.1 Adverse Event Definitions

An AE is defined as any untoward medical occurrence associated with the use of a drug in humans, whether or not considered drug related. Subjects will be instructed to contact the investigator at any time after randomization if any symptoms develop.

A treatment-emergent AE is defined as any event not present before exposure to study drug or any event already present that worsens in intensity or frequency after exposure.

A suspected adverse reaction is any AE for which there is a reasonable possibility that the study drug caused the AE. For the purposes of investigational new drug safety reporting, “reasonable possibility” means that there is evidence to suggest a causal relationship between the study drug and the AE. A suspected adverse reaction implies a lesser degree of certainty about causality than an adverse reaction.

An adverse reaction is any AE caused by a study drug. Adverse reactions belong to a subset of all suspected adverse reactions and indicate that there are reasons to conclude that the study drug caused the event.

An AE or suspected adverse reaction is considered “unexpected” if it is not listed in the investigator brochure or if it occurs with specificity or severity that has not been previously observed with the study drug being tested; or, if an investigator brochure is not required or available, the AE or suspected adverse reaction is not consistent with the risk information described in the general investigational plan or elsewhere in the current application. For example, under this definition, hepatic necrosis would be unexpected (by virtue of greater severity) if the investigator brochure referred only to elevated hepatic enzymes or hepatitis.

DocuSign Envelope ID: 774CDD36-E051-4E09-B4BF-7A852F085DB0



Similarly, cerebral thromboembolism and cerebral vasculitis would be unexpected (by virtue of greater specificity) if the investigator brochure listed only cerebral vascular accidents. "Unexpected," as used in this definition, also refers to AEs or suspected adverse reactions that are mentioned in the investigator brochure as occurring with a class of drugs or as anticipated from the pharmacological properties of the drug, but are not specifically mentioned as occurring with the particular drug under investigation.

An AE or suspected adverse reaction is considered an SAE/suspected unexpected serious adverse reaction if, in the view of either the investigator or sponsor, it results in any of the following outcomes:

- Death
- Life-threatening AE
- Inpatient hospitalization or prolongation of existing hospitalization
- Persistent or significant incapacity or substantial disruption of the ability to conduct normal life functions
- Congenital anomaly or birth defect

Important medical events that may not result in death, be life threatening, or require hospitalization may be considered serious when, based upon appropriate medical judgment, they may jeopardize the subject and may require medical or surgical intervention to prevent one of the outcomes listed in this definition. Examples of such medical events include allergic bronchospasm requiring intensive treatment in an emergency room or at home, blood dyscrasias or convulsions that do not result in inpatient hospitalization, or the development of drug dependency or drug abuse.

An AE or suspected adverse reaction is considered "life threatening" if, in the view of either the investigator or sponsor, its occurrence places the subject at immediate risk of death. It does not include an AE or suspected adverse reaction that might have caused death if it had been more severe.

#### 6.2.1.2 Eliciting and Documenting Adverse Events

Subjects will be asked a standard question to elicit any medically related changes in their well-being. They will also be asked if they have been hospitalized, had any accidents, used

DocuSign Envelope ID: 774CDD36-E051-4E09-B4BF-7A852F085DB0



any new medications, or changed concomitant medication regimens (both prescription and over-the-counter medications).

In addition to subject observations, AEs will be documented from any data collected on the AE page of the eCRF (eg, laboratory values, physical examination findings, and ECG changes) or other documents that are relevant to subject safety.

#### 6.2.1.3 Reporting Adverse Events

All AEs reported or observed during the study will be recorded on the AE page of the eCRF. Information to be collected includes drug treatment, type of event, time of onset, dosage, investigator-specified assessment of severity and relationship to study drug, time of resolution of the event, seriousness, any required treatment or evaluations, and outcome. Any AEs resulting from concurrent illnesses, reactions to concurrent illnesses, reactions to concurrent medications, or progression of disease states must also be reported. All AEs will be followed until they are resolved, stable, or judged by the investigator to be not clinically significant. The Medical Dictionary for Regulatory Activities (MedDRA) will be used to code all AEs.

Any medical condition that is present at the time that the subject is screened but does not deteriorate should not be reported as an AE. However, if it deteriorates at any time during the study, it should be recorded as an AE.

Any AE that is considered serious by the investigator or which meets SAE criteria (Section 6.2.1.1) must be reported to the sponsor immediately (after the investigator has confirmed the occurrence of the SAE). The investigator will assess whether there is a reasonable possibility that the study drug caused the SAE. The sponsor will be responsible for notifying the relevant regulatory authorities of any SAE as outlined in current International Council for Harmonisation (ICH) Guideline for Good Clinical Practice (GCP) and the National Regulatory Requirements. The investigator is responsible for notifying the IRB directly.

For this study, the following contact information will be used for SAE reporting:

| | |
|---|---|
| PPD Medical Monitor: | PPD Medical Monitor<br>3900 Paramount Parkway<br>Morrisville, NC 27560 USA<br>Telephone (24 hour): +1-888-483-7729<br>Fax: +1-888-529-3580 |

DocuSign Envelope ID: 774CDD36-E051-4E09-B4BF-7A852F085DB0



#### 6.2.1.4 Assessment of Severity

The severity (or intensity) of an AE refers to the extent to which it affects the subject's daily activities and will be classified as mild, moderate, or severe using the following criteria:

- Mild: These events require minimal or no treatment and do not interfere with the subject's daily activities.
- Moderate: These events result in a low level of inconvenience or require minor therapeutic measures. Moderate events may cause some interference with normal functioning.
- Severe: These events interrupt a subject's usual daily activity and may require systemic drug therapy or other treatment. Severe events are usually incapacitating.

Changes in the severity of an AE should be documented to allow the duration of the event at each level of intensity to be assessed. An AE characterized as intermittent does not require documentation of the onset and duration of each episode.

#### 6.2.1.5 Assessment of Causality

The investigator's assessment of an AE's relationship to study drug is part of the documentation process but is not a factor in determining what is or is not reported in the study.

The investigator will assess causality (ie, whether there is a reasonable possibility that the study drug caused the event) for all AEs and SAEs. The relationship will be classified as follows:

- Not related: There is not a reasonable possibility of relationship to study drug. The AE does not follow a reasonable temporal sequence from study drug administration or can be reasonably explained by the subject's clinical state or other factors (eg, disease under study, concurrent diseases, and concomitant medications).
- Related: There is a reasonable possibility of relationship to study drug. The AE follows a reasonable temporal sequence from study drug administration and cannot be reasonably explained by the subject's clinical state or other factors (eg, disease under study, concurrent diseases, or concomitant medications), represents a known reaction to the study drug or other drugs in its class, is consistent with the known pharmacological

properties of the study drug, and/or resolves with discontinuation of the study drug (and/or recurs with re-challenge, if applicable).

#### 6.2.1.6 Follow-Up of Adverse Events

All AEs must be reported in detail on the appropriate page of the eCRF and followed until they are resolved, stable, or judged by the investigator to be not clinically significant.

### 6.2.2 Clinical Laboratory Testing

Clinical laboratory testing will occur at screening, check-in, and at the day of discharge and at other time points specified in Section 3.1. Clinical laboratory tests will be performed by the clinical sites' local laboratories. Blood and urine samples will be collected under fasting conditions and will be prepared using standard procedures.

Repeat clinical laboratory tests may be performed at the discretion of the investigator, if necessary, to evaluate inclusion and exclusion criteria or clinical laboratory abnormalities. The clinical laboratory that will perform the tests will provide the reference ranges for all clinical laboratory parameters. Abnormal clinical laboratory values will be flagged as either high or low (or normal or abnormal) based on the reference ranges for each laboratory parameter.

The following clinical laboratory assessments will be performed:

| | |
|---|---|
| Hematology | Hematocrit, hemoglobin, mean corpuscular hemoglobin, mean corpuscular hemoglobin concentration, leukocytes (basophils, eosinophils, lymphocytes, monocytes, neutrophils), mean corpuscular volume, platelet count, red blood cell count, and red cell distribution width |
| Serum Chemistry | ALT, albumin, alkaline phosphatase, AST, bilirubin (total), urea, calcium, bicarbonate, chloride, cholesterol (total, high-density lipoprotein, and calculated low-density lipoprotein), creatinine, gamma-glutamyl transferase, creatine kinase, glucose, lactate dehydrogenase, phosphorus, potassium, sodium, total protein, lipids (total cholesterol, triglycerides, low-density lipoprotein cholesterol, high-density lipoprotein cholesterol), uric acid, and Cystatin C |
| Urinalysis | Appearance, bilirubin, color, glucose, ketones, leukocytes, reflex microscopy (performed if dipstick is positive for protein or the blood value is 1+ or greater; and includes bacteria, casts, crystals, epithelial cells, red blood cells, and white blood cells), nitrites, occult blood, pH, protein, specific gravity, and urobilinogen |
| Fecal occult blood test | Presence of occult blood in stool samples |

DocuSign Envelope ID: 774CDD36-E051-4E09-B4BF-7A852F085DB0



| | |
|---|---|
| Serology | Hepatitis B surface antigen, hepatitis C virus antibody, and HIV antibody types 1 and 2 (screening only) |
| Other analyses | All subjects: Urine drug screen (amphetamines, barbiturates, benzodiazepines, cannabinoids, cocaine metabolites, cotinine, methamphetamines, methylenedioxymethamphetamine, and opiates [including heroin, codeine, and oxycodone]), blood alcohol screen using either breath alcohol or urine test, coagulation, COVID-19 screening (the clinic will follow their standard procedures and/or local guidelines with respect to SARS-CoV-2 testing, if deemed necessary by the investigator)<br>Female subjects: Follicle-stimulating hormone, serum pregnancy test (human chorionic gonadotropin)<br>Potential Hy's law cases: AST (repeat), ALT (repeat), total bilirubin (repeat), albumin (repeat), alkaline phosphatase (repeat), direct bilirubin, creatine kinase, gamma-glutamyl transferase, activated partial thromboplastin clotting time, prothrombin time, and international normalized ratio, and total bile acids |

### 6.2.3 Vital Sign Measurements

Vital signs will include systolic and diastolic BP, pulse rate, respiratory rate, and body temperature.

**Part A (SAD)**: Vital signs will be measured at screening; check-in (Day -1); Day 1 (within 45 min before dosing) and at 1 (± 30 min), 2 (± 30 min), 4 (± 30 min), 8 (± 30 min), and 12 hours (± 30 min) after dose; in the morning on Days 2 through 4; and at the EOS after the subject has been in the seated position for at least 5 minutes.

**Part B (MAD)**: Vital signs will be recorded at screening, check-in (Day -1), Days 1 to 11, and Day 25 (EOS). On Days 1 and 7: before dose (0 hour) (within 45 minutes prior to dosing) and at 1 (± 30 min), 2 (± 30 min), 4 (± 30 min), 8 (± 30 min), 12 (± 30 min), and 24 (± 60 min) hours after dose. Vital sign measurements will be obtained prior to the morning dose on Days 2 to 6, mornings of Days 9 to 11, and the Day 25 (EOS) after the subject has been in the seated position for at least 5 minutes.

**Part C (DDI)**: Vital signs will be recorded at screening, check-in (Day -1), Days 1 to 21, and Day 24 (EOS). On Days 1 and 18: before dose (0 hour) (within 45 minutes prior to dosing) and at 1 (± 30 min), 2 (± 30 min), 4 (± 30 min), 8 (± 30 min), 12 (± 30 min), and 24 (± 60 min) hours after caffeine dose. Vital sign measurements will be obtained prior to the morning dose of INS018_055 on Days 5 to 17. Vital sign measurements taken on Days 2 to 4, Days 19 to 21, and Day 24 (EOS) will be performed in the morning after the subject has been in the seated position for at least 5 minutes.

### 6.2.4 Electrocardiograms

**Part A (SAD):** Standard 12-lead ECG recordings will be made in triplicate at screening and check-in (Day -1); single ECGs will be collected at all other specified visits. On Day 1, ECG will be recorded before dose (15 min prior to dosing) and at 1 (± 15 min), 2 (± 15 min), 4 (± 15 min), and 8 hours (± 15 min) after dose. Additional ECG recordings will be taken on Days 2 and 3, prior to discharge on Day 4, and at the EOS after the subject has been in the supine position for at least 5 minutes.

**Part B (MAD):** Standard 12-lead ECGs will be recorded in triplicate at screening and check-in (Day -1); single ECGs will be collected at all other specified visits. On Days 1 and 7, ECG will be recorded before dose and at 1 (± 15 min), 2 (± 15 min), 4 (± 15 min), 8 (± 15 min), and 12 (± 15 min) hours after dose. In addition, ECGs will be obtained prior to the morning dose on Days 2, 4, and 6; on the mornings of Days 8 and 9 (12 and 36 hours after the evening dose on Day 7); and before discharge on Day 11 (Section 3.1). All ECG recordings will be taken after the subject has been in the supine position for at least 5 minutes.

**Part C (DDI)**: Standard 12-lead ECGs will be recorded in triplicate at screening and check-in (Day -1); single ECGs will be collected at all other specified visits. On Days 1 and 18: before dose and at 1 (± 15 min), 2 (± 15 min), 4 (± 15 min), 8 (± 15 min), and 12 (± 15 min) hours after caffeine dose. In addition, ECGs will be obtained prior to the morning dose of INS018_055 on Days 5 and 11; and before discharge on Day 21 (Section 3.1). All ECG recordings will be taken after the subject has been in the supine position for at least 5 minutes.

Electrocardiogram assessments will include comments on whether the tracings are normal or abnormal, rhythm, presence of arrhythmia or conduction defects, morphology, any evidence of myocardial infarction, or ST-segment, T-wave, and U-wave abnormalities. In addition, measurements of the following intervals will be measured and reported: RR interval, PR interval, QRS width, QT interval, and QT interval corrected for heart rate using Fridericia’s formula. Clinically relevant abnormalities occurring during the study should be recorded in the AE section of the eCRF.

### 6.2.5 Physical Examinations

A full physical examination will be performed at screening. A brief physical examination will be performed at check-in (Day -1), all other listed timepoints, and EOS.

DocuSign Envelope ID: 774CDD36-E051-4E09-B4BF-7A852F085DB0



A full physical examination will include, at minimum, assessment of skin, head, ears, eyes, nose, throat, neck, thyroid, lungs, heart, cardiovascular, abdomen, lymph nodes, and musculoskeletal system/extremities. A brief physical examination will include, at minimum, assessment of skin, lungs, cardiovascular system, and abdomen (liver and spleen). Interim physical examinations may be performed at the discretion of the investigator, if necessary, to evaluate AEs or clinical laboratory abnormalities.

**Part A (SAD)**: Physical examinations will be performed approximately 24 (Day 2) and 48 hours (Day 3) after dosing.

**Part B (MAD)**: Physical examinations will be performed on Days 2, 5, and 7 after dosing, and before discharge on Day 11.

**Part C (DDI)**: Physical examinations will be performed on Days 4, 11, 18, and 21.

## 6.3 BIOMARKERS

Blood will be drawn at the times indicated in Table 3.1-1 (Part A [SAD]) and Table 3.1-2 (Part B [MAD], QD/BID dosing) for the measurement of biomarkers. The subpopulation of T-cells (CD4+ and CD8+), IL-6, TGF-β, MMP-2, MMP-9, and MMP-7 will be assessed in plasma and phospho-NF-kB p65 (Ser536), phospho-Smad2 (Ser465/467), total NF-kB p65, and total Smad2 will be assessed in peripheral blood mononuclear cells.

### 6.3.1 CD4+ and CD8+ Subpopulation of T-Cells

In nonclinical studies, INS018_055 was found to result in complex changes of lymphocyte populations, both in the circulating fraction and in lymphatic tissues. It is assumed that these changes reflect pharmacological effects of INS018_055, so that a change in circulating T-lymphocyte subpopulations could be a helpful biomarker for selection of the therapeutic dose range in late-stage clinical trials.

Characterization of T-cell subpopulations is included as a biomarker, because complex changes in lymphocytes have been observed in nonclinical studies and are assumed to represent major pharmacological effects. The latter assumption is based on the expression of the target in specific T-cells. Eosinophils, T-cells, and basophils are the group enriched with TNIK expression specifically in blood cells. A decrease in the level of lymphocytes may be due to inhibitory effects of INS018_055 on T-cell differentiation to short-lived subtypes. As described, several genome-wide association study studies revealed strong genetic associations between TNIK and lymphocyte count (PMID:27863252; PMID:32888494;

DocuSign Envelope ID: 774CDD36-E051-4E09-B4BF-7A852F085DB0



PMID:32888493). CD27/TNIK/Wnt signaling favors memory T-cell differentiation and cell division, while TNIK-deficient T-cells preferentially differentiated into short-lived effector cells, while memory T-cell formation was impaired (PMID: 32242021). These observations strongly suggest the association between TNIK and lymphocytes. Therefore, INS018_055 may induce T-cell differentiation via TNIK-signaling, consequently leading to decreased levels of T-cells upon treatment.

Further details of blood collection and processing will be provided to the site in the procedure manual. Data for exploratory biomarkers may be reported separately from the clinical study report.

### 6.3.2 Phospho-NF-kB p65 (Ser536), Phospho-Smad2 (Ser465/467), Total NF-kB p65, and Total Smad2

The PD properties of INS018_055 will be assessed through the analysis of phospho-NF-kB p65 (Ser536) and phospho-Smad2 (Ser465/467) in peripheral blood mononuclear cells, both PD markers of TNIK inhibition. The effect of INS018_055 on phospho-p65 and phospho-pSmad2 have been confirmed in the pulmonary epithelial cell-based study (Study No. IMHK-20210531), where INS018_055 reduced the phosphorylation of the both proteins on the indicated sites in dose-dependent manners. The inhibitory effects induced by INS018_055 are consistent with that caused by the knockdown of TNIK (Study No. IMHK-20201123; ShkodaLongoLongo et al 2012).

Total NF-kB p65 and total Smad2 will be assessed to conclude whether the change of phospho-NF-kB p65 (Ser536) and phospho-Smad2 (Ser465/467), if there is any, is the result of changed phosphorylation level or the result of changed total protein level.

### 6.3.3 IL-6 and TGF-β

It is established that there is a crosstalk between IL-6 and Wnt signaling, where TNIK mediates the related biological process (Longo et al 2002; Lee Y et al 2017). The effect of INS018_055 on IL-6 expression has been confirmed in the BioMAP Fibrosis panel of 3 human primary cell-based systems cell-based studies (Study No. US034-0011915-O). The current PD analysis will confirm the effect on these markers in the plasma samples from healthy subjects. Additionally, the effects of INS018_055 on the protein levels of TGF-β and IL-6 are suggested to be assessed pre- and posttreatment. Such data were already generated in the mouse bleomycin model (Study No. ISM-ME-10-v): TGF-β and IL-6 increased in the BALF after BLM inhalation and were reduced after INS018_055 treatment. These data

DocuSign Envelope ID: 774CDD36-E051-4E09-B4BF-7A852F085DB0



correlated with a positive effect of INS018_055 on the fibrotic readouts (modified Ashcroft score, collagen deposit in lung).

### 6.3.4 MMP-2, MMP-9, and MMP-7

Several protein biomarkers, MMP-2, MMP-9, and MMP-7, also encoded by the target genes of Wnt signaling have been shown to be increased in IPF (eg, matrix metalloproteinase (MMP)-2, MMP-9 (Wu et al 2007; Todd et al 2020), MMP-7 (Crawford et al 1999; Rosas et al 2008; Jenkins et al 2015). Higher levels of these MMPs have been identified to be associated with disease progression and worse survival. These results will help to elaborate the mechanism of the effect of INS018_055 on IPF pathophysiology and will guide endpoint selection in future clinical studies.

## 6.4 METABOLITE IDENTIFICATION AND PROFILING

**Part B (MAD), Cohort 8 only:**

Plasma samples for metabolite identification and profiling of INS018_055 will be collected on Day 7 at the following time points: before dosing (0 hours) (within 45 minutes prior to dosing) and at 0.25 (± 5 min), 0.5 (± 5 min), 1 (± 5 min), 2 (± 5 min), 4 (± 5 min), 6 (± 10 min), 8 (± 10 min), 10 (± 10 min), 12 (± 10 min), and 24 hours (± 30 min).

Urine samples for metabolite identification and profiling of INS018_055 will be collected over the following intervals after dosing 0 to 4, 4 to 8, 8 to 12, 12 to 24 hours on Day 7.

Details for the collection, processing, storage, and shipping of plasma and urine samples will be provided to the clinical unit in a separate manual. Data for exploratory metabolite identification and profiling may be reported separately from the clinical study report.

# 7. STATISTICAL ANALYSIS PLANS

## 7.1 SAMPLE SIZE CALCULATIONS

The total sample size of 80 evaluable subjects (40 subjects in Part A, 24 subjects in Part B, and 16 subjects in Part C of the study) is considered sufficient for the objectives of the study.

**Part A (SAD) and Part B (MAD)**

The number of subjects is based on clinical and practical considerations and not on a formal statistical power calculation.

DocuSign Envelope ID: 774CDD36-E051-4E09-B4BF-7A852F085DB0



**Part C (DDI)**

A sample size of 16 subjects is considered sufficient to detect any possible interaction between INS018_055 and caffeine.

Based on an intra-subject CV of 21.4% (Balogh et al 1992) for $AUC_{0\text{-}inf}$, the standard deviation of paired differences in AUC on log scale would be expected to be 0.3. A sample size of 16 evaluable subjects in Part C would give a 90% CI with good precision (half width of 0.131 on log scale leading to 1.3-fold between the lower and upper 90% CI limits). Furthermore, should there be no drug-drug interaction, then there would be 76% probability (power) that the 90% CI would fall within the 0.80 to 1.25 limits.

## 7.2 ANALYSIS SETS

The analysis populations are as follows:

- The PK population will include subjects who receive at least 1 dose of INS018_055 (Parts A and B) or at least one dose of caffeine (Part C) and have sufficient concentration data to support accurate estimation of at least 1 PK parameter. Subjects who experience vomiting within 2 times the median $T_{max}$ after study drug dosing will be excluded from the PK analysis.

- The pharmacodynamic (PD) population will include subjects who receive at least 1 dose of INS018_055 or placebo and have a valid baseline value and at least 1 non-missing post-baseline value for biomarker measurement.

- The safety population will include all subjects who receive at least 1 dose of study treatment.

## 7.3 DESCRIPTION OF SUBGROUPS TO BE ANALYZED

Not applicable.

## 7.4 STATISTICAL ANALYSIS

Details of all statistical analyses will be described in a separate statistical analysis plan. All data collected will be presented in data listings. Data from subjects excluded from an analysis population will be presented in the data listings, but not included in the calculation of summary statistics. Data from subjects receiving placebo in each cohort will be pooled into 1 group for analysis.

DocuSign Envelope ID: 774CDD36-E051-4E09-B4BF-7A852F085DB0



For categorical variables, frequencies and percentages will be presented. Continuous variables will be summarized using descriptive statistics (number of subjects, mean, median, SD, minimum, and maximum).

Baseline demographic and background variables will be summarized overall for all subjects. The number of subjects who enroll in the study and the number and percentage of subjects who complete the study will be presented. Frequency and percentage of subjects who withdraw or discontinue from the study, and the reason for withdrawal or discontinuation will also be summarized.

### 7.4.1 Pharmacokinetic Analyses

Plasma concentrations will be listed and summarized descriptively (number of subjects, arithmetic mean, SD, coefficient of variation [CV], median, geometric mean, geometric CV, minimum, and maximum). Plasma concentration versus actual time profiles for each subject will be presented graphically. The mean plasma concentration versus scheduled time profiles will be presented graphically. Individual urine concentration (and sample volumes) will be presented in data listings.

Plasma PK parameters derived from plasma samples using noncompartmental methods with Phoenix® WinNonlin® (Certara USA Inc., Princeton, New Jersey) Version 8.0 or higher or SAS Version 9.3 or higher (SAS Institute Inc., Cary, North Carolina) will be summarized by treatment using descriptive statistics (number of subjects, mean, SD, CV, geometric mean, geometric CV, median, minimum, and maximum). However, $T_{max}$ will be summarized using the following descriptive statistics only: number of subjects, median, minimum, and maximum. Urine PK parameters derived from urine samples will also be summarized by treatment using descriptive statistics (number of subjects, mean, SD, CV, geometric mean, geometric CV, median, minimum, and maximum).

To evaluate the effect of food on the PK of INS018_055 and metabolites, an analysis of variance model will be performed on the natural logarithms of AUCs and $C_{max}$ to calculate the ratio of geometric means and its 90% confidence interval (CI) between the fed group and the fasted group. Similarly, an ANOVA model will be performed on the natural logarithms of AUCs and $C_{max}$ to calculate the ratio of geometric means and its 90% CI between caffeine with INS018_055 (test) versus caffeine alone (reference). Wilcoxon Signed Rank test will be used to compare the $T_{max}$ between the fed group and the fasted group.

DocuSign Envelope ID: 774CDD36-E051-4E09-B4BF-7A852F085DB0



Dose proportionality will be tested on Day 1 and 7 using the power regression model for AUCs and $C_{max}$. Plots of AUCs and $C_{max}$ versus dose will be presented. The model is defined as:

$$\ln[\text{PK parameter}] = \beta_0 + \beta_1 \ln[\text{dose}]$$

where the PK parameter is an AUC or $C_{max}$. The null hypothesis being tested is that the AUCs and $C_{max}$ values are dose proportional, or slope ($\beta_1$) = 1.

Steady state will be tested by regression analysis of trough level concentrations of individual subjects from samples collected on Days 1 through 7. The model used to estimate the slope and corresponding 90% CI is:

$$C_{\text{trough}} = \beta_0 + \beta_1(\text{day} + \text{error})$$

Statistical evidence that the subject has not achieved steady state will be found if the slope is positive and significantly different from zero at the 5% level.

### 7.4.2 Safety Analyses

Adverse events will be coded by preferred term and system organ class using the latest version of the MedDRA. All AE data will be presented in a data listing. Treatment-emergent AEs will be summarized by treatment and overall, as well as by severity and relationship to study drug. Serious AEs, AEs leading to discontinuation of study drug, and death will also be presented in the data listings and summarized by treatment and overall.

Actual values and changes from baseline for clinical laboratory test results, vital sign measurements, and 12-lead ECG results will be summarized by treatment at each time point using descriptive statistics (number of subjects, mean, SD, median, minimum, and maximum). Shift tables will be generated for clinical laboratory test results. Frequency and percentage will be applied to summarize fecal occult blood results by visit. Clinical laboratory test results, vital sign measurements, 12-lead ECG results, and physical examination findings will be presented in data listings.

### 7.4.3 Biomarker Analyses

Summaries for all biomarker analysis will be based on the PD population. Summary statistics and change and percent change from baseline for all biomarkers will be presented by visit and time point. Last recheck values collected prior to the first dose of study treatment will be

DocuSign Envelope ID: 774CDD36-E051-4E09-B4BF-7A852F085DB0



considered as baseline and all rechecks will be excluded from after-dose observations in calculating summary statistics.

## 7.5 HANDLING OF MISSING DATA

Plasma/serum concentrations that are below the limit of quantification (BLQ) will be treated as zero for descriptive statistics. Mean BLQ concentrations will be presented as BLQ, and the SD and CV will be reported as not applicable. Missing concentrations will be excluded from the calculations.

For the PK analysis, BLQ values will be treated as zero with the exception that a BLQ value between 2 quantifiable concentrations will be set as missing. Missing concentrations will be treated as missing from the PK parameter calculations. If consecutive BLQ concentrations are followed by quantifiable concentrations in the terminal phase, those concentrations after BLQ concentrations will be treated as missing.

## 7.6 INTERIM ANALYSES

No formal interim safety analyses will be performed in this study.

DocuSign Envelope ID: 774CDD36-E051-4E09-B4BF-7A852F085DB0



# 8. REFERENCE LIST

Balogh A, Harder S, Vollandt R, et al. Intra-individual variability of caffeine elimination in healthy subjects. Int J Clin Pharmacol Ther Toxicol. 1992 Oct;30(10):383-7. Erratum in: Int J Clin Pharmacol Ther Toxicol. 1993;31(4):208.

Camara J, Jarai G. Epithelial-mesenchymal transition in primary human bronchial epithelial cells is Smad-dependent and enhanced by fibronectin and TNF-alpha. Fibrogenesis Tissue Repair. 2010;3(1):2.

Cao H, Wang C, Chen X, et al. Inhibition of Wnt/beta-catenin signaling suppresses myofibroblast differentiation of lung resident mesenchymal stem cells and pulmonary fibrosis. Sci Rep. 2018;8(1):13644.

Chapman H. Epithelial-mesenchymal interactions in pulmonary fibrosis. Annu Rev Physiol. 2011;73:413-35.

Chilosi M, Poletti V, Zamò A et al. Aberrant Wnt/beta-catenin pathway activation in idiopathic pulmonary fibrosis. Am J Pathol. 2003;162(5):1495-502.

Crawford HC, Fingleton BM, Rudolph-Owen LA, et al. The metalloproteinase matrilysin is a target of beta-catenin transactivation in intestinal tumors. Oncogene. 1999;18(18):2883-91.

Department of Health and Human Services (DHHS), Food and Drug Administration, Center for Drug Evaluation and Research (US). Guidance for Industry: Estimating the maximum safe starting dose in initial clinical trials for therapeutics in adult healthy volunteers. July 2005 [cited 2017 May 18]. Available from: http://www.fda.gov/downloads/Drugs/Guidances/UCM078932.pdf.

Hou J, Ma T, Cao H, et al. TNF-α-Induced NF-κB Activation Promotes Myofibroblast Differentiation of LR-MSCs and Exacerbates Bleomycin-Induced Pulmonary Fibrosis. J Cell Physio. 2018;233(3):2409-19.

Jenkins RG, Simpson JK, Saini G, et al. Longitudinal change in collagen degradation biomarkers in idiopathic pulmonary fibrosis: an analysis from the prospective, multicentre PROFILE study. Lancet Respir Med. 2015;3(6): 462–72.

Katzenstein AL, Myers JL. Idiopathic pulmonary fibrosis: clinical relevance of pathologic classification. Am J Respir Crit Care Med. 1998;157(4 Pt 1):1301-15.

DocuSign Envelope ID: 774CDD36-E051-4E09-B4BF-7A852F085DB0



Kim K, Kugler MC, Wolters PJ, et al. Alveolar epithelial cell mesenchymal transition develops in vivo during pulmonary fibrosis and is regulated by the extracellular matrix. Proc Natl Acad Sci USA. 2006;103(35):13180-5.

King TE Jr, Bradford WZ, Castro-Bernardini S, et al. A phase 3 trial of pirfenidone in patients with idiopathic pulmonary fibrosis. N Engl J Med. 2014;370(22):2083-92.

Lee JU, Cheong HS, Shim EY, et al. Gene profile of fibroblasts identify relation of CCL8 with idiopathic pulmonary fibrosis. Respir Res. 2017;18(1):3.

Lee Y, Jung JI, Park KY, et al. Synergistic inhibition effect of TNIK inhibitor KY-05009 and receptor tyrosine kinase inhibitor dovitinib on IL-6-induced proliferation and Wnt signaling pathway in human multiple myeloma cells. Oncotarget. 2017;8(25):41091-101.

Longo KA, Kennell JA, Ochocinska MJ, et al. Wnt signaling protects 3T3-L1 preadipocytes from apoptosis through induction of insulin-like growth factors. J Biol Chem. 2002;277(41):38239-44.

Mahmoudi T, Li VS, Ng SS, et al. The kinase TNIK is an essential activator of Wnt target genes. EMBO J. 2009;28(21):3329-40.

Morrisey EE. Wnt signaling and pulmonary fibrosis. Am J Pathol. 2003;162(5):1393-7.

Oda K, Yatera, K, Izumi, H, et al. Profibrotic role of WNT10A via TGF-β signaling in idiopathic pulmonary fibrosis. Respir Res. 2016;17:39.

Piguet PF, Ribaux C, Karpuz V, et al. Expression and localization of tumor necrosis factor-alpha and its mRNA in idiopathic pulmonary fibrosis. Am J Pathol. 1993;143(3):651-5.

Richeldi L, Du Bois RM, Raghu G, et al. Efficacy and safety of nintedanib in idiopathic pulmonary fibrosis. N Engl J Med. 2014;370(22):2071-82.

Rosas IO, Richards TJ, Konishi K, et al. MMP1 and MMP7 as potential peripheral blood biomarkers in idiopathic pulmonary fibrosis. PLoS Med. 2008;5(4):e93.

Selman M, King TE, Pardo A; American Thoracic Society; European Respiratory Society; American College of Chest Physicians. Idiopathic pulmonary fibrosis: prevailing and evolving hypotheses about its pathogenesis and implications for therapy. Ann Intern Med. 2001;134(2):136-51.

DocuSign Envelope ID: 774CDD36-E051-4E09-B4BF-7A852F085DB0



Shitashige M, Satow R, Jigami T, et al. Traf2- and Nck-interacting kinase is essential for Wnt signaling and colorectal cancer growth. Cancer Res. 2010;70(12):5024-33.

Shkoda A, Town JA, Griese J, et al. The germinal center kinase TNIK is required for canonical NF-κB and JNK signaling in B-cells by the EBV oncoprotein LMP1 and the CD40 receptor. PLoS Biol. 2012;10(8):e1001376.

Todd JL, Vinisko R, Liu Y, et al. Circulating matrix metalloproteinases and tissue metalloproteinase inhibitors in patients with idiopathic pulmonary fibrosis in the multicenter IPF-PRO Registry cohort. BMC Pulm Med. 2020; 20(1): 64. https://doi.org/10.1186/s12890-020-1103-4

Weigle S, Martin E, Voegtle A, et al. Primary cell-based phenotypic assays to pharmacologically and genetically study fibrotic diseases in vitro. J Biol Methods. 2019;6(2):e115.

Wu B, Crampton SP, Hughes CC. Wnt signaling induces matrix metalloproteinase expression and regulates T cell transmigration. Immunity. 2007;26(2):227-39.

Yamada T, Masuda M. Emergence of TNIK inhibitors in cancer therapeutics. Cancer Sci. 2017;108(5):818-23.

DocuSign Envelope ID: 774CDD36-E051-4E09-B4BF-7A852F085DB0



# 9. APPENDICES

## 9.1 APPENDIX 1: LIST OF ABBREVIATIONS

| Abbreviation | Term |
|---|---|
| α-SMA | α-smooth muscle actin |
| AE | adverse event |
| ALT | alanine aminotransferase |
| AR | accumulation ratio |
| AST | aspartate aminotransferase |
| AUC | area under the plasma concentration versus time curve |
| $AUC_{0-inf}$ | area under the plasma concentration versus time curve from time 0 extrapolated to infinity |
| $AUC_{0-\tau}$ | area under the plasma concentration versus time curve from time 0 to the time of the dosing interval |
| $AUC_{0-t}$ | area under the plasma concentration versus time curve from time 0 to the last quantifiable concentration |
| BID | twice daily |
| BLM | bleomycin |
| BLQ | below the limit of quantification |
| BP | blood pressure |
| $C_{av}$ | average concentration on Day 1 and Day 7 |
| CD | cluster of differentiation |
| CI | confidence interval |
| CKD-EPI | Chronic Kidney Disease Epidemiology Collaboration |
| CL/F | apparent total body clearance |
| CLr | renal clearance |
| $C_{max}$ | maximum observed plasma concentration |
| COVID-19 | Coronavirus disease 2019 |
| $C_{trough}$ | pre-dose concentrations on Days 1 through 7 |
| CV | coefficient of variation |
| CYP | cytochrome P450 |
| DDI | drug-drug interaction |
| ECM | extracellular matrix |
| ECG | electrocardiogram |
| eCRF | electronic case report form |
| EMT | epithelial to mesenchymal transition |
| EOS | end of study |
| $Fe\%_{0-t}$ | fraction of the dose excreted unchanged in urine over all time interval (0 to t) |
| FMT | fibroblast to myofibroblast transformation |
| GCP | Good Clinical Practice |
| GI | gastrointestinal |
| HIV | human immunodeficiency virus |
| IB | investigator's brochure |

DocuSign Envelope ID: 774CDD36-E051-4E09-B4BF-7A852F085DB0



| Abbreviation | Term |
|---|---|
| ICF | informed consent form |
| ICH | International Council for Harmonisation |
| IL-6 | interleukin 6 |
| IMP | investigational medicinal product |
| IP | investigational [medicinal] product |
| IPF | idiopathic pulmonary fibrosis |
| IRB | institutional review board |
| IUD | intrauterine device |
| $K_{el}$ | terminal elimination rate constant |
| MAD | multiple ascending dose |
| MATE | multidrug and toxin extrusion |
| MedDRA | Medical Dictionary for Regulatory Activities |
| MMP | matrix metalloproteinase |
| NOAEL | no observed adverse effect level |
| PD | pharmacodynamic |
| PK | pharmacokinetic(s) |
| QD | once daily |
| QTcF | QT interval by Fridericia |
| SAD | single ascending dose |
| SAE | serious adverse event |
| SARS-CoV-2 | severe acute respiratory syndrome-related coronavirus 2 |
| SCr | serum creatinine |
| SOE | schedule of events |
| SRC | safety review committee |
| $t_{1/2}$ | terminal phase half-life |
| TGF | transforming growth factor |
| $T_{max}$ | time to maximum observed plasma concentration |
| TNIK | Traf2- and Nck-interacting kinase |
| ULN | upper limit of normal |
| $V_d/F$ | apparent volume of distribution |
| $XU_{0-t}$ | total amount of drug excreted unchanged in urine over all time interval (0 to t) |

DocuSign Envelope ID: 774CDD36-E051-4E09-B4BF-7A852F085DB0



# 9.2 APPENDIX 2: STUDY GOVERNANCE

## 9.2.1 Data Quality Assurance

This study will be conducted using the quality processes described in applicable procedural documents. The quality management approach to be implemented will be documented and will comply with current ICH guidance on quality and risk management. All aspects of the study will be monitored for compliance with applicable government regulatory requirements, current GCP, the protocol, and standard operating procedures. The monitor will maintain current personal knowledge of the study through observation, review of study records and source documentation, and discussion of the conduct of the study with the investigator and staff. Electronic case report forms and electronic data capture will be utilized. The electronic data capture system is validated and compliant with National Regulatory Requirements. Each person involved with the study will have an individual identification code and password that allows for record traceability.

Important protocol deviations, should they occur during the study, will be presented in Section 10.2 of the clinical study report.

## 9.2.2 Investigator Obligations

The following administrative items are meant to guide the investigator in the conduct of the study and may be subject to change based on industry and government standard operating procedures, working practice documents, or guidelines. Changes will be reported to the IRB but will not result in protocol amendments.

### 9.2.2.1 Confidentiality

All laboratory specimens, evaluation forms, reports, and other records will be identified in a manner designed to maintain subject confidentiality. All records will be kept in a secure storage area with limited access. Clinical information will not be released without the written permission of the subject (or the subject’s legal guardian), except as necessary for monitoring and auditing by the sponsor, its designee, applicable regulators, or the IRB.

The investigator and all employees and coworkers involved with this study may not disclose or use for any purpose other than performance of the study, any data, record, or other unpublished, confidential information disclosed to those individuals for the purpose of the study. Prior written agreement from the sponsor or its designee must be obtained for the disclosure of any said confidential information to other parties.

### 9.2.2.2 Institutional Review

Federal regulations and ICH guidelines require that approval be obtained from an IRB before participation of human subjects in research studies. Before study onset, the protocol, ICF, advertisements to be used for the recruitment of study subjects, and any other written information regarding this study that is to be provided to the subject or the subject's legal guardian must be approved by the IRB. Documentation of all IRB approvals and of the IRB compliance with the ICH harmonized tripartite guideline E6(R2): GCP will be maintained by the site and will be available for review by the sponsor or its designee.

All IRB approvals should be signed by the IRB chairman or designee and must identify the IRB name and address, the clinical protocol by title or protocol number or both, and the date approval or a favorable opinion was granted.

### 9.2.2.3 Subject Consent

Written informed consent in compliance with ICH E6(R2) guidelines and applicable regulatory requirements shall be obtained from each subject before he or she enters the study or before performing any unusual or nonroutine procedure that involves risk to the subject. If any institution-specific modifications to study-related procedures are proposed or made by the site, the consent should be reviewed by the sponsor or its designee or both before IRB submission. Once reviewed, the investigator will submit the ICF to the IRB for review and approval before the start of the study. If the ICF is revised during the course of the study, all active participating subjects must sign the revised form.

Before recruitment and enrollment, each prospective subject or his/her legal guardian will be given a full explanation of the study and will be allowed to read the approved ICF. Once the investigator is assured that the subject/legal guardian understands the implications of participating in the study, the subject/legal guardian will be asked to give his or her consent to participate in the study by signing the ICF. A copy of the ICF will be provided to the subject/legal guardian.

### 9.2.2.4 Study Reporting Requirements

By participating in this study, the investigator agrees to submit reports of SAEs according to the timeline and method outlined in this protocol. In addition, the investigator agrees to submit annual reports to his or her IRB as appropriate.

DocuSign Envelope ID: 774CDD36-E051-4E09-B4BF-7A852F085DB0



#### 9.2.2.5 Investigator Documentation

Prior to beginning the study, the investigator will be asked to comply with ICH E6(R2) Section 8.2 by providing essential documents, including but not limited to, the following:

- IRB approval
- An original investigator-signed investigator agreement page of the protocol
- Curriculum vitae for the principal investigator and each subinvestigator. Current licensure must be noted on the curriculum vitae. Curriculum vitae will be signed and dated by the principal investigators and subinvestigators at study start-up, indicating that they are accurate and current.
- An IRB-approved ICF, samples of site advertisements for recruitment for this study, and any other written information about this study that is to be provided to the subject or legal guardians
- Laboratory certifications and reference ranges for any local laboratories used by the site

#### 9.2.2.6 Study Conduct

The investigator agrees to perform all aspects of this study in accordance with the ethical principles that have their origin in the Declaration of Helsinki, ICH E6(R2): GCP; the protocol; and all national, state, and local laws or regulations.

#### 9.2.2.7 Case Report Forms and Source Documents

Site personnel will maintain source documentation, enter subject data into the eCRF as accurately as possible, and will rapidly respond to any reported discrepancies.

Electronic CRFs and electronic data capture will be utilized. The electronic data capture system is validated and compliant with National Regulatory Requirements. Each person involved with the study will have an individual identification code and password that allows for record traceability. Thus, the system, and any subsequent investigative reviews, can identify coordinators, investigators, and individuals who have entered or modified records, as well as the time and date of any modifications. There may be an internal quality review audit of the data and additional reviews by the clinical monitor.

DocuSign Envelope ID: 774CDD36-E051-4E09-B4BF-7A852F085DB0



Each eCRF is presented as an electronic copy, allowing data entry by site personnel, who can add and edit data, add new subjects, identify and resolve discrepancies, and view records. This system provides immediate direct data transfer to the database, as well as immediate detection of discrepancies, enabling site coordinators to resolve and manage discrepancies in a timely manner.

Paper copies of the eCRFs and other database reports may be printed and signed by the investigator. This system provides site personnel, monitors, and reviewers with access to hardcopy audits, discrepancy reviews, and investigator comment information.

#### 9.2.2.8 Adherence to Protocol

The investigator agrees to conduct the study as outlined in this protocol, in accordance with ICH E6(R2) and all applicable guidelines and regulations.

#### 9.2.2.9 Reporting Adverse Events

By participating in this study, the investigator agrees to submit reports of SAEs according to the timeline and method outlined in this protocol. In addition, the investigator agrees to submit annual reports to his or her IRB as appropriate. The investigator also agrees to provide the sponsor with an adequate report, if applicable, shortly after completion of the investigator’s participation in the study.

#### 9.2.2.10 Investigator’s Final Report

Upon completion of the study, the investigator, where applicable, should inform the institution; the investigator/institution should provide the IRB with a summary of the study’s outcome and the sponsor and regulatory authorities with any reports required.

#### 9.2.2.11 Records Retention

Essential documents should be retained until at least 2 years after the last approval of a marketing application in an ICH region and until there are no pending or contemplated marketing applications in an ICH region or until at least 2 years have elapsed since the formal discontinuation of clinical development of the study drug. These documents should be retained for a longer period, however, if required by applicable regulatory requirements or by an agreement with the sponsor. The sponsor is responsible for informing the investigator/institution when these documents no longer need to be retained.

#### 9.2.2.12 Publications

After completion of the study, the data may be considered for reporting at a scientific meeting or for publication in a scientific journal. In these cases, the sponsor will be responsible for these activities and will work with the investigators to determine how the manuscript is written and edited, the number and order of authors, the publication to which it will be submitted, and any other related issues. The sponsor has final approval authority over all such issues.

Data are the property of the sponsor and cannot be published without their prior authorization, but data and any publication thereof will not be unduly withheld.

### 9.2.3 Study Management

#### 9.2.3.1 Monitoring

##### 9.2.3.1.1 Monitoring of the Study

The clinical monitor, as a representative of the sponsor, is obligated to follow the study closely. In doing so, the monitor will visit the investigator and study site at periodic intervals in addition to maintaining necessary telephone and email contact. The monitor will maintain current personal knowledge of the study through observation, review of study records and source documentation, and discussion of the conduct of the study with the investigator and staff.

All aspects of the study will be carefully monitored by the sponsor or its designee for compliance with applicable government regulation with respect to current ICH E6(R2) guidelines and standard operating procedures.

##### 9.2.3.1.2 Inspection of Records

The investigator and institution involved in the study will permit study-related monitoring, audits, IRB review, and regulatory inspections by providing direct access to all study records. In the event of an audit, the investigator agrees to allow the sponsor, their representatives, applicable regulatory agencies access to all study records.

The investigator should promptly notify the sponsor and study site(s) of any audits scheduled by any regulatory authorities and promptly forward copies of any audit reports received to the sponsor.

DocuSign Envelope ID: 774CDD36-E051-4E09-B4BF-7A852F085DB0



### 9.2.3.2 Management of Protocol Amendments and Deviations

#### 9.2.3.2.1 Modification of the Protocol

Any changes in this research activity, except those necessary to remove an apparent immediate hazard to the subject, must be reviewed and approved by the sponsor or designee. Amendments to the protocol must be submitted in writing to the investigator's IRB for approval before subjects are enrolled into an amended protocol.

#### 9.2.3.2.2 Protocol Deviations

The investigator or designee must document and explain in the subject's source documentation any deviation from the approved protocol. The investigator may implement a deviation from, or a change to, the protocol to eliminate an immediate hazard to study subjects without prior IRB approval. As soon as possible after such an occurrence, the implemented deviation or change, the reasons for it, and any proposed protocol amendments should be submitted to the IRB for review and approval, to the sponsor for agreement, and to the regulatory authorities, if required.

A protocol deviation is any change, divergence, or departure from the study design or procedures defined in the protocol. An important deviation (sometimes referred to as a major or significant deviation) is a subset of protocol deviations that leads to a subject being discontinued from the study, or significantly affects the subject's rights, safety, or well-being and/or the completeness, accuracy, and reliability of the study data. An important deviation can include nonadherence to inclusion or exclusion criteria or nonadherence to ICH E6(R2) guidelines.

Protocol deviations will be documented by the clinical monitor throughout the course of monitoring visits. The investigator will be notified in writing by the monitor of deviations. The IRB should be notified of all protocol deviations, if appropriate, in a timely manner.

### 9.2.3.3 Study Termination

Although the sponsor has every intention of completing the study, they reserve the right to discontinue it at any time for clinical or administrative reasons.

The end of the study is defined as the date on which the last subject completes the last visit (including the EOS visit and any additional long-term follow up). Any additional long-term follow up that is required for monitoring of the resolution of an AE or finding may be appended to the clinical study report.

DocuSign Envelope ID: 774CDD36-E051-4E09-B4BF-7A852F085DB0



#### 9.2.3.4 Final Report

Regardless of whether the study is completed or prematurely terminated, the sponsor will ensure that clinical study reports are prepared and provided to regulatory agency(ies) as required by the applicable regulatory requirement(s). The sponsor will also ensure that clinical study reports in marketing applications meet the standards of the ICH harmonized tripartite guideline E3: Structure and content of clinical study reports.

Where required by applicable regulatory requirements, an investigator signatory will be identified for the approval of the clinical study report. The investigator will be provided reasonable access to statistical tables, figures, and relevant reports and will have the opportunity to review complete study results.

Upon completion of the clinical study report, the investigator(s) will be provided with the final approved clinical study report, as appropriate.

DocuSign Envelope ID: 774CDD36-E051-4E09-B4BF-7A852F085DB0



## 9.3 APPENDIX 3: ESTIMATED GLOMERULAR FILTRATION RATE

The estimated GFR will be calculated using the 2 sets of equations developed by the CKD-EPI, which utilize SCr and serum Cystatin C, respectively.

CKD-EPI2009Scr

If female and SCr is <0.7 mg/dL:

- GFR (mL/min/1.73 $m^2$) = 144 × $(Scr/0.7)^{-0.329}$ × 0.993age (× 1.159, if Black)

If female and SCr is >0.7 mg/dL:

- GFR (mL/min/1.73 $m^2$) = 144 × $(Scr/0.7)^{-1.209}$ × 0.993age (× 1.159, if Black)

If male and SCr is <0.9 mg/dL:

- GFR (mL/min/1.73 $m^2$) = 141 × $(Scr/0.9)^{-0.411}$ × 0.993age (× 1.159, if Black)

If male and SCr is >0.9 mg/dL:

- GFR (mL/min/1.73 $m^2$) = 141 × $(Scr/0.9)^{-1.209}$ × 0.993age (× 1.159, if Black)

CKD-EPI2012cys

If female and Scys is <0.8 mg/L:

- GFR (mL/min/1.73 $m^2$) = 133 × $(Scys/0.8)^{-0.499}$ × 0.996age × 0.932

If female and Scys is >0.8 mg/L:

- GFR (mL/min/1.73 $m^2$) = 133 × $(Scys/0.8)^{-1.328}$ × 0.996age × 0.932

If male and Scys is <0.8 mg/L:

- GFR (mL/min/1.73 $m^2$) = 133 × $(Scys/0.8)^{-0.499}$ × 0.996age

If male and Scys is >0.8 mg/L:

- GFR (mL/min/1.73 $m^2$) = 133 × $(Scys/0.8)^{-1.328}$ × 0.996age

# Title Page

# CLINICAL STUDY PROTOCOL

## A Phase I, randomized, double-blind, placebo-controlled, single- and multiple-dose escalating oral administration, parallel-group study to evaluate the safety, tolerability, and pharmacokinetics of INS018_055 in healthy subjects

## INS018-055-002

| | |
|---|---|
| **Sponsor:** | Insilico Medicine Ltd. Room 368, Part 302, No. 211, Fute North Road, Free Trade Zone, China (Shanghai) |
| **Contact information for sponsors:** | Bei Zhang, MD<br>Director of Clinical Development<br>Tel: +86 18691523273 |
| **Medical Monitor:** | Tigermed Group<br>Medical Monitor Li Hanyang<br>E-mail: hanyang.li@tigermedgrp.com |
| **Protocol version:** | V2.0 |
| **Protocol Date:** | October 21, 2022 |

## CONFIDENTIAL

The concepts and information contained in this document or generated during the study are proprietary and may not be disclosed in whole or in part without the express written consent of Insilico Medicine (Shanghai).

This study will be conducted in accordance with the International Conference on Harmonization of Technical Requirements for Registration of Pharmaceuticals for Human Use Guideline E6 (R2): Good Clinical Practice (GCP), the Declaration of Helsinki (2013 edition) and the National Drug Administration (NMPA) GCP (2020 edition).

# SIGNATURE PAGE

| | |
|---|---|
| **Protocol Title:** | A Phase I, randomized, double-blind, placebo-controlled, single- and multiple-dose escalating oral administration, parallel-group study to evaluate the safety, tolerability, and pharmacokinetics of INS018_055 in healthy subjects |
| **Protocol no:** | INS018-055-002 |

| | |
|---|---|
| Bei Zhang, MD<br>Director of Clinical Development<br>Insilico Medicine (Shanghai) | Date |

# INVESTIGATOR PROTOCOL AGREEMENT PAGE

I have carefully read this study protocol and acknowledge that it contains all the necessary information required to conduct this study. I agree to conduct the study in accordance with this protocol (including any amendments) and in accordance with the Clinical Research Center Standard Operating Procedures, the International Council for Harmonization of Technical Requirements for Registration of Pharmaceuticals for Human Use Code of Practice for Quality Management of Pharmaceutical Clinical Trials, all other applicable regulations, and the recommendations in the latest edition of the Declaration of Helsinki.

________________________________________ ______________________

Signature of Principal Investigator Date

________________________________________

Printed name of principal investigator

# 签名页

**方案标题：** 一项在健康受试者中评价 INS018_055 安全性、耐受性和药代动力学的 I 期、随机、双盲、安慰剂对照、单次和多次剂量递增口服给药、平行组研究

**方案编号：** INS018-055-002

张北 2022.10.21

Bei Zhang, MD
临床开发医学部总监
英矽智能科技（上海）有限公司

日期

# 研究者方案协议页

本人已仔细阅读了本研究方案，并确认本方案包含开展本研究所需的所有必要信息。本人同意根据本方案（包括任何修正案）并按照临床研究中心标准操作规程、人用药品注册技术要求国际协调会药物临床试验质量管理规范、所有其他适用法规以及最新版赫尔辛基宣言中的建议开展研究。

[illegible] 2022.10.21

主要研究者签名 日期

[illegible]

主要研究者的印刷体姓名

## PROTOCOL VERSION HISTORY

| Version number | Version Date | Revision Summary |
|---|---|---|
| V1.0 | May 09, 2022 | Initial Version |
| V2.0 | October 21, 2022 | 1. Modify the queue settings for the MAD: from "30 mg QD/BID, 60 mg QD/BID" to "30 mg BID, 60 mg BID";<br>2. Removal of 100 mg QD/BID and addition of 90 mg BID and 120 mg QD dose groups;<br>3. The addition of 90 mg BID and 120 mg QD using sentinel dosing and detailed description;<br>4. Addition of section 1.3.3 Clinical rationale for maximum dose selection;<br>5. Details Revision. |

# Table of Content

INS018-055-002 ... 2
SIGNATURE PAGE ... 3
INVESTIGATOR PROTOCOL AGREEMENT PAGE ... 4
PROTOCOL SYNOPSIS ... 8
Study phase: I ... 8
Objectives of the study: ... 8
Study design: ... 8
Part A (single incremental dose administration [SAD]): ... 8
Part B (multiple dose escalation dosing [MAD]): ... 9
Study population: ... 10
Exclusion criteria: ... 13
Study treatment: ... 16
Study procedures: ... 16
Biomarker assessment and endpoints: ... 18
Safety assessment and endpoints: ... 18
Statistical analysis plan: ... 18
Analysis set: ... 18
Pharmacokinetic analysis: ... 19
Biomarker analysis: ... 19
Security analysis: ... 19
1. INTRODUCTION ... 22
1.1 BACKGROUND ... 22
1.2 RATIONALE FOR STUDY ... 25
1.3 RATIONALE FOR DOSE SELECTION ... 27
1.3.1 Preclinical animal toxicology data ... 27
1.3.2 Clinical rationale for starting dose selection ... 29
Table 3 : Summary of PK parameters of INS018_055 after a single dose in healthy subjects ... 29
Table 4 Summary of adverse events in healthy subjects given a single dose of INS018_055 ... 29
1.3.3 Clinical basis for maximum dose selection ... 30
Table 5 : Summary of PK parameters of INS018_055 after a single dose in healthy subjects in New Zealand and China ... 31
Table 6 : Summary of PK parameters of INS018_055 after multiple dosing in healthy subjects in New Zealand and China ... 32
Table 7 Summary of adverse events in healthy New Zealand subjects after 120 mg QD administration INS018_055 ... 33
2. STUDY OBJECTIVE ... 31
2.1 Primary objective ... 31
2.2 Secondary objective ... 31
3. STUDY DESIGN ... 31
Part A (SAD) ... 31
Part B (MAD) ... 32
3.1 SCHEDULE OF EVENTS ... 34
Table 3.1 -Table 3.11 Schedule of Research Activities for Part A (SAD) ... 35
Table 3.1 -Table 3.12 Schedule of Research Activities for Part B (MAD) ... 37
4. STUDY POPULATION ... 40
4.1 INCLUSION CRITERIA ... 40
4.2 EXCLUSION CRITERIA ... 41
4.3 OTHER SCREENING CONSIDERATIONS ... 44

4.3.1 Lifestyle restrictions ..... 44
4.4 SUBJECTS WITHDREW FROM THE STUDY ..... 47
4.4.1 Reason for withdrawal ..... 47
4.4.2 Handling of withdrawals ..... 48
Lost to follow up ..... 48
4.4.3 Replacement ..... 49
5. RESEARCH TREATMENT ..... 49
5.1 TREATMENT GIVEN ..... 49
Table 5.1 -Table 5.11 Choice of drug delivery method and timing of drug administration ..... 50
5.1.1 Dose Escalation ..... 50
5.1.2 Dose escalation stopping criteria ..... 52
5.2 EXPERIMENTAL DRUGS ..... 53
5.2.1 Study drug packaging and storage ..... 53
5.2.2 Study Drug Inventory ..... 54
5.3 METHOD OF ASSIGNING SUBJECTS TO TREATMENT GROUPS ..... 54
5.4 BLINDING ..... 55
5.4.1 Blinding procedure ..... 55
5.4.2 Emergency Unblinding and Formal Unblinding ..... 55
5.5 TREATMENT COMPLIANCE ..... 55
5.5.1 Prior and Concomitant Medications ..... 55
6. STUDY PROCEDURES ..... 56
6.1 PHARMACOKINETIC EVALUATION AND ENDPOINTS ..... 56
6.1.1 Pharmacokinetic sample collection ..... 59
6.1.2 Pharmacokinetic sample analysis ..... 59
6.2 SAFETY ASSESSMENT AND ENDPOINTS ..... 59
6.2.1 Adverse Events ..... 59
6.2.2 Clinical Laboratory Tests ..... 63
6.2.3 Vital Signs Measurement ..... 64
6.2.4 Electrocardiogram ..... 65
6.2.5 Physical examination ..... 65
6.3 BIOMARKERS ..... 66
6.3.1 CD4+ and CD8+ subpopulations of T cells ..... 66
6.3.2 IL-6 and TGF-β ..... 66
6.3.3 MMP-2, MMP-9 and MMP-7 ..... 67
7. STATISTICAL ANALYSIS PROTOCOL ..... 67
7.1 SAMPLE SIZE CALCULATION ..... 67
7.2 ANALYSIS SET ..... 67
7.3 DESCRIPTION OF THE SUBGROUP TO BE ANALYZED ..... 67
7.4 STATISTICAL ANALYSIS ..... 68
7.4.1 Pharmacokinetic analysis ..... 68
7.4.2 Safety Analysis ..... 69
7.4.3 Biomarkers Analysis ..... 69
7.5 MID-TERM ANALYSIS ..... 69
8. REFERENCE LIST ..... 70
9. PROTOCOL REVISION RECORD ..... 73
10. APPENDIX ..... 74
10.1 APPENDIX 1 : LIST OF ABBREVIATIONS ..... 74
10.2 APPENDIX 2 : STUDY MANAGEMENT ..... 76
10.2.1 Data Quality Assurance ..... 76
10.2.2 Investigator Obligations ..... 76
10.2.3 Study Management ..... 79
10.3 APPENDIX 3: COCKCROFT -GAULT EQUATIO ..... 81
10.4 APPENDIX 4: PROHIBITED COMBINED MEDICATIONS ..... 82

*2 Appendix ...... 3*
2.1 Appendix 1 SAS code example ...... 3
2.2 Appendix 2 Tables, Tables, Charts ...... 3

# PROTOCOL SYNOPSIS

**Protocol number:** INS018-055-002

**Title:** A phase 1, randomized, double-blind, placebo-controlled, single- and multiple-dose escalating oral administration, parallel-group study to evaluate the safety, tolerability, and pharmacokinetics of ins018_055 in healthy subjects

**Study phase:** I

**STUDY SITE(S):** 1 clinical research center in China

**Objectives of the study:**

The primary objectives of this study are:

- The primary objective of this study is to assess the safety and tolerability of single and multiple oral ascending doses of INS018_055 administered to healthy subjects

The secondary objectives of this study were:

- To determine the pharmacokinetic (PK) profile of INS018_055 following single and multiple oral escalating doses in healthy subjects.
- Assessment of the effect of single and multiple dose escalation oral administration of INS018_055 on biomarkers (T cell CD4+ and CD8+ subpopulations, interleukin [IL]-6, transforming growth factor [TGF]-β, matrix metalloproteinases [MMP-2, MMP-9, MMP-7]) in healthy subjects (as a measure of pharmacological activity)

**Study design:**

This is a phase I, randomized, 2-part (Part A and Part B), double-blind, placebo-controlled, single- and multiple-dose escalation study designed to evaluate the safety, tolerability, and PK characteristics of INS018_055 in healthy subjects receiving oral administration.

**Part A (single incremental dose administration [SAD]):**

Eight healthy subjects will be assigned to each of 3 consecutive dose cohorts (30, 60, 120 mg). On Day 1, subjects in each dose cohort will be randomized in a 3:1 ratio (6 active, 2 placebo) to receive either INS018_055 or matching placebo. Approximately 24 subjects will be enrolled in this part. Subjects in Part A will be admitted to the study site on Day -1, fasted (no food or drink except water) for at least 10 hours, and then

administered on Day 1. No water will be allowed 1 hour before and 1 hour after dosing. Water will be allowed at all other times. Subjects will remain fasted for 4 hours after dosing on Day 1. Subjects will leave the study site on Day 4 after the investigator (or designee) has completed the scheduled procedure and reviewed the safety data on Day 4. Subjects will return to the center for an end-of-study (EOS) visit on Day 8 (± 1 day).

Starting with cohort 1 (initial dose cohort), 2 subjects in each dosing cohort will be selected to form a sentinel cohort to reduce the risk of unanticipated adverse events (AEs) in each cohort, that not predicted by preclinical pharmacology and toxicology studies. The sentinel subjects will be dosed in a blinded fashion (1 receiving active drug and 1 receiving placebo) and monitored for at least 1 day before the remaining 6 subjects in that cohort were dosed. Initiation of dosing of the remaining 6 subjects will depend on the results of the investigator's initial safety review (indicating that the study treatment is safe and well-tolerated in the sentinel subjects).

Dose escalation will occur only after real-time PK, safety and tolerability data (including reported AEs, physical examination, vital signs, electrocardiogram (ECG) and clinical laboratory results up to 48 hours of dosing) of the preceding dose cohort for at least 6 subjects are assessed and the study treatment is deemed safe and well tolerated by the safety review committee (SRC). As new safety and/or PK data become available, the anticipated dose escalation scheme may change following review of the data by the SRC. Preliminary PK data from subjects in this study will help guide further dose escalation.

**Part B (multiple dose escalation dosing [MAD]):**

Healthy subjects will be assigned to four continuous dose cohorts to receive twice-daily (BID) or once-daily (QD) administration (BID, 30, 60, 90 mg; QD, 120 mg), with 8 subjects in each cohort. Subjects in each dose cohort will be randomized in a 3:1 ratio (6 active, 2 placebo) to receive either INS018_055 or matching placebo for 7 days, where 2 subjects from each of the 90 mg BID and 120 mg QD cohorts are selected to form a sentinel cohort, and the sentinel subjects will receive dosing in a blinded fashion (1 to receive active drug and 1 to receive placebo), and will be monitored for at least 1 day. The timing of dosing initiation for the remaining 6 subjects will depend on the results of the investigator's initial safety review (indicating that the study treatment is safe and well tolerated in the sentinel subjects). Approximately 32 subjects will be enrolled in this part.

During the conduct of Part A, PK data, safety and tolerability data (including reported AE, physical examination, clinical laboratory tests, 12-lead ECG, and vital signs) are dynamically evaluated, and if the steady-state exposures of the MAD cohort are assessed to be within the exposure range of the dose groups for which the SAD has been completed, and the SRC deems the study treatment safe and well tolerated, Part B may be conducted in parallel. The timing and dosing regimen (QD or BID) for Part B will be determined by the SRC.

Subsequent dose escalations in Part B will not be performed until safety (including AE, physical examination, clinical laboratory findings, vital signs, and 12- lead ECG) and tolerability data (up to and including Day 11) have been evaluated in at least 6 subjects from the preceding MAD dose cohort and the SRC has deemed the study

treatment safe.

As new safety or PK data become available, the dose escalation scheme may change. Instead of receiving a subsequent higher dose, subjects may receive a lower dose administration or may be administered at the same daily dose under a different dosing regimen (e.g., BID dosing instead of QD dosing) to achieve a lower maximum observed plasma concentration ($C_{max}$) value, particularly if a safety finding is believed to be linked to the $C_{max}$ values.

Each subject in Part B will be admitted to the study site on Day -1 and will begin receiving once-daily dosing on Day 1. The study drug will be administered at approximately the same time each morning. For the BID dosing (30 mg, 60 mg and 90 mg dose groups), the study treatment will be given again approximately 12 hours after the study treatment is given in the morning.

Subjects in cohorts 4 through 7 will fast (no food or drink except water) overnight for at least 10 hours before dosing on the morning of days 1 and 7, and for an additional 4 hours after study treatment administration. Drinking is not permitted 1 hour before and 1 hour after dosing. Water is allowed at all other times. When the study treatment is administered under the BID regimen, water is not restricted for evening dosing. On Days 1 and 7, lunch will be provided approximately 4 hours after morning dosing, dinner will be provided approximately 10 hours after morning dosing, and snacks will be provided approximately 13 hours after morning dosing.

On days 2 through 6, for cohorts 4 through 7, breakfast will be served approximately 60 minutes after study treatment administration. Subjects will be provided with a standard meal throughout the study period. Lunch will be provided approximately 4 hours after morning dosing, dinner will be provided approximately 10 hours after morning dosing, and a snack will be provided approximately 13 hours after morning dosing.

Physical examination, vital sign measurements, 12-lead ECG and clinical laboratory evaluation (including liver function test results) will be performed at selected time points throughout the dosing interval. Subjects will be closely monitored for AE throughout the study period and blood samples for PK analysis will be collected within 72 hours of study drug administration on Day 7. For biomarker analysis, blood samples will be collected at selected time points for the QD and BID dosing cohorts (see Table 3.1-2 for details). Fecal occult blood testing will be performed on all stool samples at screening, registration (Day -1) and throughout the study site observation period in Part B (i.e., up to Day 11).

Subjects will leave the study site on Day 11 after the investigator (or designee) has completed the scheduled procedure and reviewed the safety data. Subjects will return to the study site for an EOS visit on Day 28 (± 3 days).

**Study population:**

**Inclusion criteria:**

Each subject must meet all of the following criteria to be enrolled in this study:

1. Subjects were male or female between 18 and 45 years of age, inclusive.

2. Subjects had a body mass index of 19 to 26 kg/m$^2$ (inclusive) and weighed ≥ 50 kg (inclusive) for men and ≥ 45 kg (inclusive) for women at the time of screening.

3. Based on medical history, clinical laboratory findings, vital sign measurements, 12-lead ECG results, physical examination, chest radiograph results, and serum virological findings at screening, the investigators concluded that the subjects were in good general health.

4. Female subjects of childbearing potential must be non-pregnant and non-lactating and must be using one of the following methods of contraception throughout the treatment period until at least 28 days after the last dose of study drug, or have been surgically sterilized (i.e., hysterectomy, bilateral tubal ligation, or bilateral oophorectomy) or are postmenopausal (defined as 12 consecutive months of amenorrhea with documented plasma follicle stimulating hormone levels > 40 IU/mL). Female subjects must have a negative pregnancy test result at screening and prior to the first dose of study drug.

A highly effective method of contraception is one that has a contraceptive failure rate of less than 1% per year when used consistently. Examples are as follows:

a. Implanted contraceptives (e.g., Jadelle® )

b. IUDs containing copper or levonorgestrel (e.g., Mirena® )

c. Male sterilization, no sperm in ejaculation after vasectomy

d. Double barrier method: condom and occlusion cap (diaphragm or cervical cap/dome cap), barrier method with spermicide (foam/gel/film/cream/suppository) must be used as an add-on

e. Abstinence, defined as complete and continuous avoidance of all heterosexual sex (including during the entire period of risk associated with study treatment), was allowed without contraception only if this was the subject's preferred and daily lifestyle.

or an effective method with a contraceptive failure rate of less than 5% to 10% per year. Examples are as follows:

f. Injectable contraceptives (e.g., Depo Provera)

g. Oral contraceptives (combination hormonal contraceptives or progestogen-only "mini-pills")

h. Vaginal contraceptive ring (e.g., NuvaRing® )

Female subjects must also agree not to donate eggs from the time of administration until at least 28 days after the last dose of study drug.

Male subjects and their fertile female partners must agree to use one of the above methods of contraception for the entire treatment period until at least 28 days after the last dose of study drug. Male subjects must also agree not to donate sperm for the entire treatment period until at least 28 days after the last dose of study drug.

5. Subjects agree to comply with all protocol requirements.

6. Subjects were able to provide written informed consent.

**Exclusion criteria:**

Subjects who met any of the following criteria were excluded from the study:

1. Subject has current evidence or history of clinically significant hematologic, renal, endocrine, pulmonary, gastrointestinal, cardiovascular, hepatic, psychiatric, neurologic, or allergic disease (including drug allergy, but excluding asymptomatic seasonal allergy that was untreated at the time of administration).

2. Subjects have any condition that may affect drug absorption (e.g., gastrectomy).

3. Subjects had a history of cancer, except adequately treated basal cell or squamous cell carcinoma of the skin.

4. The subject rests for at least 5 minutes with a blood pressure (BP) > 140 mm Hg (systolic) or > 90 mm Hg (diastolic). At screening, if BP is > 140 mm Hg (systolic) or > 90 mm Hg (diastolic), BP should be measured 2 additional times and the average of the 3 BP values should be used to determine the subject's eligibility to participate.

5. At screening, the subject's 12-lead ECG shows a QT interval (QTc) corrected by the Bazett formula ($QTc=QT/RR^{0.5}$ ) > 450 msec, or a QRS interval > 120 msec. The average of three QTc (or QRS interval) values from three standard 12-lead ECGs (repeated at intervals of no more than 5 minutes) should be used to determine the subject's eligibility for participation.

6. At the time of screening, the subject has any of the following abnormalities in clinical laboratory tests (if necessary, retest once for confirmation):

    a. At screening, serum creatinine levels above the upper limit of normal (ULN) or creatinine clearance (Ccr) < 80 mL/min using the Cockcroft - Gault formula (Appendix 3) and no protein in the urine.

    b. Aspartate aminotransferase or alanine aminotransferase values > 1.5 × ULN.

    c. Fasting glucose > 110 mg/dL (6.1 mmol/L).

    d. Total bilirubin > 1.5 × ULN.

e. Routine blood test values that are outside the normal reference range of local laboratory findings and are considered clinically significant by the investigator.

f. Positive fecal occult blood test result at screening or registration (day -1).

7. Subjects have a history of any disease that may have caused total bilirubin to be higher than ULN. Subjects whose clinical laboratory test values are not significantly outside the reference range may be enrolled in this study if the investigator does not consider the values to be clinically significant.

   Note: In subjects with a history of Gilbert's syndrome, direct bilirubin may be measured, and if direct bilirubin < ULN, the subject is eligible for this study.

8. Subjects have any history of lymphoproliferative disease (such as Epstein Barr virus-associated lymphoproliferative disease as reported by some subjects receiving immunosuppressive drugs), lymphoma, leukemia, myeloproliferative disease, multiple myeloma, or signs and symptoms suggestive of current lymphatic disease.

9. Subjects have a history of relevant drug and/or food allergies (i.e., allergy to any study drug or excipient, or any severe food allergy that could result in inability to consume the standard diet of the clinical institution).

10. Subject has a current or clinically significant infection (e.g., an infection requiring hospitalization or parenteral antimicrobial therapy or the presence of an opportunistic infection within the past 6 months) or a history of chronic or recurrent infectious disease within the 6 months prior to the first dose of study drug.

11. Subject has other serious acute or chronic medical or psychiatric illness (including recent (within the past year) or active suicidal ideation or behavior or abnormal experimental results (which may increase the risk associated with study participation or experimental drug administration or may interfere with the interpretation of study results and, in the judgment of the investigator, may render the subject unsuitable for entry into this study).

12. Subjects have a history of symptomatic herpes zoster or herpes simplex, more than one episode of localized herpes zoster, or disseminated herpes zoster (single episode) present or within 12 weeks.

13. Subjects tested positive for hepatitis B surface antigen, hepatitis C virus antibody, or human immunodeficiency virus type 1 or 2 antibody at screening.

14. Subjects were pregnant or lactating females.

15. Subjects are men of childbearing potential who are unwilling or unable to use the contraceptive methods described in this protocol throughout the study period and for at least 28 days after the last dose of the experimental drug.

16. Subjects are unwilling or unable to comply with the lifestyle restrictions described in this protocol.

17. Subjects were smokers or had used nicotine or nicotine-containing products (e.g., snus, nicotine patches, nicotine chewing gum, simulated cigarettes, or inhalants) within 6 months prior to the first dose of the study drug.

18. Subjects tested positive for substance abuse or cotinine (indicating current active smoking) prior to the first dose of study drug.

19. Subjects have used any prescription or over-the-counter medication (except paracetamol [up to 2 g/day]), including herbal supplements, within 14 days prior to the first dose of study drug. Nutritional supplements are permitted provided that they are unlikely to interfere with the study results and that investigator consent has been obtained.

20. Subjects ingested grapefruit or grapefruit juice, limes or products containing limes (e.g., orange marmalade) or products containing alcohol, caffeine or xanthines within 48 hours prior to the first dose of the study drug.

21. Subjects will be vaccinated with live virus, live attenuated virus, or any live viral component within 2 weeks prior to the first dose of study drug, or will receive these vaccines at any time during the study or within 8 weeks of study completion.

22. Subject tested positive for Severe Acute Respiratory Syndrome-associated Coronavirus 2 (SARS-CoV-2). Subject received the 2019 coronavirus disease (COVID-19) vaccine within 2 weeks prior to the first dose of study drug or is scheduled to receive the COVID-19 vaccine within 12 weeks of study drug administration, or tested positive for SARS-CoV-2 during screening or had COVID-19 symptoms within 4 weeks prior to Day -1.

23. Subjects who have undergone major trauma or major surgery within 4 weeks prior to screening or who are expected to require major surgery during the trial.

24. Subjects are at risk for bleeding: genetic predisposition to bleeding, a bleeding event within 12 months prior to screening start, or abnormal laboratory coagulation parameters.

25. Subjects have a first-degree relative with a genetic immunodeficiency.

26. Subjects were study site staff and their family members who were directly involved in the implementation of the study, study site staff who were otherwise supervised by the investigator, or sponsor employees (including their family members) who were directly involved in the implementation of the study.

27. Subject has a history of alcohol abuse or drug addiction or excessive alcohol consumption (regular alcohol intake > 21 units/week for male subjects and > 14 units/week for female subjects; 1 unit equals approximately ½ pint [200

mL] of beer, 1 small glass [100 mL] of wine, or 1 cup [25 mL] of spirits) within the past year or consumed alcohol 24 hours prior to the first dose of study drug.

28. Subjects engaged in strenuous activity or contact sports within 24 hours prior to dosing and during the study.

29. The subject donated > 450 mL of blood or blood products within 30 days prior to the first dose of the study drug.

30. Subjects received study drug in another pilot study within 30 days prior to dosing or 5 drug half-lives, whichever is longer.

31. Subjects received cytochrome P450 (CYP3A4 and CYP2C8) and P-gp inhibitors and/or inducers within 4 weeks prior to the first dose of INS018_055 or may have received CYP3A4 and CYP2C8 and P-gp inhibitors and/or inducers during the study.

32. Subjects were deemed by the investigator to be unsuitable for enrollment in the study.

**Study treatment:**

**Part A**: Within each dose cohort, subjects will receive one of the study treatments (INS018_055 or matching placebo in a 3:1 ratio) on Day 1 based on the following random assignment: 30 mg INS018_055 or placebo (cohort 1), 60 mg or placebo (cohort 2), 120 mg or placebo (cohort 3)

**Part B**: For all 4 cohorts (30 mg BID or placebo [Cohort 4], 60 mg BID or placebo [Cohort 5], 90 mg BID or placebo [Cohort 6], 120 mg QD or placebo [Cohort 7]), within each dose cohort, subjects will receive BID or QD study treatment on Days 1 through 7 according to the following random assignment (INS018 _055 or matching placebo in a 3:1 ratio, where 2 subjects in each of the 90 mg BID and 120 mg QD cohorts are selected to form a sentinel cohort, and the sentinel subjects will be administered in a blinded fashion (1 receiving active drug and 1 receiving placebo) and monitored for at least 1 day before the remaining 6 subjects in the cohort receive dosing.

The dosing regimen (BID or QD) will be determined by the SRC after review of PK, safety and tolerability data from Part A. Subsequent dose escalations for Part B will be based on review of safety and tolerability data (up to and including Day 11) for at least 6 subjects in the previous MAD dose cohort and when the SRC deems the study treatment safe.

**Study procedures:**

**Pharmacokinetic evaluation and endpoints:**

**Part A**: Blood samples for PK analysis of INS018_055 and its metabolites (INS018_063 and INS018_095) will be collected at the following time points on Day 1: before (0 hours) and after administration of INS018_055 0.25, 0.5, 1, 2, 4, 6, 8, 10

and 12 hours ; 24 and 36 hours (Day 2); 48 hours (Day 3) and 72 hours (Day 4). (day 3) and 72 hours (day 4).

**Part B**: For QD administration, blood samples for PK analysis of INS018_055 and its metabolites (INS018_063 and INS018_095) will be collected at the following time points:

- Day 1: Before (0 h) and 0.25, 0.5, 1, 2, 4, 6, 8, 10 and 12 h after dosing.
- Days 2, 3, 4, 5, and 6: before dosing.
- Day 7: 0.25 (hour 0) and 0.25, 0.5, 1, 2, 4, 6, 8, 10, 12, 24 (day 8), 48 (day 9) and 72 (day 10) hours before (hour 0) and after dosing.

For BID administration, blood samples for PK analysis of INS018_055 and its metabolites (INS018_063 and INS018_095) will be collected at the following time points:

- Day 1: 0.25, 0.5, 1, 2, 4, 6, 8, 10, and 12 hours before (0 hr) and 0.25, 0.5, 1, 2, 4, 6, 8, 10, and 12 hours after morning dosing (i.e., 0 hr before evening dosing).
- Days 2, 3, 4, 5 and 6: Before morning dosing.
- Day 7: 0.25, 0.5, 1, 2, 4, 6, 8, 10, 12, 24 (Day 8), 48 (Day 9), and 72 (Day 10) hours before (0 hours) and after evening dosing.

The following plasma PK parameters were calculated as endpoints for INS018_055 and its metabolites (INS018_063 and INS018_095) using the actual sampling time rather than the planned sampling time:

- Area under the plasma concentration versus time curve (AUC) from time 0 to the last quantifiable concentration ($AUC_{0-t}$)
- AUC from time 0 extrapolated to infinity ($AUC_{0-inf}$)
- AUC (τ; $AUC_{0-\tau}$ ) from time 0 to the time of the dosing interval
- $AR_{AUC}$ calculated as $AUC_{0-t}$ (day 7)/$AUC_{0-t}$ (day 1)
- $AR_{Cmax}$ calculated as $C_{max}$ (day 7)/$C_{max}$ (day 1)
- Maximum observed plasma concentration $(C\ )_{max}$
- Time to reach $C_{max}$ $(T\ )_{max}$
- Pre-dose concentration ($_{Ctrough}$) from day 1 to day 7
- Average concentrations on days 1 and 7 $(C\ )_{av}$

- Terminal elimination rate constant ($K_{el}$)
- Terminal elimination half-life (t )$_{1/2}$
- Apparent total body clearance (CL/F)
- Peak to trough ratio calculated as Cmax/Ctrough
- Apparent volume of distribution ($V_d$ /F)
- Metabolite-to parent ratio based on AUC calculated as $AUC_{metabolite}/AUC_{parent}$
- Metabolite-to parent ratio based on $C_{max}$ calculated as $C_{max, metabolite}/C_{max, parent}$

**Biomarker assessment and endpoints:**

Blood concentrations of T-cell subsets (CD4+ and CD8+), interleukin [IL]-6, transforming growth factor [TGF]-β, and matrix metalloproteinases [MMP-2, MMP-9, MMP-7] will be assessed.

**Safety assessment and endpoints:**

Safety and tolerability will be assessed by the following endpoints: AE monitoring and recording, clinical laboratory results (routine blood, coagulation, blood biochemistry, urine routine and fecal occult blood test), vital sign measurements, 12-lead ECG results and physical examination results.

**Statistical analysis plan:**

**Sample size:**

The number of subjects was determined based on clinical and practical considerations, rather than on formal statistical certainty calculations. The total sample size of 56 subjects was considered sufficient for the purpose of the study.

**Analysis set:**

Safety Analysis Set (SS): The safety population will include all randomized enrolled subjects who receive at least 1 dose of INS018_055 or placebo.

PK Analysis Set (PKS): The PK population will include subjects who receive at least 1 dose of INS018_055 with concentration data sufficient to support accurate estimation of at least 1 PK parameter. Subjects who receive study drug administration followed by vomiting within 2 times the median time of $T_{max}$ will be excluded from the PK analysis.

Pharmacodynamic Analysis Set (PDS): The PD population will include subjects receiving at least 1 dose of INS018_055 or placebo with validated baseline (pre-dose) values and at least 1 non-deficient biomarker measure.

**Pharmacokinetic analysis:**

Plasma concentrations will be listed and summarized in a descriptive manner (number of subjects, arithmetic mean, SD, coefficient of variation [CV], median, geometric mean, geometric CV, minimum and maximum values). The plasma concentration-actual time curve for each subject will be displayed graphically. The mean plasma concentration-scheduled time curve will be displayed graphically.

PK parameters for plasma samples were derived using non-atrial methods and summarized by each treatment group using descriptive statistics (number of subjects, mean, SD, CV, geometric mean, geometric CV, median, minimum and maximum values). However, only the following descriptive statistics will be used to summarize $T_{max}$ : number of subjects, median, minimum and maximum values.

Dose proportionality will be examined using a Power Model. Plots of AUC and $C_{max}$ versus dose will be provided. The model is defined as:

$$\ln[\text{PK parameter}] = \beta_0 + \beta_1 \ln[\text{dose}]$$

where the PK parameter is AUC or $C_{max}$ . The null hypothesis tested is that the AUC and $C_{max}$ values vary proportionally with dose, or that the slope ($\beta_1$) = 1.

Steady state was assessed by repeated comparisons of individual subjects' trough concentrations in samples collected from day 1 to day 7 by Mixed Model analysis. For this analysis, the trough concentration values were log-transformed and then compared with the log-transformed trough concentration values at that time point and all other trough concentration values after that time point. The GeoLSM ratios and 90% confidence intervals were summarized.

**Biomarker analysis:**

Summary of biomarker analyses will be performed based on the PD population. Data from subjects who received placebo in each cohort will be pooled into 1 group for analysis.

Summary statistics, changes, and percent change relative to baseline will be presented for each biomarker (T cell CD4+ and CD8+ subpopulations, interleukin [IL]-6, transforming growth factor [TGF]-β, matrix metalloproteinases [MMP-2, MMP-9, MMP-7]) by visit and time point. The last review value collected before the first dose of study treatment will be considered as baseline, and all recheck values will be excluded from post-dosing observations when calculating the summary statistics.

**Security analysis:**

Adverse events will be coded by preferred terminology and system organ

classification using the latest version of the Medical Dictionary of Regulatory Activities.

All AE data will be presented in a data list. AEs occurring during treatment will be summarized by treatment group and subject as a whole, as well as by severity and relationship to study drug. severe AEs, AEs leading to early discontinuation, and death will also be listed in the data list.

Actual values and changes relative to baseline for clinical laboratory test results, vital sign measurements, and 12-lead ECG results are summarized by treatment group at each time point using descriptive statistics (number of subjects, mean, SD, median, minimum, and maximum). A table of changes in clinical laboratory results will be generated. The number and percentage of subjects will be summarized by visit for fecal occult blood results. Clinical laboratory results, vital sign measurements, 12-lead ECG results, and physical examination results are presented in the data list.

**Protocol date:** October 21, 2022

# 1. INTRODUCTION

## 1.1 BACKGROUND

Idiopathic pulmonary fibrosis (IPF) is a fatal lung disease, characterized by distorted lung architecture and loss of respiratory function as a result of alveolar epithelial cell injury and hyperplasia, enhanced extracellular matrix (ECM) deposition, and myofibroblast activation. It has been reported that epithelial to mesenchymal transition (EMT) is one of the major drivers of fibrosis with approximately 30% of ECM-producing myofibroblasts being derived from epithelial cells through EMT *(***Error! Reference source not found.** *et al 2006;* **Error! Reference source not found.** *2011;* **Error! Reference source not found.** *et al 2019)*. Fibroblast to myofibroblast transformation (FMT) is another important contributor to IPF pathology. Activated myofibroblast promote excessive ECM deposition resulting in characteristic fibroblast foci lesions (**Error! Reference source not found.**and Myers 1998). The occurrence and number of fibroblast foci can be correlated with survival (**Error! Reference source not found.** et al 2017).

Fibroblast foci occur in subepithelial layers close to areas of alveolar epithelial cell injury and repair suggesting that impaired epithelial-mesenchymal crosstalk contributes to the pathobiology of IPF. It is generally accepted that repetitive injury and subsequent repair of alveolar epithelial cells, in the presence or absence of local inflammation, represent a key pathogenic mechanism in IPF. This mechanism leads to aberrant growth factor activation and perpetuation of fibrotic transformation (**Error! Reference source not found.** et al 2001). Transforming growth factor (TGF)-β1 (TGF-β1) is known to be a key fibrogenic driver of both EMT and FMT. Additionally, tumor necrosis factor-α (TNF-α) as an important proinflammatory cytokine, is expressed by macrophages and epithelial cells in the lungs of patients with IPF and has been shown to promote TGF-β1 induced disease progression in the scenario of IPF *(***Error! Reference source not found.***et al 1993;* **Error! Reference source not found.** *and Jarai 2010)*. Although several soluble mediators, such as TGF-β1 or interleukin-1b, have a clear pathogenic role in IPF and are possible therapeutic targets (**Error! Reference source not found.** et al 2001), no pharmaceutical has been successful in clinical use till date. Two treatments (pirfenidone and nintedanib) targeting the biologic processes that drive fibrosis are approved for the treatment of IPF. While each attenuates the rate of lung function decline, neither halts disease progression nor affects long-term survival *(***Error! Reference source not found.** *et al 2014;* **Error! Reference source not found.** *et al 2014)*.

Although molecular characterization of IPF shows complex interdependence of various mechanisms, the Wnt pathway is consistently identified as a major activated signaling

cascade that significantly contributes to the pathogenesis of lung fibrosis (**Error! Reference source not found.** et al 2003; **Error! Reference source not found.** 2003; **Error! Reference source not found.**et al 2009; **Error! Reference source not found.** and Masuda 2017).

The serine/threonine kinase Traf2- and Nck-interacting kinase (TNIK), is an essential activator of Wnt target genes (**Error! Reference source not found.**et al 2009; **Error! Reference source not found.** et al 2010) and interacts with the T-cell factor /lymphoid enhancer factor and β-catenin transcriptional complex, directly mediating an important signaling mechanism of the Wnt/β-catenin pathway in its downstream part (**Error! Reference source not found.** and Masuda 2017). The Wnt/β-catenin signaling pathway also partially mediates the fibrogenic effect of TGF-β (**Error! Reference source not found.** et al 2016; **Error! Reference source not found.** et al 2018)

Persistently activated NF-κB pathway is involved in a variety of inflammatory diseases that can especially mediate the progression of pulmonary fibrosis (**Error! Reference source not found.** et al 2018). One of the upstream regulators of NF-κB signal are TRAF proteins, which are known to cooperate with TNIK (**Error! Reference source not found.** et al 2012)

Therefore, TNIK is a feasible target of pharmacologic intervention for manipulation of the Wnt signaling pathway and engages other IPF-related signaling pathways including NF-κB and TGF-β signaling pathways. Inhibition of TNIK is expected to be potentially effective to repress the signaling events even in those cases when the upstream regulators are mutated.

*INS018_055 is a potent inhibitor of the TNIK and is currently being developed by InSilico Medicine Hong Kong Limited as an orally administered potential treatment for IPF in adult humans.* INS018_055 showed potent inhibition of TNIK in a biochemical enzymatic assay with a half-maximal inhibitory concentration ($IC_{50}$) value of 23 nM. Furthermore, INS018_055 inhibited few other kinases that are known to be involved in pro-fibrotic pathways (ie, Alk4, DDR1, Fms, PDGFR, TGFBR1).

INS018_055 also potently inhibited TGF-β induced expression of α-smooth muscle actin (α-SMA) and collagen I in the lung fibroblast cell line MRC-5. In an in vitro translational cell-based model, INS018_055 showed concentration dependent inhibition of TGF-β1-mediated α-SMA expression (in FMT assay) and fibronectin expression (in EMT assay) in cells derived from IPF patients, with no or minimal signs of cytotoxicity or anti-proliferative effects at the concentrations tested.

In an in vivo efficacy model of bleomycin (BLM)-induced lung fibrosis, INS018_055 demonstrated potent efficacy and prevented BLM-induced lung fibrosis dose-dependently.

An intravenous toxicity study in mice did not reveal serious acute or delayed toxicity at single doses of 1, 3, and 10 mg/kg, and oral, single-dose maximum tolerated dose studies in mice and dogs showed a good tolerability of INS018_055 in doses up to 300 mg/kg.

Pivotal 28-day toxicity studies in mice and dogs showed dose-dependent toxicity from 60 mg/kg/day in mice and 30 mg/kg/day in dogs. The lymphohematopoietic system and the gastrointestinal (GI) tract were the major target organs of toxicity. Dose-dependent increases were also seen in physis width as well as cortical and trabecular bone thickness, that may represent (extended) pharmacological effects of INS018_055 on chondro-/osteoclast activity. In addition, there was no evidence for genotoxicity with compound INS018_055 and its 2 metabolites in a valid battery of GLP-compliant in vitro tests that included bacterial reverse mutation assay in five strains of *Salmonella typhimurium*

(TA97a, TA98, TA100, TA102, and TA1535) and the chromosome aberration test in Chinese Hamster Lung cell. There was also no evidence of clastogenic and/or aneugenic potential of compound INS018_055 in an GLP-compliant in vivo micronucleus test in peripheral blood reticulocytes following oral administration. Safety pharmacology studies for central nervous, respiratory, cardiovascular, or GI systems were performed in vitro or in vivo following oral administration of compound INS018_055 at pharmacological and supra-pharmacological doses. Compound INS018_055 had no apparent effects on physiological functions of the central nervous system, cardiovascular, respiratory, or GI systems. Compound INS018_055 and their 2 metabolites also did not produce significant effect on the cardiac repolarization, in vitro or in vivo.

For further information on INS018_055, please refer to the investigator's brochure (IB) for INS018_055.

The preclinical data generated so far along with the bioinformatic analysis provides a strong rationale to pursue INS018_055 as a clinical candidate for IPF.

The overall safety profile of INS018_055 in nonclinical studies supports clinical evaluation of safety, tolerability, pharmacokinetic (PK) profile, and biomarker responses to single and multiple (up to 7 days) oral administrations of INS018_055 in the dose ranges specified in this protocol in healthy subjects. Importantly, the adverse effects observed in nonclinical studies at high multiple of exposures are monitorable in the setting of the current clinical study. All subjects will be informed of the potential risks prior to enrollment in the study.

## 1.2 RATIONALE FOR STUDY

The purpose of this study is to provide safety, tolerability, PK and biomarker data for future clinical studies in healthy subjects receiving initial single and multiple dose escalation dosing of INS018_055. Plasma concentration analysis will determine the PK characteristics of single and multiple doses of INS018_055 (including metabolites INS018_063 and INS018_095) and will help refine dosing strategies in future studies.

This is a 2-part (A and B), continuous, dose-escalation study in healthy subjects. In Part A of the study, the continuous dose escalation healthy subject cohort will receive a single oral dose of INS018_055. In Part B, a continuous twice-daily (BID) or once-daily (QD) dosing cohort will be evaluated in healthy subjects after 7 days of oral administration of INS018_055.

After dynamic assessment of PK data, safety and tolerability data (including reported adverse events [AEs], physical examinations, clinical laboratory findings, 12-lead electrocardiograms [ECGs] and vital signs) during the conduct of Part A, if the steady-state exposure of the MAD cohort is assessed to be within the exposure range of the dose group for which the SAD has been completed and the SRC deems the study

treatment safe and well tolerated, Part B may be conducted in parallel. The timing and dosing regimen (BID or QD) for Part B will be determined by the SRC.

Subsequent dose escalations in Part B will not be performed until safety (including AE, physical examination, clinical laboratory findings, vital signs, and 12- lead ECG) and tolerability data (up to and including Day 11) have been evaluated in at least 6 subjects from the previous MAD dose cohort and the SRC has deemed the study treatment safe.

# 1.3 RATIONALE FOR DOSE SELECTION

## 1.3.1 Preclinical animal toxicology data

The human equivalent dose (HED) was calculated based on a no-observed-adverse-effect dose (NOAEL) of 30 mg/kg/day in the most sensitive animal test species mice, and this value was scaled down by a factor of 10 to obtain a maximum recommended starting dose of 0.24 mg/kg as the initial clinical dose for the first human trial. Therefore, the clinical starting dose for Part A of the New Zealand Phase I study was defined as 10 mg, which is equivalent to 0.24 mg/kg in a 60 kg adult.

Using the FDA Guidance for Industry "Estimating the Maximum Recommended Starting Dose of a Drug in a First Clinical Trial in Healthy Adult Volunteers ( DHHS 2005)", the following formula was used to calculate the HED using the animal NOAEL dose levels and animal body weight as described in the FDA Guidance for Industry, "Estimating the Maximum Recommended Starting Dose of a Drug in Healthy Adult Volunteers in First Clinical Trials ( DHHS 2005):

$$\text{HED} = \text{animal dose (mg/kg)} \times \left[\frac{\text{animal weight (kg)}}{\text{human weight (kg)}}\right]^{0.33}$$

**Table 1 Toxicology experiments of single dose: INS018_055**

| Study Number | MTD (mg/kg) | Gender | $AUC_{last}$ (h*ng/mL) |
|---|---|---|---|
| S21073AD2 (Mouse, PO) | 300 | Females | 25600 |
| | | Males | 48100 |
| S21073AD1 (Dog, PO) | 100 | Females | 12300 |
| | 300 | Males | 16500 |

**Table 2 Toxicology experiments of INS018_055 repeatedly administered for 4 weeks**

| Study Number | NOAEL (mg/kg/day) | Gender | Day 1 | | Day 28 | |
|---|---|---|---|---|---|---|
| | | | $AUC_{last}$ (h*ng/mL) | First $C_{max}$ /Second $C_{max}$ (ng/mL) | $AUC_{last}$ (h*ng/mL) | First $C_{max}$ /Second $C_{max}$ (ng/mL) |
| | | Females | 2350 | 225/419 | 3820 | 481/494 |

| | | | | | | |
|---|---|---|---|---|---|---|
| S21073RD1 (Mouse, PO, BID) | 30 | Males | 2680 | 256/414 | 4440 | 469/752 |
| | | | | | | |
| | | | **$AUC_{last}$ (h*ng/mL)** | **$C_{max}$ (ng/mL)** | **$AUC_{last}$ (h*ng/mL)** | **$C_{max}$ (ng/mL)** |
| S21073RD2 (Dog, PO, BID) | 10 | Females | 1040 ± 589 | 380 ± 161 | 2010 ± 337 | 970 ± 177 |
| | | Males | 1940 ± 1260 | 859 ±630 | 2600 ±1010 | 1320 ± 626 |

### 1.3.2 Clinical rationale for starting dose selection

A Phase I, randomized, double-blind, placebo-controlled, oral single and multiple escalating dose, parallel group study evaluating the safety, tolerability and PK profile of INS018_055 in healthy subjects is currently underway in New Zealand. The pharmacokinetic parameters and safety profile of the 10 mg and 30 mg cohorts as of the end of April 2022 are summarized below:

**Table 3 : Summary of PK parameters of INS018_055 after a single dose in healthy subjects**

| INS018_055 | | Queue | |
|---|---|---|---|
| **Parameters (unit)** | **Statistical quantities** | **10 mg (N=6)** | **30 mg (N=6)** |
| $C_{max}$ (ng/mL) | Mean ± SD | 29.0 ± 14.4 | 127 ± 46.0 |
| | GeoMean ($CV_b$ %) | 25.4 (66.8) | 121 (38.7) |
| $T_{max}$ (h) | Median | 1.00 | 1.00 |
| | Min, Max | 1.00, 2.00 | 1.00, 2.00 |
| $AUC_{0-t}$ (h*ng/mL) | Mean ± SD | 141 ± 70.7 | 597 ± 152 |
| | GeoMean ($CV_b$ %) | 123 (69.6) | 580 (29.5) |
| $AUC_{0-inf}$ (h*ng/mL) | Mean ± SD | 152 ± 71.9 | 604 ± 153 |
| | GeoMean ($CV_b$ %) | 135 (62.1) | 587 (29.3) |
| $t_{1/2}$ (h) | Mean ± SD | 10.2 ± 2.56 | 7.72 ± 0.620 |
| | GeoMean ($CV_b$ %) | 9.97 (26.4) | 7.70 (7.80) |
| CL/F (L/h) | Mean ± SD | 86.1 ± 59.3 | 52.8 ± 16.7 |
| | GeoMean ($CV_b$ %) | 73.9 (62.1) | 51.1 (29.3) |
| $V_d$ /F (L) | Mean ± SD | 1340 ± 1200 | 582 ± 158 |
| | GeoMean ($CV_b$ %) | 1060 (75.7) | 568 (25.5) |

INS018_055 After single doses of 10 mg and 30 mg, the mean drug-time curves showed a rapid absorption phase of the drug, with peak concentrations reaching around 1 h after dosing ($T_{max}$ median), and observable concentration data until 48 h after dosing, when the drug concentration decreased with di-phase elimination.

**Table 4 Summary of adverse events in healthy subjects given a single dose of INS018_055**

| Queue | Subjects# | Adverse Events | Severity | Need for combined medication | Is suspected to be related to the study drug | Ending |
|---|---|---|---|---|---|---|
| 10 mg | 101-006 | Vasovagal syncope | Mild | No | Yes | Recovery |

| | | | | | | |
|---|---|---|---|---|---|---|
| | | Fatigue | Mild | No | Yes | Recovery |
| 30 mg | 101-023 | Left forearm phlebitis | Mild | No | No | Recovery |

Safety data through the end of April 2022 showed that only one subject in the 10 mg cohort experienced two adverse events, vasovagal syncope and fatigue, which were judged by the investigator to be mild and related to the study drug, with a recovery outcome; only one subject in the 30 mg cohort experienced one case of left forearm phlebitis, which was judged by the investigator to be a mild adverse event, unrelated to the study drug , related to venous blood collection, with an outcome of recovery. The overall safety and tolerability of healthy subjects receiving a single dose of INS018_055 in the 10-30 mg dose range was good, with no severe adverse events.

Based on the above non-clinical and clinical data, the exposure of the 30 mg cohort of healthy subjects for a single dose ($AUC_{0-t}$ ) was much lower than the preclinical single dose toxicology studies in mice and dogs under MTD (see Table 1 for details) and lower than the exposure of beagle dogs in non-clinical toxicology studies at NOAEL on day 28 of repeated dosing (see Table 2 for details). In addition, the safety and tolerability in healthy New Zealand subjects at a single dose of 30 mg was good. Therefore, the starting dose was set at 30 mg for the single dose escalation phase in domestic Phase I healthy subjects.

### 1.3.3 Clinical basis for maximum dose selection

To date, INS018_055 has been administered in New Zealand in single increments of 30 mg, 60 mg, 90 mg, 120 mg dose groups and multiple increments of 30 mg, 60 mg, 120 mg dose groups in QD. Also, INS018_055 has been administered in China in single increments of 30 mg, 60 mg, 120 mg dose groups and multiple increments of 30 mg dose groups.

Based on the PK characteristics of INS018_055 obtained in healthy Chinese and New Zealand subjects, the time to peak ($T_{max}$), exposure ($C_{max}$, AUCs), and elimination half-life of INS018_055 in plasma after single administration of INS018_055 30 mg, 60 mg (as shown in Table 5), or multiple administration of 30 mg (BID/QD, as shown in Table 6) ($t_{1/2}$) did not differ significantly by race in healthy Chinese and New Zealand subjects.

**Table 5 : Summary of PK parameters of INS018_055 after a single dose in healthy subjects in New Zealand and China**

| INS018_055 | | Queue | | | | | | | |
|---|---|---|---|---|---|---|---|---|---|
| Parameters (Unit) | Statistical quantities | SAD-30mg (N=6) | | SAD-60mg (N=6) | | SAD-90mg (N=6) | SAD-90mg fed* (N=6) | SAD-120mg (N=6) | SAD-120mg (N=6) |
| | | New Zealand | China | New Zealand | China | New Zealand | New Zealand | New Zealand | China |
| $C_{max}$ (ng/mL) | Mean ± SD | 127 ± 46.0 | 118.4 ± 23.4 | 297 ± 132 | 251 ± 150 | 291 ± 130 | 180 ± 67.1 | 354 ± 116 | 679 ± 168.2 |
| | GeoMean ($CV_b$ %) | 121 (38.7) | 116.4 (20.9) | 273 (47.5) | 218 (62.1) | 270 (44.1) | 169 (42.8) | 339 (32.1) | 664 (23.0) |
| $T_{max}$ (h) | Median | 1.00 | 1.00 | 1.00 | 1.00 | 1.00 | 3.00 | 1.50 | 1.00 |
| | Min, Max | 1.00, 2.00 | 0.50, 2.00 | 0.50, 2.00 | 1.00, 2.00 | 0.50, 2.00 | 2.00, 4.00 | 1.00, 2.00 | 1.00, 2.00 |
| $AUC_{0-t}$ (h*ng/mL) | Mean ± SD | 597 ± 152 | 566 ± 136 | 1360 ± 641 | 1196 ± 513 | 1720 ± 597 | 1490 ± 537 | 2100 ± 782 | 3877 ± 674 |
| | GeoMean ($CV_b$ %) | 580 (29.5) | 554 (21.7) | 1210 (60.6) | 1111 (43.6) | 1620 (40.4) | 1390 (44.9) | 2000 (34.1) | 3829 (17.4) |
| $AUC_{0-inf}$ (h*ng/mL) | Mean ± SD | 604 ± 153 | 577 ± 142 | 1370 ± 649 | 1206 ± 514 | 1730 ± 599 | 1500 ± 538 | 2120 ± 783 | 3896 ± 675 |
| | GeoMean ($CV_b$ %) | 587 (29.3) | 564 (22.2) | 1220 (60.8) | 1121 (43.2) | 1630 (40.3) | 1400 (44.6) | 2020 (34.0) | 3848 (17.3) |
| $t_{1/2}$ (h) | Mean ± SD | 7.72 ± 0.620 | 7.24 ± 1.44 | 7.73 ± 1.23 | 7.79 ± 1.22 | 9.47 ± 4.74 | 10.2 ± 3.37 | 9.35 ± 2.73 | 11.4 ± 2.72 |
| | GeoMean ($CV_b$ %) | 7.70 (7.80) | 7.14 (17.9) | 7.64 (16.7) | 7.71 (15.4) | 8.61 (49.0) | 9.69 (35.4) | 9.03 (29.3) | 11.0 (30.4) |
| CL/F (L/h) | Mean ± SD | 52.8 ± 16.7 | 54.2 ± 10.5 | 56.4 ± 33.9 | 57.2 ± 21.7 | 59.0 ± 24.8 | 69.8 ± 34.5 | 62.1 ± 18.2 | 31.6 ± 5.33 |
| | GeoMean ($CV_b$ %) | 51.1 (29.3) | 53.2 (22.2) | 49.1 (60.8) | 53.5 (43.3) | 55.2 ± 40.3 | 64.2 (44.6) | 59.5 (34.0) | 31.2 (17.3) |
| $V_d$ /F (L) | Mean ± SD | 582 ± 158 | 550 ± 56.3 | 583 ± 255 | 648 ± 293 | 708 ± 186 | 917 ± 210 | 835 ± 274 | 514 ± 147 |
| | GeoMean ($CV_b$ %) | 568 (25.5) | 548 (10.0) | 542 (43.3) | 595 (48.1) | 686 (29.3) | 898 (22.7) | 776 (50.6) | 495 (31.9) |

Notes:
*SAD-90mg fed was the postprandial dosing group, and all other dose groups were administered on an empty stomach.

**Table 6 : Summary of PK parameters of INS018_055 after multiple dosing in healthy subjects in New Zealand and China**

| INS018_055 | | | Queue | | | |
|---|---|---|---|---|---|---|
| **Parameters (unit)** | **Statistical quantities** | **Visits** | **30 mg QD (N=6)** | **30 mg BID (N=6)** | **60 mg QD (N=6)** | **120 mg QD (N=6)** |
| | | | **New Zealand** | **China** | **New Zealand** | **New Zealand** |
| $C_{max}$ (ng/mL) | Mean ± SD | D1 | 109 ± 32.2 | 141.5 ± 29.4 | 232 ± 56.6 | 515 ± 66.7 |
| | | D7 | 87.0 ± 42.5 | 50.4 ± 8.06 | 201 ± 69.1 | 476 ± 125 |
| $T_{max}$ (h) | Median | D1 | 1.00 | 1.00 | 1.00 | 1.50 |
| | | D7 | 1.00 | 4.00 | 1.50 | 2.00 |
| $AUC_{0-t}$ (h*ng/mL) | Mean ± SD | 1 | 425 ± 156 | 476 ± 74.4 | 1180 ± 293 | 3130 ± 803 |
| | | 7 | 505 ± 203 | 766 ± 167 | 1390 ± 391 | 3460 ± 889 |
| AUC ($_{0-tau}$ h*ng/mL ) | Mean ± SD | 1 | 425 ± 156 | 476 ± 74.4 | 1180 ± 293 | 3130 ± 803 |
| | | 7 | 465 ± 179 | 766 ± 167 | 1250 ± 358 | 3120 ± 812 |
| $t_{1/2}$ (h) | Mean ± SD | 1 | 5.82 ± 0.50 | 4.25 ± 0.80 | 5.63 ± 0.93 | 5.72 ± 0.52 |
| | | 7 | 9.53 ± 2.07 | 8.73 ± 1.39 | 11.7 ± 1.46 | 10.2 ± 1.14 |
| CL/F (L/h) | Mean ± SD | 1 | 75.0 ± 23.1 | 56.0 ± 7.75 | 51.7 ± 12.6 | 38.4 ± 8.87 |
| | | 7 | 72.5 ± 26.7 | 70.8 ± 13.0 | 51.5 ± 15.0 | 40.5 ± 9.59 |
| $V_d$ /F (L) | Mean ± SD | 1 | 623 ± 172 | 342.6 ± 71.9 | 421 ± 137 | 319 ± 93.0 |
| | | 7 | 962 ± 317 | 885.3 ± 169.4 | 858 ± 243 | 606 ± 189 |
| $R_{Cmax}$ | Mean ± SD | / | 0.76 ± 0.20 | 0.36 ± 0.07 | 0.88 ± 0.29 | 0.92 ± 0.19 |
| $R_{AUC}$ | Mean ± SD | / | 1.03 ± 0.21 | 0.92 ± 0.14 | 1.06 ± 0.06 | 1.00 ± 0.04 |

In healthy New Zealand subjects, after a single administration of 90 mg of INS018055 (as shown in Table 5), peak concentrations (arithmetic mean: 291 ng/mL) were reached around 1 hour after dosing (median $T_{max}$ ), with observable concentration data until 72 hours after dosing, when drug concentrations decreased with diel elimination, with arithmetic mean values of 1720 h*ng/mL for $AUC_{0-t}$ and 1730 h*ng/mL for $AUC_{0-inf}$ , respectively. 1730 h*ng/mL.

In healthy Chinese subjects, after a single administration of 120 mg of INS018055 (as shown in Table 5), peak concentrations (arithmetic mean: 679 ng/mL) were reached around 1 h ($T_{max}$ median) after dosing, and observable concentration data were available until 72 h after dosing, when drug concentrations decreased with two-phase elimination, with arithmetic means of 3877 h*ng/mL for $AUC_{0-t}$ and 3896 h*ng/mL for AUC0-inf, respectively. /mL and 3896 h*ng/mL, respectively.

In healthy New Zealand subjects, after multiple doses of 120 mg (QD) of INS018_055 (as shown in Table 6), peak concentrations (arithmetic mean: 476 ng/mL) were reached around 2 hours ($T_{max}$ median) after dosing on day 7, with observable concentration data until 72 hours after dosing, when drug concentrations declined with diphasic elimination, with arithmetic means of $AUC_{0-t}$ and AUC0-tau of The arithmetic means of $AUC_{Cmax}$ and AUC0-tau were 3460 h*ng/mL and 3120 h*ng/mL, respectively; the arithmetic means of R and $R_{AUC}$ were 0.92 and 1.00, respectively, suggesting no significant accumulation.

**Table 7 Summary of adverse events in healthy New Zealand subjects after 120 mg QD administration INS018_055**

| Queue | Subjects# | Adverse Events | Severity | Need for combined medication | Is suspected to be related to the study drug | Ending |
|---|---|---|---|---|---|---|
| 120mg QD | 101-089 | Positive fecal occult blood | Mild | No | Yes | Recovery |
| | | Headaches | Mild | Yes | Yes | Recovery |
| | | Elevated muscle enzymes | Mild | No | Yes | Recovery |
| | 101-090 | Intermittent nausea | Mild | No | Yes | Recovery |
| | | Headaches | Mild | No | Yes | Recovery |
| | | Positive fecal occult blood | Mild | No | Yes | Recovery |
| | 101-093 | runny nose | Mild | No | No | Recovery |
| | | Functional gastrointestinal disorders | Mild | No | Yes | Recovery |
| | 101-094 | Intermittent nightmares | Mild | No | Yes | Recovery |
| | | Headaches | Mild | Yes | Yes | Recovery |

| Queue | Subjects# | Adverse Events | Severity | Need for combined medication | Is suspected to be related to the study drug | Ending |
|---|---|---|---|---|---|---|
| | 101-096 | Erythema nodosum of the skin | Mild | No | No | Recovery |
| | 101-098 | Inflammation at the site of intravenous indwelling needle | Mild | No | No | Recovery |
| | | Elevated ALT/AST | Mild | No | Yes | Recovery |
| | 101-099 | Headaches | Mild | No | Yes | Recovery |

Safety data through the end of September 2022 (as shown in Table 7) showed that all adverse events reported in the New Zealand 120 mg QD cohort were mild; no combination medications were used for adverse events except for 2 subjects who took combination medications for mild headache; and all mild adverse events had a recovered outcome. The overall safety and tolerability of healthy subjects receiving INS018_055 120 mg QD administered continuously for 7 days was good, with no serious adverse events.

Based on the above clinical data in healthy subjects in China and New Zealand, combined with the non-clinical data in Section 1.3.1, the steady-state exposure ($AUC_{0-tau}$ ) after 7 days of continuous dosing in healthy subjects in the Chinese 90 mg BID cohort will be similar to the $AUC_{0-inf}$ for 90 mg SAD in healthy subjects in New Zealand, and the daily exposure will not exceed the PK exposure for the Chinese 120 mg SAD dose (90 mg BID at steady-state for 24 h is twice the 90 mg SAD $AUC_{0-inf}$ at approximately 3460 h*ng/mL) and is close to the PK exposure at the maximum dose in New Zealand (120 mg QD), which is well below the preclinical single toxicology study exposure in mice and dogs at MTD (see Table 1 for details).Table 1 ) and lower than the preclinical toxicology study in mice at NOAEL on day 28 of repeated dosing (see Table 1 for details).Table 2 ).

The steady-state exposure ($AUC_{0-tau}$ ) after 7 days of continuous dosing in healthy subjects in the Chinese 120 mg QD cohort would be comparable to the exposure of 120 mg SAD in healthy Chinese subjects, while QD administration was essentially nonaccumulative based on the New Zealand 30-120 mg QD results; the steady-state exposure ($AUC_{0-tau}$ ) after 7 days of continuous dosing in healthy subjects in the 120 mg QD cohort may be slightly higher than the exposure of 120 mg SAD in healthy New Zealand subjects. The steady-state exposure (AUC ) after 7 days of continuous administration in healthy subjects in the 120 mg QD cohort may be slightly higher than the 120 mg QD exposure in healthy New Zealand subjects, but is essentially comparable to the 120 mg SAD exposure in healthy Chinese subjects and much lower than the preclinical single toxicology study in mice and dogs at MTD (see Table 1 for details).Table 1 ), and lower than the preclinical toxicology study in mice at NOAEL on day 28 of repeated dosing (see Table 1 for details).Table 2 ). In addition, the dose of 120 mg QD was safe and well tolerated in healthy New Zealand subjects. Therefore,

the maximum dose in domestic Phase I healthy subjects was set at 120 mg QD during multiple dose escalation phases.

# 2. Study Objective

## 2.1 Primary objective

The primary objectives of this study are:

- To evaluate the safety and tolerability of single and multiple dose escalation oral administration of INS018_055 in healthy subjects.

## 2.2 Secondary objective

The secondary objectives of this study are:

- To determine the PK characteristics of INS018_055 in healthy subjects after receiving single and multiple incremental oral doses of the drug.
- Healthy subjects were evaluated for the effects of single and multiple dose increments of oral administration on biomarkers (T-cell CD4+ and CD8+ subpopulations, interleukin [IL]-6, transforming growth factor [TGF]-β, matrix metalloproteinases [MMP-2, MMP-9, MMP-7]) (as a measure of pharmacological activity).

# 3. STUDY DESIGN

This is a phase I, randomized, 2-part (Part A and Part B), double-blind, placebo-controlled, dose-escalation study designed to evaluate the safety, tolerability, and PK characteristics of healthy subjects receiving oral administration of INS018_055.

The study will include a screening period, enrollment, treatment period, and end-of-study (EOS) visit.

**Part A (SAD)**

Healthy subjects were assigned to 3 consecutive dose cohorts (30, 60, 120 mg), 8 in each cohort. On Day 1, subjects in each dose cohort will be randomized in a 3:1 ratio (6 active, 2 placebo) to receive either INS018_055 or matching placebo, enrolling approximately 24 subjects in this arm. subjects in Part A will be admitted to the study site on Day -1, fast (no food or drink except water) for at least 10 hours, and then receive dosing on Day 1. No water will be allowed 1 hour before and 1 hour after dosing. Water will be allowed at all other times. Subjects will fast for an additional 4 hours after dosing on Day 1. Subjects will leave the study site on Day 4 after the investigator (or designee) has completed the scheduled procedure and reviewed the Day 4 safety data. Subjects will return to the center for an EOS visit on Day 8 (± 1 day).

Starting with cohort 1 (initial dose cohort), 2 subjects in each dosing cohort will be selected to form a sentinel cohort to reduce the risk of unintended AEs not predicted by preclinical pharmacology and toxicology studies in each cohort. The sentinel subjects will be administered in a blinded fashion (1 receiving active drug and 1 receiving placebo) and monitored for at least 1 day before the remaining 6 subjects in the cohort receive the drug. The timing of dosing initiation for the remaining 6 subjects will depend on the results of the investigator's initial safety review (indicating that the study treatment is safe and well-tolerated in the sentinel subjects).

Dose escalation will occur only after real-time PK, safety and tolerability data (including reported AE, physical examination, vital signs, ECG and clinical laboratory findings within 48 hours of dosing) have been evaluated in at least 6 subjects from the previous dose cohort and the study treatment is deemed safe and well tolerated by the SRC. As new safety and/or PK data become available, and following SRC review of the data, changes may beTable 5.1 -Table 5.11 The anticipated dose escalation scheme outlined in Table 5.1-1. Preliminary PK data from subjects in this study will help guide further dose escalation.

**Part B (MAD)**

Healthy subjects were assigned to 4 BID or QD sequential dose cohorts of 8 subjects each. Subjects in each dose cohort were randomized in a 3:1 ratio (6 active, 2 placebo) to receive either INS018_055 or matching placebo for 7 days, with approximately 32 subjects enrolled in this arm.

During the conduct of Part A, PK data, safety and tolerability data (including reported AE, physical examination, clinical laboratory tests, 12-lead ECG, and vital signs) are dynamically evaluated, and if the steady-state exposures of the MAD cohort are assessed to be within the exposure range of the dose groups for which the SAD has been completed, and the SRC deems the study treatment safe and well tolerated, Part B may be conducted in parallel. The timing and dosing regimen (QD or BID) for Part B will be determined by the SRC.

The four cohorts in Part B are 30 mg BID, 60 mg BID, 90 mg BID, and 120 mg QD. of these, 90 mg BID and 120 mg QD are used in an sentinel cohort to reduce the risk of unintended AE not predicted by preclinical pharmacology and toxicology studies in each cohort. The sentinel subjects will receive dosing in a blinded fashion (1 receiving active drug and 1 receiving placebo) and will be monitored for at least 1 day before the remaining 6 subjects in the cohort receive dosing. The timing of dosing initiation for the remaining 6 subjects will depend on the results of the investigator's initial safety review (indicating that the study treatment is safe and well-tolerated in the sentinel subjects).

Subsequent dose escalations in Part B will not be performed until safety (including AE, physical examination, clinical laboratory findings, vital signs, and 12- lead ECG) and tolerability data (up to and including Day 11) have been evaluated in at least 6 subjects from the previous MAD dose cohort and the SRC has deemed the study treatment safe.

As new safety or PK data become available, the dose escalation scheme may be changed. Instead of receiving a subsequent higher dose, subjects may receive a lower dose administration or may be administered at the same daily dose under a different dosing regimen (e.g., BID dosing instead of QD dosing) to achieve a lower maximum observed plasma concentration ($C_{max}$ ) value, especially if a safety outcome is believed to correlate with the $C_{max}$ value.

Each subject in Part B will be admitted to the study site on Day -1 and will begin receiving once-daily dosing on Day 1. The study drug will be administered at approximately the same time each morning. For the BID dosing (30 mg, 60 mg and 90 mg dose groups), the study treatment will be given again approximately 12 hours after the study treatment is given in the morning.

Cohorts 4 through 7 Subjects will fast (not eat or drink anything except water) overnight for at least 10 hours prior to dosing on the morning of Days 1 and 7, and for an additional 4 hours after study treatment administration. Drinking is not permitted 1 hour before and 1 hour after dosing. Water was allowed at other times. When the study treatment drug is administered under the BID regimen, water is not restricted for evening dosing. On Days 1 and 7, lunch will be provided approximately 4 hours after morning dosing, dinner will be provided approximately 10 hours after morning dosing, and a snack will be provided approximately 13 hours after morning dosing.

On days 2 through 6, for cohorts 4 through 7, breakfast will be served approximately 60 minutes after study treatment administration. Subjects will be provided with a standard meal throughout the study period. Lunch will be provided approximately 4 hours after morning dosing, dinner will be provided approximately 10 hours after morning dosing, and a snack will be provided approximately 13 hours after morning dosing.

Physical examination, vital sign measurements, 12-lead ECG and clinical laboratory evaluation (including liver function test results) will be performed at selected time points throughout the dosing interval. Subjects will be closely monitored for AE throughout the study period and blood samples for PK analysis will be collected within 72 hours of study drug administration on Day 7. For biomarker analysis, blood samples will be collected at selected time points for the QD and BID dosing cohorts (see Table 3.1-2 for details). Fecal occult blood testing will be performed on all stool samples at screening, registration (Day 1) and throughout the Part B study site observation period (i.e., up to Day 11).

Subjects in each cohort will remain at the study site from Day -1 until Day 11 when the investigator (or designee) will complete the scheduled procedure and review the safety data before leaving. Subjects will return to the study site on Day 28 (± 3 days) for an EOS visit. The duration of the study (excluding the screening period) is approximately 32 days.

## 3.1 SCHEDULE OF EVENTS

The study assessments and procedures for the screening and treatment periods are shown in Table 3.1 -Table 3.11 (Part A) and Table 3.1 -Table 3.12 (Section B).

**Table 3.1 -Table 3.11 Schedule of Research Activities for Part A (SAD)**

| Staging | Screening Period | Registration | Treatment period | | | | EOS |
|---|---|---|---|---|---|---|---|
| **Procedures[(a)]** / **Research Day** | **-28 to -2** | **-1** | **1** | **2** | **3** | **4** | **8 (± 1)** |
| Admission to Clinical Center | | X | | | | | |
| Leaving the Clinical Center[(b)] | | | | | | X | |
| Center Visits[(c)] | | | | | | | X |
| Informed Consent | X | | | | | | |
| Demographic information (gender, age, ethnicity) | X | | | | | | |
| Serology[(d)] | X | | | | | | |
| Covid-19 screening test[(e)] | X | X | | | | | |
| Serum FSH[(f)] | X | | | | | | |
| Inclusion/exclusion criteria | X | X | | | | | |
| Medical history | X | X | | | | | |
| Height, weight and BMI[(g)] | X | X | | | | | X |
| Chest X-ray | X | | | | | | |
| Physical Examination[(h)] | X | X | | X | X | | X |
| Vital Signs Measurement[(i)] | X | X | X | X | X | X | X |
| 12 Lead ECG[(j)] | X | X | X | X | X | X | X |
| Clinical Laboratory Tests[(k)] | X | X | | X | | X | |
| Urine drug/alcohol/cotinine screening[(l)] | | X | | | | | |
| Pregnancy test[(m)] | X | X | | | | | X |
| Investigational drug delivery[(n)] | | | X | | | | |
| PK blood sample collection[(o)] | | | X | X | X | X | |
| Biomarker sample collection[(p)] | | X | | X | | X | |
| Stool occult blood test[(q)] | X | X | X | X | X | X | |
| Fasting period[(r)] | | X | X | | X | | |
| Non-fasting period[(r)] | | | X | X | X | X | |
| AE[(s)] | | | ← | | X | | → |
| Pre-existing/concomitant medications | ← | | X | | | | → |

Abbreviations: AE, adverse event; BMI, body mass index; Covid-19, 2019 coronavirus disease; ECG, electrocardiogram; EOS, end of study; FSH, follicle stimulating hormone; PK, pharmacokinetics; SAD, single-dose escalating dose administration; ULN, upper limit of normal.
Notes:

(a) When procedures overlap or occur at the same time point, all blood draws should be performed after vital signs or ECG measurements, and PK sampling should be scheduled last and as close to the planned time window as possible.
(b) Leave the study site after collecting the final PK sample.
(c) You will return to the center for an EOS visit 4 days after leaving the research center.
(d) Serologic testing will include hepatitis B surface antigen, hepatitis C virus antibodies and human immunodeficiency virus type 1 and 2 antibodies. Tests will be performed at screening. A complete list of serologic evaluations is provided in Section 6.2.2 Section 6.2.2.
(e) The COVID-19 nucleic acid test report provided by a formal testing facility and valid for the duration of the study was confirmed by the investigator at the time of screening or during the study, according to the local government's outbreak prevention and control policy and hospital prevention and control requirements.
(f) Postmenopausal women only, to confirm postmenopausal status. For more detailed information see page 6.2.2 Section 6.2.2.
(g) Height and weight were measured and BMI was calculated at screening only, and only weight was measured at registration and EOS.
(h) A full physical examination will be conducted at screening. A brief physical examination will be performed at registration and EOS. A physical examination will also be performed approximately 24 and 48 hours after dosing. More detailed information is provided in Section 6.2.5 Section 6.2.5.
(i) Vital signs (respiratory rate, temperature, pulse and blood pressure) will be recorded at screening, registration (Day -1). Day 1: before (within 45 minutes before dosing) and 1 (± 30 minutes), 2 (± 30 minutes), 4 (± 30 minutes), 8 (± 30 minutes) and 12 (± 30 minutes) hours after dosing. In addition: Days 2, 3, 4 and 8. For more detailed information on vital sign measurements, see Section 6.2.3 Section 6.2.3.
(j) At screening and registration (Day -1), three standard 12-lead ECG results will be recorded at with repeated measurements no more than 5 minutes apart and results will be averaged; single ECG collections will be performed at all other prescribed visits. Day 1: Pre-dose (within 1 h before dosing) and 1 (± 15 min), 2 (± 15 min), 4 (± 15 min) and 8 (± 15 min) hours after dosing. In addition: additional ECG recordings will be made on days 2, 3, 4 and 8 (EOS). More detailed information on ECG recordings can be found in Section 6.2.4. 6.2.4 Section 6.2.4.
(k) Samples for clinical laboratory tests (routine blood, blood biochemistry, coagulation and urine) will be collected at screening, registration (Day -1), Day 2 and Day 4 (the day of departure from the study site); if laboratory tests were performed on Days -7 to -2 of the screening period, they may not be repeated on Day -1. Laboratory samples will be collected after an overnight fast of at least 10 hours. *If serum aminotransferases are elevated to > 3 × ULN, repeat raw blood biochemical laboratory tests within 48 to 72 hours to confirm the abnormality and to determine whether it is elevated or decreased. If drug-induced liver injury is suspected, close monitoring should be initiated, which may include repeat liver enzyme and serum bilirubin testing 2 or 3 times per week (at the discretion of the investigator). More detailed information on clinical laboratory assessments (including a complete list of assessments) can be found in Section 6.2.2 Section 6.2.2.
(l) Urine drug/alcohol/cotinine screening will be performed at the time of registration. For more detailed information see page 6.2.2 Section 6.2.2.
(m) All women will have a serum pregnancy test at screening and EOS, and a urine pregnancy test at registration.
(n) In each cohort (30 mg for cohort 1, 60 mg for cohort 2, and 120 mg for cohort 3), INS018_055 dosing times are referred to as "0" hours and occur on the dates shaded in gray. More detailed information on dosing is provided in Section 5.1 Section 5.1.
(o) Blood samples for PK analysis will be collected on Day 1 before dosing (within 45 minutes prior to dosing) and 0.25 (± 5 minutes), 0.5 (± 5 minutes), 1 (± 5 minutes), 2 (± 5 minutes), 4 (± 5 minutes), 6 (± 10 minutes), 8 (± 10 minutes), 10 (± 10 minutes), and 12 hours (± 10 minutes) after INS018_055 dosing; Day 3 at 48 hours (± 60 minutes); Day 4 at 24 (± 30 minutes) and 36 hours (± 30 minutes) after dosing; Day 3 at 48 hours (± 60 minutes); and Day 4 at 24 (± 30 minutes) and 36 hours (± 30 minutes) after dosing. 10 minutes); 24 (± 30 minutes) and 36 hours (± 30 minutes) on day 2; 48 hours (± 60 minutes) on day 3; and 72 hours (± 60 minutes) on day 4. PK blood sampling time points can be modified based on new data obtained for each cohort.

More detailed information on blood sample collection for PK analysis is provided in Section 6.1 Section 6.1.

(p) Blood samples for biomarker (T cell CD4+ and CD8+ subpopulations) analysis will be collected at registration (Day -1), Day 2 (i.e., 24 hours post-dose, time window ± 30 minutes) and Day 4 (i.e., 72 hours post-dose, time window ± 60 minutes) after INS018_055 administration. More detailed information on blood sample collection for biomarker analysis is provided in Section 6.3 Section 6.3.

(q) One stool sample will be collected for testing each day during screening, registration (Day -1) and Days 1 through 4. Any subject with a positive fecal occult blood on Day 4 may remain at the study site for a longer period of observation at the discretion of the investigator.

(r) More detailed information on fasting and non-fasting periods can be found on page 5.1 Section 5.1.

(s) More detailed information on AE collection and reporting is provided in Section 6.2.1 Section 6.2.1.

**Table 3.1 -Table 3.12 Schedule of Research Activities for Part B (MAD)**

| Staging | Screening Period | Registration | Treatment period | | | | | | | | | | | EOS |
|---|---|---|---|---|---|---|---|---|---|---|---|---|---|---|
| **Procedures[a] Research Day** | **-28 to -2** | **-1** | **1** | **2** | **3** | **4** | **5** | **6** | **7** | **8** | **9** | **10** | **11** | **Day 28 (± 3 days)** |
| Admission to Clinical Center | | X | | | | | | | | | | | | |
| Leaving the Clinical Center[b] | | | | | | | | | | | | | X | |
| Center Visits[c] | | | | | | | | | | | | | | X |
| Informed Consent | X | | | | | | | | | | | | | |
| Demographic information (gender, age, ethnicity) | X | | | | | | | | | | | | | |
| Serology[d] | X | | | | | | | | | | | | | |
| Covid-19 screening test[e] | X | X | | | | | | | | | | | | |
| Serum FSH[f] | X | | | | | | | | | | | | | |
| Inclusion/exclusion criteria | X | X | | | | | | | | | | | | |
| Medical history | X | X | | | | | | | | | | | | |
| Height, weight and BMI[g] | X | X | | | | | | | | | | | | X |
| Chest X-ray | X | | | | | | | | | | | | | |
| Physical Examination[h] | X | X | | X | | | X | | X | | | | X | X |
| Vital Signs Measurement[i] | X | X | X | X | X | X | X | X | X | X | X | X | X | X |
| 12 Lead ECG[j] | X | X | X | X | | X | | X | X | X | X | | X | |
| Clinical Laboratory Tests[k] | X | X | | X | | X | | | X | X | | | X | |
| Urine drug/alcohol/cotinine screening[l] | | X | | | | | | | | | | | | |
| Pregnancy test[m] | X | X | | | | | | | | | | | | X |
| Investigational drug delivery[n] | | | X | X | X | X | X | X | X | | | | | |

| | | | | | | | | | | | | | | |
|---|---|---|---|---|---|---|---|---|---|---|---|---|---|---|
| PK blood sample collection[o] | | | X | X | X | X | X | X | X | X | X | X | | |
| Biomarker sample collection[p] | | X | | | | | X | | X | | | | X | |
| Stool occult blood test[p] | X | X | X | X | X | X | X | X | X | X | X | X | X | |
| Fasting period[r] | | X | X | | X | | | X | X | | | | | |
| Non-fasting period[r] | | | X | X | X | X | X | X | X | X | X | X | X | X |
| AE[s] | | | ← X → | | | | | | | | | | | |
| Pre-existing/concomitant medications | ← X → | | | | | | | | | | | | | |

Abbreviations: AE, adverse event; BID, twice daily; BMI, body mass index; Covid-19, 2019 coronavirus disease; ECG, electrocardiogram; EOS, end of study; FSH, follicle stimulating hormone; MAD, multiple incremental dose; PK, pharmacokinetics; QD, once daily; ULN, upper limit of normal.

Notes:

(a) When procedures overlap or occur at the same time point, all blood draws should be performed after vital signs or ECG measurements, and PK sampling should be scheduled last and as close to the planned time window as possible.

(b) Subjects in the QD and BID cohorts will leave the study site on Day 11 after biomarker and fecal occult blood samples are collected.

(c) Return to the center for an EOS visit 16 to 17 days after leaving the study site.

(d) Serologic testing will include hepatitis B surface antigen, hepatitis C virus antibodies and human immunodeficiency virus type 1 and 2 antibodies. Tests will be performed at screening. A complete list of serologic evaluations is provided in Section 6.2.2 Section 6.2.2.

(e) The COVID-19 nucleic acid test report provided by a formal testing facility and valid for the duration of the study was confirmed by the investigator at the time of screening or during the study, according to the local government's outbreak prevention and control policy and hospital prevention and control requirements.

(f) Postmenopausal women only, to confirm postmenopausal status. For more detailed information see page 6.2.2 Section 6.2.2.

(g) Height and weight were measured and BMI was calculated at screening only, and only weight was measured at registration and EOS.

(h) A full physical examination will be conducted at screening. A brief physical examination will be conducted at registration and EOS. Physical examinations will also be conducted on days 2, 5, 7 and 11. More detailed information can be found in section 6.2.5 Section 6.2.5.

(i) Vital signs (respiratory rate, temperature, pulse and blood pressure) will be recorded at screening, registration (day -1), days 1 to 11 and day 28. Days 1 and 7: before dosing (within 45 minutes before dosing) and 1 (± 30 minutes), 2 (± 30 minutes), 4 (± 30 minutes), 8 (± 30 minutes), 12 (± 30 minutes) and 24 (± 60 minutes) hours after dosing. Vital sign measurements will be obtained in the morning before dosing on days 2 through 6. Vital sign measurements on days 9, 10, 11 and 28 will be taken in the morning. More detailed information on vital sign measurements can be found in Section 6.2.3. 6.2.3 Section 6.2.3.

(j) At screening and registration (Day -1), three standard 12-lead ECG results will be recorded with repeated measurements no more than 5 minutes apart and results averaged; a single ECG acquisition will be performed at all other prescribed visits. Days 1 and 7: before dosing (within 1 h before dosing) and 1 (± 15 min), 2 (± 15 min), 4 (± 15 min), 8 (± 15 min) and 12 (± 15 min) hours after dosing. In addition, ECGs will be obtained on the morning of days 2, 4, and 6 before dosing; on the morning of days 8 and 9 (12 and 36 hours after dosing on the evening of day 7); and before leaving the study site on day 11. More detailed information on ECG recordings is provided in Section 6.2.4. 6.2.4 More detailed information on the ECG recordings can be found in Section 6.2.4.

(k) Samples for clinical laboratory tests (routine blood, blood biochemistry, coagulation, and urine) will be collected at screening, registration (Day -1), Days 2, 4, 7, 8, and on the day of departure from the study site (Day 11); if laboratory tests were performed on Days -7 through -2 of the screening period, they may not be repeated on Day -1. Laboratory samples will be collected after an overnight fast of at least 10 hours. *If serum aminotransferases are elevated to > 3 × ULN, serum chemistry laboratory tests should be repeated within 48 to 72 hours to confirm the abnormality and to determine whether it is elevated or decreased. If drug-induced liver injury is suspected, close monitoring should be initiated, which may include repeat liver enzyme and serum bilirubin testing 2 or 3 times per week (at the discretion of the investigator). More detailed information on clinical laboratory assessments (including a complete list of assessments) is provided in Section 6.2.2 Section 6.2.2.

(l) Urine drug/alcohol/cotinine screening will be performed at the time of registration. For more detailed information see page 6.2.2 Section 6.2.2.

(m) All women will have a serum pregnancy test at screening and EOS, and a urine pregnancy test at registration.

(n) In each cohort (30 mg BID for cohort 4, 60 mg BID for cohort 5, 90 mg BID for cohort 6, 120 mg QD for cohort 7), the time of administration of INS018_055 is referred to as "0" hours (except for the BID cohort, where the second dose will be administered 12 hours after the initial dose) The dosing occurs on the date shaded in gray. More detailed information on dosing is provided in Section 5.1 Section 5.1.

(o) For QD administration, blood samples for PK analysis will be collected at the following time points: Day 1: before (0 hours) (within 45 minutes prior to dosing) and 0.25 (± 5 minutes), 0.5 (± 5 minutes), 1 (± 5 minutes), 2 (± 5 minutes), 4 (± 5 minutes), 6 (± 10 minutes), 8 (± 10 minutes), 10 (± 10 minutes) and 12 hours after dosing; Day 2, 3, 4, 5 and 6: before (0 hours) (within 45 minutes prior to dosing) and 0.25 (± 10 minutes) after dosing; Day 7: before (0 hours) (within 45 minutes prior to dosing) and 0.25 (± 10 minutes) after dosing (± 10 min); Days 2, 3, 4, 5 and 6: before dosing; Day 7: before dosing (0 h) (within 45 min before dosing) and 0.25 (± 5 min), 0.5 (± 5 min), 1 (± 5 min), 2 (± 5 min), 4 (± 5 min), 6 (± 10 min), 8 (± 10 min), 10 (± 10 min), 12 (± 10 min), 24 (± 10 min) and 24 (± 10 min) after dosing (± 10 min), 24 (± 30 min), 48 (± 60 min), and 72 hours (± 60 min). For BID administration, blood samples for PK analysis will be collected at the following time points: Day 1: before morning dosing (0 hours) (within 45 minutes before dosing) and 0.25 (± 5 minutes), 0.5 (± 5 minutes), 1 (± 5 minutes), 2 (± 5 minutes), 4 (± 5 minutes), 6 (± 10 minutes), 8 (± 10 minutes), 10 (± 10 minutes), and 12 hours (± 10 minutes) after morning dosing. ) and 12 hours (± 10 min) after morning dosing; Days 2, 3, 4, 5, and 6: before morning dosing; Day 7: before evening dosing (0 hour) (within 45 min prior to dosing) and at 0.25 (± 5 min), 0.5 (± 5 min), 1 (± 5 min), 2 (± 5 min), 4 (± 5 min), 6 (± 10 min), 8 (± 10 min), 10 (± 10 min), 12 (± 10 min), 24 (± 30 min), 48 (± 60 min), and 72 hours (± 60 min) after evening dosing.. More detailed information on blood sample collection for PK analysis is provided in Section 6.1 Section 6.1.

(p) For the QD and BID dosing cohorts, blood samples for T cell CD4+ and CD8+ subpopulation analysis will be collected at registration (Day -1), 6 hours after dosing on the morning of Days 5 and 7, and on the day of departure from the study site (Day 11). Blood samples for IL-6, TGF-β, MMP-2, MMP- 9 and MMP-7 assays will be collected at registration (Day -1) and 6 hours after dosing on the morning of Day 7. More detailed information on blood sample collection for biomarker analysis is provided in Section 6.3 Section 6.3.

(q) One stool sample will be collected daily for testing during screening, registration (Day -1) and throughout the Part B study site observation period (i.e., until Day 11). Any subject with a positive fecal occult blood test result on Day 11 may remain at the study site for a longer observation period at the discretion of the investigator.

(r) More detailed information on fasting and non-fasting periods can be found on page 5.1 Section 5.1.

(s) More detailed information on AE collection and reporting is provided in Section 6.2.1 Section 6.2.1.

# 4. STUDY POPULATION

Approximately 56 male and female subjects (24 subjects will be enrolled in Study Part A and an additional 32 subjects in Study Part B) will be enrolled at 1 study site in China. The sample size determination is discussed in Section 7.1 Section 7.1.

## 4.1 INCLUSION CRITERIA

Each subject must meet all of the following criteria to be enrolled in this study:

1. Subjects were male or female between 18 and 45 years of age, inclusive.
2. Subjects had a body mass index of 19 to 26 kg/m$^2$ (inclusive) and weighed ≥ 50 kg (inclusive) for men and ≥ 45 kg (inclusive) for women at the time of screening.
3. Based on medical history, clinical laboratory findings, vital sign measurements, 12-lead ECG results, physical examination, chest radiograph results, and serum virological findings at screening, the investigators concluded that the subjects were in good general health.
4. Female subjects of childbearing potential must be non-pregnant and non-lactating and must be using one of the following methods of contraception throughout the study until at least 28 days after the last dose of study drug, or have been surgically sterilized (i.e., hysterectomy, bilateral tubal ligation, or bilateral oophorectomy) or are postmenopausal (defined as 12 consecutive months of amenorrhea with documented plasma follicle stimulating hormone levels > 40 IU/mL). Female subjects must have a negative pregnancy test result at screening and prior to the first dose of study drug.

A highly effective method of contraception is one that has a contraceptive failure rate of less than 1% per year when used consistently. Examples are as follows:

a. Implanted contraceptives (e.g., Jadelle® )

b. Copper or levonorgestrel-containing intrauterine device (IUD; e.g., Mirena® )

c. Male sterilization, no sperm in ejaculation after vasectomy

d. Double barrier method: condom and and occlusion cap (diaphragm or cervical cap/dome cap), barrier method with spermicide (foam/gel/film/cream/suppository) must be used as an add-on

e. Abstinence, defined as complete and continuous avoidance of all heterosexual sex (including during the entire period of risk associated with study treatment), was allowed without contraception only if this was the subject's preferred and daily lifestyle.

or an effective method with a contraceptive failure rate of less than 5% to 10% per year. Examples are as follows:

f. Injectable contraceptives (e.g., Depo Provera)

g. Oral contraceptives (combination hormonal contraceptives or progestogen-only "mini-pills")

h. Vaginal contraceptive ring (e.g., NuvaRing® )

Female subjects must also agree not to donate eggs from the time of administration until at least 28 days after the last dose of study drug.

Male subjects and their fertile female partners must agree to use one of the above methods of contraception for the entire treatment period until at least 28 days after the last dose of study drug. Male subjects must also agree not to donate sperm for the entire treatment period until at least 28 days after the last dose of study drug.

5. Subjects agree to comply with all protocol requirements.

6. Subjects were able to provide written informed consent.

## 4.2 EXCLUSION CRITERIA

Subjects who met any of the following criteria were excluded from the study:

1. Subject has current evidence or history of clinically significant hematologic, renal, endocrine, pulmonary, GI, cardiovascular, hepatic, psychiatric, neurologic, or allergic disease (including drug allergy, but excluding asymptomatic seasonal allergy that was untreated at the time of administration).

2. Subjects have any condition that may affect drug absorption (e.g., gastrectomy).

3. Subjects had a history of cancer, except adequately treated basal cell or squamous cell carcinoma of the skin.

4. The subject rests for at least 5 minutes with a blood pressure (BP) > 140 mm Hg (systolic) or > 90 mm Hg (diastolic). At screening, if BP is > 140 mm Hg (systolic) or > 90 mm Hg (diastolic), BP should be measured 2 additional times and the average of the 3 BP values should be used to determine the subject's eligibility to participate.

5. At screening, the subject's 12-lead ECG shows a QT interval (QTc) corrected by the Bazett formula ($QTc=QT/RR^{0.5}$ ) > 450 msec, or a QRS interval > 120 msec. The average of three QTc (or QRS interval) values from three standard 12-lead ECGs (repeated at intervals of no more than 5 minutes) should be used to Determine the subject's eligibility for participation.

6. At the time of screening, the subject has any of the following abnormalities in clinical laboratory tests (if necessary, retest once for confirmation):

   a. At screening, serum creatinine (SCr) levels above the upper limit of normal (ULN) or creatinine clearance (Ccr) < 80 mL/min calculated using the Cockcroft-Gault formula (Appendix 3) and no protein in the urine.

   b. Aspartate aminotransferase (AST) or alanine aminotransferase (ALT) values > 1.5 × ULN.

   c. Fasting glucose > 110 mg/dL (6.1 mmol/L).

   d. Total bilirubin > 1.5 × ULN.

   e. Routine blood test values that are outside the normal reference range of local laboratory findings and are considered clinically significant by the investigator.

   f. Positive fecal occult blood test at screening or registration (day -1).

7. Subjects have a history of any disease that may have caused total bilirubin to be higher than ULN. Subjects whose clinical laboratory test values are not significantly outside the reference range may be enrolled in this study if the investigator does not consider the values to be clinically significant.

   Note: In subjects with a history of Gilbert's syndrome, direct bilirubin may be measured, and if direct bilirubin < ULN, the subject is eligible for this study.

8. Subjects have any history of lymphoproliferative disease (such as Epstein Barr virus-associated lymphoproliferative disease as reported by some subjects receiving immunosuppressive drugs), lymphoma, leukemia, myeloproliferative disease, multiple myeloma, or signs and symptoms suggestive of current lymphatic disease.

9. Subjects have a history of relevant drug and/or food allergies (i.e., allergy to any study drug or excipient, or any severe food allergy that could result in inability to consume the standard diet of the clinical institution).

10. Subject has a current or clinically significant infection (e.g., presence of an infection requiring hospitalization or parenteral antimicrobial therapy or presence of an opportunistic infection) or a history of chronic or recurrent infectious disease within 6 months prior to the first dose of study drug.

11. Subject has other serious acute or chronic medical or psychiatric illness (including recent (within the past year) or active suicidal ideation or behavior or abnormal experimental results (which may increase the risk associated with study participation or experimental drug administration or may interfere

with the interpretation of study results and, in the judgment of the investigator, may render the subject unsuitable for entry into this study).

12. Subjects have a history of symptomatic herpes zoster or herpes simplex, more than one episode of localized herpes zoster, or disseminated herpes zoster (single episode) present or within 12 weeks.

13. Subjects tested positive for hepatitis B surface antigen, hepatitis C virus antibody, or human immunodeficiency virus (HIV) type 1 or 2 antibody at screening.

14. Subjects were pregnant or lactating females.

15. Subjects are men of childbearing potential who are unwilling or unable to use the contraceptive methods described in this protocol throughout the study and for at least 28 days after the last dose of the experimental drug.

16. Subjects are unwilling or unable to comply with the lifestyle restrictions described in this protocol (Section 4.3.1 section).

17. Subjects were smokers or had used nicotine or nicotine-containing products (e.g., snuff, nicotine patches, nicotine chewing gum, simulated cigarettes, or inhalants) within 6 months prior to the first dose of the study drug.

18. Subjects tested positive for substance abuse or cotinine (indicating current active smoking) prior to the first dose of study drug.

19. Subjects have used any prescription or over-the-counter medication (except paracetamol [up to 2 g/day]), including herbal supplements, within 14 days prior to the first dose of study drug. Nutritional supplements are permitted provided that they are unlikely to interfere with the study results and consent has been obtained from the medical monitor and the investigator.

20. Subjects ingested grapefruit or grapefruit juice, limes or products containing limes (e.g., orange marmalade) or products containing alcohol, caffeine or xanthines within 48 hours prior to the first dose of the study drug.

21. Subjects will be vaccinated with live virus, live attenuated virus, or any live viral component within 2 weeks prior to the first dose of study drug, or will receive these vaccines at any time during the study or within 8 weeks of study completion.

22. Subject tested positive for Severe Acute Respiratory Syndrome-associated Coronavirus 2 (SARS-CoV-2). Subject received the 2019 coronavirus disease (COVID-19) vaccine within 2 weeks prior to the first dose of study drug or is scheduled to receive the COVID-19 vaccine within 12 weeks of study drug administration, or tested positive for SARS-CoV-2 during screening or had COVID-19 symptoms within 4 weeks prior to Day -1.

23. Subjects who have undergone major trauma or major surgery within 4 weeks prior to screening or who are expected to require major surgery during the trial.

24. Subjects are at risk for bleeding: genetic predisposition to bleeding, a bleeding event within 12 months prior to screening start, or abnormal laboratory coagulation parameters.

25. Subjects have a first-degree relative with a genetic immunodeficiency.

26. Subjects were study site staff and their family members who were directly involved in the implementation of the study, study site staff who were otherwise supervised by the investigator, or sponsor employees (including their family members) who were directly involved in the implementation of the study.

27. Subject has a history of alcohol abuse or drug addiction or excessive alcohol consumption (regular alcohol intake > 21 units/week for male subjects and > 14 units/week for female subjects; 1 unit equals approximately ½ pint [200 mL] of beer, 1 small glass [100 mL] of wine, or 1 unit of measurement [25 mL] of spirits) within the past year or consumed alcohol 24 hours prior to the first dose of study drug.

28. Subjects engaged in strenuous activity or contact sports within 24 hours prior to dosing and during the study.

29. The subject donated > 450 mL of blood or blood products within 30 days prior to the first dose of the study drug.

30. Subjects received study drug in another pilot study within 30 days prior to dosing or 5 drug half-lives, whichever is longer.

31. Subjects received cytochrome P450 (CYP3A4 and CYP2C8) and P-gp inhibitors and/or inducers within 4 weeks prior to the first dose of INS018_055 or may have received CYP3A4 and CYP2C8 and P-gp inhibitors and/or inducers during the study.

32. Subjects were deemed by the investigator to be unsuitable for study entry.

## 4.3 OTHER SCREENING CONSIDERATIONS

### 4.3.1 Lifestyle restrictions

#### 4.3.1.1 Meals and dietary requirements

1. Subjects should not ingest products containing grapefruit for 48 hours prior to the first dose of study drug until they leave the study site.

2. In Part A, subjects are required to fast (not eat or drink anything other than water) for at least 10 hours prior to each dose throughout the study period

and to maintain a fast until 4 hours after the study treatment is administered on Day 1. Subjects should not consume water 1 hour before and 1 hour after study treatment administration, except when administering the drug. Water will be available at all other times. A standard lunch will be provided 4 hours after the morning dose, a standard dinner will be provided 8 hours after the morning dose, and a snack will be provided approximately 13 hours after the morning dose. For Part A, all meals on Day 1 must be the same.

3. In Part B, subjects will fast overnight (no food or drink except water) for at least 10 hours before receiving the morning dose (BID or QD) on Days 1 and 7, and for an additional 4 hours after study treatment administration. Drinking is not permitted 1 hour before and 1 hour after dosing. Water was allowed at other times. When the study treatment is administered by the BID regimen, water is not restricted for evening dosing. On Days 1 and 7, lunch will be provided approximately 4 hours after the morning dose, dinner will be provided approximately 10 hours after the morning dose, and a snack will be provided approximately 13 hours after the morning dose. The composition of the meals provided on Days 1 and 7 must be the same.

4. For all Part B cohorts, breakfast will be provided approximately 60 minutes after study treatment administration on Days 2 through 6. Lunch will be provided approximately 4 hours after morning dosing, dinner will be provided approximately 10 hours after morning dosing, and a snack will be provided approximately 13 hours after morning dosing. A standard meal will be provided to subjects on days 2 through 6.

#### 4.3.1.2 Caffeine and alcohol

1. Subjects should not consume products containing caffeine or xanthines (e.g., coffee, tea, cola drinks, and chocolate) within 48 hours prior to dosing and during the study follow-up period.

2. Subjects will not be allowed to consume alcohol 24 hours prior to study drug administration, during the study site stay until they leave the study site, and 48 hours prior to the outpatient visit.

#### 4.3.1.3 Events

1. Subjects should avoid strenuous exercise for 48 hours prior to study site admission and throughout the study period. During the study period, subjects may participate in light recreational activities (e.g., watching television, reading).

2. Subjects were asked to remain at the clinical facility from Day -1 to the end of their stay.

3. Subjects are advised not to donate blood or plasma for at least 3 months after the last dose.

4. On Day 1 (Part A) and after administration on Days 1 and 7 (Part B), subjects should remain in a semi-recumbent position for at least 4 hours, except as required by study procedures; when the study treatment is administered using the BID regimen, subjects should remain in an upright (sitting or standing) position for at least 1 hour after evening dosing. On Days 2 through 6 (Part B), subjects should remain in an upright (sitting or standing) position for at least 1 hour after dosing. The subject must be continuously supervised while in an upright position.

#### 4.3.1.4 Contraceptive

If the subject is all female subjects of childbearing potential who are considered by the investigator to be sexually active and at risk for pregnancy and male subjects whose partners and themselves are of childbearing potential, they must agree to use a contraceptive method consistently and correctly for the duration of the active drug treatment and for at least 28 days after the last dose of the study drug. The investigator or his/her designee, in consultation with the subject, will confirm that the subject has selected the contraceptive method most appropriate for the individual subject and his/her female partner from the list of permitted contraceptive methods (see below) and that the subject has been instructed in the consistent and correct use of the contraceptive method. At the time of signing the informed consent form, the investigator or designee will inform the subject of the need for consistent and correct use of contraception and will document the conversation and the subject's confirmation in the subject's medical record. In addition, the investigator or designee will instruct the subject to call the appropriate person immediately if the subject or partner discontinues the contraceptive method of choice or if the subject's partner is known or suspected to be pregnant.

Highly effective contraceptive methods are those that have a contraceptive failure rate of less than 1% per year when used consistently, as follows

1. Implant contraceptives (e.g., Jadelle)

2. IUDs containing copper or levonorgestrel (e.g., Mirena)

3. Male sterilization, no sperm in ejaculation after vasectomy

4. Female sterilization (e.g., bilateral tubal ligation ["clamping or stitching"] or hysterectomy)

5. Double barrier method: condom and and occlusion cap (diaphragm or cervical cap/dome cap), barrier method with spermicide (foam/gel/film/cream/suppository) must be used as an add-on

6. Abstinence, defined as complete and continuous avoidance of all heterosexual sex (including during the entire period of risk associated with study treatment), was allowed without contraception only if this was the subject's preferred and daily lifestyle.

or an effective method with a contraceptive failure rate of less than 5% to 10% per year, as follows

7. Injectable contraceptives (e.g., Depo Provera)
8. Oral contraceptives (combination hormonal contraceptives or progestogen-only "mini-pills")
9. Vaginal contraceptive ring (e.g. NuvaRing)

In addition, female subjects must agree not to donate eggs and male subjects must agree not to donate sperm during active drug treatment and for at least 28 days after the last dose of study drug.

If a female subject becomes pregnant or a female partner of a male subject becomes pregnant, this must be reported to the investigator as soon as possible. Consent will be requested from the female subject or female partner for the collection of information about her and her infant for monitoring purposes.

## 4.4 SUBJECTS WITHDREW FROM THE STUDY

### 4.4.1 Reason for withdrawal

Subjects may withdraw informed consent and discontinue participation in the study at any time for any reason, which will not affect further treatment.

The investigator may withdraw a subject from the study if the subject

1. Poor Protocol adherence
2. Serious AE (SAE) or intolerable AE that the investigator deems necessary to withdraw from the study
3. Laboratory safety assessment shows clinically significant changes in hematology or biochemistry relative to baseline values
4. Development of symptoms or diseases listed in the exclusion criteria during the course of the study
5. The use of a drug prohibited by the Protocol is required (Section 4.2). 4.2 Section 4.2)
6. Request early termination of the study for any reason.

In addition, the investigator may withdraw subjects from the study at the request of the sponsor, or if the sponsor terminates the study, the investigator may withdraw subjects from the study. In the event of an SAE or intolerable AE, the investigator will consult with the sponsor. If a subject is discontinued due to an AE, the event will be followed until it subsides, stabilizes, or is determined by the investigator to be not clinically significant .

### 4.4.2 Handling of withdrawals

Subjects may be withdrawn from the study at any time upon request. Subjects may be discontinued from the study at any time at the discretion of the investigator or at the request of the sponsor.

When a subject withdraws from the study, the investigator should document the reason for withdrawal on the appropriate page of the electronic case report form (eCRF). If possible, any subject who withdraws early from the study will undergo all EOS evaluations. The study site will contact any subjects who do not return to the center for a final evaluation to attempt to bring them into compliance with the protocol. The status of subjects who do not complete the final assessment will be recorded in the eCRF.

If a subject withdraws from the study and revokes consent to disclose future information, no further evaluation should be conducted and no additional data should be collected. The sponsor may retain and continue to use any data collected prior to the withdrawal of consent.

**Lost to follow up**

If a subject does not return to the center for a scheduled visit, every effort should be made to contact the subject. The investigator or research center staff should attempt to contact the subject and if no response is received from the subject, the subject will be considered a missed visit. All attempts to contact the subject and information received during the attempted contact must be documented in the subject's medical record. In all cases, every effort should be made to document the subject's outcome, if possible. The investigator should ask the reason for withdrawal, request that the subject return to the center for a final visit (if applicable), and follow up with that subject regarding any unresolved AE.

All reasonable efforts must be made to locate subjects to determine and report their current status. This includes follow-up visits with persons authorized by the subject as described above. All attempts to contact the subject should be documented in that subject's medical record. If it is determined that the subject has died, the research center will use locally permitted methods to obtain the date and cause of death. If the section regarding the investigator's assistance with study follow-up through a third-party representative is included in that subject's informed consent, the investigator may assist the research center staff through a third-party representative retained by the sponsor to obtain the subject's contact information or other publicly available vital status data needed to complete the study follow-up section. Research Center staff and representatives will consult publicly available sources, such as public health registry information and databases, to obtain updated contact information. If, after all attempts to contact the subject have been exhausted, the subject remains out of follow-up status, the last known date of survival as determined by the investigator should be reported and documented in the subject's medical record.

### 4.4.3 Replacement

At the discretion of the investigator and after consultation with the medical monitor, any subject who withdraws prior to the first dose may be replaced to retain the target number of 56 evaluable subjects. Any replacement subject will receive treatment after assignment that is identical to that of the subject he or she is replacing.

# 5. RESEARCH TREATMENT

An investigational treatment is defined as any investigational treatment, marketed product, or placebo to be given to a study subject according to the study randomization grouping or treatment assignment plan.

- INS018_055 capsule doses of 30, 60 and 120 mg in Part A
- Matching placebo capsules
- INS018_055 capsule doses of 30 mg BID, 60 mg BID, 90 mg BID and 120 mg QD in Part B

Investigational treatments include both experimental drugs (IP/IMP) and non-IP/non-IMP, examples of which are listed below:

- All products investigated in clinical studies or used as control drugs (active drug or placebo)
- Pre-operative medication for study requirements

IP (also referred to as IMP in some regions) is defined as an active pharmaceutical formulation or placebo that is investigated in clinical studies or used as a control drug, including products that have received marketing approval but are used or assembled (formulated or packaged) in a manner different from the approved product, or for an unapproved indication, or for obtaining additional information on an approved product.

Subjects will be provided with INS018_055 capsules or matching placebo for oral administration. Storage and dosing instructions independent of the study protocol will be provided to the study site.

Other drugs used as supportive or rescue medications for prophylactic, diagnostic or therapeutic reasons, or as part of standard treatment for a given diagnosis, may be considered non-IP.

## 5.1 TREATMENT GIVEN

The chosen mode and timing of administration depends on the randomization grouping and includes the following IPs:

**Table 5.1 -Table 5.11 Choice of drug delivery method and timing of drug administration**

| Research Treatment | Unit dose specification/dose level | Dosing frequency | Route of administration |
|---|---|---|---|
| **Part A** | | | |
| INS018_055 | 30, 60 and 120 mg | Single dose on day 1 | Oral |
| INS018_055 Matching placebo | N/A | Single dose on day 1 | Oral |
| **Part B** | | | |
| INS018_055 | BID: 30, 60, 90 mg<br>QD: 120 mg | Day 1 to Day 7 BID<br>Day 1 to Day 7 QD | Oral |
| INS018_055 Matching placebo | N/A | Day 1 to Day 7 QD/BID | Oral |

Abbreviations: BID: twice daily; MAD: multiple dose escalation dosing; N/A: not applicable; PK: pharmacokinetic; QD: once daily; SAD: single dose escalation dosing.

During Part A, each subject will receive the assigned dose of study treatment on the morning of Day 1 according to the randomization schedule. During Part B, each subject will receive the assigned dose of study treatment on Day 1 through the morning of Day 7 according to the randomization schedule. For BID dosing, the evening dose will be administered approximately 12 hours after the morning dose. For QD administration, study drug treatment will be given at approximately the same time each morning.

Page 3 Section 3 outlines the treatment to be administered in each section. At the time of administration, the subject will receive the study drug in approximately 240 mL of room temperature water. The time of administration is referred to as the "0" time point.

Restrictions related to food and fluid intake are described in Section 4.3.1 Section 4.3.1.

## 5.1.1 Dose Escalation

### 5.1.1.1 Part A (SAD)

The safety review committee (SRC) is initiated by the sponsor and should include the sponsor, the investigator, the contract research organization (medical monitor, etc.), and other relevant personnel. the SRC conducts a blinded review of safety data for each subject and the entire cohort and makes a recommendation on whether to escalate to the next dose level. Two subjects from each dosing cohort will be selected to form an sentinel cohort to reduce the risk of unintended AEs in each cohort that were not predicted by preclinical pharmacology and toxicology studies. The sentinel subjects will receive dosing in a blinded fashion (1 active drug and 1 placebo) and will be monitored for at least 1 day prior to dosing for the remaining 6 subjects in the cohort. The timing of dosing initiation for the remaining 6 subjects will depend on the results of the investigator's initial safety review (indicating that the study treatment is safe and well tolerated for administration in the sentinel subjects).

Dose escalation will only occur after real-time PK, safety and tolerability data (including reported AE, physical examination, vital signs, 12-lead ECG and clinical laboratory results within 48 hours of dosing) have been evaluated for at least 6 subjects in the previous dose cohort and the study treatment is deemed safe and well tolerated by the SRC.

As new security and/or PK data become available, following SRC review of the data, this may changeTable 5.1 -Table 5.11 The anticipated dose escalation scheme outlined in Table 5.1-1. Preliminary PK data from subjects in this study will help guide further dose escalation.

Details of the SRC membership will be provided in the Security Committee Charter. The recommendations of the committee will also be discussed with the investigator. Recommendations to discontinue dosing will be binding.

5.1.1.2 Part B (MAD)

After evaluation of PK, safety, and tolerability data from Part A (including reported AE, physical examination, clinical laboratory findings, 12-lead ECG, and vital signs), if the steady-state exposures for the MAD cohort are assessed to be within the exposure range of the dose groups for which the SAD has been completed, and after the SRC deems the study treatment safe and well tolerated, Part B may be conducted in parallel, and the Part B The timing and dosing regimen (QD or BID) will be determined by the SRC.

For the 90 mg BID and 120 mg QD cohorts in Part B, 2 subjects each will be selected to form an sentinel cohort to reduce the risk of unintended AEs not predicted by preclinical pharmacology and toxicology studies in each cohort. The sentinel subjects will receive dosing in a blinded fashion (1 active drug and 1 placebo) and will be monitored for at least 1 day prior to dosing for the remaining 6 subjects in the cohort. The timing of dosing initiation for the remaining 6 subjects will depend on the results of the investigator's initial safety review (indicating that the study treatment is safe and well tolerated for administration in the sentinel subjects).

Subsequent dose escalations in Part B will not be performed until safety and tolerability data up to and including Day 11 have been evaluated for at least 6 subjects in the previous MAD dose cohort and the SRC has deemed the study treatment safe.

As new safety or PK data become available, the dose escalation scheme may change. Instead of receiving a subsequent higher dose, subjects may receive a lower dose administration or may be given the same daily dose under a different dosing regimen (e.g., BID dosing instead of QD dosing) to achieve a lower $C_{max}$ value, especially if the safety results are believed to correlate with the $C_{max}$ value. The Institutional Review Board (IRB) will be notified and provided with the basis for any changes prior to implementation.

### 5.1.2 Dose escalation stopping criteria

Dosing within the dose cohort will be stopped and dose escalation suspended until safety information can be reviewed if any of the following criteria are met and the following are confirmed by appropriate retesting (repeat blood draws must be performed within 24 hours):

- SAEs associated with investigational drugs that the SRC believes suggest safety and tolerability limits have been reached and prevent further safe administration to subjects
- Two or more subjects in the same dose cohort develop identical (and likely related) study drug-related AE of severe severity while on study treatment
- Acute clinically significant bleeding in two or more subjects within the same dose cohort who experience a clinically relevant non-major bleeding event during study treatment, defined as an acute clinically significant bleed that does not meet the additional criteria required to define a bleeding event as a major bleeding event and meets at least one of the following criteria: admission to the hospital for bleeding or physician-directed drug or surgical treatment for bleeding
- One subject experienced major bleeding during study treatment, defined as intracranial hemorrhage, hemoglobin drop ≥ 5 g/dL, erythropoietic cell pressure drop ≥ 30%, or need for blood transfusion
- 1 subject with serum creatinine > 2 × ULN, confirmed by retesting
- QTc > 500 msec in two or more subjects within a dose cohort, confirmed by repeat ECG examination
- AST and/or ALT > 5 × ULN in two subjects, confirmed by retest
- One subject with AST and/or ALT > 3 × ULN and total bilirubin > 2 × ULN, confirmed by retesting
- Total bilirubin > 3 × ULN in two or more subjects in the same dose cohort, confirmed by retesting
- Two subjects in the same dose cohort with ALT or AST > 3 × ULN (confirmed by retest) with signs or symptoms consistent with liver injury in the opinion of the investigator

Insilico Medicine Ltd. reserves the right not to provide investigational therapeutics in the event that development of INS018_055 is terminated for other reasons, including, but not limited to, incompatibility with other key investigational purposes.

The sponsor and investigator may decide to discontinue dosing or dose escalation within a cohort for reasons not defined above, including, but not limited to, the

observation of a single SAE in individual subjects and/or the observation of a trend of variation within a given dose cohort and/or across dose cohorts.

If any of the above criteria are met within an administered dose, incremental increases to the next higher dose will be suspended and all relevant safety data obtained throughout the study period will be evaluated to estimate the risk of continuing to give the higher administered dose. The review process may require unblinding of subjects who develop AEs as described above. The unblinded data set may include subjects in one dose cohort or, if applicable, all subjects treated to date. Recommendations for unblinding must be approved by the sponsor's medical reviewer. At the conclusion of this in-depth safety review, one of the following recommendations will be made:

- Continue research as planned
- Repeat administration of current dose in additional subjects to continue the study
- Continue the study at the dose between the current dose and the next planned dose or at the dose between the current dose and the previous lower dose
- Continue the study after implementing necessary dosing regimen adjustments based on safety, tolerability or PK analysis
- Termination of the study

The maximum acceptable dose will be lower than the dose administered to meet the discontinuation criteria unless it is decided that a lower dose should be designated as the maximum acceptable dose.

## 5.2 EXPERIMENTAL DRUGS

The study drugs to be used are as follows:

| **Products** | **Formulations provided** |
|---|---|
| INS018_055 Capsule | 5, 10, 30 mg capsules |
| Matching placebo | 0 mg Capsules |

INS018_055 capsules fill INS018_055 API directly into hydroxypropylmethylcellulose capsules without any other excipients. Matching placebo has the same appearance as INS018_055 capsules, but does not contain the active compound.

For more information on study drugs, see IB.

### 5.2.1 Study drug packaging and storage

Insilico Medicine Ltd. will provide full doses of INS018_055 capsules (5 mg, 10 mg and 30 mg) and matching placebo to the investigator and clinical site. The clinical site pharmacy will provide full doses of INS018_055 capsules (5 mg, 10 mg and 30 mg)

and matching placebo to the investigator and clinical site according to the Study Activity Schedule (SOE; Section3.1 The study treatment medication will be prepared for each subject according to the Study Activity Plan (SOE; Section 3.1). The capsules will be contained in unit dose containers and labeled according to InSilicon's regulations and clinical site labeling requirements.

The 5 mg and 10 mg capsules are size 4 and the 30 mg capsules are size 3. The 5 mg capsules have standard yellow capsules and Swedish orange opaque bodies, while the 10 mg and 30 mg capsules have Swedish orange opaque capsules and bodies.

INS018_055 Capsules (5 mg, 10 mg, and 30 mg) are packaged in 45 mL high-density polyethylene (HDPE) bottles and sealed with polypropylene (PP) continuous thread child-resistant opening caps (CRC) with aluminum foil induction closure gaskets. Each vial contains 30 capsules. The study drug should be stored at controlled room temperature (15°C to 25°C).

All study drugs must be stored in secure cabinets or rooms in accordance with labeling instructions, with access to study drugs restricted to necessary center staff. The study site will maintain temperature logs as required to establish a record of compliance with storage conditions.

### 5.2.2 Study Drug Inventory

The investigator will maintain records of all study drug receipts, including the date of receipt. Records will be maintained regarding the timing and amount of study drugs dispensed to each subject and used by the subject in the study. Reasons for deviations from the expected distribution protocol will also be documented. At study completion, to meet regulatory requirements regarding drug counts, all study drugs will be reconciled and retained or destroyed in accordance with appropriate regulations.

## 5.3 METHOD OF ASSIGNING SUBJECTS TO TREATMENT GROUPS

A randomized grouping schedule was generated by an independent statistician. Enroll eligible subjects into the current dose cohort in a 3:1 ratio. Random assignment will be made by an independent statistician for each cohort individually.

Starting with cohort 1 (initial dose cohort) through cohort 3, and cohorts 6 and 7, 2 subjects in each dosing cohort are selected to form a sentinel cohort, with the pre-post subjects receiving dosing in a blinded fashion (1 receiving active drug and 1 receiving placebo) and monitored for at least 1 day before the remaining 6 subjects in that cohort receive dosing.

## 5.4 BLINDING

### 5.4.1 Blinding procedure

This study will be a double-blind study design. ins018_055 will have the same appearance as the matched placebo capsules. The pharmacist will be responsible for dispensing the study drug in a manner that maintains a blinded state at all times.

### 5.4.2 Emergency Unblinding and Formal Unblinding

The blinded state was maintained throughout the study.

If a subject becomes seriously ill or pregnant during the study period, unblinding will occur only if it is known that administration of the study drug would affect the available treatment options for that subject. In the event of a medical emergency requiring identification of the individual subject receiving the study drug, the investigator will make every effort to contact the medical monitor within 24 hours prior to code disclosure to explain the need for code disclosure. The investigator will be responsible for documenting the time, date and reason for code unblinding and the names of those involved.

Formal unblinding takes place after the statistical analysis plan, the data review report is finalized, and the database is locked. The statistical analysis unit makes the unblinding application, the sponsor approves it, the non-blinded statistician reveals the group corresponding to the random number, and the statistical analysis unit performs the statistical analysis based on the unblinded grouping.

## 5.5 TREATMENT COMPLIANCE

All administration of the study drug will be performed in the clinical facility and directly supervised by center staff and documented in the eCRF. Center staff will confirm that the subject has received the full dose of study drug.

The date and time the study drug was administered will be recorded on the appropriate page of the eCRF. If the subject does not receive study drug administration, the reason for the missed dose will be recorded.

### 5.5.1 Prior and Concomitant Medications

Prior and concomitant medication and treatment limitations are described in Section 4.1 and 4.2 Section 4.2. Prior and concomitant medications and treatments will be coded using the latest version of the WHO Drug Dictionary.

#### 5.5.1.1 Prior medications

Information on previous medications received by the subject within 30 days prior to signing the Informed Consent Form (ICF) will be recorded on the subject's electronic case report form.

#### 5.5.1.2 Concomitant Medicine

Any concomitant medication necessary for the health of the subject for the treatment of an AE may be given during the study period at the discretion of the investigator. If concomitant medications other than those specified in the protocol are used, the investigator and sponsor will make a joint decision to allow the subject to continue the study or to discontinue the study based on the timing of drug administration, pharmacology and PK, and whether the drug use would compromise the subject's safety or affect the interpretation of the data. The investigator is responsible for ensuring that detailed information about the drug is adequately documented in the eCRF.

# 6. STUDY PROCEDURES

Prior to performing any study procedures, all potential subjects will sign the ICF, as described in Section 10.2.2.3 as described in Section 10.2.2.3. Subjects will undergo the study procedures at the time points specified in the SOE (Section 3.1).

The total amount of blood collected from each subject during the study (including any additional assessments that may be required) will not exceed 500 mL.

## 6.1 PHARMACOKINETIC EVALUATION AND ENDPOINTS

**Part A:** Blood samples for INS018_055 and metabolite PK analysis will be collected at the following time points: 0.25 (± 5 min), 0.5 (± 5 min), 1 (± 5 min), 2 (± 5 min), 4 (± 5 min), 6 (± 10 min), 8 (± 10 min) and 12 hours (± 10 min) before (within 45 minutes prior to) and after INS018_055 administration on Day 1; 24 (± 30 min) and 36 hours (± 30 min) after dosing on Day 2; 48 (± 5 min), 10 (± 10 min) and 12 hours (± 10 min) after dosing on Day 3. (± 10 min) and 12 hours (± 10 min); 24 (± 30 min) and 36 hours (± 30 min) after day 2 administration; 48 hours (± 60 min) after day 3 administration; and 72 hours (± 60 min) after day 4 administration.

**Part B:** For QD administration, blood samples for PK analysis of INS018_055 and its metabolites will be collected at the following time points:

- Day 1: Pre-dose (0 hours, within 45 minutes prior to dosing) and 0.25 (± 5 minutes), 0.5 (±5 minutes), 1 (±5 minutes), 2 (±5 minutes), 4 (±5 minutes), 6 (± 10 minutes), 8 (± 10 minutes), 10 (± 10 minutes), and 12 hours (± 10 minutes) post-dose
- Days 2, 3, 4, 5, and 6: before dosing
- Day 7: Before dosing (0 hr, within 45 min before dosing) and 0.25 (±5 min), 0.5 (±5 min), 1 (±5 min), 2 (±5 min), 4 (±5 min), 6 (±10 min), 8 (±10 min), 10 (±10 min), 12 (±10 min), 24 (±30 min), 48 (±60 min), and 72 hours (± 60 minutes)

For BID administration, blood samples for PK analysis of INS018_055 and its metabolites will be collected at the following time points:

- Day 1: Before morning dosing (0 hr, within 45 min before dosing) and 0.25 (±5 min), 0.5 (±5 min), 1 (±5 min), 2 (±5 min), 4 (±5 min), 6 (±10 min), 8 (± 10 min), 10 (± 10 min), and 12 hr (± 10 min, within 45 min before second dose) after morning dosing
- Days 2, 3, 4, 5 and 6: before morning dosing
- Day 7: 0.25 (±5 min), 0.5 (±5 min), 1 (±5 min), 2 (±5 min), 4 (±5 min), 6 (±10 min), 8 (±10 min), 10 (±10 min), 12 (±10 min), 24 (±30 min), 48 (± 60 min), and 72 hours (± 60 min) after evening dosing (0 hr, within 45 min before dosing) and after evening dosing (± 60 minutes) and 72 hours (± 60 minutes)

- The following plasma PK parameters were calculated as endpoints for INS018_055 and its metabolites using the actual sampling time rather than the planned sampling time: Area under the plasma concentration versus time curve (AUC) from time 0 to the last quantifiable concentration ($AUC_{0-t}$)
- AUC from time 0 extrapolated to infinity ($AUC_{0-inf}$)
- AUC from time 0 to the time of the dosing interval (τ; $AUC_{0-\tau}$)
- Accumulation ratio (AR), calculated as $AUC_{0-\tau}$ (Day 7)/$AUC_{0-\tau}$ (Day 1)
- AR calculated as $C_{max}$ (Day 7)/$C_{max}$ (Day 1)
- $C_{max}$
- Time to reach $C_{max}$ ($T_{max}$)
- Pre-dose concentrations on Days 1 through 7 ($C_{trough}$)
- Average concentration on Day 1 and Day 7 ($C_{av}$)
- Terminal elimination rate constant ($K_{el}$)
- Terminal elimination half-life ($t_{1/2}$)
- Apparent total body clearance (CL/F)
- Peak to trough ratio calculated as $C_{max}/C_{trough}$
- Apparent volume of distribution ($V_d$/F)
- Metabolite-to parent ratio based on AUC calculated as $AUC_{metabolite}/AUC_{parent}$

- Metabolite-to parent ratio based on $C_{max}$ calculated as $C_{max,\ metabolite}/C_{max,\ parent}$

### 6.1.1 Pharmacokinetic sample collection

Detailed information on PK sample collection, handling, storage and transport will be provided to clinical sites in a separate PK brochure.

### 6.1.2 Pharmacokinetic sample analysis

For INS018_055 and its metabolites INS018_063 & INS018_095 in human plasma, pharmacokinetic samples will be analyzed using validated liquid chromatography-tandem mass spectrometry. Analytical results and validation details will be provided in a separate bioanalytical report.

## 6.2 SAFETY ASSESSMENT AND ENDPOINTS

Safety and tolerability will be assessed by the following endpoints: AE monitoring and recording, clinical laboratory results (routine blood, coagulation, blood biochemistry, urine routine and fecal occult blood test), vital sign measurements, 12-lead ECG results and physical examination results.

For all safety assessments, the investigator will determine whether the outcome is clinically significant, defined as any change in the outcome that is medically relevant and may result in a change in medical care (e.g., active observation, diagnostic measure, or therapeutic measure). If clinical significance is observed, the significant result and reason will be documented and the AE will be reported on the AE page of the subject's eCRF. the investigator will monitor the subject until the outcome reaches the reference range or outcome at screening, or until the investigator determines that follow-up is no longer medically necessary.

### 6.2.1 Adverse Events

Any adverse medical events that occurred from the time the subject signed the informed consent until the first dose of study drug was administered are recorded as medical history and in the CRF and are not recorded as AE. Adverse events will be evaluated from the time of INS018_055 administration through the EOS and should be followed until they subside, stabilize, or are judged by the investigator to be not clinically significant.

The investigator is responsible for ensuring that all AEs and SAEs are recorded in the eCRF and reported to the sponsor without regard to their relationship to the study drug or clinical significance. If there is any doubt as to whether a clinical observation is an AE, the event should be reported.

6.2.1.1 Adverse Event Definition

AEs are defined as all adverse medical events that occur after a subject receives the study drug and can manifest as signs and symptoms, disease, or abnormal laboratory tests that are not necessarily causally related to the study drug. Subjects will be instructed to contact the investigator at any time after randomization grouping if they develop any symptoms.

AE (TEAE) occurring during treatment was defined as any event that did not occur prior to exposure to the study drug or any event that was present but increased in severity or frequency after exposure.

An AE is considered a serious adverse event (SAE) reaction if, in the opinion of the investigator or sponsor, it results in any of the following outcomes:

- Death: When the outcome of an event is "death", it can be clearly recorded and reported as a serious adverse event;
- Life-threatening AE: in this context means that the subject is already at risk of death at the time of the adverse event, and does not mean that the adverse event is assumed to be likely to result in death if more severe;
- Need for hospitalization or extension of existing hospital stay: it needs to be clear that the cause of the condition was due to an adverse event and not an admission for elective surgery, non-medical reasons, etc;
- Permanent or severe disability or loss of function;
- Congenital anomalies or birth defects;
- Other Medically Significant Events: Other Medically Significant Events: These are adverse events that may not be immediately life-threatening, death or hospitalization, but are also usually considered serious if medical measures are needed to prevent the occurrence of one of the conditions listed above. Examples include critical treatment in the emergency room or allergic bronchospasm occurring at home, cachexia or convulsions without hospitalization, development of drug dependence or addiction.

An AE or suspected adverse reaction is considered "life-threatening" if, in the opinion of the investigator or sponsor, the occurrence of the AE or suspected adverse reaction would place the subject at immediate risk of death. AEs or suspected adverse reactions of increased severity that could result in death are not included.

Suspected and Unanticipated Serious Adverse Reaction (SUSAR) refers to a suspected and unanticipated serious adverse reaction where the nature and severity of the clinical manifestation exceeds the information available in the investigator's manual, the instruction manual of the marketed drug, or the summary of product characteristics.

6.2.1.2 Collecting and recording adverse events

Subjects will be asked a standard question to draw any medically relevant changes in their health. They will also be asked if they have been hospitalized, had any accidents, used any new medications or changed their concomitant medication regimen (prescription and over-the-counter medications).

In addition to subject observations, AEs will be documented based on any data collected from the AE page of the eCRF (e.g., laboratory test values, physical examination results, and ECG changes) or other documentation related to subject safety.

6.2.1.3 Reporting Adverse Events

All AEs reported or observed during the study will be recorded on the AE page of the eCRF. Information to be collected includes medication, type of event, time of occurrence, dose, investigator-specified severity and assessment of relationship to study drug, time to event resolution, severity, any required treatment or evaluation, and outcome. Any AE resulting from concurrent disease, response to concurrent disease, response to concomitant medication, or progression of disease state must also be reported. all AEs will be followed until they subside, stabilize, or are judged by the investigator to be not clinically significant. All AEs will be coded using the Medical Dictionary of Regulatory Activities (MedDRA).

Any medical condition present at the time of the subject's screening but not progressing should not be reported as AE; however, if the condition progresses at any time during the study period, it should be recorded as AE.

Subjects began collecting SAEs from the time they signed the informed consent form. were considered severe by the investigator or met the SAE criteria (Section 6.2.1.1 Any AE that is considered by the investigator to be serious or meets the criteria for an SAE (Section 6.2.1.1) must be reported to the sponsor immediately (after the investigator confirms that an SAE has occurred). The investigator will assess whether there is a reasonable probability that the investigational drug caused the SAE. The sponsor will be responsible for notifying the appropriate regulatory agency of any SAE as described in the current International Conference on Harmonization of Technical Requirements for Registration of Pharmaceutical Products for Human Use (ICH) Guidelines for Quality Control of Pharmaceutical Clinical Trials (GCP) and national regulatory requirements. the investigator is responsible for notifying the IRB directly.

Female subjects are required to withdraw from the trial immediately after the occurrence of a pregnancy; male subjects are not required to withdraw from the trial after the occurrence of a pregnancy in their partner. Even if a subject withdraws from the trial, he or she should be followed up as much as possible to document the outcome of the pregnancy (normal birth, preterm birth, spontaneous abortion, induced abortion, stillbirth, neonatal death or congenital malformation, etc.). Pregnancy itself is not considered an adverse event or a serious adverse event. If the outcome of a pregnancy meets the criteria for a serious adverse event, such as spontaneous abortion, stillbirth, neonatal death, or congenital malformation, the investigator should report it according to SAE reporting procedures. All neonatal deaths occurring within one month of birth

should be reported as SAEs, regardless of the cause of death. In addition, any infant death occurring after the first month of life should also be reported as an SAE, provided the investigator believes that the death may be related to the study drug.

The contact information for this study for the SAE report is as follows:

| | |
|---|---|
| Tiger Med Group Medical Monitor: | Li Hanyang Hanyang.li@tigermedgrp.com |

#### 6.2.1.4 Severity assessment

The severity (or intensity) of AE is the degree to which it affects the subject's daily activities and will be classified as mild, moderate, or severe using the following criteria:

- Mild: These events require little or no treatment and do not interfere with the subject's daily activities.
- Moderate: These events result in a low degree of inconvenience or require lesser therapeutic measures. Moderate events may interfere with normal function.
- Severe: These events interrupt the subject's daily activities and may require systemic medication or other treatment. Severe events usually disable the person.

Changes in AE severity should be recorded to allow assessment of the duration of events at each severity level. For AE characterized as intermittent, it is not necessary to record the start time and duration of each episode.

#### 6.2.1.5 Causality assessment

The investigator's assessment of the relationship of the AE to the study drug is part of the documentation process, but is not a factor in determining whether to report the appropriate content in the study.

The investigator will assess the causal relationship (i.e., whether it is reasonably probable that the study drug caused the event) for all AEs and SAEs. The relationships are categorized as follows:

- Not Relevant: Not reasonably likely to be related to the study drug.AE Does not follow a reasonable chronological sequence from the administration of the study drug or can be reasonably explained by the subject's clinical status or other factors (e.g., study disease, co-morbidities, and concomitant medications).
- Relevant: reasonably likely to be related to the study drug.AE follows a reasonable chronological sequence from the administration of the study drug and cannot be reasonably explained by the subject's clinical status or other factors (e.g., study disease, co-morbidities, or concomitant medications), indicates a known response to the study drug or other drugs in its class, is

consistent with the known pharmacological properties of the study drug, and/or recurs with study drug subsides with discontinuation (and/or recurses with re-challenge, as applicable)

#### 6.2.1.6 Adverse event follow-up

All AEs must be reported in detail on the appropriate page of the eCRF and followed until they subside, stabilize, or are judged by the investigator to be not clinically significant.

### 6.2.2 Clinical Laboratory Tests

Clinical laboratory tests will be performed on the day of screening, registration, and departure from the study site, as well as on Day 3.1 and other points in time as specified in Section 3.1. Clinical laboratory examinations will be performed by the local laboratory at the clinical research center. Blood and urine samples will be collected on an empty stomach and prepared according to standard protocols.

If necessary, clinical laboratory tests may be repeated at the discretion of the investigator to evaluate inclusion and exclusion criteria or clinical laboratory test abnormalities. The clinical laboratory performing the test will provide a reference range for all clinical laboratory parameters. Abnormal clinical laboratory test values will be flagged as high or low (or normal or abnormal) based on the reference range for each laboratory parameter.

The following clinical laboratory assessments will be performed:

| | |
|---|---|
| Blood Count | Erythrocyte pressure volume, hemoglobin, mean erythrocyte hemoglobin content, mean erythrocyte hemoglobin concentration, white blood cells (basophils, eosinophils, lymphocytes, monocytes, neutrophils), mean erythrocyte volume, platelet count, red blood cell count and erythrocyte volume distribution width |
| Blood Biochemistry | ALT, albumin, alkaline phosphatase, AST, bilirubin (total), urea, calcium, chloride, total cholesterol, creatinine, gamma glutamyl transferase, creatine kinase, glucose, lactate dehydrogenase, phosphorus, potassium, sodium, total protein, lipids (total cholesterol, triglycerides, LDL cholesterol, HDL cholesterol), uric acid, and cystatin C |
| Urinalysis | Appearance, bilirubin, color, glucose, ketones, leukocytes, nitrites, occult blood, pH, protein, specific gravity and urinary bilirubinogen |
| Fecal occult blood test | Presence of occult blood in stool samples |
| Serological Virology | Hepatitis B surface antigen, hepatitis C virus antibodies and HIV 1 and 2 antibodies (at screening only) |
| Other Analysis | All subjects: urine drug screen (morphine, methamphetamine, ketamine, tetrahydrocannabinol acid, methylenedioxymethamphetamine), cotinine screen, alcohol breath test, coagulation, COVID-19 screening report (according to the local government outbreak control policy and hospital control requirements, at screening or during the study, the investigator will confirm that the subject has a valid COVID-19 nucleic acid test report from a formal testing facility. COVID-19 nucleic acid test report provided by a formal testing institution and within the validity period will be confirmed by the investigator at the time of screening or during the study)<br>Female subjects: follicle stimulating hormone, serum pregnancy and urine pregnancy tests (human chorionic gonadotropin)<br>Potential Hay's Law cases: AST (retest), ALT (retest), total bilirubin (retest), albumin (retest), alkaline phosphatase (retest), direct bilirubin, creatine kinase, gamma glutamyl transferase, activated partial thromboplastin clotting time, prothrombin time and international normalized ratio and total bile acids |

### 6.2.3 Vital Signs Measurement

Vital signs include systolic and diastolic blood pressure, pulse rate, respiratory rate and body temperature.

**Part A:** Vital signs are measured at the following time points after the subject has rested for at least 5 minutes: screening; registration (Day -1); Day 1 (within 45 minutes prior to dosing) and 1 (± 30 minutes), 2 (± 30 minutes), 4 (± 30 minutes), 8 (± 30 minutes), and 12 hours (± 30 minutes) after dosing; morning of Days 2 through 4; EOS.

**Part B:** Vital signs will be recorded at screening, registration (Day -1), Days 1 through 11, and Day 28 (EOS). Days 1 and 7: Before dosing (within 45 minutes before dosing) and 1 (± 30 minutes), 2 (± 30 minutes), 4 (± 30 minutes), 8 (± 30 minutes), 12 (± 30 minutes) and 24 (± 60 minutes) hours after dosing. Vital sign measurements will be obtained before dosing on the morning of Days 2 through 6, the morning of Days 9 through 11, and at EOS, after the subject has rested for at least 5 minutes.

#### 6.2.4 Electrocardiogram

**Part A: At** screening and registration (Day -1), three standard 12-lead ECGs will be recorded with repeated measurements no more than 5 minutes apart and results will be averaged; single ECG acquisitions will be made at all other prescribed visits. ECG will be recorded on Day 1 before dosing (within 1 h before dosing) and 1 (± 15 min), 2 (± 15 min), 4 (± 15 min), and 8 hours (± 15 min) after dosing. additional ECG recordings will be made on Days 2 and 3, before leaving the study site on Day 4, and at EOS, after the subject has remained in the supine position for at least 5 minutes.

**Part B:** Three standard 12-lead ECGs will be recorded at screening and registration (Day -1); a single ECG will be taken at all other prescribed visits. ECGs will be recorded on Days 1 and 7 prior to dosing (within 1 h prior to dosing) and 1 (± 15 min), 2 (± 15 min), 4 (± 15 min), 8 (± 15 min), and 12 (± 15 min) hours after dosing. ECGs will be collected on day 11 before leaving the study site (Section 3.1).

The ECG evaluation will include the following remarks: whether the tracing is normal or abnormal, rhythm, presence of arrhythmias or conduction defects, morphology, and whether there is any evidence of myocardial infarction or ST-segment, T-wave, or U-wave abnormalities. In addition, the following interval measurements will be measured and reported: HR, RR interval, PR interval, QRS width, QT interval, and QT interval corrected for heart rate using the Bazett formula. Clinically relevant abnormalities that occur during the study should be recorded in the AE section of the eCRF.

#### 6.2.5 Physical examination

A full physical examination will be conducted at screening. A brief physical examination will be conducted at registration (Day -1) and at EOS.

A full physical examination will include at least skin, head, ear, eye, nose, throat, neck, thyroid, lung, heart, cardiovascular, abdominal, lymph node and musculoskeletal system/extremity assessments. A brief physical examination will include at least skin, lung, cardiovascular system, and abdominal (liver and spleen) assessments. If necessary, an interim physical examination may be performed at the discretion of the investigator to evaluate for abnormalities in AE or clinical laboratory tests.

**Part A:** Physical examinations will be performed at approximately 24 (Day 2) and 48 hours (Day 3) after dosing.

**Part B:** Physical examinations will be performed on days 2, 5, 7, and before leaving the study site on day 11 after dosing.

## 6.3 BIOMARKERS

will be shown inTable 3.1 -Table 3.11 (Part A) andTable 3.1 -Table 3.12 (Part B, QD/BID administration) for measurement of biomarkers. T-cell CD4+ and CD8+ subsets will be assessed in whole blood, and IL-6, TGF-β, MMP-2, MMP-9 and MMP-7 in serum.

### 6.3.1 CD4+ and CD8+ subpopulations of T cells

In nonclinical studies, INS018_055 was found to result in complex changes of lymphocyte populations, both in the circulating fraction and in lymphatic tissues. It is assumed that these changes reflect pharmacological effects of INS018_055, so that a change in circulating T-lymphocyte subpopulations could be a helpful biomarker for selection of the therapeutic dose range in late-stage clinical trials.

Characterization of T-cell subpopulations is included as a biomarker, because complex changes in lymphocytes have been observed in nonclinical studies and are assumed to represent major pharmacological effects. The latter assumption is based on the expression of the target in specific T-cells. Eosinophils, T-cells, and basophils are the group enriched with TNIK expression specifically in blood cells. A decrease in the level of lymphocytes may be due to inhibitory effects of INS018_055 on T-cell differentiation to short-lived subtypes. As described, several genome-wide association study studies revealed strong genetic associations between TNIK and lymphocyte count (PMID:27863252; PMID:32888494; PMID:32888493). CD27/TNIK/Wnt signaling favors memory T-cell differentiation and cell division, while TNIK-deficient T-cells preferentially differentiated into short-lived effector cells, while memory T-cell formation was impaired (PMID: 32242021). These observations strongly suggest the association between TNIK and lymphocytes. Therefore, INS018_055 may induce T-cell differentiation via TNIK-signaling, consequently leading to decreased levels of T-cells upon treatment.

Further details of blood collection and processing will be provided to the site in the procedure manual. Data for exploratory biomarkers may be reported separately from the clinical study report.

### 6.3.2 IL-6 and TGF-β

It is established that there is a crosstalk between IL-6 and Wnt signaling, where TNIK mediates the related biological process (**Error! Reference source not found.** et al 2002; **Error! Reference source not found.** et al 2017). The effect of INS018_055 on IL-6 expression has been confirmed in the BioMAP Fibrosis panel of 3 human primary cell-based systems cell-based studies (Study No. US034-0011915-O). The current PD analysis will confirm the effect on these markers in the plasma samples from healthy subjects. Additionally, the effects of INS018_055 on the protein levels of TGF-β and IL-6

are suggested to be assessed pre- and posttreatment. Such data were already generated in the mouse bleomycin model (Study No. ISM-ME-10-v): TGF-β and IL-6 increased in the BALF after BLM inhalation and were reduced after INS018_055 treatment. These data correlated with a positive effect of INS018_055 on the fibrotic readouts (modified Ashcroft score, collagen deposit in lung)

#### 6.3.3 MMP-2, MMP-9 and MMP-7

Several protein biomarkers, MMP-2, MMP-9, and MMP-7, also encoded by the target genes of Wnt signaling have been shown to be increased in IPF (eg, matrix metalloproteinase (MMP)-2, MMP-9 (**Error! Reference source not found.** et al 2007; **Error! Reference source not found.** et al 2020), MMP-7 (**Error! Reference source not found.** et al 1999; **Error! Reference source not found.** et al 2008; **Error! Reference source not found.** et al 2015). Higher levels of these MMPs have been identified to be associated with disease progression and worse survival. These results will help to elaborate the mechanism of the effect of INS018_055 on IPF pathophysiology and will guide endpoint selection in future clinical studies.

# 7. STATISTICAL ANALYSIS PROTOCOL

## 7.1 SAMPLE SIZE CALCULATION

The number of subjects was determined based on clinical and practical considerations, rather than on formal statistical certainty calculations. The total sample size of 56 evaluable subjects was considered sufficient for the purpose of the study.

## 7.2 ANALYSIS SET

The analysis population is as follows:

- Safety Analysis Set (SS): The safety population will include all randomized enrolled subjects who receive at least 1 dose of INS018_055 or placebo.
- PK Analysis Set (PKS): The PK population will include subjects who receive at least 1 dose of INS018_055 with concentration data sufficient to support accurate estimation of at least 1 PK parameter. Subjects who receive study drug administration followed by vomiting within 2 times the median time of $T_{max}$ will be excluded from the PK analysis.
- PD Analysis Set (PDS): The PD population will include subjects who receive at least 1 dose of INS018_055 or placebo and have valid baseline (pre-dose) values and at least 1 non-deficient biomarker measure.

## 7.3 DESCRIPTION OF THE SUBGROUP TO BE ANALYZED

Not applicable.

## 7.4 STATISTICAL ANALYSIS

Details of all statistical analyses will be described in a separate statistical analysis plan. All data collected will be listed in the data tabulation. Data for subjects excluded from the analysis population will be listed in the data tabulation but will not be included in the calculation of summary statistics. Data for subjects receiving placebo in each cohort will be pooled into 1 group for analysis.

For categorical variables, frequencies and percentages will be presented. Descriptive statistics (number of subjects, mean, median, SD, minimum and maximum) will be used to summarize continuous variables.

An overall summary of baseline demographics and background variables will be presented for all subjects. The number of subjects in the cohort study and the number and percentage of subjects who completed the study will be listed. The frequency and percentage of subjects who withdrew or discontinued from the study and the reasons for withdrawal or discontinuation will also be summarized.

### 7.4.1 Pharmacokinetic analysis

Plasma concentrations will be listed and summarized in a descriptive manner (number of subjects, arithmetic mean, SD, coefficient of variation [CV], median, geometric mean, geometric CV, minimum and maximum values). The plasma concentration-actual time curve for each subject will be displayed graphically. The mean plasma concentration-scheduled time curve will be displayed graphically.

Descriptive statistics (number of subjects, mean, SD, CV, geometric mean, geometric CV, median, minimum and maximum) will be used to summarize by treatment group using Phoenix® WinNonlin® (Certara USA Inc., Princeton, New Jersey) version 8.3.1 or higher or SAS version (SAS Institute Inc., Cary, North Carolina) PK parameters for plasma samples obtained by non-atrial methods. However, only the following descriptive statistics will be used to summarize $T_{max}$ : number of subjects, median, minimum and maximum values.

Power Model examines the dose proportionality. Plots of AUC and $C_{max}$ versus dose will be provided. The model is defined as:

$$\ln[\text{PK parameter}] = \beta_0 + \beta_1 \ln[\text{dose}]$$

where the PK parameter is AUC or $C_{max}$ . The null hypothesis tested is that the AUC and $C_{max}$ values vary proportionally with dose, or that the slope ($\beta_1$) = 1.

Steady state was assessed by repeated comparisons of individual subjects' trough concentrations in samples collected from day 1 to day 7 by Mixed Model analysis. With this analysis, the trough concentration values were log-transformed and then compared with the log-transformed trough concentration values at that time point and all other trough concentration values after that time point. The GeoLSM ratios and 90% confidence intervals were summarized.

#### 7.4.2 Safety Analysis

Adverse events will be coded by preferred terminology and system organ classification using the latest version of MedDRA. All AE data will be presented in a data list. AEs occurring during treatment will be summarized by treatment group and subject as a whole, as well as by severity and relationship to study drug. severe AEs, AEs leading to discontinuation of study drug and death will also be listed in the data list and summarized by treatment group and subject as a whole.

Actual values and changes relative to baseline for clinical laboratory test results, vital sign measurements, and 12-lead ECG results are summarized by treatment group at each time point using descriptive statistics (number of subjects, mean, SD, median, minimum, and maximum). A table of changes in clinical laboratory results will be generated. The number and percentage of subjects will be summarized by visit for fecal occult blood results. Clinical laboratory results, vital sign measurements, 12-lead ECG results, and physical examination results are presented in the data list.

#### 7.4.3 Biomarkers Analysis

Biomarker analysis summaries will be performed based on the PD population. Data from subjects who received placebo in each cohort will be pooled into 1 group for analysis.

Summary statistics, change, and percentage change relative to baseline will be presented for each biomarker by visit and time point. The last recheck value collected prior to the first dose of study treatment will be considered as baseline and all recheck values will be excluded from the post-administration observations when calculating the summary statistic.

### 7.5 MID-TERM ANALYSIS

No formal interim safety analysis will be conducted in this study.

# 8. REFERENCE LIST

Camara J, Jarai G. Epithelial-mesenchymal transition in primary human bronchial epithelial cells is Smad-dependent and enhanced by fibronectin and TNF-alpha. Fibrogenesis Tissue Repair. 2010;3(1):2.

Cao H, Wang C, Chen X, et al. Inhibition of Wnt/beta-catenin signaling suppresses myofibroblast differentiation of lung resident mesenchymal stem cells and pulmonary fibrosis. Sci Rep. 2018;8(1):13644.

Chapman H. Epithelial-mesenchymal interactions in pulmonary fibrosis. Annu Rev Physiol. 2011;73:413-35.

Chilosi M, Poletti V, Zamò A et al. Aberrant Wnt/beta-catenin pathway activation in idiopathic pulmonary fibrosis. Am J Pathol. 2003;162(5):1495-502.

Crawford HC, Fingleton BM, Rudolph-Owen LA, et al. The metalloproteinase matrilysin is a target of beta-catenin transactivation in intestinal tumors. Oncogene. 1999;18(18):2883-91.

Department of Health and Human Services ( DHHS ), Food and Drug Administration, Center for Drug Evaluation and Research (US). Guidance for Industry: Estimating the maximum safe starting dose in initial clinical trials for therapeutics in adult healthy volunteers. July 2005 [ cited 2017 May 18]. cited 2017 May 18]. Available from: http://www.fda.gov/downloads/Drugs/Guidances/UCM078932.pdf.

Hou J, Ma T, Cao H, et al. TNF-α-Induced NF-κB Activation Promotes Myofibroblast Differentiation of LR-MSCs and Exacerbates Bleomycin-Induced Pulmonary Fibrosis. j Cell Physio. 2018;233(3):2409-19.

Jenkins RG, Simpson JK, Saini G, et al. Longitudinal change in collagen degradation biomarkers in idiopathic pulmonary fibrosis: an analysis from the Lancet Respir Med. 2015;3(6): 462-72.

Katzenstein AL, Myers JL. Idiopathic pulmonary fibrosis: clinical relevance of pathologic classification. Am J Respir Crit Care Med. 1998;157(4 Pt 1). 1301-15.

Kim K, Kugler MC, Wolters PJ, et al. Alveolar epithelial cell mesenchymal transition develops in vivo during pulmonary fibrosis and is regulated by the Proc Natl Acad Sci USA. 2006;103(35):13180-5.

King TE Jr, Bradford WZ, Castro-Bernardini S, et al. A phase 3 trial of pirfenidone in patients with idiopathic pulmonary fibrosis. N Engl J Med. 2014;370( 22):2083-92.

Lee JU, Cheong HS, Shim EY, et al. Gene profile of fibroblasts identify relation of CCL8 with idiopathic pulmonary fibrosis. Respir Res. 2017;18(1):3.

Lee Y, Jung JI, Park KY, et al. Synergistic inhibition effect of TNIK inhibitor KY-05009 and receptor tyrosine kinase inhibitor dovitinib on IL-6- induced proliferation and Wnt

signaling pathway in human multiple myeloma cells. oncotarget. 2017;8(25):41091-101.

Longo KA, Kennell JA, Ochocinska MJ, et al. Wnt signaling protects 3T3-L1 preadipocytes from apoptosis through induction of insulin-like growth factors. J Biol Chem. 2002;277(41):38239-44.

Mahmoudi T, Li VS, Ng SS, et al. The kinase TNIK is an essential activator of Wnt target genes. EMBO J. 2009;28(21):3329-40.

Morrisey EE. Wnt signaling and pulmonary fibrosis. Am J Pathol. 2003;162(5):1393-7.

Oda K, Yatera, K, Izumi, H, et al. Profibrotic role of WNT10A via TGF-β signaling in idiopathic pulmonary fibrosis. Respir Res. 2016;17:39.

Piguet PF, Ribaux C, Karpuz V, et al. Expression and localization of tumor necrosis factor-alpha and its mRNA in idiopathic pulmonary fibrosis. Am J Pathol. 1993;143(3):651-5.

Richeldi L, Du Bois RM, Raghu G, et al. Efficacy and safety of nintedanib in idiopathic pulmonary fibrosis. N Engl J Med. 2014;370(22):2071-82.

Rosas IO, Richards TJ, Konishi K, et al. MMP1 and MMP7 as potential peripheral blood biomarkers in idiopathic pulmonary fibrosis. PLoS Med. 2008;5(4). PLoS Med. 2008;5(4): e93.

Selman M, King TE, Pardo A; American Thoracic Society; European Respiratory Society; American College of Chest Physicians. Idiopathic pulmonary fibrosis: prevailing and evolving hypotheses about its pathogenesis and implications for therapy. Idiopathic pulmonary fibrosis: prevailing and evolving hypotheses about its pathogenesis and implications for therapy. Ann Intern Med. 2001;134(2):136-51.

Shitashige M, Satow R, Jigami T, et al. Traf2- and Nck-interacting kinase is essential for Wnt signaling and colorectal cancer growth. Cancer Res. 2010. 70(12):5024-33.

Shkoda A, Town JA, Griese J, et al. The germinal center kinase TNIK is required for canonical NF- B and JNK signaling in B-cells by the EBV oncoprotein LMP1 and the CD40 receptor. PLoS Biol. 2012;10(8):e1001376.

Todd JL, Vinisko R, Liu Y, et al. Circulating matrix metalloproteinases and tissue metalloproteinase inhibitors in patients with idiopathic pulmonary fibrosis in the multicenter IPF-PRO Registry cohort. BMC Pulm Med. 2020; 20(1): 64. https://doi.org/10.1186/s12890- 020-1103-4.

Weigle S, Martin E, Voegtle A, et al. Primary cell-based phenotypic assays to pharmacologically and genetically study fibrotic diseases in vitro. J Biol Methods. 2019;6(2):e115.

Wu B, Crampton SP, Hughes CC. Wnt signaling induces matrix metalloproteinase expression and regulates T cell transmigration. Immunity. 2007;26(2). 227-39.

Yamada T, Masuda M. Emergence of TNIK inhibitors in cancer therapeutics. Cancer Sci. 2017;108(5):818-23.

# 9. PROTOCOL REVISION RECORD

See separate document ("Revised Description for Protocol INS018-055-002").

# 10. APPENDIX

## 10.1 APPENDIX 1 : LIST OF ABBREVIATIONS

| Abbreviations | Terminology |
|---|---|
| α-SMA | α-smooth muscle actin |
| AE | adverse event |
| ALT | alanine aminotransferase |
| AR | accumulation ratio |
| AST | aspartate aminotransferase |
| AUC | area under the plasma concentration versus time curve |
| $AUC_{0-inf}$ | area under the plasma concentration versus time curve from time 0 extrapolated to infinity |
| $AUC_{\tau}$ | area under the plasma concentration versus time curve from time 0 to the time of the dosing interval |
| $AUC_{0-t}$ | area under the plasma concentration versus time curve from time 0 to the last quantifiable concentration |
| BID | twice daily |
| BLM | bleomycin |
| BLQ | below the limit of quantification |
| BP | blood pressure |
| $C_{av}$ | average concentration on Day 1 and Day 7 |
| CI | confidence interval |
| CKD-EPI | Chronic Kidney Disease Epidemiology Collaboration |
| CL/F | apparent total body clearance |
| CLr | renal clearance |
| $C_{max}$ | maximum observed plasma concentration |
| COVID-19 | Coronavirus disease 2019 |
| Ctrough | pre-dose concentrations on Days 1 through 7 |
| CV | coefficient of variation |
| CYP | cytochrome P450 |
| ECM | extracellular matrix |
| ECG | electrocardiogram |
| eCRF | electronic case report form |
| EMT | epithelial to mesenchymal transition |
| EOS | end of study |
| FMT | fibroblast to myofibroblast transformation |
| GCP | Good Clinical Practice |
| GI | gastrointestinal |
| HIV | human immunodeficiency virus |
| IB | investigator's brochure |
| ICF | Informed Consent Form |
| ICH | International Conference on Harmonization of Technical Requirements for Registration of Pharmaceutical Products for Human Use |
| IMP | investigational medicinal product |
| IP | investigational [medicinal] product |
| IPF | idiopathic pulmonary fibrosis |

| Abbreviations | Terminology |
|---|---|
| IRB | institutional review board |
| IUD | intrauterine device |
| $K_{el}$ | terminal elimination rate constant |
| QD | Once a day |
| QTc | QT interval derived by Bazett |
| MAD | multiple ascending dose |
| MATE | multidrug and toxin extrusion |
| MedDRA | Medical Dictionary for Regulatory Activities |
| MMP | matrix metalloproteinase |
| NOAEL | no observed adverse effect level |
| PD | pharmacodynamic |
| PK | pharmacokinetic(s) |
| SAD | single ascending dose |
| SAE | serious adverse event |
| SARS-CoV-2 | severe acute respiratory syndrome-related coronavirus 2 |
| SCr | serum creatinine |
| SOE | schedule of events |
| SRC | safety review committee |
| $t_{1/2}$ | terminal phase half-life |
| TGF | transforming growth factor |
| $T_{max}$ | time to maximum observed plasma concentration |
| TNIK | Traf2- and Nck-interacting kinase |
| ULN | upper limit of normal |
| $V_d$ /F | apparent volume of distribution |

# 10.2 APPENDIX 2 : STUDY MANAGEMENT

## 10.2.1 Data Quality Assurance

This study will be conducted in accordance with the quality processes described in the applicable procedural documents. The quality management methods implemented will be documented and will be consistent with current ICH guidance on quality and risk management. All aspects of the study will be monitored for compliance with applicable government regulatory requirements, NMPA GCP (version 2020), protocols, and standard operating procedures. The monitor will maintain an up-to-date understanding of study progress through observation, review of study records and source documents, and discussions with investigators and staff regarding study implementation. Electronic case report forms and electronic data capture systems will be used. The electronic data capture system has been validated to meet national regulatory requirements. Each person participating in the study will be given a personal identification code and password to enable traceability to records.

Significant protocol deviations (if they occur during the study) will be listed in Section 10.2 of the Clinical Study Report.

## 10.2.2 Investigator Obligations

The following management items are intended to guide investigators in conducting research and may be subject to change in accordance with industry and government standard operating procedures, work practice documents, or guidelines. Changes will be reported to the IRB, but will not result in amendments.

### 10.2.2.1 Confidentiality

All laboratory specimens, evaluation forms, reports, and other records should be identified in a manner that maintains the confidentiality of the subject's identity. All records will be maintained in a secure storage area with restricted access. Clinical information shall not be released without the written permission of the subject (or the subject's legal guardian) unless the information is required for monitoring and auditing by the sponsor, its designee, the applicable regulatory agency, or the IRB.

The investigator and all employees and associates involved in this study shall not disclose or use for any purpose other than this study any data, records, or other confidential and unreleased information disclosed to such persons for research purposes. Prior written consent must be obtained from the sponsor or its designee before any said confidential information may be disclosed to other parties.

### 10.2.2.2 Institutional Review

The Declaration of Helsinki (2013 edition), NMPA GCP (2020 edition), and ICH guidelines require IRB approval prior to human subjects participating in the study. The protocol, ICF, advertisements used to recruit study subjects, and any other written information provided to the subject or the subject's legal guardian regarding this study

must be approved by the IRB prior to the start of the study. All IRB approval documentation and documentation demonstrating that the IRB meets the requirements of ICH Tripartite Harmonization Guideline E6 (R2) and NMPA GCP (2020 Edition): GCP will be maintained by the Research Center and available for review by the sponsor or its designee.

All IRB approvals should be signed by the IRB chair or designee and must include the IRB name and address, the clinical protocol listed by title or protocol number or both, and the date of approval or favorable opinion given.

10.2.2.3 Subjects' consent form

Written informed consent shall be obtained in accordance with ICH E6 (R2) guidelines and applicable regulatory requirements prior to each subject's entry into the study or prior to any unusual or nonroutine procedures involving risk to the subject. If the investigational center proposes or makes any institution-specific changes to study-related procedures, the sponsor or its designee, or both, shall review the consent prior to submission to the IRB. Following review, the investigator will submit the ICF to the IRB for review and approval prior to the start of the study. If revisions are made to the ICF during the study, all subjects who are participating must sign the revised consent form.

Prior to recruitment and enrollment, each prospective subject or their legal guardian will be provided with a complete explanation of the study and allowed to read the approved ICF, and if the investigator is assured that the subject/legal guardian understands the implications of participation in the study, the subject/legal guardian will be asked to give consent to participate in the study by signing the ICF. A copy of the ICF will be provided to the subject/legal guardian.

10.2.2.4 Study Reporting Requirements

By participating in this study, the investigator agrees to submit SAE reports in accordance with the timeline and methods outlined in this protocol. In addition, the investigator agrees to submit annual reports to their IRB (if applicable).

10.2.2.5 Investigator Documentation

Prior to the start of the study, the investigator will be asked to provide the required documentation (including, but not limited to, the following documents) to comply with Section 8.2 of ICH E6 (R2):

- IRB Approval
- Original investigator agreement page signed by the investigator
- Curriculum vitae of the principal investigator and each assistant investigator. A current license must be indicated on the CV. The CV will be signed and dated by the principal investigator and assistant investigator at the time of study initiation to indicate that it is accurate and up-to-date.

- The IRB-approved ICF, samples of study site advertisement used to recruit for this study, and any other written information provided to the subject or legal guardian regarding this study
- Laboratory accreditation and reference ranges for any local laboratories used by the study site

10.2.2.6 Study Conduct

The investigator agrees to conduct all aspects of this study in accordance with the following: ethical principles derived from the Declaration of Helsinki, ICH E6 (R2): NMPA GCP (version 2020); protocol; and all national, state, and local laws or regulations.

10.2.2.7 Case report forms and source documents

Study site personnel will maintain source files, enter subject data into eCRF as accurately as possible, and will respond quickly to any reported discrepancies.

Electronic CRF and electronic data capture will be used. The electronic data capture system has been validated to meet national regulatory requirements. Each individual participating in the study will have an individual identification code and password to enable traceability of records. Thus, the system and any subsequent study review will identify the coordinator, the investigator, and the individual entering or modifying the record, as well as the time and date of any modifications. Internal quality audits of the data may be performed, with additional review by clinical monitors.

Each eCRF is presented as an electronic copy, allowing data entry by study site personnel who can add and edit data, add new subjects, identify and resolve discrepancies, and view records. The system immediately transfers data directly to the database and allows for immediate detection of discrepancies, thus enabling study site coordinators to resolve and manage discrepancies in a timely manner.

Paper copies of eCRF and other database reports can be printed and signed by the investigator. The system enables research center personnel, monitors and reviewers to access hard copy audit, discrepancy review and investigator remarks information.

10.2.2.8 Adherence to Protocol

The investigator agrees to conduct the research described in this protocol in accordance with ICH E6 (R2) and all applicable guidelines and regulations.

10.2.2.9 Reporting Adverse Events

By participating in this study, the investigator agrees to submit SAE reports in accordance with the timeline and methods outlined in this protocol. In addition, the investigator agrees to submit annual reports to their IRB (if applicable). The investigator also agrees to provide the appropriate reports to the sponsor shortly after the investigator completes study participation (if applicable).

10.2.2.10 Investigator's Final Report

Upon completion of the study, the investigator (if applicable) shall notify the institution; the investigator/institution shall provide the IRB with a summary of the study outcomes and provide any required reports to the sponsor and regulatory agencies.

10.2.2.11 Record Retention

For clinical trials used to apply for drug registration, the required documents should be kept until at least 5 years after the test drug is approved for marketing; for clinical trials not used to apply for drug registration, the required documents should be kept until at least 5 years after the termination of the clinical trial. However, if required by applicable regulatory requirements or agreements with the sponsor, these documents should be retained for a longer period of time. The sponsor is responsible for notifying the investigator/institution when retention of these documents is no longer necessary.

10.2.2.12 Publications

Upon completion of the study, the data may be considered for presentation at a scientific meeting or publication in a scientific journal. In these cases, the sponsor will be responsible for these activities and will work with the investigator to determine how the manuscript will be prepared and edited, the number and order of authors, the publications to be submitted, and other relevant issues. The sponsor has final approval authority for all such issues.

Data are the property of the sponsor and cannot be published without their prior authorization, but data and any publication thereof will not be unduly withheld.

### 10.2.3 Study Management

10.2.3.1 Monitoring

10.2.3.1.1 Study Monitoring

The clinical monitor, as a representative of the sponsor, has an obligation to follow the study closely. During this process, the monitor will visit the investigator and study site on a regular basis, in addition to maintaining necessary telephone and email contact. The monitor will maintain up-to-date personal knowledge of the study through observation, review of study records and source documents, and discussions with investigators and staff about study implementation.

The sponsor or its designee will carefully monitor all aspects of the study for compliance with the applicable government regulatory requirements of the current ICH E6 (R2) guidelines and standard operating procedures.

10.2.3.1.2 Inspection of Records

The Investigator and institution participating in the study will allow study-related monitoring, audits, IRB review, and regulatory verifications by providing direct access

to all research records. In the event of an audit, the investigator agrees to allow the sponsor, its representatives, and applicable regulatory agencies access to all research records.

The Investigator shall immediately notify the sponsor and study site(s) of any audits scheduled by any regulatory agency and shall immediately forward a copy of any audit report received to the Sponsor.

10.2.3.2 Management of protocol amendments and deviations

10.2.3.2.1 Modification of the protocol

The sponsor or designee must review and approve any changes in this research activity, except those necessary to eliminate apparent immediate hazard to subjects. Protocol amendments must be submitted in writing to the investigator IRB for approval prior to enrollment of subjects in the amended protocol.

10.2.3.2.2 Protocol deviation

The investigator or designee must document and explain any deviations from the approved protocol in the subject source documentation. Investigators may implement protocol deviations or changes without prior IRB approval in order to eliminate immediate hazard to study subjects. As soon as possible after such an event, the implemented deviation or change, the reasons for implementation, and any proposed protocol amendments should be submitted to the IRB for review and approval, to the sponsor for concurrence, and to the regulatory agency, if necessary.

A protocol deviation is any change, divergence, or deviation from the study design or procedures relative to those defined in the protocol. Significant deviations (sometimes referred to as major or significant deviations) are a subset of protocol deviations and are deviations that could result in termination of the study by the subject or that significantly affect the rights, safety or well-being of the subject and/or the completeness, accuracy and reliability of the study data. Significant deviations may include nonadherence to inclusion or exclusion criteria or nonadherence to ICH E6 (R2) guidelines.

Protocol deviations will be documented by the clinical monitor throughout the monitoring visit. The monitor will notify the investigator of the deviation in writing. The IRB should be notified of all protocol deviations (if applicable) in a timely manner.

10.2.3.3 Study termination

Although the sponsor's intent is to complete the study, it reserves the right to terminate the study at any time for clinical or administrative reasons.

End of study is defined as the date the last subject completed the last visit (including the EOS visit and any additional long-term follow-up). Any additional long-term follow-up visits required to monitor AE or abnormal outcome regression may be attached to the clinical study report.

#### 10.2.3.4 Final Report

Regardless of whether the study is completed or prematurely terminated, the sponsor will ensure that clinical study reports are prepared and provided to regulatory agency(ies) as required by the applicable regulatory requirement(s). The sponsor will also ensure that the clinical study report in the marketing application meets the criteria in ICH Tripartite Harmonization Guideline E3, "Structure and Content of Clinical Study Reports".

Investigator signatories will be identified to approve clinical study reports in accordance with applicable regulatory requirements. Investigators will be provided with reasonable access to statistical tables, graphs and related reports and will be given the opportunity to review the complete study results.

A final approved clinical study report will be provided to the investigator upon completion of the clinical study report, as approprate.

## 10.3 APPENDIX 3: COCKCROFT -GAULT EQUATIO

$$\text{Creatinine clearance} = \frac{(140\text{-age(year)}) \times \text{body weight(kg)}}{\text{Serum creatinine}\left(\frac{\mu\text{mol}}{\text{L}}\right) \times 0.818} \times \text{Gender correction factor(male：1.00，female：0.85)}$$

## 10.4 APPENDIX 4: PROHIBITED COMBINED MEDICATIONS

**Table 8 : Known inhibitors of CYP3A4 and CYP2C8**

| | Strong Inhibitors | Medium Inhibitors | Weak Inhibitors |
|---|---|---|---|
| CYP2C8 | Gemfibrozil | Clopidogrel, Deferasirox, Teriflunomide | Methotrexate |
| CYP3A4 | Poprevir, cobicistat , danoprevir, ritonavir, etilaprevir, grapefruit juice, indinavir, itraconazole, ketoconazole, lopinavir, paritaprevir, ombitasvir, dasabuvir, posaconazole, saquinavir, telaprevir, telanavir, telithromycin, mulindomycin, voriconazole, clarithromycin, edelaris, nefazodone, nelfinavir | Arepitant, Ciprofloxacin, Conivaptan, Crizotinib, Cyclosporine, Diltiazem, Dronedarone, Erythromycin, Fluconazole, Fluvoxamine, Imatinib, Tofopam, Verapamil | Cloxazone, cilostazol, cimetidine, clotrimazole, fosaprepitant, etretinate, ivacaftor, lomitapide, ranitidine, ranolazine, tegretol |

**Table 9 : Known inducers of CYP3A and CYP2C8**

| | Strong Inducer | Medium Inducer | Weak Inducer |
|---|---|---|---|
| CYP2C8 | - | Rifampin | - |
| CYP3A | Apalutamide, carbamazepine, enzalutamide, mitotane, phenytoin, rifampin, St. John's wort | Bosentan, efavirenz, etravirine, phenobarbital, paracetamol | Armofamide, Modafinil, Lufimide |

**Table 10 : P-gp inhibitors and inducers**

| Transporter | Inhibitors | Inducer |
|---|---|---|
| P-gp | Amiodarone, carvedilol, clarithromycin, dronedarone, itraconazole, lapatinib, lopinavir and ritonavir, propafenone, quinidine, ranolazine, ritonavir, saquinavir and ritonavir, telaprevir, telanavir and ritonavir, verapamil | Avameb, carbamazepine, phenytoin, rifampin, St. John's wort, tipranavir/ritonavir |

# 1 References

[1] State Drug Administration, Guidelines for planning and reporting of drug clinical trial data management and statistical analysis, No. 113, 2016.

[2] State Drug Administration, Biostatistical guidelines for drug clinical trials, No. 93, 2016.

[3] International Conference on Harmonisation of Technical Requirements for Registration of Pharmaceuticals for Human Use, ICH Harmonised Tripartite Guideline, Statistical Principles for Clinical Trials (E9), 5 February 1998.

[4] International Conference on Harmonization of Technical Requirements for Registration of Pharmaceuticals for Human Use, ICH Harmonised Tripartite Guideline, Structure and Content of Clinical Study Reports (E3), 30 November 1995.

# 2 Appendix

## 2.1 Appendix 1 SAS code example

```
proc mixed data =PKP METHOD=REML ORDER=INTERNAL.

model log_pkpraw = log_dose / S.

estimate 'slope (beta) and 90% Confidence Interval' log_dose 1 / alpha=0.10.

ods output estimates = _slope_from_MIXED.

ods output coef = _coef.

ods output solutionf = _solutionf_from_MIXED.

run.
```

## 2.2 Appendix 2 Tables, Tables, Charts

PK-SAP Shell is available in a separate document.
