# Supplementary material for: A small-molecule TNIK inhibitor targets fibrosis in preclinical and clinical models
Source: Nat Biotechnol. 2024 Mar 8;43(1):63–75. doi: 10.1038/s41587-024-02143-0 (PMC11738990; doi:10.1038/s41587-024-02143-0)
Supplement: Supplementary file 2 — Reporting Summary [file 41587_2024_2143_MOESM2_ESM.pdf]

nature portfolio

Corresponding author(s): Alex Zhavoronkov, PhD

Last updated by author(s): Dec 22, 2023

Reporting Summary

Nature Portfolio wishes to improve the reproducibility of the work that we publish. This form provides structure for consistency and transparency in reporting. For further information on Nature Portfolio policies, see our Editorial Policies and the Editorial Policy Checklist.

Statistics

For all statistical analyses, confirm that the following items are present in the figure legend, table legend, main text, or Methods section.

n/a	Confirmed	
☐	☒	The exact sample size (*n*) for each experimental group/condition, given as a discrete number and unit of measurement
☐	☒	A statement on whether measurements were taken from distinct samples or whether the same sample was measured repeatedly
☐	☒	The statistical test(s) used AND whether they are one- or two-sided<br>*Only common tests should be described solely by name; describe more complex techniques in the Methods section.*
☒	☐	A description of all covariates tested
☐	☒	A description of any assumptions or corrections, such as tests of normality and adjustment for multiple comparisons
☐	☒	A full description of the statistical parameters including central tendency (e.g. means) or other basic estimates (e.g. regression coefficient) AND variation (e.g. standard deviation) or associated estimates of uncertainty (e.g. confidence intervals)
☐	☒	For null hypothesis testing, the test statistic (e.g. *F*, *t*, *r*) with confidence intervals, effect sizes, degrees of freedom and *P* value noted<br>*Give P values as exact values whenever suitable.*
☒	☐	For Bayesian analysis, information on the choice of priors and Markov chain Monte Carlo settings
☒	☐	For hierarchical and complex designs, identification of the appropriate level for tests and full reporting of outcomes
☒	☐	Estimates of effect sizes (e.g. Cohen's *d*, Pearson's *r*), indicating how they were calculated

Our web collection on statistics for biologists contains articles on many of the points above.

Software and code

Policy information about availability of computer code

Data collection	For target discovery part, all data used in this manuscript are public available, and described in methods and materials.
Data analysis	For target discovery part, the analytical softwares used in this manuscript (PandaOmics v2.0 and Chemistry42) are commercially available. We have provided supplementary information for both software in the manuscript files.<br>For pre-clinical data, raw data were analyzed using Microsoft Excel software(Microsoft 365), GraphPad PRISM (Versions 6, 8) or SPSS software (Versions 25 and later). Versions used varies in different contract research organizations who performed the studies.<br>For clinical phase 0 data (Australia), analyses were performed using Phoenix WinNonlin software (Version 8.3 or higher, Certara, USA).<br>For clinical phase 1 data (New Zealand), all analyses were performed using SAS® Version 9.4.<br>For clinical phase 1 data (China), analyses were performed using SAS version 8.3.1 or higher.

For manuscripts utilizing custom algorithms or software that are central to the research but not yet described in published literature, software must be made available to editors and reviewers. We strongly encourage code deposition in a community repository (e.g. GitHub). See the Nature Portfolio guidelines for submitting code & software for further information.

Data

Policy information about availability of data

All manuscripts must include a data availability statement. This statement should provide the following information, where applicable:

- Accession codes, unique identifiers, or web links for publicly available datasets
- A description of any restrictions on data availability
- For clinical datasets or third party data, please ensure that the statement adheres to our policy

PandaOmics v2.0 and Chemistry42 are commercially available:
https://insilico.com/pandaomics
https://insilico.com/chemistry42
We havea also provided full information on the drug discovery pipelines used in this mansucript in our supplementary information section.

Data Availability Statement:
Raw data from all experimental studies are available for public access through our repository which can be found at www.insilico.com/nbt-ins018-055-tnik/. The RNA sequencing data is available for download through this repository link along with all relevant source and raw data that were presented in this manuscript. The only two datasets which are not uploaded are the EMT/FMT full imaging set and the inhalation pathology imaging set as together these images are approximately 1 TB in size. The quantitation sheets are available in the repository and the images can be provided as a physical drive for any reasonable, public requests. This statement has been included in the manuscript as well.

Humans that participated in all of the clinical trials in this study provided written consent towards their study participation.

Clinical Datasets:
Phase 0 Study (Australia)
A phase 0 micro dosing clinical trial was conducted in Australia (ACTRN12621001541897). The complete information on clinical trial registration, study protocol, data collection and outcomes are provided in the reporting summary as well as in Supplementary information 9. This includes statistical considerations, study design, patient selection criteria, procedures, outcomes and PK analysis.

Phase I study (New Zealand):
The general design of clinical trial (NCT05154240) can be found in www.clinicaltrials.gov The randomized, double blind, placebo controlled study of INS018_055 was conducted from Feb 21, 2022 (first subject administered first dose) until Sept 30th, 2022 (last subject contacted). The complete information on clinical trial registration, study protocol, data collection and outcomes are provided in the reporting summary as well as in Supplementary information 10. This includes statistical considerations, study design, patient selection criteria, procedures, outcomes and PK analysis.

Phase I study (China):
Study design and Outcomes:
Detailed information including facilities, inclusion/exclusion criteria could be found in link http://www.chinadrugtrials.org.cn/clinicaltrials.prosearch.dhtml (registration number: CTR20221542).
The complete information on clinical trial registration, study protocol, data collection and outcomes are provided in the reporting summary as well as in Supplementary information 11. This includes statistical considerations, study design, patient selection criteria, procedures, outcomes and PK analysis.

Patient Samples for EMT and FMT Assays:
All tissues used for isolation are obtained under informed consent and conform to HIPAA regulations to protect the privacy of the donor's Personally Identifiable Information. The entirety of this study adheres to the Declarations of Helsinki. Quantitation of donor cell lines used are included in the repository link for this study provided above and identified imaging datasets for these studies can be provided in the form of a physical drive as the datasets are nearly 1 TB in size.

Publicly Available -Omics Datasets Utilized in this Study:
GSE93606, GSE38958, GSE28042, GSE33566 derived from blood tissue and GSE101286, GSE72073, GSE150910, GSE92592, GSE52463, GSE83717, GSE21369, GSE15197, GSE99621, GSE138283, GSE24206 derived from lung tissue. GSE136831 - was used for scRNA-seq analysis. These datasets were deposited into the GEO and were utilized in the contexts indicated following each accession number.

Human research participants

Policy information about studies involving human research participants and Sex and Gender in Research.

Reporting on sex and gender	Clinical studies enrolled both male and female healthy volunteers.
Population characteristics	Clinical trial (phase 0, Australia):<br>For phase 0 study, 4 female subjects (50%) and 4 male subjects (50%) were enrolled into the study. Five subjects were white (62.5%); two were Asian (25%), one was not specified for race (12.5%). All 8 (100%) subjects are non-hispanic/Latino. The mean age was 28.8 years (range 18 to 42years), and the mean BMI was 24.93 kg/m2 (range 19.7 to 29.0 kg/m2).<br>Clinical trial (phase 1, New Zealand):

Part A (SAD):
For Part A, 22 female subjects (55.0%) and 18 male subjects (45.0%) were enrolled into the study. The majority of subjects were White (33 subjects [82.5%]) and not Hispanic or Latino (32 subjects [80.0%]). The mean age was 28.4 years (range 19.0 to 51.0 years), and the mean BMI was 24.95 kg/m2 (range 19.3 to 30.5 kg/m2).
Part B (MAD):
For Part B, 13 female subjects (54.2%) and 11 male subjects (45.8%) were enrolled into the study. The majority of subjects were White (19 subjects [79.2%]) and not Hispanic or Latino (21 subjects [87.5%]). The mean age was 25.8 years (range 18.0 to 50.0 years), and the mean BMI was 23.82 kg/m2 (range 18.7 to 30.1 kg/m2)
For Part C, 5 female subjects (35.7%) and 9 male subjects (64.3%) were enrolled into the study (total 14 subjects). The majority of subjects were White (12 subjects [85.7%]) and not Hispanic or Latino (10 subjects [71.4%]). The mean age was 29.1 years (range 19.0 to 48.0 years), and the mean BMI was 24.99 kg/m2 (range 19.9 to 31.1 kg/m2).
Clinical trial (phase 1, China):
Part A (SAD): 17 (70.8%) male and 7 (29.2%) female subjects were enrolled in the study; 23 (95.8%) subjects were of Han Chinese ethnicity; the mean age of the subjects in each dosage group (30 mg, 60 mg, 120 mg, and placebo) was 33.2 years old, 27.5 years old, 28.5 years old, 28.7 years old, and the mean values of weight, height, and body mass index (BMI) were 64.01 kg, 167.25 cm, and 22.79 kg/m2 , respectively, in all subjects.
Part B (MAD): 20 (83.3%) male and 4 (16.7%) female subjects were enrolled in the study; 22 (91.7%) subjects were of Han Chinese ethnicity; the mean age of subjects in each dosage group (30 mg, 60 mg, 90 mg, and placebo) was 31.8, 26.0, 25.3, and 27.8 years old, respectively; and the mean values of body weight, height, and body mass index (BMI) of all subjects were 63.06 kg, 167.79 cm, and 22.21 kg/m2 , respectively.

Recruitment

Healthy subjects were recruited according to pre-specified inclusion/exclusion criteria.
There is no known bias in selection of subjects and site.
Before entering the study, each trial participant was given a full explanation of the study to ensure the full understanding of the implications of participating the study before signing and giving a written informed consent which is in compliance with ICH E6(R2). The investigator submitted the informed consent form (ICF) to the IRB for review and approval before the initiation of the study.

Australia Phase 0:
Participants who met all of the following criteria at Screening were eligible to participate in the study:
1. Healthy female or male aged ≥ 18 and ≤ 55 years at Screening. Healthy was defined as no clinically relevant abnormalities identified by a detailed medical history, full physical examination, including blood pressure and pulse rate measurement, 12-lead electrocardiogram (ECG), and clinical laboratory tests.
2. BMI of 17.50 to 30.50 kg/m2; and a total body weight > 50 kg at Screening and Day -1.
3. Sufficient venous access for the purposes of the study.
4. Non-smoker. The participant must not have used any tobacco products within 2 months prior to Screening.
5. Females were non-pregnant and non-lactating, and agreed to use an acceptable, highly effective double contraception from Screening until study completion, including the Follow-up period. Double contraception was defined as a condom AND one other form of the following:

a. Established hormonal contraception (with approved long-acting implantable hormones, injectable hormones). Oral contraceptive pills [OCPs] could not be used as a second form of contraception by female participants, due to the unknown potential for OCP reduced effectiveness when administered in combination with INS018_055.
b. A vaginal ring or an intrauterine device (IUD) (including a hormonal IUD).
c. Documented evidence of surgical sterilisation at least 6 months prior to Screening (eg, tubal occlusion, hysterectomy, bilateral salpingectomy, or bilateral oophorectomy for women or vasectomy for men [with appropriate post-vasectomy documentation of the absence of sperm in semen] provided the male partner was a sole partner).

Women not of childbearing potential were post-menopausal for ≥ 12 months. Post-menopausal status was confirmed through testing of follicle-stimulating hormone (FSH) levels ≥ 40 IU/L at Screening for amenorrhoeic female participants.
Females who were abstinent from heterosexual intercourse were also eligible.
Periodic abstinence (eg, calendar, ovulation, symptothermal, post-ovulation methods) and withdrawal were not considered highly effective methods of birth control. Participant complete abstinence for the duration of the study and for 90 days after the last study treatment was acceptable.
Female participants who were in same-sex relationships were not required to use contraception.
Women of childbearing potential (WOCBP) had a negative pregnancy test at Screening and Day 1 and were willing to have additional pregnancy tests as required throughout the study.
Males were surgically sterile (> 30 days since vasectomy with no viable sperm), abstinent, or if engaged in sexual relations with a WOCBP, the participant and his partner were surgically sterile (eg, tubal occlusion, hysterectomy, bilateral salpingectomy, bilateral oophorectomy) or using an acceptable, highly effective contraceptive method from Screening until study completion, including the Follow-up period. Acceptable methods of contraception included the use of condoms and the use of an effective contraceptive for the female partner that included: OCPs, long-acting implantable hormones, injectable hormones, a vaginal ring, or an IUD. Participants with same-sex partners (abstinence from penile-vaginal intercourse) were eligible when this was their preferred and usual lifestyle.
Males could not donate sperm for at least 90 days after the last study treatment.
6. Willing and able to attend the trial visits and complete study assessments.
7. Willing to consume standard meals provided.
8. Able to read and understand study documents and follow Investigator and study personnel instructions during visits.
9. Able to sign the HREC approved ICF.

Exclusion Criteria

Participants who met any of the following criteria at Screening were not eligible to participate in the study:
1. Positive toxicology screening panel (urine test including qualitative identification of barbiturates, tetrahydrocannabinol [THC], amphetamines, benzodiazepines, opiates, and cocaine), or with a history of substance abuse or dependency or history

of recreational IV drug use over the last 5 years (by self-declaration).
2. Positive alcohol breath test at Screening or a history of regular alcohol consumption exceeding 14 drinks/week for women or 21 drinks/week for men (1 drink = 150 mL of wine or 360 mL of beer or 45 mL of hard liquor) within the 6 months prior to Screening.
3. Major surgery or significant trauma within 28 days (4 weeks) prior to Screening.
4. Blood pressure (BP) > 150 mmHg (systolic) or > 95 mmHg (diastolic) at Screening and Day -1, following at least 5 minutes of supine rest. If BP was > 140 mmHg (systolic) or > 90 mmHg (diastolic), the BP measurements was repeated 2 more times, at least 2 minutes apart, and the average of the 3 BP values was used to determine the participant's eligibility.
5. Heart rate < 45 beats per minute (bpm) or > 100 bpm at Screening and Day -1, following at least 5 minutes of supine rest. If heart rate was below 45 bpm or exceeds 100 bpm, the heart rate was repeated 2 more times, at least 2 minutes apart, and the average of the 3 heart rate values was used to determine the participant's eligibility.
6. 12-lead ECG demonstrating QTc > 450 msec for males or > 470 msec for females, or a QRS interval ≥ 120 msec at Screening and Day -1. If QTc exceeded 450 msec (males) or 470 msec (females), or QRS exceeded 120 msec, the ECG was repeated 2 more times, at least 2 minutes apart, and the average of the 3 QTc (or QRS) values was used to determine the participant's eligibility.
7. ANY of the following abnormalities in clinical laboratory tests at Screening, as assessed by the study-specific laboratory and confirmed by a single repeat, if deemed necessary: – Serum creatinine level above the upper limit of normal (ULN) or an estimated glomerular filtration rate value < 80 mL/min, based on the Cockcroft-Gault calculation, at Screening.
– Aspartate aminotransferase (AST) / serum glutamic oxaloacetic transaminase (SGOT) or alanine aminotransferase (ALT) / serum glutamic pyruvic transaminase (SGPT) > 1.5 × ULN.
– Fasting glucose > 5.4 mmol/L.
– Total bilirubin > 1.5 × ULN.

8. A white blood cell count < 4.0 × 109/L. Participants with borderline clinical laboratory values outside the reference range could be included in the study if the Investigator deemed that the values were not clinically significant.
9. Absolute neutrophil count of < 2 × 109/L.
10. Haematocrit below 0.4 for males and 0.35 for females.
11. Use of any IP or investigational medical device within 30 days prior to Screening, or 5 half-lives of the product (whichever was the longest) or participation in more than 4 investigational drug studies within 1 year prior to Screening.
12. Use of prescription or non-prescription drugs and dietary supplements within 7 days or 5 half-lives (whichever was longer) prior to INS018_055 administration, with the exception of paracetamol, which could be used at doses of ≤ 2 g/day, and contraceptives.
13. Blood donation (excluding plasma donations) of ≥ 500 mL or significant blood loss within 56 days prior to dosing.
14. History of sensitivity to heparin or heparin-induced thrombocytopenia.
15. Other severe acute or chronic medical or psychiatric condition including recent (within the past year) or active suicidal ideation or behaviour or laboratory abnormality, or any other abnormality that in the opinion of the Investigator could increase the risk associated with study participation or IP administration or could interfere with the interpretation of study results and make the participant inappropriate for entry into this study.
16. CRU staff members directly involved in the conduct of the study and their family members, CRU members otherwise supervised by the Investigator, or participants who were Sponsor employees including their family members directly involved in the conduct of the study.
17. Vaccination with live virus, attenuated live virus, or any live viral components within the 6 weeks prior to the first dose of study drug or was to receive these vaccines at any time during treatment or within 8 weeks following the end of study visit. Scheduled/intended to have a COVID-19 vaccine during the study (ie, from Screening through to Day 8).
18. History of any lymphoproliferative disorder (such as EBV related lymphoproliferative disorder, as reported in some participants on other immunosuppressive drugs), history of lymphoma, leukaemia, myeloproliferative disorders, multiple myeloma, or signs and symptoms suggestive of current lymphatic disease.
19. Clinically significant infection currently or within 6 months of first dose of study drug (those requiring hospitalisation or parenteral antimicrobial therapy or opportunistic infections), or a history of chronic or recurrent infectious disease.
20. Known infection with or test positive at Screening for HIV, hepatitis B or C viruses.
21. History of malignancy, except for non-melanoma skin cancer, excised more than 2 years ago, and cervical intraepithelial neoplasia that had been successfully cured more than 5 years prior to Screening.
22. Consumption of grapefruit or grapefruit juice or citrus fruits (ie, Seville oranges, pomelos, tangelos) within 7 days prior to the first dose of study medication until collection of the final pharmacokinetic blood sample.
23. History of severe allergic reactions (eg, anaphylaxis) or known sensitivity to any of the constituents of the test product.
24. Pregnant or lactating at Screening or planning to become pregnant (self or partner) at any time during the study, including the Follow-up period.
25. History of benign ethnic neutropenia.

Phase I Clinical Trial China:
Inclusion criteria:
Each subject must meet all of the following criteria to be enrolled in this study:
1. Subjects were male or female between 18 and 45 years of age, inclusive.
2. Subjects had a body mass index of 19 to 26 kg/m2 (inclusive) and weighed ≥ 50 kg (inclusive) for men and ≥ 45 kg (inclusive) for women at the time of screening.
3. Based on medical history, clinical laboratory findings, vital sign measurements, 12-lead ECG results, physical examination, chest radiograph results, and serum virological findings at screening, the investigators concluded that the subjects were in good general health.
4. Female subjects of childbearing potential must be non-pregnant and non- lactating and must be using one of the following methods of contraception throughout the treatment period until at least 28 days after the last dose of study drug, or have been surgically sterilized (i.e., hysterectomy, bilateral tubal ligation, or bilateral oophorectomy) or are postmenopausal (defined as 12 consecutive months of amenorrhea with documented plasma follicle stimulating hormone levels > 40 IU/mL). Female subjects must have a negative pregnancy test result at screening and prior to the first dose of study drug.
A highly effective method of contraception is one that has a contraceptive failure rate of less than 1% per year when used consistently. Examples are as follows:

a. Implanted contraceptives (e.g., Jadelle®)
b. IUDs containing copper or levonorgestrel (e.g., Mirena®)
c. Male sterilization, no sperm in ejaculation after vasectomy
d. Double barrier method: condom and occlusion cap (diaphragm or cervical cap/dome cap), barrier method with spermicide (foam/gel/film/cream/suppository) must be used as an add-on
e. Abstinence, defined as complete and continuous avoidance of all heterosexual sex (including during the entire period of risk associated with study treatment), was allowed without contraception only if this was the subject's preferred and daily lifestyle.
or an effective method with a contraceptive failure rate of less than 5% to 10% per year. Examples are as follows:
f. Injectable contraceptives (e.g., Depo Provera)
g. Oral contraceptives (combination hormonal contraceptives or progestogen-only "mini-pills")
h. Vaginal contraceptive ring (e.g., NuvaRing®)
Female subjects must also agree not to donate eggs from the time of administration until at least 28 days after the last dose of study drug.
Male subjects and their fertile female partners must agree to use one of the above methods of contraception for the entire treatment period until at least 28 days after the last dose of study drug. Male subjects must also agree not to donate sperm for the entire treatment period until at least 28 days after the last dose of study drug.
5. Subjects agree to comply with all protocol requirements.
6. Subjects were able to provide written informed consent.

Exclusion criteria:
Subjects who met any of the following criteria were excluded from the study:
1. Subject has current evidence or history of clinically significant hematologic, renal, endocrine, pulmonary, gastrointestinal, cardiovascular, hepatic, psychiatric, neurologic, or allergic disease (including drug allergy, but excluding asymptomatic seasonal allergy that was untreated at the time of administration).
2. Subjects have any condition that may affect drug absorption (e.g., gastrectomy).
3. Subjects had a history of cancer, except adequately treated basal cell or squamous cell carcinoma of the skin.
4. The subject rests for at least 5 minutes with a blood pressure (BP) > 140 mm Hg (systolic) or > 90 mm Hg (diastolic). At screening, if BP is > 140 mm Hg (systolic) or > 90 mm Hg (diastolic), BP should be measured 2 additional times and the average of the 3 BP values should be used to determine the subject's eligibility to participate.
5. At screening, the subject's 12-lead ECG shows a QT interval (QTc) corrected by the Bazett formula (QTc=QT/RR0.5) > 450 msec, or a QRS interval > 120 msec. The average of three QTc (or QRS interval) values from three standard 12-lead ECGs (repeated at intervals of no more than 5 minutes) should be used to determine the subject's eligibility for participation.
6. At the time of screening, the subject has any of the following abnormalities in clinical laboratory tests (if necessary, retest once for confirmation):
a. At screening, serum creatinine levels above the upper limit of normal (ULN) or creatinine clearance (Ccr) < 80 mL/min using the Cockcroft - Gault formula (Appendix 3) and no protein in the urine.
b. Aspartate aminotransferase or alanine aminotransferase values > 1.5 × ULN.
c. Fasting glucose > 110 mg/dL (6.1 mmol/L).
d. Total bilirubin > 1.5 × ULN.
e. Routine blood test values that are outside the normal reference range of local laboratory findings and are considered clinically significant by the investigator.
f. Positive fecal occult blood test result at screening or registration (day -1).
7. Subjects have a history of any disease that may have caused total bilirubin to be higher than ULN. Subjects whose clinical laboratory test values are not significantly outside the reference range may be enrolled in this study if the investigator does not consider the values to be clinically significant.
Note: In subjects with a history of Gilbert's syndrome, direct bilirubin may be measured, and if direct bilirubin < ULN, the subject is eligible for this study.
8. Subjects have any history of lymphoproliferative disease (such as Epstein Barr virus-associated lymphoproliferative disease as reported by some subjects receiving immunosuppressive drugs), lymphoma, leukemia, myeloproliferative disease, multiple myeloma, or signs and symptoms suggestive of current lymphatic disease.
9. Subjects have a history of relevant drug and/or food allergies (i.e., allergy to any study drug or excipient, or any severe food allergy that could result in inability to consume the standard diet of the clinical institution).
10. Subject has a current or clinically significant infection (e.g., an infection requiring hospitalization or parenteral antimicrobial therapy or the presence of an opportunistic infection within the past 6 months) or a history of chronic or recurrent infectious disease within the 6 months prior to the first dose of study drug.
11. Subject has other serious acute or chronic medical or psychiatric illness (including recent (within the past year) or active suicidal ideation or behavior or abnormal experimental results (which may increase the risk associated with study participation or experimental drug administration or may interfere with the interpretation of study results and, in the judgment of the investigator, may render the subject unsuitable for entry into this study).
12. Subjects have a history of symptomatic herpes zoster or herpes simplex, more than one episode of localized herpes zoster, or disseminated herpes zoster (single episode) present or within 12 weeks.
13. Subjects tested positive for hepatitis B surface antigen, hepatitis C virus antibody, or human immunodeficiency virus type 1 or 2 antibody at screening.
14. Subjects were pregnant or lactating females.
15. Subjects are men of childbearing potential who are unwilling or unable to use the contraceptive methods described in this protocol throughout the study period and for at least 28 days after the last dose of the experimental drug.
16. Subjects are unwilling or unable to comply with the lifestyle restrictions described in this protocol.
17. Subjects were smokers or had used nicotine or nicotine-containing products (e.g., snus, nicotine patches, nicotine chewing gum, simulated cigarettes, or inhalants) within 6 months prior to the first dose of the study drug.
18. Subjects tested positive for substance abuse or cotinine (indicating current active smoking) prior to the first dose of study drug.
19. Subjects have used any prescription or over-the-counter medication (except paracetamol [up to 2 g/day]), including herbal supplements, within 14 days prior to the first dose of study drug. Nutritional supplements are permitted provided that they are unlikely to interfere with the study results and that investigator consent has been obtained.
20. Subjects ingested grapefruit or grapefruit juice, limes or products containing limes (e.g., orange marmalade) or products

containing alcohol, caffeine or xanthines within 48 hours prior to the first dose of the study drug.
21. Subjects will be vaccinated with live virus, live attenuated virus, or any live viral component within 2 weeks prior to the first dose of study drug, or will receive these vaccines at any time during the study or within 8 weeks of study completion.
22. Subject tested positive for Severe Acute Respiratory Syndrome-associated Coronavirus 2 (SARS-CoV-2). Subject received the 2019 coronavirus disease (COVID-19) vaccine within 2 weeks prior to the first dose of study drug or is scheduled to receive the COVID-19 vaccine within 12 weeks of study drug administration, or tested positive for SARS-CoV-2 during screening or had COVID-19 symptoms within 4 weeks prior to Day -1.
23. Subjects who have undergone major trauma or major surgery within 4 weeks prior to screening or who are expected to require major surgery during the trial.
24. Subjects are at risk for bleeding: genetic predisposition to bleeding, a bleeding event within 12 months prior to screening start, or abnormal laboratory coagulation parameters.
25. Subjects have a first-degree relative with a genetic immunodeficiency.
26. Subjects were study site staff and their family members who were directly involved in the implementation of the study, study site staff who were otherwise supervised by the investigator, or sponsor employees (including their family members) who were directly involved in the implementation of the study.
27. Subject has a history of alcohol abuse or drug addiction or excessive alcohol consumption (regular alcohol intake > 21 units/week for male subjects and > 14 units/week for female subjects; 1 unit equals approximately ½ pint [200 mL] of beer, 1 small glass [100 mL] of wine, or 1 cup [25 mL] of spirits) within the past year or consumed alcohol 24 hours prior to the first dose of study drug.
28. Subjects engaged in strenuous activity or contact sports within 24 hours prior to dosing and during the study.
29. The subject donated > 450 mL of blood or blood products within 30 days prior to the first dose of the study drug.
30. Subjects received study drug in another pilot study within 30 days prior to dosing or 5 drug half-lives, whichever is longer.
31. Subjects received cytochrome P450 (CYP3A4 and CYP2C8) and P-gp inhibitors and/or inducers within 4 weeks prior to the first dose of INS018_055 or may have received CYP3A4 and CYP2C8 and P-gp inhibitors and/or inducers during the study.
32. Subjects were deemed by the investigator to be unsuitable for enrollment in the study.

Phase I Clinical Trial New Zealand:
Inclusion criteria:
Each subject must meet all of the following criteria to be enrolled in this study:
1. The subject is a male or female 18 to 55 years of age, inclusive.
2. The subject has a body mass index 18 to 32 kg/m2, inclusive, and a total body weight ≥50 kg, inclusive, at screening.
3. The subject is considered by the investigator to be in good general health as determined by medical history, clinical laboratory test results, vital sign measurements, 12-lead ECG results, and physical examination findings at screening.
4. Female subjects of childbearing potential must be non-pregnant and non-lactating and must use one of the methods of contraception listed below for the duration of the treatment until at least 28 days after the last dose of the study drug, or be surgically sterile (ie, hysterectomy, bilateral tubal ligation, or bilateral oophorectomy) or postmenopausal (defined as amenorrhea 12 consecutive months and documented plasma follicle-stimulating hormone level >40 IU/mL). Female subjects must have a negative pregnancy test at screening and before the first dose of study drug.

Highly effective methods of contraception are those that result in a failure rate of less than 1% per year when used consistently. Examples are provided below:
a. Implant contraceptive (eg, Jadelle®)
b. Intrauterine device (IUD) containing either copper or levonorgestrel (eg, Mirena®)
c. Male sterilization with absence of sperm in the post-vasectomy ejaculate

OR an effective method that results in a failure rate of less than 5% to 10% per year. Examples are provided below:
d. Injectable contraceptive (eg, Depo Provera)
e. Oral contraceptive pill (combined hormonal contraceptive pill or progestogen-only 'mini-pill')

f. Vaginal contraceptive ring (eg, NuvaRing®)
Female subjects must also agree not to donate eggs, from dosing until at least 28 days after the last dose of study drug.
A male subject and his female partner who is of childbearing potential must agree to use one of the methods of contraception listed above for the duration of the treatment until at least 28 days after the last dose of the study drug. A male subject must also agree not to donate sperm, for the duration of the treatment until at least 28 days after the last dose of the study drug.
5. The subject agrees to comply with all protocol requirements.
6. The subject is able to provide written informed consent.
Inclusion criteria:

1. The subject has current evidence or history of clinically significant hematological, renal, endocrine, pulmonary, GI, cardiovascular, hepatic, psychiatric, neurologic, or allergic disease (including drug allergies, but excluding untreated, asymptomatic, seasonal allergies at time of dosing).
2. The subject has any condition possibly affecting drug absorption (eg, gastrectomy).
3. The subject has a history of cancer with the exception of adequately treated basal cell or squamous cell carcinoma of the skin.
4. The subject has supine blood pressure (BP) >140 mm Hg (systolic) or >90 mm Hg (diastolic), following at least 5 minutes of supine rest. If BP is >140 mm Hg (systolic) or >90 mm Hg (diastolic), the BP should be repeated 2 more times and the average of the 3 BP values should be used to determine the subject's eligibility at screening.
5. The subject has 12-lead ECG demonstrating corrected QT interval by Fridericia (QTcF) >450 msec, or a QRS interval >120 msec at screening. If QTcF exceeds 450 msec, or QRS interval exceeds 120 msec, the ECG should be repeated 2 more times and the average of the 3 QTcF (or QRS interval) values should be used to determine the subject's eligibility.
6. The subject has ANY of the following abnormalities in clinical laboratory tests at screening, as assessed by the study-specific laboratory and confirmed by a single repeat, if deemed necessary:
a. Serum creatinine (SCr) level above the upper limit of normal (ULN) or an estimated glomerular filtration rate (GFR) value <80 mL/min/1.73 m2 calculated with the Chronic Kidney Disease Epidemiology Collaboration (CKD-EPI) formula and the absence of protein in urine, at screening.

b. Aspartate aminotransferase (AST) or alanine aminotransferase (ALT) values more than >1.5 × ULN.
c. Fasting glucose >110 mg/dL (6.1 mmol/L).
d. Total bilirubin >1.5 × ULN.
e. Hematological values outside the normal reference range for local laboratory results.
f. Positive fecal occult blood test at screening or at check-in (Day -1).
7. The subject has any medical history of disease that has the potential to cause a rise in total bilirubin over the ULN. Subjects with borderline clinical laboratory values outside the reference range may be included in the study if the investigator deems that the values are not clinically significant.
Note: Subjects with a history of Gilbert's syndrome may have a direct bilirubin measured and would be eligible for this study provided the direct bilirubin is <ULN.
8. The subject has a history of any lymphoproliferative disorder (such as Epstein Barr Virus-related lymphoproliferative disorder, as reported in some subjects on immunosuppressive drugs), history of lymphoma, leukemia, myeloproliferative disorders, multiple myeloma, or signs and symptoms suggestive of current lymphatic disease.
9. The subject has a history of relevant drug and/or food allergies (ie, allergy to any study drug or excipients, or any significant food allergy that could preclude a standard diet in the clinical unit).
10. The subject has a clinically significant infection currently or within 6 months of first dose of study drug (eg, those requiring hospitalization or parenteral antimicrobial therapy or opportunistic infections), or a history of chronic or recurrent infectious disease.
11. The subject has other severe acute or chronic medical or psychiatric condition including recent (within the past year) or active suicidal ideation or behavior or laboratory abnormality that may increase the risk associated with study participation or investigational product administration or may interfere with the interpretation of study results and, in the judgment of the investigator, would make the subject inappropriate for entry into this study.
12. The subject has or has had symptomatic herpes zoster or herpes simplex within 12 weeks, more than one episode of local herpes zoster, or a history (single episode) of disseminated zoster.
13. The subject has a positive test result for hepatitis B surface antigen, hepatitis C virus antibody, or human immunodeficiency virus (HIV) types 1 or 2 antibodies at screening.
14. The subject is a female who is pregnant or lactating.
15. The subject is a fertile male who is unwilling or unable to use a highly effective method of contraception as outlined in this protocol for the duration of the study and for at least 28 days after the last dose of investigational product.
16. The subject is unwilling or unable to comply with the lifestyle restrictions described in this protocol (Section 4.3.1).
17. The subject is a smoker or has used nicotine or nicotine-containing products (eg, snuff, nicotine patch, nicotine chewing gum, mock cigarettes, or inhalers) within 6 months before the first dose of study drug.
18. The subject has a positive test result for drugs of abuse or cotinine (indicating active current smoking) at screening or before the first dose of study drug.
19. The subject has used any prescription or over-the-counter medications (except paracetamol [up to 2 g per day]), including herbal supplements, within 14 days before the first dose of study drug. Nutritional supplements are allowed if unlikely to interfere with the study results and agreed by medical monitor and investigator.
20. The subject has consumed grapefruit or grapefruit juice, Seville orange or Seville orange-containing products (eg, marmalade), or alcohol-, caffeine-, or xanthine-containing products within 48 hours before the first dose of study drug.
21. The subject has used a known strong or moderate inhibitor or inducer of CYP1A2 within 4 weeks prior to Day 1 and through the last PK sampling point on Day 21 (only for Part C, DDI).
22. The subject will have vaccination with live virus, attenuated live virus, or any live viral components within the 2 weeks prior to the first dose of study drug or is to receive these vaccines at any time during treatment or within 8 weeks following completion of study treatment.
23. The subject has a positive test result for severe acute respiratory syndrome-related coronavirus 2 (SARS-CoV-2). The subject has received the Coronavirus disease 2019 (COVID-19) vaccine within 2 weeks prior to the first dose of study drug or plans to receive a COVID-19 vaccine within 12 weeks after study drug dosing or has positive test for SARS-CoV-2 during screening or presence of COVID-19 symptoms within 4 weeks prior to Day -1.
24. The subject has undergone significant trauma or major surgery within 4 weeks of screening.

Ethics oversight

Phase 0 Clinical Trial Australia:
Institution: CMAX Clinical Research Center, 18A North Terrace, Adelaide SA 5000, Australia
A phase 0 clinical trial, also being the first-in-human clinical trial of INS018_055, was conducted in Australia (ACTRN12621001541897). This trial was conducted in accordance with the ethical principles of Good Clinical Practice, according to the International Council for Harmonisation (ICH) Harmonised Guideline E6(R2) Integrated Addendum to ICH E6(R1): Guideline for Good Clinical Practice ICH E6(R2), annotated with comments by the Australian Therapeutic Goods Administration (TGA; 2018). This study was approved by Bellberry Human Research Ethics Committee.

Phase I Clinical Trial China:
Institution: Zhejiang Xiaoshan Hospital, 728 Yucai N Rd, Xiaoshan District, Hangzhou, Zhejiang, China, 311202
Detailed information including facilities, inclusion/exclusion criteria could be found in link http://www.chinadrugtrials.org.cn/clinicaltrials.prosearch.dhtml (registration number: CTR20221542). This study was approved by Zhejiang Xiaoshan hospital Clinical trial ethics committee. The number of ethics committee approval letter is EC-2022102505.

Phase I Clinical Trial New Zealand:
Institution: New Zealand Clinical Research Center, Christchurch, New Zealand, Level 4, 264 Antigua Street, Central city , Christchurch, 8011 New Zealand
(registration number: NCT05154240).This study was approved by Northern B Health and Disability Ethics Committee. Ethics reference number for this protocol is 2021 FULL 11770.

Note that full information on the approval of the study protocol must also be provided in the manuscript.

Field-specific reporting

Please select the one below that is the best fit for your research. If you are not sure, read the appropriate sections before making your selection.

☒ Life sciences ☐ Behavioural & social sciences ☐ Ecological, evolutionary & environmental sciences

For a reference copy of the document with all sections, see nature.com/documents/nr-reporting-summary-flat.pdf

Life sciences study design

All studies must disclose on these points even when the disclosure is negative.

Sample size	Clinical (Australia): A total of 8 healthy adult subjects were enrolled. On day -1, baseline data was taken and safety were evaluated. On day 1, A single dose of 100 µg INS018_055 was given to each subject through IV injection. Blood was collected pre-dose and 5, 15, 30 minutes, then 1, 2, 4, 8 and 24 hours post-dose for PK evaluation. After dosing, individuals were monitored for safety until discharged in Day 2. The number of participants was selected to allow for evaluation of safety/tolerability and PK in this study and is consistent with standards of practice for Phase 0 studies.<br><br>Clinical (New Zealand): 40 and 24 subjects were enrolled in Part A and Part B, respectively. Among 8 subjects per cohort, 6 subjects received treatment matching with 2 subjects received placebo (3:1). The determination of the subject number was not based on a formal statistical power calculation but based on clinical and practical considerations.<br><br>Clinical (China): 24 and 24 subjects were enrolled in Part A and Part B, respectively. Among 8 subjects per cohort, 6 subjects received treatment matching with 2 subjects received placebo (3:1). The number of subjects was determined based on clinical and practical considerations, rather than on formal statistical certainty calculations. The total sample size of 56 subjects was considered sufficient for the purpose of the study based off of prior similar studies. The final enrollment is 48 since the trial is terminated due to COVID-9 outbreak.<br><br>Animal study:<br>For lung and kidney in vivo studies receiving oral dosing of INS018_055, the sample size in most groups are between 8-10. In some cases, when there were satellite groups receiving same treatment but for PK collection, some data (lung function, clinical observation, etc) were pooled together with efficacy groups.<br>For the inhalation study, the sample sizes of each group are 12/group for vehicle and SOC group and 9/group for INS018_055 group with additional 3/group for PK collection.<br>For skin fibrosis study, the sample size is 5/group.<br>The determination on the sample sizes in in vivo study is based on the expected variations in the disease model and 3R principles (Replacement, Reduction and Refinement).<br>In vitro: no special considerations.
Data exclusions	Clinical:<br>There are no data exclusion except in below cases:<br>In New Zealand phase 1 trial, one participant (101-066) discontinued treatment and was subsequently discontinued from the study due to a TEAE of influenza-like illness.<br>Additionally in the New Zealand phase 1 trial, a few PK timepoints were referred to as 0 and not included in the study based on the pre established SAP that "BLQ value between 2 quantifiable concentrations will be set as missing."<br><br>Animal Studies: No animals were excluded from this study unless noted in the supplementary information wherein mice were sacrificed early due to health concerns that are explicitly outlined in the methods section. This was less than 5 mice throughout the entire study which were sacrificed for humane reasons prior to endpoint data collection.
Replication	For patient number in clinical study, and sample size (animal number/group), the information could be found in manuscript, or the section of Life sciences study design-Sample size in this reporting summary file.<br>Each clinical study and animal study were performed once. Two phase 1 trials were performed in different dosing regimen. Regarding preclinical animal studies, the efficacy of test compound was validated three times in bleomycin induced lung fibrosis models, though in different study designs.<br>In vitro assays<br>Assays on MRC-5, and A549, treated with INS018_055 were performed in 3 independent replicates.<br>Assays on A549, knocked-by different shRNA targeting against TNIK were performed once, respectively: (Once by shTNIK-4 and once by shTNIK-1 with different virus dilution ratio). The results of these assays are consistent.<br>Assay on HK-2 was performed once.<br>EMT and FMT assays on primary lung cells were formed in 6 donors (3 healthy and 3 IPF patients), respectively.<br>Assays performed on NHDF were performed in 5 replicates.<br>For cell viability assays, A549 and HK2, once for each cell line. Cell viability on MRC-5 was performed twice. Data of the other assay is not shown in manuscript, the conclusion is similar, with CC50>100 µM.
Randomization	Clinical trials:<br>For phase 0 study, there was no randomization and the study only has one cohort.<br>For Phase I NZ and Phase I China trials, enrolled subjects were randomly assigned within each dose cohort to receive INS018_055 or a matched placebo.<br>For all animal models utilized in this study, individual animals were randomly assigned to their respective groups.

Blinding

For all animal studies, the animals were randomized when grouping. The dosing and analysis were semi-blinded where different investigators experimentally handled the mice during model-induction (BLM, UUO, Skin fibrosis) while data analysis was performed by other investigators. This semi-blinded approach was required in order to assign animals to the correct grouping following disease-model induction since experimental animals that were not in the sham cohorts were visibly different to the sham mice but macroscopically similar across experimental groups.
In addition in UUO experiment, experimenters stated that during histological analysis, the tissue slides were coded a number for blind evaluation. Each number was generated using the RAND function of Excel software, sorted in ascending order and assigned the slides. The tissue slides were used for the following stains and evaluated by an experimenter.
Clinical trial (phase 0, Australia): Blinding was not used in phase 0 study, since it was a single cohort study.
Clinical trial (Phase 1, New Zealand): Part A and B of this clinical trial is a double-blind study design. The INS018_055 and matching placebo capsules were identical in appearance. The unblinded pharmacist was responsible for dispensing the study drug in capsules with identical appearance between INS018_055 and placebo, in a manner consistent with maintaining the blind. Reviews of unblinded safety data were performed to determine whether to move towards next dose cohort.
Blinding were maintained during dosing, sample collection and analysis.
Clinical trial (Phase1, China): INS018_055 has identical appearance to matched placebo capsules. The pharmacist was responsible for responsibility for dispensing the test drug in a manner that maintains blinding at all times.Blinding was maintained throughout the study period. If a subject becomes seriously ill or pregnant during the study period, blinding will be done only when it is known that administration of the test drug will affect the available treatment options for that subject. In the event of a medical emergency requiring identification of the individual subject receiving the test drug, the investigator should make every effort to contact the Medical Monitor within 24 hours prior to code unblinding to explain the need for code unblinding. The investigator is responsible for documenting the time, date, and reason for code unblinding, as well as the names of the participants.
(Detailed procedures could be found in protocols)

Reporting for specific materials, systems and methods

We require information from authors about some types of materials, experimental systems and methods used in many studies. Here, indicate whether each material, system or method listed is relevant to your study. If you are not sure if a list item applies to your research, read the appropriate section before selecting a response.

Materials & experimental systems

n/a	Involved in the study
☐	☒ Antibodies
☐	☒ Eukaryotic cell lines
☒	☐ Palaeontology and archaeology
☐	☒ Animals and other organisms
☐	☒ Clinical data
☒	☐ Dual use research of concern

Methods

n/a	Involved in the study
☒	☐ ChIP-seq
☒	☐ Flow cytometry
☒	☐ MRI-based neuroimaging

Antibodies

Antibodies used

Antibodies:
Western Blot: Primary antibodies: Antibody name Vendor Catalog # /Lot and dilution factor: Anti-human-fibronectin, Invitrogen, MA5-11981 (1:500); Anti-human-E-cadherin, BD Biosciences, 610182 (1:1000); Anti-human-N-cadherin (D4R1H) , Cell Signaling Technology (CST), 13116S (1:1000); Anti-human-phospho-smad2 (ser465/467)/smad3(ser423/425)(D27F4), CST, 8828S (1:1000); Anti-human-phospho-smad2 (ser465/467), CST, 3108S; Anti-human-smad2/3 (D7G7), CST, 12470S (1:1000); Anti-human-phospho-fak (tyr397) (D20B1) , CCST, 8556 (1:1000); Anti--human-FAK, CST, 3285 (1:1000); Anti--human-TNIK, CST, 32712 (1:1000); Anti-human-NF-κB p65 (D14E12), CST, 8242S (1:1000); Anti-human-Phospho-NF-κB p65 (Ser536) (93H1) (1:1000); CST, 3033S, Anti-human-beta-catenin, CST, 9562 (1:1000); Anti-human-Alpha-tubulin, Abcam, ab18251 (1:10000); Anti-human-HDAC2, Abcam, ab12169 (1:1000); Anti-human-Histone 3, Abcam, ab176842 (1:1000); anti-α-SMA antibodies (CST, #19245, 1:1000; #ab5694 and Santa Cruz, sc-56499, 1:500); GAPDH (CST, #2118S, 1:10000; Merk Millipore, MAB374, 1:2000).Secondary antibodies: goat Anti-Rabbit IgG(H+L), Abclonal, Lot 9300014001, 1: 8000; goat Anti-mouse IgG H&L, abcam, ab205719, 1:5000 or 20000; goat Anti-rabbit IgG H&L, abcam, ab205718, 1:20000)
Immunohistochemistry (IHC): Primary antibodies: anti-aSMA (Abcam, #ab5694, 1:400); Collagen I (Abcam, ab34710, 1:400; LSL co., LTD. cat#LB-1102, 1:2000). Secondary antibodies: goat anti-rabbit IgG HRP, DAKO, K4003, 10150251; Goat anti-rabbit IgG HRP, Vector Laboratories, Inc., Cat #PI-1000)
Immuno flurescence (IF): Anti-aSMA-Alexa Fluor 488 antibody (Abcam, Cat# ab184675, 1:600); anti-aSMA, Sigma-Aldrich, 1:100; anti-fibronectin 1 antibody (biohit healthcare, cat# 610001), 0.125 ug/mL in blocking buffer, DAPI (Invitrogen, Cat# D3571, 500 ng/mL) Secondary antibodies: Donkey anti-mouse Alexa 546 (Invitrogen, cat# A10036).
ELISA kits: IL-1β, SINOBEST BIO, YX-E01291M; IL-4, SINOBEST BIO, YX-E00064M; IL-6, SINOBEST BIO, YX-E00066M; TNF-α, SINOBEST BIO, YX-E00104M; Fibronectin: Takara, cat# MK115 ; Procollagen type I C-peptide (PIP): Takara, cat# MK101 ; rat hydroxyproline (kinesisDx, K11-0512) ; collagen (Abcam, ab222942)

Validation

Validations of antibodies and assay kits are provided by manufacturer.

Eukaryotic cell lines

Policy information about cell lines and Sex and Gender in Research

Cell line source(s)	Human fetal lung fibroblast cell line MRC-5 was purchased from ATCC (Cat#CCL-171) (cell viability assay performed in WuXi AppTec) and Shanghai Cell Bank, Chinese Academy of Sciences (α-SMA assay performed in BOJI)<br>Human primary bronchial epithelial cells were derived from 3 IPF donors (IPF05, IPF06, and IPF08) and 3 healthy donors (Br285, Br311 and 410955).<br>Human primary lung fibroblasts were derived from 3 IPF donors (IPF05, IPF06, IPF08) and 3 healthy donors (FB218, 03HF67101, FB2382).<br>Human lung adenocarcinoma cell line A549 was purchased from ATCC (Cat#CCL-185).<br>Human embryonic kidney 293T/17 was purchased from ATCC (Cat#CRL-11268).<br>Human kidney cell line HK-2 was purchased from ATCC (Cat#CRL-2190).<br>Normal human dermal fibroblasts (NHDF), Bioalternatives reference PF2
Authentication	No independent authentication of cell lines is available. The internal authentication of depends on the internal procedure of Contract research organizations. Assays using A549, 293T/17 and HK-2: WuXi AppTec (China); Assays using human primary bronchial epithelial cells and human primary lung fibroblasts: Charles Rivers (Netherlands); Assays using MRC-5: WuXi AppTec (China) and Guangzhou Boji Medical Biotechnological (China); Assays using NHDF: Bioalternatives (France)
Mycoplasma contamination	All cell lines used were checked and cleared for mycoplasma contimation at the following contract research organizations: WuXi AppTec (China), Charles Rivers (Netherlands), Guangzhou Boji Medical Biotechnological (China), Bioalternatives (France).
Commonly misidentified lines<br>(See ICLAC register)	No

Animals and other research organisms

Policy information about studies involving animals; ARRIVE guidelines recommended for reporting animal research, and Sex and Gender in Research

Laboratory animals	Studies performed on bleomycin-induced lung fibrosis mouse model: 7-8 weeks, C57BL/6 mice<br>The study performed on bleomycin-induced lung fibrosis rat model: 8-9 weeks, Sprague-Dawley(SD) Rat<br>Study performed on lipopolysaccharide (LPS)-induced acute lung injury mouse model: 8 weeks, C57BL/6 mice<br>The study on bleomycin induced skin fibrosis rat model: 5-6 week, SD rats<br>The study performed on unilateral ureteral obstruction(UUO) model: 7 weeks, C57BL/6 mice
Wild animals	No wild animals were used in this manuscript.
Reporting on sex	Studies performed on bleomycin-induced lung fibrosis mouse model: male<br>The study performed on bleomycin-induced lung fibrosis rat model: male<br>Study performed on LPS-induced acute lung injury mouse model: male<br>The study on bleomycin induced skin fibrosis rat model: male<br>The study performed on unilateral ureteral obstruction(UUO) model: female
Field-collected samples	No Field-collected samples were used in this manuscript.
Ethics oversight	All animal studies were ethically and humanely conducted following IACUC, IRB or relevant animal handling ethics organizational guidelines by our partnering CROs. Murine LPS and BLM studies were carried out by HD Biosciences (Shanghai) which were approved under the following AUF protocol numbers: AUF#146 and AUF#117. These AUF protocols were approved by the Institutional Animal Care and Use Committee at HD Biosciences and the studies were conducted at an AAALAC-accredited facilities. The BLM fibrosis study in rats that were administered INS018_055 as an inhalable agent was carried out by JOINN Laboratories (Suzhou). This study's IACUC approved procedural number is S-ACU22-1144. JOINN Laboratories is fully accredited by the AAALAC, and the study number provided adheres to the regulations and rules laid out by the Institutional Animal Care and Use Committee. The UUO Kidney study was performed by SMC Laboratories, Inc. (Japan). The Animal Care and Use Committee-approved protocol number is U32. All animals used in this study were housed and cared for in accordance with the Japanese Pharmacological Society Guidelines for Animal Use. The skin fibrosis model was carried out by TheraIndx LifeSciences PVT LTD (India). This study was performed using protocols approved by the Institutional Animals Ethics Committee (IAEC) of the test facility which was designed under CPCSEA guidelines for animal care. The registration number for this protocol is (No. 1852/PO/Rc/S/16/CPCSEA. For all the aforementioned studies, animals were group-housed (<5 per cage) in a temperature-controlled facilities (20-26 C) with 12h light/12h dark light cycles. All animals had ad libitum access to drinking food and water.

Note that full information on the approval of the study protocol must also be provided in the manuscript.

Clinical data

Policy information about clinical studies

All manuscripts should comply with the ICMJE guidelines for publication of clinical research and a completed CONSORT checklist must be included with all submissions.

Clinical trial registration	Clinical Trial Phase 0 (Australia): ACTRN12621001541897<br>Clinical trial (New Zealand): NCT05154240

Clinical trial (China): CTR20221542

Study protocol

Trial information has also been provided for all 3 trials in Supplementary Information 9,10, and 11, respectively.

Phase 0 Study (Australia)
A phase 0 micro dosing clinical trial was conducted in Australia (ACTRN12621001541897). The trial can be found using the clinical trial number using this database https://www.anzctr.org.au/TrialSearch.aspx

Summary: A total of 8 healthy adult subjects were enrolled. On day -1, baseline data was taken and safety were evaluated. On day 1, A single dose of 100 μg INS018_055 was given to each subject through IV injection. Blood was collected pre-dose and 5, 15, 30 minutes, then 1, 2, 4, 8 and 24 hours post-dose for PK evaluation. After dosing, individuals were monitored for safety until discharged in Day 2.

Phase I study (New Zealand):
Full study protocol of clinical trial (NCT05154240) can be found in www.clinicaltrials.gov.

Summary:This is a phase I, randomized, double-blind, placebo-controlled, oral single and multiple ascending doses, parallel group and exploratory drug-drug interaction study to evaluate the safety, tolerability, pharmacokinetics, and interaction potential of INS018_055 in healthy subjects, conducted in one clinical site in New Zealand. Protocol and its amendments were approved by the IRB before implementation unless proceeding with the changes was in the subject's best interest. Planned enrollment was a total of 80 healthy male and female subjects (40, 24, 16 subjects in Part A, B and C, respectively), aged from 18 to 55 years, with body mass index (BMI) of from 18 to 32 kg/m2, and a total body weight ≥50 kg, considered by the investigator to be in good general health based at screening.

Phase I study (China):
Full study protocol can be found at http://www.chinadrugtrials.org.cn/clinicaltrials.prosearch.dhtml (registration number: CTR20221542).

Summary:

This is a phase I, randomized, 2-part (Part A and Part B), double-blind, placebo-controlled, dose-escalation study designed to evaluate the safety, tolerability, and PK characteristics of healthy subjects receiving oral administration of INS018_055.
The study will include a screening period, enrollment, treatment period, and end-of-study (EOS) visit.

Data collection

Clinical Trial (Austrialia): A phase 0 clinical trial, also being the first-in-human clinical trial of INS018_055, was conducted in Australia (ACTRN12621001541897). This trial was conducted in accordance with the ethical principles of Good Clinical Practice, according to the International Council for Harmonisation (ICH) Harmonised Guideline E6(R2) Integrated Addendum to ICH E6(R1): Guideline for Good Clinical Practice ICH E6(R2), annotated with comments by the Australian Therapeutic Goods Administration (TGA; 2018). This study was approved by Bellberry Human Research Ethics Committee and data collected at CMAX Clinical Research Center, 18A North Terrace, Adelaide SA 5000, Australia.

Clinical trial (New Zealand): This study was licensed as registration number: NCT05154240.This study was approved by Northern B Health and Disability Ethics Committee and the Ethics reference number for this protocol is 2021 FULL 11770. The phase 1 clinical trial was conducted atN ew Zealand Clinical Research Center, Christchurch, New Zealand, Level 4, 264 Antigua Street, Central city , Christchurch, 8011 New Zealand. The first subject first dose was in Feb 21st, 2022, and last subject contact was in Sep 30th, 2022.

Clinical trial (China): The phase 1 clinical trial was conducted at Zhejiang Xiaoshan Hospital, 728 Yucai N Rd, Xiaoshan District, Hangzhou, Zhejiang, China, 311202. The first subject signed ICF: in Jul 22nd, 2022, and last subject visit was in Jan 13th, 2023.

Information on the exact data collected are outlined in the relevant sections of this reporting summary and have been included in the manuscript as part of the supplementary information for the referenced clinical trials.

Outcomes

Phase 0 Clinical Trial (Australia):
Primary Objective:
The primary objective of the study was to determine the PK of plasma INS018_055 after a single IV microdose administered to healthy participants.
Primary endpoints:
The primary endpoints of this study were:
•Volume of distribution of INS018_055 in healthy participants.
•Elimination half-life (t1/2) of INS018_055.
•Clearance of INS018_055 in healthy participants.

Phase 1 Clinical trial (New Zealand):
•The primary objective of this study was to assess the safety and tolerability of single and multiple oral escalating doses of INS018_055 administered to healthy subjects. The secondary objectives of this study were as follows:

• To determine the pharmacokinetics (PK) of INS018_055 following single and multiple oral escalating doses in healthy subjects.
• To assess the effect of food on the PK of INS018_055 following an oral dose.
The exploratory objectives of this study were as follows:
• To determine the effect of single and multiple oral escalating doses of INS018_055 on the circulating cluster of differentiation (CD)4+ and CD8+ subpopulation of T cells (as a measure of pharmacological activity) in healthy subjects.
• To determine the effect of multiple oral escalating doses of INS018_055 on the levels of phospho-nuclear factor kappa B (NF-κB) p65 (Ser536), phospho-SMAD family member (Smad)2 (Ser465/467), interleukin (IL)-6, transforming growth factor beta (TGF-β), matrix metalloproteinase (MMP)-2, MMP-7, MMP-9, total NF-κB p65, and total Smad2 in healthy subjects. The results of these exploratory objective analyses will be presented separately from the clinical study report (CSR).
• To assess the interaction potential of INS018_055 with a cytochrome P450 (CYP)1A2 substrate (caffeine).
• To characterize the metabolite profiles of INS018_055 in plasma and urine following the highest dose level in the multiple ascending dose (MAD) study part (Cohort 8 in Part B). The results of these exploratory objective analyses will be presented separately from the CSR.

Phase 1 Clinical trial (China):
The primary objectives of this study are:
• The primary objective of this study is to assess the safety and tolerability of single and multiple oral ascending doses of INS018_055 administered to healthy subjects
The secondary objectives of this study were:
• To determine the pharmacokinetic (PK) profile of INS018_055 following single and multiple oral escalating doses in healthy subjects.
• Assessment of the effect of single and multiple dose escalation oral administration of INS018_055 on biomarkers (T cell CD4+ and CD8+ subpopulations, interleukin [IL]-6, transforming growth factor [TGF]-β, matrix metalloproteinases [MMP-2, MMP-9, MMP-7]) in healthy subjects (as a measure of pharmacological activity)
