# Supplementary material for: A small-molecule TNIK inhibitor targets fibrosis in preclinical and clinical models
Source: Nat Biotechnol. 2024 Mar 8;43(1):63–75. doi: 10.1038/s41587-024-02143-0 (PMC11738990; doi:10.1038/s41587-024-02143-0)
Supplement: Supplementary file 4 — Unprocessed western blots for Figs. 2 and 5 and Extended Data Figs. 5 and 6. [file 41587_2024_2143_MOESM4_ESM.pdf]

Insilico Medicine Restricted (Level 3)

Figure 2C MRC-5

Well set -1

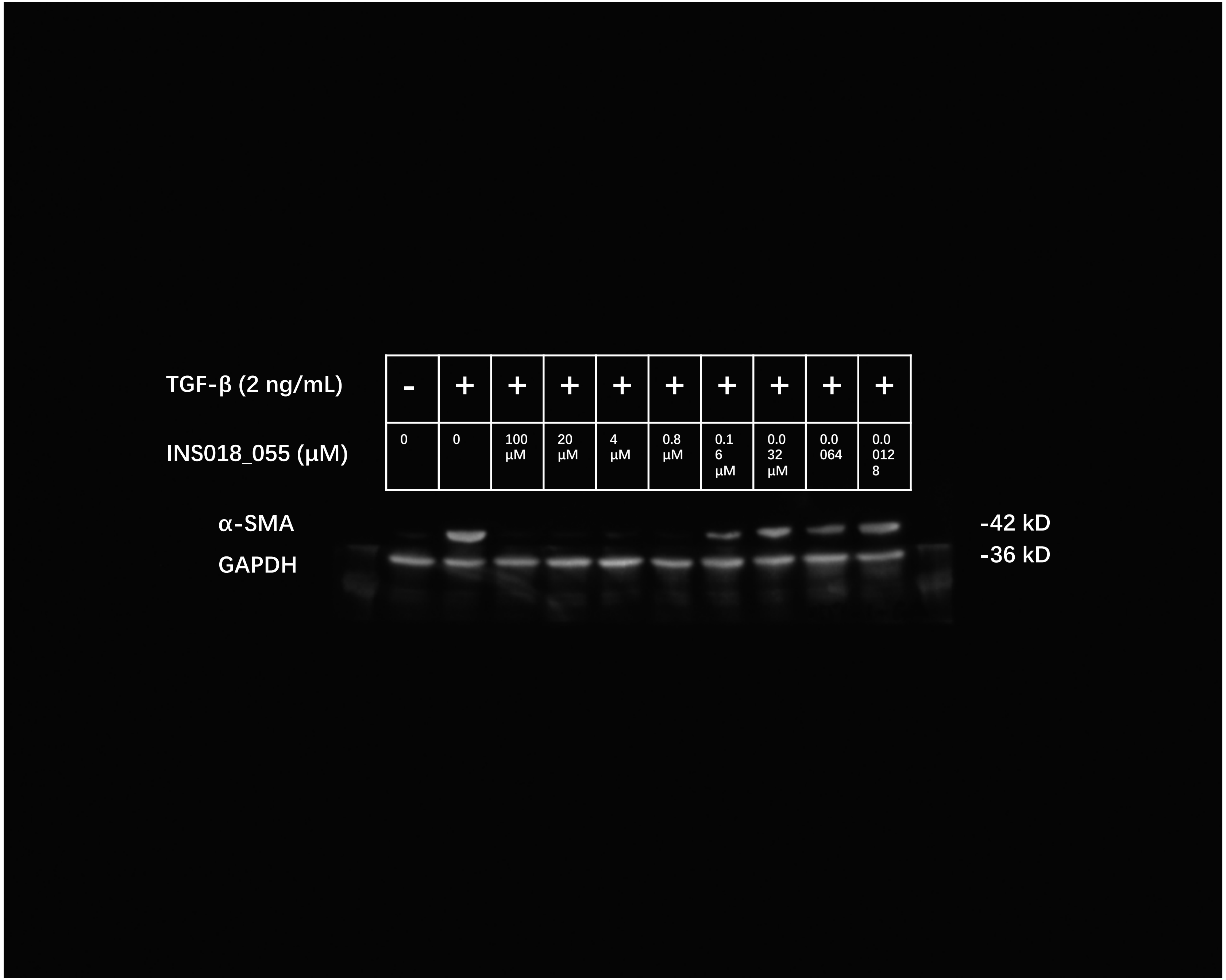

Image shapes reflect membranes cut for multiple staining panels

Insilico Medicine Restricted (Level 3)

Figure 2C MRC-5

Well set -2

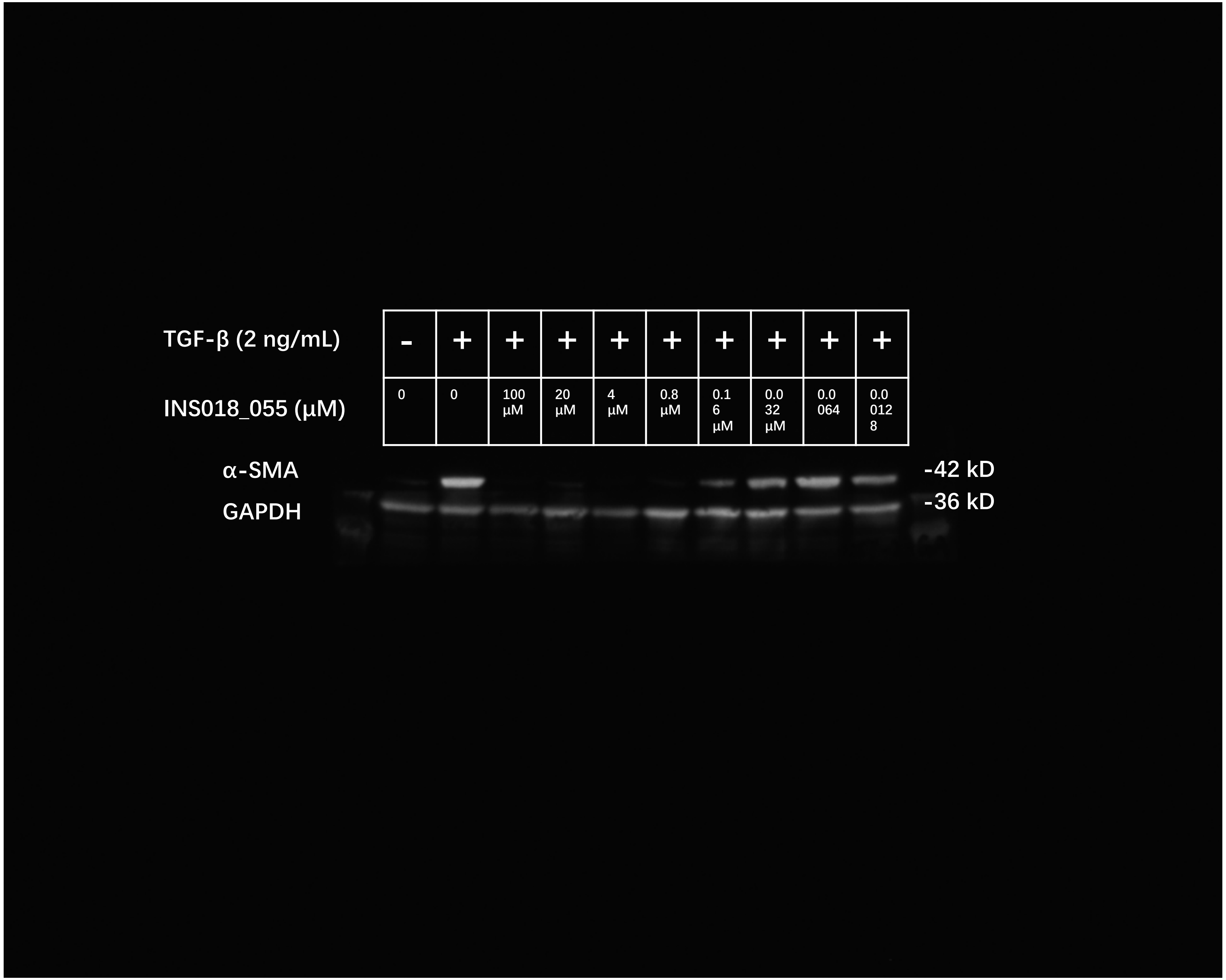

Image shapes reflect membranes cut for multiple staining panels

Insilico Medicine Restricted (Level 3)

Figure 2C MRC-5

Well set -3

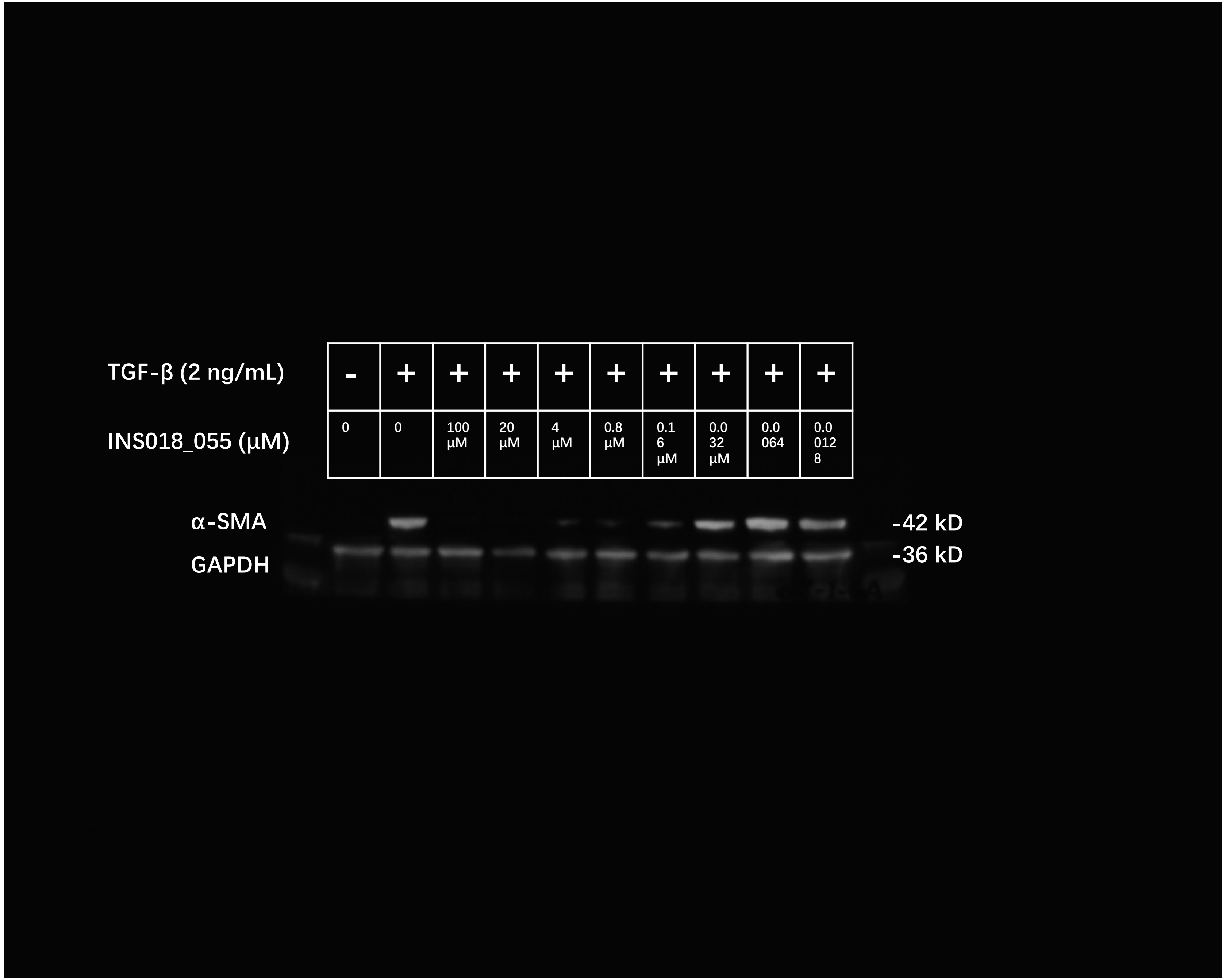

Image shapes reflect membranes cut for multiple staining panels

Insilico Medicine Restricted (Level 3)

Fig 2E

Image shapes reflect membranes cut for multiple staining panels

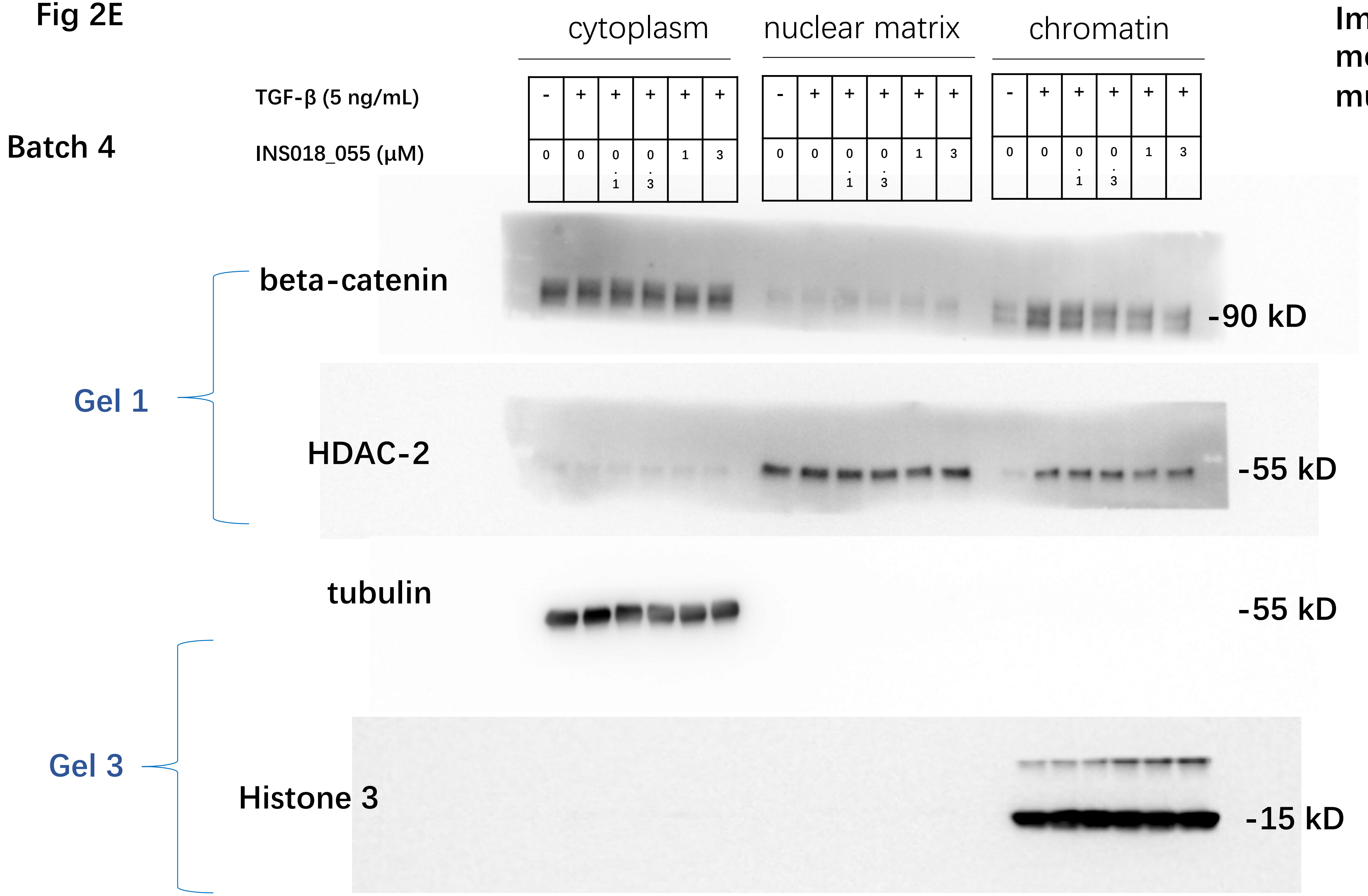

Insilico Medicine Restricted (Level 3)

Fig 2E Batch 2 -Gel1

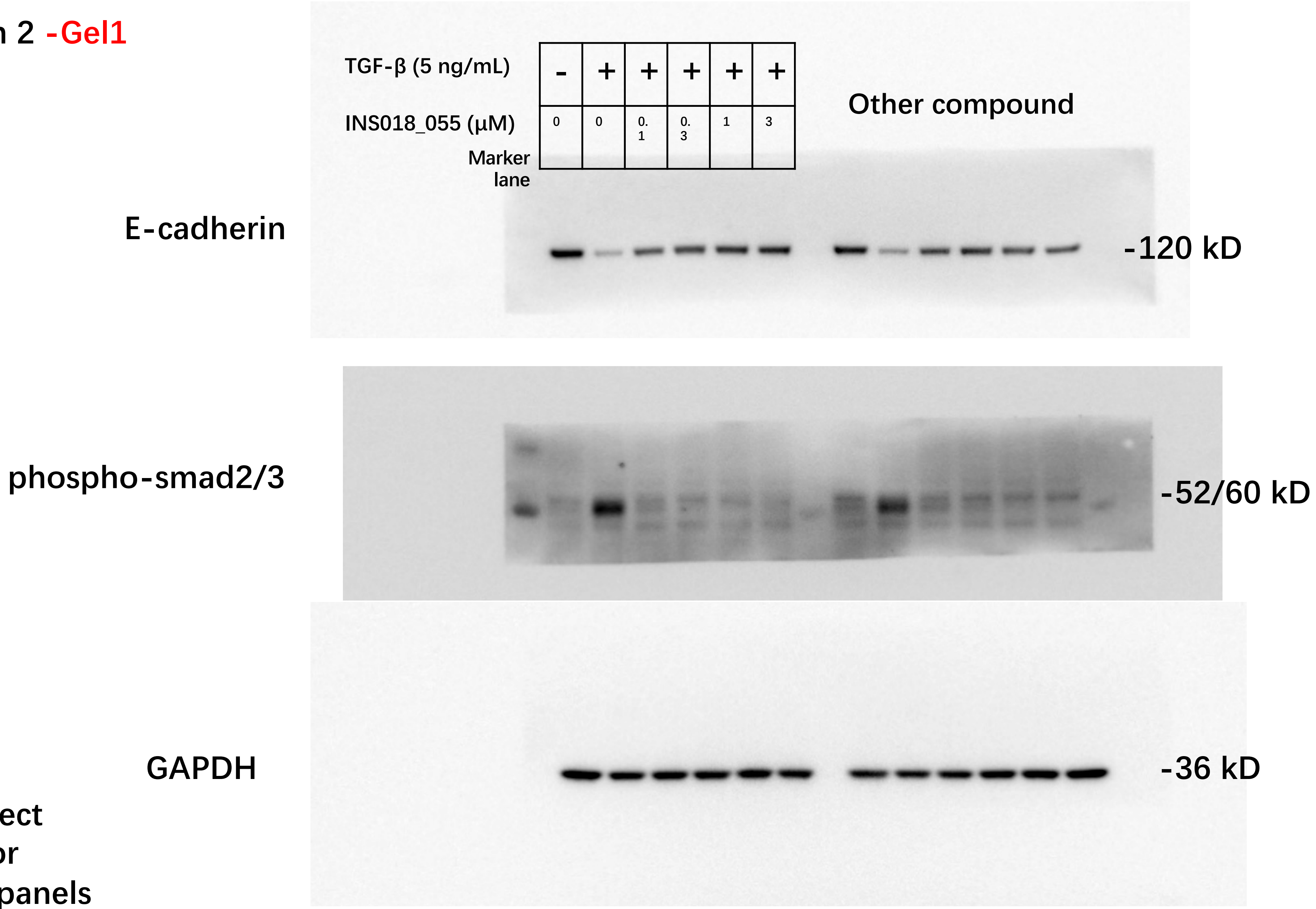

Image shapes reflect membranes cut for multiple staining panels

Insilico Medicine Restricted (Level 3)

Fig 2E Batch 2 –Gel2

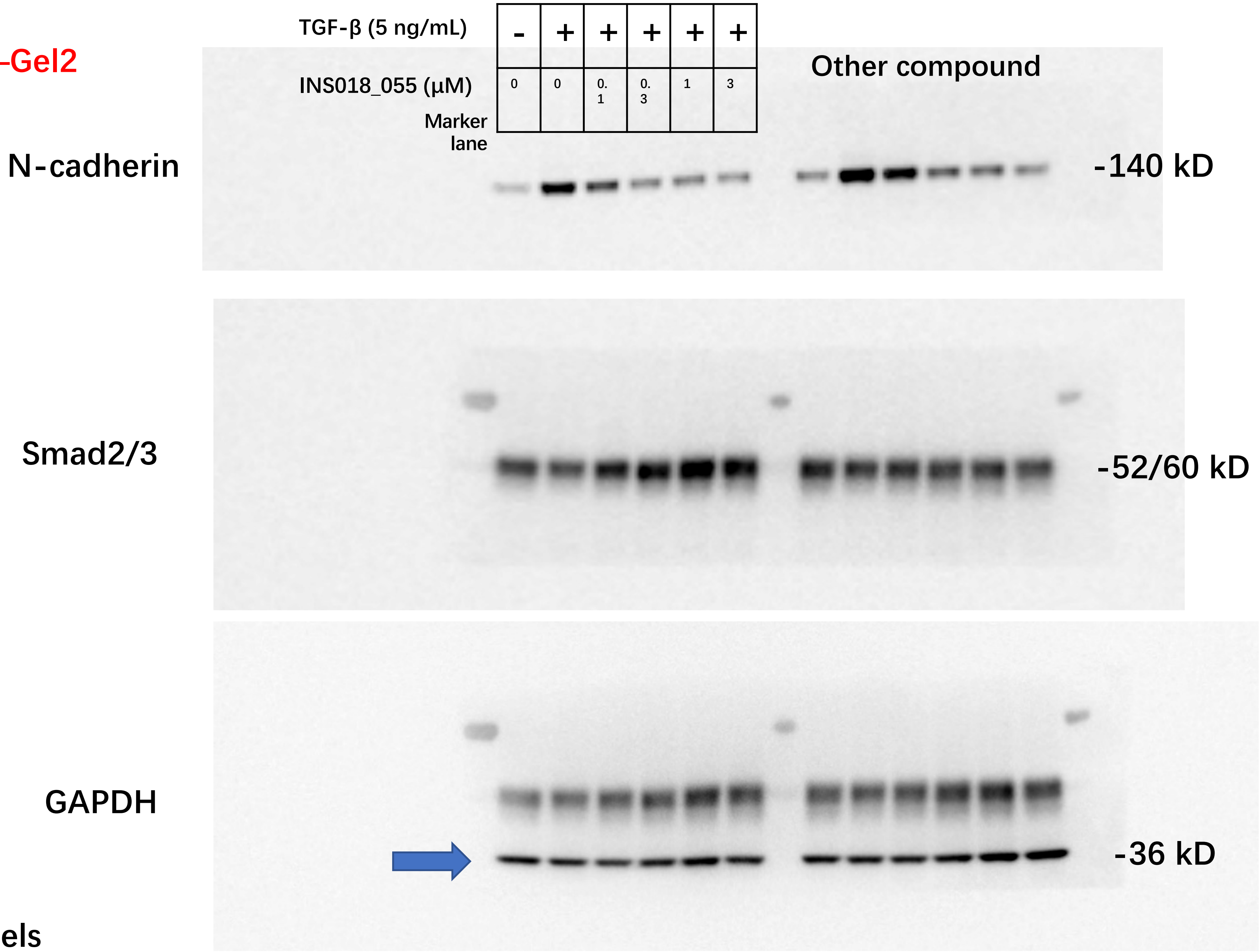

Image shapes reflect membranes cut for multiple staining panels

Insilico Medicine Restricted (Level 3)

Fig 2F Batch 1 – Gel 3

TNF-α (20 ng/mL)	-	-	-	-	-	-	+	+	+	+	+
TGF-β (5 ng/mL)	-	+	+	+	+	+	+	+	+	+	+
INS018_055 (μM)	0	0	0.1	0.3	1	3	0	0.1	0.3	1	3

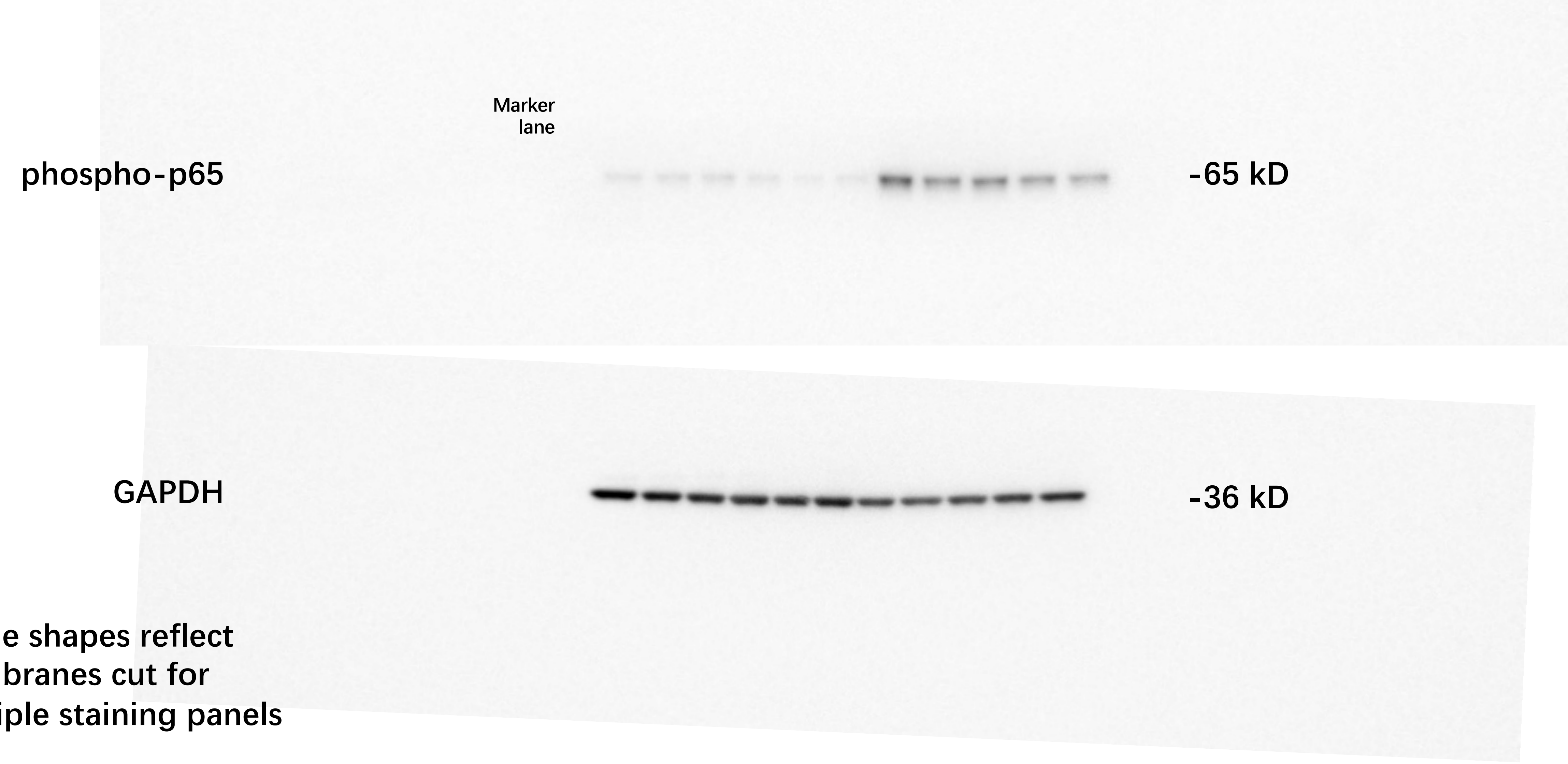

Image shapes reflect membranes cut for multiple staining panels

Insilico Medicine Restricted (Level 3)

Fig 2F Batch 1 – Gel 4

TNF-α (20 ng/mL)	-	-	-	-	-	-	+	+	+	+	+
TGF-β (5 ng/mL)	-	+	+	+	+	+	+	+	+	+	+
INS018_055 (μM)	0	0	0.1	0.3	1	3	0	0.1	0.3	1	3

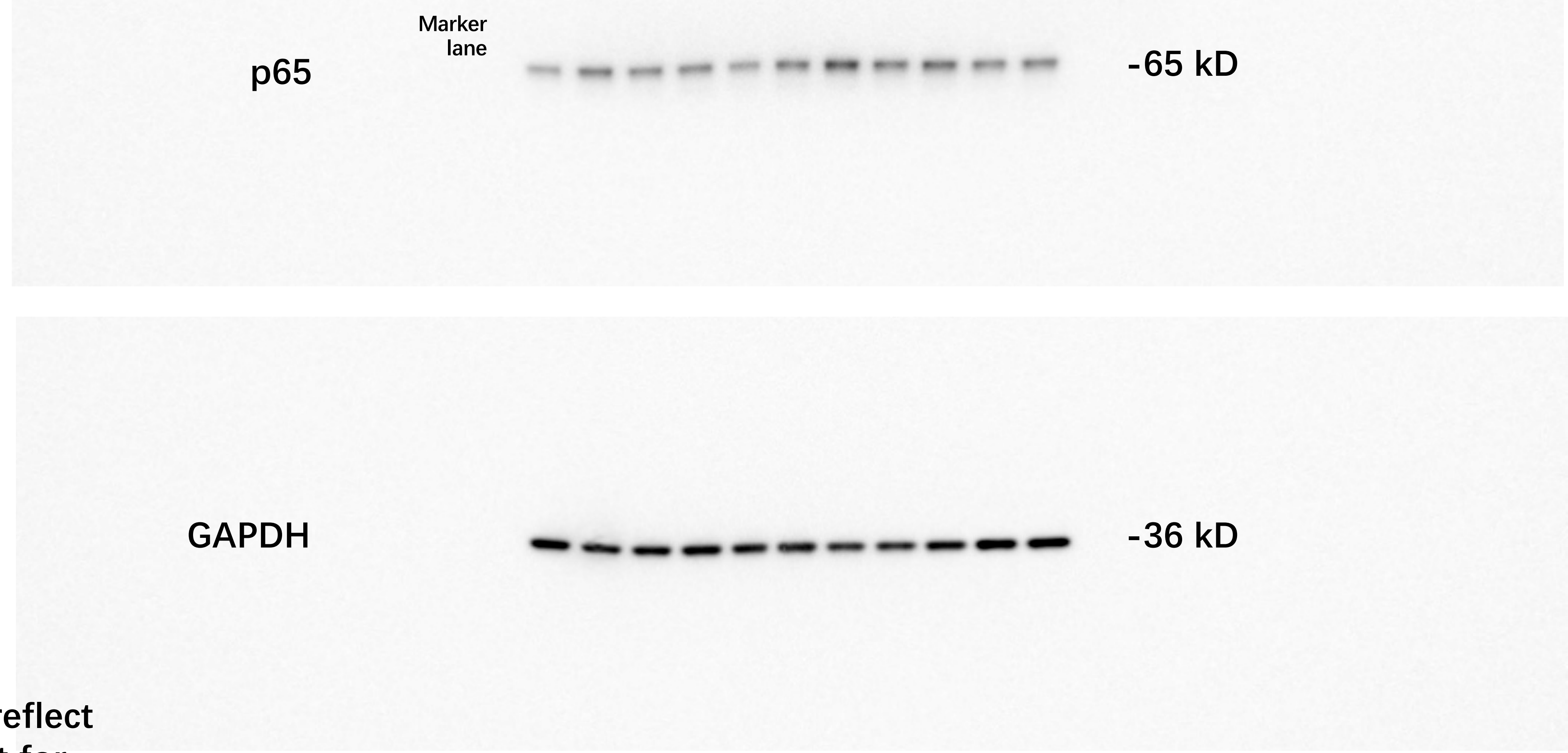

Image shapes reflect membranes cut for multiple staining panels

Insilico Medicine Restricted (Level 3)

Fig 2G

Gel 1

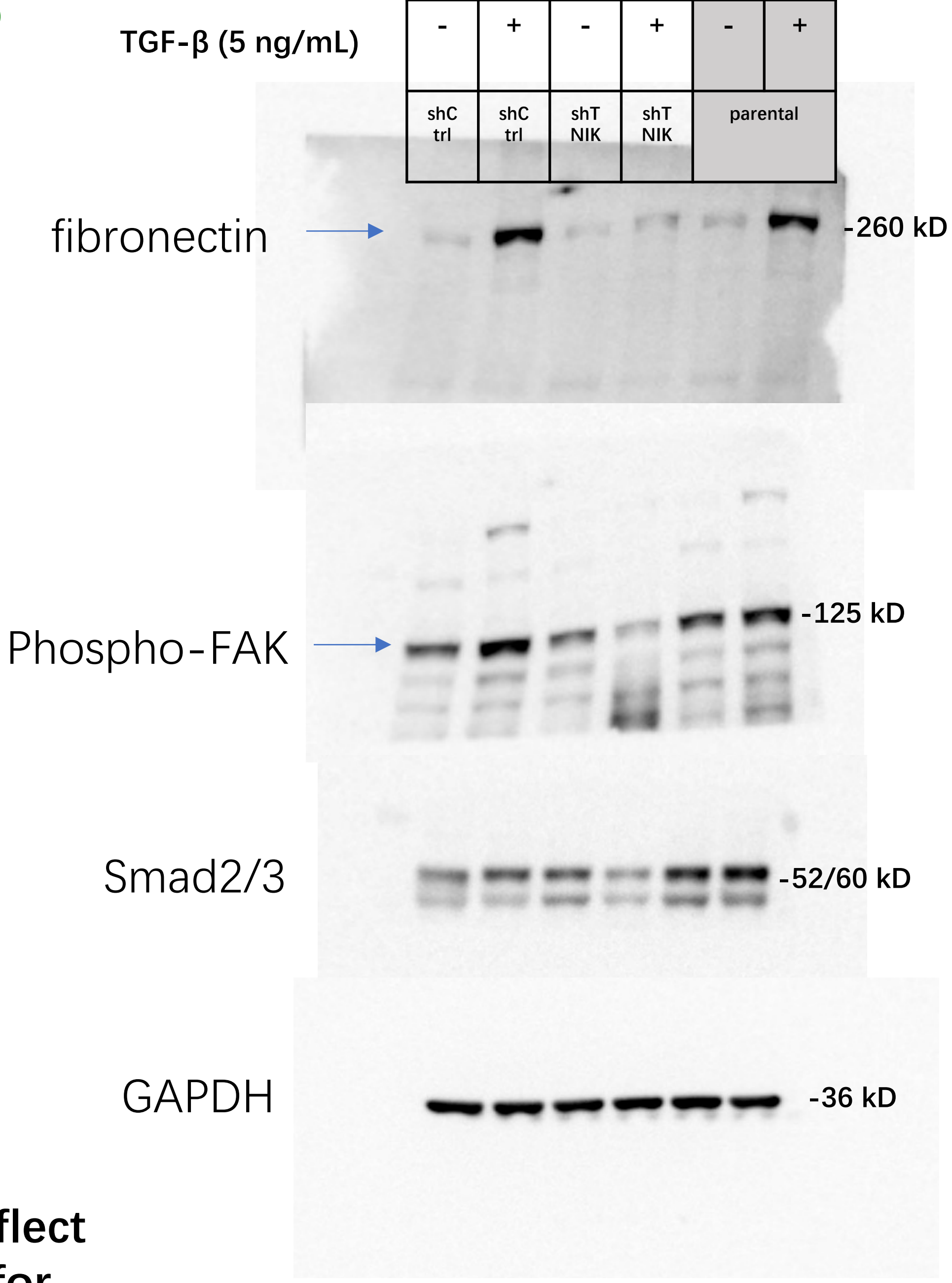

Image shapes reflect membranes cut for multiple staining panels

Insilico Medicine Restricted (Level 3)

Fig 2G

Gel 2

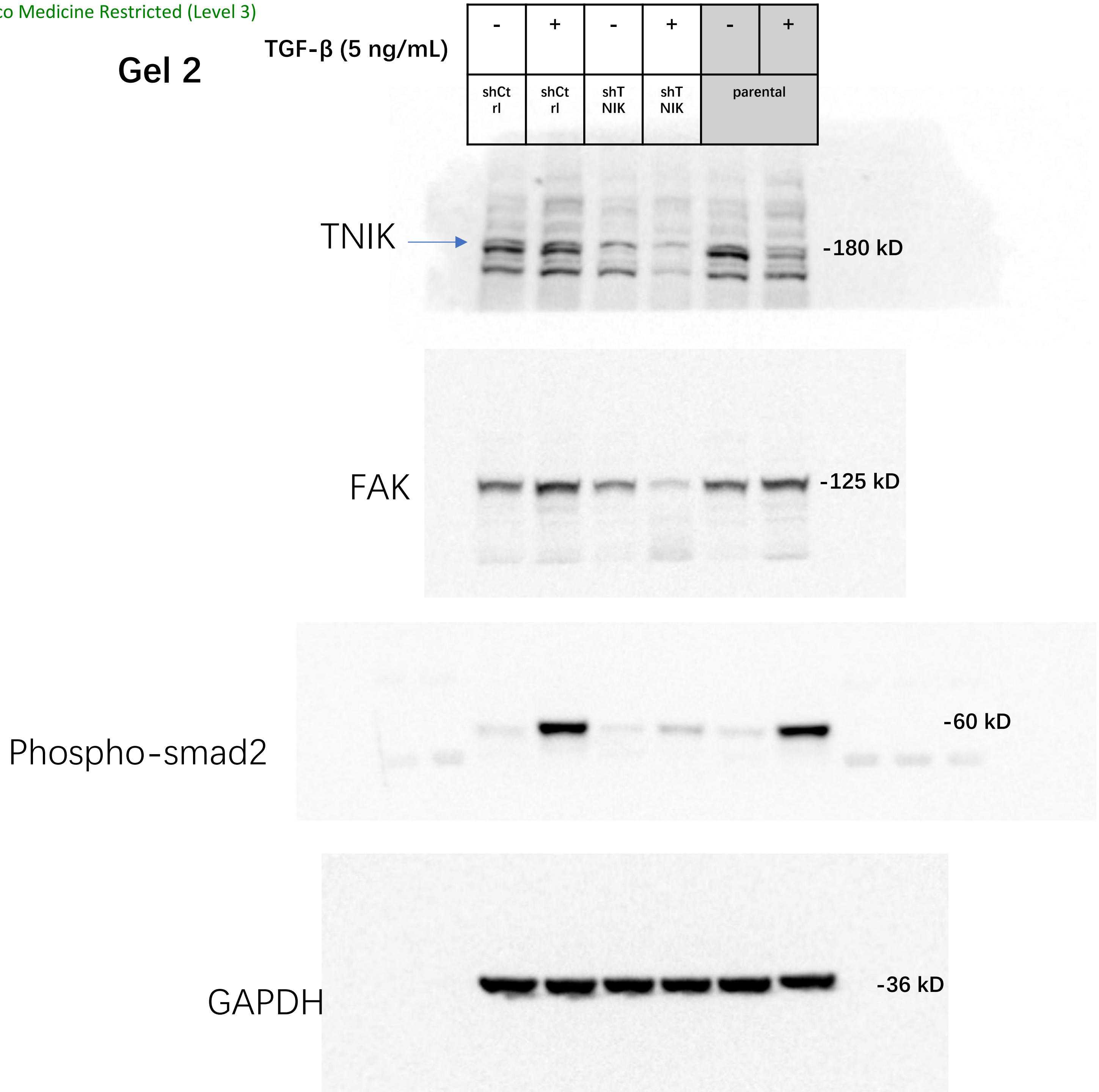

Image shapes reflect membranes cut for multiple staining panels

Insilico Medicine Restricted (Level 3)

Fig 2G

Gel 3

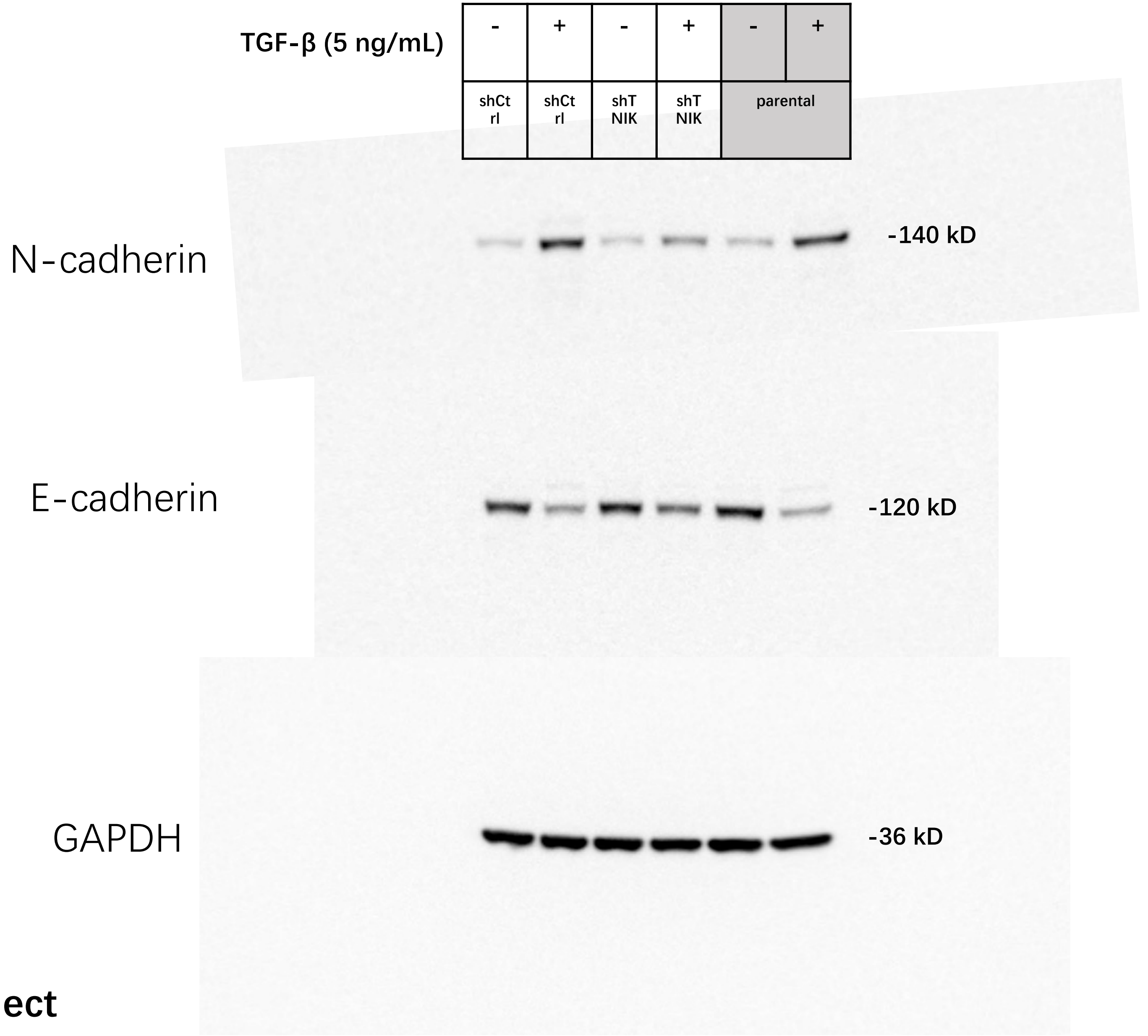

Image shapes reflect membranes cut for multiple staining panels

Insilico Medicine Restricted (Level 3)

Figure 5A HK-2

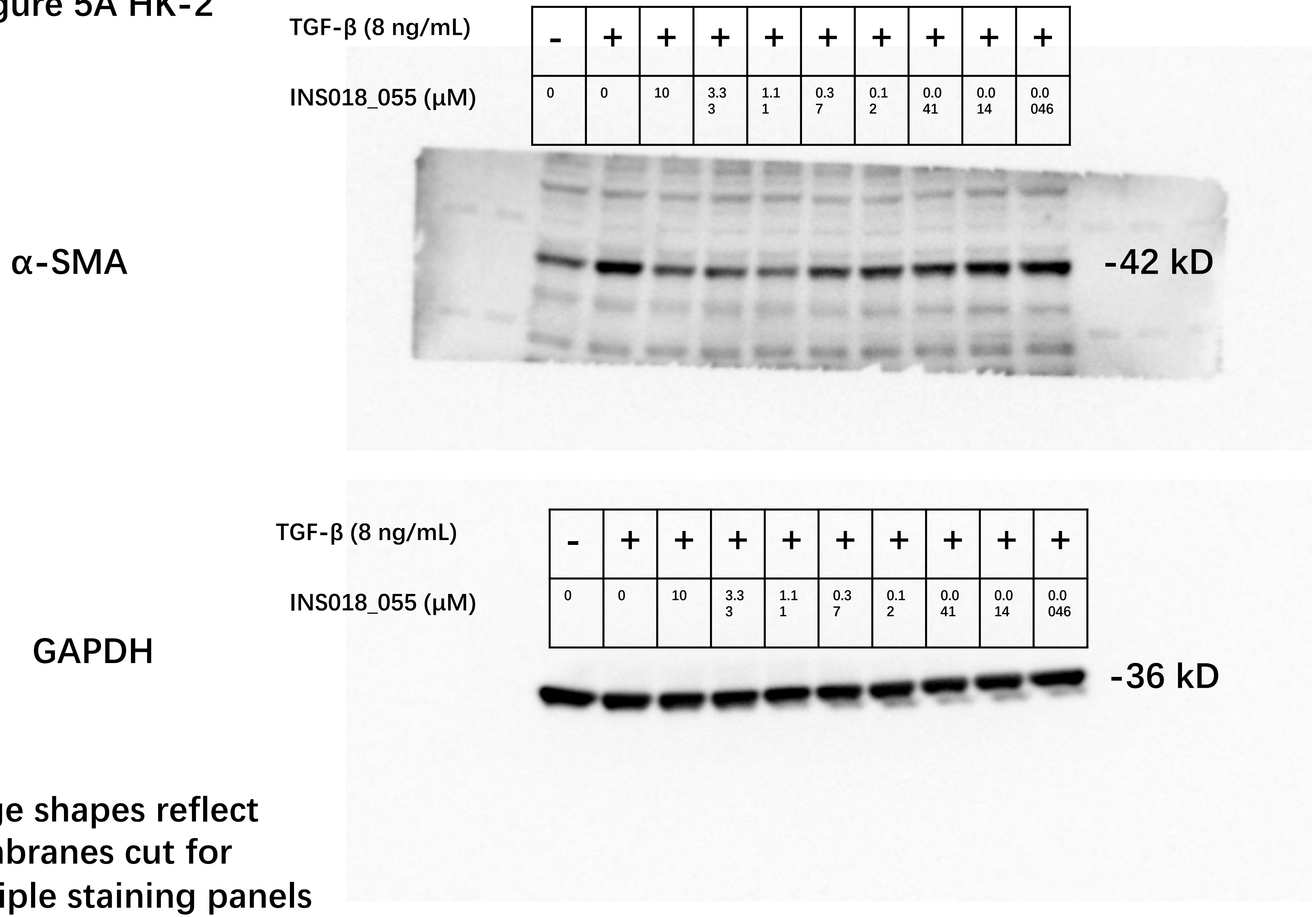

Image shapes reflect membranes cut for multiple staining panels

Insilico Medicine Restricted (Level 3)

Extended Data Fig 5B

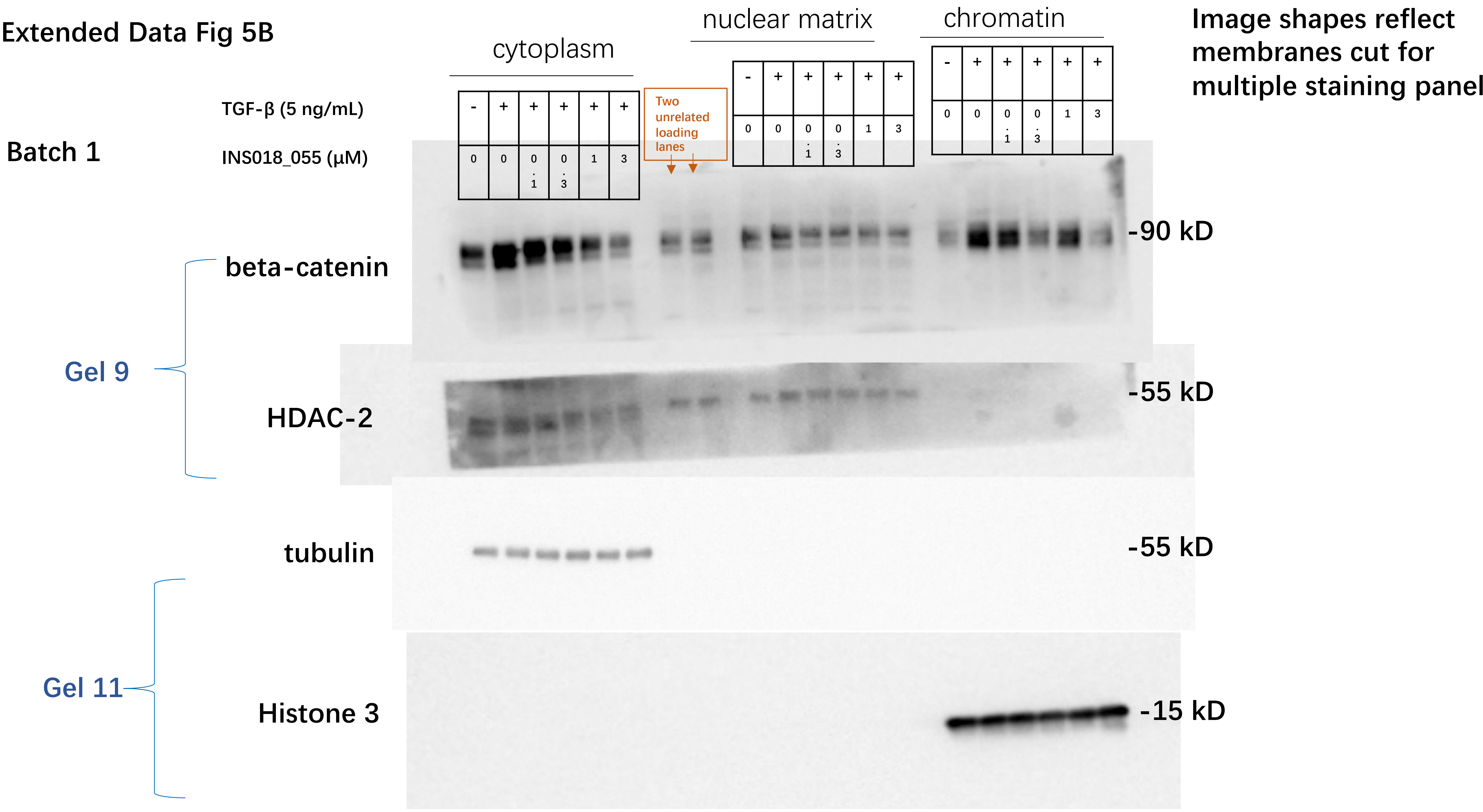

Insilico Medicine Restricted (Level 3)

Extended Data Fig 5B

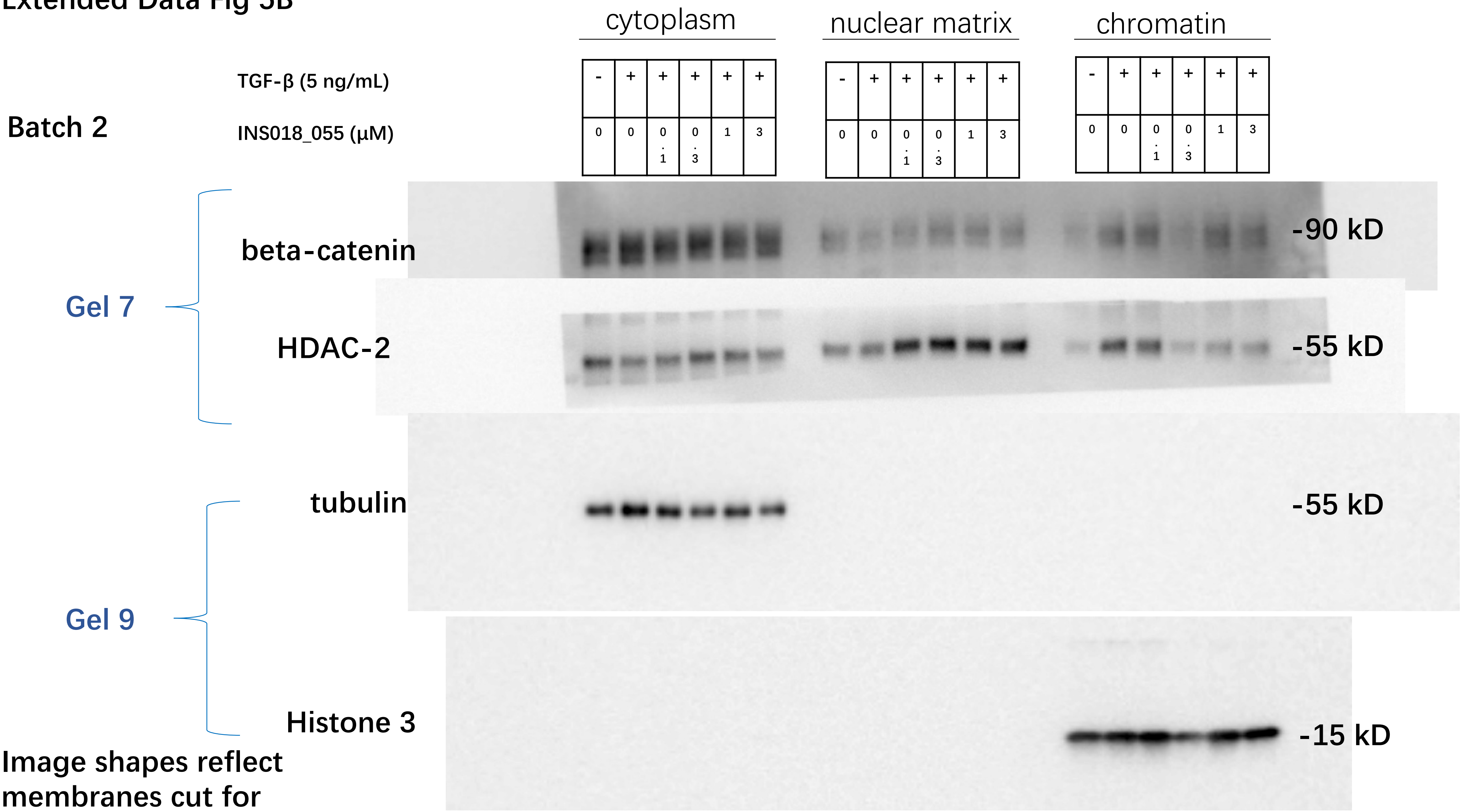

Image shapes reflect membranes cut for multiple staining panels

Insilico Medicine Restricted (Level 3)

Extended Data Fig 5B

Batch 1 -Gel1

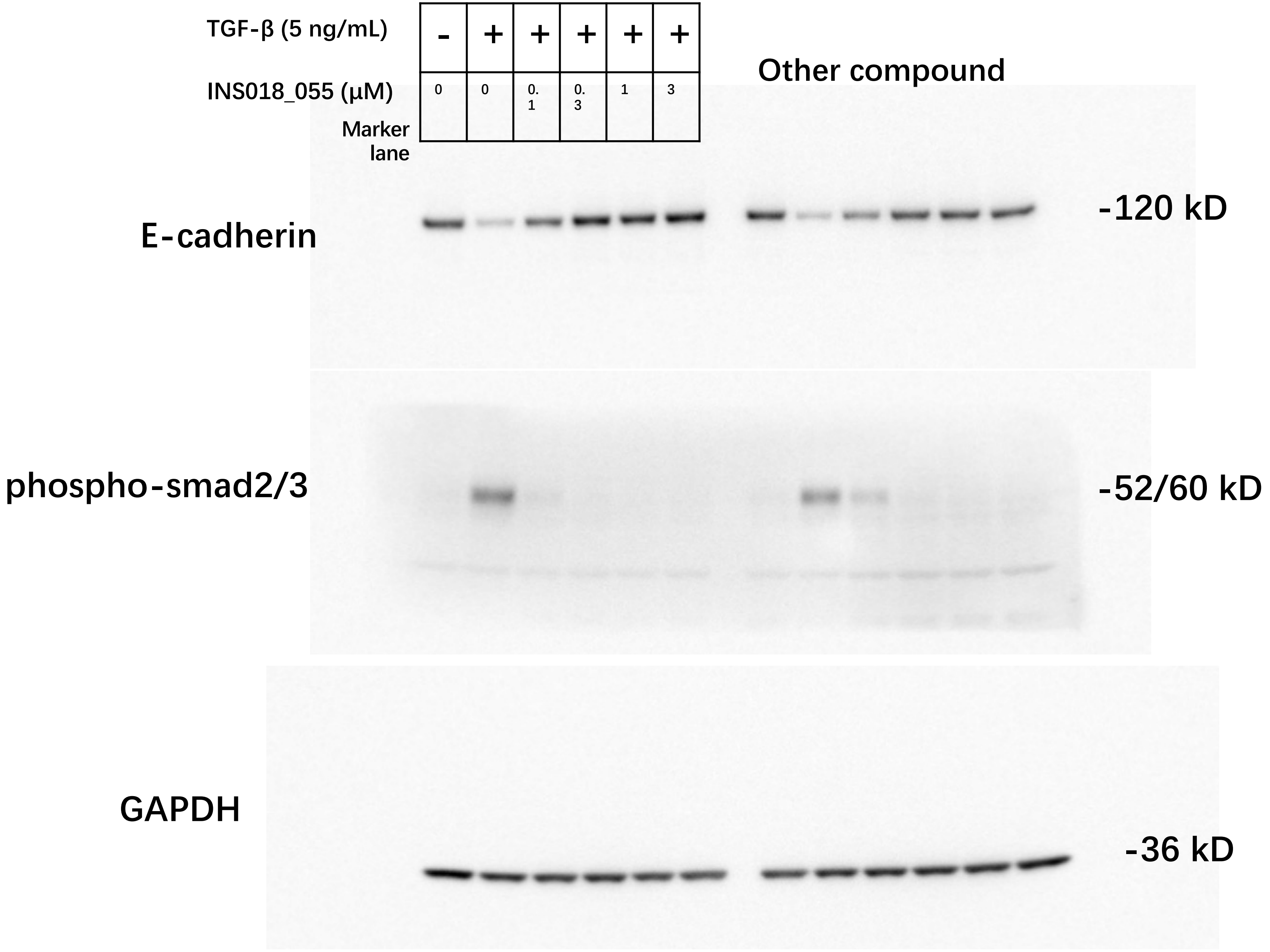

Image shapes reflect membranes cut for multiple staining panels

Insilico Medicine Restricted (Level 3)

Extended Data Fig 5B

Batch 2 –Gel2

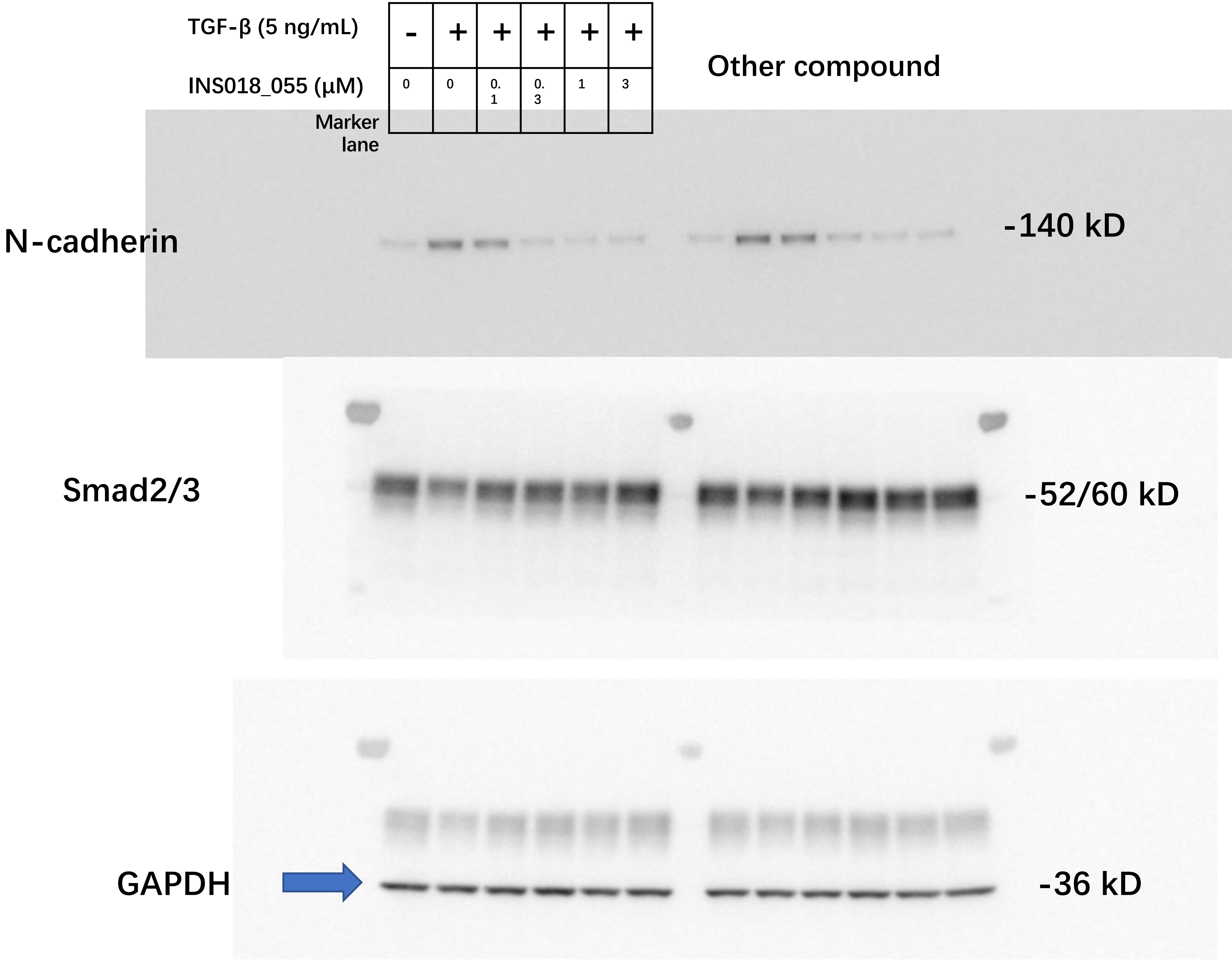

Image shapes reflect membranes cut for multiple staining panels

Insilico Medicine Restricted (Level 3)

Extended Data Fig 5B

Batch 3 -Gel1

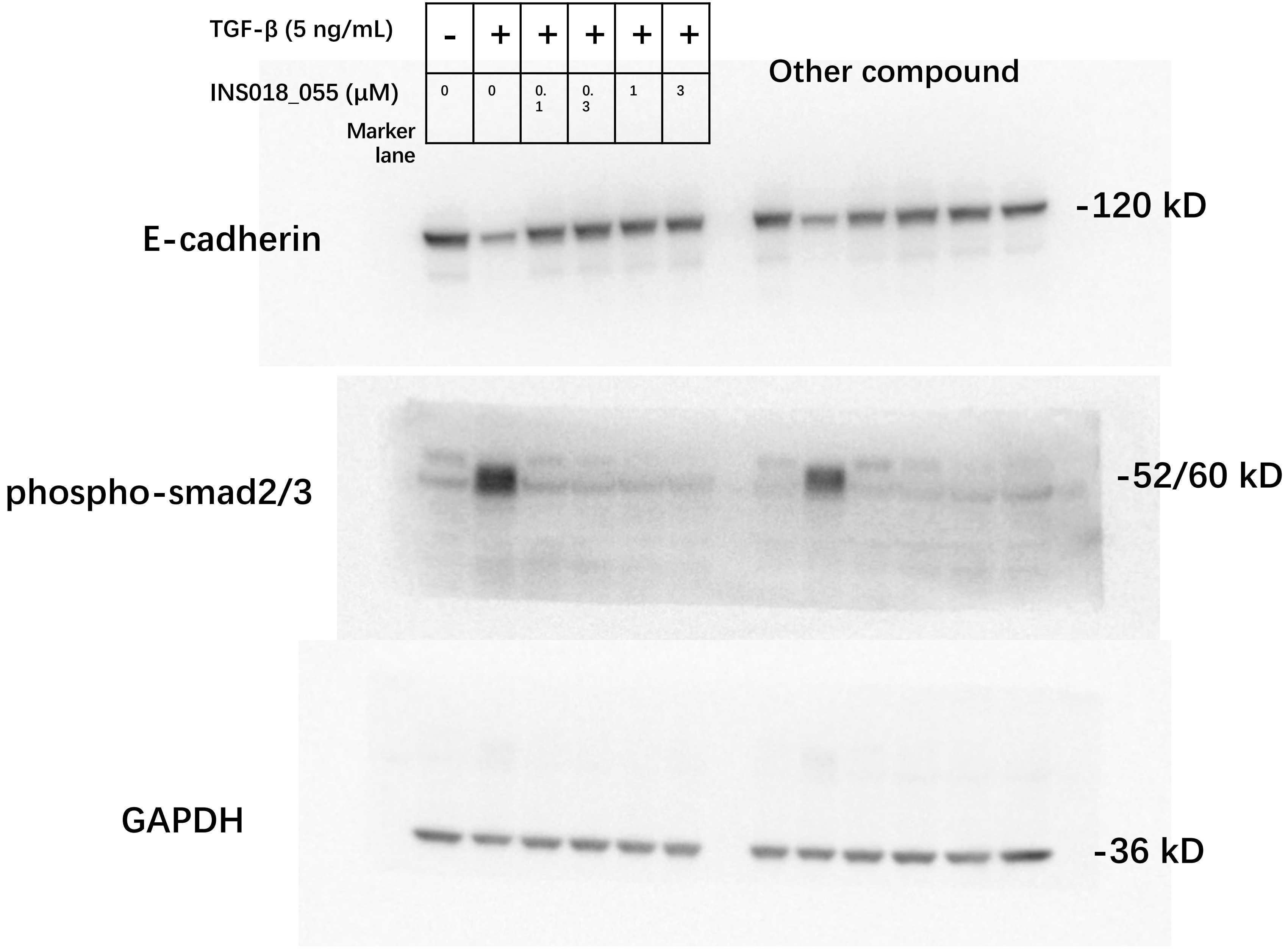

Image shapes reflect membranes cut for multiple staining panels

Insilico Medicine Restricted (Level 3)

Extended Data Fig 5B

Batch 3 –Gel2

TGF-β (5 ng/mL)	-	+	+	+	+	+
INS018_055 (μM)	0	0	0.1	0.3	1	3
Marker lane						

Other compound

N-cadherin

GAPDH

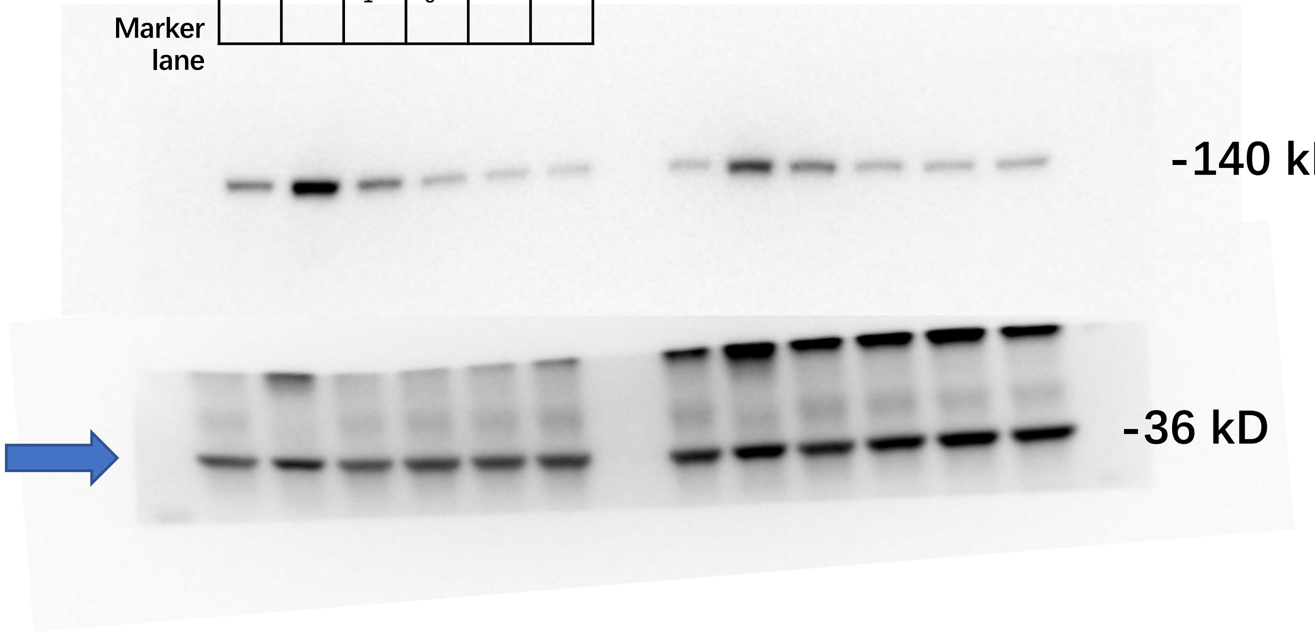

-140 kD

-36 kD

Image shapes reflect membranes cut for multiple staining panels

Insilico Medicine Restricted (Level 3)

Extended Data Fig 5B

Batch 3 –a re-run gel

TGF-β (5 ng/mL)	-	+	+	+	+	+
INS018_055 (μM)	0	0	0.1	0.3	1	3

Marker lane

Other compound

Smad2/3

GAPDH

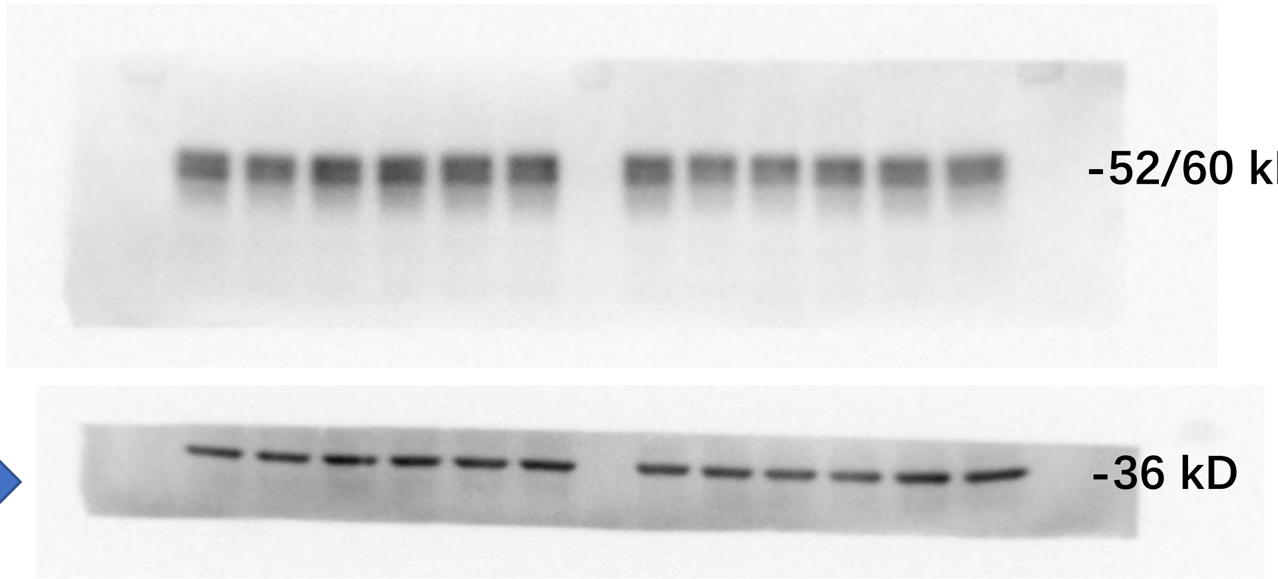

-52/60 kD

-36 kD

Image shapes reflect membranes cut for multiple staining panels

Insilico Medicine Restricted (Level 3)

Extended Data Fig 5B

Batch 2 – Gel 5

TNF-α (20 ng/mL)	-	-	-	-	-	-	+	+	+	+	+
TGF-β (5 ng/mL)	-	+	+	+	+	+	+	+	+	+	+
INS018_055 (μM)	0	0	0.1	0.3	1	3	0	0.1	0.3	1	3

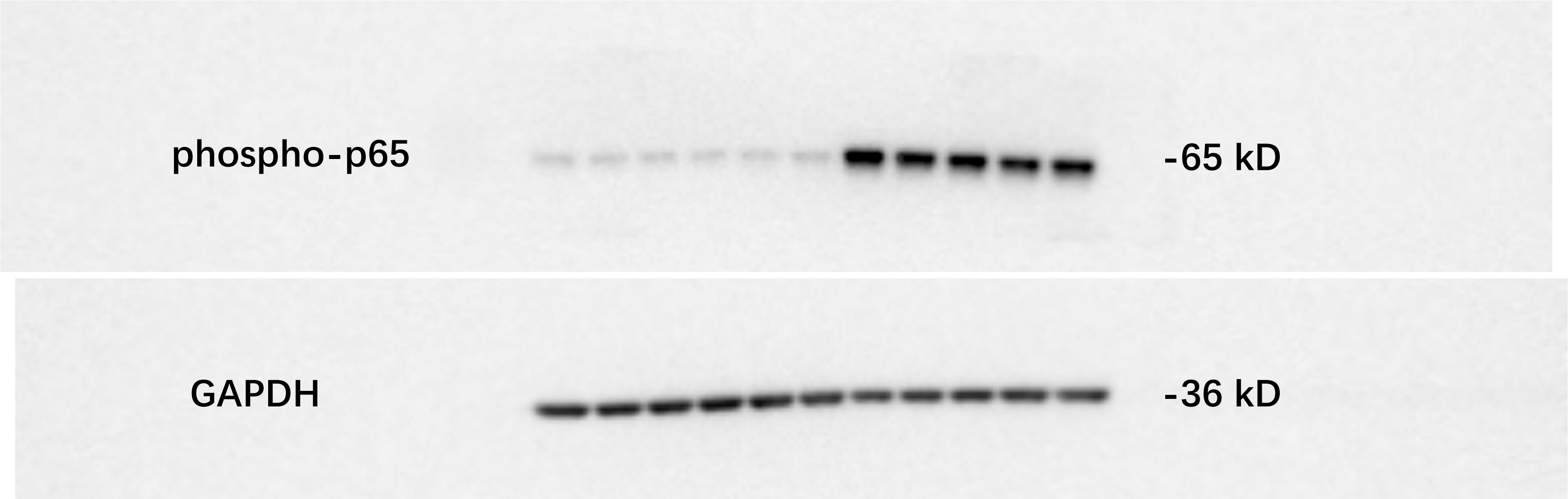

Image shapes reflect membranes cut for multiple staining panels

Insilico Medicine Restricted (Level 3)

Extended Data Fig 5B

Batch 2 – Gel 6

TNF-α (20 ng/mL)	-	-	-	-	-	-	+	+	+	+	+
TGF-β (5 ng/mL)	-	+	+	+	+	+	+	+	+	+	+
INS018_055 (μM)	0	0	0.1	0.3	1	3	0	0.1	0.3	1	3

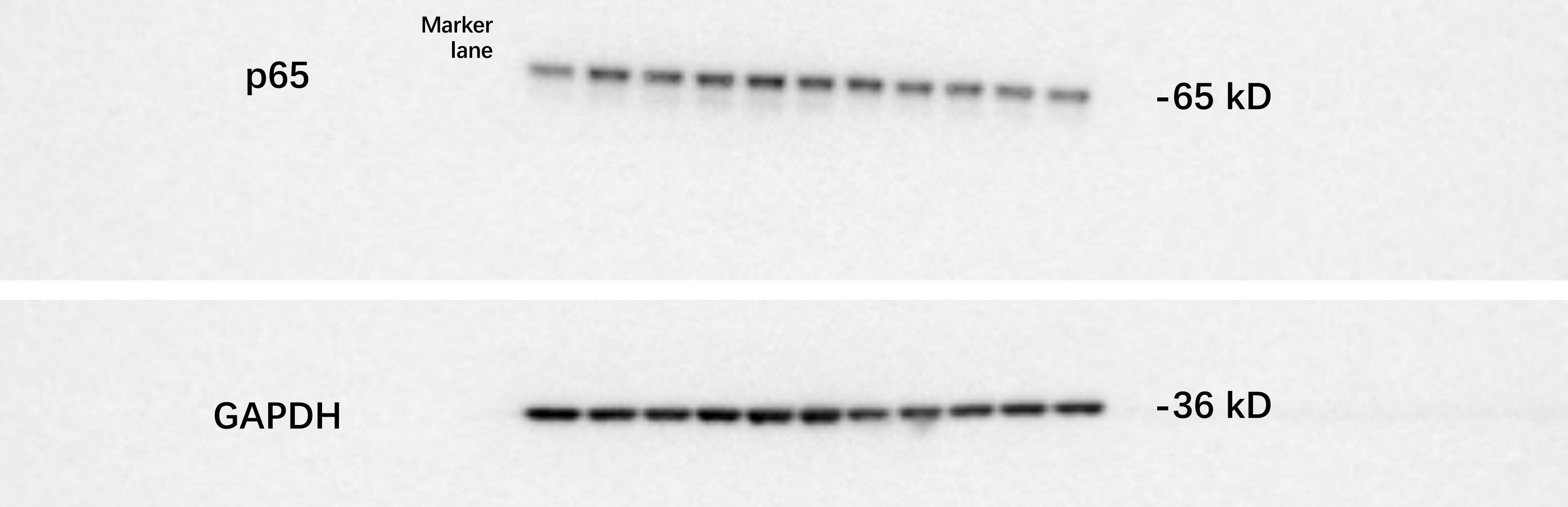

Image shapes reflect membranes cut for multiple staining panels

Insilico Medicine Restricted (Level 3)

Extended Data Fig 5B

Batch 3 – Gel 4

TNF-α (20 ng/mL)	-	-	-	-	-	-	+	+	+	+	+
TGF-β (5 ng/mL)	-	+	+	+	+	+	+	+	+	+	+
INS018_055 (μM)	0	0	0.1	0.3	1	3	0	0.1	0.3	1	3

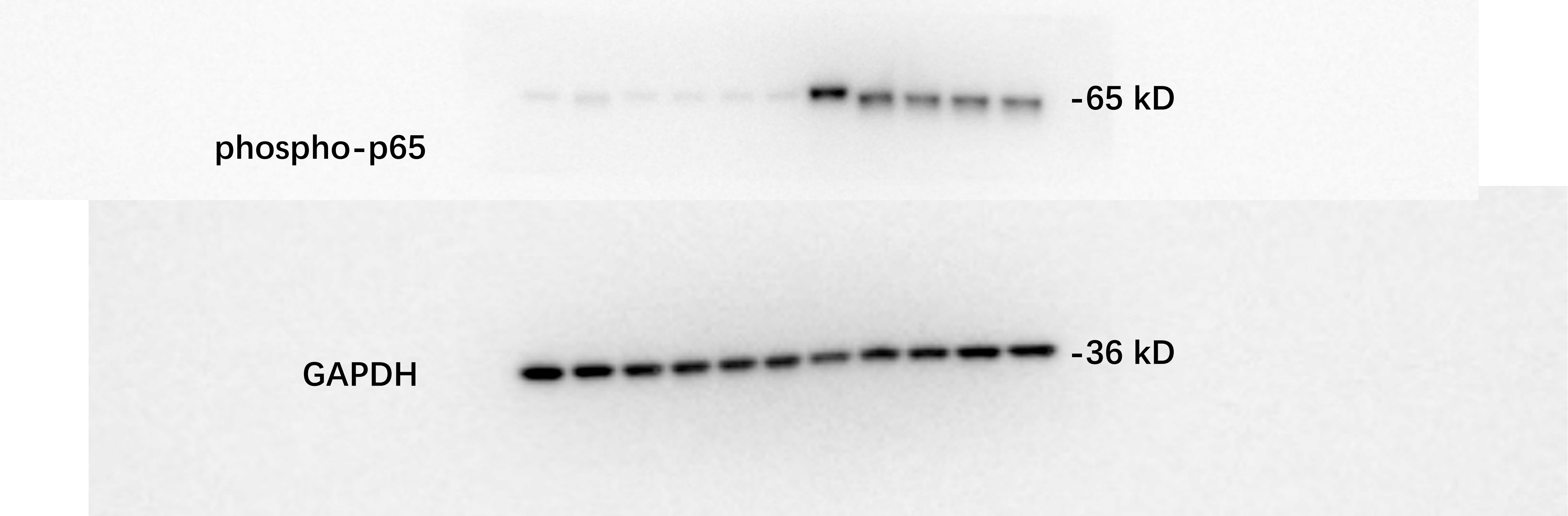

Image shapes reflect membranes cut for multiple staining panels

Insilico Medicine Restricted (Level 3)

Extended Data Fig 5B

Batch 3 – Gel 3

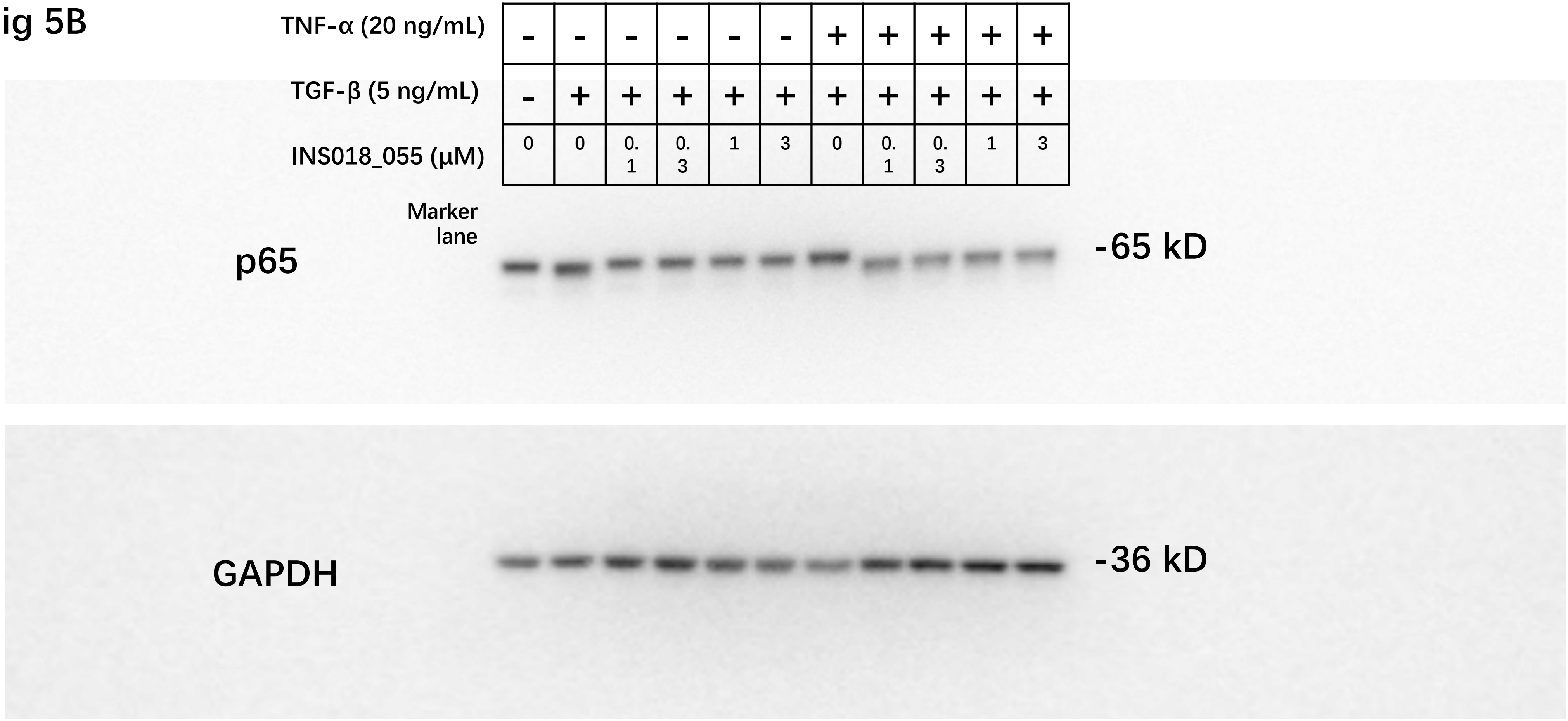

Image shapes reflect membranes cut for multiple staining panels

Insilico Medicine Restricted (Level 3)

Extended Data Fig 6B

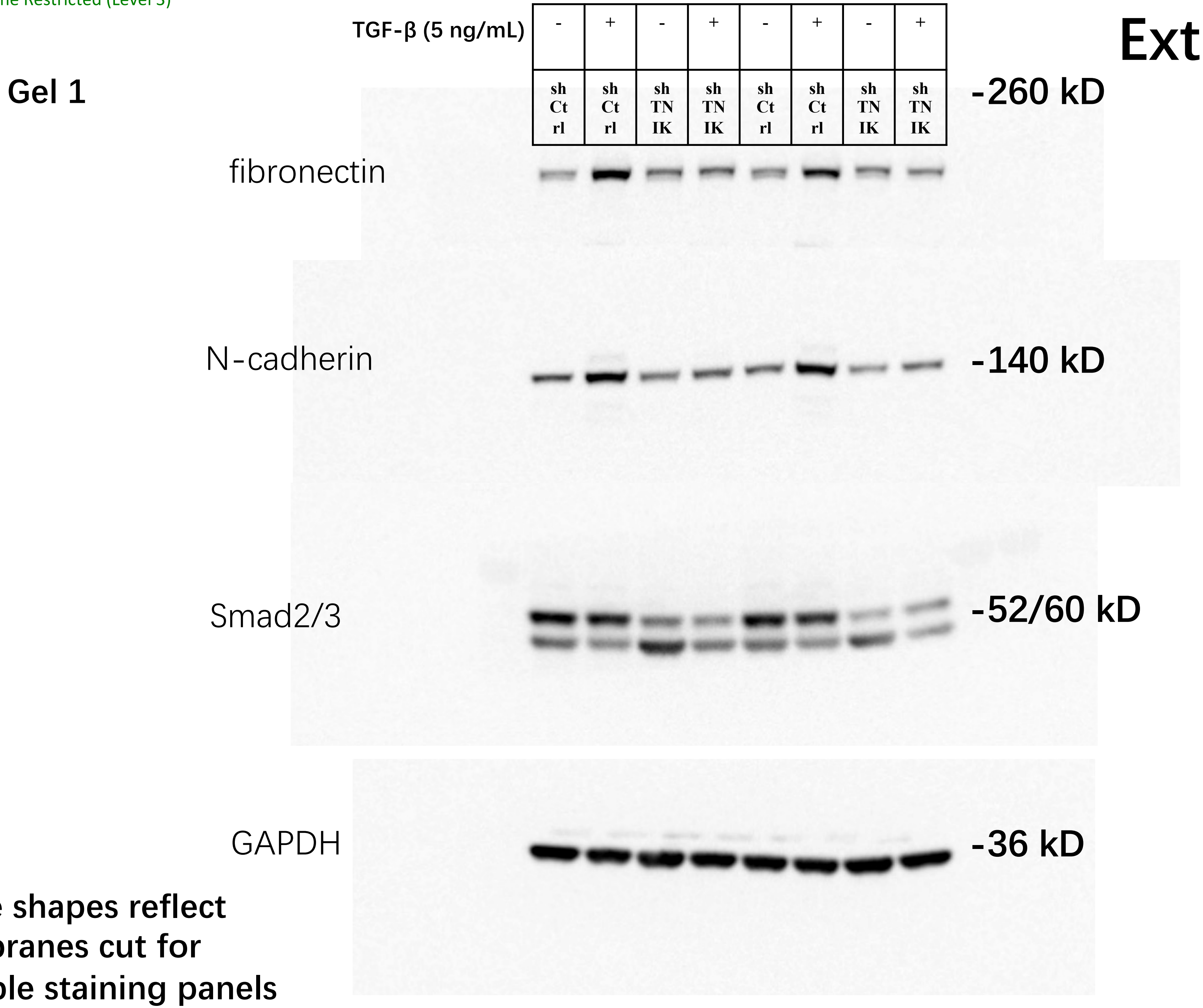

Image shapes reflect membranes cut for multiple staining panels

Insilico Medicine Restricted (Level 3)

Extended Data Fig 6B

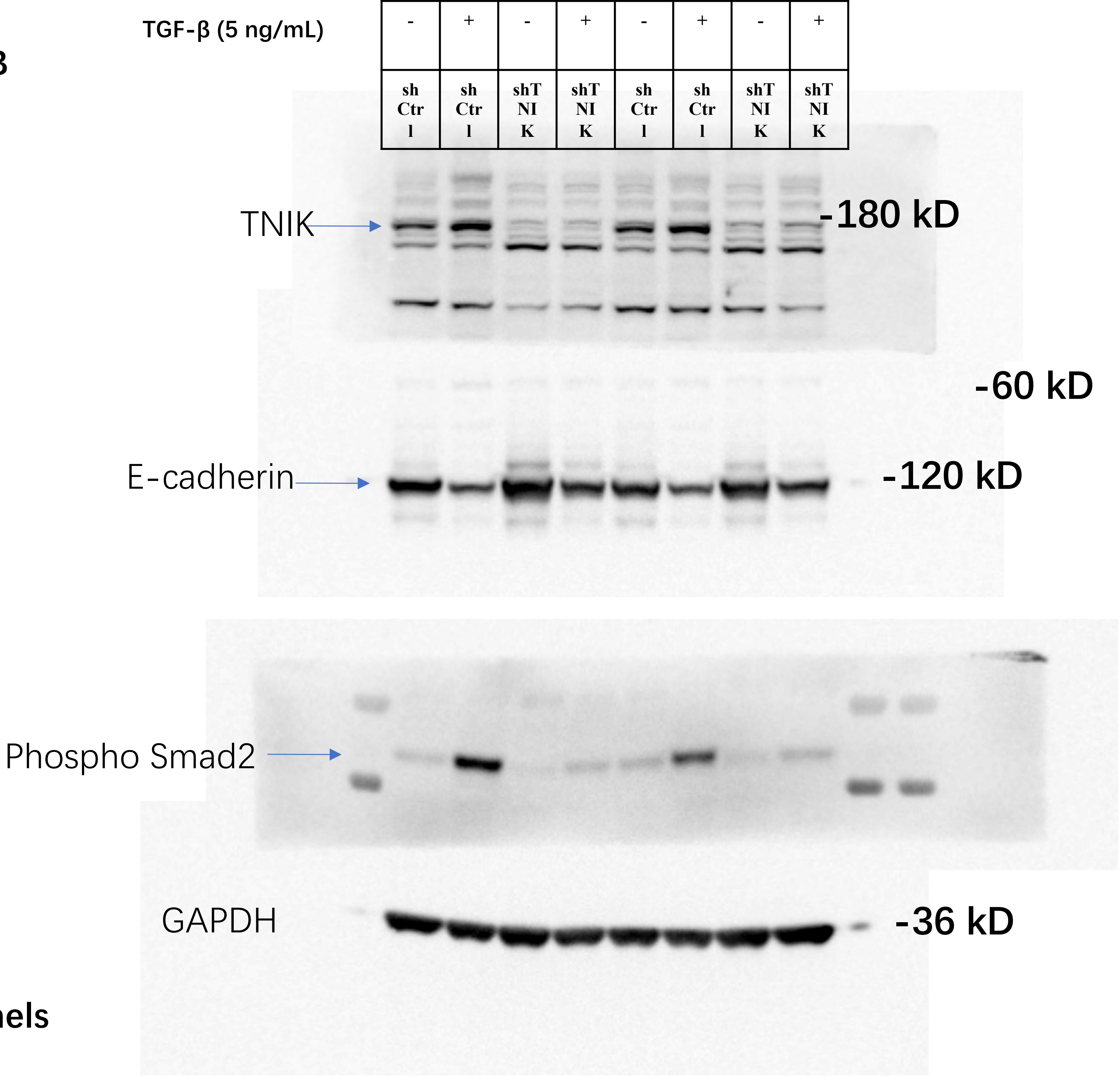

Image shapes reflect membranes cut for multiple staining panels
